# Discovery of TNG462: A Highly Potent and Selective MTA-Cooperative PRMT5 Inhibitor to Target Cancers with *MTAP* Deletion

**DOI:** 10.1021/acs.jmedchem.4c03067

**Published:** 2025-03-04

**Authors:** Kevin M. Cottrell, Kimberly J. Briggs, Alice Tsai, Matthew R. Tonini, Douglas A. Whittington, Shanzhong Gong, Colin Liang, Patrick McCarren, Minjie Zhang, Wenhai Zhang, Alan Huang, John P. Maxwell

**Affiliations:** Tango Therapeutics, Boston, Massachusetts 02215, United States

## Abstract

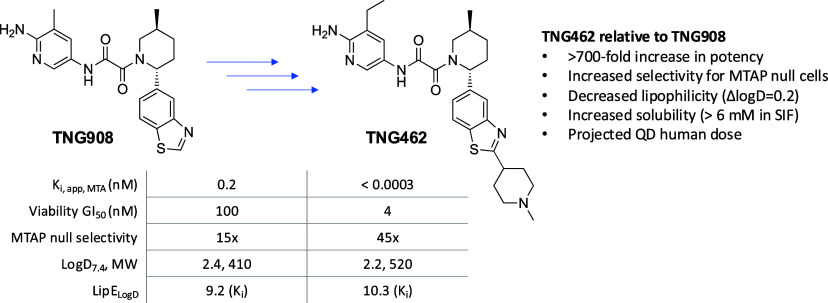

The gene encoding for MTAP is one of the most commonly deleted genes in cancer, occurring in approximately 10–15% of all human cancer. We have previously described the discovery of TNG908, a brain-penetrant clinical-stage compound that selectively targets *MTAP*-deleted cancer cells by binding to and inhibiting PRMT5 cooperatively with MTA, which is present in elevated concentrations in *MTAP*-deleted cells. Herein we describe the discovery of TNG462, a more potent and selective MTA-cooperative PRMT5 inhibitor with improved DMPK properties that is selective for *MTAP*-deleted cancers and is currently in Phase I/II clinical trials.

## Introduction

PRMT5 plays an important role in the regulation of diverse cellular processes via dimethylation of target proteins involved in splicing regulation, cell cycle progression, apoptosis, the DNA-damage response, and other functions.^1−3^ Due to its involvement in these critical functions, PRMT5 is considered an essential gene. It has been shown that PRMT5 inhibition by small molecules can selectively kill cancer cells with homozygous deletion of the *MTAP* gene if the inhibitors leverage the consequence of *MTAP* deletion, namely, accumulation of the substrate of the MTAP protein, methylthioadenosine (MTA).^4−6^ MTA is a weak, endogenous inhibitor of PRMT5 that binds competitively with *S*-adenosylmethionine (SAM).^7^ Compounds that bind PRMT5 cooperatively with MTA, and not with SAM, can selectively inhibit PRMT5 in *MTAP*-deleted (MTAP-null) cells and kill those cells while sparing normal (MTAP WT) cells, since normal cells have functioning MTAP and therefore low levels of MTA. Targeting MTAP-null cancer cells via selective inhibition of the PRMT5·MTA complex can improve tolerability relative to nonselective PRMT5 inhibitors^8−11^ by limiting inhibition of PRMT5 in normal cells. Given that *MTAP* deletion occurs in approximately 10–15% of all human cancer, and that there are at least 15 cancer types where the frequency of *MTAP* loss occurs in ≥10% of patients,^7,12^ molecules that selectively kill MTAP-null cancer cells provide a striking opportunity to deliver a targeted treatment to a significant patient population.

**Figure 1:**
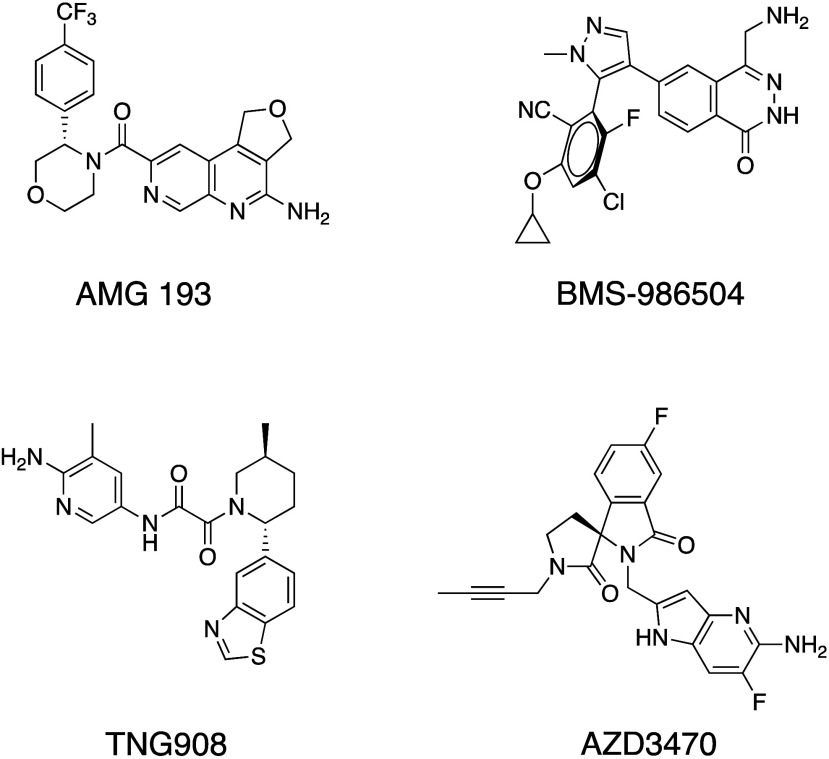
Clinical-stage MTA-cooperative PRMT5 inhibitors AMG 193, BMS-986504 (previously MRTX1719), TNG908, and AZD3470.

There are multiple structurally distinct clinical stage compounds that are MTA-cooperative and target *MTAP*-deleted cancers. These include AMG 193, BMS-986504 (previously MRTX1719), and TNG908, which have demonstrated activity and improved tolerability compared to the first generation PRMT5 inhibitors in early reports,^13,14^ and AZD3470 which has not reported clinical data as of the time publication of this report (Figure 1).

TNG908 is a blood–brain barrier (BBB)-penetrant compound with HAP1 MTAP-null viability GI_50_ = 100 nM and 15-fold selectivity for MTAP WT cells.^4,15^ Upon the selection of TNG908 as a development candidate we set out to discover molecules with greater than 10-fold improvement in viability potency (HAP1 MTAP-null GI_50_ < 10 nM), increased selectivity (>30-fold vs MTAP WT cells), and a longer predicted human *T*_1/2_ sufficient to enable QD dosing while minimizing the *C*_max_/*C*_trough_ ratio to maintain at least GI_90_ coverage of target at trough, which we believed would maximize efficacy *in vivo*. Brain penetration was not a requirement for this discovery effort and restriction to peripheral tissues provided diversity in the portfolio. Reports on a third generation, brain penetrant MTA-cooperative PRMT5 inhibitor, TNG456, will be shared in due course.

To this end, we have discovered **TNG462**, a highly potent and selective small molecule PRMT5 inhibitor that acts via an MTA-cooperative mechanism and has a long predicted human *in vivo* half-life. **TNG462** has a PRMT5·MTA *K*_i_ ≤ 300 fM, HAP1 MTAP-null cellular SDMA IC_50_ = 800 pM and viability GI_50_ = 4 nM, an average 45-fold selectivity against MTAP WT cells, displays consistent pharmacokinetic properties across preclinical species with a predicted *T*_1/2_ in humans >24 h, and is in Phase 1/2 clinical trials for *MTAP*-deleted cancers (NCT05732831).

## Medicinal Chemistry

**Figure 2:**
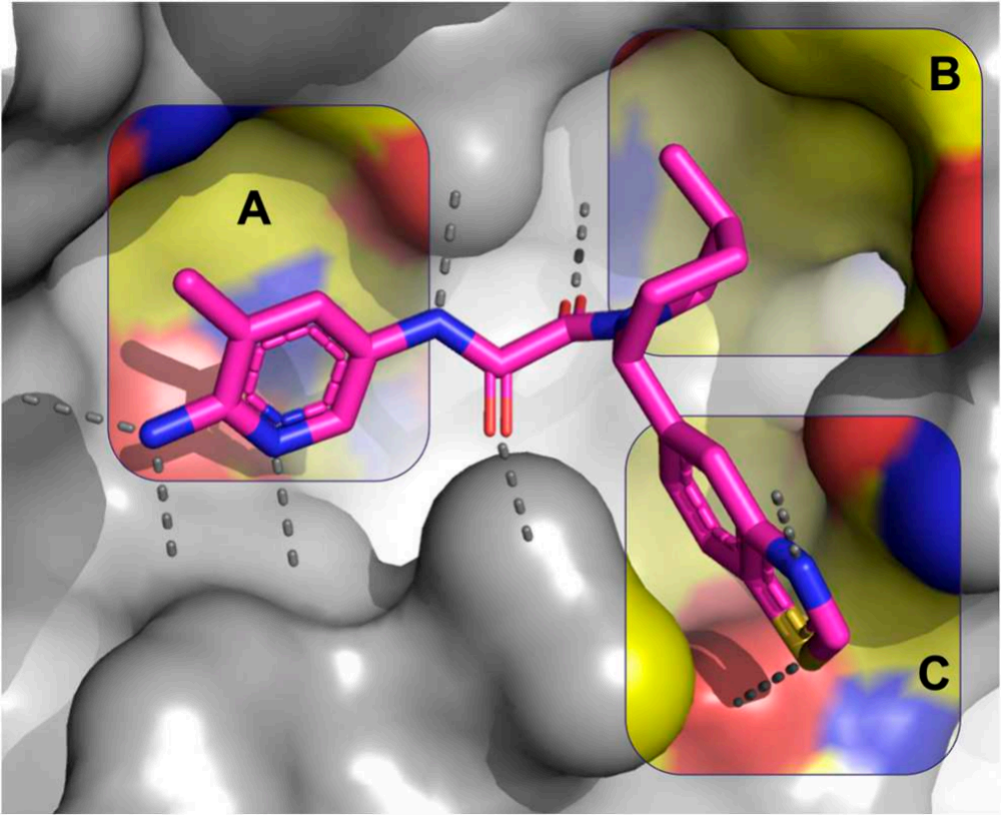
X-ray crystal structure of TNG908 (magenta) bound to PRMT5·MTA (PDB entry 8VEY) with areas of medicinal chemistry focus highlighted: (A) aminopyridine positioned near MTA, (B) small pocket adjacent to the 4-position of the piperidine ring, (C) polar residues close to the benzothiazole ring.

Leveraging the SAR we had generated in the oxamide series that led to TNG908, we sought to discover more potent and selective molecules with DMPK properties consistent with once-daily dosing potential. Using the crystal structures of TNG908 and similar compounds as reference, we prioritized three main areas of the molecule to increase potency and selectivity: (A) around the pyridine which sits near MTA, (B) at the 4-position of the piperidine where there is a small, lipophilic pocket, and (C) near the benzothiazole ring where there are several polar residues (Figure 2). This report focuses on a selection of efforts from exploration at each of these regions.

### Area A

**Figure 3:**
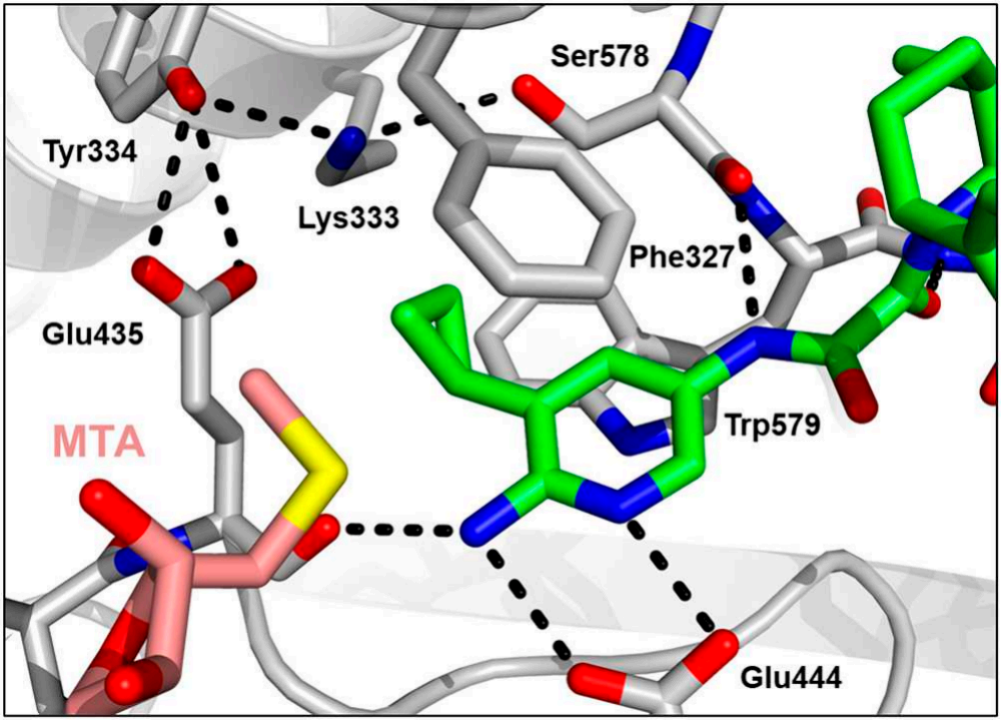
X-ray crystal structure of **3** (green) bound to PRMT5·MTA (PDB entry 9N3N) highlighting steric pressure of cyclopropyl on Glu435 which leads to increased selectivity.

At this stage of the program the potency of many analogs had reached the lower limit of detection in the biochemical assay, so SAR was driven using an isogenic pair of HAP1 MTAP WT and MTAP-null cell lines, where PRMT5-dependent symmetric dimethylarginine (SDMA) modified protein levels were quantified by an in-cell western (ICW) assay, and cell viability was measured using a 7-day CellTiter-Glo assay. Based on our understanding of the mechanism of selectivity,^4,5,16^ we hypothesized that increasing the size of the substituent on the pyridine would put more steric pressure on Glu435, preventing it from rotating into a position permissive to SAM binding, leading to increased selectivity. To understand the effect on potency and selectivity we prepared a series of compounds where we modified the pyridyl methyl of TNG908 with substituents of increasing size, a selection of which are shown in Table 1. With increasing size, both potency and selectivity improved to a point and then sharply decreased once a steric volume exceeded the tolerated limit. For example, compared to TNG908, ethyl (**2**), cyclopropyl (**3**), and oxetane (**4**) all had GI_50_ within 2-fold and significantly improved selectivity (HAP1 MTAP-null GI_50_ = 82, 62, and 53 nM with 43-, 62-, and 50-fold selectivity relative to MTAP WT, respectively). Changing the substituent to a larger group such as THF (**5**), methylcyclopropyl (**7**), and *tert*-butyl (**8**) led to a significant reduction in potency (HAP1 MTAP-null GI_50_ = 410, 1100, 5500, and 1800 nM, respectively) suggesting the size limitation of the pocket was reached. A crystal structure of **3** (Figure 3) shows the cyclopropyl ring effectively fills the pocket, making van der Waals contacts with surrounding residues and blocking Glu435 from adopting the rotamer required to allow SAM to bind, leading to enhanced selectivity. However, these larger, more lipophilic substituents had reduced *in vitro* metabolic stability (Table 1) and increased *in vivo* clearance (data not shown) relative to TNG908. 3-oxetanyl analog **4**, however, with reduced lipophilicity, had good *in vitro* metabolic stability (hCl_int,mic_ = 15 μL min^–1^ mg^–1^) but unfortunately this compound lacked an *in vivo*–*in vitro* correlation (IVIVC), and had an *in vivo* clearance above hepatic blood flow in IV PK studies (1 mpk) in all species tested (data not shown).

**Table 1:**
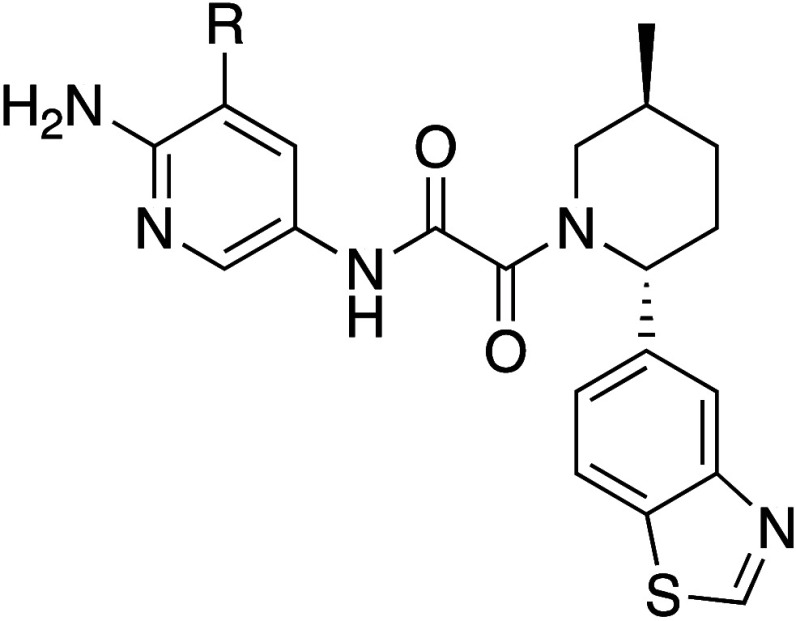

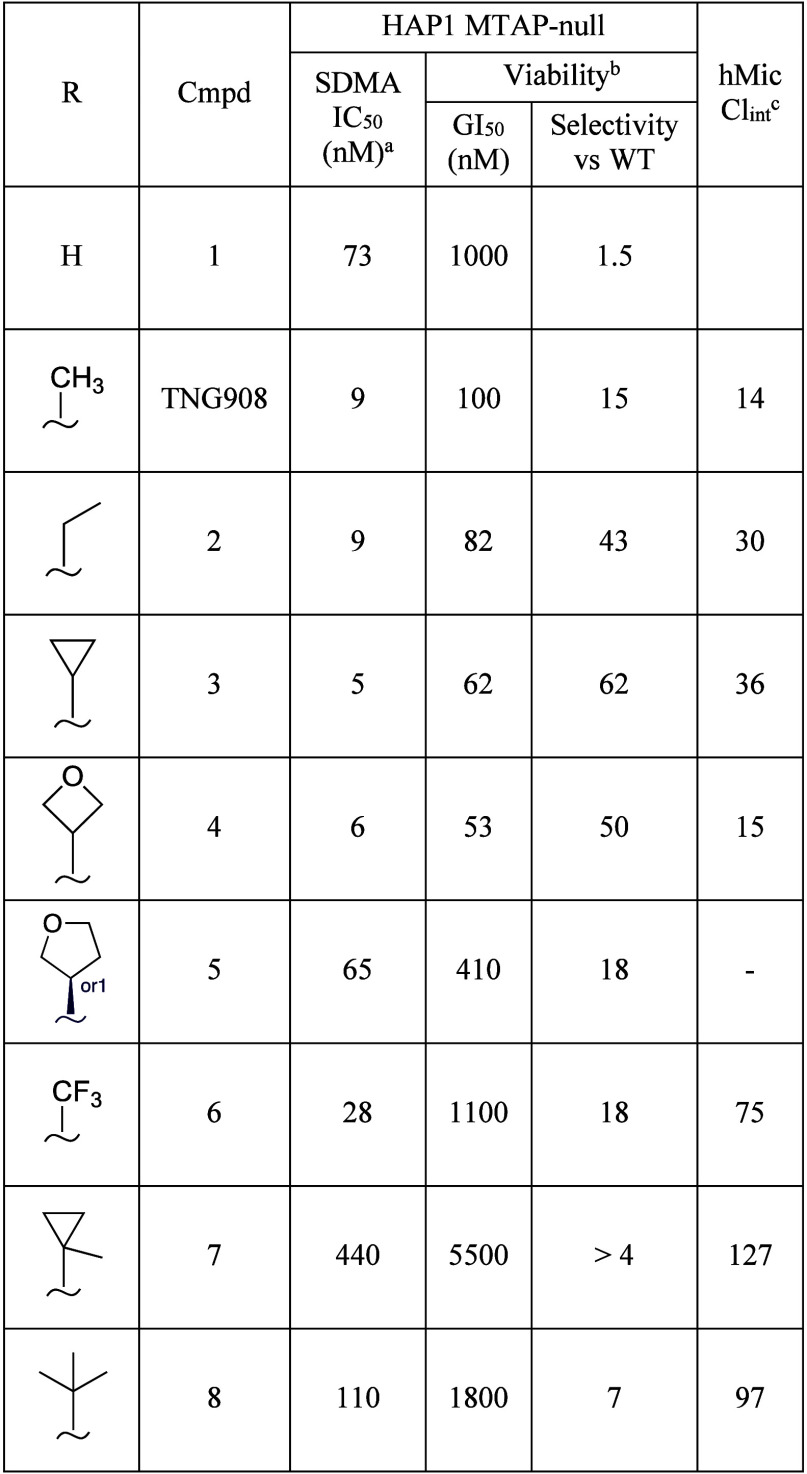
Characterization of Cellular Activity and Metabolic Stability of Compounds **1**–**8** and TNG908

a Inhibition of PRMT5 determined by an SDMA in-cell western assay in the HAP1 *MTAP*-isogenic cell line pair following 24 h compound treatment.

b Viability growth inhibition assessed after 7 days using a CellTiter-Glo luminescence-based assay in HAP1 MTAP-null and HAP1 MTAP WT cells.

c Human liver microsomes, Cl_int_, μL min^–1^ mg^–1^.

With these observations we explored whether bicyclic compounds could achieve better potency and selectivity without the poor metabolic stability that plagued the alkyl substituted series. We previously reported that engaging Glu435 and Lys333 with a carboxamide increased both potency and selectivity,^4^ so we prepared analogs that could engage one or more of the neighboring amino acids Glu435, Lys333, Tyr334, or Ser578 with hydrogen bonds. A representative set of examples from this effort is shown in Table 2.

**Table 2:**
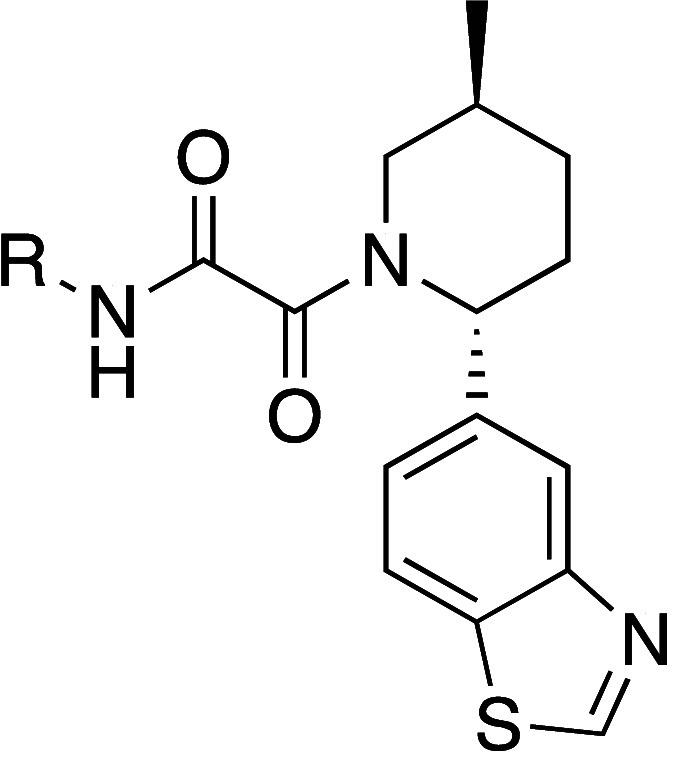

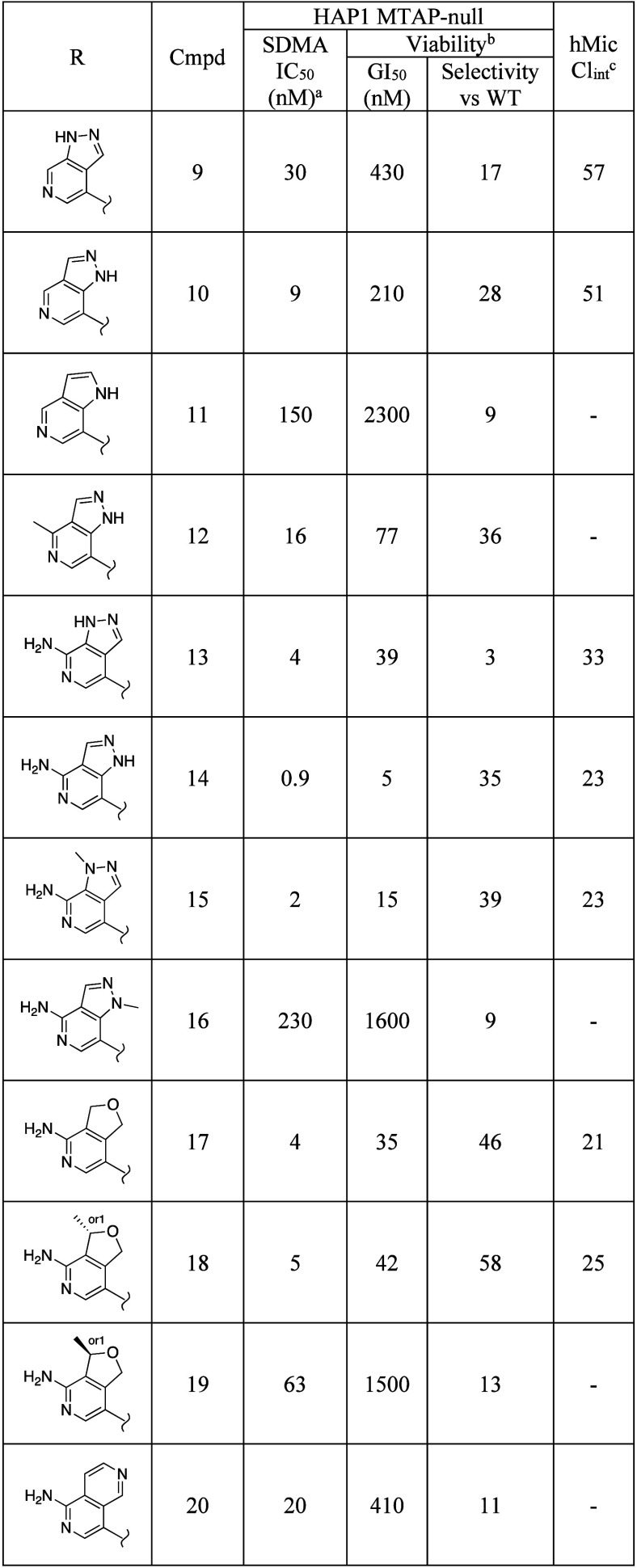
Characterization of Cellular Activity and Metabolic Stability of Compounds **9**–**20**

a Inhibition of PRMT5 determined by an SDMA in-cell western assay in the HAP1 *MTAP*-isogenic cell line pair following 24 h compound treatment.

b Viability growth inhibition assessed after 7 days using a CellTiter-Glo luminescence-based assay in HAP1 MTAP-null and HAP1 MTAP WT cells.

c Human liver microsomes, Cl_int_, μL min^–1^ mg^–1^.

Due to synthetic accessibility, we initially prepared several examples without the amino ortho to the pyridine nitrogen to test tolerance to cyclization, while simultaneously developing viable syntheses of the amino analogs. Compounds **9** and **10** both showed only a 4- and 2- fold decreased potency relative to **TNG908** (HAP1 MTAP-null GI_50_ = 430 nM and 210 nM, respectively) despite the absence of the amino group which had provided 2-log improvement in potency in other series as previously reported.^4^ Removing a nitrogen from pyrazolopyridine **10** to give pyrrolopyridine **11** led to a 10-fold loss in potency. Adding a methyl at the 4-position of **10** led to improved potency and selectivity (**12**, HAP1 MTAP-null GI_50_ = 77 nM, 36-fold selectivity vs MTAP WT cells) and changing the methyl to amine further improved potency by 42-fold compared to the desamino analog, (**14**, HAP1 MTAP-null GI_50_ = 5 nM, 35-fold selectivity vs MTAP WT cells). Interestingly, addition of the NH_2_ to **9** also gave a significant increase in potency (**13**, HAP1 MTAP-null GI_50_ = 39 nM) but selectivity was significantly reduced (3-fold vs HAP1 MTAP WT cells), possibly due to interactions that favor binding with SAM.

**Figure 4:**
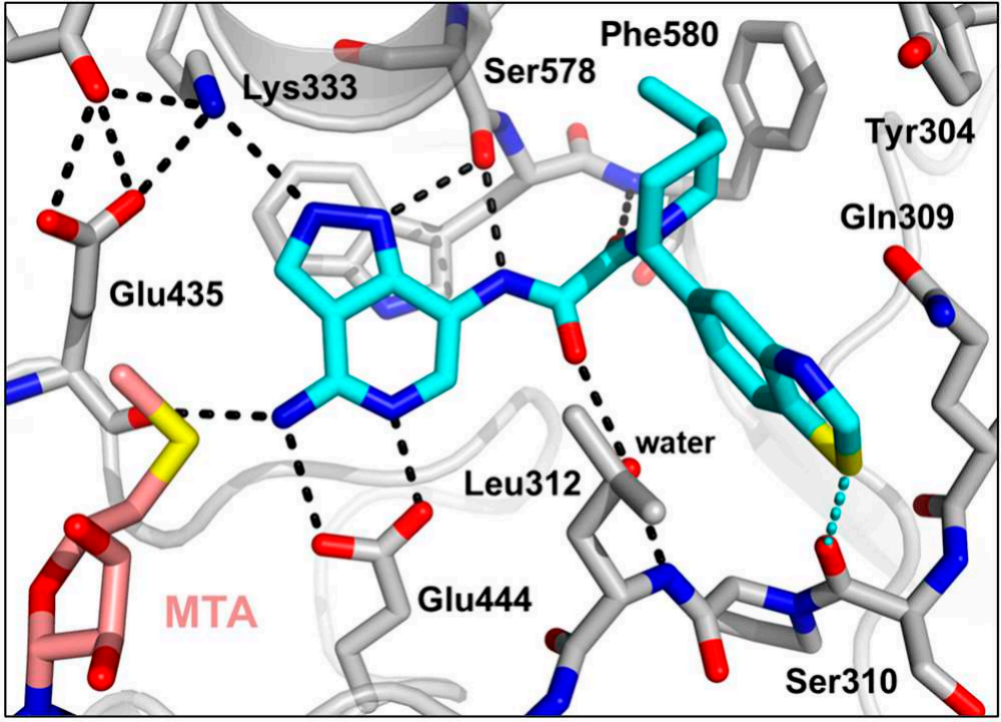
X-ray crystal structure of **14** (blue) bound to PRMT5·MTA (PDB entry 9N3O) which shows an extensive hydrogen bond network achieved with aminopyrazolopyridine oxamides.

A crystal structure of **14** (Figure 4) highlights the interactions that led to its improved potency. The nitrogens form hydrogen bonds with the side chain amine of Lys333 and the backbone carbonyl of Ser578. The NH_2_ on the pyridine forms hydrogen bonds with the backbone carbonyl of Glu435 and the side chain carboxylate of Glu444. These interactions combine with the three oxamide hydrogen bonds to create an extended network of hydrogen bonds across the molecule which can help explain the excellent potency. The structure also rationalizes why **11**, which lacks the nitrogen that can hydrogen bond to Lys333, loses 10-fold in potency compared to **10**.

Further exploration revealed that *N*-methylation of **14** significantly reduced potency (**16**, HAP1 MTAP-null GI_50_ = 1600 nM), presumably due to the loss of the hydrogen bond to Ser578 as well as the presence of the methyl affecting the dihedral angle to the oxamide. In contrast, the selectivity that had been lost in **13** was recovered by projecting more steric bulk toward Glu435 via N-methylation to give **15** (HAP1 MTAP-null GI_50_ = 15 nM with 39-fold selectivity vs HAP1 MTAP WT cells), while potency was maintained.

With the observation that Lys333 hydrogen bonds with the indazole nitrogen atom, we prepared several other analogs to engage it with different ring systems, a few examples of which are also shown in Table 2. Removing aromaticity and utilizing O as the hydrogen bond acceptor we prepared **17**, which has good potency and selectivity (HAP1 MTAP-null GI_50_ = 35 nM, 46-fold selectivity vs HAP1 MTAP WT). To test whether increased steric bulk at the 3-position could further improve selectivity as had been observed in **15**, we prepared both 3-methylated isomers of **17 (18** and **19**, stereochemistry at the chiral methyl arbitrarily assigned). One of these isomers (**18**) maintained potency and had a slight improvement in selectivity vs MTAP WT (58 vs 46-fold), while the other lost significant potency (HAP1 MTAP-null GI_50_ = 1500 nM). Meanwhile, 6–6 rings systems were also explored. This is exemplified by 2,6-naphthyridine (**20**) which showed modest potency and selectivity (HAP1 MTAP-null GI_50_ = 410 nM, 11-fold selectivity vs HAP1 MTAP WT), behavior that was similar in other 6–6 systems tested (not shown).

**Figure 5:**
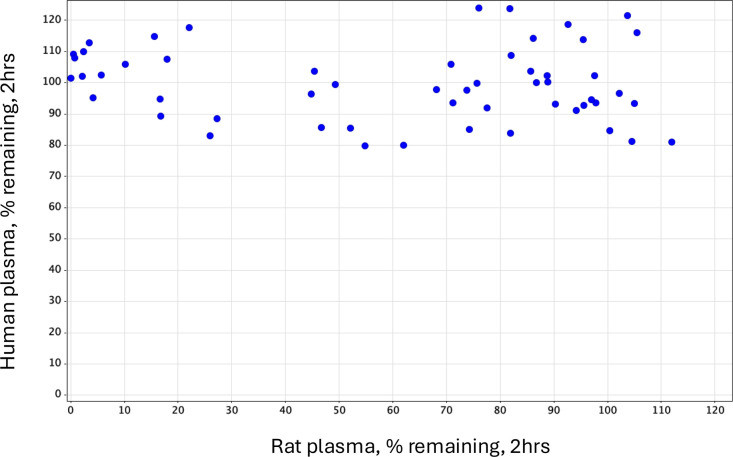
Human vs rat plasma stability at 2 h, 37 °C. Oxamides are stable in human plasma but have variable stability in rat plasma.

From these efforts, **14** met the potency and selectivity goals for the program. Despite having modest *in vitro* human liver microsome stability, we chose to assess *in vivo* PK in dog and cynomolgus monkey to understand the potential for **14** to achieve sufficient exposure in humans. Throughout the oxamide series optimization programs, we did not use rats for *in vivo* assessment due to variable rodent plasma stability which may have contributed to a disconnect between *in vitro* and *in vivo* clearance that was frequently observed. The variable plasma stability was likely due to elevated levels of proteases and esterases in rodent plasma which could lead to hydrolysis of the oxamide moiety. We never observed significant *in vitro* instability in dog, cynomolgus monkey, or human plasma or blood. Figure 5 shows human vs rat plasma stability data. All compounds in the series that were tested showed >80% remaining after 2 h incubation in human plasma, which is considered fully stable within the error of the assay, and no correlation to rat plasma stability was observed. In rodent plasma the compounds could be stabilized *in vitro* with the addition of a protease inhibitor such as phenylmethylsulfonyl fluoride (PMSF) to obtain true protein binding values.

In *in vivo* DMPK studies, **14** showed low total clearance and high bioavailability in dogs (Cl = 3.6 mL min^–1^ kg^–1^ at 1 mpk IV, and F = 82% at 3 mpk PO), and high total clearance and moderate bioavailability in cynomolgous monkey (Cl = 25 mL min^–1^ kg^–1^ at 1 mpk IV, and F = 43% at 3 mpk PO) (Table 3). These and other *in vivo* data along with *in vitro* stability data across species led to a predicted human dose that required a BID schedule to achieve GI_90_ target coverage at trough, so we continued efforts to identify molecules with a further improved profile.

**Table 3:** *In Vitro* and *In Vivo* ADME/PK data for **14**

	dog	cynomolgus monkey	human
microsomes, Cl_int_^a^	13	73	23
hepatocytes, Cl_int_^b^	10	38	2
protein binding (% unbound)	<1	14	20
clearance (mL min^–1^ kg^–1^)^c^^,^^d^	3.6	25	–
Vd_ss_ (L/kg)^c^^,^^d^	0.9	2.2	–
F (%)^c^^,^^d^	82	43	–

a Liver microsomes, Cl_int_, μL min^–1^ mg^–1^.

b Hepatocytes, Cl_int_, μL min^–1^/10^6^ cells

c IV/PO dosing in beagle dog (vehicle, IV: 0.2 mg/mL solution of 1% v/v DMSO/99% 20% w/v HP-β-CD in saline; PO: 0.3 mg/mL suspenstion of 0.5% methylcellulose w/v in water, *n* = 3 per arm).

d IV/PO dosing in cynomolgus monkey (vehicle, IV: 0.2 mg/mL solution of 1% v/v DMSO/99% 20% w/v HP-β-CD in saline; PO: 0.3 mg/mL suspension 0.5% methylcellulose w/v in water, *n* = 3 per arm).

### Area B

The crystal structure of TNG908 with PRMT5·MTA revealed a small pocket near the 4-position of the piperidine (Figure 1). This pocket was partially filled by the initial HTS hit in this series, but as the series was optimized, 180° rotation of the piperidine oriented the 2-substitutions away from this pocket and into the orientation shown in Figures 1 and 4.^4^ To try and access this pocket from the current series we explored substitutions at the 4-position of the piperidine, as well as conversion to N-substituted piperazines. A representative selection of these compounds, as well as the morpholine analog, is shown in Table 4. We also explored acyclic attachments to the oxamide which will be shared in a subsequent publication.

**Table 4:**
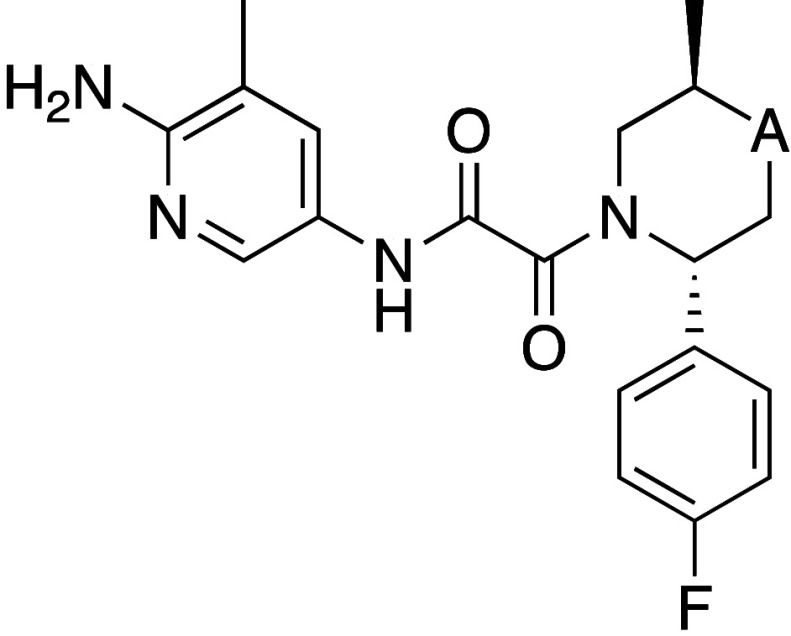

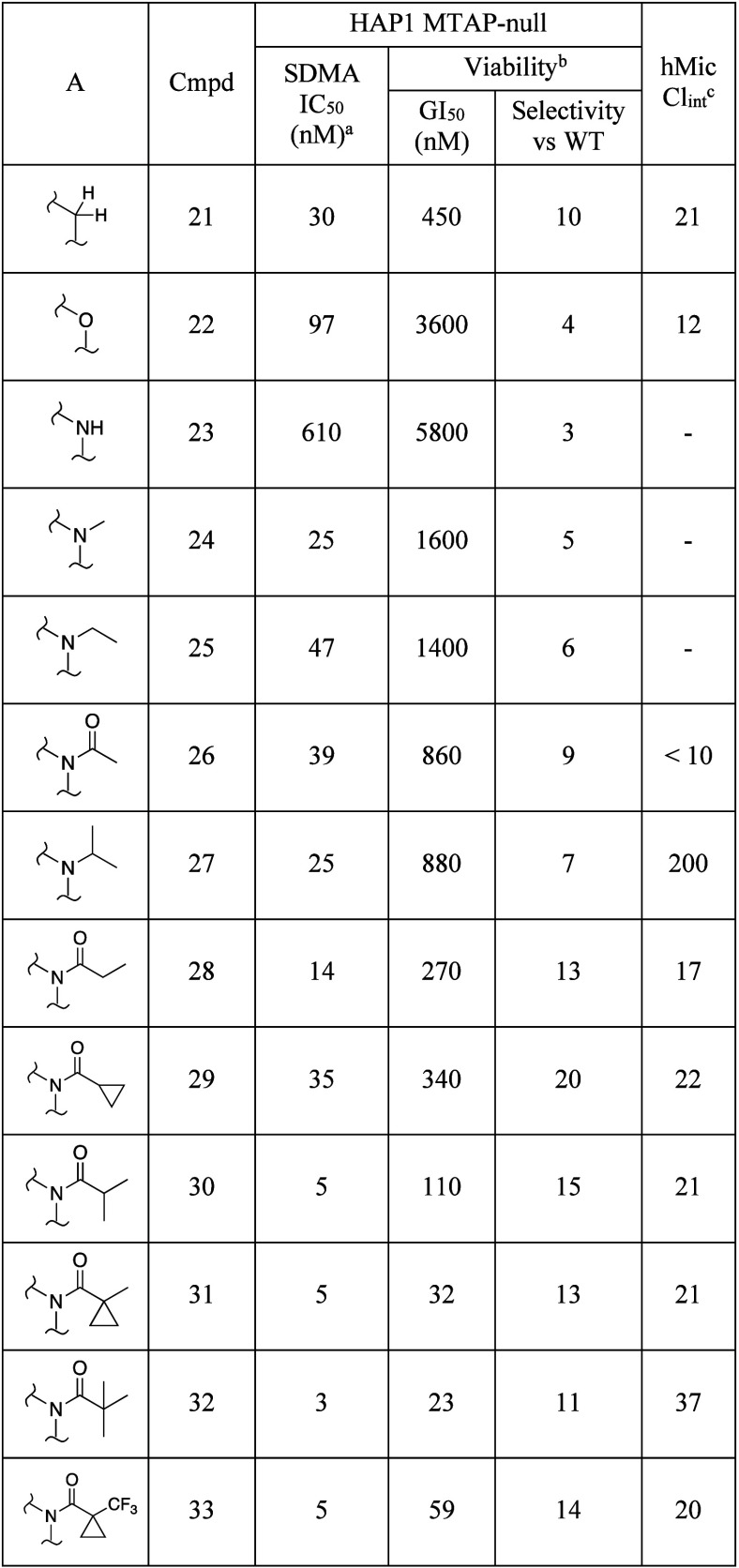
Characterization of Cellular Activity and Metabolic Stability of Compounds **21**–**33**

a Inhibition of PRMT5 determined by an SDMA in-cell western assay in the HAP1 *MTAP*-isogenic cell line pair following 24 h compound treatment.

b Viability growth inhibition assessed after 7 days using a CellTiter-Glo luminescence-based assay in HAP1 MTAP-null and HAP1 MTAP WT cells.

c Human liver microsomes, Cl_int_, μL min^–1^ mg^–1^.

For much of this effort we utilized a 4-F-phenyl substitution that was equipotent to the unsubstituted phenyl series as the baseline for exploration. Benzothiazole analogs, which had given potency increases relative to the phenyl and 4-F-phenyl in the piperidine series of TNG908, but which required more synthetic effort, were prepared in the most interesting cases to crosscheck the SAR.

**Figure 6:**
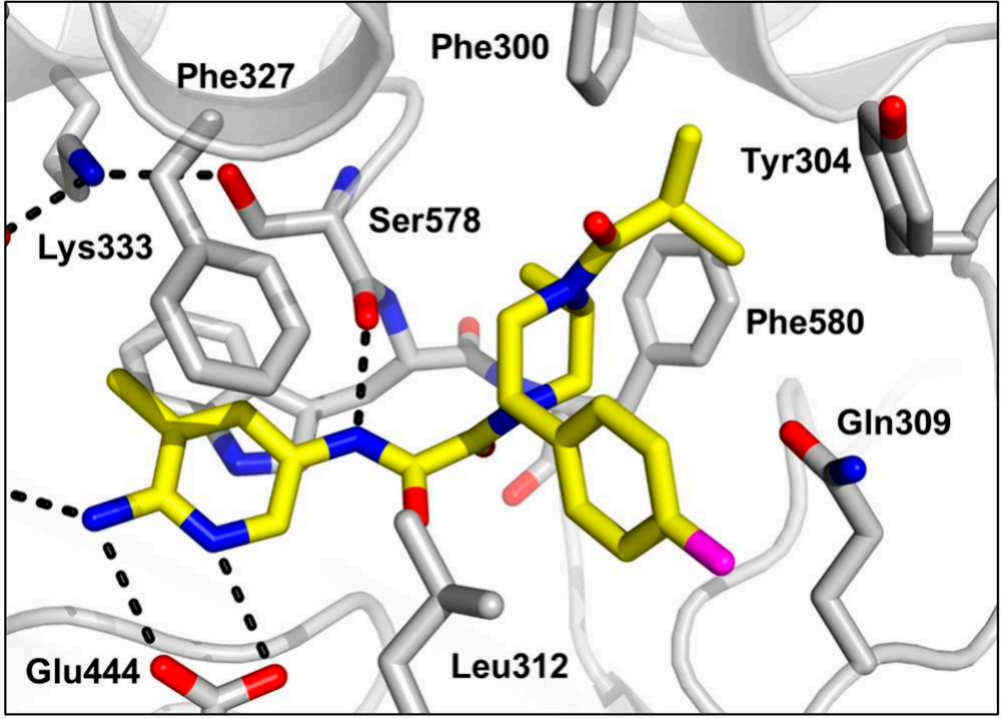
X-ray crystal structure of **30** (yellow) bound to PRMT5·MTA (PDB entry 9N3P) highlighting the occupancy of the pocket with piperazine amides.

Baseline piperidine compound (**21**) had modest potency and selectivity (HAP1 MTAP-null GI_50_ = 450 nM, 10-fold selectivity vs HAP1 MTAP WT). Changing the ring to morpholine or unsubstituted piperazine (**22**, **23**) led to significant loss of potency (HAP1 MTAP-null GI_50_ = 3600 and 5800 nM, respectively). Alkylation of the piperazine nitrogen led to gradual improvements in potency with increasing size and lipophilicity (**24**, **25**, **27**) but no improvement in selectivity. Acylation of the piperazine nitrogen also improved potency (**26**, HAP1 MTAP-null GI_50_ = 860 nM) while larger, more lipophilic amides further improved potency with a modest improvement in selectivity, as exemplified by isopropyl, methylcyclopropyl, *tert*-butyl, and trifluoromethylcyclopropyl carboxamides (**30**–**33)**, which were 4–20-fold more potent than the baseline piperidine, with selectivity of 11–15-fold. A crystal structure of **30** in PRMT5·MTA (Figure 6) shows the amide substituent fills the small pocket with minor adjustments in the Phe300 and Tyr304 side chains to accommodate the added bulk. However, despite achieving the goal of utilizing this pocket for potency improvements, none of these ring modifications and/or substitutions approached our targets for potency and selectivity, and they also had poor *in vitro* metabolic stability.

Next, we explored combinations of these modified rings with the best substituents from the exploration in Area A, a sample of which are shown in Table 5. Combining these modifications gave good potency and, in some cases, selectivity, but generally with modest to poor metabolic stability, and never with a better profile than **14** overall. For example, combining the piperazine series with the pyrazolopyridine of **14** (**34**–**38**) showed good potency that improved with increasing size and lipophilicity of the piperazine substituent, but as potency improved, selectivity generally decreased and metabolic stability was modest to poor. Also, while addition of the benzothiazole had increased potency 5 to 10-fold in the piperidine series,^4^ addition of the benzothiazole in the aminopyrazolopyridine-piperazine series decreased potency 4 to 8-fold (**39**–**41** vs **35**, **36**, **38**).

**Table 5:**
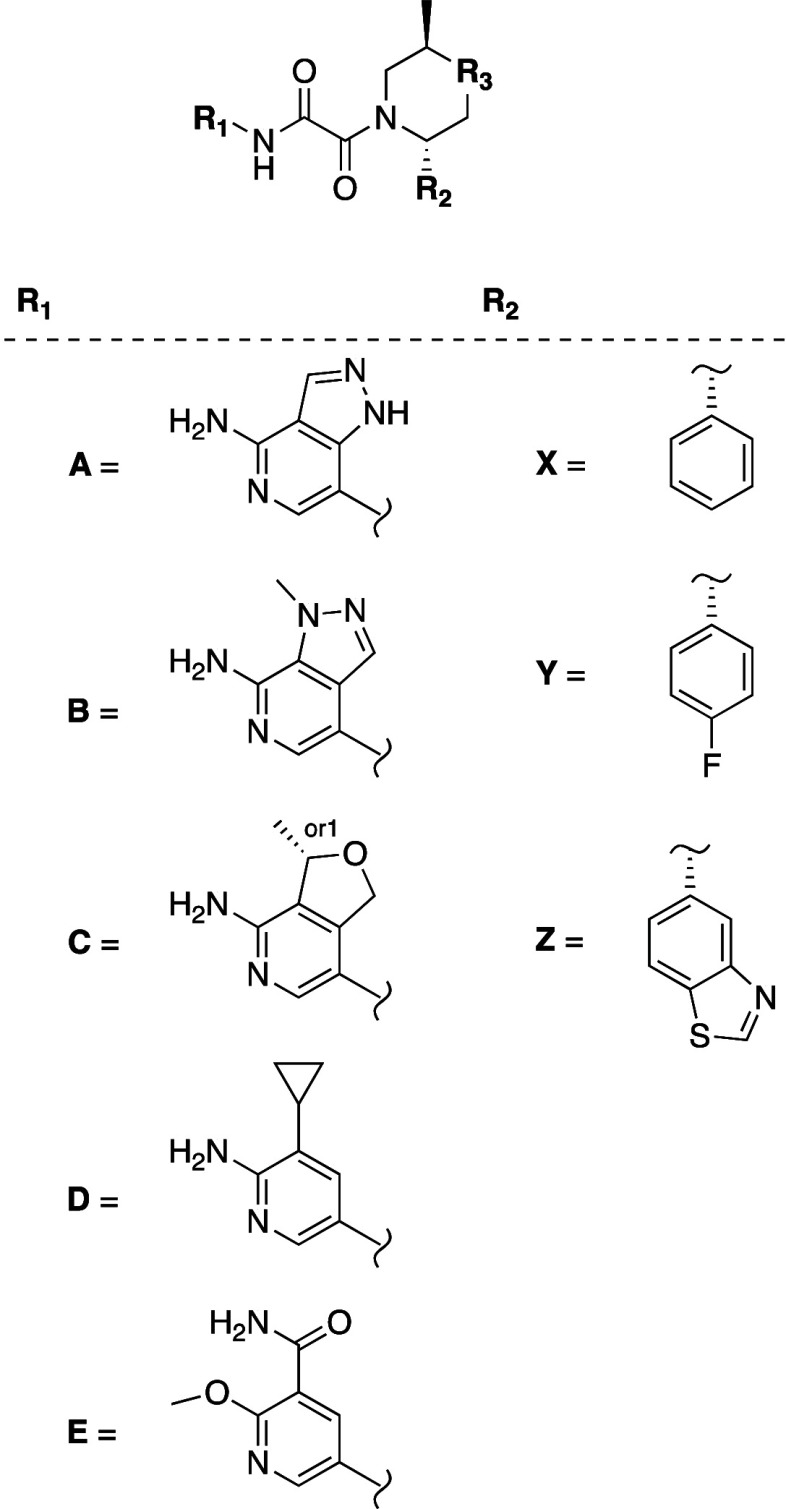

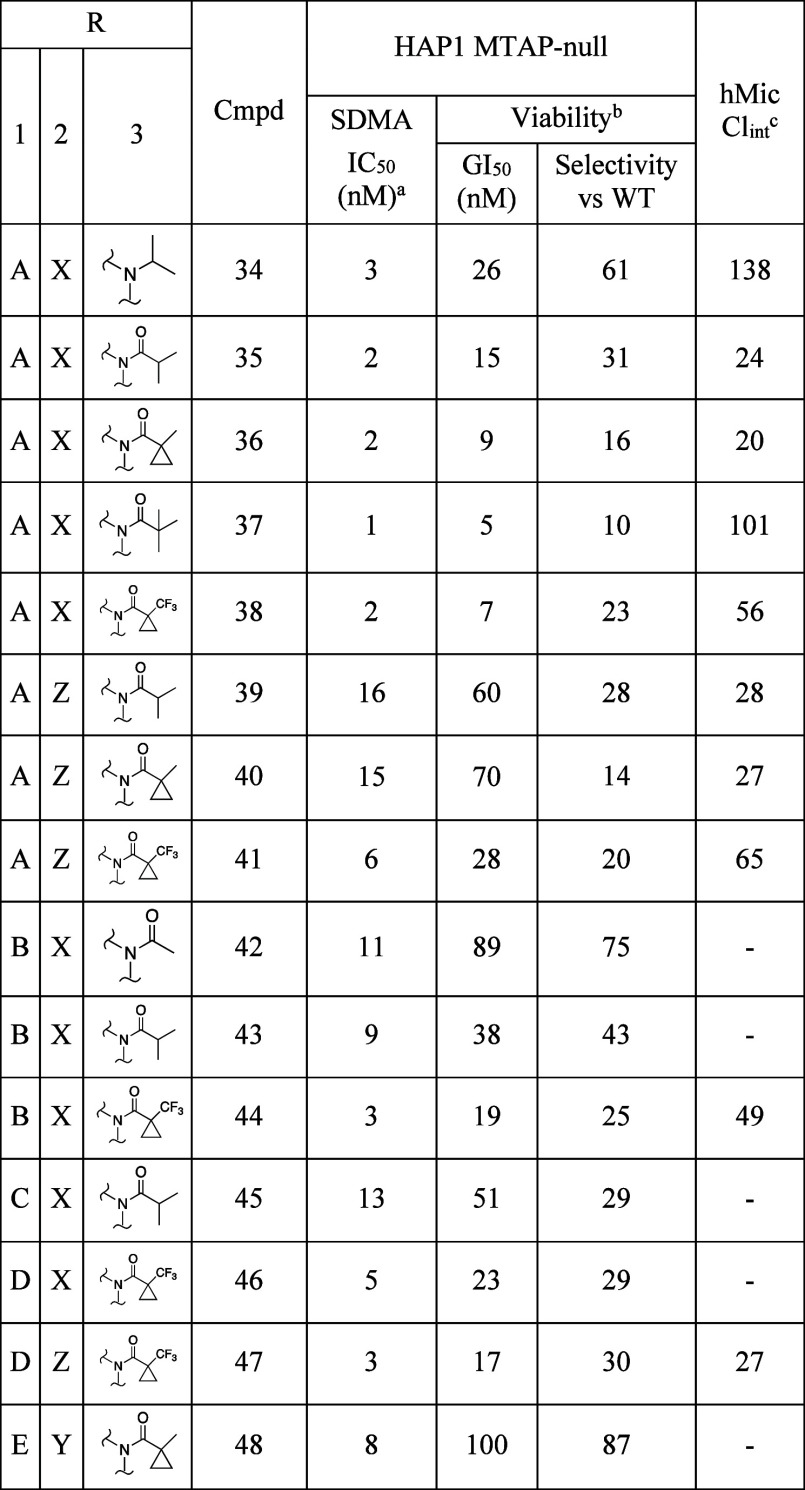
Characterization of Cellular Activity and Metabolic Stability of Compounds **34**–**48**

a Inhibition of PRMT5 determined by an SDMA in-cell western assay in the HAP1 *MTAP*-isogenic cell line pair following 24 h compound treatment.

b Viability growth inhibition assessed after 7 days using a CellTiter-Glo luminescence-based assay in HAP1 MTAP-null and HAP1 MTAP WT cells.

c Human liver microsomes, Cl_int_, μL min^–1^ mg^–1^.

Combination of piperazines with the methylated, regioisomeric pyrazolopyridine (**42**–**44**) were generally less potent than the baseline piperidine **15** (HAP1 MTAP-null GI_50_ = 89, 38, and 19 nM, respectively, compared to 15 nM) but had good selectivity vs MTAP WT (75-, 43-, and 25-fold). Other combinations of the most active and selective pyridines with substituted piperazines, while increasing potency compared to the baseline matched molecular pairs,^17^ did not achieve sufficient improvements in potency (**45**, **46**, **48**) and again, no further improvement was gained by adding the benzothiazole to one of the more potent analogs (**46**) to give **47** (HAP1 MTAP-null GI_50_ = 23 nM vs 17 nM, respectively).

In all, modifying and/or substituting the ring in this position of the molecule failed to produce the potency and metabolic stability that would be needed to advance a molecule from this series.

### Area C

Shifting focus to the area of the binding pocket around the benzothiazole, we hypothesized that electrostatic interactions with polar side chains could lead to increased potency. Glu320 sits toward the edge of the pocket and is solvent exposed in a position that suggested it could be reached with an appropriate substitution off the 2-position of the benzothiazole ring. To test our hypothesis, we extended basic amines into the region via both alkyl chains and rings. If successful, in addition to improving potency, this approach offered the potential to increase *in vivo* half-life, an important goal of the program, by modulating the volume of distribution (Vd_ss_).^18^ Select examples of that exploration are shown in Table 6.

**Table 6:**
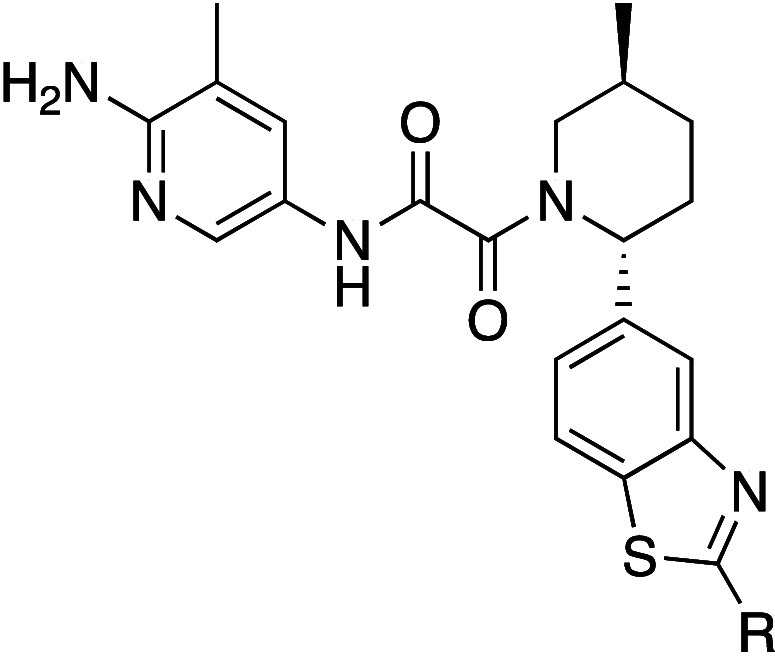

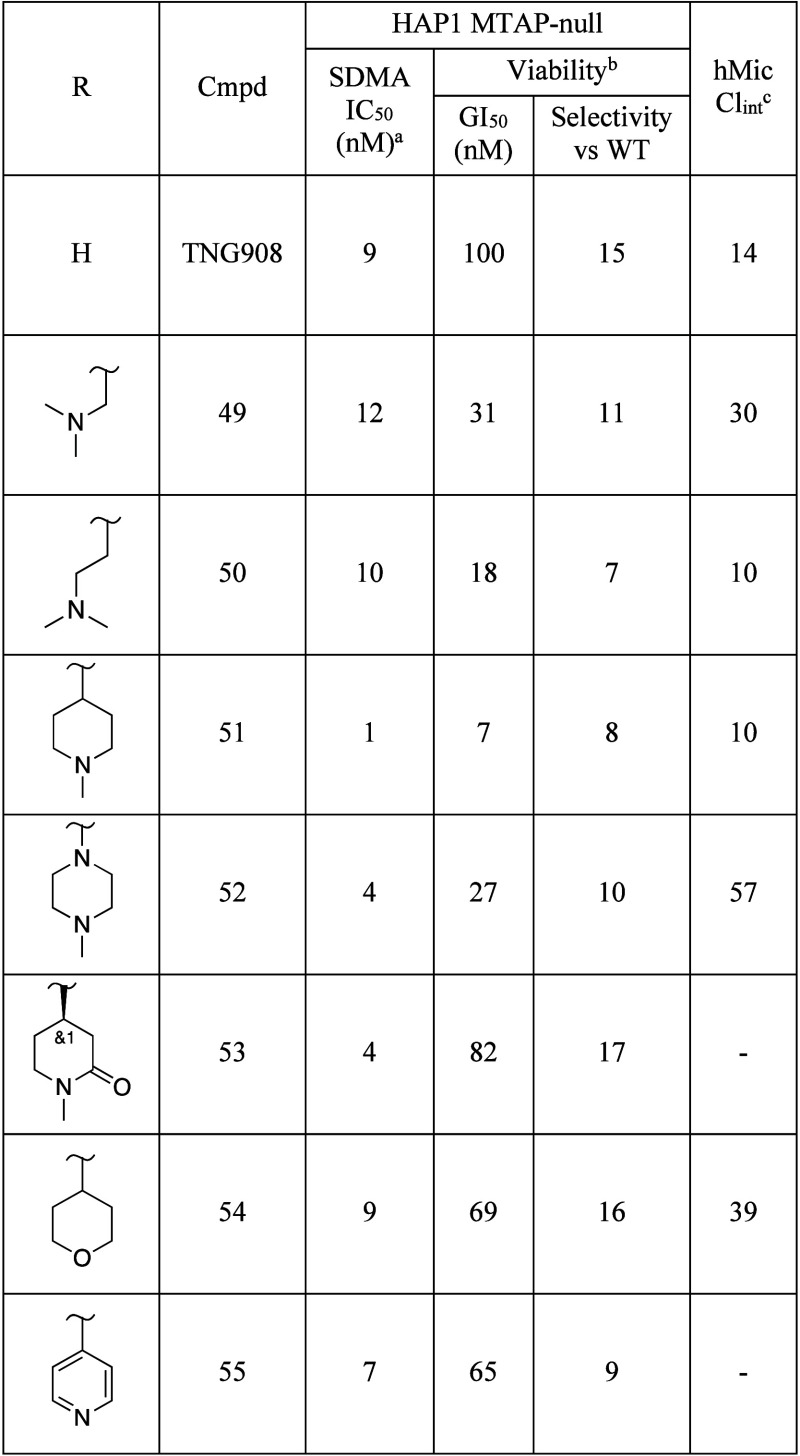
Characterization of Cellular Activity and Metabolic Stability of Compounds **49**–**55** and TNG908

a Inhibition of PRMT5 determined by an SDMA in-cell western assay in the HAP1 *MTAP*-isogenic cell line pair following 24 h compound treatment.

b Viability growth inhibition assessed after 7 days using a CellTiter-Glo luminescence-based assay in HAP1 MTAP-null and HAP1 MTAP WT cells.

c Human liver microsomes, Cl_int_, μL min^–1^ mg^–1^.

A single methylene linker to the amine in **49** gained about 3-fold in viability potency but had poor human microsomal stability (HAP1 MTAP-null GI_50_ = 31 nM, human Cl_int,mic_ = 30 μL min^–1^ mg^–1^). Extending one methylene further (**50**) led to a 5-fold improvement in potency with better microsomal stability (HAP1 MTAP-null GI_50_ = 18 nM, human Cl_int,mic_ = 10 μL min^–1^ mg^–1^). Utilizing an *N*-methylpiperidine ring (**51**) gave the best potency in the series, again with good stability (HAP1 MTAP-null GI_50_ = 7 nM, human Cl_int,mic_ = 10 μL min^–1^ mg^–1^). *N*-methylpiperazine **52** had potency similar to **49**, but also had poor human microsomal stability (HAP1 MTAP-null GI_50_ = 27 nM, human Cl_int,mic_ = 57 μL min^–1^ mg^–1^). Nonbasic compounds **53** and **54** and less basic pyridine **55** were less potent than the *N*-methylpiperidine. All these compounds had similar or worse selectivity against MTAP WT compared to TNG908.

**Figure 7:**
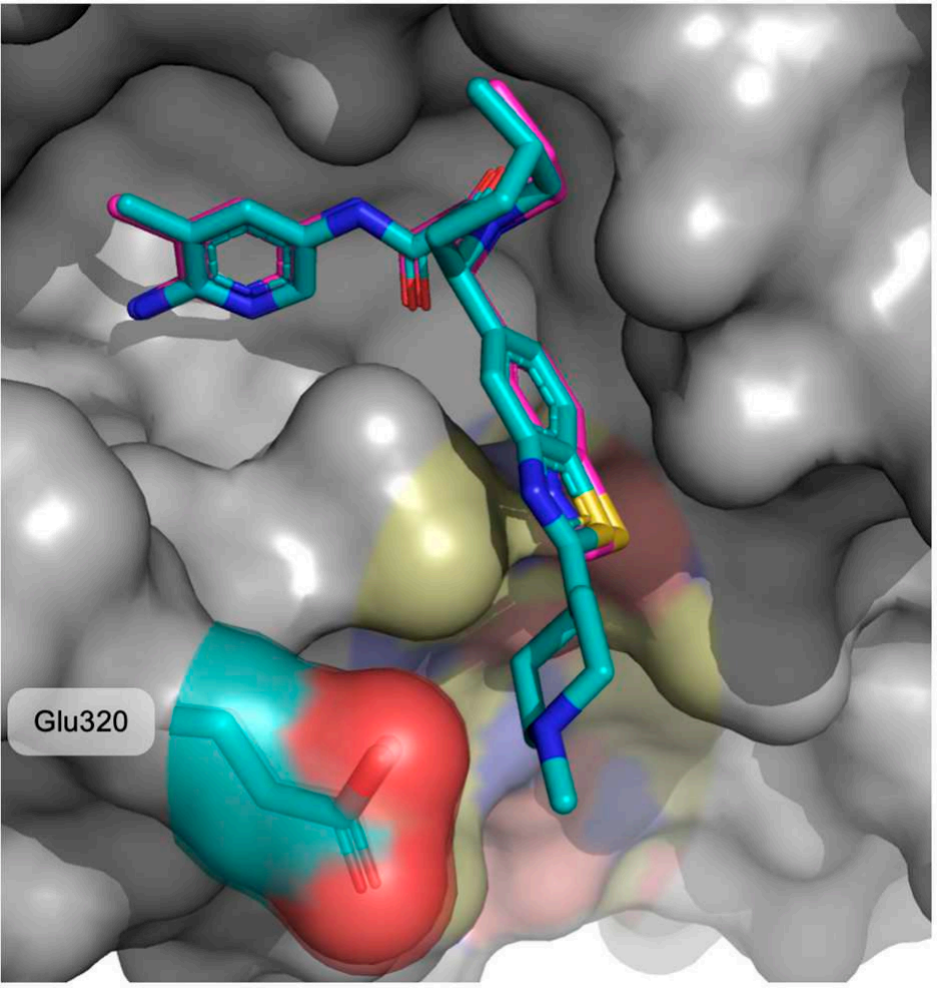
X-ray crystal structure overlay of **51** (teal) and TNG908 (magenta) bound to PRMT5·MTA (PDB entries 9N3Q and 8VEY) highlighting the salt bridge formed between the piperidine nitrogen of **51** and Glu320.

A crystal structure of **51** (Figure 7) shows that the *N*-methylpiperidine engages Glu320 in a salt bridge with a distance between the acid of Glu320 and the N of the piperidine of 3.9 Å, consistent with the distances observed between ion-paired amino acid side chains in proteins.^19,20^ With the significant increase in potency achieved via this interaction, we combined these pendant amines with the best pyridine analogs we had discovered in Area A. A representative set of *N*-methylpiperidine analogs is shown in Table 7.

**Table 7:**
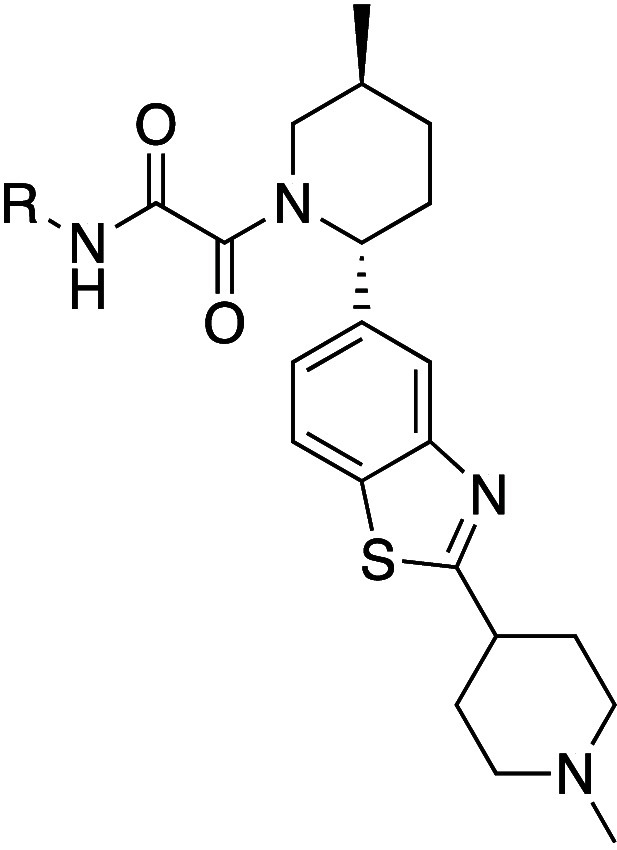

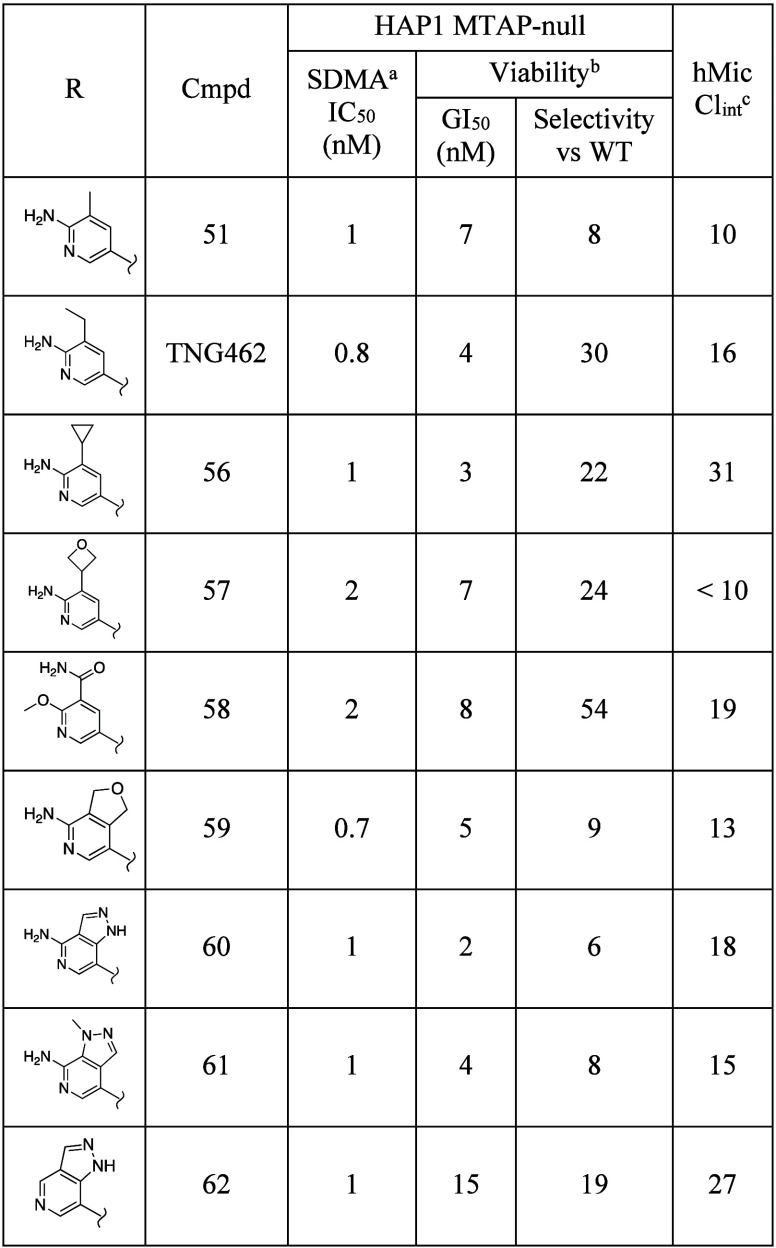
Characterization of Cellular Activity and Metabolic Stability of Compounds **56**–**62** and **TNG462**

a Inhibition of PRMT5 determined by an SDMA in-cell western assay in the HAP1 *MTAP*-isogenic cell line pair following 24 h compound treatment.

b Viability growth inhibition assessed after 7 days using a CellTiter-Glo luminescence-based assay in HAP1 MTAP-null and HAP1 MTAP WT cells.

c Human liver microsomes, Cl_int_, μL min^–1^ mg^–1^.

With **51** as baseline, we observed that 5-ethyl-6-aminopyridine (**TNG462**) slightly improved potency while increasing selectivity to 30-fold vs HAP1 MTAP WT cells, with good human microsomal stability (HAP1 MTAP-null GI_50_ = 4 nM, human Cl_int,mic_ = 16 μL min^–1^ mg^–1^). 5-cyclopropyl analog **56** also had excellent potency, slightly less selectivity, but poor human microsomal stability (HAP1 MTAP-null GI_50_ = 3 nM, 22-fold selectivity vs MTAP WT, human Cl_int,mic_ = 31 μL min^–1^ mg^–1^). Oxetane **57** had good potency with excellent stability (HAP1 MTAP-null GI_50_ = 7 nM, human Cl_int,mic_ = < 10 μL min^–1^ mg^–1^), but similar to the unsubstituted benzothiazole/oxetane analog **4**, it did not demonstrate IVIVC and had poor *in vivo* clearance in all species tested (data not shown). 2-Methoxynicotinamide **58**, a group that we had previously reported as very selective,^4^ also had good potency and stability with excellent selectivity (HAP1 MTAP-null GI_50_ = 8 nM, 54-fold selectivity vs MTAP WT, human Cl_int,mic_ = 19 μL min^–1^ mg^–1^). However, analysis of both *in vitro* and *in vivo* data in dog and cynomolgus monkey suggested that similar to **14**, it likely would require a significant BID dose to achieve coverage of GI_90_ at trough due to a short predicted human half-life, so it was not pursued further.

**Figure 8:**
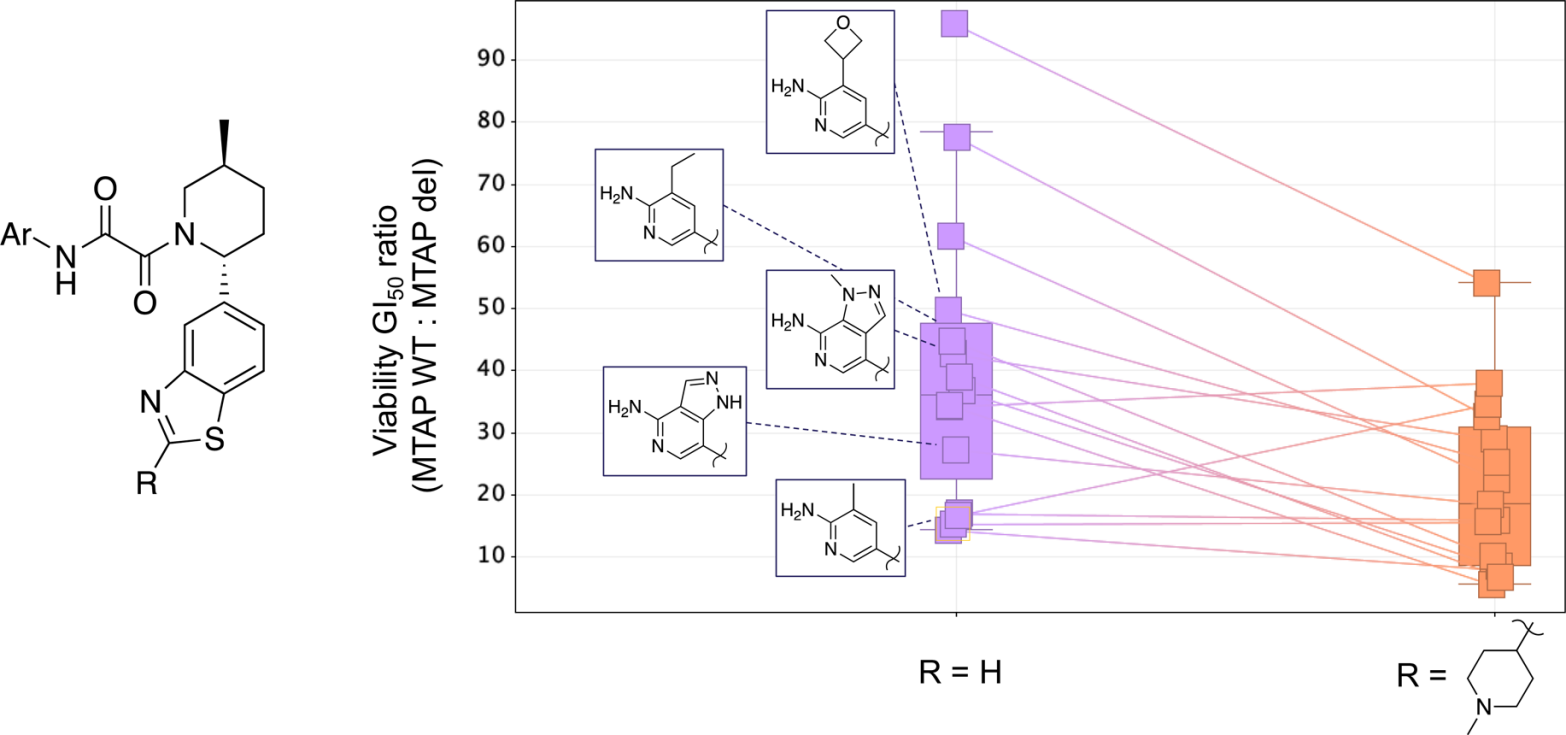
Generally decreasing viability selectivity versus MTAP WT with *N*-methylpiperidine substitution at the benzothiazole 2-position vs unsubstituted benzothiazole (2-H) matched molecular pairs. Matched molecular pairs^17^ are represented by squares that are connected by a line, and four examples are highlighted.

Bicyclic compounds (**59**–**61**) all achieved good potency (HAP1 MTAP-null GI_50_ = 5, 2, and 4 nM, respectively) but they had poor selectivity (9, 6, and 8-fold selectivity vs MTAP WT, respectively), unlike the matched molecular pairs in the unsubstituted benzothiazole series (**17**, **14**, **15**) where they had excellent selectivity (46-, 35-, and 39-fold selectivity vs MTAP WT, respectively). This trend was observed throughout the series, i.e., when Glu320 is engaged in a salt bridge, bicyclic compounds generally had poor selectivity (Figure 8). This reduced selectivity is frequently the result of a greater increase in potency in MTAP WT cells than in MTAP-null cells when piperidine is incorporated on the benzothiazole in the bicyclic series. The comparative increase in potency is smaller when piperidine is added to benzothiazole in the monocyclic pyridine series. For example, addition of piperidine to the benzothiazole in TNG908 to give **51** increased potency by 14-fold in MTAP-null cells and by 30-fold in MTAP WT cells (MTAP-null GI_50_ = 100 nM and 7 nM and MTAP WT GI_50_ = 1600 nM and 53 nM, respectively for TNG908 and **51**), decreasing selectivity by only 2-fold. However, addition of the piperidine to the benzothiazole in the bicyclic pyrazolopyridine-containing **14** to give **60** increased potency by only 2-fold in MTAP-null cells but by 13-fold in MTAP WT cells (MTAP-null GI_50_ = 100 nM and 7 nM and MTAP WT GI_50_ = 1600 nM and 53 nM, respectively for **14** and **60**), leading to a 6-fold reduction in selectivity from 35-fold to 6-fold. This differential may be explained by subtle movement of the molecules when the salt bridge with Glu320 is engaged that are tolerated to different degrees depending on the aryl group and cofactor.

With the addition of the *N*-methylpiperidine, these compounds generally showed excellent potency and improved metabolic stability relative to the unsubstituted benzothiazole series, and with **TNG462** we had identified a compound that met the potency and selectivity requirements of the program and had good *in vitro* metabolic stability. Given these attributes we further profiled **TNG462***in vitro* and *in vivo*.

## Biochemical and Biophysical Characterization

To measure the biochemical potency of **TNG462**, we explored multiple approaches. **TNG462** had potency which was below the detection limit of both the fluorescence polarization peptide displacement and the radiometric FlashPlate assays in the presence or absence of MTA. SPR binding assays were also attempted, however, due to the slow dissociation rate of **TNG462** and the consequently long dissociation measurement time, the stability of the PRMT5 enzyme complex on the SPR chip became a concern and we could not obtain satisfactory results. We turned to an enzyme activity recovery assay, which biochemically measures the rate of compound dissociation from the enzyme–inhibitor complex, to determine the potency or to estimate the potency boundary of **TNG462**. The H4 peptide concentration was increased from 1 μM, which was used in the *K*_i_ determination for TNG908,^4^ to 10 μM to enhance peptide competition. We first measured the **TNG462** dissociation from PRMT5:**TNG462** binary complex in the absence of MTA. We did not observe any nonlinear increase in the enzyme activity progress curve over 5 h (see the Supporting Information), which suggests that the residence time half-life of **TNG462** is >5 h and longer assay time would be needed to assess the recovery of enzyme activity, again leading to concern about the stability of the enzyme. Due to this limitation, we used the initial apparent recovery rate in the assay to calculate the potency of **TNG462** (see the Supporting Information). Without MTA, the calculated average *K*_i_ = 2.9 ± 0.6 pM. With the addition of MTA we observed a reduced initial apparent enzymatic rate, indicating increased potency of **TNG462** in the presence of MTA. However, accurate measurement of *K*_i_ is challenging as the apparent initial rate is close to the background control in which no enzyme is present, therefore the *K*_i_ of **TNG462** can only be estimated to be ≤300 femtomolar.

## Further *In Vitro* Profiling of **TNG462**

**Figure 9:**
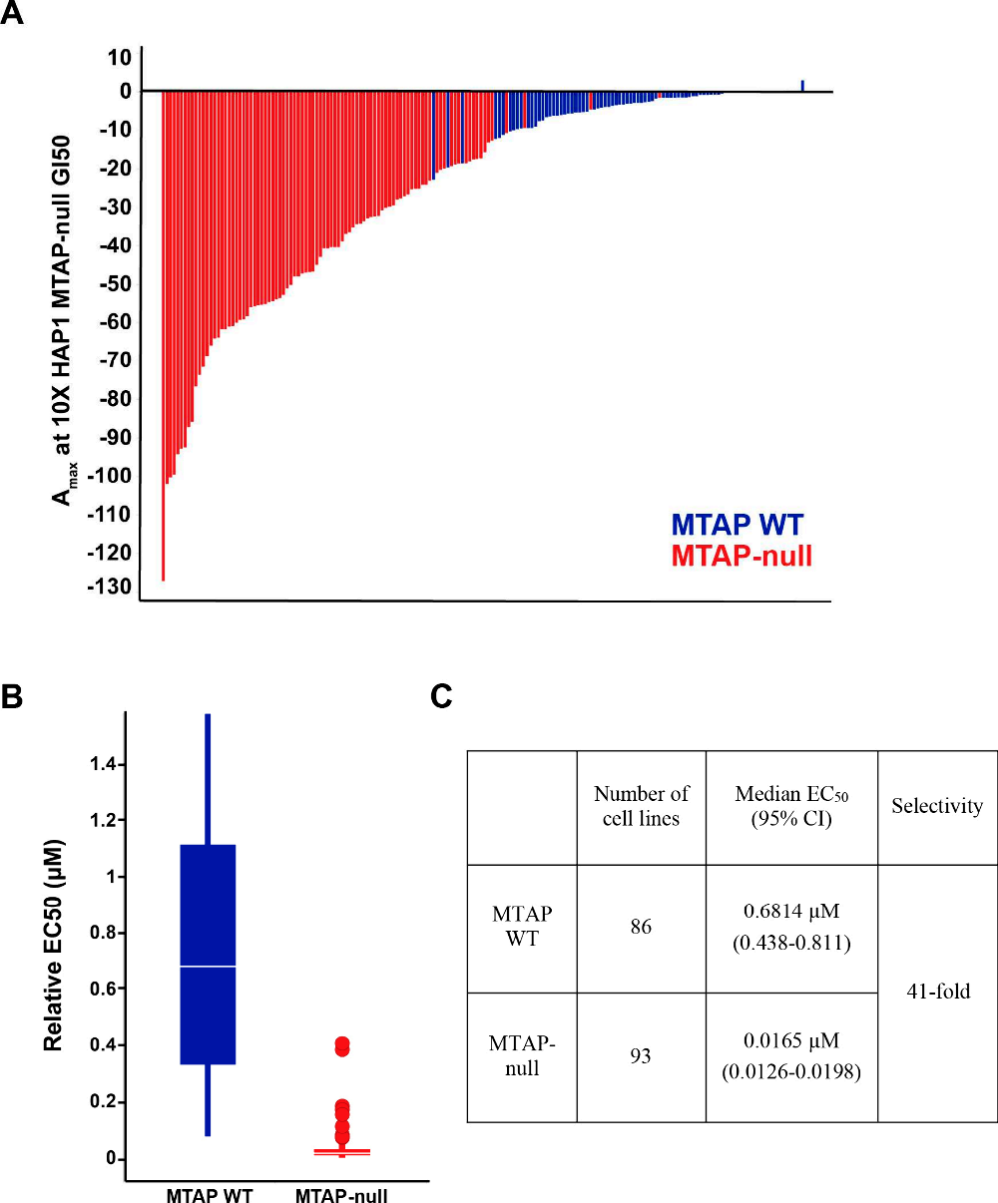
**TNG462** in a 179-cancer cell line panel, using a 7-day viability assay, demonstrating excellent selectivity for MTAP-null cell lines. Maximum effect relative to the HAP1 MTAP-null cell line (A), median EC50s for the 86 MTAP WT cell lines and 93 MTAP-null cell lines (B and C).

In the HAP1 MTAP-null SDMA in-cell western (PD) assay **TNG462** has an IC_50_ = 800 pM (Table 7) and an IC_90_ = 8 nM. Selectivity to WT at IC_90_ was 33-fold (HAP1 MTAP WT SDMA in-cell western IC_90_ = 280 nM). **TNG462** was also profiled for off-target activity against a panel of 39 methyltransferases at 1 and 10 μM and showed no significant activity other than with PRMT5·MEP50 (see the Supporting Information). **TNG462** was also tested in an *in vitro* toxicology safety panel (SAFETYscan E/IC_50_) of 78 known off-target binding and functional assays up to 10 μM and had no activity of concern (see the Supporting Information). **TNG462** had an IC_50_ = 8.6 μM in a hERG syncropatch assay, which is nearly 800-fold over the targeted MTAP-null GI_90_ (11 nM). **TNG462** was also evaluated in a 7-day viability assay with a selection of 4 *MTAP*-isogenic cell lines and had an average MTAP-null GI_50_ = 5 nM, with average selectivity vs MTAP WT = 44-fold (Table 8). Additionally, **TNG462** was assessed in the same 7-day viability assay using a 179-cancer cell line panel representing multiple lineages and showed an median 41-fold selectivity for MTAP-null cell lines relative to MTAP WT cell lines (Figure 9).

**Table 8:** Cellular Viability Data in Four *MTAP*-Isogenic Cell Lines^a^

	GI_50_ ± SD (nM)		
cell line (histology)	MTAP-null	MTAP WT	selectivity
LU99 (NSCLC)	8.3 ± 5.6	299 ± 190	36-fold
LN18 (GBM)	5.5 ± 3.6	319 ± 117	58-fold
HCT116 (CRC)	1.8 ± 0.5	94 ± 31	52-fold
HAP1 (CML)	3.9 ± 1.2	113 ± 43	29-fold
Average Selectivity = 44-fold			

a Viability growth inhibition assessed after 7 days using a CellTiter-Glo luminescence-based assay in the indicated *MTAP*-isogenic cell line pairs.

## Pharmacokinetic Profiling of **TNG462** in Preclinical Species

The PK properties of **TNG462** were evaluated in beagle dogs and cynomolgus monkeys (Table 9). Following a 1 mg/kg IV dose of **TNG462** to beagle dogs (*n* = 3), clearance was 23 mL min^–1^ kg^–1^, volume of distribution was 21 L/kg, and half-life was 14 h. Oral administration of a 3 mg/kg dose of **TNG462** to beagle dogs resulted in a *C*_max_ of 0.064 μg/mL, AUC_inf_ of 1.19 h μg/mL, with 52% bioavailability. Following a 1 mg/kg IV dose of **TNG462** to cynomolgus monkeys (*n* = 3), clearance was 20 mL min^–1^ kg^–1^, volume of distribution was 26 L/kg, and half-life was 20 h. Oral administration of a 3 mg/kg dose of **TNG462** to cynomolgus monkeys (*n* = 3) resulted in a *C*_max_ of 0.058 μg/mL, AUC_inf_ of 0.727 h μg/mL, with 32% bioavailability. As hypothesized, the higher Vd_ss_ driven by the basic amine of the piperidine led to an increased half-life, and these data led to a predicted human half-life >24 h which supports once daily dosing. The predicted exposure with this dose regiment has a *C*_max_/*C*_min_ ratio < 2-fold which covers MTAP-null GI_90_ at trough and keeps *C*_max_ low to avoid concentrations that cover MTAP WT. In all, **TNG462** met the goals of the program and was advanced into pharmacology studies to measure *in vivo* activity.

**Table 9:** *In Vivo* PK Characterization of **TNG462**

species	clearance (mL min^–1^ kg^–1^)	Vd_ss_ (L/kg)	*T*_1/2_ (h)	F (%)	PPB (% unbound)^c^
dog^a^	23	21	14	52	26.8
cynomolgus monkey^b^	23	26	20	32	25.0

a IV/PO dosing in beagle dog (vehicle, IV: 1 mg/mL solution of 1% v/v DMSO/99% 20% w/v HP-β-CD in saline; PO: 3 mg/mL solution of 1% v/v DMSO/99% 20% w/v HP-β-CD in water, *n* = 3 per arm).

b IV/PO dosing in cynomolgus monkey (vehicle, IV: 1 mg/mL solution of 1% v/v DMSO/99% 20% w/v HP-β-CD in saline; PO: 3 mg/mL solution of 1% v/v DMSO/99% 20% w/v HP-β-CD in water, *n* = 3 per arm).

c Plasma protein binding, % unbound.

## **TNG462** Shows Extended Hold on PD and Strong Antitumor Efficacy in MTAP-Null Models

**Figure 10:**
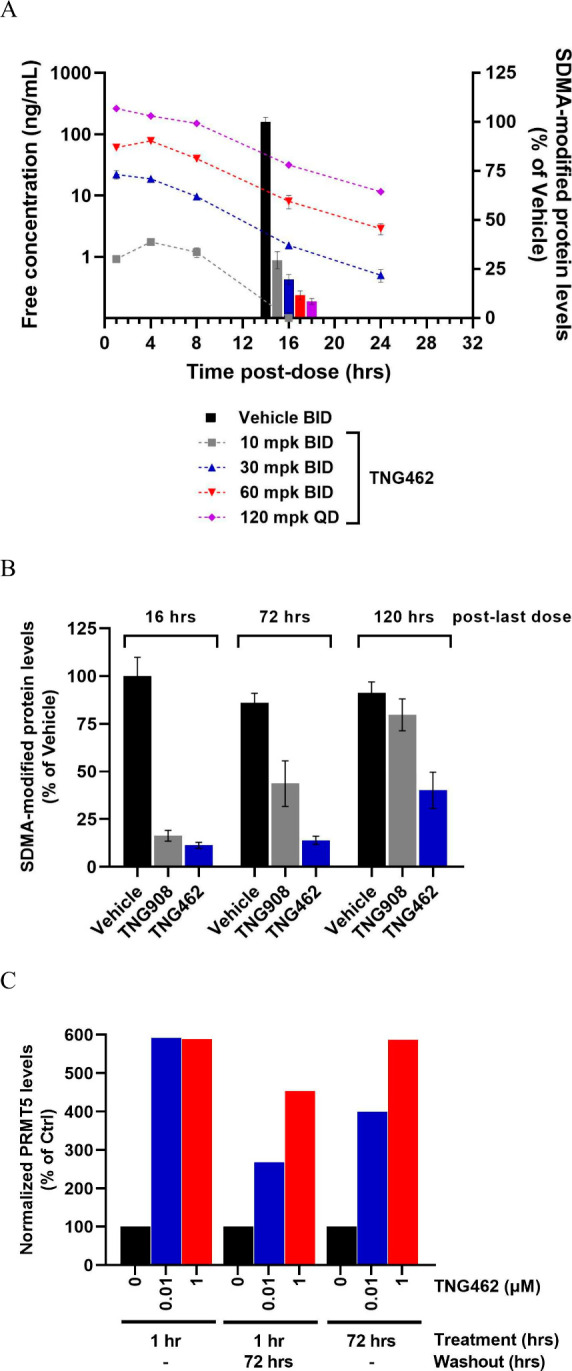
**TNG462** treatment drives dose-dependent and durable PD activity in the LU99 MTAP-null NSCLC xenograft model. PK/PD analysis of **TNG462** following compound treatment for 7-days. (A) PRMT5 activity by quantification of a single SDMA-modified protein substrate and free **TNG462** plasma concentrations were determined at the indicated time points postlast dose. *N* = 4 tumors/time point/dose level. Data are presented as mean ± SEM for the PD analyses. (B) SDMA-modified protein levels in tumors collected at the indicated time points postlast dose. TNG908 was dosed at 120 mg/kg BID and **TNG462** was dosed at 30 mg/kg BID for 7-days. *N* = 4 tumors/time point/treatment. Data are presented as mean ± SEM. (C) Cellular thermostability assay where the LN18 MTAP-null cancer cell line was treated with **TNG462** at the indicated concentrations and treatment conditions.

The pharmacodynamic activity of **TNG462** was evaluated in the LU99 MTAP-null NSCLC cell line-derived xenograft (CDX) model in a 7-day PK/PD study. **TNG462** was administered by oral gavage to tumor-bearing mice at well-tolerated doses (10, 30, or 60 mg/kg BID or 120 mg/kg QD) resulting in dose-dependent plasma exposures. For controls, tumor-bearing mice were also dosed with either vehicle or 120 mg/kg BID TNG908. Following 7-days of dosing, tumors were collected at 16, 72, or 120 h post-last dose and SDMA-modified protein levels were determined by immunoblot. Dose-dependent decreases in SDMA-modified protein levels were observed following **TNG462** treatment and it was further observed that **TNG462** was able to maintain a durable hold on PD response extending up to 120 h postlast dose whereas TNG908 only maintained a PD hold for 72 h (Figure 10A and B). These results could not be explained by exposure as the free exposures for each molecule were matched to ensure similar coverage of the *in vitro* PD IC_50_. The durable PD hold by **TNG462** observed *in vivo* was supported by an *in vitro* study demonstrating that **TNG462** conferred strong thermostability to cellular PRMT5 even after compound had been removed for 72 h in a washout study using the LN18 MTAP-null cancer cell line (Figure 10C).

**Figure 11:**
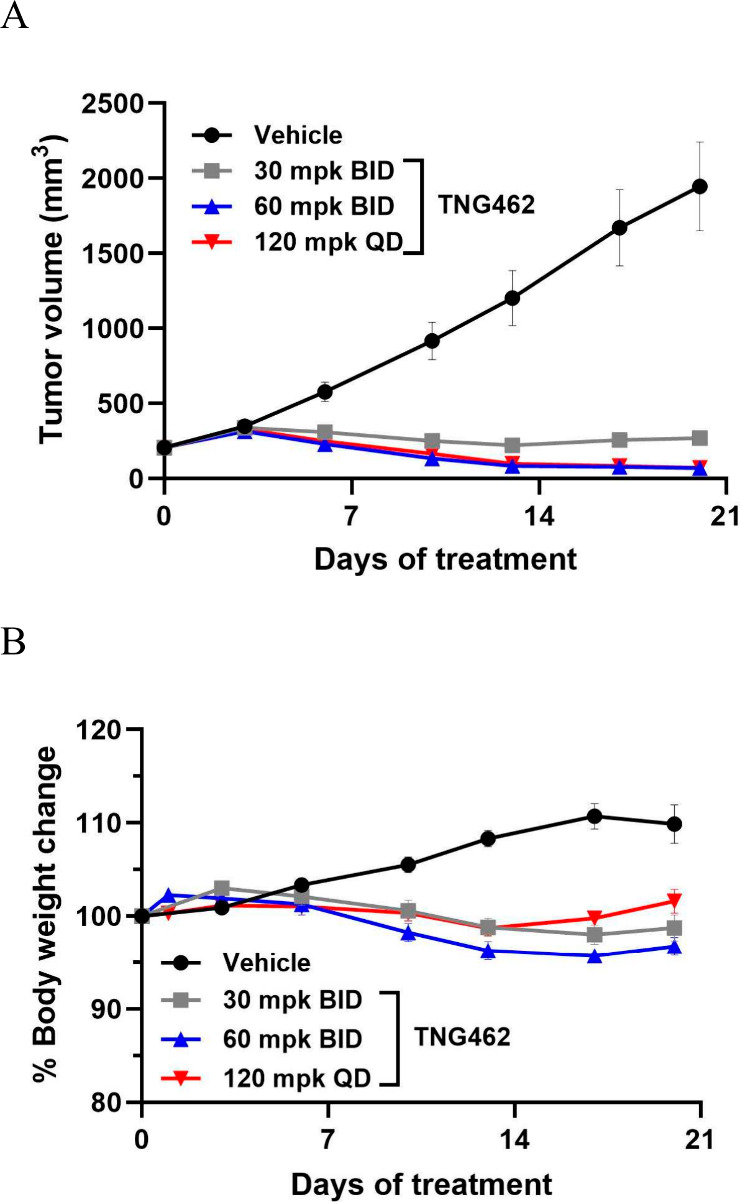
**TNG462** treatment drives dose-dependent antitumor activity at well-tolerated doses in the LU99 MTAP-null NSCLC xenograft model. (A) Efficacy and (B) tolerability were determined for **TNG462** at the indicated dose levels for 21 days. *n* = 8 mice/group. Data are presented as mean ± SEM.

Consistent with the pharmacodynamic activity of **TNG462**, strong antitumor activity was also demonstrated in the LU99 CDX model at 30 mg/kg BID, 60 mg/kg BID or 120 mg/kg QD (96% tumor growth inhibition (TGI), and 66 or 65% tumor regressions, respectively) (Figure 11).

**Figure 12:**
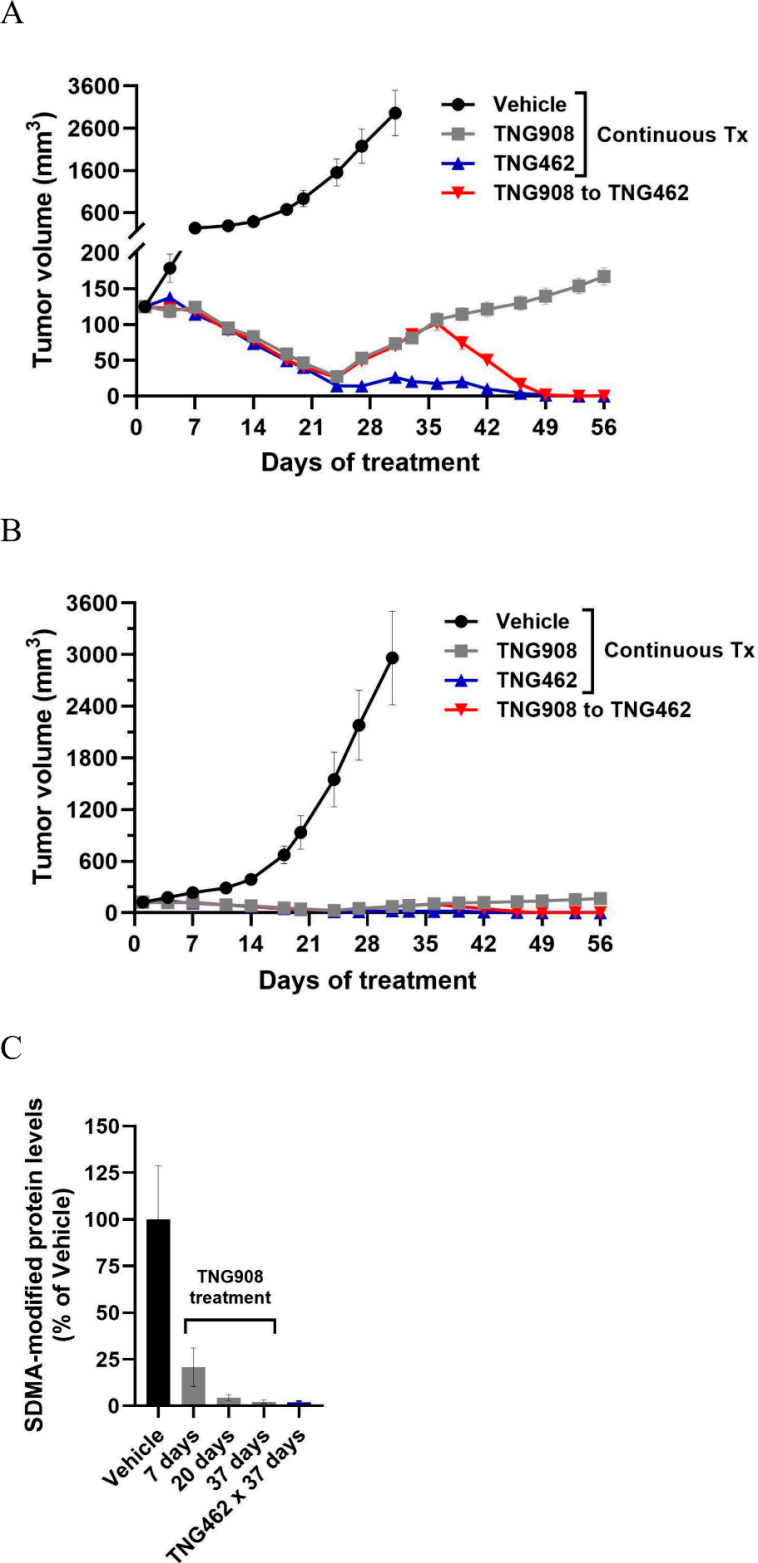
**TNG462** can resensitize an MTAP-null model with an incomplete response to TNG908. 40 mg/kg BID **TNG462** or 120 mg/kg BID TNG908 were dosed orally as indicated. *n* = 10–12 mice/group. Tumor volumes are plotted in (A) and (B). The *y*-axis in (A) is broken to highlight the region of interest. (C) Tumoral SDMA levels were determined at the indicated time points from satellite PD groups treated with TNG908 or **TNG462**.

As **TNG462** was more potent than TNG908 and with improved DMPK properties, we questioned whether **TNG462** could resensitize tumors with an incomplete response to TNG908. TNG908 treatment maintains strong tumor control in the OCI-LY19 MTAP-null DLBCL xenograft model, but there is a slow tumor regrowth after a tumor volume nadir at 24 days of treatment. When treatment was switched from 120 mg/kg BID TNG908 to 60 mg/kg BID **TNG462**, tumors immediately regressed again suggesting that the more potent **TNG462** restores sensitivity to PRMT5 inhibition (Figure 12).

**Figure 13:**
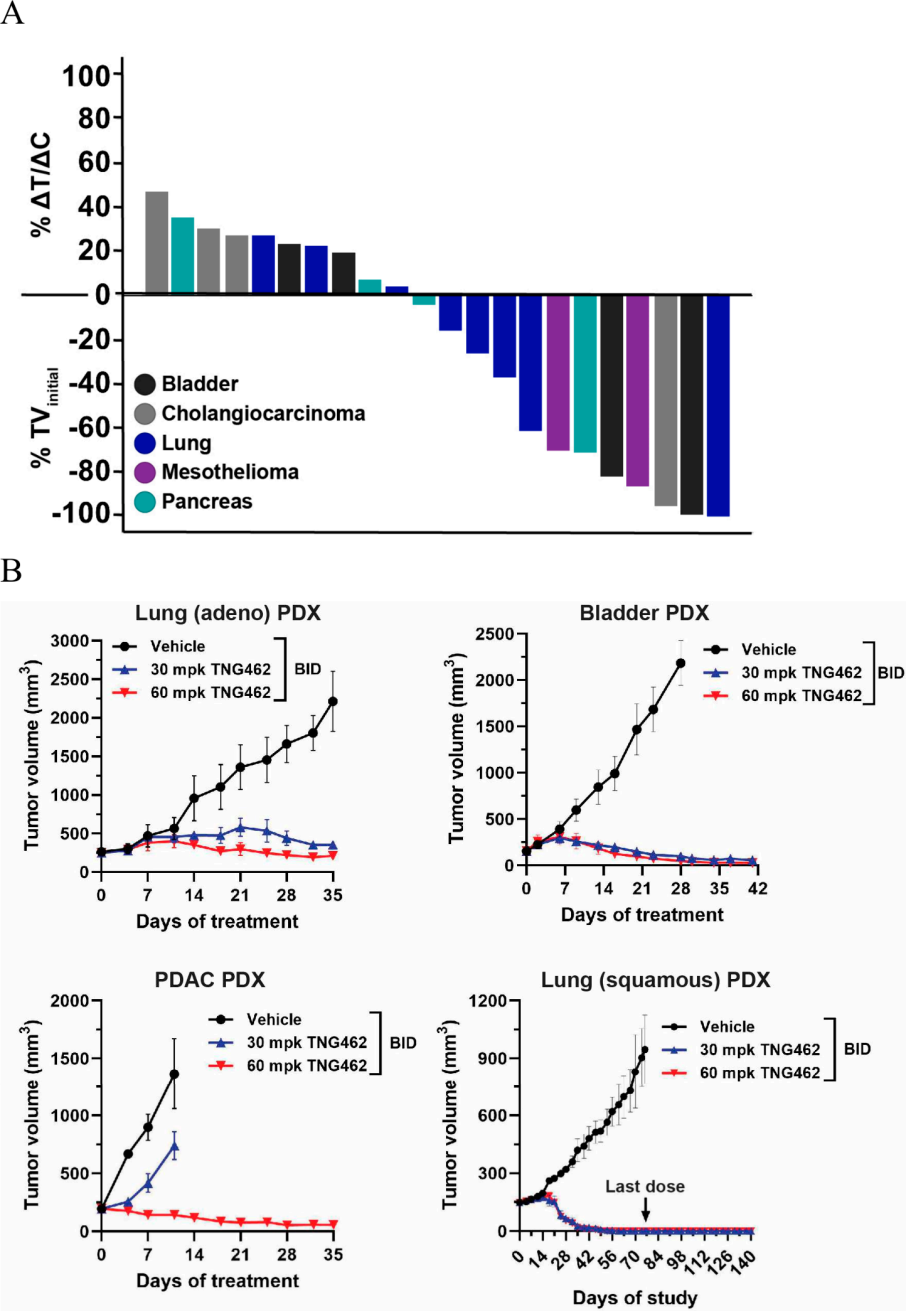
**TNG462** is efficacious in a panel of MTAP-null PDX models representing clinically relevant indications. (A) **TNG462** was dosed orally at 60 mg/kg BID. *n* = 3–5 mice/group/model. Lung PDX models represent either squamous or adenocarcinoma subtypes. (B) Selected PDX models from (A) representing the indicated tumor types. For the lung (squamous) PDX model, **TNG462** was dosed at either 30 or 60 mg/kg BID until day 76 when treatment was withdrawn. The mice were observed until day 140.

To evaluate whether the **TNG462** antitumor activity was limited by tumor histology, we also profiled **TNG462** in a panel of 22 MTAP-null patient-derived xenograft (PDX) models representing tumor histologies that are frequently MTAP-deleted. As a note, we excluded glioblastoma from our study as **TNG462** is not predicted to be blood-brain barrier penetrant (data not shown). Oral administration of 60 mg/kg BID **TNG462** drove strong antitumor activity across all PDX models evaluated, with 10 (45%) models demonstrating 53–96% TGI and 12 (55%) models demonstrating tumor shrinkage including a complete response that was maintained in a PDX model representing nonsmall cell lung cancer even after removal of treatment (Figure 13).

Collectively, these data demonstrate that **TNG462** drives on-target, dose- and concentration-dependent antitumor activity in *MTAP*-deleted xenograft models representing multiple tumor histologies. Indeed, single agent **TNG462** was able to drive tumor regressions, including a complete response, in xenograft models representing multiple tumor types, demonstrating the potential for **TNG462** to drive meaningful clinical responses in *MTAP*-deleted solid tumors.

## Synthesis

**Scheme 1:**
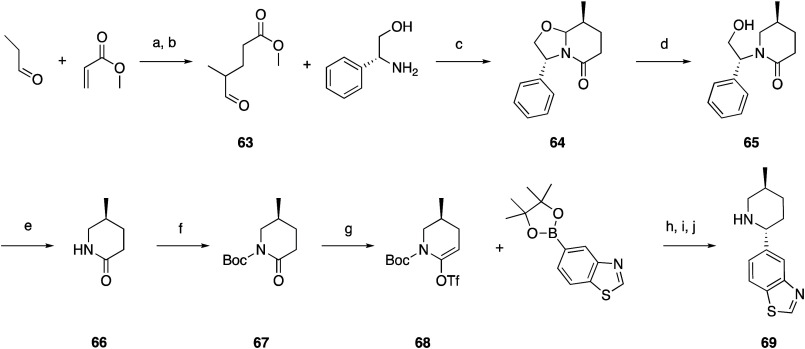
Synthesis of Intermediate **69** Reagents and conditions: (a) K_2_CO_3_, piperidine; (b) AcOH, H_2_O; (c) Na_2_SO_4_, Et_2_O, DCM; (d) Et_3_SiH, TiCl_4_, THF; (e) Na, NH_3_; (f) Boc_2_O, DMAP, DCM; (g) 1,1,1-trifluoro-*N*-phenyl-*N*-(trifluoromethylsulfonyl)methanesulfonamide, LHMDS, THF, from −78 to −25 °C; (h) Pd(dppf)Cl_2_, dioxane, H_2_O; (i) 4 M HCl, MeOH; (j) NaBH_4_, Na_2_CO_3_, MeOH.

Key intermediate **69** was prepared starting with propionaldehyde which was condensed with methyl acrylate via piperidine enamine to yield **63**. Lactam **64** was then stereoselectively prepared by cyclocondensation of **63** with (*R*)-phenylglycinol. Reductive cleavage of the oxazolidine C–O bond by treatment with titanium tetrachloride in the presence of triethylsilane, followed by treatment with sodium in liquid ammonia to cleave the benzylic C–N bond removed the chiral auxiliary. This yielded chiral lactam **66** which was Boc protected to **67**, followed by triflate formation (**68**). Suzuki coupling with 5-pinacol ester of benzothiazole gave the enamine, which upon Boc deprotection with TFA gave the imine, which was reduced with sodium borohydride, giving primarily the *trans* isomer (9:1 *trans*:*cis* ratio). This isomer was separated from the minor *cis* isomer through chiral chromatography to yield **69** (Scheme 1) which was used in later steps to form oxamides.

**Scheme 2:**
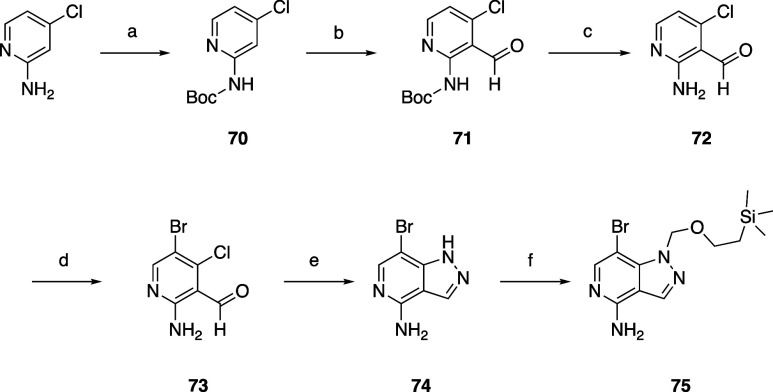
Synthesis of Intermediate **75** Reagents and conditions: (a) Boc_2_O, toluene; (b) nBuLi, NaHSO_4_·H_2_O, DMF, THF; (c) TFA, DCM; (d) NBS, ACN; (e) N_2_H_4_·H_2_O, dioxane; (f) (2-(chloromethoxy)ethyl)trimethylsilane, NaH, DMF.

The common pyrazolopyridine intermediate **75** was prepared starting with 4-chloropyridin-2-amine, which was Boc protected to **70**, then formylated to **71** with nBuLi and DMF. The Boc group was removed with TFA, and the 5-position was brominated to **73**. The pyrazole ring was formed with hydrazine in dioxane to **74**, followed by SEM protection to give **75** (Scheme 2) which was used in later steps to form oxamides.

**Scheme 3:**
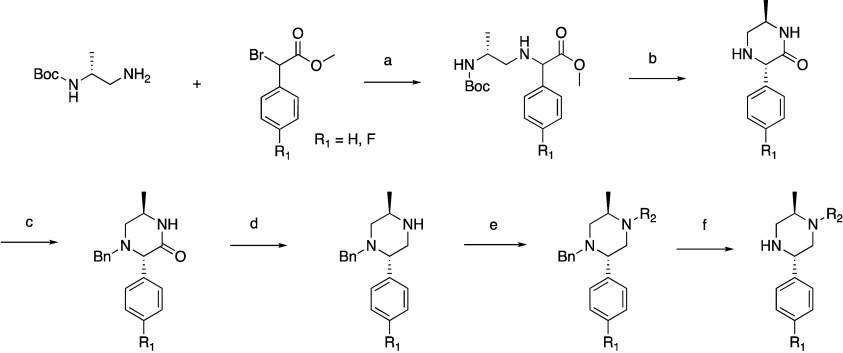
General Synthesis of Substituted Piperazines Used in **23**–**38**, **42**–**46**, and **48** Reagents and conditions: (a) TEA, DCM; (b) 4 M HCl/dioxane, MeOH; (c) benzyl chloride, TEA, DCM; (d) BH_3_·SMe_2_, THF; (e) amides: R_2_COCl, TEA, DCM; amines: R_2_-aldehyde or ketone, NaBH_3_CN, AcOH, DCM; (f) 10% Pd/C, AcOH, MeOH.

A common synthesis (Scheme 3) of substituted 2-aryl piperazines started with *tert*-butyl (*R*)-(1-aminopropan-2-yl)carbamate which displaced the bromo of either methyl 2-bromo-2-phenylacetate or its *p*-F variant in the presence of TEA in DCM to give methyl 2-(((*R*)-2-((*tert*-butoxycarbonyl)amino)propyl)amino)-2-phenylacetate or its *p*-F variant. These were then cyclized upon deprotection with HCl to the corresponding *trans*-piperazinone, which was benzyl protected then reduced to the piperazine with borane in THF. Amides could then be prepared with the appropriate acid chloride in the presence of TEA in DCM, and tertiary amines could be prepared through reductive amination with the appropriate aldehyde or ketone in the presence of NaBH_3_CN and acetic acid in DCM. Upon Boc deprotection the amine could be used in later steps to prepare oxamides.

**Scheme 4:**
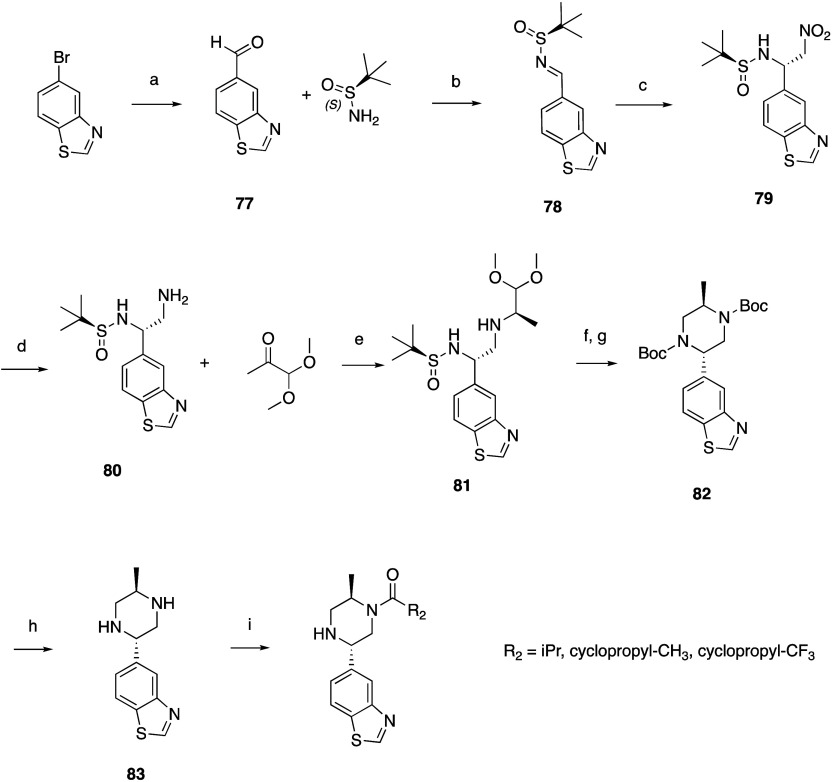
General Synthesis of Substituted Piperazine Benzothiazoles Reagents and conditions: (a) Pd(dppf)Cl_2_, TEA, TES, CO, DMF; (b) Ti(OCH_2_CH_3_)_4_, DCM; (c) nitromethane, KOtBu, THF; (d) NaBH_4_, NiCl_2_·H_2_O, MeOH; (e) Na_2_SO_4_, AcOH, NaBH_4_, DCE; (f) HCl/H_2_O, NaBH_3_CN; (g) Boc_2_O, K_2_CO_3_, MeOH, H_2_O; (h) HCl, MeOH; (i) R_2_-carboxylic acid, HATU, TEA, DMF.

Substituted 2-benzothiazole piperazines were prepared (Scheme 4) starting with 5-bromobenzothiazole which was converted to aldehyde **77** in the presence of Pd(dppf)Cl_2_, TEA, TES, and carbon monoxide in DMF. The *S* enantiomer of Ellman’s sulfinamide was used as a chiral auxiliary to form the *N*-*tert*-butanesulfinyl aldimine **78** in the presence of titanium(IV) ethoxide. Reductive homologation with nitromethane and potassium *sec*-butoxide yielded nitro compound **79** which was reduced to the amine **80** with sodium borohydride in the presence of NiCl_2_·H_2_O in methanol. Reductive amination with 1,1-dimethoxyacetone gave **81** which, upon treatment with HCl and NaBH_3_CN, cyclized to the piperazine, which was Boc-protected to enable isolation of **82**. Removal of the Boc groups with HCl in methanol yielded **83**. Selective acylation with the appropriate carboxylic acid in the presence of HATU and TEA in DMF yielded the corresponding piperazine intermediates, which were used in later steps to form oxamides.

**Scheme 5:**
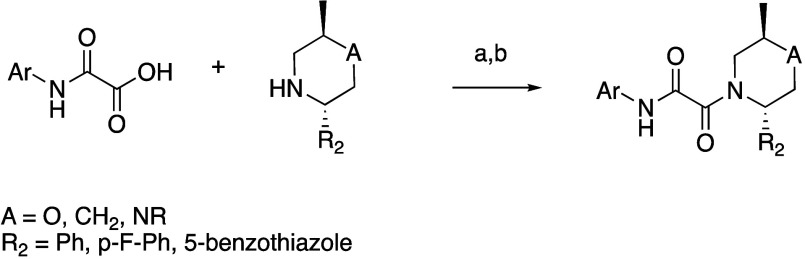
General Synthesis of Oxamides, Compounds **2**, **3**, **5**, **22**, **24**–**33**, **39**–**41**, **46**–**56**, **58**, and **62** Reagents and conditions: (a) HATU, TEA, DMF; (b) TFA, DCM (for **39**–**41** and **62**).

**Scheme 6:**
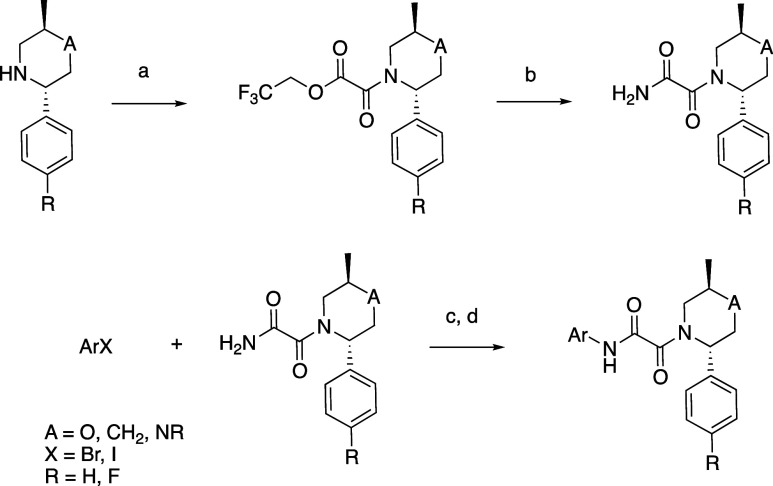
General Synthesis of Oxamides, Compounds **4**, **9**, **11**–**21**, **34**–**38**, **42**–**45**, **57**, and **59**–**61** Reagents and conditions: (a) 2,2,2-trifluoroethyl 2-chloro-2-oxoacetate, TEA, DCM; (b) NH_3_, MeOH; (c) (*S,S*)-(+)-*N*,*N*′-dimethyl-1,2-cyclohexanediamine, Cu, CuI, Cs_2_CO_3_, dioxane; (d) deprotection where appropriate.

**Scheme 7:**
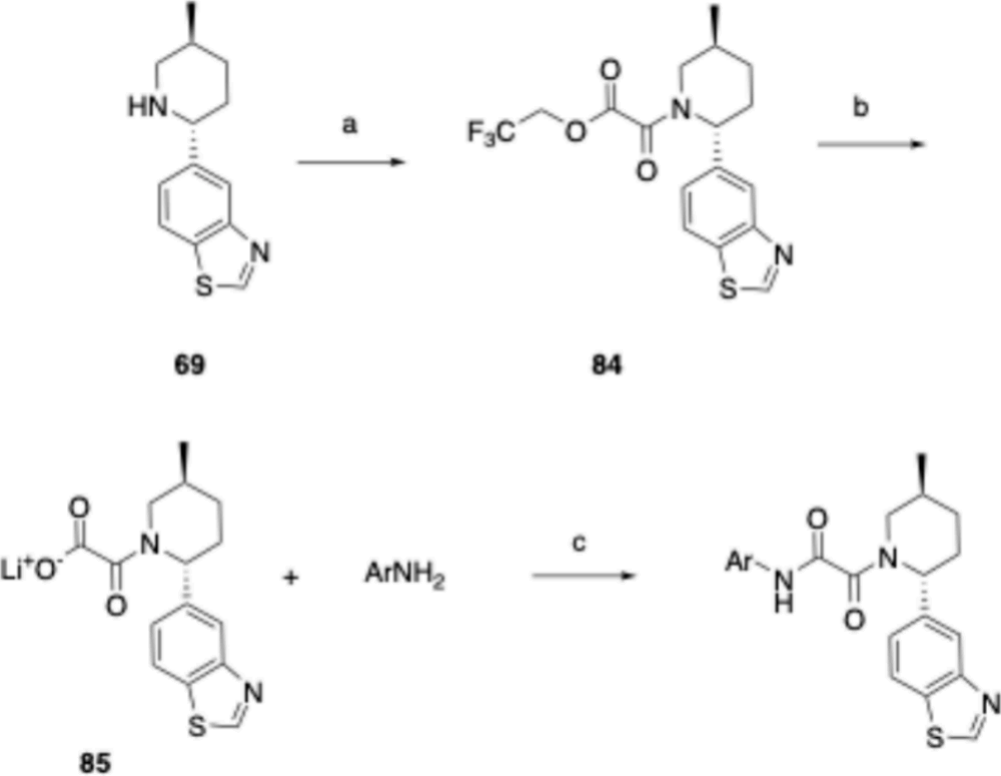
General Synthesis of Oxamides, Compounds **1**, **6**–**8**, and **10** Reagents and conditions: (a) 2,2,2-trifluoroethyl 2-chloro-2-oxoacetate, TEA, DCM; (b) LiOH·H_2_O, THF; (c) HATU, DMF.

Final oxamides were formed via several routes. Substituted pyridine oxamic acids could be coupled to the appropriate piperidine, piperazine, or morpholine with HATU, in the presence of TEA in DMF, followed by deprotection when appropriate (Scheme 5). Alternatively, morpholines, piperidines, or piperazines could be converted to the trifluoroethyl oxamic ester with 2,2,2-trifluoroethyl 2-chloro-2-oxoacetate in the presence of TEA in DCM, and this then converted to the primary oxamide with ammonia in methanol. The primary oxamide could then be converted to the final oxamide by copper-mediated cross coupling with the appropriate aryl halide and subsequent deprotection where needed (Scheme 6). For benzothiazole piperidine oxamides, **69** was converted to the trifluoroethyl oxamic ester **84** as previously described. This was then hydrolyzed with LiOH·H_2_O to the oxamate lithium salt, **85**. Coupling to the appropriate aryl amine with HATU in DMF led to the desired oxamide (Scheme 7).

**Scheme 8:**
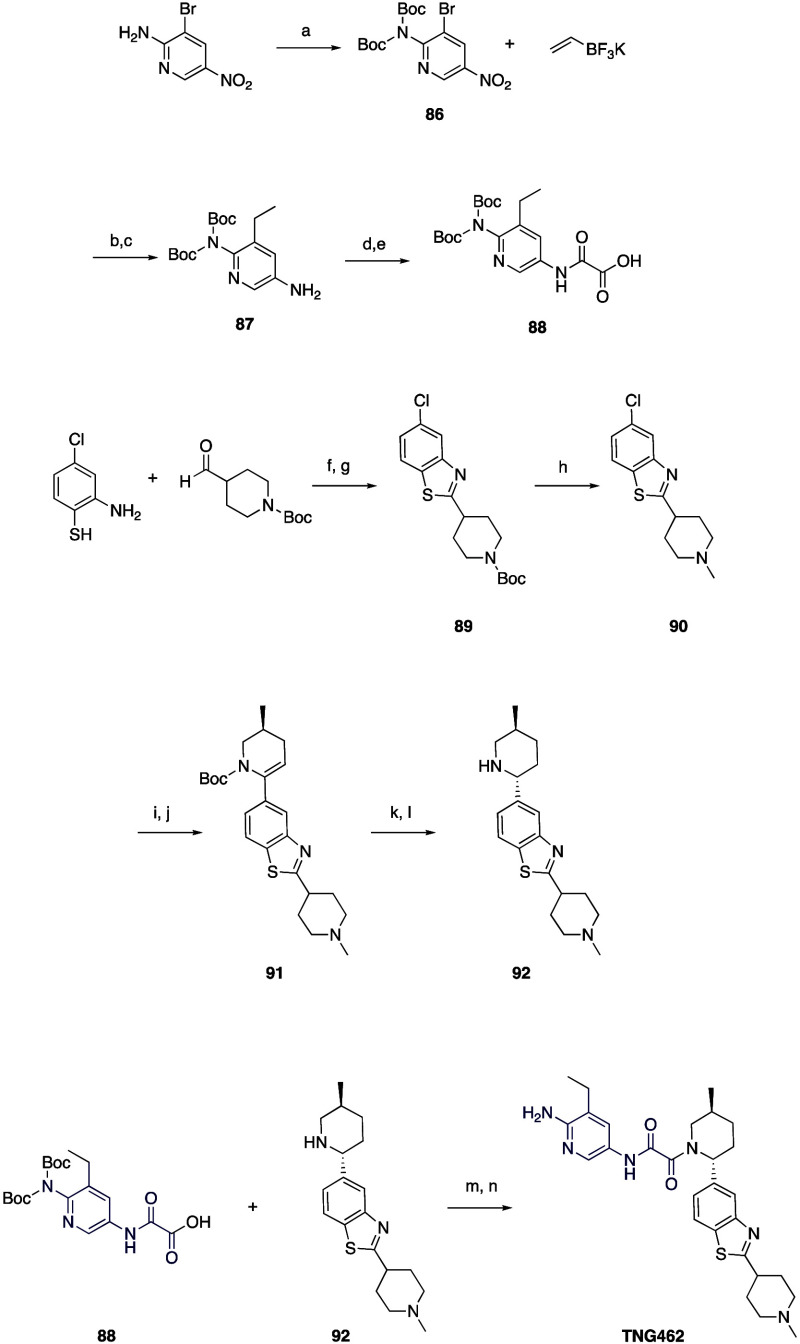
Synthesis of **TNG462** Reagents and conditions: (a) Boc_2_O, NaH, DMF, 97% yield; (b) K_2_CO_3_, Pd(dppf)Cl_2_, dioxane, H_2_O; (c) H_2_, Pd/C, MeOH, 91% yield, 2 steps; (d) ethyl 2-chloro-2-oxoacetate, DIPEA, ACN; (e) LiOH·H_2_O, MeOH, THF, 41% yield, 2 steps; (f) blue LED; (g) PCC, silica gel, 65% yield; (h) HCHO, HCOOH, 60% yield; (i) XPhos G2 Pd precatalyst, KOAc, (Bpin)_2_, dioxane; (j) **68**, Pd(dppf)Cl_2_, K_2_CO_3_, H_2_O, dioxane, 23% yield, 2 steps; (k) 4 M HCl, MeOH; (l) NaBH_4_, MeOH, 84% yield, 2 steps; (m) HATU, TEA, DMF; (n) 4 M HCl, MeOH, 19% yield, 2 steps.

The preparation of **TNG462** (Scheme 8) began with bis-Boc protection of 3-bromo-5-nitropyridin-2-amine using Boc_2_O and sodium hydride in DMF to give **86**. Suzuki-Miyaura coupling with vinyl potassium trifluoroborate, Pd(dppf)Cl_2_, and potassium carbonate with dioxane and water, followed by reduction of the nitro group with H_2_ and Pd/C in methanol led to **87**. Construction of the oxamate was achieved by formation of the ethyl ester with ethyl 2-chloro-2-oxoacetate and Hünig’s base in acetonitrile, followed by hydrolysis with lithium hydroxide in methanol and THF to give **88**. Concomitantly, the benzothiazole portion of the molecule was constructed starting with 2-amino-4-chlorobenzenethiol which was converted to the benzothiazole by a two-step reaction that includes the condensation with *tert*-butyl 4-formylpiperidine-1-carboxylate followed by conversion to **89** with pyridinium chlorochromate (PCC) on silica gel. The Boc group was converted to methyl via Eschweiler-Clarke methylation using formic acid and formaldehyde to give **90**. This was then converted to the pinacol boronate with XPhos G2 Pd precatalyst, pinacol diboron, and potassium acetate in dioxane, followed by Suzuki-Miyaura coupling to triflate **68** to yield the Boc protected enamine, **91**. Removal of the Boc group with 4 M HCl in methanol gave the imine which was reduced with sodium borohydride in methanol to give predominantly the desired *trans* isomer (9:1 *trans*:*cis*) **92**. Coupling with **88** using HATU and TEA in DMF gave the oxamide. The Boc groups were removed with 4 M HCl in methanol and residual *cis* isomer was removed with chiral SFC to give **TNG462**.

## Conclusions

In summary, a series of highly potent and selective compounds was discovered that inhibit PRMT5 cooperatively with MTA. Among these compounds is **TNG462**, which has a biochemical potency below the limit of assay detection with an estimated PRMT5·MTA *K*_i_ ≤ 300 fM, HAP1 MTAP-null SDMA IC_50_ = 800 pM, viability GI_50_ = 4 nM, and 45-fold selectivity against MTAP WT. **TNG462** has moderate clearance and bioavailability in preclinical species and a high volume of distribution, leading to a predicted human half-life >24 h, which supports QD dosing and provides a low *C*_max_/*C*_trough_ ratio which enables coverage of GI_90_ at trough. **TNG462** has strong efficacy across a panel of both cell-derived and patient-derived xenograft models including those representing pancreatic, nonsmall cell lung, mesothelioma, and bladder cancer, which all have a high prevalence of *MTAP* deletion, and demonstrates extended PD hold *in vivo*, with levels of SDMA below assay detection limit up to 72 h post-last dose. **TNG462** was nominated as a development candidate and is currently in Phase I/II clinical studies (NCT05732831).

## Experimental Section

### General Procedures

All chemicals were provided by Enamine Ltd., WuXi AppTec, or other commercial suppliers and used as received unless otherwise indicated. All solvents were treated according to standard methods. All reactions were monitored by LC-MS analysis using Agilent 1260 LC/MSD instruments with an Agilent Poroshell 120 SB-C18 4.6 mm × 30 mm, 2.7 μm column; column temperature = 60 °C; mobile phase A = water (0.1% formic acid), mobile phase B = acetonitrile (0.1% formic acid); flow rate = 1.5 mL/min; gradient: 0.01 min–1% B, 5.00 min–100% B, 5.99 min–100% B; MS ionization mode: electrospray ionization (ESI); MS scan range = 83–1000 *m*/*z*; UV detection at 215, 254, 280 nm, unless otherwise specified. Thin-layer chromatography (TLC) with precoated silica gel GF254 (0.2 mm) was used, and the results were visualized using either UV light or KMnO_4_ stain. Proton nuclear magnetic resonance (^1^H NMR) spectra were recorded at 400, 500, or 600 MHz on Varian or Bruker instrumentation; chemical shifts were calibrated using residual nondeuterated solvents CHCl_3_ (δ = 7.26 ppm), DMSO (δ = 2.50 ppm), or MeOH (δ = 3.31 ppm) and expressed in δ ppm. Coupling constants (*J*), when given, are reported in hertz. Multiplicities are reported using the following abbreviations: s = singlet, d = doublet, dd = doublet of doublets, t = triplet, q = multiplet (range of multiplets is given), br = broad signal, dt = doublet of triplets. ^19^F NMR spectra were recorded at 376 MHz (Varian), and ^13^C NMR spectra were recorded at 101, 126, or 151 MHz (Varian). ^13^C NMR chemical shifts are reported relative to the central CHCl_3_ (δ = 77.16 ppm), DMSO (δ = 39.52 ppm), or MeOH (δ = 49.00 ppm), and chemical shifts are reported in parts per million. All final compounds were purified by reverse phase high-performance liquid chromatography (HPLC) or supercritical fluid chromatography (SFC) or silica gel chromatography (100–200 mesh). HPLC was done with an Agilent 1260 HPLC instrument (Agilent Technologies, Germany) equipped with a G7161A Preparative Binary Pump, a G7157A Prep Autosampler, a G7115A DAD WR, and a G7159B Preparative Fraction Collector. The Open Lab CDS software (version C.01.10 was used for instrument control, data acquisition and data handling). SFC was done with a Waters 100q Prep SFC System. Chiral HPLC analytical analysis was done with an Agilent 1200 HPLC instrument (Agilent Technologies, Germany) equipped with a G1379B degasser, a G1312A Binary Pump, a G1329A ALS autosampler, a G1315A Diode Array Detector. Chiral SFC analytical analysis was done with an Agilent 1260 SFC instrument (Agilent Technologies, Germany) equipped with a G1379B degasser, a G1312B Binary Pump, a G1313A ALS autosampler, a G1316A thermostated column compartment, a G1315D Diode Array Detector and an Aurora SFC system. Melting points were taken using OptiMelt Automated Melting Point System Digital Image Processing Technology SRS Stanford Research Systems, 2 °C/min (5 °C/min at high melting point). Optical rotation was measured with an Anton Paar GmbH MCP 300 polarimeter (accuracy = ±0.003°). Standard conditions for analysis: solution concentration = 0.5 g/100 mL (methanol solvent), wavelength = 589 nm, temperature = 21 °C. All oxamides exist as rotamers in ^1^H NMR spectra. All compounds are >95% pure by HPLC.

#### Methyl 4-Methyl-5-oxopentanoate (**63**)

To a stirring mixture of piperidine, 99% (293.22 g, 3.44 mol, 340 mL) and potassium carbonate - granular (95.19 g, 688.72 mmol, 41 mL), propanal (100 g, 1.72 mol, 123 mL) was added dropwise. The reaction mixture was stirred at room temperature for 18 h. After 18 h, the reaction mixture was filtered through a pad of Na_2_SO_4_ and washed with MTBE (1000 mL). The filtrate was dried over Na_2_SO_4_, filtered, and concentrated under reduced pressure. The obtained crude enamine was dissolved in MeCN (1000 mL) and methyl prop-2-enoate (296.46 g, 3.44 mol, 310 mL) was added dropwise to the solution at room temperature. The resulting reaction mixture was refluxed for 16 h. After 16 h, acetic acid (206.79 g, 3.44 mol, 197 mL) was added followed by water (1000 mL) and the resulting solution was heated at reflux for an additional 16 h after which the mixture was cooled to room temperature, saturated with NaCl, and extracted with MTBE (2 × 1000 mL). The combined organic phase was dried over Na_2_SO_4_, filtered, and concentrated under reduced pressure to obtain crude methyl 4-methyl-5-oxopentanoate (**63**) (264 g, crude). The crude product was used for the next step without any further purification. ^1^H NMR (CDCl_3_, 500 MHz): δ 1.09 (d, 3H), 1.60–1.72 (m, 1H), 1.94–2.00 (m, 1H), 2.29–2.42 (m, 3H), 3.64 (s, 3H), 9.59 (s, 1H).

#### (3*R*,8*S*,8a*R*)-8-Methyl-3-phenyltetrahydro-2*H*-oxazolo[3,2-*a*]pyridin-5(3*H*)-one (**64**)

A mixture of **63** (67.8 g, 470.28 mmol), (2*R*)-2-amino-2-phenyl-ethanol (64.51 g, 470.28 mmol) and sodium sulfate, anhydrous (66.80 g, 470.28 mmol) in DCM (800 mL) and diethyl ether (200 mL) was stirred at 0 °C for 5 h. After 5 h, the resulting suspension was filtered, and the filtrate was concentrated under reduced pressure. The residue was purified by column chromatography (Interchim; 800 g SiO_2_; chloroform/acetonitrile with acetonitrile from 0–15%, flow rate = 120 mL/min, Rv = 4–10 cv.) to give product (3*R*,8*S*,8a*R*)-8-methyl-3-phenyltetrahydro-2*H*-oxazolo[3,2-*a*]pyridin-5(3*H*)-one (**64**) (59.8 g, 258.55 mmol, 55% yield) as a light-yellow gum. ^1^H NMR (CDCl_3_, 500 MHz): δ 1.20 (d, *J* = 6.3 Hz, 3H), 1.42–1.56 (m, 1H), 1.86–2.00 (m, 2H), 2.22–2.44 (m, 2H), 4.00 (dd, *J* = 8.8, 1.2 Hz, 1H), 4.07–4.17 (m, 1H), 4.42 (dd, *J* = 8.9, 3.2 Hz, 1H), 4.87–4.95 (m, 1H), 7.17–7.32 (m, 5H). LCMS (ESI): [M + H]^+^*m*/*z*: calcd 231.1; found 232.2; *t*_R_ = 1.12 min.

#### (*S*)-1-((*R*)-2-Hydroxy-1-phenylethyl)-5-methylpiperidin-2-one (**65**)

Triethylsilane (1.51 g, 12.97 mmol, 2.1 mL) and titanium tetrachloride (3.69 g, 19.46 mmol) were added to a solution of **64** (1 g, 4.32 mmol) in anhydrous DCM (25 mL) and the mixture was stirred at 40 °C for 24 h. Additional titanium tetrachloride (3.69 g, 19.46 mmol, 764 μL) and triethylsilane (1.51 g, 12.97 mmol, 2.1 mL) were added, and the stirring was continued at 50 °C for 24 h. The mixture was poured into saturated aqueous NaHCO_3_ (100 mL) solution. The aqueous phase was filtered over Celite and extracted with CH_2_Cl_2_. The combined organic extracts were dried over Na_2_SO_4_, filtered, and concentrated under reduced pressure to give a residue, which was purified by column chromatography (Companion Combiflash; 40 g SiO_2_; MTBE/methanol with methanol from 0–8%, flow rate = 40 mL/min, Rv = 9–11 cv.) to give product (*S*)-1-((*R*)-2-hydroxy-1-phenylethyl)-5-methylpiperidin-2-one (**65**) (0.51 g, 2.19 mmol, 51% yield) as a colorless oil. ^1^H NMR (CDCl_3_, 400 MHz): δ 0.93 (d, *J* = 6.4 Hz, 3H), 1.44–1.54 (m, 1H), 1.77–1.86 (m, 2H), 2.40–2.62 (m, 2H), 2.83 (t, *J* = 11.0 Hz, 1H), 2.92–2.99 (m, 1H), 4.04–4.21 (m, 2H), 5.71–5.79 (m, 2H), 7.17–7.37 (m, 5H). LCMS (ESI): [M + H]^+^*m*/*z*: calcd 233.1; found 234.2; *t*_R_ = 0.98 min.

#### (*S*)-5-Methylpiperidin-2-one (**66**)

Ammonia (500 mL) was condensed into a three-necked, 1000 mL, round-bottomed flask equipped with a coldfinger condenser at −78 °C. A solution of **65** (17.2 g, 73.72 mmol) in dry THF (100 mL) was added and the temperature was raised to −33 °C. Sodium (5.08 g, 221.17 mmol) was added in small portions until a blue color persisted, and the mixture was stirred at −33 °C for 3 min. The reaction was quenched by the addition of solid NH_4_Cl until the blue color disappeared, and then the mixture was stirred at room temperature for 5 h. CH_2_Cl_2_ was added, the solid was filtered, and the solvent was removed under reduced pressure to give (*S*)-5-methylpiperidin-2-one (**66**) (15 g, crude) as a light-yellow oil. The residue was used for the next step without any further purification. ^1^H NMR (DMSO-*d*_6_, 400 MHz): δ 1.01 (d, *J* = 6.6 Hz, 3H, CH3), 1.45–1.51 (m, 1H, H-4), 1.83–1.99 (m, 2H, H-4, H-5), 2.34 (ddd, *J* = 17.8, 10.8, 6.4 Hz, 1H, H-3), 2.43 (ddd, *J* = 17.8, 6.4, 3.5 Hz, 1H, H-3), 2.92 (t, *J* = 10.8 Hz, 1H, H-6), 3.26–3.33 (m, 1H, H-6), 6.10 (br.s, 1H, NH).

#### (*S*)-*tert*-Butyl 5-Methyl-2-oxopiperidine-1-carboxylate (**67**)

To a solution of **66** (15 g, 79.54 mmol, crude) and DMAP (971.7 mg, 7.95 mmol) in DCM (500 mL) was added di-*tert*-butyl dicarbonate (17.36 g, 79.54 mmol, 18.3 mL) dropwise at 21 °C. The resulting reaction mixture was stirred for 1 h. After 1 h, the resulting solution was diluted with 10% aq. HCl and brine, dried over Na_2_SO_4_ and concentrated under reduced pressure to obtain crude product, which was purified by column chromatography (Companion Combiflash, 330 g of SiO_2_, petroleum ether/MTBE with MTBE from 10–25%, flow rate = 100 mL/min, Rv = 6 CV) to give product (*S*)-*tert*-butyl 5-methyl-2-oxopiperidine-1-carboxylate (**67**) (9 g, 42.20 mmol, 53% yield) as a yellow solid. ^1^H NMR (400 MHz, DMSO-*d*_6_) δ 0.96 (d, *J* = 6.6 Hz, 3H), 1.42 (s, 9H),), 1.74–1.86 (m, 1H), 1.87–1.97 (m, 1H), 2.30–2.39 (m, 2H), 2.98–3.11 (m, 1H), 3.12 (s, 1H), 3.60–3.68 (m, 1H). LCMS (ESI): [M + H]^+^*m*/*z*: calcd 213.2; found 158.2; *t*_R_ = 1.24 min (*t*-Bu cleavage).

#### *tert*-Butyl (*S*)-3-Methyl-6-(((trifluoromethyl)sulfonyl)oxy)-3,4-dihydropyridine-1(2*H*)-carboxylate (**68**)

LiHMDS (1 M in THF/ethylbenzene, 1.05 L, 1.05 equiv) was added dropwise under argon to a cooled to −78 °C solution of **67** (180 g, 1.0 equiv) in THF (1.80 L). The resulting solution was stirred at −78 °C for 1.5 h followed by the addition of 1,1,1-trifluoro-*N*-phenyl-*N*-(trifluoromethylsulfonyl)methanesulfonamide (377 g, 1.25 equiv) in THF (1.2 L). The reaction mixture was stirred at −78 °C for 1 h then allowed to warm slowly to 25 °C. The reaction was quenched with 10% NaOH (2 L) at 10–20 °C. The organic layer was separated, the aqueous layer was extracted with ethyl acetate (1 L), the combined organic extracts were concentrated and the residue was purified by column chromatography (SiO_2_, petroleum ether/ethyl acetate; 150/1 to 80/1) to afford *tert*-butyl (*S*)-3-methyl-6-(((trifluoromethyl)sulfonyl)oxy)-3,4-dihydropyridine-1(2*H*)-carboxylate (**68**) (272 g, 90% yield, 96.6% purity) as a colorless oil. ^1^H NMR (400 MHz, CDCl_3_) δ 5.25 (t, *J* = 3.8 Hz, 1H), 3.87 (dd, *J* = 12.8, 3.3 Hz, 1H), 2.99 (dd, *J* = 12.7, 9.2 Hz, 1H), 2.45–2.34 (m, 1H), 1.97–1.89 (m, 1H), 1.89–1.76 (m, 1H), 1.49 (s, 9H), 0.99 (d, *J* = 6.5 Hz, 3H). LCMS (ESI): [M + H]^+^*m*/*z*: calcd 345.09; found 290.0 ([P – *t*-Bu]^+^); *t*_R_ = 1.408 min.

#### 5-((2*R*,5*S*)-5-Methylpiperidin-2-yl)benzo[*d*]thiazole (**69**)

**68** (115, 3333 mmol, 1 equiv) and 5-(4,4,5,5-tetramethyl-1,3,2-dioxaborolan-2-yl)benzo[*d*]thiazole (87.0 g, 333 mmol, 1.00 equiv) were stirred in H_2_O (575 mL) and dioxane (1.72 L), and Na_2_CO_3_ (106 g, 999 mmol, 3.00 equiv) was added. The suspension was degassed under vacuum and purged with N_2_ three times. Pd(dppf)Cl_2_ (12.2 g, 16.6 mmol, 0.05 equiv) was added and the suspension was degassed under vacuum and purged with N_2_ three times. The reaction was stirred for 12 h at 80 °C, then cooled to 20 °C and filtered. The solid was then washed with ethyl acetate (1.5 L) and the combined organics were concentrated. The residue was then dissolved with ethyl acetate (2.00 L) and washed with water (1.50 L). The aqueous layer was extracted with ethyl acetate (500 mL), and the combined organic layer was concentrated. The residue was purified by column chromatography (SiO_2_, petroleum ether/ethyl acetate = 40/1 to 8/1) to yield *tert*-butyl (*S*)-6-(benzo[*d*]thiazol-5-yl)-3-methyl-3,4-dihydropyridine-1(2*H*)-carboxylate (63.0 g, 191 mmol, 57% yield) as a yellow solid. ^1^H NMR: (400 MHz, DMSO-*d*_6_) δ 9.36 (s, 1H), 8.08 (d, *J* = 8.38 Hz, 1H), 7.90 (d, *J* = 1.38 Hz, 1H), 7.37–7.40 (m, 1H), 5.46 (t, *J* = 3.56 Hz, 1H), 3.91–3.95 (m, 1H), 3.04–3.10 (m, 1H), 2.37–2.47 (m, 1H), 1.80–1.98 (m, 2H), 0.98 (d, *J* = 6.38 Hz, 3H), 0.94 (s, 9H). *tert*-butyl (*S*)-6-(benzo[*d*]thiazol-5-yl)-3-methyl-3,4-dihydropyridine-1(2*H*)-carboxylate (63.0 g, 191 mmol, 1.00 equiv) was stirred in MeOH (250 mL) and HCl/MeOH (4 M, 477 mL, 10.0 equiv) was added. The reaction was stirred for 5 h at 20 °C after which the solvent was removed under reduced pressure. The residue was dissolved with water (500 mL) and saturated Na_2_CO_3_ was added to pH = 8–9. The aqueous layer was extracted with ethyl acetate (800 mL × 3) and the combined organic layer was concentrated to give (*S*)-5-(5-methyl-3,4,5,6-tetrahydropyridin-2-yl)benzo[*d*]thiazole (44.0 g, crude) as a colorless oil which was used without purification; ^1^H NMR (DMSO-*d*_6_, 500 MHz): δ 0.95 (m, 3H), 1.35 (m, 1H), 1.65 (m, 1H), 1.89 (m, 1H), 2.67 (m, 1H), 2.87 (d, 1H), 3.19 (t, 1H). 3.95 (d, 1H), 8.02 (d, 1H), 8.14 (d, 1H), 8.43 (s, 1H), 9.41 (s, 1H). (*S*)-5-(5-methyl-3,4,5,6-tetrahydropyridin-2-yl)benzo[*d*]thiazole (44.0 g, 191 mmol, 1.00 equiv) was stirred in MeOH (290 mL) and NaBH_4_ (11.2 g, 297 mmol, 1.56 equiv) was added at 0 °C. The reaction was stirred at 0 °C for 2 h, after which it was quenched with water (500 mL). The solvent was removed under reduced pressure and the aqueous layer was extracted with DCM (200 mL × 3). The combined organic layer was concentrated to give 5-((2*R*,5*S*)-5-methylpiperidin-2-yl)benzo[*d*]thiazole (**69**) as a yellow oil that was used in the next step reaction without purification; ^1^H NMR (DMSO-*d*_6_, 400 MHz): δ 0.91 (d, 3H), 1.18 (m, 1H), 1.30–1.60 (m, 6H), 2.47 (t, 1H), 3.17 (d, 1H), 3.73 (d, 1H), 7.52 (d, 1H). 7.88 (d, 1H), 8.12 (s, 1H), 8.98 (s, 1H). LCMS (ESI): [M + H]^+^*m*/*z*: calcd 232.1; found 233.0; *t*_R_ = 0.691 min.

#### *tert*-Butyl (4-Chloropyridin-2-yl)carbamate (**70**)

Di-*tert*-butyl dicarbonate (221 g, 1.01 mol, 232 mL) was added to the suspension of 4-chloropyridin-2-amine (100 g, 778 mmol) in toluene (1 L). The resulting reaction mixture was stirred at 80 °C for 16 h. The volatiles were then removed under reduced pressure and the residue was taken up in cold hexane (800 mL). The resulting gray precipitate was collected by filtration, and it was stirred with boiling chloroform (1.40 L) for 10 min and the resulting slurry was filtered while hot. The filtrate was concentrated under reduced pressure to afford *tert*-butyl (4-chloropyridin-2-yl)carbamate (**70**) (139 g, 610 mmol, 78% yield). ^1^H NMR (400 MHz, CDCl_3_) δ 9.25 (s, 1H), 8.21 (d, *J* = 5.3 Hz, 1H), 8.1 (s, 1H), 6.96 (dd, *J* = 5.3, 1.3 Hz, 1H), 1.54 (s, 9H).

#### *tert*-Butyl (4-Chloro-3-formylpyridin-2-yl)carbamate (**71**)

*n*-Butyllithium, 23% (2.5 M) in hexanes (128 g, 459 mmol, 185 mL, 23% purity) was added dropwise to a solution of **70** (50.0 g, 219 mmol) in tetrahydrofuran (1 L) at −70 °C under argon atmosphere. After the addition was complete, the resulting mixture was stirred at the same temperature for 40 min, then dimethylformamide (48.0 g, 656 mmol, 50.8 mL) was added quickly keeping the temperature below −80 °C. The cooling bath was removed, and the resulting mixture was slowly warmed to −40 °C. A solution of sodium hydrogen sulfate monohydrate (103 g, 743 mmol) in water (700 mL) was added in one portion and resulting mixture was stirred at ambient temperature for 20 min. It was then partitioned between ethyl acetate (1 L) and water (800 mL). The organic layer was washed with brine (2 × 500 mL), dried over anhydrous sodium sulfate and concentrated under reduced pressure to afford *tert*-butyl (4-chloro-3-formylpyridin-2-yl)carbamate (**71**) (50.3 g, 196 mmol, 90% yield). ^1^H NMR (400 MHz, CDCl_3_) δ 10.72 (br s, 1H), 10.53 (s, 1H), 8.51 (d, 1H), 7.05 (d, 1H), 1.54 (s, 9H).

#### 2-Amino-4-chloronicotinaldehyde (**72**)

Trifluoroacetic acid (112 g, 980 mmol, 75.5 mL) was added dropwise to the solution of **71** (50.3 g, 196 mmol) in dichloromethane (300 mL). The resulting mixture was stirred at 25 °C for 16 h. The volatiles were removed under reduced pressure and the residue was partitioned between DCM (500 mL) and 10% aq. K_2_CO_3_ solution (400 mL). The organic layer was separated, dried over anhydrous sodium sulfate, and concentrated *in vacuo* to afford 2-amino-4-chloronicotinaldehyde (**72**) (30.0 g, 192 mmol, 98% yield). ^1^H NMR (500 MHz, CDCl_3_) δ 10.43 (s, 1H), 8.87–8.16 (br s, 1H), 8.09 (d, *J* = 5.3 Hz, 1H), 6.65 (d, *J* = 4.9 Hz, 1H), 6.41–5.49 (br s, 1H).

#### 2-Amino-5-bromo-4-chloronicotinaldehyde (**73**)

*N*-Bromosuccinimide (39.2 g, 220 mmol, 18.7 mL) was added to a solution of **72** (30.0 g, 192 mmol) in acetonitrile (500 mL). The resulting mixture was stirred at 70 °C for 20 h. The solvent was then removed under reduced pressure and the residue was diluted with cold water (200 mL). The resulting yellow precipitate was filtered and dried, affording 2-amino-5-bromo-4-chloronicotinaldehyde (**73**) (42.0 g, 178 mmol, 93% yield). ^1^H NMR (400 MHz, DMSO) δ 10.31 (s, 1H), 8.45 (s, 1H), 8.28–7.83 (br s, 2H).

#### 7-Bromo-1*H*-pyrazolo[4,3-*c*]pyridin-4-amine (**74**)

Hydrazine monohydrate (53.6 g, 1.07 mol, 52.2 mL) was added to the solution of **73** (42.0 g, 178 mmol) in dioxane (450 mL). The resulting mixture was stirred at 100 °C for 48 h. The volatiles were removed under reduced pressure and the residue was diluted with cold water (150 mL). The resulting brown precipitate was filtered and dried, affording 7-bromo-1*H*-pyrazolo[4,3-*c*]pyridin-4-amine (**74**) (26.2 g, 123 mmol, 69% yield). LCMS (ESI): [M + H]^+^*m*/*z*: calcd 213.0; found 213.2; *t*_R_ = 0.288 min. ^1^H NMR (400 MHz, DMSO) δ 13.43 (br s, 1H), 8.24 (s, 1H), 7.67 (s, 1H), 6.80–6.90 (2H, br s).

#### 7-Bromo-1-((2-(trimethylsilyl)ethoxy)methyl)-1*H*-pyrazolo[4,3-*c*]pyridin-4-amine (**75**)

Sodium hydride (in oil dispersion) 60% dispersion in mineral oil (5.89 g, 154 mmol, 60% purity) was added portion-wise to a solution of **74** (26.2 g, 123 mmol) in dimethylformamide (250 mL) which was cooled to 10 °C. After H_2_ evolution ceased, 2-(chloromethoxy)ethyl-trimethyl-silane (22.6 g, 135 mmol, 23.9 mL) was added dropwise. The resulting mixture was then stirred at ambient temperature for 3 h, after which it was concentrated under reduced pressure and the residue was purified by gradient column chromatography (SiO_2_, CHCl_3_/ACN) affording 7-bromo-1-((2-(trimethylsilyl)ethoxy)methyl)-1*H*-pyrazolo[4,3-*c*]pyridin-4-amine (**75**) (14.8 g, 43.1 mmol, 35% yield), which exists as two regioisomers (protection at each nitrogen). ^1^H NMR (400 MHz, CDCl_3_) δ 7.94 (s, 2H), 5.98 (s, 2H), 5.11 (br s, 2H), 3.68–3.53 (t, 2H), 1–0.8 (t, 2H), 0.07 to −0.18 (m, 9H) and ^1^H NMR (400 MHz, CDCl_3_) δ 8.29 (s, 1H), 7.81 (s, 1H), 5.71 (s, 2H), 5.46–4.71 (br s, 2H), 3.74–3.59 (t, 2H), 1.01–0.83 (t, 2H), −0.03 (s, 9H). LCMS (ESI): [M + H]^+^*m*/*z*: calcd 342.05; found [343]+; *t*_R_ = 0.952 and 1.001 min.

#### Example of Synthesis of Substituted Piperazines (Scheme 3, R_1_ = F, R_2_ = CO(Me)iPr)

##### Methyl 2-(((*R*)-2-((*tert*-Butoxycarbonyl)amino)propyl)amino)-2-(4-fluorophenyl)acetate

Methyl 2-bromo-2-(4-fluorophenyl)acetate (1 equiv) was added dropwise to a solution of *tert*-butyl *N*-[(1*R*)-2-amino-1-methylethyl]carbamate (1 equiv) and TEA (2 equiv) in MeCN. The resulting mixture was stirred at 20 °C for 14 h at which point the volatiles were removed under reduced pressure and the residue was partitioned between 10% aq. K_2_CO_3_ solution and MTBE. The organic layer was separated, dried over Na_2_SO_4_ and concentrated under reduced pressure to obtain methyl 2-(((*R*)-2-((*tert*-butoxycarbonyl)amino)propyl)amino)-2-(4-fluorophenyl)acetate, which was used further without purification. LCMS (ESI): [M]^+^*m*/*z*: calcd 340.2; found 341.2; *t*_R_ = 1.036 min.

##### (3*S*,6*R*)-3-(4-Fluorophenyl)-6-methylpiperazin-2-one

Hydrogen chloride solution (4.0 M in dioxane, 4 equiv) was added portionwise to a solution of methyl 2-(((*R*)-2-((*tert*-butoxycarbonyl)amino)propyl)amino)-2-(4-fluorophenyl)acetate (1 equiv) in MeOH. The resulting mixture was stirred at 20 °C for 14 h after which the volatiles were removed under reduced pressure and the residue was partitioned between 25% aq. K_2_CO_3_ solution and DCM. The organic layer was separated and the aqueous layer was extracted with DCM twice. The combined DCM layers were dried over Na_2_SO_4_ and concentrated under vacuum. After the solvent was removed, the residue was heated to 80 °C under reduced pressure (approximately 15 Torr) for 1 h, then it was dissolved in boiling toluene. The resulting solution was left at 20 °C for 6 h. The obtained white crystals were filtered and dried, affording (3*S*,6*R*)-3-(4-fluorophenyl)-6-methylpiperazin-2-one. Yield: 31%. LCMS (ESI): [M]^+^*m*/*z*: calcd 208.2; found 209.2; *t*_R_ = 0.238 min.

##### (3*S*,6*R*)-4-Benzyl-3-(4-fluorophenyl)-6-methylpiperazin-2-one

Benzoyl chloride (1.10 g, 7.84 mmol) was added dropwise to a solution of (3*S*,6*R*)-3-(4-fluorophenyl)-6-methylpiperazin-2-one (2.1 g, 6.54 mmol, TFA) and TEA (1.98 g, 19.61 mmol, 2.7 mL) in DCM (30 mL) and the resulting mixture was stirred at 20 °C for 2 h after which 20% aq. K_2_CO_3_ solution (20 mL) was added and stirring was continued for 10 min then the organic layer was separated, dried over K_2_CO_3_, and concentrated under reduced pressure, affording (3*S*,6*R*)-4-benzyl-3-(4-fluorophenyl)-6-methylpiperazin-2-one (2.33 g, crude). LCMS (ESI): [M]^+^*m*/*z*: calcd 312.2; found 313.2; *t*_R_ = 1.047 min.

##### (2*S*,5*R*)-1-Benzyl-2-(4-fluorophenyl)-5-methylpiperazine

Borane dimethyl sulfide complex (2.66 g, 35.06 mmol, 3.3 mL) was added dropwise to a solution of (3*S*,6*R*)-4-benzyl-3-(4-fluorophenyl)-6-methylpiperazin-2-one (2.19 g, 7.01 mmol) in THF (30 mL). The resultant mixture was stirred at 65 °C for 18 h after which it was cooled to room temperature and the excess borane was destroyed by dropwise addition of MeOH (10 mL). After H_2_ evolution ceased, the volatiles were removed under reduced pressure and the residue was taken up in 2 M aq. HCl (40 mL) and stirred at 50 °C for 40 min. The resultant cloudy solution was filtered and extracted with DCM (2 × 10 mL). The DCM layers were discarded, and the aqueous layer was basified to pH ≈ 11 with solid potassium hydroxide. The precipitated amine was extracted with DCM (2 × 25 mL). The organic layers were separated, dried over K_2_CO_3_, and concentrated under vacuum, affording (2*S*,5*R*)-1-benzyl-2-(4-fluorophenyl)-5-methylpiperazine (1.31 g, 4.61 mmol, 66% yield). LCMS (ESI): [M]^+^*m*/*z*: calcd 284.2; found 285.2; *t*_R_ = 1.049 min.

##### ((2*R*,5*S*)-4-Benzyl-5-(4-fluorophenyl)-2-methylpiperazin-1-yl)(1-methylcyclopropyl)methanone

1-Methylcyclopropanecarbonyl chloride (500.31 mg, 4.22 mmol) was added dropwise to a solution of (2*S*,5*R*)-1-benzyl-2-(4-fluorophenyl)-5-methyl-piperazine (1 g, 3.52 mmol) and TEA (711 mg, 7.03 mmol, 980 μL) in DCM (30 mL). The mixture was stirred at 20 °C for 2 h after which a 15% aq. K_2_CO_3_ solution (20 mL) was added, and stirring was continued for 10 min. The organic layer was then separated, dried over K_2_CO_3_, and concentrated under reduced pressure, affording [(2*R*,5*S*)-4-benzyl-5-(4-fluorophenyl)-2-methylpiperazin-1-yl]-(1-methylcyclopropyl)methanone (1.35 g, crude). LCMS(ESI): [M]^+^*m*/*z*: calcd 366.2; found 367.2; *t*_R_ = 1.372 min.

##### ((2*R*,5*S*)-5-(4-Fluorophenyl)-2-methylpiperazin-1-yl)(1-methylcyclopropyl)methanone

Palladium, 10% on carbon (350 mg, 328.89 μmol) was added to a solution of [(2*R*,5*S*)-4-benzyl-5-(4-fluorophenyl)-2-methyl-piperazin-1-yl]-(1-methylcyclopropyl)methanone (1.35 g, 3.68 mmol) in MeOH (30 mL) and acetic acid (10 mL). The flask was evacuated and backfilled with H_2_ from an attached balloon. The resulting mixture was stirred at 50 °C for 16 h, then the catalyst was filtered off and the filtrate was concentrated under reduced pressure. The residue was partitioned between a 10% aq. K_2_CO_3_ solution (20 mL) and DCM (40 mL). The organic layer was separated, dried over K_2_CO_3_, and concentrated under vacuum, affording [(2*R*,5*S*)-5-(4-fluorophenyl)-2-methylpiperazin-1-yl]-(1-methylcyclopropyl)methanone (0.96 g, 3.47 mmol, 94% yield). LCMS(ESI): [M]^+^*m*/*z*: calcd 276.2; found 277.2; *t*_R_ = 0.702 min.

#### Benzo[*d*]thiazole-5-carbaldehyde (**77**)

A mixture of 5-bromo-1,3-benzothiazole (25 g, 116.78 mmol), Pd(dppf)Cl_2_ (4.37 g, 5.98 mmol), TEA (39.93 g, 394.60 mmol, 55.00 mL), TES (43.75 g, 376.26 mmol), CO, and DMF (100 mL) was stirred at 80 °C for 12 h under CO (50 psi). The resulting mixture was quenched with water (100 mL) and extracted with EtOAc (200 mL × 3). The combined organic layer was washed with saturated NH_4_Cl aqueous solution (100 mL × 2), brine (100 mL), dried over anhydrous Na_2_SO_4_, filtered, and concentrated under reduced pressure to give residue which was purified by flash chromatography (ISCO; 80 g of AgelaFlash Silica Flash Column, petroleum ether/EtOAc with EtOAc from 0–50%, flow rate = 30 mL/min, to afford benzo[*d*]thiazole-5-carbaldehyde (**77**) (13 g, 79.66 mmol, 68% yield) as a yellow solid.

#### (*S*,*E*)-*N*-(Benzo[*d*]thiazol-5-ylmethylene)-2-methylpropane-2-sulfinamide (**78**)

A mixture of **77** (10 g, 61.28 mmol), 2-methylpropane-2-sulfinamide (10.00 g, 82.51 mmol), titanium tetraethoxide (43.60 g, 191.14 mmol, 40.00 mL) and DCM (20 mL) was stirred at 20 °C for 12 h. The resulting mixture was quenched with water (100 mL) and extracted with DCM (100 mL × 3). The combined organic layer was washed with saturated NH_4_Cl aqueous solution (100 mL × 2), brine (100 mL), dried over anhydrous Na_2_SO_4_, filtered, and concentrated under reduced pressure to afford (*S*,*E*)-*N*-(benzo[*d*]thiazol-5-ylmethylene)-2-methylpropane-2-sulfinamide (**78**) (10 g, 37.54 mmol, 61% yield) as a yellow solid. LCMS (ESI) [M + H]^+^*m*/*z*: calcd 267.0; found 267.0. ^1^H NMR (400 MHz, methanol-*d*_4_) δ 9.39 (s, 1 H), 8.76 (s, 1 H), 8.58 (br s, 1 H), 8.19–8.32 (m, 1 H), 8.09 (br d, *J* = 8.1 Hz, 1 H), 1.32 (s, 10 H).

#### (*S*)-*N*-((*S*)-1-(Benzo[*d*]thiazol-5-yl)-2-nitroethyl)-2-methylpropane-2-sulfinamide (**79**)

A mixture of KOtBu (1 M in THF, 200 mL) and THF (100 mL) was cooled to 0 °C. Nitromethane (47.46 g, 777.52 mmol, 42 mL) was added and the reaction was stirred for 1 h. **78** (10 g, 37.54 mmol) was added at 0 °C and then stirred at 20 °C for 47 h. The mixture was concentrated under reduced pressure to give a residue which was purified by flash chromatography (ISCO; 40 g of AgelaFlash Silica Flash Column, EtOAc/MeOH with MeOH from 0–10%, flow rate = 30 mL/min) to afford (*S*)-*N*-((*S*)-1-(benzo[*d*]thiazol-5-yl)-2-nitroethyl)-2-methylpropane-2-sulfinamide (**79**) (8 g, 24.43 mmol, 65% yield) as a yellow oil. ^1^H NMR (400 MHz, methanol-*d*_4_) δ 9.31 (s, 1 H), 8.08–8.21 (m, 2 H), 7.58 (dd, *J* = 8.4, 1.6 Hz, 1 H), 5.31–5.40 (m, 1 H), 5.06–5.15 (m, 1 H), 4.98 (dd, *J* = 13.4, 6.3 Hz, 1 H), 1.21 (s, 9 H). LCMS (ESI) [M + H]^+^*m*/*z*: calcd 328.1, found 328.1

#### (*S*)-*N*-((*S*)-2-Amino-1-(benzo[*d*]thiazol-5-yl)ethyl)-2-methylpropane-2-sulfinamide (**80**)

A mixture of **79** (7.5 g, 22.91 mmol), dichloronickel;hexahydrate (6 g, 25.24 mmol), and MeOH (50 mL) was cooled to 0 °C. NaBH_4_ (4.4 g, 116.30 mmol) was added slowly and stirred at 0 °C for 2 h. The mixture was filtered and concentrated under reduced pressure to give (*S*)-*N*-((*S*)-2-amino-1-(benzo[*d*]thiazol-5-yl)ethyl)-2-methylpropane-2-sulfinamide (**80**) (7.5 g, crude) as black oil that was used as is. LCMS (ESI) [M + H]^+^*m*/*z*: calcd 298.1, found 298.0.

#### (*S*)-*N*-((*S*)-1-(Benzo[*d*]thiazol-5-yl)-2-(((*R*)-1,1-dimethoxypropan-2-yl)amino)ethyl)-2-methylpropane-2-sulfinamide (**81**)

A mixture of (*S*)-*N*-((*S*)-2-amino-1-(benzo[*d*]thiazol-5-yl)ethyl)-2-methylpropane-2-sulfinamide (7.5 g, 25.22 mmol), 1,1-dimethoxypropan-2-one (8 g, 67.72 mmol), Na_2_SO_4_ (13 g, 91.52 mmol), AcOH (1.50 g, 30.74 mmol), and DCE (100 mL) was stirred at 20 °C for 12 h. NaBH_4_ (3.00 g, 79.18 mmol) was added and the mixture was stirred at 20 °C for 1 h. The resulting mixture was quenched by addition of water (50 mL) and extracted with DCM (100 mL × 3). The combined organic layer was washed with saturated NH_4_Cl aqueous solution (50 mL × 2), brine (50 mL), dried over anhydrous Na_2_SO_4_, filtered, and concentrated under reduced pressure to give (*S*)-*N*-((*S*)-1-(benzo[*d*]thiazol-5-yl)-2-(((*R*)-1,1-dimethoxypropan-2-yl)amino)ethyl)-2-methylpropane-2-sulfinamide (**81**) (11 g, crude) as a yellow oil. LCMS (ESI) [M + H]^+^*m*/*z*: calcd 400.2, found 400.1.

#### Di-*tert*-butyl (2*S*,5*R*)-2-(Benzo[*d*]thiazol-5-yl)-5-methylpiperazine-1,4-dicarboxylate (**82**)

A mixture of **81** (4.5 g, 11.3 mmol) and HCl/H_2_O (12 M, 12 mL) was stirred at 20 °C for 1 h after which the mixture was concentrated under reduced pressure to give a residue which was diluted with MeOH (30 mL). NaBH_3_(CN) (700 mg, 11.1 mmol), Na_2_SO_4_ (5.4 g, 38.0 mmol), and MeOH (30 mL) were added, and the mixture was stirred at 20 °C for 12 h. Boc_2_O (8.56 g, 39.2 mmol), K_2_CO_3_ (4.8 g, 34.7 mmol), and H_2_O (15 mL) were added and the mixture was stirred at 20 °C for 2 h. The resulting mixture was quenched by addition of water (100 mL) and extracted with EtOAc (100 mL × 3). The combined organic layer was washed with brine (100 mL), dried over anhydrous Na_2_SO_4_, filtered, and concentrated under reduced pressure to give a residue which was purified by flash chromatography (ISCO; 12 g of AgelaFlash Silica Flash Column, petroleum ether/EtOAc with EtOAc from 0–30%, flow rate = 30 mL/min, 254 nm) to afford di-*tert*-butyl (2*S*,5*R*)-2-(benzo[*d*]thiazol-5-yl)-5-methylpiperazine-1,4-dicarboxylate (**82**) (0.65 g, 13% yield) as a yellow oil. LCMS (ESI) [M + H]^+^*m*/*z*: calcd 434.2, found 234.3.

#### 5-((2*S*,5*R*)-5-Methylpiperazin-2-yl)benzo[*d*]thiazole (**83**)

A mixture of **82** (1.00 g, 2.31 mmol) and MeOH/HCl (4 M, 5 mL) was stirred at 20 °C for 2 h. The mixture was concentrated under reduced pressure to give 5-((2*S*,5*R*)-5-methylpiperazin-2-yl)benzo[*d*]thiazole (**83**) (560 mg, crude, 2·HCl) as a yellow solid. LCMS (ESI) [M + H]^+^*m*/*z*: calcd 234.1, found 234.2.

#### 1-((2*R*,5*S*)-5-(Benzo[*d*]thiazol-5-yl)-2-methylpiperazin-1-yl)-2-methylpropan-1-one (**39b**) (Scheme 4, R_2_ = iPr)

To a solution of **83** (100 mg, 0.327 mmol, 2·HCl) in DMF (3 mL) was added HATU (140 mg, 0.368 mmol) and TEA (0.200 mL, 1.43 mmol). 2-methylpropanoic acid (30 mg, 0.341 mmol) in DMF (2 mL)) was added dropwise at −30 °C. The resulting mixture was stirred at 20 °C for 2 h. The mixture was concentrated and purified by flash chromatography (Column: SepaFlash Spherical C18, 15 g, 40–60 μm, 120 Å; MeCN/water (0.05 v % NH_3_–H_2_O) with MeCN from 0–30%, 25 mL/min, 254 nm) to afford 1-((2*R*,5*S*)-5-(benzo[*d*]thiazol-5-yl)-2-methylpiperazin-1-yl)-2-methylpropan-1-one (**39b**) (70 mg, crude) as a white solid. LCMS (ESI) [M + H]^+^*m*/*z*: calcd 304.1, found 304.2. See other examples in the Supporting Information.

#### General Procedure for Scheme 5

The appropriate piperidine, piperazine, or morpholine (1 equiv), oxamic acid (1 equiv), and TEA (2.5 equiv + 1.0 equiv per each acid equivalent, if amine salt used) were stirred in DMF. HATU (1.5 equiv) was added, and the resulting mixture was stirred overnight. The reaction mixture was concentrated under vacuum and the residue was purified by HPLC to obtain the desired product. When appropriate, the material was deprotected as indicated and/or isomers were separated by chiral SFC or HPLC. See details in the Supporting Information.

#### General Procedure for Scheme 6

2,2,2-Trifluoroethyl 2-chloro-2-oxoacetate (1.2 equiv) was slowly added to a stirred solution of the appropriate piperazine or piperidine (1 equiv) and TEA (2 equiv) in dry THF (0.2 M) at 25 °C. The resulting mixture was stirred at 25 °C for 0.5 h, then NH_3_(g) was bubbled through the reaction mixture at 25 °C for 0.5 h. The resulting ammonium chloride precipitate was filtered and discarded, the filtrate was concentrated in vacuum to afford the corresponding oxamide-NH_2_ which was added to a mixture of halopyridine (1 equiv), (*S*,*S*)-(+)-*N*,*N*′-dimethyl-1,2-cyclohexanediamine (1 equiv), Cu (0.5 equiv), CuI (0.1 equiv), and Cs_2_CO_3_ (2 equiv) in dioxane (0.18 M). The resulting mixture was stirred at 100 °C for 18 h, then filtered through a pad of Celite and concentrated *in vacuo*. The residue was purified by HPLC. Where indicated the material was deprotected. Where indicated the material was further purified with chiral SFC or similar. See details in the Supporting Information.

#### 2,2,2-Trifluoroethyl 2-((2*R*,5*S*)-2-(Benzo[*d*]thiazol-5-yl)-5-methylpiperidin-1-yl)-2-oxoacetate (**84**)

To a stirred solution of **69** (9.70 g, 41.75 mmol) in dichloromethane (200 mL) was added triethylamine (8.7 mL, 62.62 mmol) at room temperature. The resulting reaction mixture was cooled to 0 °C, then 2,2,2-trifluoroethyl 2-chloro-2-oxo-acetate (8.35 g, 43.84 mmol) was added dropwise. The reaction was stirred for 30 min at 0 °C then allowed warmed to room temperature and stirred for 16 h. The reaction mixture was concentrated under reduced pressure to obtain 22.2 g of crude 2,2,2-trifluoroethyl 2-((2*R*,5*S*)-2-(benzo[*d*]thiazol-5-yl)-5-methylpiperidin-1-yl)-2-oxoacetate (**84**) as a yellow gum, which was used for the next step without purification. LCMS (ESI): [M + H]^+^*m*/*z*: calcd 386.11; found [387]+; *t*_R_ = 3.659.

#### Lithium 2-[(2*R*,5*S*)-2-(1,3-Benzothiazol-5-yl)-5-methyl-1-piperidyl]-2-oxoacetate (**85**)

To a solution of **84** (5.05 g, 13.07 mmol) in THF (50 mL) was added lithium hydroxide monohydrate, 98% (548.45 mg, 13.07 mmol) as a solution in water (10 mL) and the resulting mixture was left to stir at room temperature for 1 h, after which the mixture was evaporated to dryness, dissolved in water, and washed with DCM three times. The aqueous layer was acidified to pH = 1 and extracted with EtOAc twice. The combined organics were washed with brine, dried over Na_2_SO_4_, and evaporated to afford lithium 2-[(2*R*,5*S*)-2-(1,3-benzothiazol-5-yl)-5-methyl-1-piperidyl]-2-oxoacetate (**85**) (4.47 g, crude) which was used as a lithium salt in the next steps without further purification. LCMS (ESI): [M + H]^+^*m*/*z*: calcd 304.36; found [305]+; *t*_R_ = 1.347 min.

#### General Procedure for Oxamide Formation in Scheme 7

Aryl amine (1 equiv) and HATU (1.05 equiv) were stirred in DMF (0.2 M) at room temperature. The mixture was stirred for 15 min after which oxamic acid, lithium salt (1 equiv) was added. The solution was stirred overnight, after which it was poured into water and extracted with EtOAc. The combined organic layers were washed with water, then brine, then concentrated *in vacuo*. The residue was purified as described and deprotected where appropriate. See details in the Supporting Information.

#### *tert*-Butyl (3-Bromo-5-nitropyridin-2-yl)(*tert*-butoxycarbonyl)carbamate (**86**)

3-bromo-5-nitropyridin-2-amine (1.50 kg, 6.88 mol) was stirred in THF (7.50 L) at 0 °C. DMAP (168 g, 1.38 mol) was added, and the mixture was stirred for 0.5 h at 0 °C. Boc_2_O (3.15 kg, 14.4 mol, 3.32 L) in THF (3.00 L) was added and the reaction mixture was stirred for 2 h at 25 °C. The reaction mixture was poured into water (15.0 L) and separated. The aqueous layer was extracted with EtOAc (5.00 L × 2) and the combined organic layer was dried with Na_2_SO_4_ and concentrated to give *tert*-butyl (3-bromo-5-nitropyridin-2-yl)(tert-butoxycarbonyl)carbamate (**86**) (5.60 kg, 97% yield) as a white solid which was used without further purification in the next reaction. ^1^H NMR (400 MHz CDCl_3_) δ 9.28 (d, *J* = 2.41 Hz, 1H), 8.75 (d, *J* = 2.41 Hz, 1H), 1.43 (s, 18H).

#### *tert*-Butyl (5-Amino-3-ethylpyridin-2-yl)(*tert*-butoxycarbonyl)carbamate (**87**)

**86** (2.80 kg, 6.69 mol) was stirred in dioxane (22.4 L) and H_2_O (5.60 L). Potassium trifluoro(vinyl)boronate (1.08 kg, 8.03 mol) was added at 25 °C. K_2_CO_3_ (4.63 kg, 33.4 mol) was then added, and the vessel was purged with Ar three times. Pd(dppf)Cl_2_·CH_2_Cl_2_ (218 g, 267 mmol) was added, and the reaction mixture was stirred for 2 h at 80 °C after which it was poured into water (28.0 L) and separated. The aqueous layer was extracted with EtOAc (10.0 L × 2) and the organic layer was dried with Na_2_SO_4_ and concentrated. *tert*-Butyl (*tert*-butoxycarbonyl)(5-nitro-3-vinylpyridin-2-yl)carbamate (4.50 kg, crude) was obtained as a yellow solid and was used to next step reaction and without purification. ^1^H NMR 400 MHz (MeOD-*d*_4_) δ 9.21 (d, *J* = 2.64 Hz, 1H), 8.86 (d, *J* = 2.51 Hz, 1H), 6.75 (dd, *J* = 17.50, 11.11 Hz, 1H), 6.14 (d, *J* = 17.44 Hz, 1H), 5.73 (d, *J* = 11.04 Hz, 1H), 1.38 (s, 18H). Pd/C (450 g, 10%) and Pd(OH)_2_ (432 g, 20%) were stirred in MeOH (45.0 L). *tert-*Butyl (*tert*-butoxycarbonyl)(5-nitro-3-vinylpyridin-2-yl)carbamate (4.50 kg, 13.3 mol) was added and the resulting mixture was stirred under H_2_ (50 psi) at 50 °C for 24 h. The reaction was filtered and the filtrate was concentrated to give *tert*-butyl (5-amino-3-ethylpyridin-2-yl)(*tert*-butoxycarbonyl)carbamate (**87**) (3.80 kg, 91% yield) as a yellow solid which was used in the next step without purification. ^1^H NMR (400 MHz MeOD-*d*_4_) δ 7.67 (d, *J* = 2.75 Hz, 1H), 7.06 (d, *J* = 2.63 Hz, 1H), 2.44 (q, *J* = 7.55 Hz, 2H), 1.38 (s, 19H), 1.21 (t, *J* = 7.63 Hz, 3H).

#### 2-((6-(Bis(*tert*-butoxycarbonyl)amino)-5-ethylpyridin-3-yl)amino)-2-oxoacetic Acid (**88**)

**87** (1.90 kg, 5.63 mol) was stirred in ACN (13.3 L) at 0 °C under N_2_ atmosphere. DIEA (1.09 kg, 8.45 mol, 1.47 L) was added dropwise followed by ethyl 2-chloro-2-oxo-acetate (922 g, 6.76 mol, 756 mL) dropwise. The reaction mixture was stirred for 24 h at 25 °C after which it was poured into water (12.0 L) and separated. The aqueous layer was extracted with EtOAc (5.00 L × 2) and the organic layer was dried with Na_2_SO_4_ and concentrated. The residue was purified by column chromatography (SiO_2_, petroleum ether/ethyl acetate = 100/1 to 1/1) to give ethyl 2-((6-(bis(*tert*-butoxycarbonyl)amino)-5-ethylpyridin-3-yl)amino)-2-oxoacetate (3.00 kg, 61% yield) as a white solid. ^1^H NMR (400 MHz CDCl_3_) δ 9.02 (s, 1H), 8.49 (d, *J* = 2.63 Hz, 1H), 8.21 (d, *J* = 2.50 Hz, 1H), 4.43 (q, *J* = 7.13 Hz, 2H), 2.58 (q, *J* = 7.50 Hz, 2H), 1.41–1.45 (m, 3H), 1.39 (s, 18H), 1.24 (t, *J* = 7.57 Hz, 3H). Ethyl 2-((6-(bis(*tert*-butoxycarbonyl)amino)-5-ethylpyridin-3-yl)amino)-2-oxoacetate (3.00 kg, 6.86 mol) was stirred in THF (6.00 L) and MeOH (6.00 L) at 0 °C. LiOH·H_2_O (575 g, 13.7 mol) in H_2_O (6.00 mL) was added dropwise at 0 °C and the mixture was stirred for 3 h after which the mixture was acidified to pH = 3 with a solution of 1 M HCl. The precipitate that formed was filtered and dried to give 2-((6-(bis(*tert*-butoxycarbonyl)amino)-5-ethylpyridin-3-yl)amino)-2-oxoacetic acid (**88**) (1.80 kg, 68%) as a white solid which was used to next step reaction and without purification. ^1^H NMR (400 MHz MeOD-*d*_4_) δ 8.71 (d, *J* = 2.50 Hz, 1H), 8.26 (d, *J* = 2.50 Hz, 1H), 2.59 (q, *J* = 7.59 Hz, 2H), 1.39 (s, 18H), 1.27 (t, *J* = 7.57 Hz, 3H).

#### *tert*-Butyl 4-(5-Chlorobenzo[*d*]thiazol-2-yl)piperidine-1-carboxylate (**89**)

2-Amino-4-chlorobenzenethiol (100 g, 626 mmol, 1.00 equiv) was stirred in ethyl acetate (700 mL). *tert*-butyl 4-formylpiperidine-1-carboxylate (160 g, 751 mmol, 1.20 equiv) was added and the reaction mixture was irradiated with a 100 W blue LED bulb at 25 °C for 3 h. The white solid that formed was filtered and dissolved in THF (1500 mL) in a sealed tube. PCC (189 g, 877 mmol, 1.10 equiv) and dioxosilane (191 g, 3.19 mol, 4.00 equiv) were added under N_2_ at 25 °C, then DCM (1500 mL) was added, and the mixture was stirred for 5 h at 20–25 °C. The reaction mixture was filtered, and the filtrate was concentrated. The residue was purified by column chromatography (SiO_2_, petroleum ether/ethyl acetate = 100/1 to 2/1) to afford *tert*-butyl 4-(5-chlorobenzo[*d*]thiazol-2-yl)piperidine-1-carboxylate (**89**) (550 g, 65% yield) as a white solid. ^1^H NMR (400 MHz, CDCl_3_) δ 8.12 (d, *J* = 8.53 Hz, 1H), 8.04 (d, *J* = 2.01 Hz, 1H), 7.47 (dd, *J* = 8.53, 2.01 Hz, 1H), 4.03 (br d, *J* = 12.30 Hz, 2H), 3.34–3.40 (m, 1H), 2.93 (br s, 2H), 2.09 (br d, *J* = 10.67 Hz, 2H), 1.58–1.70 (m, 1H), 1.54–1.71 (m, 1H), 1.32–1.45 (m, 9H).

#### 5-Chloro-2-(1-methylpiperidin-4-yl)benzo[*d*]thiazole (**90**)

**89** (500 g, 1.42 mol, 1.00 equiv) was stirred with formic acid (1.00 L) and formaldehyde (575 g, 7.08 mol, 527 mL, 37.0% by weight, 5.00 equiv) under N_2_ at 25 °C, then stirred for 3 h at 90–95 °C. HCl in ethyl acetate (4 M, 3000 mL) was added. The resultant white solid was filtered and dried at 45 °C to give 5-chloro-2-(1-methylpiperidin-4-yl)benzo[*d*]thiazole (**90**) (395 g, 1.12 mol, 60% yield, HCl) as a white solid. ^1^H NMR (400 MHz CDCl_3_) δ 7.95 (d, *J* = 1.97 Hz, 1H), 7.74–7.78 (m, 1H), 7.30–7.36 (m, 1H), 3.09 (tt, *J* = 11.51, 3.84 Hz, 1H), 2.98 (br d, *J* = 11.84 Hz, 2H), 2.34 (s, 3H), 2.08–2.22 (m, 4H), 1.93–2.03 (m, 2H).

#### *tert*-Butyl (*S*)-3-Methyl-6-(2-(1-methylpiperidin-4-yl)benzo[*d*]thiazol-5-yl)-3,4-dihydropyridine-1(2*H*)-carboxylate (**91**)

**90** (200 g, 659 mmol, 1.00 equiv, HCl) was stirred with X-phos G2 Pd precatalyst (18.2 g, 23.1 mmol, 0.035 equiv) and (Bpin)_2_ (251 g, 989 mmol, 1.50 equiv) in dioxane (3.0 L). The reaction mixture was purged with N_2_ three times. KOAc (259 g, 2.64 mol, 4.00 equiv) was added and the reaction mixture was stirred for 10 h at 105–110 °C. The reaction mixture was filtered, and the filtrate was concentrated and the resultant residue was purified by column chromatography (SiO_2_, petroleum ether/ethyl acetate = 10/1 to 0/1) to afford (*S*)-5-(5-methyl-3,4,5,6-tetrahydropyridin-2-yl)-2-(1-methylpiperidin-4-yl)benzo[*d*]thiazole (93 g, 259 mmol, 39% yield) as a yellow solid which was then stirred with **68** in dioxane (1.00 L). H_2_O (500 mL) and K_2_CO_3_ (107 g, 778 mmol, 3.00 equiv) were added and the suspension was degassed under vacuum and purged with N_2_ three times. Pd(dppf)Cl_2_ (9.50 g, 12.9 mmol, 0.05 equiv) was added and the suspension was degassed under vacuum and purged with N_2_ three times. The reaction was stirred for 12 h at 90 °C. The reaction mixture was then cooled to 20 °C and filtered and the solid was washed with ethyl acetate (400 mL). The combined organics were concentrated, and the residue was dissolved in ethyl acetate (400 mL) and washed with H_2_O (200 mL). The aqueous layer was extracted with ethyl acetate (100 mL) and the combined organic layer was concentrated. The residue was purified by column chromatography (SiO_2_, petroleum ether/ethyl acetate = 40/1 to 8/1) to give *tert*-butyl (*S*)-3-methyl-6-(2-(1-methylpiperidin-4-yl)benzo[*d*]thiazol-5-yl)-3,4-dihydropyridine-1(2*H*)-carboxylate (**91**) (66.2 g, 155 mmol, 60% yield) as a brown oil. ^1^HNMR (400 MHz CDCl_3_) δ 7.91 (s, 1H),7.75 (d, *J* = 8.33 Hz, 1H), 7.31 (d, *J* = 8.11 Hz, 1H), 5.39 (br s, 1H), 4.11 (br d, *J* = 8.33 Hz, 1H), 3.13 (br d, *J* = 9.21 Hz, 1H), 2.96–3.08 (m, 3H), 2.40 (s, 3H), 2.24 (br d, *J* = 11.18 Hz, 3H), 1.96–2.09 (m, 3H), 1.88 (ddd, *J* = 18.74, 8.66, 3.73 Hz, 1H), 1.53 (s, 1H), 1.25 (s, 4H), 1.O04 (br d, *J* = 6.80 Hz, 12H).

#### 5-((2*R*,5*S*)-5-Methylpiperidin-2-yl)-2-(1-methylpiperidin-4-yl)benzo[*d*]thiazole (**92**)

**91** (66.0 g, 154 mmol, 1.00 equiv) was stirred in MeOH (200 mL) and 4 M HCl/MeOH (660 mL) for 5 h at 20 °C. The solvent was removed under reduce pressure and the residue was dissolved in H_2_O (300 mL) and saturated Na_2_CO_3_ was added to bring the solution to pH = 8–9. The aqueous layer was extracted with ethyl acetate (200 mL × 3) and the combined organic layer was concentrated to afford (*S*)-5-(5-methyl-3,4,5,6-tetrahydropyridin-2-yl)-2-(1-methylpiperidin-4-yl)benzo[*d*]thiazole (44.3 g, 135 mmol, 88% yield) as a brown solid which was used in the next step without purification. ^1^H NMR (400 MHz CDCl_3_) δ 8.27 (s, 1H), 7.96 (br d, *J* = 8.50 Hz, 1H), 7.83 (dd, *J* = 8.51, 1.25 Hz, 1H), 4.04 (br dd, *J* = 17.57, 2.19 Hz, 1H), 3.22–3.36 (m, 1H), 3.10 (br t, *J* = 10.76 Hz, 1H), 2.99 (br d, *J* = 10.63 Hz, 2H), 2.87 (br dd, *J* = 18.07, 3.69 Hz, 1H), 2.59–2.74 (m, 1H), 2.34 (d, *J* = 1.38 Hz, 3H), 2.07–2.26 (m, 5H), 1.89–2.06 (m, 4H), 1.75 (br d, *J* = 3.38 Hz, 1H), 1.34–1.51 (m, 1H), 1.22–1.30 (m, 1H), 0.93–1.08 (m, 3H). This material was dissolved in MeOH (250 mL) and NaBH_4_ (5.63 g, 149 mmol, 1.10 equiv) was added at 0 °C. The reaction was stirred at 0 °C for 1 h, after which the reaction mixture was quenched with H_2_O (200 mL) and the organic solvent was removed under reduce pressure. The aqueous layer was extracted with DCM (200 mL × 3) and the combined organic layer was concentrated to afford 5-((2*R*,5*S*)-5-methylpiperidin-2-yl)-2-(1-methylpiperidin-4-yl)benzo[*d*]thiazole (**92**) (42.7 g, 129 mmol, 96% yield) as a brown solid which was used in the next step without purification. ^1^H NMR (400 MHz CDCl_3_) δ 7.92–8.02 (m, 1 H), 7.95 (s, 1H), 7.78 (br d, *J* = 7.38 Hz, 1H), 7.43 (br d, *J* = 8.25 Hz, 1H), 3.69 (br d, *J* = 11.13 Hz, 1H), 3.17 (br d, *J* = 11.26 Hz, 1H), 3.09 (br t, *J* = 11.01 Hz, 1H), 2.98 (br d, *J* = 10.63 Hz, 2H), 2.46 (br t, *J* = 11.07 Hz, 1H), 2.34 (s, 3H), 2.07–2.23 (m, 4H), 1.94–2.06 (m, 2H), 1.88 (br t, *J* = 13.70 Hz, 3H), 1.54–1.76 (m, 2H), 1.11–1.29 (m, 1H), 0.91 (br d, *J* = 5.63 Hz, 3H).

#### *N*-(6-Amino-5-ethylpyridin-3-yl)-2-((2*R*,5*S*)-5-methyl-2-(2-(1-methylpiperidin-4-yl)benzo[*d*]thiazol-5-yl)piperidin-1-yl)-2-oxoacetamide (**TNG462**)

**88** (53.0 g, 129 mmol, 1.00 equiv) and **92** (42.7 g, 129 mmol, 1.00 equiv) were stirred in DMF(250 mL). TEA (39.3 g, 388 mmol, 54.1 mL, 3.00 equiv) was added at 0 °C, followed by HATU (49.3 g, 129 mmol, 1.00 equiv). The reaction was stirred for 6 h at 25 °C. The reaction was then poured into H_2_O (500 mL) and extracted with ethyl acetate (500 mL × 3). The combined organic layer was washed with brine (200 mL × 2) and the organic layer was concentrated. The residue was purified by column chromatography (SiO_2_, petroleum ether/ethyl acetate = 1/1 to 0/1) to afford *tert*-butyl (*tert*-butoxycarbonyl)(3-ethyl-5-(2-((2*R*,5*S*)-5-methyl-2-(2-(1-methylpiperidin-4-yl)benzo[*d*]thiazol-5-yl)piperidin-1-yl)-2-oxoacetamido)pyridin-2-yl)carbamate (60.0 g, 83.2 mmol, 64% yield) as a brown solid. This was then stirred in MeOH (60.0 mL) and 4 M HCl/MeOH (600 mL) was added, and the reaction was stirred at 20–25 °C for 5 h. The solvent was then removed under reduced pressure and the residue was dissolved with H_2_O (300 mL) and saturated Na_2_CO_3_ was added to bring the solution to pH = 8–9. The aqueous layer was then extracted with ethyl acetate (200 mL × 3) and the combined organic layer was concentrated. The crude product was purified by reverse-phase HPLC (Waters SFC350 preparative SFC; DAICEL CHIRALPAK IC column (250 mm × 50 mm, 10 μm); mobile phase A = CO_2_, mobile phase B = MeOH (0.1% NH_3_·H_2_O)/ACN, 1:1 v/v; gradient: %B = 60% isocratic elution mode; flow rate = 200 g/min; wavelength = 220 nm; column temperature = 40 °C; system back pressure = 100 bar) to afford **TNG462** (13.4 g, 25.1 mmol, 30% yield, 97.6% purity) as a white solid. ^1^H NMR (400 MHz CDCl_3_) δ 9.23–9.37 (m, 1H), 8.03–8.17 (m, 1H), 7.92 (br s, 1H), 7.83 (br d, *J* = 7.94 Hz, 1H), 7.71 (br s, 1H), 7.28–7.38 (m, 1H), 6.56 (br s, 1H), 5.92 (br s, 1H), 4.77 (br d, *J* = 13.23 Hz, 1H), 4.47 (br d, *J* = 11.47 Hz, 2H), 4.19–4.30 (m, 1H), 3.39 (br d, *J* = 12.57 Hz, 1H), 2.90–3.14 (m, 3H), 2.13–2.53 (m, 14H), 1.84–2.06 (m, 5 H), 1.42 (br s, 1H), 1.17–1.32 (m, 3H), 1.05–1.14 (m, 3H). Melting point of crystalline material = 145.8 °C. [α]_D_^21^ +158.68 (*c* = 0.121 g/100 mL, EtOH). LCMS (ESI): [M + H]^+^*m*/*z* calcd 521.27; found 521.2. *t*_R_ = 1.784 min. HRMS (ESI, + vw ion): *m*/*z* calcd for C_28_H_37_N_6_O_2_S [M + H ] 521.2693; found 521.2697.

### HAP1 MTAP WT and MTAP-Null In-Cell Western Assay

Detailed methods can be found in Cottrell et al., 2024.^4^ In brief, the HAP1 *MTAP*-isogenic cell line pair was acquired from Horizon Discovery (HZGHC004894c005) and maintained in DMEM (high glucose) + 10% FBS in a humidified, 10% CO_2_ tissue culture incubator. The SAM-cooperative PRMT5 inhibitor, GSK3326595, was sourced from Selleck Chemicals and maintained as a 10 mM DMSO stock. HAP1 MTAP WT and MTAP-null cells were treated with compounds for 24 h in 384-well microtiter plates, and then normalized SDMA levels were determined using a multi-mAb SDMA antibody (Cell Signaling 13222) and DRAQ5 (LiCor 926-32211 and VWR 10761-508). Background signal was determined by signal from wells treated with 1 μM GSK3326595. Data analysis was performed using the 4-parameter logistic (4-PL) Hill equation with maximal effect constrained to 0. The fit was performed using GraphPad Prism or the default IC_50_ fitting procedure in Dotmatics Studies 5.4 as part of a customized data analysis protocol.

### Cell Line Viability Assays

Detailed methods can be found in Cottrell et al., 2024.^4^ In brief, the HAP1 and HCT116 *MTAP*-isogenic cell line pairs were acquired from Horizon Discovery (HZGHC004894c005 and HD R02-033, respectively), the LU99 and LN18 MTAP-isogenic cell line pairs were engineered by stable introduction of a full-length MTAP cDNA under the control of a UbiC promoter. All cell lines were maintained in DMEM (high glucose) + 10% FBS in a humidified, 10% CO_2_ tissue culture incubator and confirmed for their MTAP status by immunoblot. Cell viability was determined by CellTiter-Glo following 7-days of compound treatment. Data are plotted as % of the DMSO control wells and fit using a four-parameter logistic (4-PL) Hill equation with maximal effect or baseline constrained to 0. The fit was performed using GraphPad Prism or the default IC_50_ fitting procedure in Dotmatics Studies 5.4 as part of a customized data analysis protocol. Absolute IC_50_ values are reported for each cell line.

For the 179-cancer cell line panel, potency is reported as a relative IC_50_ as determined by a 4-PL Hill equation (GraphPad Prism), and selectivity was visualized by plotting the maximum effect (Amax) of TNG462 at 40 nM according to the curve fit.

### Cellular PRMT5 Thermostability Assay

The LN18 MTAP-null cell line was either treated with TNG462 for 1 h and immediately assayed, treated for 1 h and assayed 72 h following compound washout and culture in compound-free media, or following 72 h compound treatment. Treated cells were collected by trypsinization, washed with 1× PBS, and resuspended in a buffer containing 30 mM bicine, 0.003% Tween-20, and 150 mM NaCl. Cells were lysed by 3 freeze/thaw cycles using liquid N_2_ and a room temperature water bath. Supernatant was heated to 57 °C for 3 min and then centrifuged to removed insoluble protein. Immunoblots were run to detect PRMT5 (CST #2252) and densitometry was performed by the Licor analysis tool kit.

### *In Vivo* Pharmacology

All protocols for *in vivo* pharmacology studies were approved by the relevant Institutional Animal Care and Use Committees (Pharmaron, Beijing, China; CrownBio, San Diego, CA, and Taicang and Beijing, China; Champions Oncology, Rockville, MD; XenoSTART, San Antonio, TX; Charles River, Freiburg, Germany) following the guidance of the Association of Assessment and Accreditation of Laboratory Animal Care.

Following acclimatization, LU99 or OCI-LY19 cancer cells were injected subcutaneously into the right flank of 6- to 8-week-old female BALB/c nude mice and allowed to form palpable tumors. Mice were randomized to treatment groups with a mean tumor volume of approximately 200 mm^3^ (LU99 efficacy), 300 mm^3^ (LU99 PK/PD) or 125 mm^3^ (OCI-LY19) in size. **TNG462** or TNG908 were formulated in 5% DMA/20% Captisol in water (LU99) or acidified water (pH 4–6; OCI-LY19). TNG908 was formulated in 5% DMA/20% Captisol. PDX studies were conducted with similar study designs.

Tumor lengths and widths were measured using calipers, and tumor volumes (TVs) were calculated as (length × width × width)/2. For data analysis, tumor growth inhibition (%TGI) was calculated as %TGI = [1 – (Treated TV_final_ – Treated TV_initial_)/(Vehicle TV_final_ – Vehicle TV_initial_)] × 100, and tumor regression (%TV) was calculated as %TV = [mean TV_final_ – mean TV_initial_] × 100. Tumor volume data were analyzed using GraphPad Prism software.

### Western Blotting

Protein lysates were generated by lysis of frozen tumor tissue using RIPA buffer. Samples were normalized by protein concentration using Pierce Rapid Gold BCA Protein Assay Kit (A53225). SDS-PAGE was run using Invitrogen NuPAGE 4–12% Bis-Tris Midi Protein Gels (WG1402BOX). Antibodies SDMA (CST#13222), ACTB (CST#3700) were used at 1:1000 dilution.

## Supporting Information Available

General experimental and chemical procedures; NMR spectra and HPLC/LCMS traces of final compounds; analytical data for TNG462; small-molecule crystal structure for TNG462; biochemical recovery assay; methyltransferase panel for TNG462; Eurofins SAFETYscan panel for TNG462; MDR1-MDCKII assay; human liver microsomes assay; hepatocyte stability assay; kinetic solubility assay; hERG assay; *in vivo* pharmacokinetics; plasma protein binding assay; plasma stability assay; in cell western assay; cellular viability assay; cellular thermostability assay; *in vivo* pharmacology; Western blotting; PRMT5:MEP50 expression and purification; PRMT5:MEP50 crystallography (PDF)
Molecular formula strings (CSV)

## Abbreviations

BBB: blood–brain barrier
CDX: cell-line-derived xenograft
DMPK: drug metabolism and pharmacokinetics
GI_50_: growth inhibition 50%
HTS: high-throughput screening
MTAP: methylthioadenosine phosphorylase
MTA: methylthioadenosine
PDX: patient-derived xenograft
PRMT5: protein arginine methyltransferase 5
SAM: *S*-adenosylmethionine
