# Supplementary material for: Discovery of TNG462: A Highly Potent and Selective MTA-Cooperative PRMT5 Inhibitor to Target Cancers with MTAP Deletion
Source: J Med Chem. 2025 Mar 4;68(5):5097–119. doi: 10.1021/acs.jmedchem.4c03067 (PMC11912494; doi:10.1021/acs.jmedchem.4c03067)
Supplement: Supplementary file 1 — jm4c03067_si_001.pdf [file jm4c03067_si_001.pdf]

SUPPORTING INFORMATION

DISCOVERY OF TNG462: A HIGHLY POTENT AND SELECTIVE MTA-COOPERATIVE PRMT5 INHIBITOR TO TARGET CANCERS WITH *MTAP* DELETION

Kevin M. Cottrell*‡, Kimberly J. Briggs‡, Alice Tsai‡, Matthew R. Tonini‡, Douglas A. Whittington‡, Shanzhong Gong‡, Colin Liang‡, Patrick McCarren‡, Minjie Zhang‡, Wenhai Zhang‡, Alan Huang‡, and John P. Maxwell‡.

‡Tango Therapeutics, Boston, MA 02215, United States
*Corresponding Author kcottrell@tangotx.com

Table of Contents

General experimental and chemical procedures…………………………………………………………S1
NMR Spectra and HPLC/LCMS traces of final compounds……………………………………………………S69
Analytical data for TNG462……………………………………………………………………S171
Small molecule crystal structure of TNG462…………………………………………………… S181
Biochemical recovery assay……………………………………………………………………S189
Methyltransferase panel for TNG462……………………………………………………………S189
Eurofins SAFETYscan panel for TNG462…………………………………………………………S190
MDR1-MDCKII assay……………………………………………………………………………S193
Human liver microsomes assay…………………………………………………………………S193
Hepatocyte stability assay………………………………………………………………………S193
Kinetic solubility assay…………………………………………………………………………S193
hERG assay……………………………………………………………………………………S193
In vivo PK……………………………………………………………………………………S194
Plasma protein binding assay……………………………………………………………………S194
Plasma stability assay……………………………………………………………………………S194
In cell western assay……………………………………………………………………………S195
Cellular viability assay…………………………………………………………………………S195
Cellular thermostability assay……………………………………………………………………S195
In vivo pharmacology……………………………………………………………………………S195
Western blotting………………………………………………………………………………S195
PRMT5:MEP50 expression and purification………………………………………………………S195
PRMT5:MEP50 crystallography…………………………………………………………………S195

GENERAL EXPERIMENTAL AND CHEMICAL PROCEDURES

All chemicals were provided by Enamine Ltd., WuXi Apptech, or other commercial suppliers and used as received unless otherwise indicated. All solvents were treated according to standard methods. All reactions were monitored and analysis of final compounds performed by LC-MS using Agilent 1260 LC/MSD instruments, with an Agilent Poroshell 120 SB-C18 4.6 x 30mm 2.7 µm column, column Temperature: 60 °C, mobile phase: A – H_2O (0.1% formic acid), B – ACN (0.1% formic acid), flow rate: 1.5 mL/min, gradient: 0.01 min – 1% B, 5.00 min – 100% B, 5.99 min – 100% B, MS Ionization mode: Electrospray ionization (ESI), MS Scan range: 83 – 1000 m/z, UV detection: 215 nm, 254 nm, 280 nm unless otherwise specified. Thin-layer chromatography (TLC) with pre-coated silica gel GF254 (0.2 mm) was used and the results were visualized using either UV light or $KMnO_4$ stain. Proton nuclear magnetic resonance (^{1}H-NMR) spectra were recorded at 400, 500 or 600 MHz on Varian or Bruker instrumentation; chemical shifts were calibrated using residual non-deuterated solvents $CHCl_3$ (δ = 7.26 ppm), DMSO (δ = 2.50 ppm) or MeOH (δ = 3.31 ppm) and expressed in *δ* ppm. Coupling constants (*J*), when given, are reported in hertz. Multiplicities are reported using the following abbreviations: s = singlet, d = doublet, dd = doublet of doublets, t = triplet, q = multiplet (range of multiplets is given), br = broad signal, dt = doublet of triplets. ^{19}F NMR spectra were recorded at 376 MHz (Varian), ^{13}C NMR spectra were recorded at 101, 126 or 151 MHz (Varian). ^{13}C NMR chemical shifts for ^{13}C NMR are reported relative to the central $CHCl_3$ (δ = 77.16 ppm), DMSO (δ = 39.52 ppm) or MeOH (δ = 49.00 ppm) and chemical shifts are reported in parts per million (ppm). All final compounds were purified by reverse phase high-performance liquid chromatography (HPLC) or supercritical fluid chromatography (SFC) or silica gel chromatography (100-200 mesh). HPLC was done with an Agilent 1260 HPLC instrument (Agilent Technologies, Germany) equipped with a G7161A Preparative Binary Pump, a G7157A Prep Autosampler, a G7115A DAD WR and a G7159B Preparative Fraction Collector. The Open Lab CDS software (version C.01.10 was used for instrument control, data acquisition and data handling). SFC was done with a Waters 100q Prep SFC System. Chiral HPLC analytical analysis was done with an Agilent 1200 HPLC instrument (Agilent Technologies, Germany) equipped with a G1379B degasser, a G1312A Binary Pump, a G1329A ALS autosampler, a G1315A Diode Array Detector. Chiral SFC analytical analysis was done with an Agilent 1260 SFC instrument (Agilent Technologies, Germany) equipped with a G1379B degasser, a G1312B Binary Pump, a G1313A ALS autosampler, a G1316A thermostatted column compartment, a G1315D Diode Array Detector and an Aurora SFC systems. Melting points were taken using OptiMelt Automated Melting Point System Digital Image Processing Technology SRS Stanford Pesearch Systems, 2 °C/min (5 °C/min at high melting point). Optical rotation was measured with Polarimeter Anton Paar GmbH MCP 300 (Accuracy: ±0.003°) used to measure the angle of optical rotation. Standard conditions for analysis: solution concentration 0.5 g/100 mL (solvent: MeOH), wavelength 589 nm, temperature 21 °C. All oxamides exist as rotamers in ^{1}H NMR spectra. All compounds are > 95% pure by HPLC.

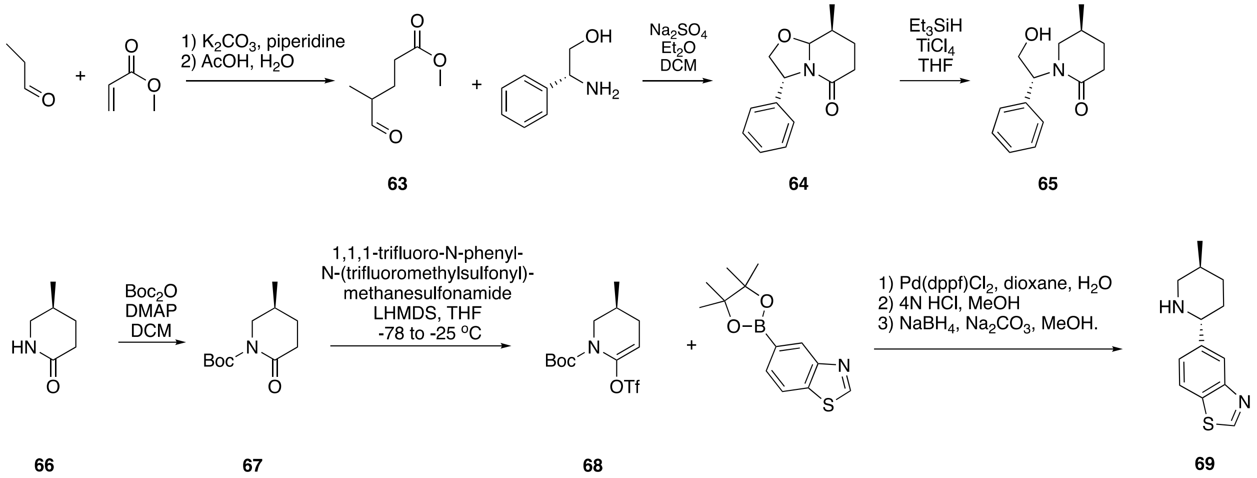

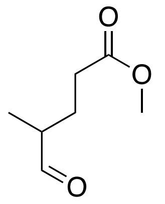

methyl 4-methyl-5-oxopentanoate (**63**)
To a stirring mixture of piperidine, 99% (293.22 g, 3.44 mol, 340.16 mL) and potassium carbonate - granular (95.19 g, 688.72 mmol), propanal (100 g, 1.72 mol, 123.46 mL) was added dropwise. The reaction mixture was stirred at room temperature for 18 h. After 18 h, the reaction mixture was filtered through a pad of Na_2SO_4 and washed with MTBE (1000 mL). The filtrate was dried over Na_2SO_4, filtered, and concentrated under reduced pressure. The obtained crude enamine was dissolved in MeCN (1000 mL) and methyl prop-2-enoate (296.46 g, 3.44 mol, 310.10 mL) was added dropwise to the solution at room temperature. The resulting reaction mixture was refluxed for 16 h. After 16 h, acetic acid (206.79 g, 3.44 mol, 196.94 mL) was added followed by water (1000 mL) and the resulting solution was heated at reflux for an additional 16 h after which the mixture was cooled to room temperature, saturated with NaCl, and extracted with MTBE (2 x 1000 mL). The combined organic phase was dried over Na_2SO_4, filtered, and concentrated under reduced pressure to obtain crude methyl 4-methyl-5-oxopentanoate, **63** (264 g, crude). The crude product was used for the next step without any further purification. ^{1}H NMR (500 MHz, $CDCl_3$) δ 1.09 (d, 3H), 1.60 – 1.72 (m, 1H), 1.94 – 2.00 (m, 1H), 2.29 – 2.42 (m, 3H), 3.64 (s, 3H), 9.59 (s, 1H).

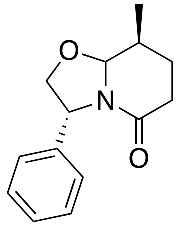

(3*R*,8*S*,8a*R*)-8-methyl-3-phenyltetrahydro-2*H*-oxazolo[3,2-a]pyridin-5(3*H*)-one (**64**).
A mixture of **63** (67.8 g, 470.28 mmol), (2*R*)-2-amino-2-phenyl-ethanol (64.51 g, 470.28 mmol) and sodium sulfate, anhydrous (66.80 g, 470.28 mmol) in DCM (800 mL) and diethyl ether (200 mL) was stirred at 0 °C for 5 h. After 5 h, the resulting suspension was filtered, and the filtrate was concentrated under reduced pressure. The residue was purified by column chromatography (Interchim; 800 g SiO_2; chloroform/acetonitrile with acetonitrile from 0 - 15%, flow rate = 120 mL/min, Rv=4-10 cv.) to give product (3*R*,8*S*,8a*R*)-8-methyl-3-phenyltetrahydro-2*H*-oxazolo[3,2-a]pyridin-5(3*H*)-one, **64** (59.8 g, 258.55 mmol, 55% yield) as a light-yellow gum. ^{1}H NMR (500 MHz, $CDCl_3$) δ 1.20 (d, *J* = 6.3 Hz, 3H), 1.42 - 1.56 (m, 1H), 1.86 – 2.00 (m, 2H), 2.22 - 2.44 (m, 2H), 4.00 (dd, *J* = 8.8, 1.2 Hz, 1H), 4.07 – 4.17 (m, 1H), 4.42 (dd, *J* = 8.9, 3.2 Hz, 1H), 4.87 – 4.95 (m, 1H), 7.17 - 7.32 (m, 5H). LCMS (ESI): [M+H]+ m/z: calcd 231.1; found 232.2; Rt = 1.12 min.

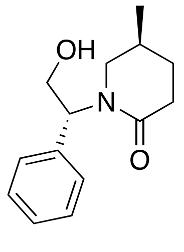

(*S*)-1-((*R*)-2-hydroxy-1-phenylethyl)-5-methylpiperidin-2-one (**65**).

Triethylsilane (1.51 g, 12.97 mmol, 2.07 mL) and titanium tetrachloride (3.69 g, 19.46 mmol) were added to a solution of **64** (1 g, 4.32 mmol) in anhydrous DCM (25 mL) and the mixture was stirred at 40 °C for 24 h. Additional titanium tetrachloride (3.69 g, 19.46 mmol, 764.36 μL) and triethylsilane (1.51 g, 12.97 mmol, 2.07 mL) were added, and the stirring was continued at 50 °C for 24 h. The mixture was poured into saturated aqueous $NaHCO_3$ (100 mL) solution. The aqueous phase was filtered over Celite and extracted with CH_2Cl_2. The combined organic extracts were dried over Na_2SO_4, filtered, and concentrated under reduced pressure to give a residue, which was purified by column chromatography (Companion Combiflash; 40 g SiO_2; MTBE/methanol with methanol from 0 - 8%, flow rate = 40 mL/min, Rv = 9 - 11 cv.) to give product (*S*)-1-((*R*)-2-hydroxy-1-phenylethyl)-5-methylpiperidin-2-one, **65** (0.51 g, 2.19 mmol, 51% yield) as a colorless oil. ^{1}H NMR (400 MHz, $CDCl_3$) δ 0.93 (d, *J* = 6.4 Hz, 3H), 1.44 - 1.54 (m, 1H), 1.77 - 1.86 (m, 2H), 2.40 – 2.62 (m, 2H), 2.83 (t, *J* = 11.0 Hz, 1H), 2.92 – 2.99 (m, 1H), 4.04 – 4.21 (m, 2H), 5.71 – 5.79 (m, 2H), 7.17 – 7.37 (m, 5H). LCMS (ESI): [M+H]+ m/z: calcd 233.1; found 234.2; Rt = 0.98 min.

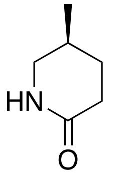

(*S*)-5-methylpiperidin-2-one (**66**).

Ammonia (500 mL) was condensed into a three-necked, 1000 mL, round-bottomed flask equipped with a cold finger condenser at −78 °C. A solution of **65** (17.2 g, 73.72 mmol) in dry THF (100 mL) was added and the temperature was raised to −33 °C. Sodium (5.08 g, 221.17 mmol) was added in small portions until a blue color persisted, and the mixture was stirred at −33 °C for 3 minutes. Th e reaction was quenched by the addition of solid NH_4Cl until the blue color disappeared, and then the mixture was stirred at room temperature for 5 h. CH_2Cl_2 was added, the solid was filtered, and the solvent was removed under reduced pressure to give (*S*)-5-methylpiperidin-2-one, **66** (15 g, crude) as a light-yellow oil. The residue was used for the next step without any further purification. ^{1}H NMR (400 MHz, DMSO-*d*$_6$) δ 1.01 (d, *J* = 6.6 Hz, 3H, CH3), 1.45-1.51 (m, 1H, H-4), 1.83-1.99 (m, 2H, H-4, H-5), 2.34 (ddd, *J* = 17.8, 10.8, 6.4 Hz, 1H, H-3), 2.43 (ddd, *J* = 17.8, 6.4, 3.5 Hz, 1H, H-3), 2.92 (t, *J* = 10.8 Hz, 1H, H-6), 3.26-3.33 (m, 1H, H-6), 6.10 (br s, 1H, NH).

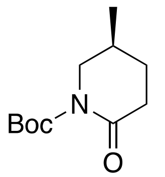

(*S*)-tert-butyl 5-methyl-2-oxopiperidine-1-carboxylate (**67**).

To a solution of **66** (15 g, 79.54 mmol, crude) and DMAP (971.67 mg, 7.95 mmol) in DCM (500 mL) was added di-tert-butyl dicarbonate (17.36 g, 79.54 mmol, 18.25 mL) dropwise at 21 °C. The resulting reaction mixture was stirred for 1 h. After 1 h, the resulting solution was diluted with 10% aq. HCl and brine, dried over Na_2SO_4 and concentrated under reduced pressure to obtain crude product, which was purified by column chromatography (Companion Combiflash, 330g SiO_2, petroleum ether/MTBE with MTBE from 10~25%, flow rate = 100 mL/min, Rv = 6 CV) to give product (*S*)-tert-butyl 5-methyl-2-oxopiperidine-1-carboxylate, **67** (9 g, 42.20 mmol, 53% yield) as a yellow solid. ^{1}H NMR (400 MHz, DMSO-*d*$_6$) δ 0.96 (d, *J* = 6.6 Hz, 3H), 1.42 (s, 9H), 1.74 – 1.86 (m, 1H), 1.87 – 1.97 (m, 1H), 2.30 – 2.39 (m, 2H), 2.98 - 3.11 (m, 1H), 3.12 (s, 1H), 3.60 – 3.68 (m, 1H). LCMS (ESI): $[M+H]^+$ m/z: calcd 213.2; found 158.2; Rt = 1.24 min (*t*-Bu cleavage).

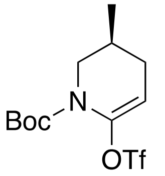

tert-butyl (*S*)-3-methyl-6-(((trifluoromethyl)sulfonyl)oxy)-3,4-dihydropyridine-1(2*H*)-carboxylate (**68**).

LiHMDS (1 M in THF/ethylbenzene, 1.05 L, 1.05 eq) was added dropwise under argon to a cooled -78 °C solution of **67** (180 g, 1.0 eq) in THF (1.80 L). The resulting solution was stirred at -78 °C for 1.5 h followed by the addition of 1,1,1-trifluoro-*N*-phenyl-*N*-(trifluoromethylsulfonyl)methanesulfonamide (377 g, 1.25 eq) in THF (1.2 L). The reaction mixture was stirred at -78 °C for 1 h then allowed to warm slowly to 25 °C. The reaction was quenched with 10% NaOH (2 L) at 10 – 20 °C. The organic layer was separated, the aqueous layer was extracted with ethyl acetate (1 L), the combined organic extracts were concentrated, and the residue was purified by column chromatography (SiO_2, petroleum ether / ethyl acetate; 150/1 to 80/1) to afford *tert*-butyl (*S*)-3-methyl-6-(((trifluoromethyl)sulfonyl)oxy)-3,4-dihydropyridine-1(2*H*)-carboxylate, **68** (272 g, 90% yield, 96.6% purity) as a colorless oil. ^{1}H NMR (400 MHz, $CDCl_3$) δ 5.25 (t, *J* = 3.8 Hz, 1H), 3.87 (dd, *J* = 12.8, 3.3 Hz, 1H), 2.99 (dd, *J* = 12.7, 9.2 Hz, 1H), 2.45 - 2.34 (m, 1H), 1.97 - 1.89 (m, 1H), 1.89 - 1.76 (m, 1H), 1.49 (s, 9H), 0.99 (d, *J* = 6.5 Hz, 3H). LCMS (ESI): [M+H]+ m/z: calcd 345.09; found 290.0 ([P -t-Bu]+); Rt = 1.408 min.

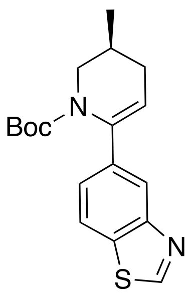

tert-butyl (*S*)-6-(benzo[*d*]thiazol-5-yl)-3-methyl-3,4-dihydropyridine-1(2*H*)-carboxylate.
71 (115, 3333 mmol, 1 eq) and 5-(4,4,5,5-tetramethyl-1,3,2-dioxaborolan-2-yl)benzo[d]thiazole (87.0 g, 333 mmol, 1.00 eq) were stirred in H_2O (575 mL) and dioxane (1.72 L), and Na_2CO_3 (106 g, 999 mmol, 3.00 eq) was added. The suspension was degassed under vacuum and purged with N_2 three times. Pd(dppf)Cl_2 (12.2 g, 16.6 mmol, 0.05 eq) was added and the suspension was degassed under vacuum and purged with N_2 three times. The reaction was stirred for 12 h at 80 °C, then cooled to 20 °C and filtered. The solid was then washed with ethyl acetate (1.5 L) and the combined organics were concentrated. The residue was then dissolved with ethyl acetate (2.00 L) and washed with water (1.50 L). The aqueous was extracted with ethyl acetate (500 mL), and the combined organic layer was concentrated. The residue was purified by column chromatography (SiO_2, petroleum ether/ethyl acetate=40/1 to 8/1) to yield *tert*-butyl (*S*)-6-(benzo[*d*]thiazol-5-yl)-3-methyl-3,4-dihydropyridine-1(2*H*)-carboxylate (63.0 g, 191 mmol, 57% yield) as a yellow solid. ^{1}H NMR (400 MHz, DMSO-d_6) δ 9.36 (s, 1H), 8.08 (d, *J* = 8.38 Hz, 1H), 7.90 (d, *J* = 1.38 Hz, 1H), 7.37-7.40 (m, 1H), 5.46 (t, *J* = 3.56 Hz, 1H), 3.91-3.95 (m, 1H), 3.04-3.10 (m, 1H), 2.37-2.47 (m, 1H), 1.80-1.98 (m, 2H), 0.98 (d, *J* = 6.38 Hz, 3H), 0.94 (s, 9H).

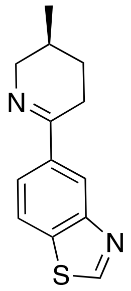

(*S*)-5-(5-methyl-3,4,5,6-tetrahydropyridin-2-yl)benzo[*d*]thiazole.
tert-butyl (*S*)-6-(benzo[d]thiazol-5-yl)-3-methyl-3,4-dihydropyridine-1(2*H*)-carboxylate (63.0 g, 191 mmol, 1.00 eq) was stirred in MeOH (250 mL) and HCl / MeOH (4 M, 477 mL, 10.0 eq) was added. The reaction was stirred for 5 h at 20 °C after which the solvent was removed under reduced pressure. The residue was dissolved with water (500 mL) and saturated Na_2CO_3 was added to pH = 8 ~ 9. The aqueous was extracted with ethyl acetate (800 mL x 3) and the combined organic layer was concentrated to give (*S*)-5-(5-methyl-3,4,5,6-tetrahydropyridin-2-yl)benzo[*d*]thiazole (44.0 g, crude) as a colorless oil which was used without purification. ^{1}H NMR (500 MHz, DMSO-d_6) δ 0.95 (m, 3H), 1.35 (m, 1H), 1.65 (m, 1H), 1.89 (m, 1H), 2.67 (m, 1H), 2.87 (d, 1H), 3.19 (t, 1H), 3.95 (d, 1H), 8.02 (d, 1H), 8.14 (d, 1H), 8.43 (s, 1H), 9.41 (s, 1H).

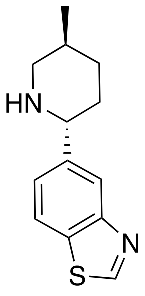

5-((2*R*,5*S*)-5-methylpiperidin-2-yl)benzo[*d*]thiazole (**69**).
(*S*)-5-(5-methyl-3,4,5,6-tetrahydropyridin-2-yl)benzo[d]thiazole (44.0 g, 191 mmol, 1.00 eq) was stirred in MeOH (290 mL) and $NaBH_4$ (11.2 g, 297 mmol, 1.56 eq) was added at 0 °C. The reaction was stirred at 0 °C for 2 h, after which it was quenched with water (500 mL). The solvent was removed under reduce pressure and the aqueous was extracted with DCM (200 mL x 3). The combined organic layer was concentrated to give 5-((2*R*,5*S*)-5-methylpiperidin-2-yl)benzo[*d*]thiazole, **69**, as a yellow oil that was used in the next step reaction without purification. ^{1}H NMR (400 MHz, DMSO-d_6) δ 0.91 (d, 3H), 1.18 (m, 1H), 1.30 – 1.60 (m, 6H), 2.47 (t, 1H), 3.17 (d, 1H), 3.73 (d, 1H), 7.52 (d, 1H), 7.88 (d, 1H), 8.12 (s, 1H), 8.98 (s, 1H). LCMS (ESI): [M+H]+ m/z: calcd 232.1; found 233.0; Rt = 0.691 min.

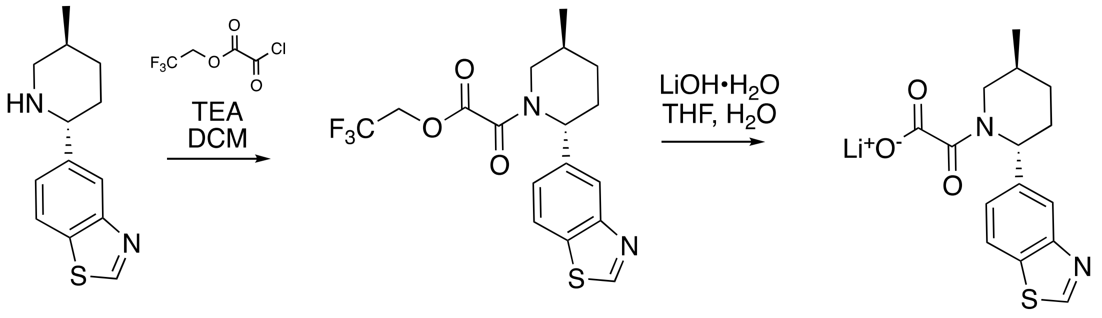

69 **84** **85**

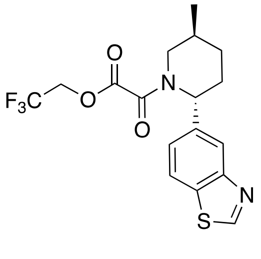

2,2,2-trifluoroethyl 2-((2*R*,5*S*)-2-(benzo[*d*]thiazol-5-yl)-5-methylpiperidin-1-yl)-2-oxoacetate (**84**).
To a stirred solution of **69** (9.70 g, 41.75 mmol) in dichloromethane (1.17 mL) was added triethylamine (6.34 g, 62.62 mmol, 8.73 mL) at room temperature. The resulting reaction mixture was cooled to 0 °C, then 2,2,2-trifluoroethyl 2-chloro-2-oxo-acetate (8.35 g, 43.84 mmol) was added dropwise. The reaction was stirred for 30 minutes at 0 °C then allowed warmed to room temperature and stirred for 16 h. The reaction mixture was concentrated under reduced pressure to obtain 22.2 g of crude **84**, 2,2,2-trifluoroethyl 2-((2*R*,5*S*)-2-(benzo[*d*]thiazol-5-yl)-5-methylpiperidin-1-yl)-2-oxoacetate, as yellow gum, which was used for the next step without purification. LCMS (ESI): [M+H]+ m/z: calcd 386.11; found [387]+; Rt = 3.659.

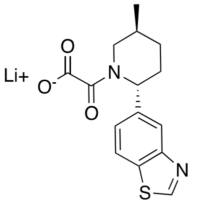

Lithium 2-[(2*R*,5*S*)-2-(1,3-benzothiazol-5-yl)-5-methyl-1-piperidyl]-2-oxo-acetate, (**85**).
To a solution of **84** (5.05 g, 13.07 mmol) in THF (50 mL) was added lithium hydroxide monohydrate, 98% (548.45 mg, 13.07 mmol, 363.21 uL) as a solution in water (10 mL) and the resulting mixture was left to stir at room temperature for 1 h, after which the mixture was evaporated to dryness, dissolved in water, and washed with DCM three times. The aqueous was acidified to pH = 1 and extracted with EtOAc twice. The combined organics were washed with brine, dried over Na_2SO_4, and evaporated to afford lithium 2-[(2*R*,5*S*)-2-(1,3-benzothiazol-5-yl)-5-methyl-1-piperidyl]-2-oxo-acetate, **85** (4.47 g, crude) which was used as a lithium salt in the next steps without further purification. LCMS (ESI): [M+H]+ m/z: calcd 304.36; found [305]+; Rt = 1.347 min.

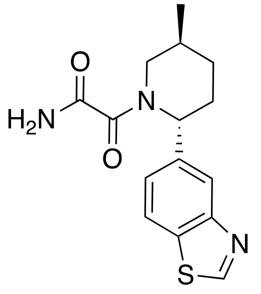

2-((2*R*,5*S*)-2-(benzo[*d*]thiazol-5-yl)-5-methylpiperidin-1-yl)-2-oxoacetamide, **Intermediate 4b**.
150 mL THF was cooled to -20 °C and NH_3 was bubbled into it over 15 minutes, then **84** (3.50 g, 9.06 mmol) was added. The resulting reaction mixture was stirred at -20 °C for 30 minutes, then warmed to room temperature. Upon completion, the reaction mixture was concentrated under reduced pressure to obtain crude product, 2-((2*R*,5*S*)-2-(benzo[*d*]thiazol-5-yl)-5-methylpiperidin-1-yl)-2-oxoacetamide, **Intermediate 4b** (2.7g, 98% yield) as a light-yellow solid. LCMS (ESI): [M+H]+ m/z: calcd 303.12; found [304]+; Rt = 2.728 min.

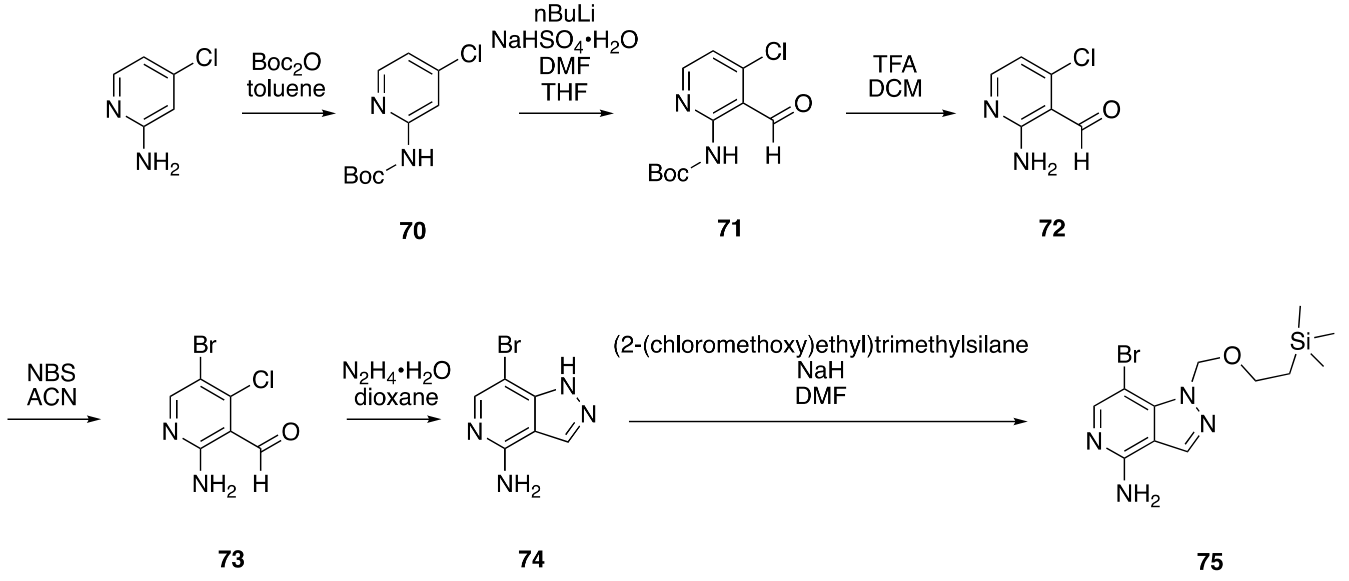

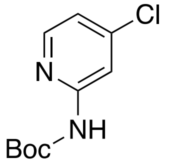

tert-butyl (4-chloropyridin-2-yl)carbamate (**70**).
Di-tert-butyl dicarbonate (221 g, 1.01 mol, 232 mL) was added to the suspension of 4-chloropyridin-2-amine (100 g, 778 mmol) in toluene (1 L). The resulting reaction mixture was stirred at 80 °C for 16 h. The volatiles were then removed under reduced pressure and the residue was taken up in cold hexane (800 mL). The resulting grey precipitate was collected by filtration, and it was stirred with boiling chloroform (1.40 L) for 10 minutes and the resulting slurry was filtered while hot. The filtrate was concentrated under reduced pressure to afford *tert*-butyl (4-chloropyridin-2-yl)carbamate, **70** (139 g, 610 mmol, 78% yield). ^{1}H NMR (400 MHz, $CDCL_3$) δ 9.25 (s, 1H), 8.21 (d, *J* = 5.3 Hz, 1H), 8.1 (s, 1H), 6.96 (dd, *J* = 5.3, 1.3 Hz, 1H), 1.54 (s, 9H).

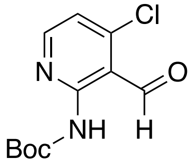

tert-butyl (4-chloro-3-formylpyridin-2-yl)carbamate (**71**).
n-butyllithium (2.5 M in hexanes, 459 mmol, 185 mL) was added dropwise to a solution of **70** (50.0 g, 219 mmol) in tetrahydrofuran (1 L) at -70 °C under argon atmosphere. After the addition was complete, the resulting mixture was stirred at the same temperature for 40 min then, dimethylformamide (48.0 g, 656 mmol, 50.8 mL) was added quickly keeping the temperature below -80 °C. Then cooling bath was then removed and the resulting mixture was slowly warmed to -40 °C. A solution of sodium hydrogen sulfate monohydrate (103 g, 743 mmol) in water (700 mL) was added in one portion and resulting mixture was stirred at ambient temperature for 20 minutes. It was then partitioned between ethyl acetate (1 L) and water (800 mL). The organic layer was washed with brine (2 × 500 mL), dried over anhydrous sodium sulfate and concentrated under reduced pressure to afford **71**, *tert*-butyl (4-chloro-3-formylpyridin-2-yl)carbamate (50.3 g, 196 mmol, 90% yield). ^{1}H NMR (400 MHz, $CDCL_3$) δ 10.72 (br s, 1H), 10.53 (s, 1H), 8.51 (d, 1H), 7.05 (d, 1H), 1.54 (s, 9H).

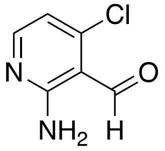

2-amino-4-chloronicotinaldehyde (**72**).
Trifluoroacetic acid (112 g, 980 mmol, 75.5 mL) was added dropwise to the solution of **71** (50.3 g, 196 mmol) in dichloromethane (300 mL). The resulting mixture was stirred at 25 °C for 16 h. The volatiles were removed under reduced pressure and the residue was partitioned between DCM (500 mL) and 10% aq. K_2CO_3 solution (400 mL). The organic layer was separated, dried over anhydrous sodium sulfate, and concentrated in vacuo to afford **72**, 2-amino-4-chloronicotinaldehyde (30.0 g, 192 mmol, 98% yield). ^{1}H NMR (500 MHz, $CDCl_3$) δ 10.43 (s, 1H), 8.87 - 8.16 (br s, 1H), 8.09 (d, *J* = 5.3 Hz, 1H), 6.65 (d, *J* = 4.9 Hz, 1H), 6.41 - 5.49 (br s, 1H).

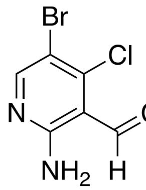

2-amino-5-bromo-4-chloronicotinaldehyde (**73**).
N-bromosuccinimide (39.2 g, 220 mmol, 18.7 mL) was added to a solution of **72** (30.0 g, 192 mmol) in acetonitrile (500 mL). The resulting mixture was stirred at 70 °C for 20 h. The solvent was then removed under reduced pressure and the residue was diluted with cold water (200 mL). The resulting yellow precipitate was filtered and dried, affording **73**, 2-amino-5-bromo-4-chloronicotinaldehyde (42.0 g, 178 mmol, 93% yield). ^{1}H NMR (400 MHz, DMSO-d_6) δ 10.31 (s, 1H), 8.45 (s, 1H), 8.28 - 7.83 (br s, 2H).

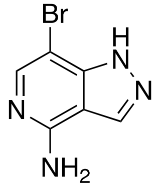

7-bromo-1*H*-pyrazolo[4,3-*c*]pyridin-4-amine (**74**).
Hydrazine monohydrate (53.6 g, 1.07 mol, 52.2 mL) was added to the solution of **73** (42.0 g, 178 mmol) in dioxane (450 mL). The resulting mixture was stirred at 100 °C for 48 h. The volatiles were removed under reduced pressure and the residue was diluted with cold water (150 mL). The resulting brown precipitate was filtered and dried, affording **74**, 7-bromo-1*H*-pyrazolo[4,3-*c*]pyridin-4-amine (26.2 g, 123 mmol, 69 % yield). LCMS(ESI): $[M+H]^+$ m/z: calcd 213.0; found 213.2; Rt = 0.288 min. ^{1}H NMR (400 MHz, DMSO-d_6) δ 13.43 (br s, 1H), 8.24 (s, 1H), 7.67 (s, 1H), 6.80-6.90 (2H, br s).

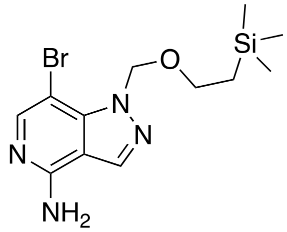

7-bromo-1-((2-(trimethylsilyl)ethoxy)methyl)-1*H*-pyrazolo[4,3-*c*]pyridin-4-amine (**75**).
Sodium hydride (in oil dispersion) 60% dispersion in mineral oil (5.89 g, 154 mmol, 60 % purity) was added portion-wise to a solution of **74** (26.2 g, 123 mmol) in dimethylformamide (250 mL) which was cooled to 10 °C. After H_2 evolution ceased, 2-(chloromethoxy)ethyl-trimethyl-silane (22.6 g, 135 mmol, 23.9 mL) was added dropwise. The resulting mixture was then stirred at ambient temperature for 3 h, after which it was concentrated under reduced pressure and the residue was purified by gradient column chromatography (SiO_2, $CHCl_3$/ACN) affording **75**, 7-bromo-1-((2-(trimethylsilyl)ethoxy)methyl)-1*H*-pyrazolo[4,3-*c*]pyridin-4-amine (14.8 g, 43.1 mmol, 35% yield), which exists as two regioisomers (protection at each nitrogen). ^{1}H NMR (400 MHz, $CDCl_3$) δ 7.94 (s, 2H), 5.98 (s, 2H), 5.11 (br s, 2H), 3.68 - 3.53 (t, 2H), 1 - 0.8 (t, 2H), 0.07 - -0.18 (m, 9H) and ^{1}H NMR (400 MHz, CDCl3) δ 8.29 (s, 1H), 7.81 (s, 1H), 5.71 (s, 2H), 5.46 - 4.71 (br s, 2H), 3.74 - 3.59 (t, 2H), 1.01 - 0.83 (t, 2H), -0.03 (s, 9H). LCMS (ESI): [M+H]+ m/z: calcd 342.05; found [343]+; Rt = 0.952 and 1.001 min.

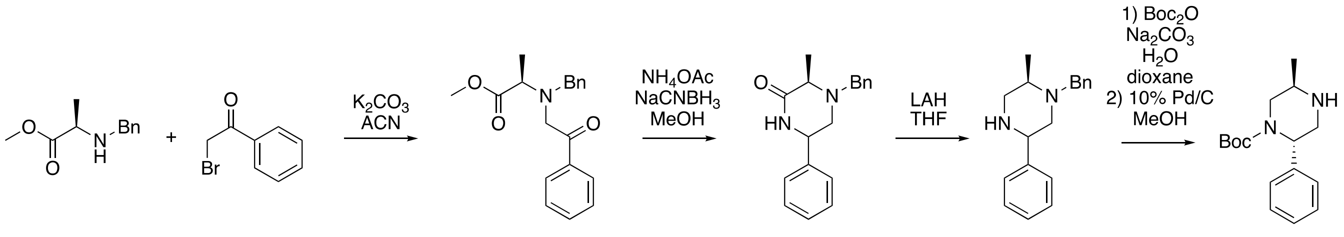

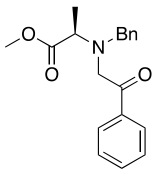

Methyl *N*-benzyl-*N*-(2-oxo-2-phenylethyl)-*D*-alaninate.

Methyl benzyl-*D*-alaninate (1 eq) was dissolved in MeCN and potassium carbonate - granular (1.1 eq) was added followed by addition of 2-bromo-1-phenylethan-1-one (1 eq). The resulting mixture was vigorously stirred overnight. The reaction mixture was concentrated in vacuum and water was added to the residue. The resulting mixture was extracted with MTBE and combined organic layers were dried over Na_2SO_4, filtered and evaporated to obtain methyl *N*-benzyl-*N*-(2-oxo-2-phenylethyl)-*D*-alaninate which was used further without purification. LCMS (ESI): [M]+ m/z: calcd 311.2; found 312.2; Rt = 1.439 min.

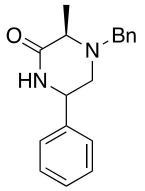

(3*R*)-4-benzyl-3-methyl-6-phenylpiperazin-2-one.
Methyl *N*-benzyl-*N*-(2-oxo-2-phenylethyl)-*D*-alaninate (1 eq) was dissolved in MeOH and ammonium acetate (10 eq) was added, followed by addition of sodium cyanoborohydride (1.2 eq). The resulting mixture was stirred for 1 h and then heated to reflux and stirred overnight. The reaction mixture was cooled and concentrated under vacuum. The residue was basified by addition of aq. K_2CO_3. The resulting mixture was extracted with MTBE and combined organic layers were dried over Na_2SO_4, filtered, and evaporated. The residue was purified by column chromatography (EtOAc:Hexane from 2:1 to 5:1) to obtain (3*R*)-4-benzyl-3-methyl-6-phenylpiperazin-2-one, 77% yield. LCMS (ESI): [M]+ m/z: calcd 280.2; found 281.2; Rt = 1.123 min.

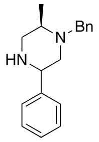

(2*R*)-1-benzyl-2-methyl-5-phenylpiperazine.
LAH (3 eq) was suspended in THF, and the resulting suspension was heated to reflux. A solution of (3*R*)-4-benzyl-3-methyl-6-phenylpiperazin-2-one (1 eq) in THF was added dropwise to the suspension, maintaining a gentle reflux. After addition was complete, the reaction mixture was refluxed for 4 h, then allowed to cool to room temperature and stirred overnight. Water was carefully added dropwise to the reaction mixture followed by addition of aq. KOH solution and water. The resulting mixture was stirred for 30 min and filtered. The filter cake was rinsed with THF, and the filtrate was concentrated under vacuum to obtain (2*R*)-1-benzyl-2-methyl-5-phenylpiperazine, 85% yield. LCMS (ESI): [M]+ m/z: calcd 266.2; found 267.2; Rt = 0.959 min.

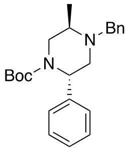

tert-butyl (2*S*,5*R*)-4-benzyl-5-methyl-2-phenylpiperazine-1-carboxylate.
(2*R*)-1-benzyl-2-methyl-5-phenylpiperazine (1 eq) was dissolved in dioxane and sodium carbonate (2 eq) was added followed by addition of water. Di-tert-butyl dicarbonate (2 eq) was added dropwise and the reaction mixture was stirred overnight, then concentrated under vacuum. The residue was diluted with water and the resulting mixture was extracted with ethyl acetate twice. The combined organic layers were washed with brine, dried over Na_2SO_4, filtered, and evaporated. The residue was purified, and *trans* isomer separated from *cis* isomer by column chromatography, eluting with a mixture of hexane and EtOAc (15:1) to obtain *tert*-butyl (2*S*,5*R*)-4-benzyl-5-methyl-2-phenylpiperazine-1-carboxylate, 26% yield. LCMS (ESI): [M]+ m/z: calcd 366.2; found 367.2; Rt = 3.202 min.

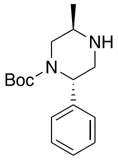

tert-butyl (2*S*,5*R*)-5-methyl-2-phenylpiperazine-1-carboxylate.
tert-butyl (2*S*,5*R*)-4-benzyl-5-methyl-2-phenylpiperazine-1-carboxylate (1 eq) was dissolved in MeOH and palladium, 10% on carbon, Type 487, dry (0.4 eq) was added. The reaction mixture was evacuated and backfilled three times with H_2 and then stirred at 1 atm (balloon) overnight. The catalyst was filtered off and the filtrate was concentrated under vacuum to obtain *tert*-butyl (2*S*,5*R*)-5-methyl-2-phenylpiperazine-1-carboxylate, 98% yield. LCMS (ESI): [M]+ m/z: calcd 276.2; found 277.2; Rt = 0.997 min.

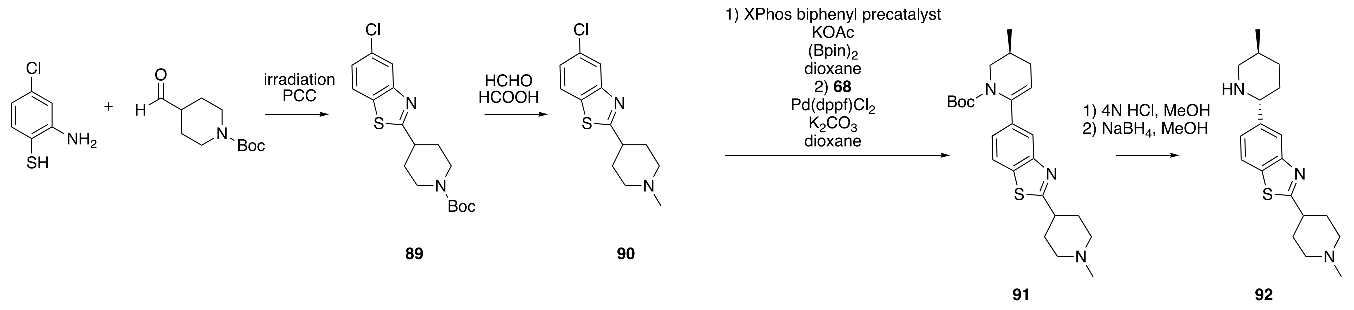

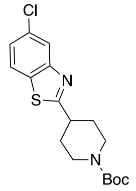

tert-butyl 4-(5-chlorobenzo[*d*]thiazol-2-yl)piperidine-1-carboxylate (**89**).
2-amino-4-chlorobenzenethiol (100 g, 626 mmol, 1.00 eq) was stirred in ethyl acetate (700 mL). *tert*-butyl 4-formylpiperidine-1-carboxylate (160 g, 751 mmol, 1.20 eq) was added and the reaction mixture was irradiated with a 100 W blue LED bulb at 25 °C for 3 h. The white solid that formed was filtered and dissolved in THF (1500 mL) in a sealed tube. PCC (189 g, 877 mmol, 1.10 eq) and dioxosilane (191 g, 3.19 mol, 4.00 eq) were added under N_2 at 25 °C, then DCM (1500 mL) was added, and the mixture was stirred for 5 h at 20 - 25 °C. The reaction mixture was filtered, and the filtrate was concentrated. The residue was purified by column chromatography (SiO_2, petroleum ether / ethyl acetate = 100 / 1 to 2 / 1) to afford *tert*-butyl 4-(5-chlorobenzo[*d*]thiazol-2-yl)piperidine-1-carboxylate, **89** (550 g, 65% yield) as a white solid. ^{1}H NMR (400 MHz, $CDCl_3$) δ 8.12 (d, *J* = 8.53 Hz, 1H), 8.04 (d, *J* = 2.01 Hz, 1H), 7.47 (dd, *J* = 8.53, 2.01 Hz, 1H), 4.03 (br d, *J* = 12.30 Hz, 2H), 3.34 - 3.40 (m, 1H), 2.93 (br s, 2H), 2.09 (br d, *J* = 10.67 Hz, 2H), 1.58 - 1.70 (m, 1H), 1.54 - 1.71 (m, 1H), 1.32 - 1.45 (m, 9H).

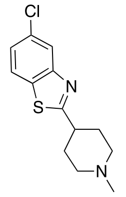

5-chloro-2-(1-methylpiperidin-4-yl)benzo[*d*]thiazole (**90**).
89 (500 g, 1.42 mol, 1.00 eq) was stirred with formic acid (1.00 L) and formaldehyde (575 g, 7.08 mol, 527 mL, 37.0% purity, 5.00 eq) under N_2 at 25 °C, then stirred for 3 h at 90 - 95 °C. HCl in ethyl acetate (4 M, 3000 mL) was added. The resultant white solid was filtered and dried at 45 °C. 5-chloro-2-(1-methylpiperidin-4-yl)benzo[*d*]thiazole, **90** (395 g, 1.12 mol, 60% yield, HCl) as a white solid. ^{1}H NMR (400 MHz, $CDCl_3$) δ 7.95 (d, *J* = 1.97 Hz, 1H), 7.74 - 7.78 (m, 1H), 7.30 - 7.36 (m, 1H), 3.09 (tt, *J* = 11.51, 3.84 Hz, 1H), 2.98 (br d, *J* = 11.84 Hz, 2H), 2.34 (s, 3H), 2.08 - 2.22 (m, 4H), 1.93 - 2.03 (m, 2H).

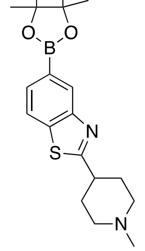

2-(1-methylpiperidin-4-yl)-5-(4,4,5,5-tetramethyl-1,3,2-dioxaborolan-2-yl)benzo[*d*]thiazole.
90 (200 g, 659 mmol, 1.00 eq , HCl) was stirred with XPhos biphenyl Precatalyst (18.2 g, 23.1 mmol, 0.035 eq) and $(Bpin)_2$ (251 g, 989 mmol, 1.50 eq) in dioxane (3.0 L). The reaction mixture was purged with N_2 three times. KOAc (259 g, 2.64 mol, 4.00 eq) was added and the reaction mixture was stirred for 10 h at 105 - 110 °C. The reaction mixture was filtered, and the filtrate was concentrated and the resultant residue was purified by column chromatography (SiO_2, petroleum ether/ethyl acetate = 10/1 to 0/1) to afford 2-(1-methylpiperidin-4-yl)-5-(4,4,5,5-tetramethyl-1,3,2-dioxaborolan-2-yl)benzo[*d*]thiazole (93 g, 259 mmol, 39% yield) as a yellow solid.

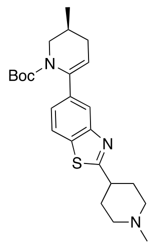

tert-butyl (*S*)-3-methyl-6-(2-(1-methylpiperidin-4-yl)benzo[*d*]thiazol-5-yl)-3,4-dihydropyridine-1(2*H*)-carboxylate (**91**).
2-(1-methylpiperidin-4-yl)-5-(4,4,5,5-tetramethyl-1,3,2-dioxaborolan-2-yl)benzo[*d*]thiazole was stirred with **68** in dioxane (1.00 L). H_2O (500 mL) and K_2CO_3 (107 g, 778 mmol, 3.00 eq) were added and the suspension was degassed under vacuum and purged with N_2 three times. Pd(dppf)Cl_2 (9.50 g, 12.9 mmol, 0.05 eq) was added and the suspension was degassed under vacuum and purged with N_2 three times. The reaction was stirred for 12 h at 90 °C. The reaction mixture was then cooled to 20 °C and filtered and the solid was washed with ethyl acetate (400 mL). The combined organics were concentrated and the residue was dissolved in ethyl acetate (400mL) and washed with H_2O (200 mL). The aqueous was extracted with ethyl acetate (100 mL) and the combined organic layer was concentrated. The residue was purified by column chromatography (SiO_2, petroleum ether/ethyl acetate = 40/1 to 8/1). *tert*-butyl (*S*)-3-methyl-6-(2-(1-methylpiperidin-4-yl)benzo[*d*]thiazol-5-yl)-3,4-dihydropyridine-1(2*H*)-carboxylate, **91** (66.2 g, 155 mmol, 60% yield) as a brown oil. ^{1}H NMR (400 MHz, $CDCl_3$) δ 7.91 (s, 1H), 7.75 (d, *J* = 8.33 Hz, 1H), 7.31 (d, *J* = 8.11 Hz, 1H), 5.39 (br s, 1H), 4.11 (br d, *J* = 8.33 Hz, 1H), 3.13 (br d, *J* = 9.21 Hz, 1H), 2.96 - 3.08 (m, 3H), 2.40 (s, 3H), 2.24 (br d, *J* = 11.18 Hz, 3H), 1.96 - 2.09 (m, 3H), 1.88 (ddd, *J* = 18.74, 8.66, 3.73 Hz, 1H), 1.53 (s, 1H) ,1.25 (s, 4H), 1.04 (br d, *J* = 6.80 Hz, 12H).

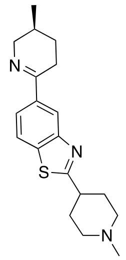

(*S*)-5-(5-methyl-3,4,5,6-tetrahydropyridin-2-yl)-2-(1-methylpiperidin-4-yl)benzo[*d*]thiazole.
91 (66.0 g, 154 mmol, 1.00 eq) was stirred in MeOH (200 mL) and 4 N HCl / MeOH (660 mL) for 5 h at 20 °C. The solvent was removed under reduced pressure and the residue was dissolved in H_2O (300 mL) and saturated Na_2CO_3 was added to bring the solution to pH = 8 - 9. The aqueous solution was extracted with ethyl acetate (200 mL x 3) and the combined organic layer was concentrated to afford (*S*)-5-(5-methyl-3,4,5,6-tetrahydropyridin-2-yl)-2-(1-methylpiperidin-4-yl)benzo[*d*]thiazole (44.3 g, 135 mmol, 88% yield) as a brown solid which was used in the next step without purification. ^{1}H NMR (400 MHz, $CDCl_3$) δ 8.27 (s, 1H), 7.96 (br d, *J* = 8.50 Hz, 1H), 7.83 (dd, *J* = 8.51, 1.25 Hz, 1H), 4.04 (br dd, *J* = 17.57, 2.19 Hz, 1H), 3.22 - 3.36 (m, 1H), 3.10 (br t, *J* = 10.76 Hz, 1H), 2.99 (br d, *J* = 10.63 Hz, 2H), 2.87 (br dd, *J* = 18.07, 3.69 Hz, 1H), 2.59 - 2.74 (m, 1H), 2.34 (d, *J* = 1.38 Hz, 3H), 2.07 - 2.26 (m, 5H), 1.89 - 2.06 (m, 4H), 1.75 (br d, *J* = 3.38 Hz, 1H) ,1.34 - 1.51 (m, 1H), 1.22 - 1.30 (m, 1H), 0.93 - 1.08 (m, 3H).

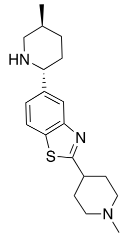

5-((2*R*,5*S*)-5-methylpiperidin-2-yl)-2-(1-methylpiperidin-4-yl)benzo[*d*]thiazole (**92**).
(*S*)-5-(5-methyl-3,4,5,6-tetrahydropyridin-2-yl)-2-(1-methylpiperidin-4-yl)benzo[*d*]thiazole was dissolved in MeOH (250 mL) and $NaBH_4$ (5.63 g, 149 mmol, 1.10 eq) was added at 0 °C. The reaction was stirred at 0 °C for 1 h, after which the reaction mixture was quenched with H_2O (200 mL) and the organic solvent was removed under reduced pressure. The aqueous layer was extracted with DCM (200 mL x 3) and the combined organic layer was concentrated to afford 5-((2*R*,5*S*)-5-methylpiperidin-2-yl)-2-(1-methylpiperidin-4-yl)benzo[*d*]thiazole, **92** (42.7 g, 129 mmol, 96% yield) as a brown solid which was used in the next step without purification. ^{1}H NMR (400 MHz, $CDCl_3$) δ 7.92 - 8.02 (m, 1 H), 7.95 (s, 1H), 7.78 (br d, *J* = 7.38 Hz, 1H), 7.43 (br d, *J* = 8.25 Hz, 1H), 3.69 (br d, *J* = 11.13 Hz, 1H), 3.17 (br d, *J* = 11.26 Hz, 1H), 3.09 (br t, *J* = 11.01 Hz, 1H), 2.98 (br d, *J* = 10.63 Hz, 2H), 2.46 (br t, *J* = 11.07 Hz, 1H), 2.34 (s, 3H), 2.07 - 2.23 (m, 4H), 1.94 - 2.06 (m, 2H), 1.88 (br t, *J* = 13.70 Hz, 3H), 1.54 - 1.76 (m, 2H), 1.11 - 1.29 (m, 1H), 0.91 (br d, *J* = 5.63 Hz, 3H).

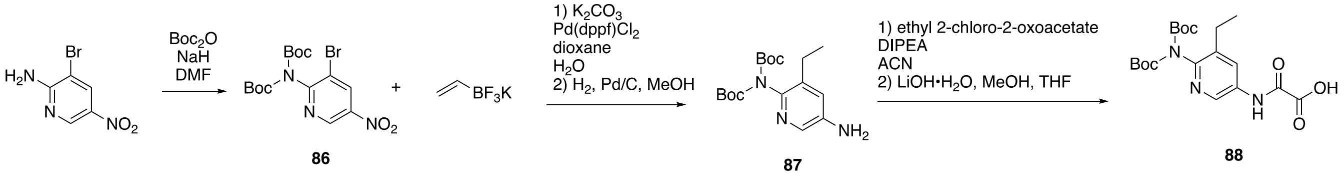

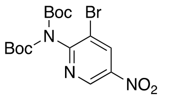

tert-butyl (3-bromo-5-nitropyridin-2-yl)(*tert*-butoxycarbonyl)carbamate (**86**).
3-bromo-5-nitropyridin-2-amine (1.50 kg, 6.88 mol) was stirred in THF (7.50 L) at 0 °C. DMAP (168 g, 1.38 mol) was added, and the mixture was stirred for 0.5 h at 0 °C. Boc_2O (3.15 kg, 14.4 mol, 3.32 L) in THF (3.00 L) was added and the reaction mixture was stirred for 2 h at 25 °C. The reaction mixture was poured into water (15.0 L) and separated. The aqueous layer was extracted with EtOAc (5.00 L x 2) and the combined organic layer was dried with Na_2SO_4 and concentrated to give *tert*-butyl 3-bromo-5-nitropyridin-2-yl)(*tert*-butoxycarbonyl)carbamate, **86** (5.60 kg, 97% yield) as a white solid which was used without further purification in the next reaction. ^{1}H NMR (400 MHz, $CDCl_3$) δ 9.28 (d, J = 2.41 Hz, 1H), 8.75 (d, J = 2.41 Hz, 1H), 1.43 (s, 18H).

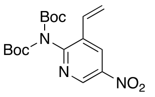

tert-butyl (*tert*-butoxycarbonyl)(5-nitro-3-vinylpyridin-2-yl)carbamate.
86 (2.80 kg, 6.69 mol) was stirred in dioxane (22.4 L) and H_2O (5.60 L). Potassium vinyltrifluoroborate (1.08 kg, 8.03 mol) was added at 25 °C. K_2CO_3 (4.63 kg, 33.4 mol) was then added and the vessel was purged with Ar three times. Pd(dppf)Cl_2•CH_2Cl_2 (218 g, 267 mmol) was added and the reaction mixture was stirred for 2 h at 80 °C after which it was poured into water (28.0 L) and separated. The aqueous solution was extracted with EtOAc (10.0 L x 2) and the organic layer was dried with Na_2SO_4 and concentrated. *tert*-butyl (*tert*-butoxycarbonyl)(5-nitro-3-vinylpyridin-2-yl)carbamate (4.50 kg, crude) was obtained as a yellow solid and was used in the next step without purification. ^{1}H NMR (400 MHz, MeOD-d_4) δ 9.21 (d, J = 2.64 Hz, 1H), 8.86 (d, J = 2.51 Hz, 1H), 6.75 (dd, J = 17.50, 11.11 Hz, 1H), 6.14 (d, J = 17.44 Hz, 1H), 5.73 (d, J = 11.04 Hz, 1H), 1.38 (s, 18H).

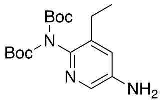

tert-butyl (5-amino-3-ethylpyridin-2-yl)(*tert*-butoxycarbonyl)carbamate (**87**).
Pd/C (450 g, 10% wt of Pd) and Pd(OH)$_2$/C (432 g, 20% wt of Pd(OH)$_2$) were stirred in MeOH (45.0 L). *tert*-butyl (*tert*-butoxycarbonyl)(5-nitro-3-vinylpyridin-2-yl)carbamate (4.50 kg, 13.3 mol) was added and the resulting mixture was stirred under H_2 (50 psi) at 50 °C for 24 h. The reaction was filtered and the filtrate was concentrated to give *tert*-butyl (5-amino-3-ethylpyridin-2-yl)(*tert*-butoxycarbonyl)carbamate, **87** (3.80 kg, 91% yield) as a yellow solid which was used in the next step without purification. ^{1}H NMR (400 MHz, MeOD-d_4) δ 7.67 (d, J = 2.75 Hz, 1H), 7.06 (d, J = 2.63 Hz, 1H), 2.44 (q, J = 7.55 Hz, 2H), 1.38 (s, 19H), 1.21 (t, J = 7.63 Hz, 3H).

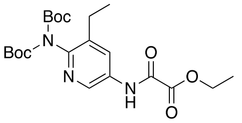

Ethyl 2-((6-(bis(*tert*-butoxycarbonyl)amino)-5-ethylpyridin-3-yl)amino)-2-oxoacetate.
87 (1.90 kg, 5.63 mol) was stirred in ACN (13.3 L) at 0 °C under N_2 atmosphere. DIEA (1.09 kg, 8.45 mol, 1.47 L) was added dropwise followed by ethyl 2-chloro-2-oxo-acetate (922 g, 6.76 mol, 756 mL) dropwise. The reaction mixture was stirred for 24 h at 25 °C after which it was poured into water (12.0 L) and separated. The aqueous was extracted with EtOAc (5.00 L x 2) and the organic layer was dried with Na_2SO_4 and concentrated. The residue was purified by column chromatography (SiO_2, petroleum ether / ethyl acetate = 100/1 to 1/1) to give ethyl 2-((6-(bis(*tert*-butoxycarbonyl)amino)-5-ethylpyridin-3-yl)amino)-2-oxoacetate (3.00 kg, 61% yield) as a white solid. ^{1}H NMR (400 MHz, $CDCl_3$) δ 9.02 (s, 1H), 8.49 (d, J = 2.63 Hz, 1H), 8.21 (d, J = 2.50 Hz, 1H), 4.43 (q, J = 7.13 Hz, 2H), 2.58 (q, J = 7.50 Hz, 2H), 1.41 - 1.45 (m, 3H), 1.39 (s, 18H), 1.24 (t, J = 7.57 Hz, 3H).

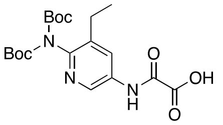

2-((6-(bis(*tert*-butoxycarbonyl)amino)-5-ethylpyridin-3-yl)amino)-2-oxoacetic acid (**88**).

Ethyl 2-((6-(bis(*tert*-butoxycarbonyl)amino)-5-ethylpyridin-3-yl)amino)-2-oxoacetate (3.00 kg, 6.86 mol) was stirred in THF (6.00 L) and MeOH (6.00 L) at 0 °C. LiOH•H_2O (575 g, 13.7 mol) in H_2O (6.00 mL) was added dropwise at 0 °C and the mixture was stirred for 3 h after which the mixture was acidified to pH = 3 with a solution of 1 M HCl. The precipitate that formed was filtered and dried to give 2-((6-(bis(*tert*-butoxycarbonyl)amino)-5-ethylpyridin-3-yl)amino)-2-oxoacetic acid, **88** (1.80 kg, 68%) as a white solid which was used in the next step reaction without purification. ^{1}H NMR (400 MHz, MeOD-d_4) δ 8.71 (d, J = 2.50 Hz, 1H), 8.26 (d, J = 2.50 Hz, 1H), 2.59 (q, J = 7.59 Hz, 2H), 1.39 (s, 18H), 1.27 (t, J = 7.57 Hz, 3H).

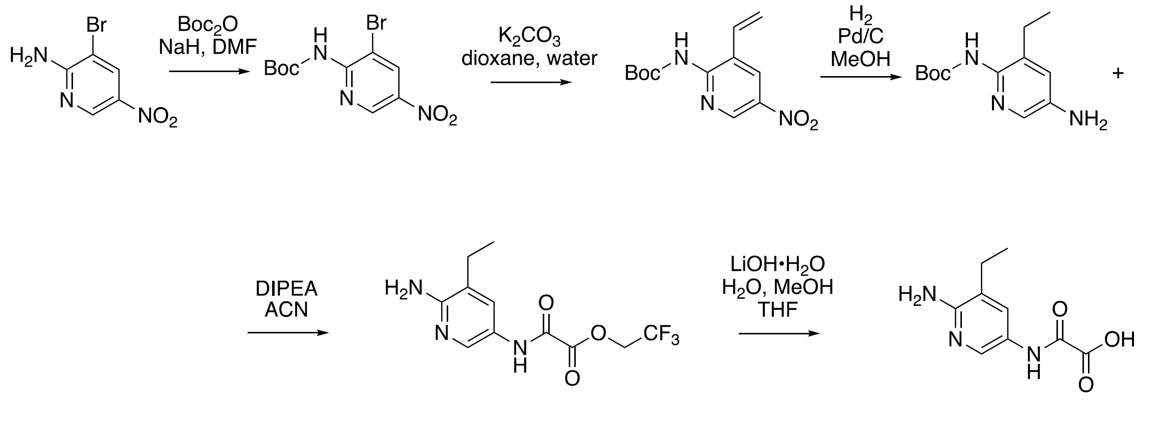

Intermediate 2a

tert-butyl (3-bromo-5-nitropyridin-2-yl)carbamate.

To a solution of 3-bromo-5-nitro-pyridin-2-amine (30 g, 137.61 mmol) in DMF (200 mL) was added sodium hydride (in oil dispersion) 60% dispersion in mineral oil (5.54 g, 138.42 mmol, 60% purity) portionwise at 0 °C. The resulting mixture was stirred for 0.5 h (to the end of gas evolution) and a solution of di-tert-butyl dicarbonate (31.53 g, 144.49 mmol, 33.16 mL) in DMF (50 mL) was added dropwise. The resulting mixture was stirred at 25 °C for 12 h. The mixture was quenched with water (1000 mL), the precipitate was filtered and dissolved in EtOAc (900 mL) + THF (100 mL), washed with brine (2 x 300 mL), dried over Na_2SO_4, and evaporated in vacuo to obtain white crystals. This precipitate was filtered, washed with EtOAc (30 mL), MTBE (2 x 50 mL) and dried to obtain *tert*-butyl (3-bromo-5-nitropyridin-2-yl)carbamate (32 g, 100.59 mmol, 73% yield) . ^{1}H NMR (500 MHz, DMSO-d_6) δ 1.47 (s, 9H), 3.3 (s, 2H), 8.81 (s, 1H), 9.15 (s, 1H), 9.88 (s, 1H). LCMS (ESI): [M-tBu+H]+ m/z: calcd 318.1; found 264.0; Rt = 1.152 min.

tert-butyl *N*-(5-nitro-3-vinyl-2-pyridyl)carbamate.

A solution of *tert*-butyl (3-bromo-5-nitropyridin-2-yl)carbamate (27 g, 84.87 mmol), potassium vinyltrifluoroborate (13.64 g, 101.85 mmol) and potassium carbonate (58.65 g, 424.36 mmol, 25.61 mL) in dioxane (400 mL) and water (100 mL) was evacuated and refilled with Ar three times. To this solution was added [1,1′-bis(diphenylphosphino)ferrocene]dichloropalladium(II), complex with dichloromethane (3.47 g, 4.24 mmol) and the resulting mixture was stirred at 80 °C for 2 h. The resulting mixture was cooled, filtered, and the solvent was removed. The residue was dissolved in water (350 mL) and extracted with EtOAc:THF 9:1 (3 x 300 mL). The organic layer was dried over Na_2SO_4. 90 % of the solvent was removed and a precipitate formed and was filtered, washed with MTBE, and dried to give *tert*-butyl *N*-(5-nitro-3-vinyl-2-pyridyl)carbamate (13.5 g, 50.89 mmol, 60% yield). ^{1}H NMR (400 MHz, DMSO-d_6) δ 1.45 (s, 9H), 3.3 (s, 2H), 5.54 (d, 1H), 6.06 (d, 1H), 6.75 (dd, 1H), 8.07 (s, 1H), 9.08 (s, 1H), 9.93 (s, 1H). LCMS (ESI): [M-tBu+H]+ m/z: calcd 265.1; found 210.0; Rt = 1.311 min.

tert-butyl *N*-(5-amino-3-ethyl-2-pyridyl)carbamate.

To a solution of *tert*-butyl *N*-(5-nitro-3-vinyl-2-pyridyl)carbamate (13 g, 49.01 mmol) in methanol (400 mL), palladium, 10% on carbon (52.15 g, 49.01 mmol, 10% wt Pd) was added. The resulting mixture was stirred under H_2 atmosphere at 25 °C for 24 h. The catalyst was filtered, the solvent was evaporated in vacuo, the residue was dissolved in ACN (150 mL) and evaporated in vacuo to obtain *tert*-butyl *N*-(5-amino-3-ethyl-2-pyridyl)carbamate (11 g, 46.36 mmol, 95% yield). ^{1}H NMR (400 MHz, DMSO-d_6) δ 1.03 (t, 3H), 1.36 (s, 9H), 2.37 (q, 2H), 3.3 (s, 2H), 6.76 (s, 1H), 7.52 (s, 1H), 8.40 (s, 1H). LCMS (ESI): [M+H]+ m/z: calcd 237.3; found 238.2; Rt = 0.901 min.

2,2,2-trifluoroethyl 2-((6-amino-5-ethylpyridin-3-yl)amino)-2-oxoacetate.

To a solution of *tert*-butyl *N*-(5-amino-3-ethyl-2-pyridyl)carbamate (11 g, 46.36 mmol) and DIPEA (8.99 g, 69.53 mmol, 12.11 mL) in ACN (350 mL) was added 2,2,2-trifluoroethyl 2-chloro-2-oxo-acetate (10.16 g, 53.31 mmol) dropwise at 0 °C under argon. The reaction mixture was then stirred for 4 h at room temperature, then evaporated in vacuo. The residue, 2,2,2-trifluoroethyl 2-((6-amino-5-ethylpyridin-3-yl)amino)-2-oxoacetate, was used for the next step without purification.

2-((6-amino-5-ethylpyridin-3-yl)amino)-2-oxoacetic acid, **Intermediate 2a**.

To a solution of crude 2,2,2-trifluoroethyl 2-((6-amino-5-ethylpyridin-3-yl)amino)-2-oxoacetate obtained in the previous step in methanol (100 mL) and THF (100 mL), a solution of lithium hydroxide, monohydrate (5.58 g, 132.88 mmol, 1.85 mL) in water (150 mL) was added. The resulting mixture was stirred at 0 °C for 3 h, then the organic solvents were removed and the aqueous solution was extracted with MTBE (3 x 200 mL) and acidified with a solution of sodium bisulfate monohydrate (9.63 g, 69.76 mmol) in water (50 mL). The precipitate that formed was filtered and dried to obtain 2-((6-amino-5-ethylpyridin-3-yl)amino)-2-oxoacetic acid (13.5 g, 43.4 mmol, 94% yield) as a white solid. ^{1}H NMR (400 MHz, DMSO-d_6) δ 1.08 (t, 3H), 1.39 (s, 9H), 2.52 (q, 2H), 8.00 (s, 1H), 8.55 (s, 1H), 8.95 (s, 1H), 10.78 (s, 1H).

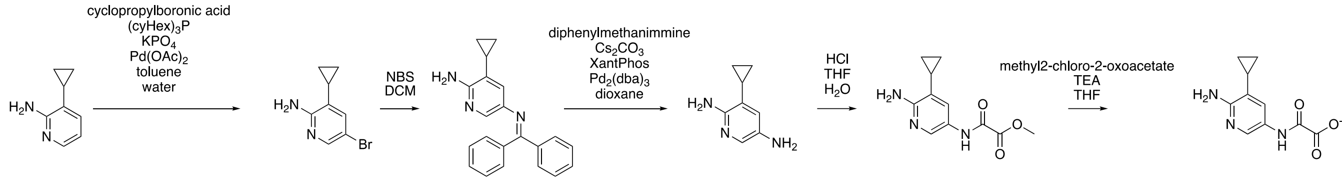

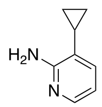

3-cyclopropylpyridin-2-amine.

To a solution of 3-bromopyridin-2-amine (50.0 g, 289 mmol) in toluene (500 mL) and water (100 mL) was added cyclopropyl boronic acid (32.3 g, 376 mmol), tricyclohexylphosphine (8.10 g, 28.9 mmol), potassium phosphate tribasic anhydrous (184 g, 867 mmol) and palladium (II) acetate (3.24 g, 14.5 mmol). The reaction mixture purged with Ar then stirred at 90 °C for 16 h. The mixture was concentrated in vacuo and the resulting residue was diluted with water and extracted with MTBE (3 x 500 mL). The combined organic layers were dried over sodium sulfate and concentrated to dryness in vacuo to afford 3-cyclopropylpyridin-2-amine (59.0 g, crude) as a brown oil. LCMS (ESI): [M+H]+ m/z: calcd 135.1; found 135.2; Rt = 0.609.

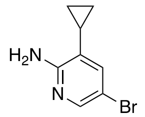

5-bromo-3-cyclopropylpyridin-2-amine.

N-bromosuccinimide (50.9 g, 286 mmol, 24.2 mL) was added portion-wise to a solution of 3-cyclopropylpyridin-2-amine (59.0 g, 286 mmol) in dry DCM. The reaction mixture was stirred at room temperature for 3 h, after which it was washed with water. The aqueous layer was extracted with DCM. The combined organic extracts were washed with brine and dried over Na_2SO_4. The solvent was removed under reduced pressure and the residue was purified by column chromatography (Interchim; 800g SiO2, chloroform/acetonitrile with acetonitrile from 0~40%, flow rate = 150 mL/min, Rv = 5-6 CV) to afford 5-bromo-3-cyclopropylpyridin-2-amine (30.4 g, 143 mmol, 50% yield) as a brown solid. LCMS (ESI): [M+H]+ m/z: calcd 213.01 and 215.01; found 215.0; Rt = 0.834.

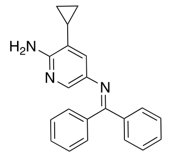

5-(benzhydrylideneamino)-3-cyclopropyl-pyridin-2-amine.

5-bromo-3-cyclopropylpyridin-2-amine (15.0 g, 70.4 mmol), diphenylmethanimine (14.0 g, 77.4 mmol, 12.9 mL), cesium carbonate (68.8 g, 211 mmol), XantPhos (2.04 g, 3.52 mmol) and $Pd_2(dba)_3$ (1.61 g, 1.76 mmol) were stirred in dioxane (250 mL) and the mixture was purged with Ar and stirred at 90 °C for 12 h under Ar atmosphere. The reaction mixture was cooled to room temperature, diluted with MTBE (250 mL), and filtered. The filtrate was concentrated in vacuo to afford 5-(benzhydrylideneamino)-3-cyclopropyl-pyridin-2-amine (27.3 g, crude) as brown gum, ^{1}H NMR (400 MHz, $CDCl_3$) δ 0.26 – 0.27 (m, 2H), 0.75 – 0.76 (m, 2H), 1.46 – 1.47 (m, 1H), 4.52 (br, 2H), 6.62 (m, 1H), 7.08 (m, 2H), 7.29 - 7.41 (m, 6H), 7.53 (m, 1H), 7.69 (m, 2H).

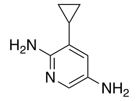

3-cyclopropylpyridine-2,5-diamine.

5-(benzhydrylideneamino)-3-cyclopropyl-pyridin-2-amine (15.0 g, 35.9 mmol) was then stirred in THF (150 mL) and water (100 mL) and hydrochloric acid, 36% w/w aq. soln. (4.50 g, 123 mmol, 5.63 mL) was added. The resulting mixture was stirred at 21 °C for 18 h and then diluted with water (20 mL) and extracted with MTBE (2 x 25 mL). The organic layers were discarded. The aqueous layer was basified to pH≈10-11 with solid K_2CO_3 and extracted with DCM (4 x 25 mL). The combined organic layers were dried over K_2CO_3 and concentrated under reduced pressure to afford 3-cyclopropylpyridine-2,5-diamine (5.30 g, crude) as a red oil. LCMS (ESI): [M+H]+ m/z: calcd 150.11; found 150.2; Rt = 0.323.

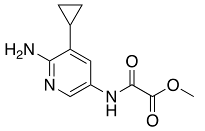

Methyl 2-[(6-amino-5-cyclopropyl-3-pyridyl)amino]-2-oxo-acetate.

Methyl 2-chloro-2-oxo-acetate (3.48 g, 28.4 mmol) was added to a solution of 3-cyclopropylpyridine-2,5-diamine (5.30 g, 35.5 mmol) and TEA (3.95 g, 39.1 mmol, 5.45 mL) in THF (25 mL) at 0 °C. After stirring at room temperature for 2 h, the resulting mixture was filtered and concentrated in vacuo. The residue was subjected to column chromatography (ISCO® Interchim; 120 g SiO_2, MTBE/methanol with methanol from 0 ~ 95 %, flow rate = 60 mL/min, Rf = 4.5 CV) to afford material that was further purified by HPLC (0.5-6.5 min 0-35 % water - ACN; flow: 30 mL/min, column: Waters SunFire C18, 100 x 19 mm, 5 µm) to afford methyl 2-[(6-amino-5-cyclopropyl-3-pyridyl)amino]-2-oxo-acetate (900 mg, 11 % yield) as a light-yellow solid. LCMS (ESI): [M+H]+ m/z: calcd 236.1; found 236.2; Rt = 0.519.

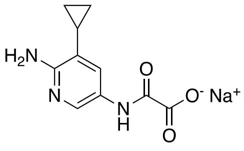

Sodium 2-((6-amino-5-cyclopropylpyridin-3-yl)amino)-2-oxoacetate, **Intermediate 3a**.

To a solution of methyl 2-[(6-amino-5-cyclopropyl-3-pyridyl)amino]-2-oxo-acetate in MeOH (50 mL), sodium hydroxide (153 mg, 3.83 mmol, 71.8 µL) was added and the mixture was stirred at room temperature for 1 h. The mixture was concentrated in vacuo to afford sodium 2-((6-amino-5-cyclopropylpyridin-3-yl)amino)-2-oxoacetate, **Intermediate 3a** (0.95 g, 100% yield) as a brown gum. LCMS (ESI): [M+H]+ m/z: calcd 222.08; found 222.2; Rt = 0.509.

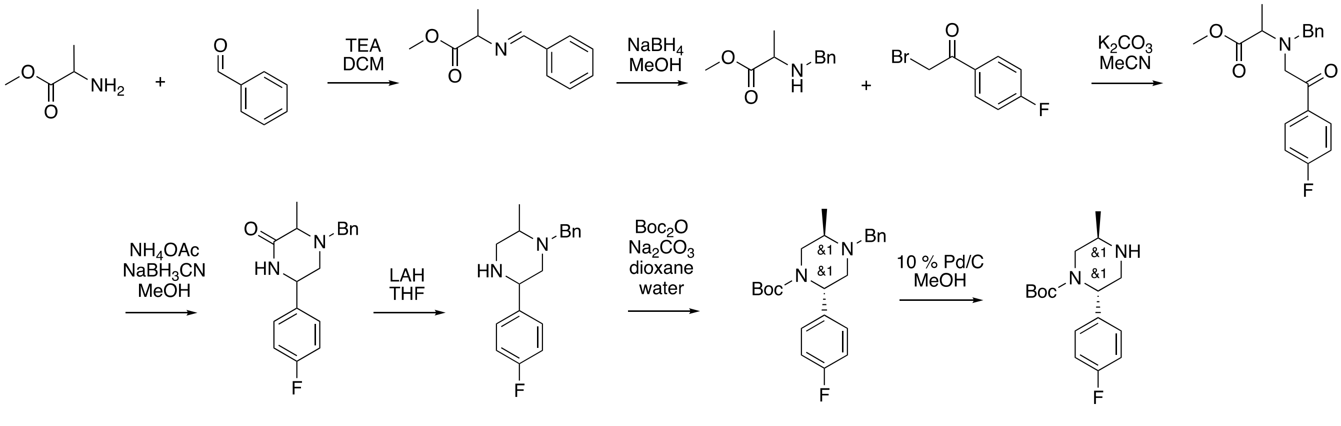

intermediate 23a

Methyl 2-[(*E*)-benzylideneamino]propanoate.

Methyl 2-aminopropanoate (31.65 g, 226.77 mmol, HCl) and benzaldehyde (24.06 g, 226.77 mmol) were stirred in DCM (380 mL). TEA (27.54 g, 272.12 mmol, 37.93 mL) was added and the solution was stirred overnight after which it was washed with water (75 mL), dried over Na_2SO_4, filtered, and evaporated to obtain methyl 2-[(*E*)-benzylideneamino]propanoate (33.16 g, 173.41 mmol, 76% yield). ^1H NMR (400 MHz, $CDCl_3$) δ (1.56 (d, 3H), 3.77 (s, 3H), 4.17 (m, 1H), 7.44 (m, 3H), 7.78 (m, 2H), 8.33 (m, 1H). LCMS (ESI): [M]+ m/z: calcd 191.2; found 192.2; Rt = 0.703 min.

Methyl 2-(benzylamino)propanoate.

Methyl 2-[(*E*)-benzylideneamino]propanoate (33.16 g, 173.41 mmol) was stirred in MeOH (500 mL) and cooled to 0 °C in an ice bath. Sodium borohydride (2.30 g, 60.69 mmol, 2.15 mL) was added portion wise and the reaction mixture was allowed to warm to room temperature and stirred overnight. Water (100 mL) was added and the organics were removed under vacuum. Water (150 mL) was added and the resulting mixture was extracted with DCM (2 x 200 mL), dried over Na_2SO_4, filtered and evaporated to obtain methyl 2-(benzylamino)propanoate (35.2 g, crude). ^1H NMR (400 MHz, $CDCl_3$) δ 1.35 (d, 3H), 1.77 (m, 1H), 3.44 (m, 1H), 3.70 (m, 1H), 3.75 (s, 3H), 3.80 (m, 1H), 7.39 (m, 5H). LCMS (ESI): $[M]^+$ m/z: calcd 193.2; found 194.2; Rt = 0.742 min.

Methyl 2-[benzyl-[2-(4-fluorophenyl)-2-oxo-ethyl]amino]propanoate.

Methyl 2-(benzylamino)propanoate (34.62 g, 179.14 mmol) was stirred in MeCN (900 mL) and potassium carbonate - granular (27.23 g, 197.05 mmol, 11.89 mL) was added, followed by 2-bromo-1-(4-fluorophenyl)ethanone (38.88 g, 179.14 mmol) and the resulting mixture was vigorously stirred overnight. The reaction mixture was concentrated under vacuum and then water (300 mL) was added to the residue. The resulting mixture was extracted with MTBE (2 x 300 mL) and combined organic layers were dried over Na_2SO_4, filtered, and evaporated to obtain methyl 2-[benzyl-[2-(4-fluorophenyl)-2-oxo-ethyl]amino]propanoate (62.57 g, crude) which was used further without purification. ^1H NMR (400 MHz, $CDCl_3$) δ 1.34 (d, 3H), 3.64 (m, 2H), 3.76 (s, 3H), 3.86 (m, 1H), 4.06 (m, 2H), 7.05 (m, 2H), 7.33 (m, 5H), 7.92 (m, 2H). LCMS (ESI): $[M]^+$ m/z: calcd 329.2; found 330.2; Rt = 1.392 min.

4-benzyl-6-(4-fluorophenyl)-3-methyl-piperazin-2-one.

Methyl 2-[benzyl-[2-(4-fluorophenyl)-2-oxo-ethyl]amino]propanoate (62.57 g, 189.97 mmol) was dissolved in MeOH (620 mL) and ammonium acetate (146.43 g, 1.90 mol) was added, followed by sodium cyanoborohydride (14.33 g, 227.97 mmol). The resulting mixture was stirred for 1 h and then heated to reflux overnight. The reaction mixture was then cooled and concentrated under vacuum. The residue was basified by addition of aq. K_2CO_3 (200g in 500ml of water). The resulting mixture was extracted with MTBE (2 x 500 mL) and the combined organic layers were dried over Na_2SO_4, filtered, and evaporated. The residue was purified by column chromatography (EtOAc:Hexane from 2:1 to 5:1) to obtain 4-benzyl-6-(4-fluorophenyl)-3-methyl-piperazin-2-one (26.41 g, 88.52 mmol, 47% yield). ^{1}H NMR (500 MHz, $CDCl_3$) δ 1.55 (d, 3H), 2.68 (m, 2H), 3.41 (m, 2H), 3.92 (m, 1H), 4.61 (m, 1H), 5.85 (m, 1H), 7.02 (m, 2H), 7.26 (m, 7H). LCMS (ESI): $[M]^+$ m/z: calcd 298.2; found 299.2; Rt = 1.174 min.

1-benzyl-5-(4-fluorophenyl)-2-methyl-piperazine.
Lithium aluminium hydride (10.08 g, 265.56 mmol) was suspended in THF (400 mL) and then heated to reflux. A solution of 4-benzyl-6-(4-fluorophenyl)-3-methyl-piperazin-2-one (26.41 g, 88.52 mmol) in THF (100 mL) was added dropwise, maintaining a gentle reflux. After the addition was complete, the reaction mixture was refluxed for 4 h and then cooled to room temperature and stirred overnight. Water (10 mL) was carefully added dropwise to the pre-cooled reaction mixture followed by addition of a aq. KOH solution (10 mL) and water (20 mL). The resulting mixture was stirred for 30 min and filtered. The filter cake was rinsed with THF (100 mL) and the filtrate was concentrated under vacuum to obtain 1-benzyl-5-(4-fluorophenyl)-2-methyl-piperazine (24.94 g, 87.70 mmol, 99% yield). ^{1}H NMR (400 MHz, $CDCl_3$) δ 1.18 (d, 3H), 1.99 (m, 1H), 2.50 (m, 2H), 2.76 (m, 1H), 2.92 (m, 2H), 3.16 (m, 1H), 3.82 (m, 2H), 6.93 (m, 2H), 7.32 (m, 7H). LCMS (ESI): $[M]^+$ m/z: calcd 284.2; found 285.2; Rt = 0.949 min.

Rac-tert-butyl trans-4-benzyl-2-(4-fluorophenyl)-5-methyl-piperazine-1-carboxylate.
1-Benzyl-5-(4-fluorophenyl)-2-methyl-piperazine (24.94 g, 87.70 mmol) was dissolved in dioxane (250 mL) and sodium carbonate (18.59 g, 175.41 mmol, 7.35 mL) was added followed by water (250 mL). Di-*tert*-butyl dicarbonate (38.28 g, 175.41 mmol, 40.25 mL) was added dropwise to the resulting mixture and it was stirred overnight then concentrated under vacuum. The residue was diluted with water (300 mL) and the resulting mixture was extracted with EtOAc (2 x 300 mL). The combined organic layers were washed with brine (250 mL), dried over Na_2SO_4, filtered, and concentrated. The residue was purified by column chromatography, eluting with a mixture of hexane and EtOAc (15:1) to obtain *cis*- and *trans*- isomers in the order of elution –
Rac-tert-butyl *cis*-4-benzyl-2-(4-fluorophenyl)-5-methylpiperazine-1-carboxylate (4.5 g, 11.70 mmol, 13% yield, 84% purity);
mixture of 1:1 (7.79 g, 20.26 mmol, 23% yield);
Rac-tert-butyl *trans*-4-benzyl-2-(4-fluorophenyl)-5-methylpiperazine-1-carboxylate, less pure fraction 1 (3.31 g, 8.61 mmol, 10% yield, 93% purity);
Rac-tert-butyl *trans*-4-benzyl-2-(4-fluorophenyl)-5-methylpiperazine-1-carboxylate, more pure fraction 2 (5.27 g, 13.71 mmol, 16% yield, 99% purity). ^{1}H NMR (500 MHz, DMSO-d_6) δ 1.06 (d, 3H), 1.45 (s, 9H), 2.86 (m, 1H), 2.95 (m, 2H), 3.14 (m, 1H), 3.55 (m, 3H), 3.71 (m, 1H), 5.16 (m, 1H), 6.92 (m, 2H), 7.29 (m, 6H). LCMS (ESI): $[M]^+$ m/z: calcd 384.2; found 385.2; Rt = 1.287 min.

Rac-tert-butyl *trans*-2-(4-fluorophenyl)-5-methyl-piperazine-1-carboxylate, **Intermediate 23a**
Rac-tert-butyl *trans*-4-benzyl-2-(4-fluorophenyl)-5-methylpiperazine-1-carboxylate, fraction 2 (5.27 g, 13.72 mmol) was dissolved in MeOH (125 mL) and palladium, 10% on carbon, Type 487, dry (583.91 mg, 5.49 mmol) was added. The reaction mixture was evacuated and back filled three times with H_2 and stirred at 1 atm (balloon) overnight. The catalyst was filtered off and the filtrate was concentrated under vacuum to obtain *rac-tert*-butyl *trans*-2-(4-fluorophenyl)-5-methyl-piperazine-1-carboxylate, **Intermediate 23a** (4.12 g, crude). ^{1}H NMR (400 MHz, $CDCl_3$) δ 1.19 (d, 3H), 1.37 (s, 9H), 1.55 (m, 1H), 3.08 (m, 2H), 3.33 (m, 2H), 3.52 (m, 1H), 4.94 (m, 1H), 7.00 (m, 2H), 7.27 (m, 2H). LCMS (ESI): $[M]^+$ m/z: calcd 294.2; found 295.2; Rt = 1.079 min.

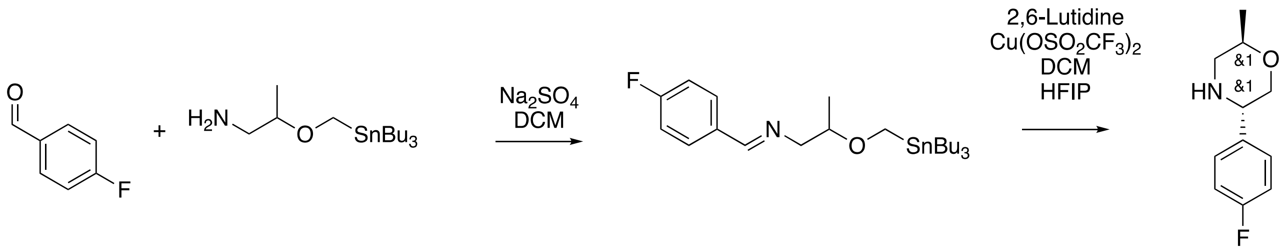

Intermediate 22a

(*E*)-1-(4-fluorophenyl)-*N*-(2-((tributylstannyl)methoxy)propyl)methanimine.
4-Fluorobenzaldehyde (1 g, 8.06 mmol, 862.07 μL) and 2-(tributylstannylmethoxy)propan-1-amine (3.05 g, 8.06 mmol) was dissolved in DCM (45 mL) then sodium sulfate, anhydrous (11.44 g, 80.57 mmol, 4.27 mL) was added and the reaction mixture was stirred overnight at 20 °C. The reaction mixture was filtered, the solid was washed with DCM, and filtrate was concentrated under vacuum to give (*E*)-1-(4-fluorophenyl)-*N*-[2-(tributylstannylmethoxy)propyl]methanimine (4.67 g, crude) which was used in the next step withour further purification. ^{1}H NMR (400 MHz, $CDCl_3$) δ 0.88 (m, 15H), 1.26 (m, 10H), 1.46 (m, 5H), 3.53 (m, 1H), 3.63 (m, 3H), 3.84 (m, 1H), 7.09 (m, 2H), 7.73 (m, 2H), 8.20 (m, 1H).

Rac-trans-5-(4-fluorophenyl)-2-methyl-morpholine, **Intermediate 22a**.
A flask was charged with copper (II) trifluoromethanesulfonate (3.49 g, 9.65 mmol) and DCM (50 mL), then HFIP (50 mL) was added via a syringe. 2,6-Lutidine, 98+% (1.03 g, 9.65 mmol, 1.12 mL) was added in one portion to the grey suspension affording a green suspension. This suspension was stirred at 20 °C for 1 h affording a more homogeneous dark green suspension. A solution of (*E*)-1-(4-fluorophenyl)-*N*-[2-(tributylstannylmethoxy)propyl]methanimine (4.67 g, 9.65 mmol) in DCM (15 mL) was added dropwise via syringe. The resulting reaction mixture was stirred at 20 °C for 12 h. The reaction mixture was quenched with a pre-mixed solution of 1:1 water-NH_4OH solution. The blue aqueous layer was separated and extracted with DCM. The combined organic layers were washed with a pre-mixed solution of 1:1 water-NH_4OH solution and brine , dried over Na_2SO_4, and concentrated by rotary evaporation. The crude product was purified by column chromatography (MeCN/MeOH from 100/0 to 0/100) to give *rac-trans*-5-(4-fluorophenyl)-2-methyl-morpholine (0.5 g, 2.56 mmol, 26.54% yield) as an orange gum. LCMS (ESI): [M]+ m/z: calcd 195.2; found 196.2; Rt = 0.597 min.

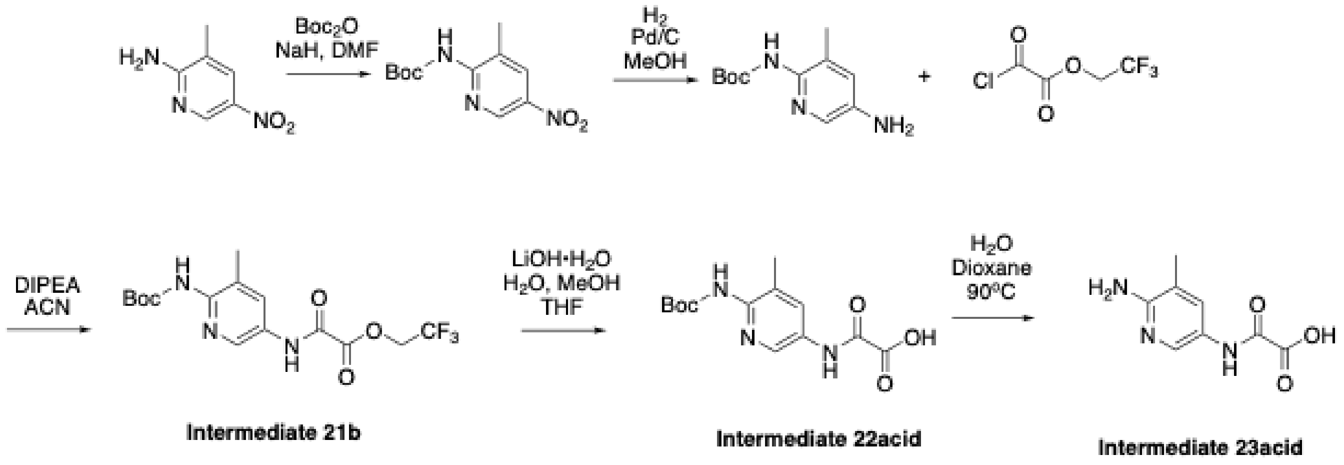

tert-butyl (3-methyl-5-nitropyridin-2-yl)carbamate.

To a solution of 3-methyl-5-nitro-pyridin-2-amine (60 g, 391.80 mmol) in DMF (525 mL) was added sodium hydride (60% dispersion in mineral oil, 16.51 g, 412.88 mmol) portionwise at 0 °C. The resulting mixture was stirred for 0.5 h (to the end of gas evolution) and a solution of di-*tert*-butyl dicarbonate (89.79 g, 411.39 mmol, 94.41 mL) in DMF (75 mL) was added dropwise. The resulting mixture was stirred at 25 °C for 18 h. The mixture was quenched with water (1000 mL), and the precipitate that formed was filtered off and dried in vacuo to obtain crude product (100 g). This material was purified by gradient column chromatography on silica gel ($CHCl_3$-MTBE as eluent) to obtain *tert*-butyl (3-methyl-5-nitropyridin-2-yl)carbamate (46 g, 181.64 mmol, 46% yield). ^{1}H NMR (400 MHz, DMSO-d_6) δ 1.49 (s, 9H), 2.32 (s, 3H), 8.37 (s, 1H), 8.97 (s, 1H), 9.59 (s, 1H). LCMS (ESI): [M-C(CH_3)$_3$+H]+ m/z: calcd 253.26; found 198.2; Rt = 1.272 min.

tert-butyl (5-amino-3-methylpyridin-2-yl)carbamate.

To a solution of *tert*-butyl (3-methyl-5-nitropyridin-2-yl)carbamate (46 g, 181.64 mmol) in MeOH (600 mL) was added palladium on activated carbon 10% (4.60 g, 43.22 mmol). The resulting mixture was stirred under H_2 atmosphere for 24 h. The catalyst was filtered and the solvent was evaporated in vacuo. The resultant residue was dissolved in DCM (500 ml), dried over Na_2SO_4, and evaporated in vacuo to obtain *tert*-butyl (5-amino-3-methylpyridin-2-yl)carbamate (38 g, 170.20 mmol, 94% yield). ^{1}H NMR (400 MHz, DMSO-d_6) δ 1.40 (s, 9H), 2.03 (s, 3H), 3.30 (brs, 2H), 6.80 (s, 1H), 7.54 (s, 1H), 8.52 (s, 1H). LCMS (ESI): [M+H]+ m/z: calcd 223.2; found 224.2; Rt = 0.67 min.

2,2,2-trifluoroethyl 2-((6-((*tert*-butoxycarbonyl)amino)-5-methylpyridin-3-yl)amino)-2-oxoacetate, **Intermediate 21b**.

To a solution of *tert*-butyl (5-amino-3-methylpyridin-2-yl)carbamate (17.6 g, 78.83 mmol) and DIPEA (15.28 g, 118.24 mmol, 20.60 mL) in ACN (250 mL) was added 2,2,2-trifluoroethyl 2-chloro-2-oxo-acetate (17.27 g, 90.65 mmol) dropwise at 0 °C under argon. The reaction mixture was then stirred for 24 h at room temperature, then evaporated in vacuo. The resultant residue was diluted with water (575 mL). The precipitate that formed was filtered off, washed with water, and dried in vacuo to afford the product 2,2,2-trifluoroethyl 2-((6-((*tert*-butoxycarbonyl)amino)-5-methylpyridin-3-yl)amino)-2-oxoacetate (30 g, crude). ^{1}H NMR (400 MHz, DMSO-d_6) δ 1.42 (s, 9H), 2.15 (s, 3H), 4.96 (q, 2H), 7.93 (s, 1H), 8.49 (s, 1H), 9.03 (s, 1H), 11.06 (s, 1H). LCMS (ESI): [M-C(CH_3)$_3$+H]+ m/z: calcd 377.32; found 322.0; Rt = 1.274 min.

2-((6-((*tert*-butoxycarbonyl)amino)-5-methylpyridin-3-yl)amino)-2-oxoacetic acid, **Intermediate 22acid**.

A mixture of 2,2,2-trifluoroethyl 2-((6-((*tert*-butoxycarbonyl)amino)-5-methylpyridin-3-yl)amino)-2-oxoacetate (30 g, 79.51 mmol) and lithium hydroxide, monohydrate (6.67 g, 159.02 mmol, 4.42 mL) in a mixture of THF (120 mL), methanol (120 mL), and water (120 mL) was stirred at 5 °C. After 2 h the volatile organic solvents were removed in vacuo. The resultant residue was acidified with sodium hydrogen sulfate, monohydrate (21.96 g, 159.02 mmol) to pH 5, and the precipitate that formed was filtered, washed with water, and dried in vacuo to afford the product 2-((6-((*tert*-butoxycarbonyl)amino)-5-methylpyridin-3-yl)amino)-2-oxoacetic acid, **Intermediate 22acid** (23 g, 77.89 mmol, 98% yield). ^{1}H NMR (400 MHz, DMSO-d_6) δ 1.44 (s, 9H), 2.16 (s, 3H), 7.97 (s, 1H), 8.51 (s, 1H), 8.98 (s, 1H), 10.70 (s, 1H) LCMS (ESI): [M-C(CH_3)$_3$+H]+ m/z: calcd 295.29; found 240.0; Rt = 0.829 min.

2-((6-amino-5-methylpyridin-3-yl)amino)-2-oxoacetic acid, **Intermediate 23acid**.

A suspension of **Intermediate 22acid** (2 g, 6.77 mmol) was stirred in a mixture of 1.4-dioxane (5 mL) and water (10 mL) at 90 °C for 3 h, then cooled, and concentrated in vacuo. The residue was diluted with water (10 mL), filtered, and the filtrate was acidified with concentrated aqueous hydrochloric acid to pH 5. The precipitate was filtered, washed with water (2 x 5 mL), and dried in vacuo at 60 °C to afford 2-((6-amino-5-methylpyridin-3-yl)amino)-2-oxoacetic acid, **Intermediate 23acid** (950 mg, 4.87 mmol, 72% yield) as light-yellow solid. ^{1}H NMR (400 MHz, DMSO-d_6) δ 2.01 (s, 3H), 5.43 (s, 2H), 7.59 (s, 1H), 8.11 (s, 1H), 9.78 (s, 1H). LCMS (ESI): [M+H]+ m/z: calcd 195.2; found 196.2; Rt = 0.211 min.

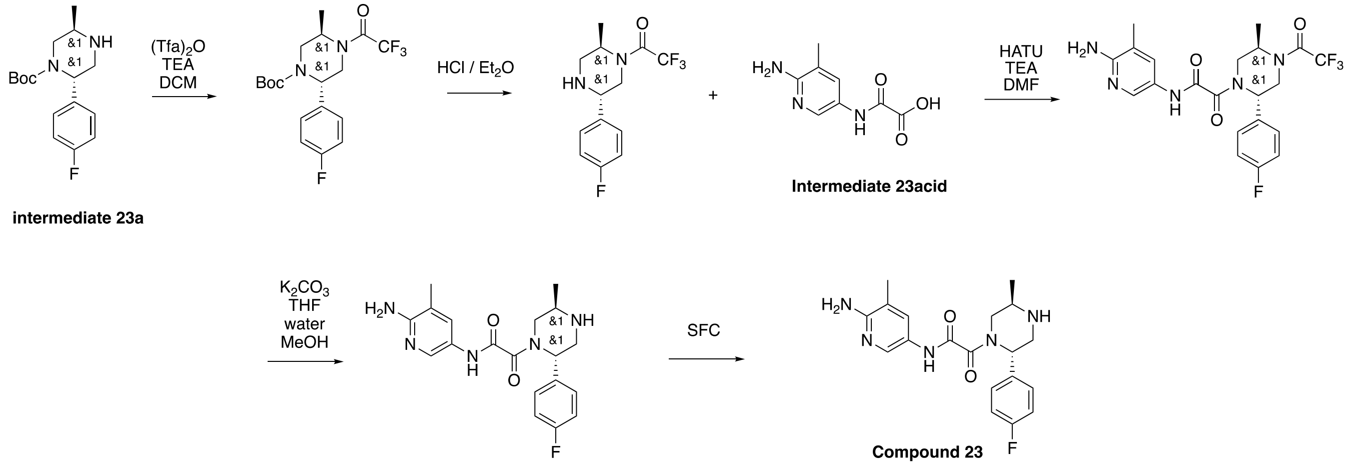

rac-tert-butyl *trans*-2-(4-fluorophenyl)-5-methyl-4-(2,2,2-trifluoroacetyl)piperazine-1-carboxylate.
Intermediate 23a (0.4 g, 1.36 mmol) was dissolved in DCM (1 mL) and TEA (165.00 mg, 1.63 mmol, 227.28 µL) was added in one portion. The reaction mixture was cooled to 0 °C, and trifluoroacetic anhydride (285.40 mg, 1.36 mmol, 191.55 µL) in DCM (1 mL) was slowly added to the mixture. The reaction mixture was stirred at room temperature for 3 h. The mixture was diluted with H_2O (40 mL) and stirred for 30 min at room temperature, then the organic phase was dried with Na_2SO_4 and concentrated. The crude material was used without further purification. LCMS (ESI): [M-Boc]+ m/z: calcd 290.2; found 291.2; Rt = 1.570 min

rac-2,2,2-trifluoro-1-(*trans*-5-(4-fluorophenyl)-2-methylpiperazin-1-yl)ethan-1-one.
A solution of *rac-tert*-butyl *trans*-2-(4-fluorophenyl)-5-methyl-4-(2,2,2-trifluoroacetyl)piperazine-1-carboxylate (503 mg, 1.29 mmol) in Et_2O/HCl (3.5 mL) was stirred at room temperature for 12 h. The resulting solid was collected by filtration and washed with MTBE (2 x 20 mL) to obtain *rac*-2,2,2-trifluoro-1-(*trans*-5-(4-fluorophenyl)-2-methylpiperazin-1-yl)ethan-1-one (310 mg, crude, HCl). LCMS (ESI): [M]+ m/z: calcd 290.2; found 291.2; Rt = 0.919 min.

rac-N-(6-amino-5-methylpyridin-3-yl)-2-(*trans*-2-(4-fluorophenyl)-5-methyl-4-(2,2,2-trifluoroacetyl)piperazin-1-yl)-2-oxoacetamide.
rac-2,2,2-trifluoro-1-(*trans*-5-(4-fluorophenyl)-2-methylpiperazin-1-yl)ethan-1-one (1 eq), **Intermediate 23acid** (1 eq), and TEA (3.5 eq) were stirred in DMF. HATU (1.5 eq) was added and the resulting mixture was stirred overnight. The reaction mixture was concentrated in vacuum and the residue was purified by HPLC to obtain *rac-N*-(6-amino-5-methylpyridin-3-yl)-2-(*trans*-2-(4-fluorophenyl)-5-methyl-4-(2,2,2-trifluoroacetyl)piperazin-1-yl)-2-oxoacetamide as a white solid; LCMS (ESI): [M]+ m/z: calcd 467.2; found 468.2; Rt = 1.113 min.

rac-N-(6-amino-5-methylpyridin-3-yl)-2-(*trans*-2-(4-fluorophenyl)-5-methylpiperazin-1-yl)-2-oxoacetamide.
rac-N-(6-amino-5-methylpyridin-3-yl)-2-(*trans*-2-(4-fluorophenyl)-5-methyl-4-(2,2,2-trifluoroacetyl)piperazin-1-yl)-2-oxoacetamide (650 mg, 1.39 mmol) was dissolved in THF (4 mL)/MeOH (2 mL). Potassium carbonate-granular (576.58 mg, 4.17 mmol) was dissolved in water (2 mL) and added one portion to the reaction mixture. The mixture was stirred at 26 °C for 12 h. EtOAc (50 mL) was added in one portion to the mixture, then the combined organic phases were washed with aqueous NaCl (3 x 15 mL), dried with Na_2SO_4, and concentrated. The crude material (0.8 g) was evaporated with dioxane / HCl (25 mL) and purified by HPLC (2 - 7 min 10 – 30 % MeCN - water, 30 mL / min, column: SunFire C18, 100 x 19 mm, 5 µM loading pump: 4 mL / min MeCN) to afford *rac-N*-(6-amino-5-methylpyridin-3-yl)-2-(*trans*-2-(4-fluorophenyl)-5-methylpiperazin-1-yl)-2-oxoacetamide (39.4 mg, 106.08 µmol, 8% yield). LCMS (ESI): [M]+ m/z: calcd 371.2; found 372.2; Rt = 0.733 min.

N-(6-amino-5-methylpyridin-3-yl)-2-((2*S*,5*R*)-2-(4-fluorophenyl)-5-methylpiperazin-1-yl)-2-oxoacetamide, **23**.
The mixture of diastereomers was separated by chiral chromatography (Column: Chiralpak AD-H-III (250 x 20 mm , 5 µm); Mobile phase : Hexane-IPA-MeOH 50-25-25 Flow Rate: 12 mL / min;) to obtain *N*-(6-amino-5-methyl-3-pyridyl)-2-[(2*S*,5*R*)-2-(4-fluorophenyl)-5-methyl-piperazin-1-yl]-2-oxo-acetamide, **23** (6.7 mg, 18.04 µmol, 17% yield) (Rt = 94.47 min) and *N*-(6-amino-5-methyl-3-pyridyl)-2-[(2*R*,5*S*)-2-(4-fluorophenyl)-5-methyl-piperazin-1-yl]-2-oxo-acetamide (7.38 mg, 19.87 µmol, 19% yield) (RT = 71.15 min).
Rel Time for *N*-(6-amino-5-methyl-3-pyridyl)-2-[(2*R*,5*S*)-2-(4-fluorophenyl)-5-methyl-piperazin-1-yl]-2-oxo-acetamide in analytical conditions (column: IC, Hexane-IPA-MeOH, 75-15-15, 0.6 mL / min as mobile phase) 33.05 min and **Compound 23** = 38.47 min.
N-(6-amino-5-methyl-3-pyridyl)-2-[(2*R*,5*S*)-2-(4-fluorophenyl)-5-methyl-piperazin-1-yl]-2-oxo-acetamide: ^{1}H NMR (600 MHz, DMSO-d_6) δ 1.15 – 1.21 (m, 3H), 1.98 – 2.03 (m, 3H), 2.90 – 3.27 (m, 3H), 3.35 – 3.42 (m, 2H), 3.51 – 3.94 (m, 1H), 5.07 – 5.48 (m, 1H), 5.58 – 5.67 (m, 2H), 7.14 – 7.25 (m, 2H), 7.39 – 7.52 (m, 3H), 7.91 – 8.08 (m, 1H), 10.42 – 10.57 (m, 1H). LCMS (ESI): [M]+ m/z: calcd 371.2; found 372.2; Rt = 1.169 min. **Compound 23**: ^{1}H NMR (600 MHz, DMSO-d_6) δ 1.11 – 1.18 (m, 3H), 1.98 – 2.04 (m, 3H), 2.90 – 3.21 (m, 2H), 3.34 – 3.45 (m, 3H), 3.48 – 3.92 (m, 1H), 4.97 – 5.44 (m, 1H), 5.56 – 5.70 (m, 2H), 7.16 – 7.24 (m, 2H), 7.43 – 7.51 (m, 3H), 7.91 – 8.02 (m, 1H), 10.41 – 10.55 (m, 1H). LCMS (ESI): [M]+ m/z: calcd 371.2; found 372.2; Rt = 1.115 min.

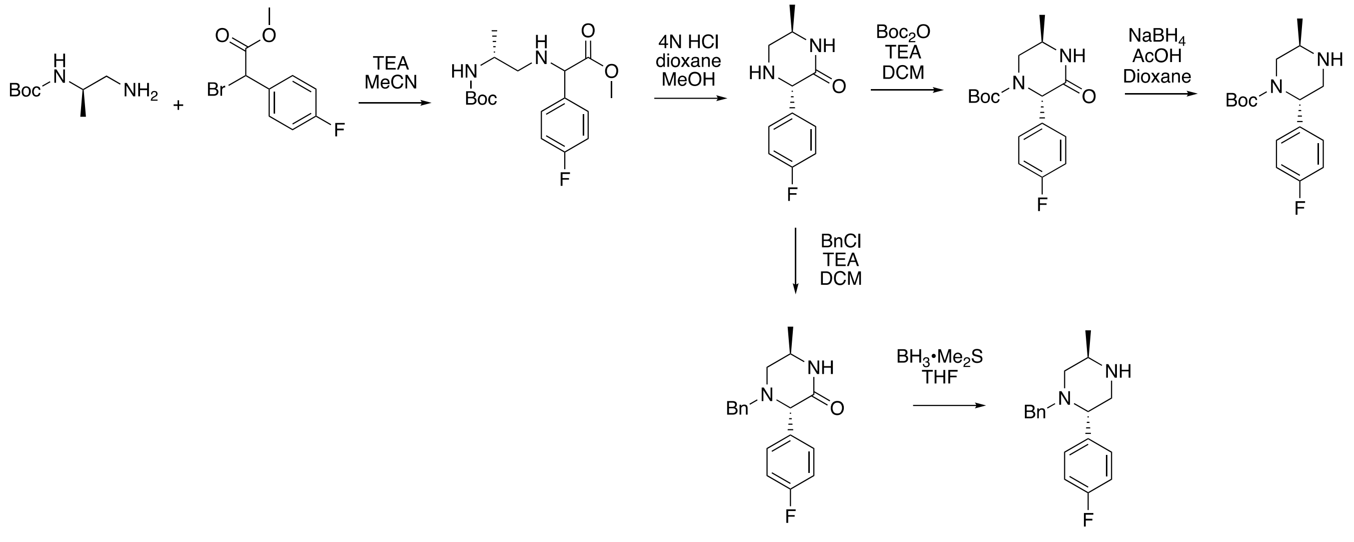

Intermediate 31a

Methyl 2-(((*R*)-2-((*tert*-butoxycarbonyl)amino)propyl)amino)-2-(4-fluorophenyl)acetate.
Methyl 2-bromo-2-(4-fluorophenyl)acetate (1 eq) was added dropwise to a solution of *tert*-butyl *N*-[(1*R*)-2-amino-1-methyl-ethyl]carbamate (1 eq) and TEA (2 eq) in MeCN. The resulting mixture was stirred at 20 °C for 14 h at which point the volatiles were removed under reduced pressure and the residue was partitioned between 10% aq. K_2CO_3 solution and MTBE. The organic layer was separated, dried over Na_2SO_4 and concentrated under reduced pressure to obtain methyl 2-(((*R*)-2-((*tert*-butoxycarbonyl)amino)propyl)amino)-2-(4-fluorophenyl)acetate which was used further without purification. LCMS (ESI): [M]+ m/z: calcd 340.2; found 341.2; Rt = 1.036 min.

(3*S*,6*R*)-3-(4-fluorophenyl)-6-methylpiperazin-2-one.
4.0 M hydrogen chloride solution in dioxane (4 eq) was added portion wise to a solution of methyl 2-(((*R*)-2-((*tert*-butoxycarbonyl)amino)propyl)amino)-2-(4-fluorophenyl)acetate (1 eq) in MeOH. The resulting mixture was stirred at 20 °C for 14 h after which the volatiles were removed under reduced pressure and the residue was partitioned between 25 % aq. K_2CO_3 solution and DCM. The organic layer was separated and the aqueous layer was extracted with DCM twice. The combined DCM layers were dried over Na_2SO_4 and concentrated under vacuum. After the solvent was removed, the residue was heated to 80 °C under reduced pressure (approx. 15 torr) for 1 h, then it was dissolved in boiling toluene. The resulting solution was left at 20 °C for 6 h. The obtained white crystals were filtered and dried, affording (3*S*,6*R*)-3-(4-fluorophenyl)-6-methylpiperazin-2-one. Yield: 31%. LCMS (ESI): [M]+ m/z: calcd 208.2; found 209.2; Rt = 0.238 min.

tert-butyl (2*S*,5*R*)-2-(4-fluorophenyl)-5-methyl-3-oxopiperazine-1-carboxylate.
Boc_2O (1.15 eq) was added dropwise to the solution of (3*S*,6*R*)-3-(4-fluorophenyl)-6-methylpiperazin-2-one (1.0 eq) and TEA (1.2 eq) in DCM. The mixture was stirred at 20 °C for 4 h after which the volatiles were removed under reduced pressure and the residue was triturated with cold hexane (60 mL). The resultant precipitate was filtered and dried, affording *tert*-butyl (2*S*,5*R*)-2-(4-fluorophenyl)-5-methyl-3-oxopiperazine-1-carboxylate. Yield: 99%. LCMS (ESI): [M-t-Bu]+ m/z: calcd 252.2; found 253.2; Rt = 1.183 min.

tert-butyl (2*S*,5*R*)-2-(4-fluorophenyl)-5-methylpiperazine-1-carboxylate.
A solution of *tert*-butyl (2*S*,5*R*)-2-(4-fluorophenyl)-5-methyl-3-oxopiperazine-1-carboxylate (1 eq) and acetic acid (10 eq) in dioxane was added dropwise to a refluxed solution of $NaBH_4$ (10 eq) in dioxane. The reaction mixture was refluxed for 3 h after which it was cooled to room temperature, quenched with water, and extracted with EtOAc. The organic layer was separated, dried over Na_2SO_4, and evaporated to give the crude which was purified by flash chromatography (MTBE/MeOH (0~100%) as an eluent mixture) to afford *tert*-butyl (2*S*,5*R*)-2-(4-fluorophenyl)-5-methylpiperazine-1-carboxylate. Yield: 27%. LCMS (ESI): [M]+ m/z: calcd 294.2; found 295.2; Rt = 1.024 min.

(3*S*,6*R*)-4-benzyl-3-(4-fluorophenyl)-6-methylpiperazin-2-one.
Benzyl chloride (1.10 g, 7.84 mmol) was added dropwise to a solution of (3*S*,6*R*)-3-(4-fluorophenyl)-6-methylpiperazin-2-one (2.1 g, 6.54 mmol, TFA) and TEA (1.98 g, 19.61 mmol, 2.73 mL) in DCM (30 mL). The resulting mixture was stirred at 20 °C for 2 h after which 20% aq. K_2CO_3 solution (20 mL) was added and stirring was continued for 10 min then the organic layer was separated, dried over K_2CO_3, and concentrated under reduced pressure, affording (3*S*,6*R*)-4-benzyl-3-(4-fluorophenyl)-6-methylpiperazin-2-one (2.33 g, crude). LCMS (ESI): [M]+ m/z: calcd 312.2; found 313.2; Rt = 1.047 min.

(2*S*,5*R*)-1-benzyl-2-(4-fluorophenyl)-5-methylpiperazine, **Intermediate 31a**.
Borane dimethyl sulfide complex (2.66 g, 35.06 mmol, 3.33 mL) was added dropwise to a solution of (3*S*,6*R*)-4-benzyl-3-(4-fluorophenyl)-6-methylpiperazin-2-one (2.19 g, 7.01 mmol) in THF (30 mL). The resultant mixture was stirred at 65 °C for 18 h after which it was cooled to room temperature and the excess borane was destroyed by dropwise addition of MeOH (10 mL). After H_2 evolution ceased, the volatiles were removed under reduced pressure and the residue was taken up in 2 M aq. HCl (40 mL) and stirred at 50 °C for 40 minutes. The resultant cloudy solution was filtered and extracted with DCM (2 x 10 mL). The DCM layers were discarded and the aqueous layer was basified to pH ≈ 11 with solid potassium hydroxide. The precipitated amine was extracted with DCM (2 x 25 mL). The organic layers were separated, dried over K_2CO_3, and concentrated under vacuum, affording (2*S*,5*R*)-1-benzyl-2-(4-fluorophenyl)-5-methylpiperazine, **Intermediate 31a** (1.31 g, 4.61 mmol, 66% yield). LCMS (ESI): [M]+ m/z: calcd 284.2; found 285.2; Rt = 1.049 min.

General Procedure A1

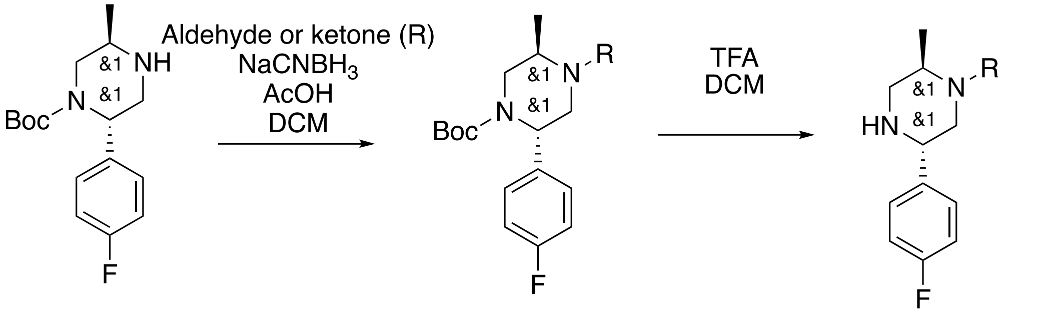

intermediate 23a

Intermediate Xa

Intermediate 23a (1 eq) was dissolved in DCM and R-aldehyde or R-ketone (1.5 eq) was added followed by addition of AcOH (1 eq). Sodium cyanoborohydride (2 eq) was then added and the resulting mixture was stirred overnight. Saturated $NaHCO_3$ aq. solution was added to the reaction mixture and the resulting mixture was extracted with DCM twice. The combined organic layers were dried over Na_2SO_4, filtered, and evaporated to obtain desired Boc-alkylated piperazine (crude). Boc-alkylated piperazine (1 eq) was dissolved in DCM and TFA (15 eq) was added. The resulting mixture was stirred at 25 °C for 1 h, and then evaporated under vacuum. Crushed ice (15 g) was added to the residue and pH was adjusted to 10 with a 7 M aqueous solution of K_2CO_3. The resulting mixture was extracted with DCM. The combined organic extracts were dried over sodium sulfate and evaporated in vacuum to afford **Intermediate Xa**.

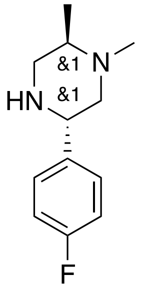

Intermediate 24a

rac-trans-5-(4-fluorophenyl)-1,2-dimethylpiperazine, **Intermediate 24a**.
Aldehyde = formaldehyde. No AcOH used.
Boc protected: LCMS (ESI): [M]+ m/z: calcd 308.2; found 309.2; Rt = 1.028 min.
Intermediate 24a: LCMS (ESI): [M]+ m/z: calcd 208.2; found 209.2; Rt = 0.466 min.

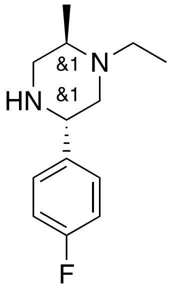

Intermediate 25a

rac-trans-1-ethyl-5-(4-fluorophenyl)-2-methylpiperazine, **Intermediate 25a**.
Aldehyde = CH_3CHO.

Boc protected: LCMS (ESI): [M]+ m/z: calcd 322.2; found 323.2; Rt = 0.923 min.
Intermediate 25a: Yield: 49.7 mg (2HCl; 12%).
HPLC conditions: Column: SunFire 100 x 19 mm, 5 μM; 2 - 10 min 0 - 50% MeOH – water + HCl 30 mL / min (loading pump 4 mL MeOH).
LCMS (ESI): [M]+ m/z: calcd 222.2; found 223.2; Rt = 0.411 min.

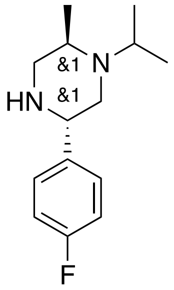

Intermediate 27a

rac-trans-5-(4-fluorophenyl)-1-isopropyl-2-methylpiperazine, **Intermediate 27a**.
Ketone: acetone.
Boc protected: LCMS (ESI): [M]+ m/z: calcd 336.2; found 337.2; Rt = 1.077 min.
Intermediate 27a: LCMS (ESI): [M]+ m/z: calcd 236.2; found 237.2; Rt = 0.663 min.

General Procedure A2

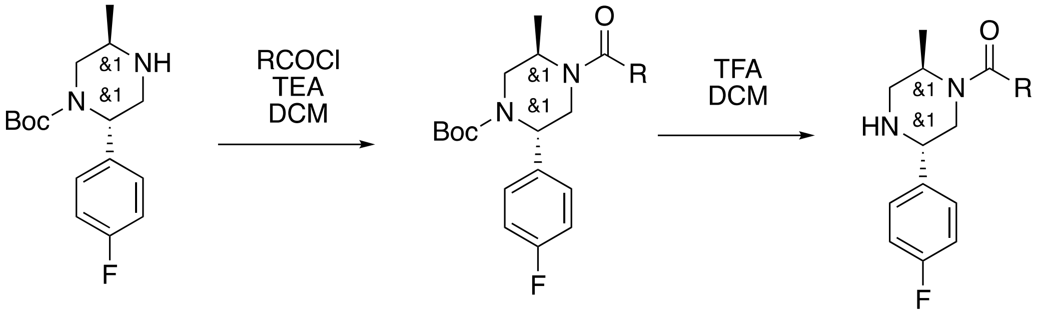

intermediate 23a

Intermediate Xb

Intermediate 23a (1 eq) and TEA (3 eq) were stirred in DCM and the resulting solution was cooled to -5 °C in an ice / MeOH bath. R-acid chloride (1.3 eq) was added dropwise to the previous solution and the resulting mixture was allowed to warm to room temperature and stirred overnight. The reaction mixture was diluted with DCM and the resulting solution was washed with water twice, dried over Na_2SO_4, filtered, and evaporated to obtain the desired Boc-acylated piperazine. Boc-acylated piperazine (1 eq) was dissolved in DCM and TFA (15 eq) was added. The resulting mixture was stirred at 25 °C for 1 h, and then evaporated under vacuum. Crushed ice (15 g) was added to the residue and pH was adjusted to 10 with a 7 M aqueous solution of K_2CO_3. The resulting mixture was extracted with DCM. The combined organic extracts were dried over sodium sulfate and evaporated in vacuum to afford **Intermediate Xb**.

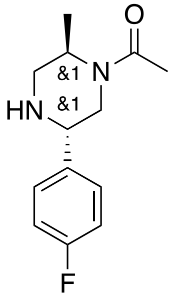

Intermediate 26b

rac-1-(*trans*-5-(4-fluorophenyl)-2-methylpiperazin-1-yl)ethan-1-one, **Intermediate 26b**.

Acid chloride: Acetyl chloride.
Boc intermediate: LCMS (ESI): [M-Boc]+ m/z: calcd 236.2; found 237.2; Rt = 1.364 min.
Intermediate 26b: Yield: 64%. LCMS (ESI): [M]+ m/z: calcd 236.2; found 237.2; Rt = 0.638 min.

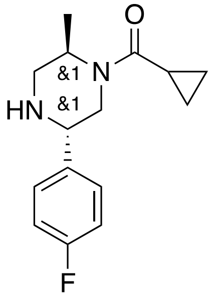

Intermediate 29b

rac-cyclopropyl(*trans*-5-(4-fluorophenyl)-2-methylpiperazin-1-yl)methanone, **Intermediate 29b**.
Acid chloride: cyclopropanecarbonyl chloride.
Boc intermediate: Yield: 96%. LCMS (ESI): [M-t-Bu]+ m/z: calcd 306.2; found 307.2; Rt = 1.480 min.
Intermediate 29b: LCMS (ESI): [M]+ m/z: calcd 262.2; found 263.2; Rt = 0.649 min.

General Procedure A3

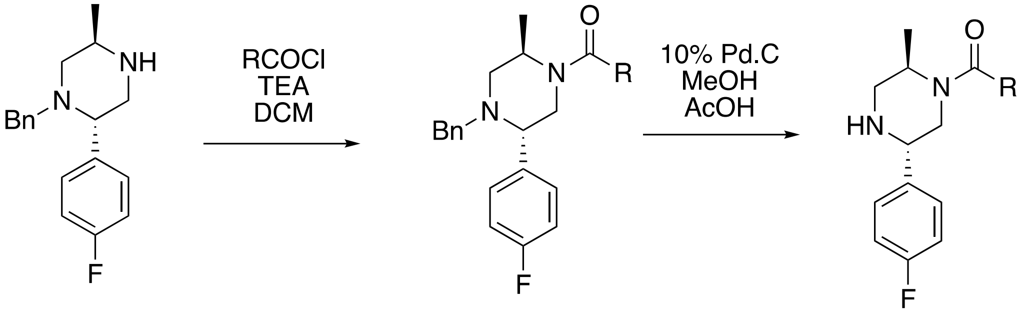

intermediate 31a

Intermediate Xb

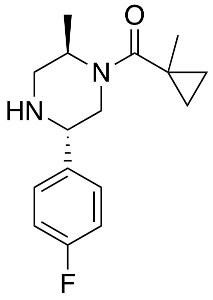

Intermediate 31b

((2*R*,5*S*)-5-(4-fluorophenyl)-2-methylpiperazin-1-yl)(1-methylcyclopropyl)methanone, **Intermediate 31b**.
Acid chloride: 1-methylcyclopropane-1-carbonyl chloride.
Benzyl intermediate: LCMS (ESI): [M]+ m/z: calcd 366.2; found 367.2; Rt = 1.372 min.
Intermediate 31b: Yield: 94%. LCMS (ESI): [M]+ m/z: calcd 276.2; found 277.2; Rt = 0.702 min.

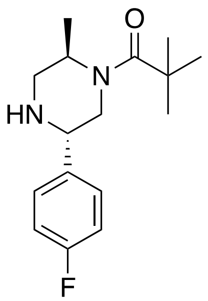

Intermediate 32b

1-((2*R*,5*S*)-5-(4-fluorophenyl)-2-methylpiperazin-1-yl)-2,2-dimethylpropan-1-one, **Intermediate 32b**.
Acid chloride: 2,2-dimethylpropanoyl chloride.
Benzyl intermediate: LCMS (ESI): [M]+ m/z: calcd 368.2; found 369.2; Rt = 1.589 min.
Intermediate 32b: Yield: 96%. LCMS (ESI): [M]+ m/z: calcd 278.2; found 279.2; Rt = 0.928 min.

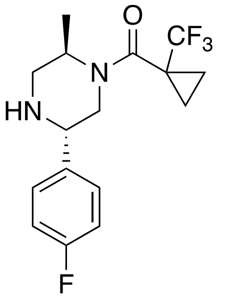

Intermediate 33b

((2*R*,5*S*)-5-(4-fluorophenyl)-2-methylpiperazin-1-yl)(1-(trifluoromethyl)cyclopropyl)methanone, **Intermediate 33b**.
Acid chloride: 1-(trifluoromethyl)cyclopropanecarbonyl chloride.
Benzyl intermediate: LCMS (ESI): [M]+ m/z: calcd 420.2; found 421.2; Rt = 1.606 min.
Intermediate 33b: Yield: 98%. LCMS (ESI): [M]+ m/z: calcd 330.2; found 331.2; Rt = 0.789 min.

General Procedure A4

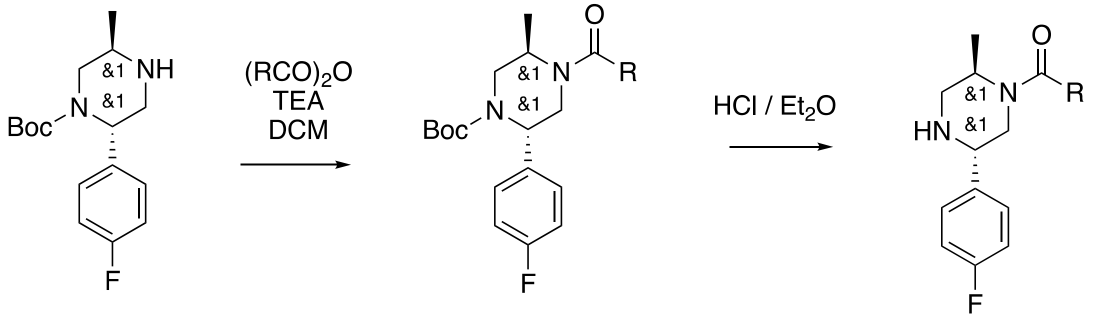

Intermediate 23a **Intermediate Xc**

Intermediate 23a (1 eq) and TEA (3 eq) were mixed together in DCM and the resulting solution was cooled to -5°C in an ice/MeOH bath. $(RCO)_2O$ (1 eq) was added dropwise and the resulting mixture was allowed to warm to room temperature and stirred overnight. The reaction mixture was diluted with DCM and the resulting solution was washed with water twice, dried over Na_2SO_4, filtered, and evaporated to obtain **Intermediate Xc**.

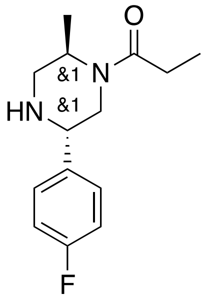

Intermediate 28c

rac-1-(*trans*-5-(4-fluorophenyl)-2-methylpiperazin-1-yl)propan-1-one, **Intermediate 28c**.
Anhydride: propionic anhydride.
Boc intermediate: LCMS (ESI): [M-Boc]+ m/z: calcd 250.2; found 251.2; Rt = 1.448 min.
Intermediate 28c: LCMS (ESI): [M]+ m/z: calcd 250.2; found 251.2; Rt = 0.751 min.

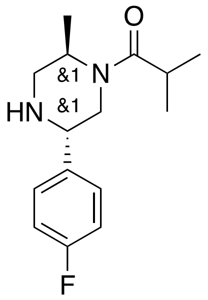

Intermediate 30c

rac-1-(*trans*-5-(4-fluorophenyl)-2-methylpiperazin-1-yl)-2-methylpropan-1-one, **Intermediate 30c**.
Anhydride: isobutyric anhydride.
Boc intermediate: Yield: 99%. ^{1}H NMR (400 MHz, $CDCl_3$) δ 0.93 (d, 3H), 1.11 (m, 3H), 1.28 (m, 2H), 1.50 (s, 9H), 2.78 (m, 2H), 3.24 (m, 1H), 3.89 (m, 3H), 4.92 (m, 1H), 5.22 (m, 1H), 6.97 (m, 2H), 7.22 (m, 2H). LCMS (ESI): [M-Boc]+ m/z: calcd 264.2; found 265.2; Rt = 1.492 min.
Intermediate 30c: Yield: 89%. ^{1}H NMR (500 MHz, $CDCl_3$) δ 1.06 (d, 6H), 1.26 (m, 2H), 1.33 (d, 3H), 1.83 (m, 1H), 2.62 (m, 2H), 3.00 (m, 1H), 3.42 (m, 1H), 4.15 (m, 1H), 6.99 (m, 2H), 7.35 (m, 2H). LCMS (ESI): [M]+ m/z: calcd 264.2; found 265.2; Rt = 0.840 min.

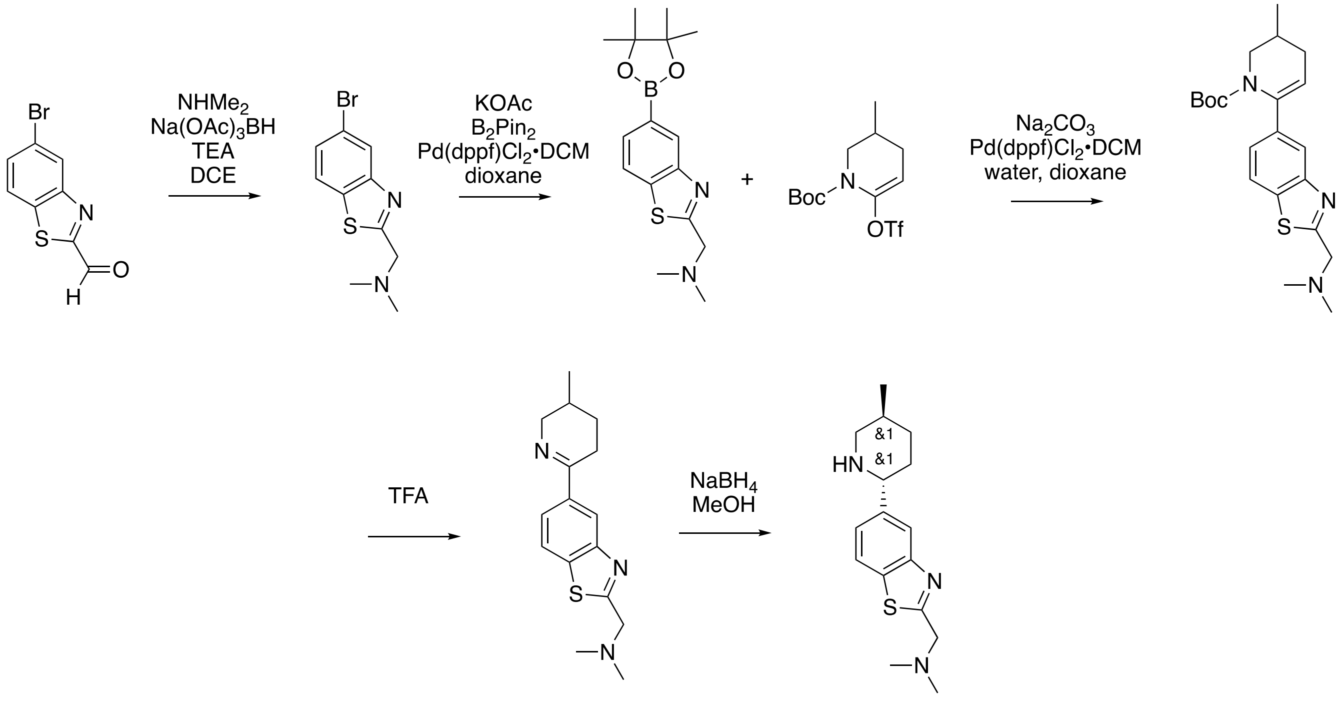

Intermediate 49a

1-(5-bromobenzo[*d*]thiazol-2-yl)-*N,N*-dimethylmethanamine.
To the stirred solution of 5-bromobenzo[*d*]thiazole-2-carbaldehyde (1eq) in 1,2-dichloroethane, dimethylamine (2 eq) was added and allowed to stir at 25 °C for 2 h, sodium(trisacetoxy)borohydride (2 eq) was added. The reaction mixture was stirred at 25 °C for 16 h. After completion, the reaction mixture was evaporated, quenched with water, and neutralized by K_2CO_3 to pH = 10. The aqueous phase was extracted with $CHCl_3$ twice. The combined organic phase was dried over Na_2SO_4 and evaporated under reduced pressure to afford 1-(5-bromobenzo[*d*]thiazol-2-yl)-*N,N*-dimethylmethanamine. Yield: 92%. LCMS (ESI): [M]+ m/z: calcd 271.2; found 272.2; Rt = 0.742 min.

N,N-dimethyl-1-(5-(4,4,5,5-tetramethyl-1,3,2-dioxaborolan-2-yl)benzo[*d*]thiazol-2-yl)methanamine.
1-(5-bromobenzo[*d*]thiazol-2-yl)-*N,N*-dimethylmethanamine (1 eq), B_2Pin_2 (1.1 eq) and KOAc (2 eq) were mixed in dioxane. The resulting mixture was evacuated and then backfilled with Ar, this operation was repeated three times, then Pd(dppf)Cl_2•DCM (0.05 eq) was added under Ar. The reaction mixture was stirred under argon at 90 °C for 14 h, then cooled and filtered. The filter cake was washed with dioxane twice. The solvent was evaporated to afford *N,N*-dimethyl-1-(5-(4,4,5,5-tetramethyl-1,3,2-dioxaborolan-2-yl)benzo[*d*]thiazol-2-yl)methanamine. LCMS (ESI): [M]+ m/z: calcd 318.2; found 319.2; Rt = 1.074 min.

tert-butyl 6-(2-((dimethylamino)methyl)benzo[*d*]thiazol-5-yl)-3-methyl-3,4-dihydropyridine-1(2*H*)-carboxylate.
N,N-dimethyl-1-(5-(4,4,5,5-tetramethyl-1,3,2-dioxaborolan-2-yl)benzo[*d*]thiazol-2-yl)methanamine (1 eq), *tert*-butyl 3-methyl-6-(trifluoromethylsulfonyloxy)-3,4-dihydro-2*H*-pyridine-1-carboxylate (1.2 eq) , sodium carbonate (3 eq) were mixed together in dioxane-water mixture (3:1). The resulting mixture was evacuated and then backfilled with Ar. This operation was repeated two times, then Pd(dppf)Cl_2•DCM (819.86 mg, 1.00 mmol) was added and the reaction mixture was stirred under Ar at 90 °C overnight, then cooled down and concentrated under vacuum. The residue was diluted with MTBE and stirred for 0.5 h. Anhydrous sodium sulfate was added and the resulting mixture was filtered. The filter cake was then washed with MTBE (5 x 50 mL). The filtrate was concentrated under vacuum to afford *tert*-butyl 6-(2-((dimethylamino)methyl)benzo[*d*]thiazol-5-yl)-3-methyl-3,4-dihydropyridine-1(2*H*)-carboxylate. LCMS (ESI): [M]+ m/z: calcd 387.2; found 388.2; Rt = 1.052 min.

N,N-dimethyl-1-(5-(5-methyl-3,4,5,6-tetrahydropyridin-2-yl)benzo[*d*]thiazol-2-yl)methanamine.
A solution of *tert*-butyl 6-(2-((dimethylamino)methyl)benzo[*d*]thiazol-5-yl)-3-methyl-3,4-dihydropyridine-1(2*H*)-carboxylate (1 eq) in TFA (15 eq) was stirred at room temperature for 1 h and then concentrated under vacuum. Cold water was added to the residue and the resulting mixture was extracted with DCM twice. The DCM layer was discarded, and the aqueous layer was basified to pH 11. The resulting mixture was extracted with DCM twice. The combined organic extracts were dried over sodium sulfate and concentrated under vacuum to afford *N,N*-dimethyl-1-(5-(5-methyl-3,4,5,6-tetrahydropyridin-2-yl)benzo[*d*]thiazol-2-yl)methanamine. LCMS (ESI): [M]+ m/z: calcd 287.2; found 288.2; Rt = 0.607 min.

rac-*N,N*-dimethyl-1-(5-(*trans*-5-methylpiperidin-2-yl)benzo[*d*]thiazol-2-yl)methanamine, **Intermediate 49a**.
N,N-dimethyl-1-(5-(5-methyl-3,4,5,6-tetrahydropyridin-2-yl)benzo[*d*]thiazol-2-yl)methanamine (1 eq) was dissolved in MeOH and the resulting solution was cooled to 0 °C in an ice bath. Sodium borohydride (2 eq) was added portion wise and the reaction mixture was allowed to warm to room temperature and stirred overnight. Water was added to the reaction mixture and the resulting mixture was concentrated in vacuum. The residue was diluted with water and the resulting mixture was extracted with DCM twice, dried over Na_2SO_4, filtered, and evaporated to obtain *rac*-*N,N*-dimethyl-1-(5-(*trans*-5-methylpiperidin-2-yl)benzo[*d*]thiazol-2-yl)methanamine, **Intermediate 49a**. LCMS (ESI): [M]+ m/z: calcd 289.2; found 290.2; Rt = 0.664 min.

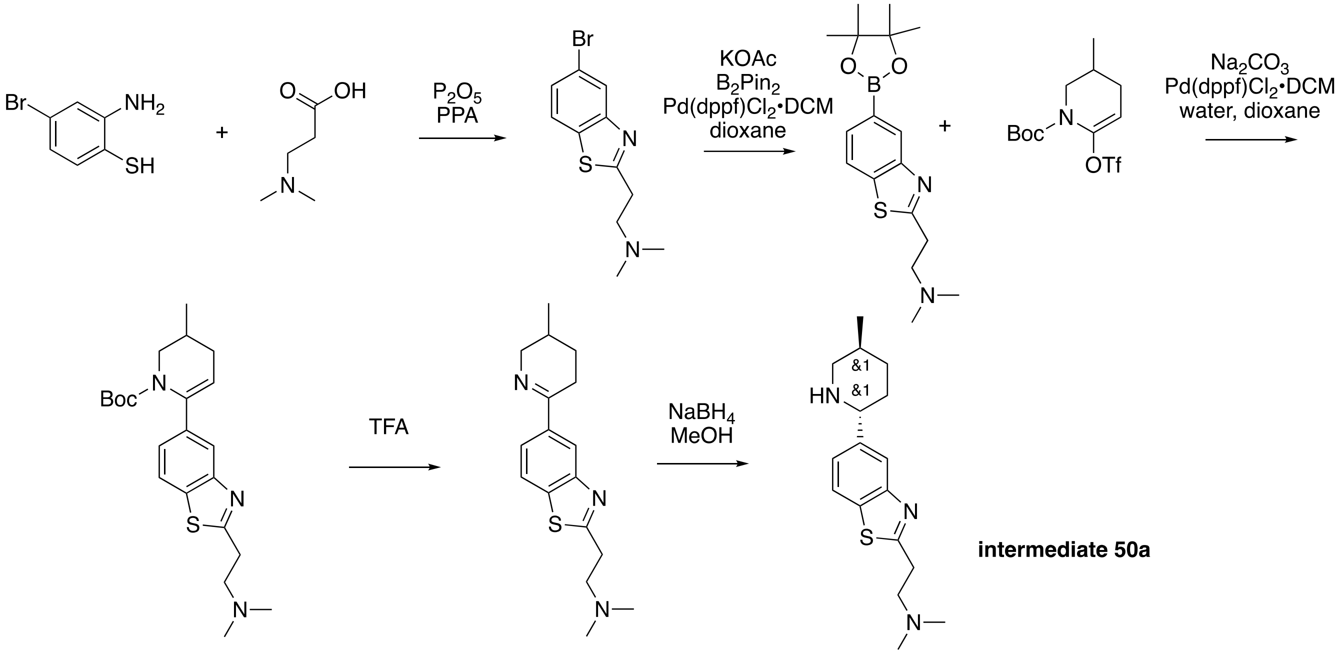

intermediate 50a

2-(5-bromobenzo[*d*]thiazol-2-yl)-*N,N*-dimethylethan-1-amine.
Phosphoric acid (4 eq) and phosphorus pentoxide (4 eq) were mixed together. The reaction suspension was stirred at room temperature for 10 min, then 2-amino-4-bromobenzenethiol (1 eq) followed by 3-(dimethylamino)propanoic acid (1.2 eq) were added under Ar. The solution was stirred at 110 °C for 18 h then it was triturated with water, basified (NaOH, 10% aq.) to pH = 10, extracted with DCM twice, dried and evaporated in vacuum to give crude product which was purified by silica gel with $CHCl_3$/MeOH as an eluent mixture. 2-(5-bromobenzo[*d*]thiazol-2-yl)-*N,N*-dimethylethan-1-amine. Yield: 11%. LCMS (ESI): [M]+ m/z: calcd 285.2; found 286.2; Rt = 0.824 min.

N,N-dimethyl-2-(5-(4,4,5,5-tetramethyl-1,3,2-dioxaborolan-2-yl)benzo[*d*]thiazol-2-yl)ethan-1-amine.
2-(5-bromobenzo[*d*]thiazol-2-yl)-*N,N*-dimethylethan-1-amine (1 eq), B_2Pin_2 (1.1 eq) and KOAc (2 eq) were mixed in dioxane. The resulting mixture was evacuated and then backfilled with Ar. This operation was repeated three times, then Pd(dppf)Cl_2•DCM (0.05 eq) was added under Ar. The reaction mixture was stirred under argon at 90 °C for 14 h, then cooled and filtered. The filter cake was washed with dioxane twice. The solvent was evaporated to afford *N,N*-dimethyl-2-(5-(4,4,5,5-tetramethyl-1,3,2-dioxaborolan-2-yl)benzo[*d*]thiazol-2-yl)ethan-1-amine. LCMS (ESI): [M]+ m/z: calcd 332.2; found 333.2; Rt = 1.004 min.

tert-butyl 6-(2-(2-(dimethylamino)ethyl)benzo[*d*]thiazol-5-yl)-3-methyl-3,4-dihydropyridine-1(2*H*)-carboxylate.
N,N-dimethyl-2-(5-(4,4,5,5-tetramethyl-1,3,2-dioxaborolan-2-yl)benzo[*d*]thiazol-2-yl)ethan-1-amine (1 eq), *tert*-butyl 3-methyl-6-(trifluoromethylsulfonyloxy)-3,4-dihydro-2H-pyridine-1-carboxylate (1.2 eq) , sodium carbonate (3 eq) were mixed together in dioxane-water mixture (3:1). The resulting mixture was evacuated and then backfilled with Ar. This operation was repeated two times, then Pd(dppf)Cl_2•DCM (819.86 mg, 1.00 mmol) was added and the reaction mixture was stirred under Ar at 90 °C overnight, then cooled down and concentrated under vacuum. The residue was diluted with MTBE and stirred for 0.5 h. Anhydrous sodium sulfate was added and the resulting mixture was filtered. The filter cake was then washed with MTBE (5 x 50 mL). The filtrate was concentrated under vacuum to afford *tert*-butyl 6-(2-(2-(dimethylamino)ethyl)benzo[*d*]thiazol-5-yl)-3-methyl-3,4-dihydropyridine-1(2*H*)-carboxylate. LCMS (ESI): [M]+ m/z: calcd 401.2; found 402.2; Rt = 1.064 min.

N,N-dimethyl-2-(5-(5-methyl-3,4,5,6-tetrahydropyridin-2-yl)benzo[*d*]thiazol-2-yl)ethan-1-amine.
tert-butyl 6-(2-(2-(dimethylamino)ethyl)benzo[*d*]thiazol-5-yl)-3-methyl-3,4-dihydropyridine-1(2*H*)-carboxylate (1 eq) in TFA (15 eq) was stirred at room temperature for 1 h and then concentrated under vacuum. Cold water was added to the residue and the resulting mixture was extracted with DCM twice. The DCM layer was discarded, and the aqueous layer was basified to pH 11. The resulting mixture was extracted with DCM twice. The combined organic extracts were dried over sodium sulfate and concentrated under vacuum to afford *N,N*-dimethyl-2-(5-(5-methyl-3,4,5,6-tetrahydropyridin-2-yl)benzo[*d*]thiazol-2-yl)ethan-1-amine. LCMS (ESI): [M]+ m/z: calcd 301.2; found 302.2; Rt = 0.641 min.

rac-N,N-dimethyl-2-(5-(*trans*-5-methylpiperidin-2-yl)benzo[*d*]thiazol-2-yl)ethan-1-amine, **Intermediate 50a**.
N,N-dimethyl-2-(5-(5-methyl-3,4,5,6-tetrahydropyridin-2-yl)benzo[*d*]thiazol-2-yl)ethan-1-amine (1 eq) was dissolved in MeOH and the resulting solution was cooled to 0 °C in an ice bath. Sodium borohydride (2 eq) was added portion wise and the reaction mixture was allowed to warm to room temperature and stirred overnight. Water was added to the reaction mixture and the resulting mixture was concentrated in vacuum. The residue was diluted with water and the resulting mixture was extracted with DCM twice, dried over Na_2SO_4, filtered, and evaporated to obtain *rac-N,N*-dimethyl-2-(5-(*trans*-5-methylpiperidin-2-yl)benzo[*d*]thiazol-2-yl)ethan-1-amine, **Intermediate 50a.** LCMS (ESI): [M]+ m/z: calcd 303.2; found 304.2; Rt = 0.486 min.

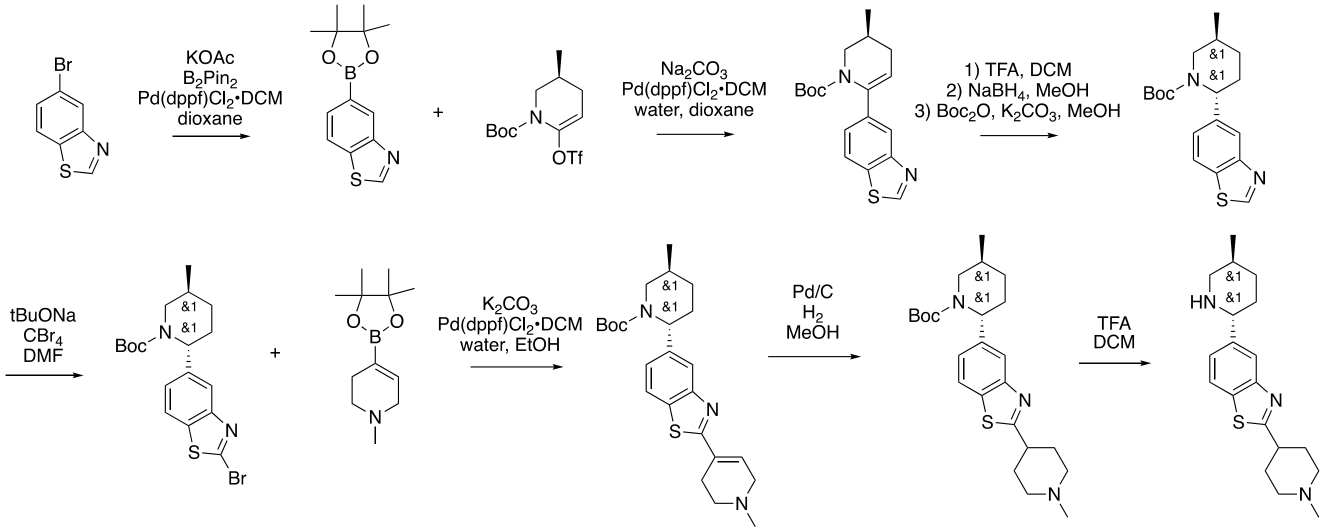

Intermediate 51a

5-(4,4,5,5-tetramethyl-1,3,2-dioxaborolan-2-yl)benzo[*d*]thiazole.
A mixture of 5-bromo-1,3-benzothiazole (5 g, 23.4 mmol), KOAc (1.32 g, 46.7 mmol), 4,4,5,5-tetramethyl-2-(4,4,5,5-tetramethyl-1,3,2-dioxaborolan-2-yl)-1,3,2-dioxaborolane (9.00 g, 35.4 mmol), cyclopentyl(diphenyl)phosphane;dichloropalladium;iron (1.71 g, 2.34 mmol) and dioxane (50 mL) was stirred at 100 °C for 24 h. The resulting mixture was quenched by addition of water (100 mL) and extracted with EtOAc (100 mL x 3). The combined organic layer was washed with saturated NH_4Cl aqueous solution (100 mL x 2), brine (100 mL), dried over anhydrous Na_2SO_4, filtered, and concentrated under reduced pressure to give a residue which was purified by flash chromatography (ISCO®; 24 g AgelaFlash® Silica Flash Column, petroleum ether/EtOAc with EtOAc from 0~10%, Flow Rate: 30 mL/min, 254 nm) to afford 5-(4,4,5,5-tetramethyl-1,3,2-dioxaborolan-2-yl)-1,3-benzothiazole (5.4 g, 89% yield) as a white solid.

tert-butyl 6-(benzo[*d*]thiazol-5-yl)-3-methyl-3,4-dihydropyridine-1(2*H*)-carboxylate.
A mixture of 5-(4,4,5,5-tetramethyl-1,3,2-dioxaborolan-2-yl)-1,3-benzothiazole (5 g, 19.2 mmol), *tert*-butyl 3-methyl-6-(trifluoromethylsulfonyloxy)-3,4-dihydro-2*H*-pyridine-1-carboxylate (7.6 g, 22.0 mmol), Na_2CO_3 (6.60 g, 62.3 mmol), cyclopentyl(diphenyl)phosphane;dichloromethane;dichloropalladium;iron (1.60 g, 1.96 mmol), dioxane (50 mL) and H_2O (20 mL) was stirred at 85 °C for 12 h. The resulting mixture was quenched by addition of water (100 mL) and extracted with EtOAc (100 mL x 3). The combined organic layer was washed with saturated NH_4Cl aqueous solution (100 mL x 2), brine (100 mL), dried over anhydrous Na_2SO_4, filtered, and concentrated under reduced pressure to give a residue, which was purified by flash chromatography (ISCO®; 24 g AgelaFlash® Silica Flash Column, petroleum ether/EtOAc with EtOAc from 0~10%, Flow Rate: 30 mL/min) to afford *tert*-butyl 6-(benzo[*d*]thiazol-5-yl)-3-methyl-3,4-dihydropyridine-1(2*H*)-carboxylate (4 g, 63% yield) as yellow oil. ^1H NMR (400 MHz, MeOD-*d4*) δ 9.20 - 9.34 (m, 1H), 7.95 - 8.03 (m, 2H), 7.46 (dd, *J* = 8.4, 1.6 Hz, 1H), 5.51 (t, *J* = 3.8 Hz, 1H), 4.03 (dd, *J* = 12.3, 3.3 Hz, 1H), 3.17 (dd, *J* = 12.4, 8.9 Hz, 1H), 2.50 (ddd, *J* = 18.6, 6.1, 3.9 Hz, 1H), 1.99 - 2.08 (m, 1H), 1.89 - 1.99 (m, 1H), 0.94 - 1.13 (m, 12H).

rac-tert-butyl *trans*-2-(benzo[*d*]thiazol-5-yl)-5-methylpiperidine-1-carboxylate.
A mixture of *tert*-butyl 6-(benzo[*d*]thiazol-5-yl)-3-methyl-3,4-dihydropyridine-1(2*H*)-carboxylate (4 g, 12.1 mmol), TFA (64.9 mmol, 5 mL) and DCM (5 mL) was stirred at 20 °C for 1 h. Then the mixture was concentrated under reduced pressure to give a mixture to which was added K_2CO_3 (5.03 g, 36.42 mmol), MeOH (20 mL) and $NaBH_4$ (1 g, 26.43 mmol) at 0 °C, then the mixture was stirred at 0 °C for 0.5 h. *tert*-butoxycarbonyl *tert*-butyl carbonate (6.67 g, 30.6 mmol) and H_2O (10 mL) was then added to the mixture and the mixture was stirred at 20 °C for 2.5 h. The resulting mixture was quenched by addition of water (100 mL) and extracted with EtOAc (100 mL x 3). The combined organic layer was washed with saturated NH_4Cl aqueous solution (100 mL x 2), brine (100 mL), dried over anhydrous Na_2SO_4, filtered, and concentrated under reduced pressure to give a residue which was purified by flash chromatography (ISCO®; 24 g AgelaFlash® Silica Flash Column, petroleum ether/EtOAc with EtOAc from 0~20%, Flow Rate: 30 mL/min) to afford *rac-tert*-butyl *trans*-2-(benzo[*d*]thiazol-5-yl)-5-methylpiperidine-1-carboxylate (3.2 g, 80% yield) as a yellow oil.

rac-tert-butyl *trans*-2-(2-bromobenzo[*d*]thiazol-5-yl)-5-methylpiperidine-1-carboxylate.
To a mixture of *rac-tert*-butyl *trans*-2-(benzo[*d*]thiazol-5-yl)-5-methylpiperidine-1-carboxylate (3.2 g, 9.63 mmol), sodium 2-methylpropan-2-olate (3.73 g, 38.8 mmol) and DMF (10 mL) was added CBr_4 (3.54 g, 10.7 mmol) and the mixture was stirred at 20 °C for 0.5 h. The resulting mixture was quenched by addition of water (30 mL) and extracted with EtOAc (100 mL x 3). The combined organic layer was washed with saturated NH_4Cl aqueous solution (30 mL x 2), brine (30 mL), dried over anhydrous Na_2SO_4, filtered, and concentrated under reduced pressure to give *rac-tert*-butyl *trans*-2-(2-bromobenzo[*d*]thiazol-5-yl)-5-methylpiperidine-1-carboxylate (3.2 g, 81% yield) as yellow oil. ^1H NMR (400 MHz, MeOD-*d4*) δ 7.79 - 7.86 (m, 1H), 7.70 (s, 1H), 7.28 (br d, *J* = 9.5 Hz, 1H), 3.65 (br d, *J* = 12.1 Hz, 1H), 2.97 - 3.03 (m, 1H), 1.97 - 2.14 (m, 3H), 1.63 - 1.80 (m, 3H), 1.33 - 1.36 (m, 9H), 0.95 - 0.98 (m, 3H).

rac-tert-butyl *trans*-5-methyl-2-(2-(1-methyl-1,2,3,6-tetrahydropyridin-4-yl)benzo[*d*]thiazol-5-yl)piperidine-1-carboxylate.
To a mixture of *rac-tert*-butyl *trans*-2-(2-bromobenzo[*d*]thiazol-5-yl)-5-methylpiperidine-1-carboxylate (2.5 g, 6.08 mmol), 1-methyl-4-(4,4,5,5-tetramethyl-1,3,2-dioxaborolan-2-yl)-3,6-dihydro-2H-pyridine (2 g, 8.96 mmol) in EtOH (6 mL) and H_2O (2 mL) were added $Pd(PPh_3)_4$ (750 mg, 0.649 mmol) and K_2CO_3 (2.60 g, 18.8 mmol). The resulting mixture was sealed and degassed under vacuum and purged with N_2 three times, and then stirred at 95 °C for 12 h under N_2 atmosphere. The resulting mixture was quenched by addition of water (50 mL) and extracted with EtOAc (100 mL x 3). The combined organic layer was washed with saturated NH_4Cl aqueous solution (50 mL x 2), brine (50 mL), dried over anhydrous Na_2SO_4, filtered, and concentrated under reduced pressure to give a residue which was purified by flash chromatography (ISCO®; 25g AgelaFlash® Silica Flash Column, DCM/MeOH with MeOH from 0~10%,

Flow Rate: 30 mL/min, 254 nm) to afford *rac-tert*-butyl *trans*-5-methyl-2-(2-(1-methyl-1,2,3,6-tetrahydropyridin-4-yl)benzo[*d*]thiazol-5-yl)piperidine-1-carboxylate (1.6 g, 62% yield) as brown solid. LCMS (ESI): [M+H]+ m/z: calcd 428.2, found 428.1.

rac-tert-butyl *trans*-5-methyl-2-(2-(1-methylpiperidin-4-yl)benzo[*d*]thiazol-5-yl)piperidine-1-carboxylate.
A mixture of *rac-tert*-butyl *trans*-5-methyl-2-(2-(1-methyl-1,2,3,6-tetrahydropyridin-4-yl)benzo[*d*]thiazol-5-yl)piperidine-1-carboxylate (1.6 g, 3.74 mmol), Pd/C (1.09 g, 10 wt% of Pd with 50 wt% of water) and MeOH (20 mL) was stirred at 20 °C for 36 h under H_2 (15 psi). The mixture was filtered and concentrated under reduced pressure to give *rac-tert*-butyl *trans*-5-methyl-2-(2-(1-methylpiperidin-4-yl)benzo[*d*]thiazol-5-yl)piperidine-1-carboxylate (800 mg, 50% yield) as yellow oil. ^{1}H NMR (400 MHz, MeOD-d_4) δ 7.94 (br d, *J* = 8.4 Hz, 1H), 7.80 (s, 1H), 7.29 - 7.37 (m, 1H), 5.38 (br s, 1H), 3.77 (br d, *J* = 13.5 Hz, 1H), 3.07 - 3.19 (m, 2H), 3.01 (br d, *J* = 10.5 Hz, 2H), 2.33 (s, 3H), 2.14 - 2.30 (m, 6H), 1.93 - 2.07 (m, 3H), 1.78 (br s, 2H), 1.45 (s, 9H), 1.07 (br d, *J* = 7.0 Hz, 3H). LCMS (ESI): [M+H]+ m/z: calcd 430.2, found 430.2.

rac-5-(*trans*-5-methylpiperidin-2-yl)-2-(1-methylpiperidin-4-yl)benzo[*d*]thiazole, **Intermediate 51a**.
To a mixture of *rac-tert*-butyl *trans*-5-methyl-2-(2-(1-methylpiperidin-4-yl)benzo[*d*]thiazol-5-yl)piperidine-1-carboxylate (1.1 g, 2.56 mmol) in DCM (10 mL) was added TFA (26.0 mmol, 2 mL). The resulting mixture was stirred at 20 °C for 2 h. The mixture was concentrated under reduced pressure to give a residue and Na_2CO_3 and MeOH were added. The mixture was stirred at 20 °C for 1 h. The resulting solution was concentrated under reduced pressure to give a residue to which DCM (50 mL) was added. The mixture was filtered and concentrated under reduced pressure to give *rac*-5-(*trans*-5-methylpiperidin-2-yl)-2-(1-methylpiperidin-4-yl)benzo[*d*]thiazole, **Intermediate 51a** (800 mg, crude) as yellow oil. LCMS (ESI): [M+H]+ m/z: calcd 330.2, found 330.1.

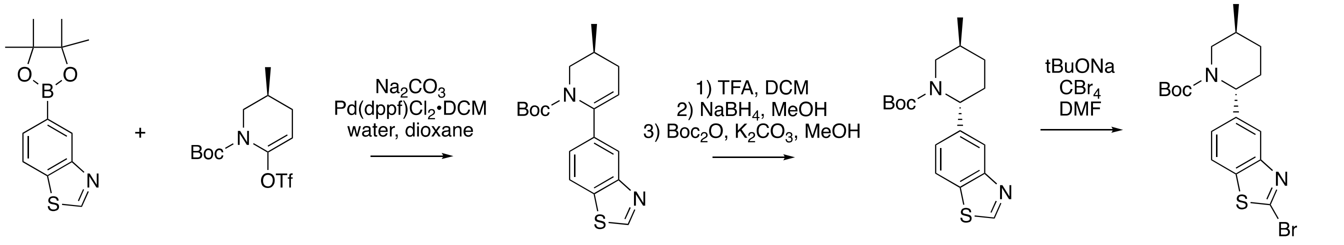

Intermediate 52a'

tert-butyl (*S*)-6-(benzo[*d*]thiazol-5-yl)-3-methyl-3,4-dihydropyridine-1(2*H*)-carboxylate.
A mixture of 5-(4,4,5,5-tetramethyl-1,3,2-dioxaborolan-2-yl)-1,3-benzothiazole (4 g, 15.3 mmol), *tert*-butyl (3*S*)-3-methyl-6-(trifluoromethylsulfonyloxy)-3,4-dihydro-2*H*-pyridine-1-carboxylate (6.0 g, 17.4 mmol), Na_2CO_3 (5.28 g, 49.8 mmol), cyclopentyl(diphenyl)phosphane;dichloromethane;dichloropalladium;iron (1.28 g, 1.57 mmol), dioxane (50 mL), and H_2O (20 mL) were stirred at 85 °C for 12 h. The resulting mixture was quenched by addition of water (100 mL) and extracted with EtOAc (100 mL x 3). The combined organic layer was washed with saturated NH_4Cl aqueous solution (100 mL x 2), brine (100 mL), dried over anhydrous Na_2SO_4, filtered, and concentrated under reduced pressure to give a residue which was purified by flash chromatography (ISCO®; 24 g AgelaFlash® Silica Flash Column, petroleum ether/EtOAc with EtOAc from 0~10%, Flow Rate: 30 mL/min, 254 nm) to afford *tert*-butyl (*S*)-6-(benzo[*d*]thiazol-5-yl)-3-methyl-3,4-dihydropyridine-1(2*H*)-carboxylate (4.1 g, 81% yield) as yellow solid. LCMS (ESI): [M]+ m/z: calcd 329.2; found 330.2; Rt = 1.635 min.

tert-butyl (2*R*,5*S*)-2-(benzo[*d*]thiazol-5-yl)-5-methylpiperidine-1-carboxylate.
A mixture of *tert*-butyl (*S*)-6-(benzo[*d*]thiazol-5-yl)-3-methyl-3,4-dihydropyridine-1(2*H*)-carboxylate (4.1 g, 12.4 mmol), TFA (7.58 g, 66.5 mmol) and DCM (5 mL) was stirred at 20 °C for 1 h. The mixture was concentrated under reduced pressure to give a residue which was mixed with K_2CO_3 (5.16 g, 37.3 mmol), MeOH (20 mL) and $NaBH_4$ (410 mg, 10.8 mmol) at 0 °C stirred at 0°C for 30 minutes. *tert*-butoxycarbonyl *tert*-butyl carbonate (6.83 g, 31.3 mmol) and H_2O (10 mL) were added and the mixture was stirred at 20 °C for 2.5 h then quenched by addition of water (100 mL) and extracted with EtOAc (100 mL x 3). The combined organic layer was washed with saturated NH_4Cl aqueous solution (100 mL x 2), brine (100 mL), dried over anhydrous Na_2SO_4, filtered, and concentrated under reduced pressure to give a residue which was purified by flash chromatography (ISCO®; 24 g AgelaFlash® Silica Flash Column, petroleum ether/EtOAc with EtOAc from 0~20%, Flow Rate: 30 mL/min, 254nm) to afford *tert*-butyl (2*R*,5*S*)-2-(benzo[*d*]thiazol-5-yl)-5-methylpiperidine-1-carboxylate (3 g, 73% yield) as yellow oil. LCMS (ESI): [M]+ m/z: calcd 332.2; found 333.2; Rt = 3.995 min.

tert-butyl (2*R*,5*S*)-2-(2-bromobenzo[*d*]thiazol-5-yl)-5-methylpiperidine-1-carboxylate, **Intermediate 52a'**.
To a mixture of *tert*-butyl (2*R*,5*S*)-2-(benzo[*d*]thiazol-5-yl)-5-methylpiperidine-1-carboxylate (2.83 g, 8.51 mmol), sodium;2-methylpropan-2-olate (2.45 g, 25.5 mmol) and DMF (10 mL) was added CBr_4 (3.13 g, 9.44 mmol), and the mixture was stirred at 20 °C for 1 h. then quenched by addition of water (30 mL) and extracted with EtOAc (100 mL x 3). The combined organic layer was washed with saturated NH_4Cl aqueous solution (30 mL x 2), brine (30 mL), dried over anhydrous Na_2SO_4, filtered, and concentrated under reduced pressure to give a residue which was purified by flash chromatography (ISCO®; 40 g AgelaFlash® Silica Flash Column, petroleum ether/EtOAc with EtOAc from 0~20%, Flow Rate: 30 mL/min, 254 nm) to afford **Intermediate 52a'**, *tert*-butyl (2*R*,5*S*)-2-(2-bromobenzo[*d*]thiazol-5-yl)-5-methylpiperidine-1-carboxylate (1.4 g, 40% yield) as yellow solid. LCMS (ESI): [M]+ m/z: calcd 411.2; found 412.2; Rt = 4.734 min.

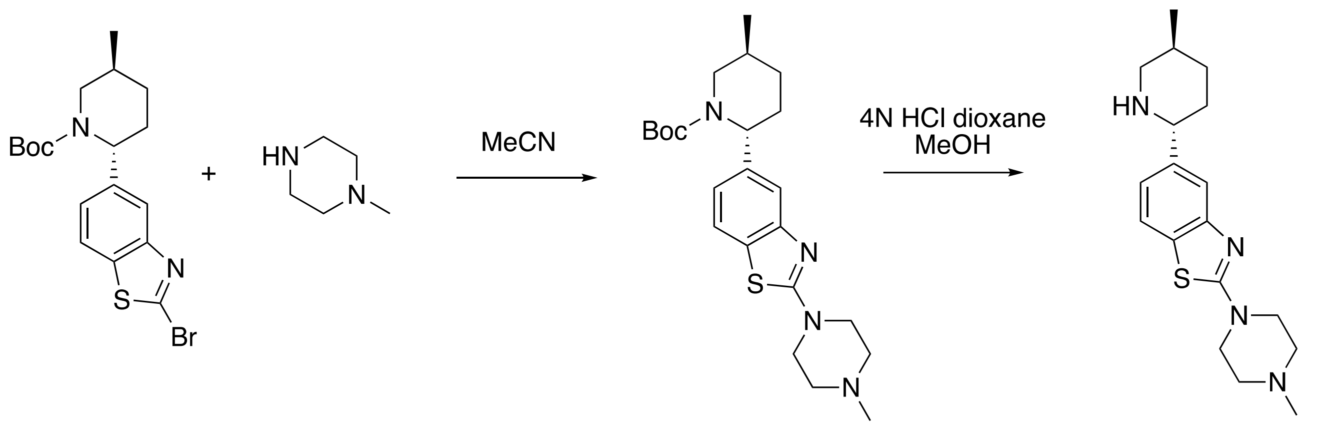

Intermediate 52a'

Intermediate 52a

tert-butyl (2*R*,5*S*)-5-methyl-2-(2-(4-methylpiperazin-1-yl)benzo[*d*]thiazol-5-yl)piperidine-1-carboxylate.
1-Methylpiperazine (852.22 mg, 8.51 mmol, 943.77 µL) was added in one portion at 25 °C to a solution of **Intermediate 52a'** (700 mg, 1.70 mmol) in MeCN (20 mL). The resulting mixture was stirred at 70 °C for 8 h, then cooled down and concentrated under vacuum. The residue was basified to pH 11 with 10 % aqueous sodium hydroxide solution and extracted with DCM (2 x 25 mL). The combined organic extracts were dried over sodium sulfate and concentrated under vacuum to afford *tert*-butyl (2*R*,5*S*)-5-methyl-2-(2-(4-methylpiperazin-1-yl)benzo[*d*]thiazol-5-yl)piperidine-1-carboxylate (750 mg, crude) as brown solid, which was used directly in the next step. LCMS (ESI): [M]+ m/z: calcd 430.2; found 431.2; Rt = 3.638 min.

2-(4-methylpiperazin-1-yl)-5-((2*R*,5*S*)-5-methylpiperidin-2-yl)benzo[*d*]thiazole, **Intermediate 52a**.
4.0 M hydrogen chloride solution in dioxane (31.50 g, 120.09 mmol, 39.38 mL) was added in one portion to a stirred solution of *tert*-butyl (2*R*,5*S*)-5-methyl-2-(2-(4-methylpiperazin-1-yl)benzo[*d*]thiazol-5-yl)piperidine-1-carboxylate (775.25 mg, 1.80 mmol) in MeOH (25 mL). The resulting mixture was stirred at 25 °C for 1 h, and then evaporated to dryness under vacuum to afford crude 2-(4-methylpiperazin-1-yl)-5-((2*R*,5*S*)-5-methylpiperidin-2-yl)benzo[*d*]thiazole, **Intermediate 52a** (900 mg, crude, 3 HCl) as brown solid, which was used directly in the next step. LCMS (ESI): [M]+ m/z: calcd 330.2; found 331.2; Rt = 0.544 min.

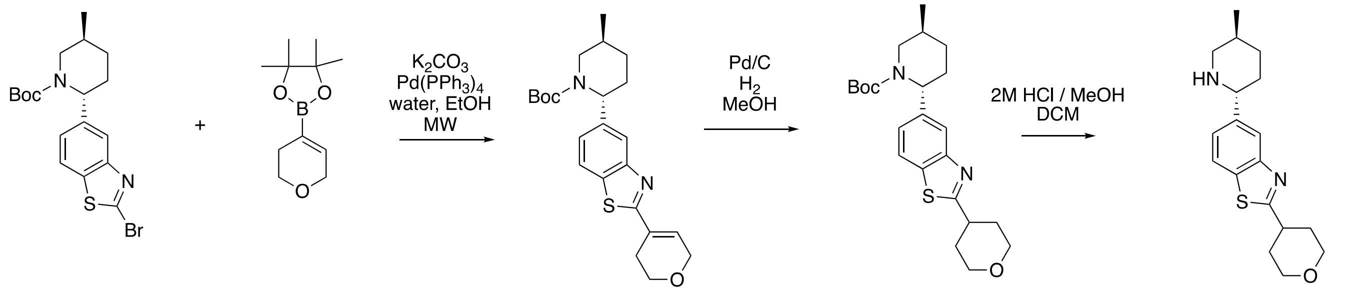

Intermediate 52a'

Intermediate 54a

tert-butyl (2*R*,5*S*)-2-(2-(3,6-dihydro-2*H*-pyran-4-yl)benzo[*d*]thiazol-5-yl)-5-methylpiperidine-1-carboxylate.
A mixture of *tert*-butyl (2*R*,5*S*)-2-(2-bromobenzo[*d*]thiazol-5-yl)-5-methylpiperidine-1-carboxylate, **Intermediate 52a'** (200 mg, 0.486 mmol), 2-(3,6-dihydro-2*H*-pyran-4-yl)-4,4,5,5-tetramethyl-1,3,2-dioxaborolane (120 mg, 0.571 mmol), K_2CO_3 (200 mg, 1.45 mmol), $Pd(PPh_3)_4$ (60 mg, 51.9 µmol), EtOH (5 mL) and H_2O (1 mL) was stirred at 95 °C for 1.5 h. The resulting mixture was quenched by addition of water (10 mL) and extracted with EtOAc (20 mL x 3). The combined organic layer was washed with saturated NH_4Cl aqueous solution (10 mL x 2), brine (10 mL), dried over anhydrous Na_2SO_4, filtered, and concentrated under reduced pressure to give a residue which was purified by flash chromatography (ISCO®; 24 g AgelaFlash® Silica Flash Column, petroleum ether/EtOAc with EtOAc from 0~40%, Flow Rate: 30 mL/min, 254 nm) to afford *tert*-butyl (2*R*,5*S*)-2-(2-(3,6-dihydro-2*H*-pyran-4-yl)benzo[*d*]thiazol-5-yl)-5-methylpiperidine-1-carboxylate (200 mg, 99% yield) as yellow solid. LCMS (ESI): [M]+ m/z: calcd 414.56; found 415.1; Rt = 1.024 min.

tert-butyl (2*R*,5*S*)-5-methyl-2-(2-(tetrahydro-2*H*-pyran-4-yl)benzo[*d*]thiazol-5-yl)piperidine-1-carboxylate.
A mixture of *tert*-butyl (2*R*,5*S*)-2-(2-(3,6-dihydro-2*H*-pyran-4-yl)benzo[*d*]thiazol-5-yl)-5-methylpiperidine-1-carboxylate (200 mg, 0.482 mmol), Pd/C (200 mg, 10 wt % Pd/C with 50 wt % water) and MeOH (5 mL) was stirred at 30 °C for 12 h under H_2 atmosphere (15 psi). The mixture was filtered and concentrated under reduced pressure to give *tert*-butyl (2*R*,5*S*)-5-methyl-2-(2-(tetrahydro-2*H*-pyran-4-yl)benzo[*d*]thiazol-5-yl)piperidine-1-carboxylate (130 mg, 65% yield) as yellow oil. LCMS (ESI): [M]+ m/z: calcd 416.58; found 417.2; Rt = 1.045 min

5-((2*R*,5*S*)-5-methylpiperidin-2-yl)-2-(tetrahydro-2*H*-pyran-4-yl)benzo[*d*]thiazole, **Intermediate 54a**.
A mixture of *tert*-butyl (2*R*,5*S*)-5-methyl-2-(2-(tetrahydro-2*H*-pyran-4-yl)benzo[*d*]thiazol-5-yl)piperidine-1-carboxylate (130 mg, 0.312 mmol), 2 M MeOH / HCl (1 mL, 2 mmol) and DCM (4 mL) was stirred at 20 °C for 2 h. The mixture was concentrated under reduced pressure to give **Intermediate 54a**, 5-

((2*R*,5*S*)-5-methylpiperidin-2-yl)-2-(tetrahydro-2*H*-pyran-4-yl)benzo[*d*]thiazole (120 mg, crude, HCl) as a yellow solid. LCMS (ESI): [M]+ m/z: calcd 316.46; found 317.1; Rt = 0.708 min.

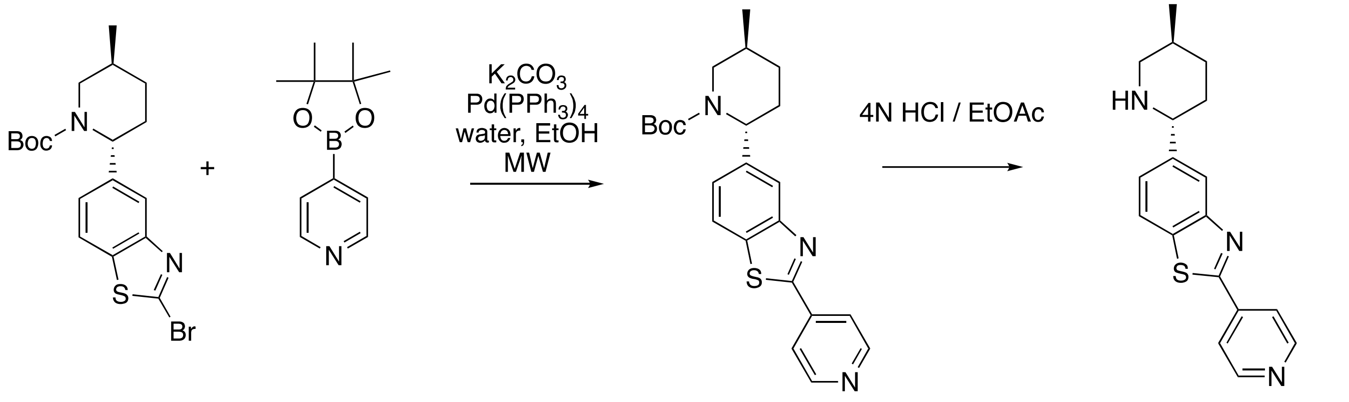

Intermediate 52a'

Intermediate 55a

tert-butyl (2*R*,5*S*)-5-methyl-2-(2-(pyridin-4-yl)benzo[*d*]thiazol-5-yl)piperidine-1-carboxylate.
A mixture of *tert*-butyl 2-(2-bromo-1,3-benzothiazol-5-yl)-5-methyl-piperidine-1-carboxylate, **Intermediate 52a'** (300 mg, 0.739 mmol), 4-(4,4,5,5-tetramethyl-1,3,2-dioxaborolan-2-yl)pyridine (160 mg, 0.780 mmol), $Pd(PPh_3)_4$ (90.0 mg, 77.9 umol), K_2CO_3 (312 mg, 2.26 mmol), H_2O (3 mL) and EtOH (10 mL) was stirred at 95 °C for 1.5 hours under microwave. The resulting mixture was quenched by addition of water (10 mL) and extracted with EtOAc (50 mL x 3). The combined organic layer was washed with saturated NH_4Cl aqueous solution (10 mL x 2), brine (10 mL), dried over anhydrous Na_2SO_4, filtered and concentrated under reduced pressure to give a residue which was purified by flash chromatography (ISCO®; 12 g AgelaFlash® Silica Flash Column, petroleum ether/EtOAc with EtOAc from 0~60%, Flow Rate: 30 mL/min, 254nm) to afford *tert*-butyl (2*R*,5*S*)-5-methyl-2-(2-(pyridin-4-yl)benzo[*d*]thiazol-5-yl)piperidine-1-carboxylate (210 mg, 70.3% yield) as a yellow solid.

5-((2*R*,5*S*)-5-methylpiperidin-2-yl)-2-(pyridin-4-yl)benzo[*d*]thiazole, **Intermediate 55a**. A mixture of *tert*-butyl (2*R*,5*S*)-5-methyl-2-(2-(pyridin-4-yl)benzo[*d*]thiazol-5-yl)piperidine-1-carboxylate (200 mg, 0.488 mmol) and 4 M HCl / EtOAc (4 mL, 16 mmol) was stirred at 20 °C for 2 h. The mixture was concentrated under reduced pressure to give 5-((2*R*,5*S*)-5-methylpiperidin-2-yl)-2-(pyridin-4-yl)benzo[*d*]thiazole, **Intermediate 55a** (176 mg, crude, HCl) as a yellow solid. LCMS (ESI): [M]+ m/z: calcd 309.43; found 310.1; Rt = 0.669 min

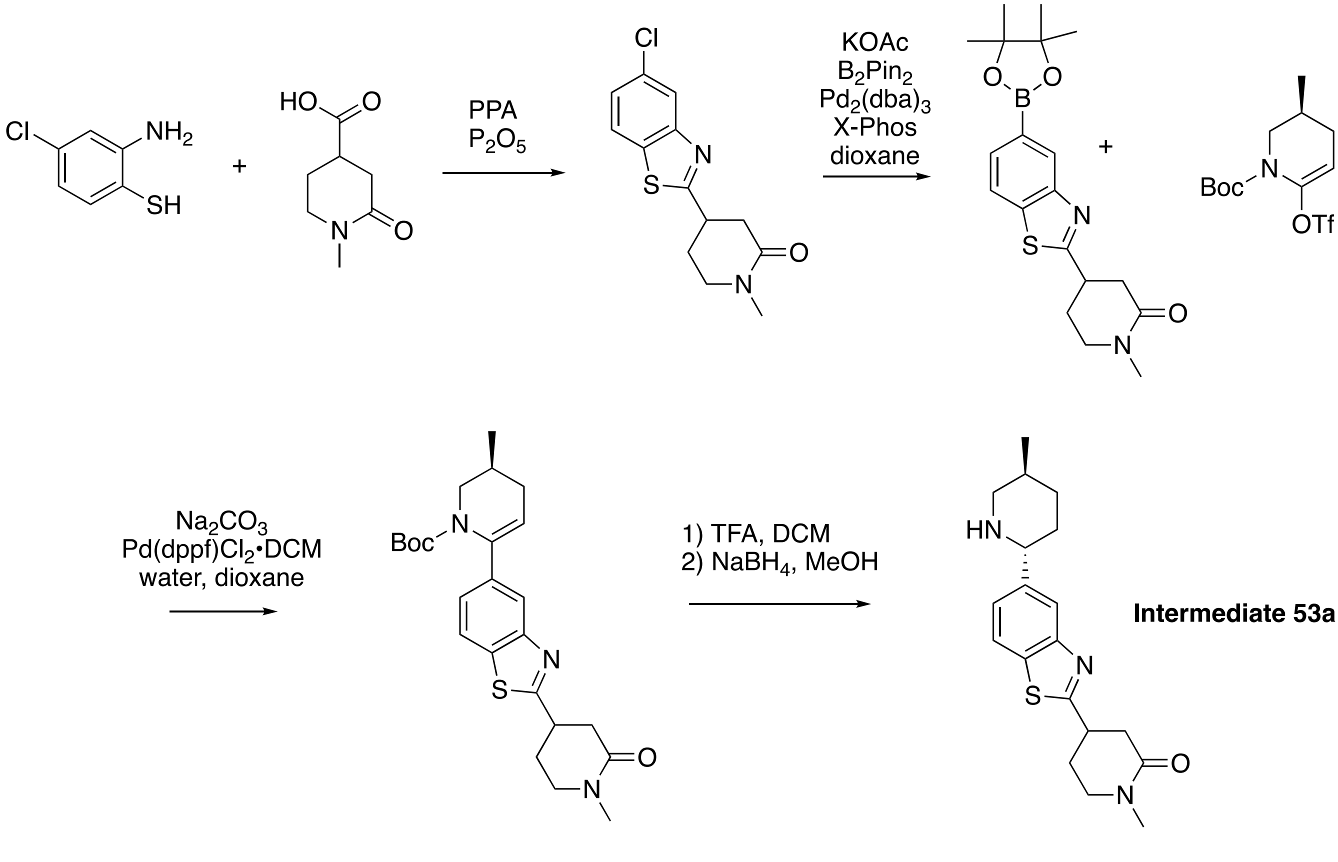

Intermediate 53a

4-(5-chlorobenzo[*d*]thiazol-2-yl)-1-methylpiperidin-2-one.
Phosphoric acid (4 eq) and phosphorus pentoxide (4 eq) were mixed together. The reaction suspension was stirred at room temperature for 10 min, then 2-amino-4-chlorobenzenethiol (1 eq) followed by 1-methyl-2-oxopiperidine-4-carboxylic acid (1.2 eq) were added under Ar. The solution was stirred at 110 °C for 18 h then it was triturated with water, basified (NaOH, 10% aq.) to pH=10, extracted with DCM twice, dried under Na_2SO_4, and evaporated under vacuum and purified by silica gel with $CHCl_3$/MTBE (gradient 10-100% MTBE) to give 4-(5-chlorobenzo[*d*]thiazol-2-yl)-1-methylpiperidin-2-one as a white solid. Yield: 35%. LCMS (ESI): [M]+ m/z: calcd 280.2; found 281.2; Rt = 1.118 min.

1-methyl-4-(5-(4,4,5,5-tetramethyl-1,3,2-dioxaborolan-2-yl)benzo[*d*]thiazol-2-yl)piperidin-2-one.
tris(dibenzylideneacetone)dipalladium(0) (303.31 mg, 331.23 umol) and XPhos (631.61 mg, 1.32 mmol) were added to a solution of 4-(5-chlorobenzo[*d*]thiazol-2-yl)-1-methylpiperidin-2-one (3.1 g, 11.04 mmol) and 4,4,5,5-tetramethyl-2-(4,4,5,5-tetramethyl-1,3,2-dioxaborolan-2-yl)-1,3,2-dioxaborolane (3.64 g, 14.35 mmol) in dioxane (100 mL). The flask was evacuated and refilled with Ar 3 times. Potassium acetate (2.17 g, 22.08 mmol, 1.38 mL) was added under a stream of Ar. The resulting mixture was stirred at 100 °C for 12 h under Ar, then cooled and evaporated under vacuum. Water (200 mL) was added and it was extracted with DCM (2 x 100 mL), dried over sodium sulfate, and evaporated under vacuum to give 5 g of crude product which was purified by silica gel column chromatography using $CHCl_3$/MeCN gradient (10-100% MeCN) to afford 1-methyl-4-(5-(4,4,5,5-tetramethyl-1,3,2-dioxaborolan-2-yl)benzo[*d*]thiazol-2-yl)piperidin-2-one (3 g, 8.06 mmol, 73% yield). LCMS (ESI): [M]+ m/z: calcd 372.2; found 373.2; Rt = 1.299 min.

tert-butyl (3*S*)-3-methyl-6-(2-(1-methyl-2-oxopiperidin-4-yl)benzo[*d*]thiazol-5-yl)-3,4-dihydropyridine-1(2*H*)-carboxylate.
1-methyl-4-(5-(4,4,5,5-tetramethyl-1,3,2-dioxaborolan-2-yl)benzo[*d*]thiazol-2-yl)piperidin-2-one (1 eq), *tert*-butyl (3*S*)-3-methyl-6-(trifluoromethylsulfonyloxy)-3,4-dihydro-2*H*-pyridine-1-carboxylate (1.2 eq), and sodium carbonate (3 eq) were mixed together in a dioxane-water mixture (3:1). The resulting mixture was evacuated and then backfilled with Ar. This operation was repeated two times, then Pd(dppf)Cl_2•DCM (819.86 mg, 1.00 mmol) was added and the reaction mixture was stirred under Ar at 90 °C overnight, then cooled and concentrated under vacuum. The residue was diluted with MTBE and stirred for 0.5 h. Anhydrous sodium sulfate was added and the resulting mixture was filtered. The filter cake was washed with MTBE (5 x 50 mL) and discarded. The filtrate was concentrated in vacuum to afford *tert*-butyl (3*S*)-3-methyl-6-(2-(1-methyl-2-oxopiperidin-4-yl)benzo[*d*]thiazol-5-yl)-3,4-dihydropyridine-1(2*H*)-carboxylate. LCMS (ESI): [M]+ m/z: calcd 441.2; found 442.2; Rt = 4.017 min.

1-methyl-4-(5-((2*R*,5*S*)-5-methylpiperidin-2-yl)benzo[*d*]thiazol-2-yl)piperidin-2-one, **Intermediate 53a**.
A solution of *tert*-butyl (3*S*)-3-methyl-6-(2-(1-methyl-2-oxopiperidin-4-yl)benzo[*d*]thiazol-5-yl)-3,4-dihydropyridine-1(2*H*)-carboxylate (1 eq) in TFA (15 eq) was stirred at room temperature for 1 h, and then concentrated under vacuum. Cold water was added to the residue and the resulting mixture was extracted with DCM twice. The DCM layer was discarded, and the aqueous layer was basified to pH 11. The resulting mixture was extracted with DCM twice. The combined organic extracts were dried over sodium sulfate and concentrated under vacuum to afford 1-methyl-4-(5-((*S*)-5-methyl-3,4,5,6-tetrahydropyridin-2-yl)benzo[*d*]thiazol-2-yl)piperidin-2-one. LCMS (ESI): [M]+ m/z: calcd 341.2; found 342.2; Rt = 0.860 min. 1-methyl-4-(5-((*S*)-5-methyl-3,4,5,6-tetrahydropyridin-2-yl)benzo[*d*]thiazol-2-yl)piperidin-2-one (1 eq) was dissolved in MeOH and the resulting solution was cooled to 0 °C in an ice bath. Sodium borohydride (2 eq) was added portion-wise to the solution. After addition was complete, the reaction mixture was allowed to warm to room temperature and stirred overnight. Water was added to the reaction mixture and the resulting mixture was concentrated under vacuum. The residue was diluted with water and the resulting mixture was extracted with DCM twice, dried over Na_2SO_4, filtered, and evaporated to obtain 1-methyl-4-(5-((2*R*,5*S*)-5-methylpiperidin-2-yl)benzo[*d*]thiazol-2-yl)piperidin-2-one, **Intermediate 53a**. LCMS (ESI): [M]+ m/z: calcd 343.2; found 344.2; Rt = 0.706 min.

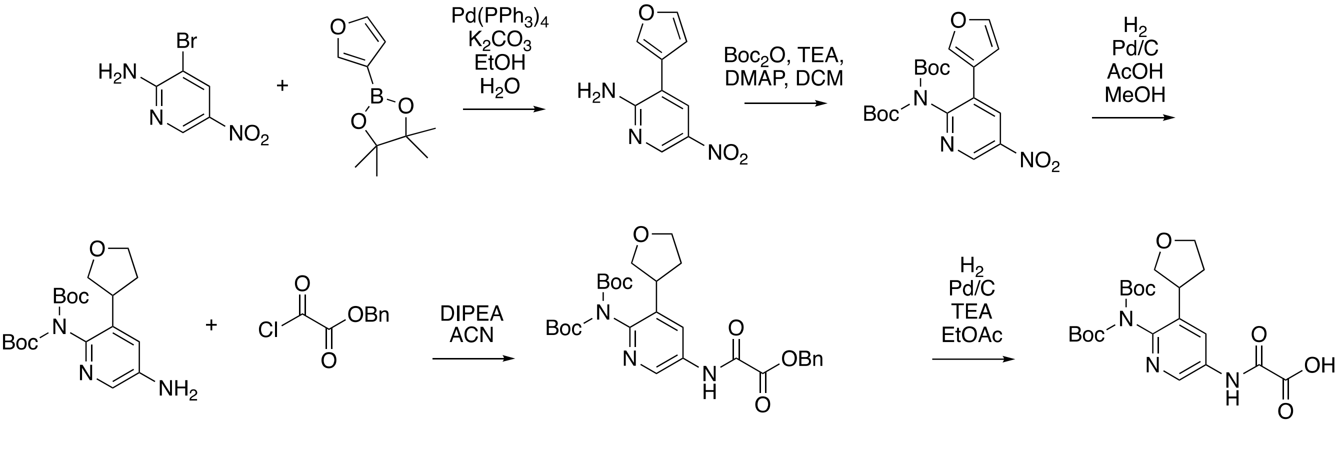

Intermediate 5a

3-(furan-3-yl)-5-nitropyridin-2-amine.
To a mixture of 3-bromo-5-nitro-pyridin-2-amine (3 g, 13.8 mmol) and 2-(3-furyl)-4,4,5,5-tetramethyl-1,3,2-dioxaborolane (3.5 g, 18.0 mmol) in EtOH (20 mL) and H_2O (6 mL) were added $Pd(PPh_3)_4$ (1.5 g, 1.30 mmol) and K_2CO_3 (5.7 g, 41.2 mmol). The resulting mixture was sealed and degassed under vacuum and purged with N_2 three times, then stirred at 95 °C for 12 h under N_2 atmosphere. The resulting mixture was quenched with water (100 mL) and extracted with EtOAc (100 mL x 3). The combined organic layer was washed with saturated NH_4Cl aqueous solution (100 mL x 2), brine (100 mL), dried over anhydrous Na_2SO_4, filtered, and concentrated under reduced pressure. The residue was purified by flash chromatography (ISCO®; 25 g AgelaFlash® Silica Flash Column, petroleum ether / EtOAc with EtOAc from 0 ~ 50 %, flow rate = 30 mL / min) to afford 3-(furan-3-yl)-5-nitropyridin-2-amine (1.5 g, crude) as yellow solid. LCMS (ESI): [M+H]+ m/z: calcd 206.0, found 206.1.

tert-butyl (*tert*-butoxycarbonyl)(3-(furan-3-yl)-5-nitropyridin-2-yl)carbamate.

To a mixture of 3-(furan-3-yl)-5-nitropyridin-2-amine (1.5 g, 7.31 mmol) in DCM (15 mL) was added $(Boc)_2O$ (5 g, 22.9 mmol), DMAP (170 mg, 1.39 mmol), and TEA (3 mL, 21.5 mmol). The resulting mixture was stirred at 20 °C for 12 h. The resulting mixture was quenched with water (100 mL) and extracted with EtOAc (100 mL x 3). The combined organic layer was washed with saturated NH_4Cl aqueous solution (100 mL x 2), brine (100 mL), dried over anhydrous Na_2SO_4, filtered, and concentrated under reduced pressure. The residue was purified by flash chromatography (ISCO®; 25 g AgelaFlash® Silica Flash Column, petroleum ether/EtOAc with EtOAc from 0 ~ 20 %, flow rate = 30 mL / min) to afford *tert*-butyl (*tert*-butoxycarbonyl)(3-(furan-3-yl)-5-nitropyridin-2-yl)carbamate (1.6 g, 54% yield) as yellow solid. ^{1}H NMR (400 MHz, MeOD-*d₄*) δ 9.23 (d, *J* = 2.6 Hz, 1H), 8.79 (d, *J* = 2.6 Hz, 1H), 7.94 (s, 1H), 7.73 (t, *J* = 1.7 Hz, 1H), 6.73 - 6.88 (m, 1H), 1.31 (s, 18H). LCMS (ESI): [M+H]+ m/z: calcd 406.2, found 406.1.

Rac-tert-butyl (5-amino-3-(tetrahydrofuran-3-yl)pyridin-2-yl)(*tert*-butoxycarbonyl)carbamate.
A mixture of *tert*-butyl (*tert*-butoxycarbonyl)(3-(furan-3-yl)-5-nitropyridin-2-yl)carbamate (500 mg, 1.23 mmol), MeOH (6 mL), AcOH (2 mL, 1.23 mmol) and Pd/C (100 mg, 0.494 mmol, 10 % of Pd with 50 % of water, wt %) was stirred at 50 °C for 12 h under H_2 (balloon). The resulting mixture was filtered and concentrated under reduced pressure to give *tert*-butyl (5-amino-3-(tetrahydrofuran-3-yl)pyridin-2-yl)(*tert*-butoxycarbonyl)carbamate (600 mg, crude, 2•AcOH) as yellow solid. ^{1}H NMR (400 MHz, MeOD-d_4) δ 7.69 (d, *J* = 2.8 Hz, 1H), 7.14 (d, *J* = 2.8 Hz, 1H), 4.09 (td, *J* = 8.4, 4.8 Hz, 1H), 3.99 (t, *J* = 7.9 Hz, 1H), 3.84 - 3.92 (m, 1H), 3.68 (dd, *J* = 8.5, 6.0 Hz, 1H), 2.30 - 2.40 (m, 1H), 1.92 - 1.97 (m, 2H), 1.39 (d, *J* = 4.5 Hz, 18H). LCMS (ESI): [M+H]+ m/z: calcd 380.2, found 380.2.

Rac-benzyl 2-((6-(bis(*tert*-butoxycarbonyl)amino)-5-(tetrahydrofuran-3-yl)pyridin-3-yl)amino)-2-oxoacetate.
A mixture of *rac-tert*-butyl (5-amino-3-(tetrahydrofuran-3-yl)pyridin-2-yl)(*tert*-butoxycarbonyl)carbamate (600 mg, 1.20 mmol, 2•AcOH), benzyl 2-chloro-2-oxo-acetate (360 mg, 1.81 mmol), and DIPEA (1 mL, 5.74 mmol) in MeCN (10 mL) was stirred at 20 °C for 12 h. The resulting mixture was quenched with water (50 mL) and extracted with EtOAc (100 mL x 3). The combined organic layer was washed with saturated NH_4Cl aqueous solution (100 mL), brine (100 mL), dried over anhydrous Na_2SO_4, filtered, and concentrated under reduced pressure. The residue was purified by flash chromatography (ISCO®; 25 g AgelaFlash® Silica Flash Column, petroleum ether / EtOAc with EtOAc from 0 ~ 30 %, flow rate = 30 mL / min) to afford *rac*-benzyl 2-((6-(bis(*tert*-butoxycarbonyl)amino)-5-(tetrahydrofuran-3-yl)pyridin-3-yl)amino)-2-oxoacetate (300 mg, 46% yield) as white solid. LCMS (ESI): [M+H]+ m/z: calcd 542.2, found 542.3.

Rac-2-((6-(bis(tert-butoxycarbonyl)amino)-5-(tetrahydrofuran-3-yl)pyridin-3-yl)amino)-2-oxoacetic acid, **Intermediate 5a**.
To a mixture of *rac*-benzyl 2-((6-(bis(*tert*-butoxycarbonyl)amino)-5-(tetrahydrofuran-3-yl)pyridin-3-yl)amino)-2-oxoacetate (300 mg, 0.554 mmol) in EtOAc (6 mL) was added TEA (1.65 mmol, 0.23 mL) and Pd/C (100 mg, 0.494 mmol, 10% of Pd with 50 % of water, wt %). The resulting mixture was sealed and degassed under vacuum and purged with H_2 three times, then stirred at 20 °C for 12 h under H_2 (balloon). The resulting mixture was filtered and concentrated under reduced pressure to give *rac*-2-((6-(bis(tert-butoxycarbonyl)amino)-5-(tetrahydrofuran-3-yl)pyridin-3-yl)amino)-2-oxoacetic acid, **Intermediate 5a** (250 mg, crude, Et_3N) as white solid. ^{1}H NMR (400 MHz, MeOD-d_4) δ 8.75 (d, *J* = 2.5 Hz, 1H), 8.35 (d, *J* = 2.3 Hz, 1H), 4.08 - 4.19 (m, 1H), 4.04 (t, *J* = 7.9 Hz, 1H), 3.90 (q, *J* = 7.8 Hz, 1H), 3.72 (dd, *J* = 8.5, 6.0 Hz, 1H), 3.37 - 3.49 (m, 1H), 2.32 - 2.49 (m, 1H), 1.97 - 2.07 (m, 1H), 1.39 (s, 18H). LCMS (ESI): [M+H]+ m/z: calcd 452.2, found 452.2.

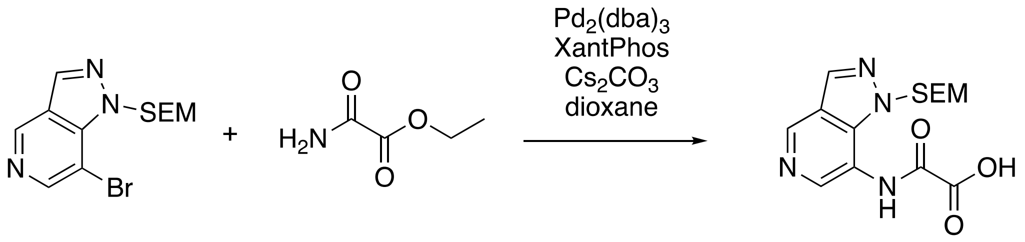

Intermediate 62a

2-oxo-2-((1-((2-(trimethylsilyl)ethoxy)methyl)-1*H*-pyrazolo[4,3-*c*]pyridin-7-yl)amino)acetic acid, **Intermediate 62a**.
To a mixture of 2-[(7-bromopyrazolo[4,3-*c*]pyridin-1-yl)methoxy]ethyl-trimethyl-silane (420 mg, 1.28 mmol) and ethyl 2-amino-2-oxo-acetate (450 mg, 3.84 mmol) in dioxane (10 mL) were added Cs_2CO_3 (630 mg, 1.93 mmol), XantPhos (126 mg, 0.253 mmol), and $Pd_2(dba)_3$ (110 mg, 0.120 mmol). The resulting mixture was sealed, degassed under vacuum, purged with N_2 three times, then stirred at 100 °C for 12 h under N_2 atmosphere. The resulting mixture was quenched by addition of water (50 mL) and extracted with EtOAc (100 mL x 2). The combined water layer was adjusted to pH = 5 with 2 N HCl/H_2O, and the mixture was extracted with DCM (100 mL x 5). The combined water layer was washed with brine (50 mL), dried over anhydrous Na_2SO_4, filtered, and concentrated under reduced pressure to give crude product, which was purified by preparative HPLC (Instrument: Gilson GX-281 Liquid Handler, Gilson 322 Pump, Gilson 156 UV Detector; Column: Welch Xtimate C18 150 x 25 mm x 5 μm; Mobile phase A: H_2O with 0.225% FA (v%); Mobile phase B: MeCN; Gradient: B from 18% to 48% in 7.8 min, hold 100% B for 2 min; Flow Rate: 25 mL/min; Column Temperature: 30 °C; Wavelength: 220 nm, 254 nm) to afford 2-oxo-2-((1-((2-(trimethylsilyl)ethoxy)methyl)-1*H*-pyrazolo[4,3-*c*]pyridin-7-yl)amino)acetic acid, **Intermediate 62a** (60 mg, 14% yield) as yellow solid. LCMS (ESI): [M+H]+ m/z: calcd 337.1, found 337.0.

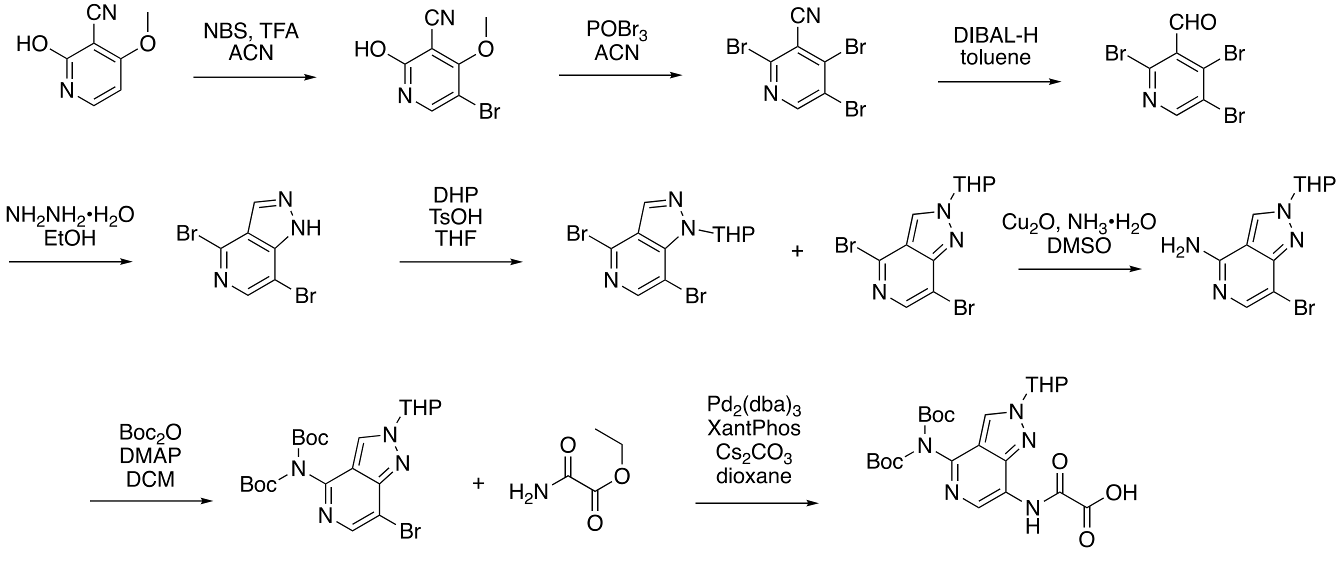

Intermediate 39a

5-bromo-2-hydroxy-4-methoxynicotinonitrile.
To a solution of 2-hydroxy-4-methoxy-pyridine-3-carbonitrile (50 g, 0.333 mol) and NBS (70 g, 0.393 mol) in ACN (200 mL) was added TFA (6.5 mL, 0.0844 mol). The mixture was stirred at 80 °C for 12 h. The resulting mixture was filtered and the filter cake was concentrated under reduced pressure to afford 5-bromo-2-hydroxy-4-methoxynicotinonitrile (70 g, 92% yield) as off-white solid. LCMS (ESI): [M+H]+ m/z: calcd 231.1, found 231.0.

2,4,5-tribromonicotinonitrile.
To a solution of 5-bromo-2-hydroxy-4-methoxynicotinonitrile (30 g, 0.131 mol) in ACN (100 mL) was added $POBr_3$ (75 g, 0.262 mol). The mixture was stirred at 80 °C for 4 h. The resulting mixture was quenched with water (50 mL) and extracted with EtOAc (50 mL x 3). The combined organic layer was washed with saturated $NaHCO_3$ aqueous solution (50 mL x 2), brine (50 mL), dried over anhydrous Na_2SO_4, filtered, and concentrated under reduced pressure to give a residue which was purified by flash chromatography (ISCO®; 40 g AgelaFlash® Silica Flash Column, petroleum ether / EtOAc with EtOAc from 0 ~ 30 %, flow rate = 30 mL / min, 254 nm) to afford 2,4,5-tribromonicotinonitrile (25 g, 56% yield) as a solid.

2,4,5-tribromonicotinaldehyde.
To a solution of 2,4,5-tribromonicotinonitrile (25 g, 0.0733 mol) in toluene (60 mL) was added DIBAL-H (1 M solution in toluene, 110 mL) at -40 °C. The mixture was stirred at -40 °C for 1 h, then it was quenched with 1 M HCl aqueous solution (100 mL) and stirred at 20 °C for 1 h. The resulting mixture was adjusted to pH = 7 with saturated NaOH aqueous solution, and extracted with EtOAc (100 mL x 3). The combined organic layer was washed with saturated NH_4Cl aqueous solution (100 mL x 2), brine (100 mL), dried over anhydrous Na_2SO_4, filtered, and concentrated under reduced pressure to give 2,4,5-tribromonicotinaldehyde (20 g, 79% yield) as yellow solid. ^1H NMR (400 MHz, DMSO-d_6) δ 10.03 (s, 1 H), 8.82 (s, 1 H).

4,7-dibromo-1*H*-pyrazolo[4,3-*c*]pyridine.
To a solution of 2,4,5-tribromonicotinaldehyde (20 g, 58.2 mmol) in EtOH (200 mL) was added hydrazine hydrate (4 mL, 70.5 mmol, 85% purity). The mixture was stirred at 80 °C for 12 h. The resulting mixture was quenched with water (100 mL) and extracted with EtOAc (100 mL x 3). The combined organic layer was washed with saturated NH_4Cl aqueous solution (100 mL x 2), brine (100 mL), dried over anhydrous Na_2SO_4, filtered, and concentrated under reduced pressure to give 4,7-dibromo-1*H*-pyrazolo[4,3-*c*]pyridine (14 g, 87% yield) as a yellow solid. LCMS (ESI): [M+H]+ m/z: calcd 277.9, found 277.7.

4,7-dibromo-1-(tetrahydro-2*H*-pyran-2-yl)-1*H*-pyrazolo[4,3-*c*]pyridine and 4,7-dibromo-2-(tetrahydro-2*H*-pyran-2-yl)-2*H*-pyrazolo[4,3-*c*]pyridine.
To a solution of 4,7-dibromo-1*H*-pyrazolo[4,3-*c*]pyridine (4 g, 0.0144 mol) and 3,4-dihydro-2H-pyran (7 mL, 0.0771 mol) in THF (30 mL) was added TsOH (300 mg, 5.76 mmol). The mixture was stirred at 65 °C for 12 h.The resulting mixture was quenched with water (100 mL) and extracted with EtOAc (100 mL x 3). The combined organic layer was washed with saturated NH_4Cl aqueous solution (100 mL x 2), brine (100 mL), dried over anhydrous Na_2SO_4, filtered, and concentrated under reduced pressure. The residue was purified by flash chromatography (ISCO®; 20 g AgelaFlash® Silica Flash Column, petroleum ether / EtOAc with EtOAc from 0 ~ 20 %, flow rate =30 mL / min,254 nm) to afford 4,7-dibromo-1-(tetrahydro-2*H*-pyran-2-yl)-1*H*-pyrazolo[4,3-*c*]pyridine (2.8 g, 54% yield) and 4,7-dibromo-2-(tetrahydro-2*H*-pyran-2-yl)-2*H*-pyrazolo[4,3-*c*]pyridine (1.7 g, 33% yield) as yellow solid. LCMS (ESI): [M+H]+ m/z: calcd 361.9, found 362.0.

7-bromo-2-(tetrahydro-2*H*-pyran-2-yl)-2*H*-pyrazolo[4,3-*c*]pyridin-4-amine.
To a solution of 4,7-dibromo-2-tetrahydropyran-2-yl-pyrazolo[4,3-*c*]pyridine (2 g, 5.54 mmol) and ammonium hydroxide (900 mg, 0.0257 mol, 1.00 mL) in DMSO (6 mL) was added Cu_2O (80 mg, 0.559 mmol). The mixture was stirred at 100 °C for 12 h. The resulting mixture was quenched with water (20 mL) and extracted with EtOAc (50 mL x 3). The combined organic layer was washed with saturated NH_4Cl aqueous solution (20 mL x 2), brine (20 mL), dried over anhydrous Na_2SO_4, filtered, and concentrated under reduced pressure to afford 7-bromo-2-(tetrahydro-2*H*-pyran-2-yl)-2*H*-pyrazolo[4,3-*c*]pyridin-4-amine (1.3 g, crude) as a yellow oil. LCMS (ESI): [M+H]+ m/z: calcd 297.0, found 296.9.

tert-butyl (7-bromo-2-(tetrahydro-2*H*-pyran-2-yl)-2*H*-pyrazolo[4,3-*c*]pyridin-4-yl)(*tert*-butoxycarbonyl)carbamate.
To a solution of 7-bromo-2-(tetrahydro-2*H*-pyran-2-yl)-2*H*-pyrazolo[4,3-*c*]pyridin-4-amine (1.3 g, 4.37 mmol) and DMAP (550 mg, 4.50 mmol) in DCM (6 mL) was added *tert*-butoxycarbonyl *tert*-butyl carbonate (2 mL, 8.75 mmol). The mixture was stirred at 20 °C for 12 h. The resulting mixture was quenched

with water (50 mL) and extracted with EtOAc (50 mL x 3). The combined organic layer was washed with saturated NH_4Cl aqueous solution (50 mL x 2), brine (50 mL), dried over anhydrous Na_2SO_4, filtered, and concentrated under reduced pressure to give a residue which was purified by flash chromatography (ISCO®; 20 g AgelaFlash® Silica Flash Column, petroleum ether / EtOAc with EtOAc from 0 ~ 20 %, flow rate =30 mL / min, 254 nm) to afford *tert*-butyl (7-bromo-2-(tetrahydro-2*H*-pyran-2-yl)-2*H*-pyrazolo[4,3-*c*]pyridin-4-yl)(*tert*-butoxycarbonyl)carbamate (1 g, 46% yield) as a white solid. LCMS (ESI): [M+H]+ m/z: calcd 499.1, found 499.3.

2-((4-(bis(*tert*-butoxycarbonyl)amino)-2-(tetrahydro-2*H*-pyran-2-yl)-2*H*-pyrazolo[4,3-*c*]pyridin-7-yl)amino)-2-oxoacetic acid, **Intermediate 39a**.
To a solution of *tert*-butyl (7-bromo-2-(tetrahydro-2*H*-pyran-2-yl)-2*H*-pyrazolo[4,3-*c*]pyridin-4-yl)(*tert*-butoxycarbonyl)carbamate (200 mg, 0.402 mmol), ethyl 2-amino-2-oxo-acetate (200 mg, 1.71 mmol), XantPhos (60 mg, 0.104 mmol), and $Pd_2(dba)_3$ (40 mg, 0.0437 mmol) in dioxane (10 mL) was added Cs_2CO_3 (400 mg, 1.23 mmol). The resulting mixture was sealed and degassed under vacuum and purged with N_2 three times, then stirred at 130 °C for 2 h under microwave. The resulting mixture was filtered and concentrated under reduced pressure to give a residue which was purified by flash chromatography (Biotage®, Column: SepaFlash® Sphercial C18, 40 g, 40 - 60 μm, 120 Å; MeCN / water (0.05 % NH_3-H_2O) with MeCN from 0 ~ 30 %, 30 mL / min, 254 nm) to afford 2-((4-(bis(*tert*-butoxycarbonyl)amino)-2-(tetrahydro-2*H*-pyran-2-yl)-2*H*-pyrazolo[4,3-*c*]pyridin-7-yl)amino)-2-oxoacetic acid (120 mg, 30% yield) as a white solid. LCMS (ESI): [M+H]+ m/z: calcd 506.2, found 506.3.

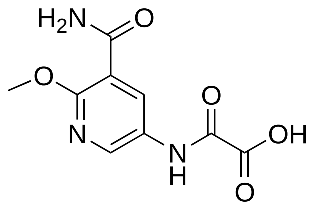

2-((5-carbamoyl-6-methoxypyridin-3-yl)amino)-2-oxoacetic acid, **Intermediate 48a**.
To a solution of 5-amino-2-methoxy-pyridine-3-carboxamide (10.06 g, 60.18 mmol, 1 eq) and TEA (8.39 mL, 60.18 mmol, 1 eq) in dry THF (250 mL) was added 2,2,2-trifluoroethyl 2- chloro-2-oxo-acetate (12.04 g, 63.19 mmol, 1.05 eq) in 50 mL dry THF at 0 °C. After stirring at room temperature for 12 h the resulting mixture was evaporated to dryness to give 2,2,2- trifluoroethyl 2-[(5-carbamoyl-6-methoxy-3-pyridyl)amino]-2-oxo-acetate (29 g, crude) as a light-pink solid, which was used in the next step without further purification. 1H NMR (DMSO-d_6, 400 MHz) δ 3.97 (s, 3H), 5.00 (q, 2H), 7.75 (brs, 2H), 8.55 (s, 1H), 8.63 (s, 1H), 11.21 (s, 1H). To a solution of this material (15 g, 46.70 mmol, 1 eq) in MeOH (400 mL) was added lithium hydroxide monohydrate (3.92 g, 93.40 mmol, 2 eq) and the resulting mixture was left to stir at room temperature for 1 h, after which the resulting mixture was evaporated to dryness and dissolved in H_2O which was then acidified to pH=1 with aqueous hydrochloric acid. The precipitate was filtered and then suspended in MeOH and TEA (13.02 mL, 93.40 mmol, 2 eq) was added until the solution became clear. The resulting mixture was evaporated to dryness to give -((5-carbamoyl-6-methoxypyridin-3-yl)amino)-2-oxoacetic acid, **Intermediate 48a** (10.35 g, 30.41 mmol, 65% yield, Et_3N salt) as a beige solid. ^{1}H NMR (400 MHz, DMSO-d_6) δ 1.17 (s, 12H), 3.06 (q, 6H), 3.92 (s, 3H), 7.70 (d, 2H), 8.60 (s, 1H), 10.34 (s, 1H), 10.34 (br s, 1H).

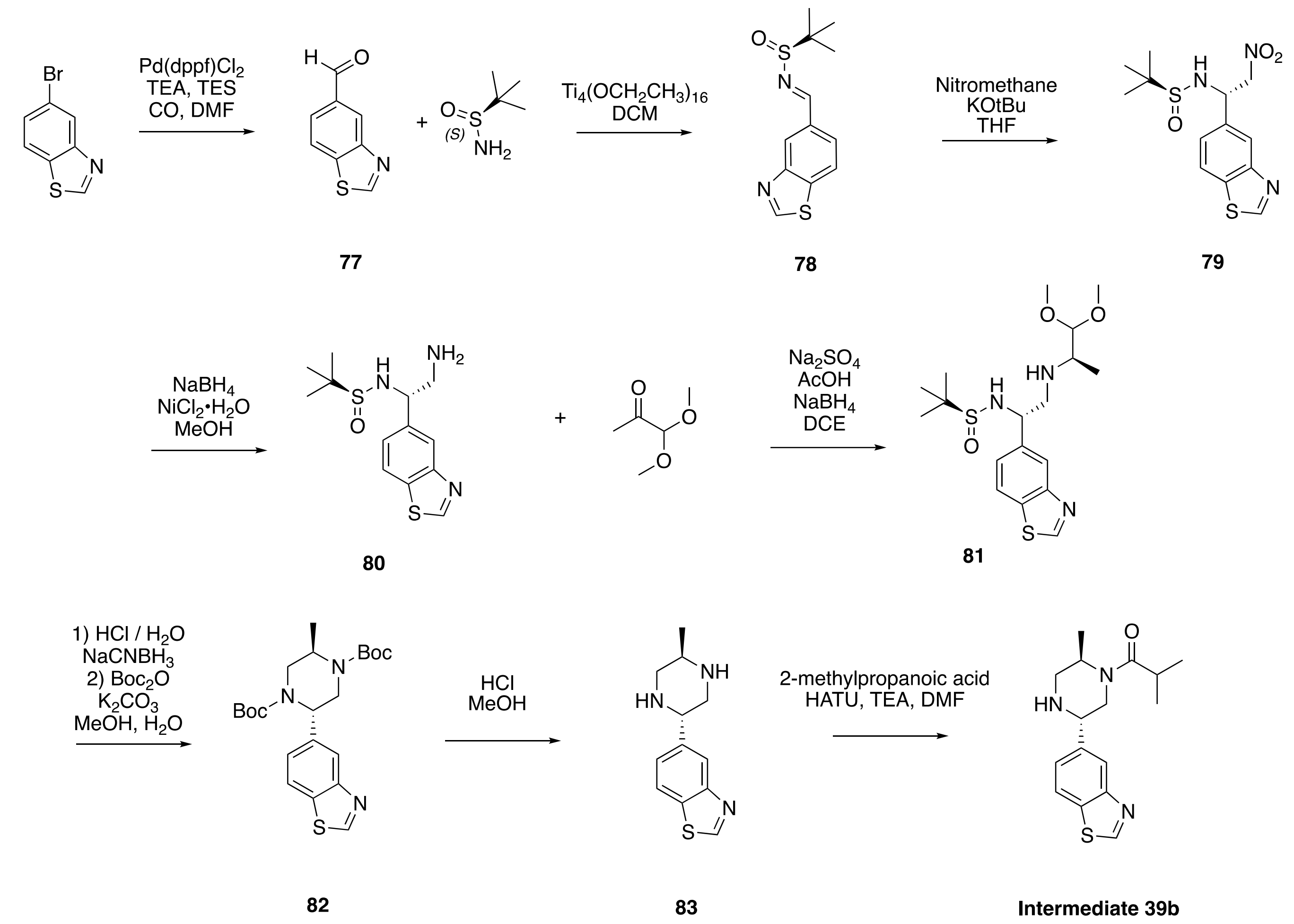

benzo[*d*]thiazole-5-carbaldehyde (**77**).
A mixture of 5-bromo-1,3-benzothiazole (25 g, 116.78 mmol), Pd(dppf)Cl_2 (4.37 g, 5.98 mmol), TEA (39.93 g, 394.60 mmol, 55.00 mL), TES (43.75 g, 376.26 mmol), and DMF (100 mL) was stirred at 80 °C for 12 h under CO (g) (50 psi). The resulting mixture was quenched with water (100 mL) and extracted with EtOAc (200 mL x 3). The combined organic layer was washed with saturated NH_4Cl aqueous solution (100 mL x 2), brine (100 mL), dried over anhydrous Na_2SO_4, filtered, and concentrated under reduced pressure to give residue which was purified by flash chromatography (ISCO®; 80 g AgelaFlash® Silica Flash Column, petroleum ether / EtOAc with EtOAc from 0 ~ 50 %, Flow Rate: 30 mL / min, to afford 1,3-benzothiazole-5-carbaldehyde (13 g, 79.66 mmol, 68 % yield) as yellow solid.

(*S,E*)-*N*-(benzo[*d*]thiazol-5-ylmethylene)-2-methylpropane-2-sulfinamide (**78**).
A mixture of **77** (10 g, 61.28 mmol), 2-methylpropane-2-sulfinamide (10.00 g, 82.51 mmol), titanium(IV) ethoxide (43.60 g, 191.14 mmol, 40 mL) and DCM (20 mL) was stirred at 20 °C for 12 h. The resulting mixture was quenched with water (100 mL) and extracted with DCM (100 mL x 3). The combined organic layer was washed with saturated NH_4Cl aqueous solution (100 mL x 2), brine (100 mL), dried over anhydrous Na_2SO_4, filtered, and concentrated under reduced pressure to afford (*S,E*)-*N*-(benzo[*d*]thiazol-5-ylmethylene)-2-methylpropane-2-sulfinamide, (10 g, 37.54 mmol, 61 % yield) as a yellow solid. LCMS (ESI) [M+H]+ m/z: calcd 267.0; found 267.0. ^{1}H NMR (400 MHz, MeOD-*d₄*) δ 9.39 (s, 1 H), 8.76 (s, 1 H), 8.58 (br s, 1 H), 8.19 - 8.32 (m, 1 H), 8.09 (br d, *J* = 8.1 Hz, 1 H), 1.32 (s, 10 H).

(*S*)-*N*-((*S*)-1-(benzo[*d*]thiazol-5-yl)-2-nitroethyl)-2-methylpropane-2-sulfinamide (**79**).
A mixture of potassium *tert*-butoxide (1 M, 200 mL) and THF (100 mL) was cooled to 0 °C. Nitromethane (47.46 g, 777.52 mmol, 42 mL) was added and the reaction was stirred for 1 h. (*S,E*)-*N*-(benzo[*d*]thiazol-5-ylmethylene)-2-methylpropane-2-sulfinamide, **78** (10 g, 37.54 mmol) was added at 0 °C and then stirred at 20 °C for 47 h. The mixture was concentrated under reduced pressure to give a residue which was purified by flash chromatography (ISCO®; 40g AgelaFlash® Silica Flash Column, EtOAc / MeOH with MeOH from 0 ~ 10 %, Flow Rate: 30 mL / min) to afford (*S*)-*N*-((*S*)-1-(benzo[*d*]thiazol-5-yl)-2-nitroethyl)-2-methylpropane-2-sulfinamide **79** (8 g, 24.43 mmol, 65 % yield) as yellow oil. ^{1}H NMR (400 MHz, MeOD-*d₄*) δ 9.31 (s, 1 H), 8.08 - 8.21 (m, 2 H), 7.58 (dd, *J* = 8.4, 1.6 Hz, 1 H), 5.31 - 5.40 (m, 1 H), 5.06 - 5.15 (m, 1 H), 4.98 (dd, *J* = 13.4, 6.3 Hz, 1 H), 1.21 (s, 9 H). LCMS (ESI): [M+H]+ m/z: calcd 328.1, found 328.1

(*S*)-*N*-((*S*)-2-amino-1-(benzo[*d*]thiazol-5-yl)ethyl)-2-methylpropane-2-sulfinamide (**80**).
A mixture of (*S*)-*N*-((*S*)-1-(benzo[*d*]thiazol-5-yl)-2-nitroethyl)-2-methylpropane-2-sulfinamide, **79** (7.5 g, 22.91 mmol), nickel(II) chloride hexahydrate (6 g, 25.24 mmol), and MeOH (50 mL) was cooled to 0 °C. $NaBH_4$ (4.4 g, 116.30 mmol) was added slowly and stirred at 0 °C for 2 h. The mixture was filtered and concentrated under reduced pressure to give (*S*)-*N*-((*S*)-2-amino-1-(benzo[*d*]thiazol-5-yl)ethyl)-2-methylpropane-2-sulfinamide **80** (7.5 g, crude) as black oil that was used as is. LCMS (ESI): [M+H]+ m/z: calcd 298.1, found 298.0.

(*S*)-*N*-((*S*)-1-(benzo[*d*]thiazol-5-yl)-2-(((*R*)-1,1-dimethoxypropan-2-yl)amino)ethyl)-2-methylpropane-2-sulfinamide (**81**).

A mixture of (*S*)-*N*-((*S*)-2-amino-1-(benzo[*d*]thiazol-5-yl)ethyl)-2-methylpropane-2-sulfinamide, **80** (7.5 g, 25.22 mmol), 1,1-dimethoxypropan-2-one (8 g, 67.72 mmol), Na_2SO_4 (13 g, 91.52 mmol), AcOH (1.50 g, 30.74 mmol), and DCE (100 mL) was stirred at 20 °C for 12 h. $NaBH_4$ (3.00 g, 79.18 mmol) was added and the mixture was stirred at 20 °C for 1 h. The resulting mixture was quenched by addition of water (50 mL) and extracted with DCM (100 mL x 3). The combined organic layer was washed with saturated NH_4Cl aqueous solution (50 mL x 2), brine (50 mL), dried over anhydrous Na_2SO_4, filtered, and concentrated under reduced pressure to give (*S*)-*N*-((*S*)-1-(benzo[*d*]thiazol-5-yl)-2-(((*R*)-1,1-dimethoxypropan-2-yl)amino)ethyl)-2-methylpropane-2-sulfinamide, **81** (11 g, crude) as yellow oil. LCMS (ESI): [M+H]+ m/z: calcd 400.2, found 400.1.

di-*tert*-butyl (2*S*,5*R*)-2-(benzo[*d*]thiazol-5-yl)-5-methylpiperazine-1,4-dicarboxylate (**82**).

A mixture of (*S*)-*N*-((*S*)-1-(benzo[*d*]thiazol-5-yl)-2-(((*R*)-1,1-dimethoxypropan-2-yl)amino)ethyl)-2-methylpropane-2-sulfinamide, **81** (4.5 g, 11.3 mmol) and HCl / H_2O (12 M, 12 mL) was stirred at 20 °C for 1 h after which the mixture was concentrated under reduced pressure to give a residue which was diluted with MeOH (30 mL). $NaBH_3(CN)$ (700 mg, 11.1 mmol), Na_2SO_4 (5.4 g, 38.0 mmol), and MeOH (30 mL) were added and the mixture was stirred at 20 °C for 12 h. Boc_2O (8.56 g, 39.2 mmol), K_2CO_3 (4.8 g, 34.7 mmol), and H_2O (15 mL) were added and the mixture was stirred at 20 °C for 2 h. The resulting mixture was quenched by addition of water (100 mL) and extracted with EtOAc (100 mL x 3). The combined organic layer was washed with brine (100 mL), dried over anhydrous Na_2SO_4, filtered, and concentrated under reduced pressure to give a residue which was purified by flash chromatography (ISCO®; 12g AgelaFlash® Silica Flash Column, petroleum ether/EtOAc with EtOAc from 0 ~ 30 %, Flow Rate: 30 mL / min, 254 nm) to afford di-*tert*-butyl (2*S*,5*R*)-2-(benzo[*d*]thiazol-5-yl)-5-methylpiperazine-1,4-dicarboxylate, **82** (0.65 g, 13 % yield) as yellow oil. LCMS (ESI): [M+H]+ m/z: calcd 434.2, found 234.3.

5-((2*S*,5*R*)-5-methylpiperazin-2-yl)benzo[*d*]thiazole (**83**).

A mixture of di-*tert*-butyl (2*S*,5*R*)-2-(benzo[*d*]thiazol-5-yl)-5-methylpiperazine-1,4-dicarboxylate, **82** (1.00 g, 2.31 mmol) and MeOH / HCl (4 M, 5 mL) was stirred at 20 °C for 2 h. The mixture was concentrated under reduced pressure to give 5-((2*S*,5*R*)-5-methylpiperazin-2-yl)benzo[*d*]thiazole, **83** (560 mg, crude, 2HCl) as yellow solid. LCMS (ESI): [M+H]+ m/z: calcd 234.1, found 234.2

1-((2*R*,5*S*)-5-(benzo[*d*]thiazol-5-yl)-2-methylpiperazin-1-yl)-2-methylpropan-1-one, **Intermediate 39b**.

To a solution of **83** (100 mg, 0.327 mmol, 2•HCl) in DMF (3 mL) was added HATU (140 mg, 0.368 mmol) and TEA (0.200 mL, 1.43 mmol). 2-methylpropanoic acid (30 mg, 0.341 mmol) in DMF (2 mL)) was added dropwise at -30 °C. The resulting mixture was stirred at 20 °C for 2 h. The mixture was concentrated and purified by flash chromatography (Column: SepaFlash® Sphercial C18, 15 g, 40 - 60 μm, 120 Å; MeCN / water (0.05 v % NH_3-H_2O) with MeCN from 0 - 30%, 25 mL / min, 254 nm) to afford 1-((2*R*,5*S*)-5-(benzo[*d*]thiazol-5-yl)-2-methylpiperazin-1-yl)-2-methylpropan-1-one, **Intermediate 39b** (70 mg, crude) as a white solid. LCMS (ESI): [M+H]+ m/z: calcd 304.1, found 304.2.

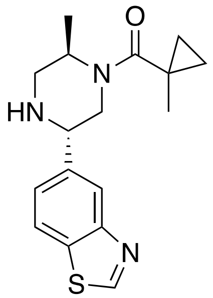

((2*R*,5*S*)-5-(benzo[*d*]thiazol-5-yl)-2-methylpiperazin-1-yl)(1-methylcyclopropyl)methanone, **Intermediate 40b**.

To a mixture of **Intermediate 39b'** (100 mg, 0.327 mmol, 2HCl) in DMF (2 mL) was added HATU (190 mg, 0.499 mmol) and TEA (225 μL, 1.61 mmol). 1-methylcyclopropanecarboxylic acid (30 mg, 0.299 mmol) in DMF (2mL) was then added at -20 °C. The resulting mixture was stirred at -20 °C for 2 h. The mixture was concentrated and the residue was purified by flash chromatography (Column: SepaFlash® Sphercial C18, 40 g, 40-60 μm, 120 Å; MeCN / water (0.05 v% NH_3-H_2O) with MeCN from 0 – 30 %, 40 mL / min, 254 nm) to afford ((2*R*,5*S*)-5-(benzo[*d*]thiazol-5-yl)-2-methylpiperazin-1-yl)(1-methylcyclopropyl)methanone, **Intermediate 40b** (40 mg, 39% yield) as yellow solid. LCMS (ESI): [M+H]+ m/z: calcd 316.1, found 316.3.

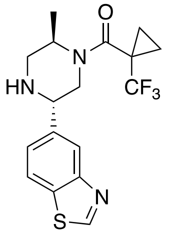

((2*R*,5*S*)-5-(benzo[*d*]thiazol-5-yl)-2-methylpiperazin-1-yl)(1-(trifluoromethyl)cyclopropyl)methanone, **Intermediate 41b**.

To a solution of **83** (100 mg, 0.327 mmol, 2HCl) in DMF (3 mL) was added, HATU (148 mg, 0.389 mmol) and TEA (1.61 mmol, 0.225 mL). 1-(trifluoromethyl)cyclopropanecarboxylic acid (55 mg, 0.357 mmol) in DMF (2 mL) was added at -30 °C. The resulting mixture was stirred at 20 °C for 12 h. The mixture was concentrated and purified by flash chromatography (Column: SepaFlash® Sphercial C18, 40 g, 40 - 60 μm, 120 Å; MeCN / water (0.05 v %

NH_3-H_2O) with MeCN from 0 – 30 %, 40 mL / min, 254 nm) to afford ((2*R*,5*S*)-5-(benzo[*d*]thiazol-5-yl)-2-methylpiperazin-1-yl)(1-(trifluoromethyl)cyclopropyl)methanone, **Intermediate 41b** (60 mg, 50% yield) as a white solid. LCMS (ESI): [M+H]+ m/z: calcd 370.1, found 370.2.

Compounds 2, 3, 5, 22, 24 – 33, 39 – 41, 46 – 56, 58, 62

General Procedure A

The appropriate piperidine, piperazine, or morpholine (1 eq), oxamic acid (1 eq) and TEA (2.5 eq + 1.0 eq per each acid eq, if amine salt used) were stirred in DMF. HATU (1.5 eq) was added and the resulting mixture was stirred overnight. The reaction mixture was concentrated under vacuum and the residue was purified by HPLC to obtain the desired product. When appropriate, the material was deprotected as indicated and/or isomers were separated by chiral SFC or HPLC.

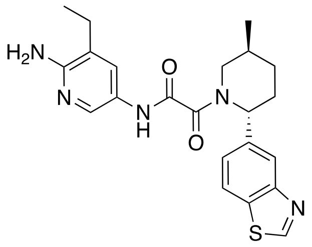

N-(6-amino-5-ethylpyridin-3-yl)-2-((2*R*,5*S*)-2-(benzo[*d*]thiazol-5-yl)-5-methylpiperidin-1-yl)-2-oxoacetamide, **Compound 2**.
Amine: **69**. Acid: **Intermediate 2a**. Yield: 53 %. HPLC purification (column: YMC-Actus Triart C18, 100 x 20 mm, 5 μm; 35 – 60 % 0 - 5 min H_2O / ACN /0.1 % NH_4OH, flow: 30 mL / min as mobile phase then another column: Chromatorex 18 SMB 100-5T 100 x 19 mm, 5 μm; 30 – 60 % 0 – 5 min H_2O / MeOH / 0.1 % FA, flow: 30 mL / min as mobile phase) to afford *N*-(6-amino-5-ethylpyridin-3-yl)-2-((2*R*,5*S*)-2-(benzo[*d*]thiazol-5-yl)-5-methylpiperidin-1-yl)-2-oxoacetamide, **Compound 2**. [α]21D = +135.0° (c = 0.1 g/100 mL EtOH). ^{1}H NMR (600 MHz, DMSO-d_6) δ 1.04 (t, 6H), 1.36 (m, 1H), 1.72 (m, 1H), 1.87 (m, 1H), 2.13 (m, 1H), 2.35 (m, 2H), 3.78 (m, 3H), 4.33 (s, 1H), 5.66 (m, 3H), 7.43 (d, 1H), 7.51 (d, 1H), 8.00 (d, 1H), 8.06 (d, 1H), 8.17 (m, 1H), 9.40 (s, 1H), 10.56 (m, 1H). LCMS (ESI): [M+1]+ m/z: calcd 423.2; found 424.2; Rt = 2.082 min.

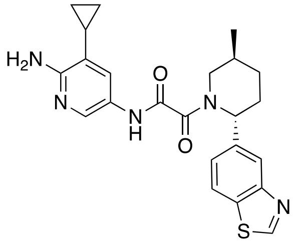

N-(6-amino-5-cyclopropylpyridin-3-yl)-2-((2*R*,5*S*)-2-(benzo[*d*]thiazol-5-yl)-5-methylpiperidin-1-yl)-2-oxoacetamide, **Compound 3**.
Amine: **69**. Acid: **Intermediate 3a**. Yield: 33 %. HPLC (0.5 - 6.5 min 40 - 65% water - ACN; flow: 30 mL / min, column: Waters SunFire C18, 100 x 19 mm, 5 μm) to afford a brown solid.
Further purification on chiral columns to remove trace enantiomer gave 100% de and 100% ee.
Column: ChiralART YMC (250 x 20 mm, 5 μm); Mobile phase: Hexane-IPA-MeOH, 50-25-25. Flow Rate: 12 mL / min, RT = 36.76 min
Analytical: Chiralpak IC (250 x 4.6, 5 μm), CO_2-MeOH, 50-50, 2.0 mL / min, RT = 12.095 min
N-(6-amino-5-cyclopropylpyridin-3-yl)-2-((2*R*,5*S*)-2-(benzo[*d*]thiazol-5-yl)-5-methylpiperidin-1-yl)-2-oxoacetamide, **Compound 3,** ^{1}H NMR (600 MHz, DMSO-d_6) δ 0.44 (m, 2H), 0.86 (m, 2H), 1.02 (m, 3H), 1.35 (m, 1H), 1.65 (m, 2H), 1.88 (m, 1H), 2.15 (m, 1H), 2.31 (m, 1H), 2.79 (m, 1H), 3.78 (m, 1H), 5.64 (m, 3H), 7.33 (m, 1H), 7.46 (m, 1H), 8.02 (m, 2H), 8.17 (m, 1H), 9.38 (m, 1H), 10.52 (m, 1H)
LCMS (ESI): [M+H]+ m/z: calcd 436.2; found 436.2; Rt = 2.006.

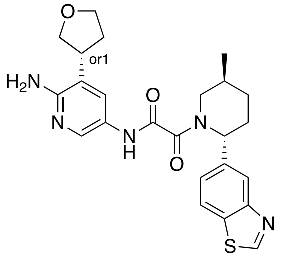

N-(6-amino-5-((*R**)-tetrahydrofuran-3-yl)pyridin-3-yl)-2-((2*R*,5*S*)-2-(benzo[*d*]thiazol-5-yl)-5-methylpiperidin-1-yl)-2-oxoacetamide, **Compound 5**.
Amine: **69**. Acid: **Intermediate 5a**. Yield: 91 %. Flash chromatography (ISCO®; 4 g AgelaFlash® Silica Flash Column, petroleum ether / EtOAc with EtOAc from 0 ~ 65 %, flow rate = 30 mL / min) to afford *tert*-butyl (5-(2-((2*R*,5*S*)-2-(benzo[*d*]thiazol-5-yl)-5-methylpiperidin-1-yl)-2-oxoacetamido)-3-(tetrahydrofuran-3-yl)pyridin-2-yl)(*tert*-butoxycarbonyl)carbamate as a white solid. LCMS (ESI) [M+H]+ m/z: calcd 666.3, found 666.3. The material (110 mg, 0.165 mmol) was stirred in DCM (3 mL) and TFA (2 mL, 26.0 mmol) was added at 20 °C and stirred for 2 h. The mixture was concentrated under reduced pressure to give a crude product, which was purified by preparative HPLC (Instrument: Gilson GX-281 Liquid Handler, Gilson 322 Pump, Gilson

156 UV Detector; Column: Phenomenex Gemini-NX, 80 x 40 mm x 3 μm; Mobile phase A: H_2O with 10 mmol NH_4HCO_3 (v %); Mobile phase B: MeCN; Gradient: B from 22 % to 52 % in 9.5 min, hold 100 % B for 2 min; Flow Rate: 25 mL / min; Column Temperature: 30° C; Wavelength: 220 nm, 254 nm) to afford *N*-(6-amino-5-(tetrahydrofuran-3-yl)pyridin-3-yl)-2-((2*R*,5*S*)-2-(benzo[*d*]thiazol-5-yl)-5-methylpiperidin-1-yl)-2-oxoacetamide (40 mg, 52.0% yield) as a white solid. LCMS (ESI) [M+H]+ m/z: calcd 466.2, found 466.2. HPLC: 100% at 220 nm, HPLC: 100 % at 254 nm.

Chiral separation: SFC (Instrument: Acssh-CO; Column: YMC-IB, 250 mm × 30 mm ×10 μm; Mobile phase: Hexane-IPA (0.1 % NH_3 v %) = 70 /30; Flow Rate: 80 mL / min; Column Temperature: 38 °C; Nozzle Pressure: 100 bar; Nozzle Temperature: 60 °C; Evaporator Temperature: 20 °C; Trimmer Temperature: 25 °C; Wavelength: 220 nm). The fraction was concentrated under reduced pressure and then lyophilized overnight to give *N*-(6-amino-5-((*R**)-tetrahydrofuran-3-yl)pyridin-3-yl)-2-((2*R*,5*S*)-2-(benzo[*d*]thiazol-5-yl)-5-methylpiperidin-1-yl)-2-oxoacetamide, **Compound 5** and *N*-(6-amino-5-((*S**)-tetrahydrofuran-3-yl)pyridin-3-yl)-2-((2*R*,5*S*)-2-(benzo[*d*]thiazol-5-yl)-5-methylpiperidin-1-yl)-2-oxoacetamide, with stereochemistry at the THF assigned arbitrarily.

N-(6-amino-5-((*R**)-tetrahydrofuran-3-yl)pyridin-3-yl)-2-((2*R*,5*S*)-2-(benzo[*d*]thiazol-5-yl)-5-methylpiperidin-1-yl)-2-oxoacetamide, **Compound 5** (9.3 mg, Peak 1, retention time: 3.791 min, white solid). ^{1}H NMR (400 MHz, methanol-d_4) δ 9.26 (s, 1H), 7.95 - 8.25 (m, 3H), 7.66 - 7.86 (m, 1H), 7.39 - 7.64 (m, 1H), 5.48 - 5.96 (m, 1H), 3.96 - 4.22 (m, 2H), 3.68 - 3.92 (m, 3H), 3.44 (br s, 2H), 2.37 (br s, 3H), 1.89 - 2.10 (m, 3H), 1.47 (br d, *J* = 19.8 Hz, 1H), 1.15 (d, *J* = 6.8 Hz, 3H). LCMS (ESI) [M+H]+ m/z: calcd 466.2, found 466.2; HPLC: 97.95 % at 220 nm, 99.21 % at 254 nm; 99.9 % ee. *N*-(6-amino-5-((*S**)-tetrahydrofuran-3-yl)pyridin-3-yl)-2-((2*R*,5*S*)-2-(benzo[*d*]thiazol-5-yl)-5-methylpiperidin-1-yl)-2-oxoacetamide, (10.2 mg, Peak 2, Retention time: 4.236 min, white solid). ^{1}H NMR (400 MHz, MeOD-d_4) δ 9.26 (s, 1H), 7.97 - 8.26 (m, 3H), 7.69 - 7.91 (m, 1H), 7.41 - 7.65 (m, 1H), 5.48 - 5.95 (m, 1H), 4.04 (br s, 2H), 3.65 - 3.93 (m, 3H), 3.44 (br s, 2H), 2.36 (br s, 3H), 1.88 - 2.09 (m, 3H), 1.43 - 1.58 (m, 1H), 1.15 (br d, *J* = 7.0 Hz, 3H). LCMS (ESI): [M+H]+ m/z: calcd 466.2, found 466.2; HPLC: 98.30 % at 220 nm, 100% at 254nm; 99.3% ee.

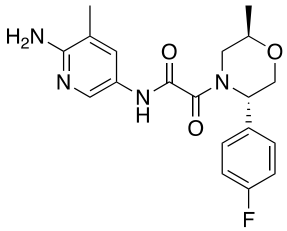

N-(6-amino-5-methylpyridin-3-yl)-2-((2*R*,5*S*)-5-(4-fluorophenyl)-2-methylmorpholino)-2-oxoacetamide, **Compound 22**.

Amine: **Intermediate 22a**. Acid: **Intermediate 23acid**. Yield: 19 %. Boc protected: LCMS (ESI): [M]+ m/z: calcd 472.2; found 473.2; Rt = 1.325 min. *tert*-Butyl *N*-[5-[[2-[5-(4-fluorophenyl)-2-methyl-morpholin-4-yl]-2-oxo-acetyl]amino]-3-methyl-2-pyridyl]carbamate (0.153 g, 323.80 μmol) was dissolved in dioxane (2 mL) and H_2O (2 mL) and stirred overnight at 100 °C. The next day it was evaporated under vacuum and purified by HPLC to obtain *N*-(6-amino-5-methyl-3-pyridyl)-2-[5-(4-fluorophenyl)-2-methyl-morpholin-4-yl]-2-oxo-acetamide (0.0495 g, 132.92 μmol, 41% yield). LCMS (ESI): [M]+ m/z: calcd 372.2; found 373.2; Rt = 0.969 min.

Chiral separation: (Column: Chiralcel OJ-H (250 x 20 mm, 5 μm); Mobile phase Hexane-IPA-MeOH 50-25-25 Flow Rate: 12 mL / min) to obtain *N*-(6-amino-5-methylpyridin-3-yl)-2-((2*R*,5*S*)-5-(4-fluorophenyl)-2-methylmorpholino)-2-oxoacetamide, **Compound 22** (16.40 mg, 44.04 μmol, 36% yield) and *N*-(6-amino-5-methylpyridin-3-yl)-2-((2*S*,5*R*)-5-(4-fluorophenyl)-2-methylmorpholino)-2-oxoacetamide (0.01643 g, 44.12 μmol, 36% yield) .

Rel Time for *N*-(6-amino-5-methylpyridin-3-yl)-2-((2*R*,5*S*)-5-(4-fluorophenyl)-2-methylmorpholino)-2-oxoacetamide, **Compound 22** in analytical conditions (column: OJ-3, Hexane-IPA-MeOH, 50-25-25, 0.15 mL / min as mobile phase) 10.35 min and for *N*-(6-amino-5-methylpyridin-3-yl)-2-((2*S*,5*R*)-5-(4-fluorophenyl)-2-methylmorpholino)-2-oxoacetamide 13.16 min.

N-(6-amino-5-methylpyridin-3-yl)-2-((2*R*,5*S*)-5-(4-fluorophenyl)-2-methylmorpholino)-2-oxoacetamide, **Compound 22**: Retention time: 10.35 min. ^{1}H NMR (600 MHz, DMSO-d_6) δ 1.22 (d, 3H), 2.00 (s, 3H), 3.90 (m, 5H), 5.45 (m, 3H), 7.19 (m, 2H), 7.46 (m, 3H), 7.95 (m, 1H), 10.47 (m, 1H). LCMS (ESI): [M]+ m/z: calcd 372.2; found 373.2; Rt = 1.166 min. *N*-(6-amino-5-methylpyridin-3-yl)-2-((2*S*,5*R*)-5-(4-fluorophenyl)-2-methylmorpholino)-2-oxoacetamide: Retention time: 13.16 min. ^{1}H NMR (600 MHz, DMSO-d_6) δ 1.22 (d, 3H), 2.00 (s, 3H), 3.85 (m, 5H), 5.45 (m, 3H), 7.19 (m, 2H), 7.42 (m, 3H), 7.95 (m, 1H), 10.47 (d, 1H). LCMS (ESI): [M]+ m/z: calcd 372.2; found 373.2; Rt = 1.136 min.

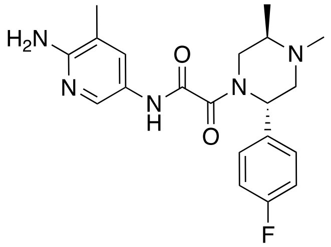

N-(6-amino-5-methylpyridin-3-yl)-2-((2*S*,5*R*)-2-(4-fluorophenyl)-4,5-dimethylpiperazin-1-yl)-2-oxoacetamide, **Compound 24**.

Amine: **Intermediate 24a**. Acid: **Intermediate 23acid**. Yield: 23 %. HPLC conditions: Column SunFire 19 x 100mm; 0 - 75 % 2 - 8 min; water / MeOH + NH_3; 30 mL / min; loading pump 4 mL / min MeOH. ^{1}H NMR (600 MHz, DMSO-d_6) δ 0.94 (m, 3H), 2.01 (m, 3H), 2.23 (s, 3H), 2.92 (m, 4H), 3.72 (m, 1H), 5.27 (m, 1H), 5.62 (m, 2H), 7.17 (m, 2H), 7.50 (m, 3H), 8.00 (s, 1H), 10.50 (d, 1H). LCMS (ESI): [M]+ m/z: calcd 385.2; found 386.2; Rt = 1.500 min.

Chiral separation: 94.5 mg (245.18 μmol) separated via (Column: Chiralpak IA (250 x 20 mm x 5 μm); Mobile phase : Hexane-IPA-MeOH, 50-25-25 Flow Rate: 12 mL/min; Column Temperature: 24 °C; Wavelength: 205 nm, 215nm, 254nm) to obtain *N*-(6-amino-5-methylpyridin-3-yl)-2-((2*S*,5*R*)-2-(4-fluorophenyl)-4,5-dimethylpiperazin-1-yl)-2-oxoacetamide, **Compound 24** (41.83 mg, 108.53 μmol, 44% yield) (Rt = 32.47 min) and *N*-(6-amino-5-methylpyridin-3-yl)-2-((2*R*,5*S*)-2-(4-fluorophenyl)-4,5-dimethylpiperazin-1-yl)-2-oxoacetamide (46.42 mg, 120.44 μmol, 49% yield) (Rt = 25.26 min).

Rel Time for **Compound 24** in analytical conditions (column: IA, IPA-MeOH, 50-50, 0.5 mL/min as mobile phase) 18.95 min and for *R*, *S* enantiomer = 24.84 min.
^{1}H NMR (600 MHz, DMSO-d_6) δ 0.94 (d, 3H), 2.00 – 2.03 (m, 3H), 2.23 (s, 3H), 2.76 – 2.87 (m, 2H), 2.87 – 3.08 (m, 2H), 3.44 – 4.00 (m, 1H), 5.04 – 5.49 (m, 1H), 5.63 (s, 2H), 7.10 – 7.20 (m, 2H), 7.45 – 7.56 (m, 3H), 8.00 (d, 1H), 10.48 – 10.56 (m, 1H). LCMS (ESI): [M]+ m/z: calcd 385.2; found 386.2; Rt = 1.601 min.

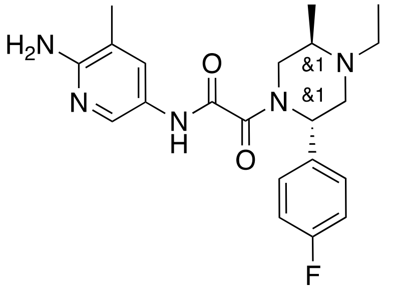

Rac-N-(6-amino-5-methylpyridin-3-yl)-2-(*trans*-4-ethyl-2-(4-fluorophenyl)-5-methylpiperazin-1-yl)-2-oxoacetamide, **Compound 25**.
Amine: **Intermediate 25a**. Acid: **Intermediate 23acid**. Yield: 15 %. HPLC conditions: Column SunFire 19 x 100 mm; 40 - 70 %, 2 - 10 min; water / MeOH + NH_3; 30 mL / min; loading pump 4 mL / min MeOH. ^{1}H NMR (600 MHz, DMSO-d_6) δ 0.93 (d, 3H), 1.01 (t, 3H), 1.98 – 2.04 (m, 3H), 2.38 – 2.42 (m, 2H), 2.71 – 2.97 (m, 2H), 2.96 – 3.08 (m, 1H), 3.14 – 3.23 (m, 1H), 3.47 – 4.00 (m, 1H), 5.06 – 5.52 (m, 1H), 5.62 (s, 2H), 7.14 – 7.22 (m, 2H), 7.44 – 7.50 (m, 1H), 7.50 – 7.58 (m, 2H), 8.00 (s, 1H), 10.49 (br s, 1H). LCMS (ESI): [M]+ m/z: calcd 399.2; found 400.2; Rt = 0.485 min.

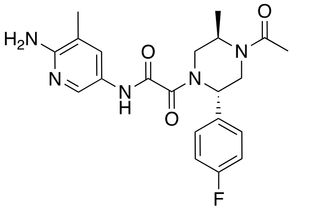

2-((2*S*,5*R*)-4-acetyl-2-(4-fluorophenyl)-5-methylpiperazin-1-yl)-*N*-(6-amino-5-methylpyridin-3-yl)-2-oxoacetamide, **Compound 26**.
Amine: **Intermediate 26b**. Acid: **Intermediate 23acid**. Yield: 36 %. LCMS (ESI): [M]+ m/z: calcd 413.2; found 414.2; Rt = 0.952 min.
Chiral separation: 148 mg (357.97 µmol) separated via (Chiralcel OJ-H, 250 x 20 mm, 5-I Hexane-IPA-MeOH, 50-25-25, 13 mL / min, 35 mg / inj., 1 hr / inj., 4 injections) to obtain 2-((2*R*,5*S*)-4-acetyl-2-(4-fluorophenyl)-5-methylpiperazin-1-yl)-*N*-(6-amino-5-methylpyridin-3-yl)-2-oxoacetamide (62.47 mg, 151.10 µmol, 42% yield) with Rt = 28.54 min and 2-((2*S*,5*R*)-4-acetyl-2-(4-fluorophenyl)-5-methylpiperazin-1-yl)-*N*-(6-amino-5-methylpyridin-3-yl)-2-oxoacetamide, **Compound 26** (102.36 mg, 247.58 µmol, 69% yield) with Rt = 16.52 min.
Rel Time for 2-[(2*R*,5*S*)-4-acetyl-2-(4-fluorophenyl)-5-methyl-piperazin-1-yl]-N-(6-amino-5-methyl-3-pyridyl)-2-oxo-acetamide in analytical conditions (column: OJ-H, Hexane-IPA-MeOH, 50-25-25, 0.6 mL / min as mobile phase) 16.67 min and for **Compound 26** 31.00 min.
2-[(2*R*,5*S*)-4-acetyl-2-(4-fluorophenyl)-5-methyl-piperazin-1-yl]-N-(6-amino-5-methyl-3-pyridyl)-2-oxo-acetamide:
Retention time: 16.67 min
^{1}H NMR (600 MHz, DMSO-d_6) δ 1.18 (m, 3H), 1.96 (m, 6H), 3.02 (m, 2H), 3.73 (m, 2H), 4.74 (m, 1H), 5.56 (m, 3H), 7.27 (m, 4H), 7.50 (m, 1H), 7.94 (m, 1H), 10.53 (m, 1H). LCMS (ESI): [M]+ m/z: calcd 413.2; found 414.2; Rt = 1.793 min.
Compound 26:
Retention time: 31.00 min
^{1}H NMR (600 MHz, DMSO-d_6) δ 1.20 (m, 3H), 2.00 (m, 6H), 3.13 (m, 2H), 3.64 (m, 1H), 4.52 (m, 2H), 5.56 (m, 3H), 7.19 (m, 2H), 7.37 (m, 2H), 7.50 (m, 1H), 7.94 (m, 1H), 10.53 (m, 1H). LCMS (ESI): [M]+ m/z: calcd 413.2; found 414.2; Rt = 1.818 min.

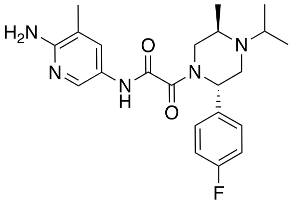

N-(6-amino-5-methylpyridin-3-yl)-2-((2*S*,5*R*)-2-(4-fluorophenyl)-4-isopropyl-5-methylpiperazin-1-yl)-2-oxoacetamide, **Compound 27**.
Amine: **Intermediate 27a**. Acid: **Intermediate 23acid**. Yield: 17 %. HPLC conditions: Column SunFire 19 x 100 mm; 0 - 75%, 2-8 min; water / MeOH + NH_3; 30 mL / min; loading pump 4 mL / min MeOH. ^{1}H NMR (600 MHz, DMSO-d_6) δ 0.99 (m, 9H), 2.01 (m, 3H), 2.77 (m, 3H), 3.15 (m, 2H), 3.77 (m, 1H), 5.53 (m, 3H), 7.16 (m, 2H), 7.48 (m, 3H), 7.98 (m, 1H), 10.45 (m, 1H). LCMS (ESI): [M]+ m/z: calcd 413.2; found 414.2; Rt = 1.615 min.
Chiral separation: (37.2 mg, 89.97 µmol) was chirally separated (Column: Chiralpak IC (250 x 20mm x 5 µm); Mobile phase: Hexane-IPA-MeOH, 80-10-10, Flow Rate: 12 mL / min; Column Temperature: 24 °C; Wavelength: 205 nm, 270 nm, 308 nm) to obtain *N*-(6-amino-5-methylpyridin-3-yl)-2-((2*S*,5*R*)-2-(4-fluorophenyl)-4-isopropyl-5-methylpiperazin-1-yl)-2-oxoacetamide, **Compound 27** (13.12 mg, 31.73 µmol, 35% yield) (Rt = 34.69 min) and *N*-(6-amino-5-methylpyridin-3-yl)-2-((2*R*,5*S*)-2-(4-fluorophenyl)-4-isopropyl-5-methylpiperazin-1-yl)-2-oxoacetamide (13.68 mg, 33.08 µmol, 37% yield) (Rt = 45.09 min).
Rel Time for **Compound 27** in analytical conditions (column: IC, Hexane-IPA-MeOH, 80-10-10, 0.6 mL / min as mobile phase) 45.17 min and for *N*-(6-amino-5-methylpyridin-3-yl)-2-((2*R*,5*S*)-2-(4-fluorophenyl)-4-isopropyl-5-methylpiperazin-1-yl)-2-oxoacetamide 35.11 min.
Compound 27: Retention time: 45.17 min

^{1}H NMR (600 MHz, DMSO-d_6) δ 0.97 (d, 6H), 1.05 (d, 3H), 1.97 – 2.05 (m, 3H), 2.61 – 2.67 (m, 1H), 2.78 – 2.92 (m, 1H), 3.08 – 3.25 (m, 2H), 3.25 – 3.41 (m, 1H), 3.51 – 4.01 (m, 1H), 5.06 – 5.41 (m, 1H), 5.58 – 5.68 (m, 2H), 7.10 – 7.21 (m, 2H), 7.41 – 7.56 (m, 3H), 7.92 – 8.04 (m, 1H), 10.41 – 10.54 (m, 1H). LCMS (ESI): [M]+ m/z: calcd 413.2; found 414.2; Rt = 1.498 min.
N-(6-amino-5-methylpyridin-3-yl)-2-((2*R*,5*S*)-2-(4-fluorophenyl)-4-isopropyl-5-methylpiperazin-1-yl)-2-oxoacetamide:
Retention time: 35.11 min
^{1}H NMR (600 MHz, DMSO-d_6) δ 0.97 (d, 6H), 1.05 (d, 3H), 1.98 – 2.04 (m, 3H), 2.61 – 2.66 (m, 1H), 2.78 – 2.91 (m, 1H), 3.08 – 3.23 (m, 2H), 3.23 – 3.42 (m, 1H), 3.51 – 4.00 (m, 1H), 5.06 – 5.41 (m, 1H), 5.58 – 5.66 (m, 2H), 7.10 – 7.20 (m, 2H), 7.41 – 7.54 (m, 3H), 7.93 – 8.02 (m, 1H), 10.42 – 10.50 (m, 1H). LCMS (ESI): [M]+ m/z: calcd 413.2; found 414.2; Rt = 1.503 min.

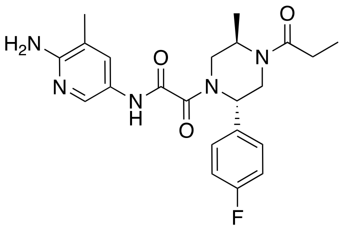

N-(6-amino-5-methylpyridin-3-yl)-2-((2*S*,5*R*)-2-(4-fluorophenyl)-5-methyl-4-propionylpiperazin-1-yl)-2-oxoacetamide, **Compound 28**.
Amine: **Intermediate 28c**. Acid: **Intermediate 23acid**. Yield: 25 %. HPLC conditions: Column SunFire 19 x 100 mm, 5 μm; 15 - 25%, 2 - 10 min; MeCN 30 mL / min; loading pump 4 mL / min MeCN. ^{1}H NMR (600 MHz, DMSO-d_6) δ 0.85 – 0.88 (m, 1H), 0.95 (td, 2H), 1.10 (d, 1H), 1.22 (dd, 2H), 2.00 – 2.04 (m, 3H), 2.16 – 2.28 (m, 1H), 2.37 – 2.45 (m, 1H), 2.68 – 3.25 (m, 2H), 3.51 – 3.61 (m, 0,5H), 3.63 – 3.78 (m, 0,5H), 3.99 – 4.53 (m, 1,5H), 4.93 (dd, 0,5H), 5.27 – 5.67 (m, 3H), 7.15 – 7.23 (m, 2H), 7.27 – 7.32 (m, 1H), 7.36 – 7.52 (m, 2H), 7.84 – 8.03 (m, 1H), 10.49 – 10.65 (m, 1H). LCMS (ESI): [M]+ m/z: calcd 427.2; found 428.2; Rt = 1.831 min.
Chiral separation: (Chiralcel OJ-H-I (250 x 20mm, 5 μm), Hexane-IPA-MeOH, 50-25-25, 12 ml/min) to obtain *N*-(6-amino-5-methylpyridin-3-yl)-2-((2*S*,5*R*)-2-(4-fluorophenyl)-5-methyl-4-propionylpiperazin-1-yl)-2-oxoacetamide, **Compound 28** (35.73 mg, 83.58 μmol, 40% yield) (Rt = 15.56 min) and *N*-(6-amino-5-methylpyridin-3-yl)-2-((2*R*,5*S*)-2-(4-fluorophenyl)-5-methyl-4-propionylpiperazin-1-yl)-2-oxoacetamide (37.99 mg, 88.87 μmol, 43% yield) (Rt = 27.36 min).
Rel Time for *N*-(6-amino-5-methylpyridin-3-yl)-2-((2*R*,5*S*)-2-(4-fluorophenyl)-5-methyl-4-propionylpiperazin-1-yl)-2-oxoacetamide in analytical conditions (column: OJ-H, Hexane-IPA-MeOH, 50-25-25, 0.6 mL / min as mobile phase) 15.50 min and for **Compound 28** 29.71 min.
N-(6-amino-5-methylpyridin-3-yl)-2-((2*R*,5*S*)-2-(4-fluorophenyl)-5-methyl-4-propionylpiperazin-1-yl)-2-oxoacetamide:
Retention time: 15.50 min
^{1}H NMR (600 MHz, DMSO-d_6) δ 0.87 (td, 1H), 0.95 (td, 2H), 1.10 (d, 1H), 1.22 (dd, 2H), 1.95 – 2.06 (m, 3,5H), 2.16 – 2.29 (m, 1H), 2.38 – 2.45 (m, 0,5H), 2.69 – 3.26 (m, 1,5H), 3.50 – 3.60 (m, 0,5H), 3.65 – 3.74 (m, 0,5H), 3.95 – 4.03 (m, 0,5H), 4.07 – 4.18 (m, 1H), 4.36 – 4.59 (m, 0,5H), 4.89 – 4.96 (m, 0,5H), 5.27 – 5.67 (m, 3H), 7.13 – 7.25 (m, 2H), 7.28 – 7.42 (m, 2H), 7.48 – 7.52 (m, 1H), 7.83 – 8.04 (m, 1H), 10.35 – 10.65 (m, 1H). LCMS (ESI): [M]+ m/z: calcd 427.2; found 428.2; Rt = 2.251 min.
Compound 28:
Retention time: 29.71 min
^{1}H NMR (600 MHz, DMSO-d_6) δ 0.87 (td, 1H), 0.95 (td, 2H), 1.10 (d, 1H), 1.22 (dd, 2H), 1.96 – 2.07 (m, 3,5H), 2.16 – 2.30 (m, 1H), 2.37 – 2.43 (m, 0,5H), 2.68 – 3.22 (m, 1,5H), 3.49 – 3.60 (m, 0,5H), 3.65 – 3.74 (m, 0,5H), 3.96 – 4.04 (m, 0,5H), 4.05 – 4.17 (m, 1H), 4.36 – 4.59 (m, 0,5H), 4.93 (dd, 0,5H), 5.26 – 5.69 (m, 3H), 7.15 – 7.24 (m, 2H), 7.28 – 7.42 (m, 2H), 7.48 – 7.53 (m, 1H), 7.84 – 8.05 (m, 1H), 10.34 – 10.62 (m, 1H). LCMS (ESI): [M]+ m/z: calcd 427.2; found 428.2; Rt = 2.240 min.

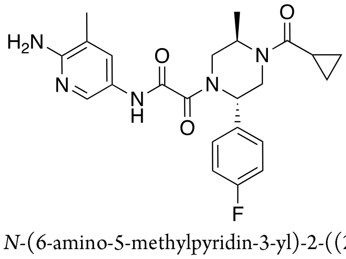

N-(6-amino-5-methylpyridin-3-yl)-2-((2*S*,5*R*)-4-(cyclopropanecarbonyl)-2-(4-fluorophenyl)-5-methylpiperazin-1-yl)-2-oxoacetamide, **Compound 29**.
Amine: **Intermediate 29b**. Acid: **Intermediate 23acid**. Yield: 14 %. HPLC conditions: Column SunFire 19 x 100 mm, 5 μm; 30 - 55%, 2 - 10 min; MeOH + NH_3 30 mL / min; loading pump 4 mL / min MeOH. ^{1}H NMR (600 MHz, DMSO-d_6) δ 0.41 – 0.70 (m, 2H), 0.70 – 0.79 (m, 2H), 1.10 – 1.28 (m, 3H), 1.86 – 1.96 (m, 1H), 1.97 – 2.07 (m, 3H), 2.80 – 3.28 (m, 2H), 3.61 – 4.10 (m, 1H), 4.43 – 4.58 (m, 1H), 4.59 – 4.92 (m, 1H), 5.29 – 5.61 (m, 1H), 5.61 – 5.69 (m, 2H), 7.14 – 7.24 (m, 2H), 7.25 – 7.37 (m, 1H), 7.37 – 7.41 (m, 1H), 7.42 – 7.52 (m, 1H), 7.86 – 8.08 (m, 1H), 10.41 – 10.63 (m, 1H). LCMS (ESI): [M]+ m/z: calcd 439.2; found 440.2; Rt = 2.379 min.
Chiral separation: 47.25 mg (107.51 μmol) separated via (Column: Chiralcel OD-H (250 x 20 mm, 5 μm), Hexane-IPA-MeOH, 70-15-15, 12 mL / min) to obtain *N*-(6-amino-5-methylpyridin-3-yl)-2-((2*S*,5*R*)-4-(cyclopropanecarbonyl)-2-(4-fluorophenyl)-5-methylpiperazin-1-yl)-2-oxoacetamide, **Compound 29** (20.08 mg) (Rt = 21.28 min) and *N*-(6-amino-5-methylpyridin-3-yl)-2-((2*R*,5*S*)-4-(cyclopropanecarbonyl)-2-(4-fluorophenyl)-5-methylpiperazin-1-yl)-2-oxoacetamide (19.37 mg) (Rt = 25.04 min).
Retention time for *N*-(6-amino-5-methylpyridin-3-yl)-2-((2*R*,5*S*)-4-(cyclopropanecarbonyl)-2-(4-fluorophenyl)-5-methylpiperazin-1-yl)-2-oxoacetamide in analytical conditions (column: OD-H, Hexane-IPA-MeOH, 50-25-25, 0.6 mL / min as mobile phase) = 11.22 min and for **Compound 29** = 10.17 min.
N-(6-amino-5-methylpyridin-3-yl)-2-((2*R*,5*S*)-4-(cyclopropanecarbonyl)-2-(4-fluorophenyl)-5-methylpiperazin-1-yl)-2-oxoacetamide: ^{1}H NMR (600 MHz, DMSO-d_6) δ 0.41 – 0.74 (m, 4H), 1.04 – 1.28 (m, 3H), 1.89 – 2.06 (m, 4H), 2.60 – 3.20 (m, 1H), 3.44 – 4.08 (m, 2H), 4.47 – 4.85 (m, 2H), 5.32 – 5.63 (m, 2H), 6.04 (m, 1H), 7.16 – 7.58 (m, 5H), 7.96 – 8.08 (m, 1H), 10.56 – 10.71 (m, 1H). LCMS (ESI): [M]+ m/z: calcd 439.2; found 440.2; Rt = 2.111 min.

Compound 29: ^{1}H NMR (600 MHz, DMSO-d_6) δ 0.41 – 0.74 (m, 4H), 1.04 – 1.28 (m, 3H), 1.89 – 2.06 (m, 4H), 2.60 – 3.20 (m, 1H), 3.44 – 4.08 (m, 2H), 4.47 – 4.85 (m, 2H), 5.32 – 5.63 (m, 2H), 6.04 (m, 1H), 7.16 – 7.58 (m, 5H), 7.96 – 8.08 (m, 1H), 10.56 – 10.71 (m, 1H).
LCMS (ESI): [M]+ m/z: calcd 439.2; found 440.2; Rt = 2.106 min.

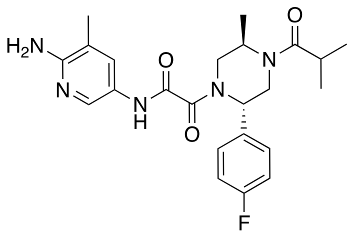

N-(6-amino-5-methylpyridin-3-yl)-2-((2*S*,5*R*)-2-(4-fluorophenyl)-4-isobutyryl-5-methylpiperazin-1-yl)-2-oxoacetamide, **Compound 30**.
Amine: **Intermediate 30c**. Acid: **Intermediate 23acid**. Yield: 16 %. HPLC conditions: Column SunFire 19 x 100 mm, 5 μm; 40 - 55%, 2 - 10 min; MeOH + NH_3 30 mL / min; loading pump 4 mL / min MeOH. LCMS (ESI): [M]+ m/z: calcd 441.2; found 442.2; Rt = 0.992 min.
Chiral separation: 95 mg (215.18 μmol) separated via (Column: Chiralpak AD-H III (250 x 20 mm, 5 μm); Mobile phase : IPA-MeOH 50-50 Flow Rate: 12 mL / min; m = 0.09 g, 3 injections , 30 mg / inj., 4.2 h) to obtain crude product which was re-purified (Column: Chiralcel OD-H (250 x 20 mm , 5 μm); Mobile phase: Hexane-IPA-MeOH 80-10-10 Flow Rate: 12 mL / min; m = 0.045 g, 4 injections, 11 mg / inj., 2.5 h) to obtain *N*-(6-amino-5-methylpyridin-3-yl)-2-((2*S*,5*R*)-2-(4-fluorophenyl)-4-isobutyryl-5-methylpiperazin-1-yl)-2-oxoacetamide, **Compound 30** (27.63 mg, 62.58 μmol, 29% yield) with Rt = 16.487 min and *N*-(6-amino-5-methylpyridin-3-yl)-2-((2*R*,5*S*)-2-(4-fluorophenyl)-4-isobutyryl-5-methylpiperazin-1-yl)-2-oxoacetamide (25.06 mg, 56.76 μmol, 26% yield) with Rt = 36.316 min.
Rel Time for *N*-(6-amino-5-methylpyridin-3-yl)-2-((2*R*,5*S*)-2-(4-fluorophenyl)-4-isobutyryl-5-methylpiperazin-1-yl)-2-oxoacetamide in analytical conditions (column: OD-H, Hexane-IPA-MeOH, 80-10-10, 0.6 mL / min as mobile phase) 29.85 min and for **Compound 30** 25.27 min.
N-(6-amino-5-methylpyridin-3-yl)-2-((2*R*,5*S*)-2-(4-fluorophenyl)-4-isobutyryl-5-methylpiperazin-1-yl)-2-oxoacetamide: Retention time: 29.85 min. LCMS (ESI): [M]+ m/z: calcd 441.2; found 442.2; Rt = 2.362 min. ^{1}H NMR (600 MHz, DMSO-d_6) δ 0.97 (m, 9H), 2.00 (m, 3H), 2.73 (m, 1H), 3.13 (m, 1H), 3.67 (m, 1H), 4.16 (m, 2H), 5.01 (m, 1H), 5.63 (m, 2H), 7.18 (m, 2H), 7.33 (m, 2H), 7.50 (m, 1H), 8.02 (m, 1H), 10.58 (m, 1H).
Compound 30: Retention time: 25.27 min. LCMS (ESI): [M]+ m/z: calcd 441.2; found 442.2; Rt = 2.357 min. ^{1}H NMR (600 MHz, DMSO-d_6) δ 1.00 (m, 9H), 2.02 (m, 3H), 2.85 (m, 2H), 3.61 (m, 1H), 3.95 (m, 2H), 4.83 (m, 1H), 5.63 (m, 3H), 7.24 (m, 4H), 7.50 (m, 1H), 8.02 (m, 1H), 10.58 (m, 1H).

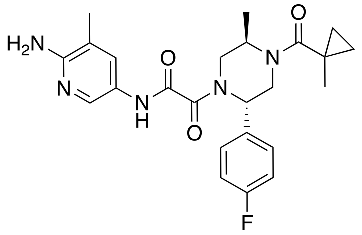

N-(6-amino-5-methylpyridin-3-yl)-2-((2*S*,5*R*)-2-(4-fluorophenyl)-5-methyl-4-(1-methylcyclopropane-1-carbonyl)piperazin-1-yl)-2-oxoacetamide, **Compound 31**.
Amine: **Intermediate 31b**. Acid: **Intermediate 23acid**. Yield: 42 %. HPLC conditions: Column YMC Triart C18, 20 x 100 mm, 5 μm; 20 – 70 %, 0 - 5 min; MeOH / water + 0.1% NH_4OH 40 mL / min; loading pump 4 mL / min MeOH. ^{1}H NMR (600 MHz, DMSO-d_6) δ 0.46 (m, 2H), 0.71 (m, 2H), 1.05 (m, 3H), 1.26 (m, 3H), 2.02 (m, 4H), 2.99 (m, 1H), 3.66 (m, 1H), 4.27 (m, 1H), 4.70 (m, 1H), 5.64 (m, 3H), 7.19 (m, 2H), 7.29 (m, 1H), 7.37 (m, 1H), 7.48 (m, 1H), 8.02 (m, 1H), 10.62 (m, 1H). LCMS (ESI): [M]+ m/z: calcd 453.2; found 454.2; Rt = 2.546 min.

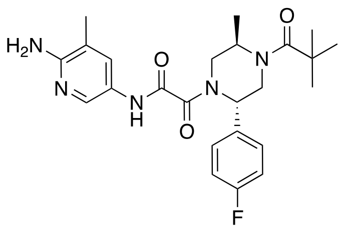

N-(6-amino-5-methylpyridin-3-yl)-2-((2*S*,5*R*)-2-(4-fluorophenyl)-5-methyl-4-pivaloylpiperazin-1-yl)-2-oxoacetamide, **Compound 32**.
Amine: **Intermediate 32b**. Acid: **Intermediate 23acid**. Yield: 48 %. HPLC conditions: Column YMC Triart C18, 20 x 100 mm, 5 μm; 50 – 80 %, 0 - 5 min; MeOH / water + 0.1% NH_4OH 40 mL / min; loading pump 4 mL / min MeOH. ^{1}H NMR (600 MHz, DMSO-d_6) δ 1.08 (m, 9H), 1.23 (m, 3H), 2.02 (m, 3H), 3.20 (m, 2H), 3.82 (dd, 1H), 4.45 (m, 1H), 4.89 (m, 1H), 5.64 (m, 3H), 7.17 (m, 2H), 7.28 (m, 1H), 7.37 (m, 1H), 7.48 (m, 1H), 8.02 (m, 1H), 10.58 (m, 1H). LCMS (ESI): [M]+ m/z: calcd 455.2; found 456.2; Rt = 2.828 min.

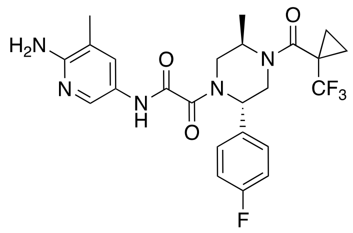

N-(6-amino-5-methylpyridin-3-yl)-2-((2*S*,5*R*)-2-(4-fluorophenyl)-5-methyl-4-(1-(trifluoromethyl)cyclopropane-1-carbonyl)piperazin-1-yl)-2-oxoacetamide, **Compound 33**.

Amine: **Intermediate 33b**. Acid: **Intermediate 23acid**. Yield: 27 %. HPLC conditions: Column YMC Triart C18, 20 x 100 mm, 5 μm; 40 – 55 %, 0 - 5 min; MeOH / water + 0.1% NH_4OH 40 mL / min; loading pump 4 mL / min MeOH. ^1H NMR (600 MHz, DMSO-d_6) δ 0.94 – 1.02 (m, 1H), 1.13 – 1.25 (m, 3H), 1.26 – 1.36 (m, 3H), 1.97 – 2.04 (m, 3H), 2.80 – 3.23 (m, 1H), 3.31 – 3.50 (m, 1H), 3.56 – 4.05 (m, 1H), 4.06 – 4.44 (m, 1H), 4.44 – 5.06 (m, 1H), 5.30 – 5.62 (m, 1H), 5.62 – 5.82 (m, 2H), 7.12 – 7.20 (m, 2H), 7.22 – 7.32 (m, 1H), 7.32 – 7.41 (m, 1H), 7.42 – 7.59 (m, 1H), 7.96 – 8.08 (m, 1H), 10.40 – 10.78 (m, 1H). LCMS (ESI): [M]+ m/z: calcd 507.2; found 508.2; Rt = 2.526 min.

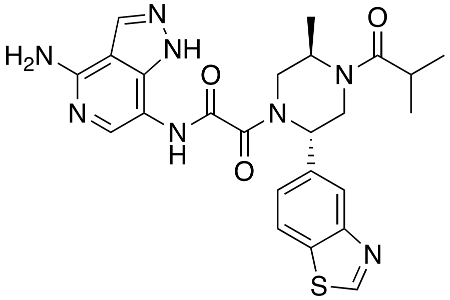

N-(4-amino-1*H*-indazol-7-yl)-2-((2*S*,5*R*)-2-(benzo[*d*]thiazol-5-yl)-4-isobutyryl-5-methylpiperazin-1-yl)-2-oxoacetamide, **Compound 39**.

Amine: **Intermediate 39b**. Acid: **Intermediate 39a**. Deprotection: bis-Boc protected material (100 mg, 0.126 mmol), DCM (4 mL) and TFA (0.2 mL, 2.60 mmol) were stirred at 20 °C for 2 h. The resulting solution was adjusted to pH = 8 with NH_3-H_2O then concentrated under reduced pressure to give a crude product, which was purified by preparative HPLC (Instrument: Gilson GX-281 Liquid Handler, Gilson 322 Pump, Gilson 156 UV Detector; Column: 2_Phenomenex Gemini C18, 75 x 40 mm x 3 μm; Mobile phase A: H_2O with 0.05 % NH_3-H_2O (v %); Mobile phase B: MeCN; Gradient: B from 12 % to 42 % in 9.5 min, hold 100 % B for 3 min; Flow Rate: 25 mL / min; Column Temperature: 30 °C; Wavelength: 220 nm, 254 nm) to afford 10 mg of a crude) white solid. LCMS (ESI) [M+H]+ m/z: calcd 507.2, found 507.3. The material was separated from residual isomers by SFC (Instrument: Berger, MULTIGR AM-II; Column: DAICEL CHIRALCEL OJ (250 mm x 30 mm x 10 μm); Mobile phase: supercritical Hexane-IPA (0.1 % NH_3, MeOH v %) = 70 / 30; Flow Rate:70 mL / min; Column Temperature: 38 °C; Nozzle Pressure: 100 bar; Nozzle Temperature: 60 °C; Evaporator Temperature: 20 °C; Trimmer Temperature: 25 °C; Wavelength: 220 nm) to afford *N*-(4-amino-1*H*-indazol-7-yl)-2-((2*S*,5*R*)-2-(benzo[*d*]thiazol-5-yl)-4-isobutyryl-5-methylpiperazin-1-yl)-2-oxoacetamide, **Compound 39** (4.9 mg, white solid). ^1H NMR (400 MHz, MeOD-d_4) δ 9.15 - 9.35 (m, 1H), 8.16 - 8.26 (m, 1H), 8.03 - 8.14 (m, 2H), 7.47 - 7.81 (m, 2H), 5.68 - 6.12 (m, 1H), 5.21 (br d, *J* = 14.5 Hz, 1H), 4.28 - 4.42 (m, 1H), 3.90 - 4.17 (m, 1H), 3.41 - 3.64 (m, 1H), 2.56 - 3.13 (m, 1H), 1.44 (dd, *J* = 10.5, 6.8 Hz, 2H), 1.27 - 1.35 (m, 2H), 1.13 (dd, *J* = 6.6, 1.5 Hz, 2H), 0.96 - 1.05 (m, 3H), 0.69 (dd, *J* = 8.9, 6.6 Hz, 1H). LCMS (ESI): [M+H]+ m/z: calcd 507.2, found 507.3; HPLC: 100% at 254 nm; SFC: 93.6 % ee.

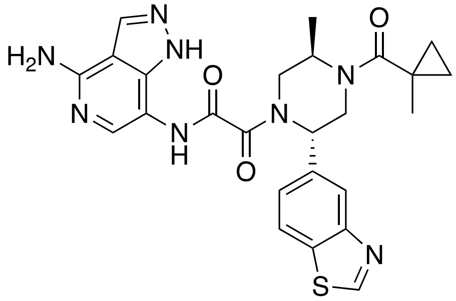

N-(4-amino-1*H*-indazol-7-yl)-2-((2*S*,5*R*)-2-(benzo[*d*]thiazol-5-yl)-5-methyl-4-(1-methylcyclopropane-1-carbonyl)piperazin-1-yl)-2-oxoacetamide, **Compound 40**.

Amine: **Intermediate 40b**. Acid: **Intermediate 39a**. Deprotection: bis-Boc protected material (40 mg, 49.8 μmol), DCM (4 mL) and TFA (2 mL, 25.9 mmol) were stirred at 20 °C for 4 h. The resulting solution was adjusted to pH = 8 with NH_3-H_2O then concentrated under reduced pressure to give a crude product, which was purified by flash chromatography (Biotage®, Column: SepaFlash® Sphercial C18, 40 g, 40-60 μm, 120 Å; MeCN / water (0.05 % NH_3-H_2O) with MeCN from 0 ~ 30 %, 30 mL / min, 254 nm) to afford *N*-(4-amino-1*H*-indazol-7-yl)-2-((2*S*,5*R*)-2-(benzo[*d*]thiazol-5-yl)-5-methyl-4-(1-methylcyclopropane-1-carbonyl)piperazin-1-yl)-2-oxoacetamide, **Compound 40** (7 mg, 27% yield) as white solid. ^1H NMR (400 MHz, MeOD-d_4) δ 9.10 -

9.35 (m, 1 H), 8.00 - 8.37 (m, 3 H), 7.42 - 7.88 (m, 2 H), 6.12 (br s, 1 H), 5.22 (br s, 1 H), 4.17 - 4.40 (m, 1 H), 3.40 - 3.68 (m, 1 H), 0.86 - 1.62 (m, 8 H), 0.30 - 0.82 (m, 4 H). LCMS (ESI): [M+H]+ m/z: calcd 519.2, found 519.3; HPLC: 100% at 254 nm; Chiral SFC： 81 % ee.

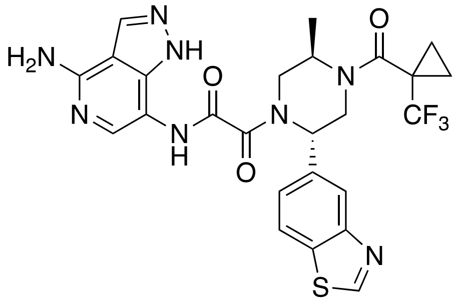

N-(4-amino-1*H*-indazol-7-yl)-2-((2*S*,5*R*)-2-(benzo[*d*]thiazol-5-yl)-5-methyl-4-(1-(trifluoromethyl)cyclopropane-1-carbonyl)piperazin-1-yl)-2-oxoacetamide, **Compound 41**.

Amine: **Intermediate 41b**. Acid: **Intermediate 39a**. Deprotection: bis-Boc protected material (70 mg, 81.7 μmol), DCM (1 mL) and TFA (0.4 mL, 5.20 mmol) was stirred at 20 °C for 14 h. Upon concentration, the residue was purified by preparative TLC (silica, DCM/MeOH = 1:1, 254 nm) to afford product (40 mg, 65% purity) as yellow solid which was purified by flash chromatography (Column: SepaFlash® Sphercial C18, 20 g, 40 - 60 μm, 120 Å; MeCN / water (0.05 v % NH_3-H_2O) with MeCN from 0 – 38 %, 30 mL / min, 254 nm) to give *N*-(4-amino-1*H*-indazol-7-yl)-2-((2*S*,5*R*)-2-(benzo[*d*]thiazol-5-yl)-5-methyl-4-(1-(trifluoromethyl)cyclopropane-1-carbonyl)piperazin-1-yl)-2-oxoacetamide, **Compound 41** (10.7 mg, 23 % yield) as white solid. ^{1}H NMR (400 MHz, MeOD-*d*$_4$) δ 9.19 - 9.30 (m, 1H), 8.26 (br s, 1H), 8.00 - 8.18 (m, 2H), 7.75 (br s, 1H), 7.46 - 7.64 (m, 1H), 6.17 (br s, 1H), 5.26 (br d, *J* = 14.3 Hz, 1H), 4.16 - 4.39 (m, 2H), 3.50 - 3.70 (m, 1H), 1.19 - 1.55 (m, 7H), 1.05 (br s, 1H). ^{19}F NMR (376 MHz, MeOD-*d*$_4$) δ -68.81; LCMS (ESI): [M+H]+ m/z: calcd 573.2, found 573.3; HPLC: 99.16 % at 254 nm; SFC: 90.6 % ee.

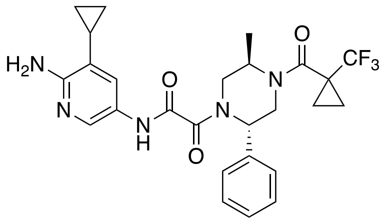

N-(6-amino-5-cyclopropylpyridin-3-yl)-2-((2*S*,5*R*)-5-methyl-2-phenyl-4-(1-(trifluoromethyl)cyclopropane-1-carbonyl)piperazin-1-yl)-2-oxoacetamide, **Compound 46**.

Amine: **Intermediate 46a**. Acid: **Intermediate 3a**. LCMS (ESI): [M]+ m/z: calcd 615.2; found 616.2; Rt = 1.342 min. Coupled material was stirred in dioxane and water at 90 °C overnight. The next day it was evaporated under vacuum and purified by HPLC (HPLC conditions: Column YMC Triart C18. 20 x 100 mm, 5 μm; 40 – 90 %, 0 - 5 min; MeOH / water + 0.1% NH_4OH 30 mL / min; loading pump 4 mL / min MeOH to obtain *N*-(6-amino-5-cyclopropylpyridin-3-yl)-2-((2*S*,5*R*)-5-methyl-2-phenyl-4-(1-(trifluoromethyl)cyclopropane-1-carbonyl)piperazin-1-yl)-2-oxoacetamide, **Compound 46.**
^{1}H NMR (600 MHz, DMSO-*d*$_6$) δ 0.40 – 0.52 (m, 2H), 0.85 – 0.91 (m, 2H), 0.93 – 1.01 (m, 1H), 1.11 – 1.23 (m, 3H), 1.25 – 1.35 (m, 3H), 1.58 – 1.69 (m, 1H), 2.57 – 3.12 (m, 1H), 3.19 – 3.29 (m, 0.7H), 3.63 – 3.73 (m, 0.3H), 3.83 – 4.57 (m, 2H), 4.88 – 5.29 (m, 1H), 5.38 – 5.76 (m, 1H), 5.76 – 5.87 (m, 2H), 7.21 – 7.37 (m, 6H), 7.99 – 8.15 (m, 1H), 10.56 (br s, 1H). LCMS (ESI): [M]+ m/z: calcd 515.2; found 516.2; Rt = 2.870 min.

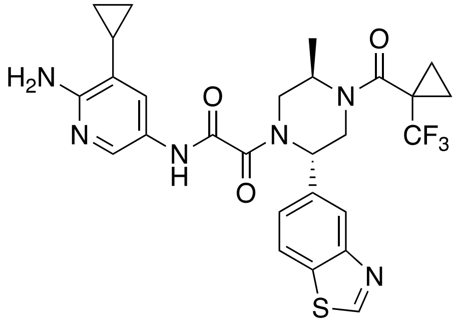

N-(4-amino-3-cyclopropylphenyl)-2-((2*S*,5*R*)-2-(benzo[*d*]thiazol-5-yl)-5-methyl-4-(1-(trifluoromethyl)cyclopropane-1-carbonyl)piperazin-1-yl)-2-oxoacetamide, **Compound 47**.

Amine: **Intermediate 41b**. Acid: **Intermediate 3a**. Purified by preparative HPLC (Instrument: Gilson GX-281 Liquid Handler, Gilson 322 Pump, Gilson 156 UV Detector; Column: Waters Xbridge 150 × 25 mm × 5 μm; Mobile phase A: H_2O with 0.05 % NH_3-H_2O (v %); Mobile phase B: MeCN; Gradient: B from 52% to 82% in 9.5 min, hold 100% B for 2.5 min; Flow Rate: 25 mL/min; Column Temperature: 30oC; Wavelength: 220 nm, 254 nm) to afford *N*-(4-amino-3-cyclopropylphenyl)-2-((2*S*,5*R*)-2-(benzo[*d*]thiazol-5-yl)-5-methyl-4-(1-(trifluoromethyl)cyclopropane-1-carbonyl)piperazin-1-yl)-2-oxoacetamide, **Compound 47** (8 mg, 36% yield) as a white solid. ^{1}H NMR (400 MHz, MeOD-*d*$_4$) δ 9.07 - 9.30 (m, 1 H), 7.81 - 8.11 (m, 3 H), 7.19 - 7.55 (m,

2 H), 5.07 - 6.01 (m, 1 H), 3.92 - 4.29 (m, 3 H), 3.36 - 3.57 (m, 1 H), 1.59 (br s, 1 H), 1.04 - 1.40 (m, 6 H), 0.66 - 0.99 (m, 4 H), 0.50 (br d, *J* = 4.5 Hz, 1 H), - 0.01 - 0.32 (m, 1 H). LCMS (ESI): [M+H]+ m/z: calcd 573.2, found 573.2; HPLC: 100 % at 220 nm, 100 % at 254 nm; 100 % ee.

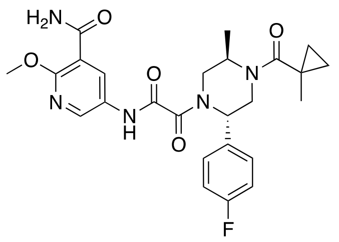

5-(2-((2*S*,5*R*)-2-(4-fluorophenyl)-5-methyl-4-(1-methylcyclopropane-1-carbonyl)piperazin-1-yl)-2-oxoacetamido)-2-methoxynicotinamide, **Compound 48**.

Amine: rac-**Intermediate 31b**. Acid: **Intermediate 48a**. Yield: 13 %. HPLC conditions: Column SunFire 19 x 100 mm, 5 μm; 30 - 80%, 2 - 10 min; MeOH + NH_3 30 mL / min; loading pump 4 mL / min MeOH. ^{1}H NMR (600 MHz, DMSO-d_6) δ 0.19 – 0.91 (m, 4H), 1.05 (s, 3H), 1.12 – 1.41 (m, 3H), 2.67 – 3.23 (m, 2H), 3.43 – 3.78 (m, 1H), 3.88 – 3.99 (m, 3H), 4.05 – 5.81 (m, 3H), 7.08 – 7.25 (m, 2H), 7.25 – 7.44 (m, 2H), 7.66 – 7.81 (m, 2H), 8.39 – 8.64 (m, 2H), 10.56 – 11.29 (m, 1H). LCMS (ESI): [M]+ m/z: calcd 497.2; found 498.2; Rt = 2.780 min.

Chiral separation: 23.8 mg (47.84 μmol) separated via (Column: Chiralcel OD-H (250 x 20 mm, 5 μm), Hexane-IPA-MeOH, 80-10-10, 12 mL / min) to obtain 5-(2-((2*S*,5*R*)-2-(4-fluorophenyl)-5-methyl-4-(1-methylcyclopropane-1-carbonyl)piperazin-1-yl)-2-oxoacetamido)-2-methoxynicotinamide, **Compound 48** (12.04 mg, 51% yield) (Rt = 31.79 min) and 5-(2-((2*R*,5*S*)-2-(4-fluorophenyl)-5-methyl-4-(1-methylcyclopropane-1-carbonyl)piperazin-1-yl)-2-oxoacetamido)-2-methoxynicotinamide (9.68 mg, 41% yield) (Rt = 39.11 min).

Rel Time for 5-(2-((2*R*,5*S*)-2-(4-fluorophenyl)-5-methyl-4-(1-methylcyclopropane-1-carbonyl)piperazin-1-yl)-2-oxoacetamido)-2-methoxynicotinamide in analytical conditions (column: OD-H, Hexane-IPA-MeOH, 60-20-20, 0.6 mL / min as mobile phase) 11.27 min and for **Compound 48** 13.02 min.

5-(2-((2*R*,5*S*)-2-(4-fluorophenyl)-5-methyl-4-(1-methylcyclopropane-1-carbonyl)piperazin-1-yl)-2-oxoacetamido)-2-methoxynicotinamide:

Retention time: 11.27 min. ^{1}H NMR (600 MHz, DMSO-d_6) δ 0.36 – 0.56 (m, 2H), 0.59 – 0.72 (m, 1H), 0.73 – 1.02 (m, 1H), 1.05 (s, 3H), 1.06 – 1.38 (m, 4H), 2.86 – 3.21 (m, 1H), 3.34 – 3.79 (m, 1H), 3.93 – 3.99 (m, 3H), 4.00 – 4.51 (m, 1H), 4.50 – 4.98 (m, 1H), 5.29 – 5.90 (m, 1H), 7.14 – 7.23 (m, 2H), 7.26 – 7.41 (m, 2H), 7.69 – 7.79 (m, 2H), 8.42 – 8.62 (m, 2H), 10.89 – 11.29 (m, 1H). LCMS (ESI): [M]+ m/z: calcd 497.2; found 498.2; Rt = 2.745 min.

Compound 48: Retention time: 13.02 min. ^{1}H NMR (600 MHz, DMSO-d_6) δ 0.36 – 0.56 (m, 2H), 0.59 – 0.72 (m, 1H), 0.73 – 1.02 (m, 1H), 1.05 (s, 3H), 1.06 – 1.38 (m, 4H), 2.86 – 3.21 (m, 1H), 3.34 – 3.79 (m, 1H), 3.93 – 3.99 (m, 3H), 4.00 – 4.51 (m, 1H), 4.50 – 4.98 (m, 1H), 5.29 – 5.90 (m, 1H), 7.14 – 7.23 (m, 2H), 7.26 – 7.41 (m, 2H), 7.69 – 7.79 (m, 2H), 8.42 – 8.62 (m, 2H), 10.89 – 11.29 (m, 1H).

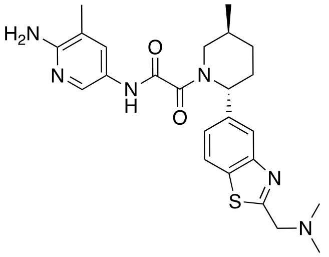

N-(6-amino-5-methylpyridin-3-yl)-2-((2*R*,5*S*)-2-(2-((dimethylamino)methyl)benzo[*d*]thiazol-5-yl)-5-methylpiperidin-1-yl)-2-oxoacetamide, **Compound 49**.

Amine: **Intermediate 49a**. Acid: **Intermediate 23acid**. Yield: 10 %. HPLC conditions: Column SunFire 19 x 100 mm, 5 μm; 50 – 75 %, 2 - 8 min; water-MeCN + NH_3 30 mL / min; loading pump 4 mL / min MeCN. ^{1}H NMR (600 MHz, DMSO-d_6) δ 0.71 – 0.78 (m, 2H), 1.03 (m, 3H), 1.32 – 1.39 (m, 1H), 1.67 – 1.73 (m, 2H), 1.85 (m, 1H), 1.97 (m, 1H), 2.08 (m, 2H), 2.30 (m, 6H), 2.78 (m, 1H), 3.86 (m, 3H), 5.62 (m, 2H), 7.34 – 7.54 (m, 2H), 7.85 (s, 1H), 7.88 – 8.06 (m, 2H), 10.51 (m, 1H). LCMS (ESI): [M]+ m/z: calcd 466.2; found 467.2; Rt = 2.007 min.

Chiral separation: Column: Chiralpak AS-H (250 x 20 mm, 5 μm); Mobile phase: Hexane-MeOH-IPA, 60-20-20; Flow Rate: 12 mL / min) to obtain *N*-(6-amino-5-methylpyridin-3-yl)-2-((2*R*,5*S*)-2-(2-((dimethylamino)methyl)benzo[*d*]thiazol-5-yl)-5-methylpiperidin-1-yl)-2-oxoacetamide, **Compound 49** (18.9 mg, 40.51 μmol, 32% yield) (Rt = 11.78 min) and *N*-(6-amino-5-methylpyridin-3-yl)-2-((2*S*,5*R*)-2-(2-((dimethylamino)methyl)benzo[*d*]thiazol-5-yl)-5-methylpiperidin-1-yl)-2-oxoacetamide (18.6 mg, 39.86 μmol, 31% yield) (Rt = 18.73 min). Rel Time for *N*-(6-amino-5-methylpyridin-3-yl)-2-((2*S*,5*R*)-2-(2-((dimethylamino)methyl)benzo[*d*]thiazol-5-yl)-5-methylpiperidin-1-yl)-2-oxoacetamide in analytical conditions (column: AS-H, Hexane-IPA-MeOH, 50-25-25, 0.6 mL / min as mobile phase) = 11.73 min and for **Compound 49** = 8.74 min.

N-(6-amino-5-methylpyridin-3-yl)-2-((2*S*,5*R*)-2-(2-((dimethylamino)methyl)benzo[*d*]thiazol-5-yl)-5-methylpiperidin-1-yl)-2-oxoacetamide:

Retention time: 11.73 min

^{1}H NMR (600 MHz, DMSO-d_6) δ 10.57 – 10.49 (m, 1H), 8.09 – 7.95 (m, 2H), 7.91 – 7.83 (m, 1H), 7.54 – 7.32 (m, 2H), 5.74 – 5.25 (m, 3H), 4.05 – 3.45 (m, 3H), 3.25 – 2.75 (m, 1H), 2.30 (s, 6H), 2.29 – 2.05 (m, 2H), 2.05 – 1.95 (m, 3H), 1.93 – 1.82 (m, 1H), 1.75 – 1.66 (m, 1H), 1.41 – 1.29 (m, 1H), 1.05 – 1.00 (m, 3H). LCMS (ESI): [M]+ m/z: calcd 466.2; found 467.2; Rt = 1.458 min.

Compound 49: Retention time: 8.74 min. ^{1}H NMR (600 MHz, DMSO-d_6) δ 10.57 – 10.47 (m, 1H), 8.08 – 7.94 (m, 2H), 7.91 – 7.83 (m, 1H), 7.52 – 7.32 (m, 2H), 5.71 – 5.24 (m, 3H), 4.05 – 3.44 (m, 3H), 3.28 – 2.76 (m, 1H), 2.30 (s, 6H), 2.28 – 2.06 (m, 2H), 2.04 – 1.96 (m, 3H), 1.91 – 1.84 (m, 1H), 1.76 – 1.67 (m, 1H), 1.40 – 1.31 (m, 1H), 1.04 – 1.01 (m, 3H). LCMS (ESI): [M]+ m/z: calcd 466.2; found 467.2; Rt = 1.460 min.

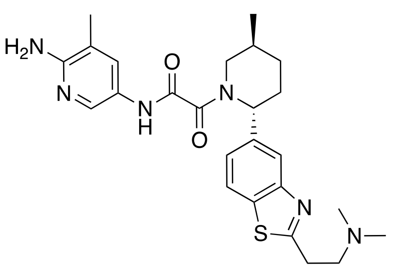

N-(6-amino-5-methylpyridin-3-yl)-2-((2*R*,5*S*)-2-(2-(2-(dimethylamino)ethyl)benzo[*d*]thiazol-5-yl)-5-methylpiperidin-1-yl)-2-oxoacetamide, **Compound 50**.

Amine: **Intermediate 50a**. Acid: **Intermediate 23acid**. Yield: 10 %. HPLC conditions: Column YMC Triart C18, 20 x 100 mm, 5 µm; 30 – 55 %, 0 - 5 min; MeOH / water + 0.1% NH_4OH 40 mL / min; loading pump 4 mL / min MeOH. LCMS (ESI): [M]+ m/z: calcd 480.2; found 481.2; Rt = 1.828 min.

Chiral separation: 57 mg (118.60 µmol) was chiral separated (Column: Chiralpak IA-II (250 x 20 mm, 5 µm), MeOH-IPA, 50-50, 10 mL / min) to obtain *N*-(6-amino-5-methylpyridin-3-yl)-2-((2*R*,5*S*)-2-(2-(2-(dimethylamino)ethyl)benzo[*d*]thiazol-5-yl)-5-methylpiperidin-1-yl)-2-oxoacetamide, **Compound 50** (18.7 mg, 38.91 µmol, 66% yield) (Rt = 33.47 min) and *N*-(6-amino-5-methylpyridin-3-yl)-2-((2*S*,5*R*)-2-(2-(2-(dimethylamino)ethyl)benzo[*d*]thiazol-5-yl)-5-methylpiperidin-1-yl)-2-oxoacetamide (20 mg, 41.61 µmol, 70% yield) (Rt = 57.96 min).

Rel Time for *N*-(6-amino-5-methylpyridin-3-yl)-2-((2*S*,5*R*)-2-(2-(2-(dimethylamino)ethyl)benzo[*d*]thiazol-5-yl)-5-methylpiperidin-1-yl)-2-oxoacetamide in analytical conditions (column: OJ-H, Hexane-IPA-MeOH, 60-20-20, 0.6 ml/min as mobile phase) = 15.71 min and for **Compound 50** = 37.52 min.

N-(6-amino-5-methylpyridin-3-yl)-2-((2*S*,5*R*)-2-(2-(2-(dimethylamino)ethyl)benzo[*d*]thiazol-5-yl)-5-methylpiperidin-1-yl)-2-oxoacetamide: Retention time: 15.71 min. ^{1}H NMR (600 MHz, DMSO-d_6) δ 1.02 – 1.04 (m, 3H), 1.22 – 1.40 (m, 2H), 1.66 – 1.90 (m, 3H), 2.04 – 2.37 (m, 9H), 2.68 – 2.78 (m, 2H), 3.20 – 3.23 (m, 2H), 3.47 (d, 1H), 4.03 (d, 1H), 5.27 – 5.69 (m, 3H), 7.32 – 7.50 (m, 2H), 7.84 – 8.04 (m, 3H), 10.56 (m, 1H). LCMS (ESI): [M]+ m/z: calcd 480.2; found 481.2; Rt = 1.818 min.

Compound 50: Retention time: 37.52 min. ^{1}H NMR (600 MHz, DMSO-d_6) δ 1.02 – 1.04 (m, 3H), 1.22 – 1.39 (m, 2H), 1.66 – 1.71 (m, 2H), 1.84 – 2.32 (m, 10H), 2.69 (m, 1H), 3.21 (m, 3H), 3.47 (d, 1H), 4.04 (d, 1H), 5.27 – 5.69 (m, 3H), 7.32 – 7.50 (m, 2H), 7.84 – 8.04 (m, 3H), 10.56 (m, 1H). LCMS (ESI): [M]+ m/z: calcd 480.2; found 481.2; Rt = 1.818 min.

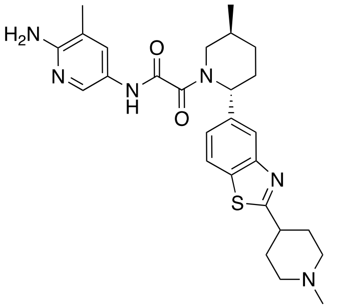

N-(6-amino-5-methylpyridin-3-yl)-2-((2*R*,5*S*)-5-methyl-2-(2-(1-methylpiperidin-4-yl)benzo[*d*]thiazol-5-yl)piperidin-1-yl)-2-oxoacetamide, **Compound 51**.

Amine: **Intermediate 51a**. Acid: **Intermediate 23acid**. Yield: 29 %. Flash chromatography (ISCO®; 4 g x 2 AgelaFlash® Silica Flash Column, DCM/MeOH with EtOAc from 0~18%, flow rate = 30 mL / min. LCMS (ESI) [M+H]+ m/z: calcd 507.3, found 507.2.

Chiral separation: 50 mg (0.0987 mmol) via chiral SFC (Instrument: Instrument: Thar800Q; Daicel Chiralpak IG (250 mm x 30 mm x 10 µm); Mobile phase: supercritical CO_2 / EtOH (0.1 % NH_3-H_2O, v %) = 50/50; Flow Rate: 80 mL / min; Column Temperature: 38 °C; Nozzle Pressure: 100 bar; Nozzle Temperature: 60 °C; Evaporator Temperature: 20 °C; Trimmer Temperature: 25 °C; Wavelength: 220 nm) to give *N*-(6-amino-5-methylpyridin-3-yl)-2-((2*R*,5*S*)-5-methyl-2-(2-(1-methylpiperidin-4-yl)benzo[*d*]thiazol-5-yl)piperidin-1-yl)-2-oxoacetamide, **Compound 51**
(peak 2, retention time: 1.620 min) as a white solid. ^{1}H NMR (400 MHz, MeOD-d_4) δ 7.58 - 8.15 (m, 4H), 7.35 - 7.52 (m, 1H), 5.42 - 5.89 (m, 1H), 3.66 - 4.14 (m, 1H), 3.43 (br d, *J* = 12.5 Hz, 1H), 3.14 (br s, 1H), 3.01 (br d, *J* = 11.0 Hz, 2H), 2.34 (s, 3H), 2.13 - 2.31 (m, 7H), 1.87 - 2.11 (m, 6H), 1.47 (br d, *J* = 9.8 Hz, 1H), 1.14 (d, *J* = 6.8 Hz, 3H). LCMS (ESI): [M+H]+ m/z: calcd 507.3, found 507.2; HPLC: 100% at 220nm, 100% at 254 nm; 100% ee.

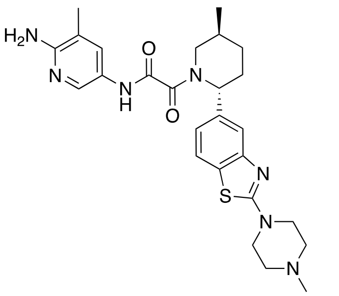

N-(6-amino-5-methylpyridin-3-yl)-2-((2*R*,5*S*)-5-methyl-2-(2-(4-methylpiperazin-1-yl)benzo[*d*]thiazol-5-yl)piperidin-1-yl)-2-oxoacetamide, **Compound 52**.

Amine: **Intermediate 52a**. Acid: **Intermediate 23acid**. Yield: 18 %. HPLC conditions: Column YMC Triart C18 20 x 100 mm, 5 µm; 0 – 1 – 5 min, 30 – 30 - 65 %; water / MeCN + 0.1% NH_4OH 40 mL / min; loading pump 4 mL / min MeCN. **Compound 52**: ^{1}H NMR (600 MHz, DMSO-d_6) δ 1.02 (m, 3H), 1.34

(m, 1H), 1.71 (m, 1H), 1.86 (m, 1H), 2.03 (m, 3H), 2.13 (m, 1H), 2.21 (s, 4H), 2.42 (t, 4H), 3.01 (dd, 1H), 3.52 (m, 5H), 5.60 (m, 3H), 7.04 (dd, 1H), 7.43 (m, 2H), 7.75 (m, 1H), 8.01 (m, 1H), 10.52 (m, 1H). LCMS (ESI): [M]+ m/z: calcd 507.2; found 508.2; Rt = 1.998 min.

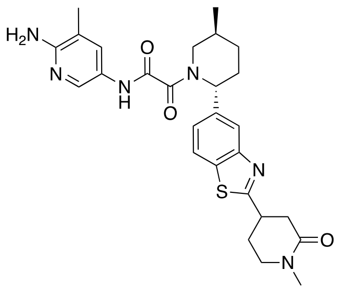

N-(6-amino-5-methylpyridin-3-yl)-2-((2*R*,5*S*)-5-methyl-2-(2-(1-methyl-2-oxopiperidin-4-yl)benzo[*d*]thiazol-5-yl)piperidin-1-yl)-2-oxoacetamide, **Compound 53**.
Amine: **Intermediate 53a**. Acid: **Intermediate 23acid**. Yield: 12 %. HPLC conditions: Column YMC Triart C18 20 x 100 mm, 5 µm; 0 – 1 – 6 min, 40 – 40 - 65 %; water / MeOH + 0.1% NH_4OH, flow 30 mL / min; loading pump 4 mL / min MeOH. **Compound 53**: ^{1}H NMR (600 MHz, DMSO-d_6) δ 1.02 – 1.04 (m, 3H), 1.31 – 1.39 (m, 1H), 1.69 – 1.71 (m, 1H), 1.84 – 2.31 (m, 9H), 2.60 – 2.82 (m, 5H), 3.39 – 4.05 (m, 4H), 5.27 – 5.69 (m, 3H), 7.36 – 7.50 (m, 2H), 7.88 – 8.09 (m, 3H), 10.52 – 10.56 (m, 1H). LCMS (ESI): [M]+ m/z: calcd 520.2; found 521.2; Rt = 2.359 min.

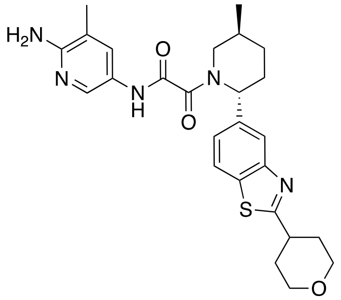

N-(6-amino-5-methylpyridin-3-yl)-2-((2*R*,5*S*)-5-methyl-2-(2-(tetrahydro-2*H*-pyran-4-yl)benzo[*d*]thiazol-5-yl)piperidin-1-yl)-2-oxoacetamide, **Compound 54**.
Amine: **Intermediate 54a**. Acid: **Intermediate 23acid**. (Column: SepaFlash® Sphercial C18, 40 g, 40-60 µm, 120 Å; MeCN / water (0.5 % NH_3-H_2O) with MeCN from 0 - 60%, 25 mL / min; Wavelength: 220 nm, 254 nm) to afford a racemic product which was separated by chiral SFC (Instrument: Berger, Multigr AM-II; Column: Daicel Chiralcel OJ-H (250 mm x 30 mm, 5 µm); Mobile phase: supercritical CO_2 / MeOH (0.1% NH_3-H_2O, v %) = 40 / 60; Flow Rate: 60 mL / min; Column Temperature: 38 °C; Nozzle Pressure: 100 bar; Nozzle Temperature: 60 °C; Evaporator Temperature: 20 °C; Trimmer Temperature: 25 °C; Wavelength: 220 nm, 254nm) to afford *N*-(6-amino-5-methylpyridin-3-yl)-2-((2*S*,5*R*)-5-methyl-2-(2-(tetrahydro-2*H*-pyran-4-yl)benzo[*d*]thiazol-5-yl)piperidin-1-yl)-2-oxoacetamide (peak 4, retention time = 8.365 min) and *N*-(6-amino-5-methylpyridin-3-yl)-2-((2*R*,5*S*)-5-methyl-2-(2-(tetrahydro-2*H*-pyran-4-yl)benzo[*d*]thiazol-5-yl)piperidin-1-yl)-2-oxoacetamide, **Compound 54** (peak 2, retention time = 4.575 min).
N-(6-amino-5-methylpyridin-3-yl)-2-((2*S*,5*R*)-5-methyl-2-(2-(tetrahydro-2*H*-pyran-4-yl)benzo[*d*]thiazol-5-yl)piperidin-1-yl)-2-oxoacetamide (30 mg, brown solid). ^{1}H NMR (600 MHz, DMSO-d_6) δ 10.29 (br s, 1 H), 7.98 - 8.05 (m, 2 H), 7.90 (s, 1 H), 7.49 (br s, 1 H), 7.39 (br d, *J* = 8.3 Hz, 1 H), 5.59 (br s, 1 H), 5.35 (br s, 2 H), 3.90 - 4.03 (m, 3 H), 3.52 (td, *J* = 11.5, 2.1 Hz, 3 H), 3.35 - 3.44 (m, 2 H), 2.14 - 2.34 (m, 2 H), 2.05 (br s, 5 H), 1.75 - 1.96 (m, 4 H), 1.37 (br d, *J* = 10.3 Hz, 1 H), 1.06 (d, *J* = 7.0 Hz, 3 H). LCMS (ESI): [M+H]+ m/z: calcd 494.2, found 494.1; HPLC: 100% at 220nm, 100% at 254nm; 100% ee.
N-(6-amino-5-methylpyridin-3-yl)-2-((2*R*,5*S*)-5-methyl-2-(2-(tetrahydro-2*H*-pyran-4-yl)benzo[*d*]thiazol-5-yl)piperidin-1-yl)-2-oxoacetamide, **Compound 54** (30 mg, brown solid). ^{1}H NMR (600 MHz, DMSO-d_6) δ 10.35 (br s, 1 H), 8.08 (br d, *J* = 8.3 Hz, 2 H), 7.96 (s, 1 H), 7.54 (br s, 1 H), 7.45 (br d, *J* = 7.8 Hz, 1 H), 5.66 (br s, 1 H), 5.41 (br s, 2 H), 4.02 (br d, *J* = 11.0 Hz, 3 H), 3.53 - 3.66 (m, 3 H), 2.19 - 2.43 (m, 2 H), 2.11 (br s, 6 H), 1.77 - 2.00 (m, 4 H), 1.42 (br d, *J* = 11.3 Hz, 1 H), 1.12 (d, *J* = 7.0 Hz, 3 H). LCMS (ESI): [M+H]+ m/z: calcd 494.2, found 494.1; HPLC: 100% at 220 nm, 100% at 254nm; 95.9% ee.

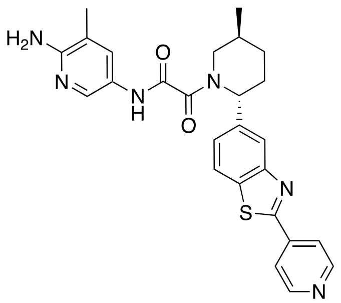

N-(6-amino-5-methylpyridin-3-yl)-2-((2*R*,5*S*)-5-methyl-2-(2-(pyridin-4-yl)benzo[*d*]thiazol-5-yl)piperidin-1-yl)-2-oxoacetamide, **Compound 55**.
Amine: **Intermediate 55a**. Acid: **Intermediate 23acid**. Yield 72 %. Preparative HPLC (Instrument: Gilson GX-281 Liquid Handler, Gilson 322 Pump, Gilson 156 UV Detector; Column: Waters Xbridge 150 × 25 mm × 5 µm; Mobile phase A: H_2O with 0.05 % NH_3-H_2O (v%); Mobile phase B: MeCN; Gradient: B from 31% to 61% in 9.5 min, hold 100% B for 2.5 min; Flow Rate: 25 mL / min; Column Temperature: 30 °C; Wavelength: 220 nm, 254 nm).

Residual isomers removed with chiral SFC: 50 mg (0.103 mmol) (Instrument: Thar 800Q; Daicel Chiralpak IG (250 mm x 30 mm, 10 μm); Mobile phase: supercritical CO_2 / EtOH (0.1% NH_3-H_2O, v %) = 45 / 55; Flow Rate: 80 mL / min; Column Temperature: 38 °C; Nozzle Pressure: 100 bar; Nozzle Temperature: 60 °C; Evaporator Temperature: 20 °C; Trimmer Temperature: 25 °C; Wavelength: 220 nm) to give *N*-(6-amino-5-methylpyridin-3-yl)-2-((2*R*,5*S*)-5-methyl-2-(2-(pyridin-4-yl)benzo[*d*]thiazol-5-yl)piperidin-1-yl)-2-oxoacetamide, **Compound 55** (50 mg, single known enantiomer with trans relative chemistry, peak 2, retention time: 2.682 min, white solid). ^{1}H NMR (400 MHz, MeOD-d_4) δ 8.75 (br d, *J* = 5.0 Hz, 2 H), 8.13 (br d, *J* = 12.1 Hz, 5 H), 7.30 - 7.78 (m, 2 H), 5.45 - 5.97 (m, 1 H), 3.72 - 4.18 (m, 1 H), 3.37 - 3.54 (m, 1 H), 3.22 (br s, 1 H), 2.39 (br s, 2 H), 2.19 (br s, 2 H), 1.92 - 2.09 (m, 2 H), 1.51 (br d, *J* = 11.9 Hz, 1 H), 1.18 (br d, *J* = 6.8 Hz, 3 H). LCMS (ESI): [M+H]+ m/z: calcd 487.2, found 487.2; HPLC: 100% at 220nm, 100% at 254nm; 100% ee.

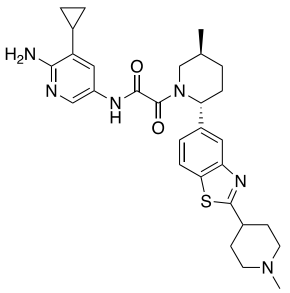

N-(6-amino-5-cyclopropylpyridin-3-yl)-2-((2*R*,5*S*)-5-methyl-2-(2-(1-methylpiperidin-4-yl)benzo[*d*]thiazol-5-yl)piperidin-1-yl)-2-oxoacetamide, **Compound 56**.

Amine: **92**. Acid: **Intermediate 3a**. Purified by preparative HPLC (Instrument: Gilson GX-281 Liquid Handler, Gilson 322 Pump, Gilson 156 UV Detector; Column: Waters XBridge 150 × 25 mm × 5 μm; Mobile phase A: H_2O with 0.05 % NH_3•H_2O (v %); Mobile phase B: MeCN; Gradient: B from 25 % to 55 % in 7.8 min, hold 100 % B for 2.5 min; Flow Rate: 25 mL / min; Column Temperature: 30 °C; Wavelength: 220 nm, 254 nm) to afford the desired oxamide (90 mg, 31% yield) as white solid. LCMS (ESI) [M+H]+ m/z: calcd 533.3, found 533.4. Residual isomers were removed with SFC (50 mg, 0.0939 mmol) Instrument: Thar 800Q; Column: Daicel Chiralpak IG (250 mm x 30 mm, 10 μm); Mobile phase: supercritical CO_2 / EtOH (0.1 % NH_3-H_2O, v %) = 70 / 30; Flow Rate: 80 mL / min; Column Temperature: 38 °C; Nozzle Pressure: 100 bar; Nozzle Temperature: 60 °C; Evaporator Temperature: 20 °C; Trimmer Temperature: 25 °C; Wavelength: 220 nm to give *N*-(6-amino-5-cyclopropylpyridin-3-yl)-2-((2*R*,5*S*)-5-methyl-2-(2-(1-methylpiperidin-4-yl)benzo[*d*]thiazol-5-yl)piperidin-1-yl)-2-oxoacetamide, **Compound 56** (peak 2, retention time = 5.200 min).
(40 mg, white solid). ^{1}H NMR (400 MHz, MeOD-d_4) δ 8.12 (br s, 1 H), 7.88 - 8.03 (m, 2 H), 7.20 - 7.85 (m, 2 H), 5.47 - 5.89 (m, 1 H), 3.67 - 4.17 (m, 1 H), 3.36 - 3.54 (m, 1 H), 3.15 (br s, 1 H), 3.03 (br d, *J* = 12.0 Hz, 2 H), 2.13 - 2.45 (m, 9 H), 1.85 - 2.05 (m, 4 H), 1.42 - 1.76 (m, 2 H), 1.16 (d, *J* = 7.0 Hz, 3 H), 0.86 - 1.03 (m, 2 H), 0.46 - 0.66 (m, 2 H). LCMS (ESI): [M+H]+ m/z: calcd 533.3, found 533.3; HPLC: 99.45 % at 220 nm, 100 % at 254 nm; 100 % ee.

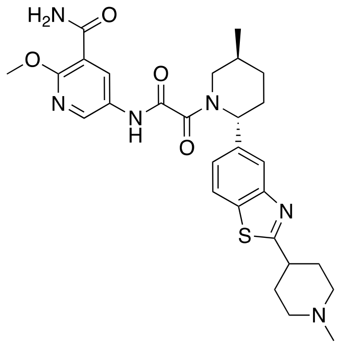

2-methoxy-5-(2-((2*R*,5*S*)-5-methyl-2-(2-(1-methylpiperidin-4-yl)benzo[*d*]thiazol-5-yl)piperidin-1-yl)-2-oxoacetamido)nicotinamide, **Compound 58**.

Amine: **92**. Acid: **Intermediate 48a**. Yield: 23 %. HPLC purification: (Instrument: Gilson GX-281 Liquid Handler, Gilson 322 Pump, Gilson 156 UV Detector; Column: Durashell 150 × 25 mm × 5 μm; Mobile phase A: H_2O with 0.05 % NH_3-H_2O (v%); Mobile phase B: MeCN; Gradient: B from 28 % to 58 % in 9.5 min, hold 100% B for 2.0 min; Flow Rate: 25 mL / min; Column Temperature: 30 °C; Wavelength: 220 nm, 254 nm) to afford 2-methoxy-5-(2-((2*R*,5*S*)-5-methyl-2-(2-(1-methylpiperidin-4-yl)benzo[*d*]thiazol-5-yl)piperidin-1-yl)-2-oxoacetamido)nicotinamide, **Compound 58** as a white solid. ^{1}H NMR (400 MHz, MeOD-d_4) δ 8.59 - 8.74 (m, 1 H), 8.33 - 8.56 (m, 1 H), 7.92 - 8.06 (m, 2 H), 7.43 - 7.54 (m, 1 H), 5.44 - 5.93 (m, 1 H), 4.02 - 4.17 (m, 3 H), 3.78 (br s, 1 H), 3.13 - 3.28 (m, 2 H), 3.03 - 3.12 (m, 2 H), 2.19 - 2.44 (m, 9 H), 1.90 - 2.11 (m, 4 H), 1.43 - 1.56 (m, 1 H), 1.17 (d, *J* =7.0 Hz, 3 H). LCMS (ESI): [M+H]+ m/z: calcd 551.1, found 551.2; HPLC: 100% at 220nm, 100% at 254nm; 99.0% ee.

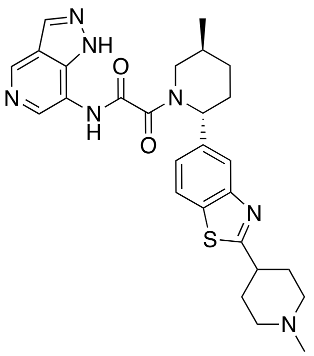

2-((2*R*,5*S*)-5-methyl-2-(2-(1-methylpiperidin-4-yl)benzo[*d*]thiazol-5-yl)piperidin-1-yl)-2-oxo-*N*-(1*H*-pyrazolo[4,3-*c*]pyridin-7-yl)acetamide, **Compound 62**.

Amine: **92**. Acid: **Intermediate 62a**. SEM-protected: Flash chromatography (ISCO®; 4 g AgelaFlash® Silica Flash Column, DCM / MeOH with MeOH from 0 ~ 25 %, flow rate = 30 mL / min, 254 nm) to afford 2-[(5*S*)-5-methyl-2-[2-(1-methyl-4-piperidyl)-1,3-benzothiazol-5-yl]-1-piperidyl]-2-oxo-*N*-[1-(2-trimethylsilylethoxymethyl)pyrazolo[4,3-c]pyridin-7-yl]acetamide (43% yield) as yellow oil. LCMS (ESI) [M+H]+ m/z: calcd 648.3, found 648.4. A mixture of 2-[(5*S*)-5-methyl-2-[2-(1-methyl-4-piperidyl)-1,3-benzothiazol-5-yl]-1-piperidyl]-2-oxo-*N*-[1-(2-trimethylsilylethoxymethyl)pyrazolo[4,3-c]pyridin-7-yl]acetamide (50.0 mg, 0.0772 mmol) and TFA (2 mL, 25.9 mmol) in DCM (2 mL) was stirred at 20 °C for 12 h. The mixture was concentrated under reduced pressure to give a crude product, which was purified by preparative HPLC (Instrument: Gilson GX-281 Liquid Handler, Gilson 322 Pump, Gilson 156 UV Detector; Column: Phenomenex Gemini-NX 80 x 40 mm x 3 µm; Mobile phase A: H_2O with 0.05 % NH_3-H_2O+10 mM NH_4HCO_3 (v %); Mobile phase B: MeCN; Gradient: B from 25 % to 55 % in 9.5 min, hold 100 % B for 0 min; Flow Rate: 25 mL / min; Column Temperature: 30° C; Wavelength: 220 nm, 254 nm) to afford 2-((2*R*,5*S*)-5-methyl-2-(2-(1-methylpiperidin-4-yl)benzo[*d*]thiazol-5-yl)piperidin-1-yl)-2-oxo-*N*-(1*H*-pyrazolo[4,3-*c*]pyridin-7-yl)acetamide (20 mg, 50% yield) as a white solid. LCMS (ESI) [M+H]+ m/z: calcd 518.2, found 518.3; HPLC: 100%@254nm. Residual isomers were removed by SFC (Instrument: Berger, Multigr AM-II; Column: Daicel Chiralpak AD, 250 mm × 30 mm × 10 µm; Mobile phase: supercritical CO_2 / EtOH (0.1 % NH_3-H_2O, v %) = 60 / 40; Flow Rate: 80 mL / min; Column Temperature: 38° C; Nozzle Pressure: 100 bar; Nozzle Temperature: 60° C; Evaporator Temperature: 20 °C; Trimmer Temperature: 25 °C; Wavelength: 220 nm) to afford 2-((2*R*,5*S*)-5-methyl-2-(2-(1-methylpiperidin-4-yl)benzo[*d*]thiazol-5-yl)piperidin-1-yl)-2-oxo-*N*-(1*H*-pyrazolo[4,3-*c*]pyridin-7-yl)acetamide, **Compound 62** (8 mg, retention time: 5.036 min, white solid). ^{1}H NMR (400 MHz, methanol-*d$_4$*) δ 8.81 - 9.11 (m, 1H), 8.21 - 8.57 (m, 2H), 7.82 - 8.09 (m, 2H), 7.48 (br s, 1H), 5.63 - 6.03 (m, 1H), 3.91 - 4.23 (m, 2H), 3.50 (br d, *J* = 14.1 Hz, 1H), 2.98 - 3.22 (m, 2H), 2.13 - 2.45 (m, 8H), 1.88 - 2.09 (m, 4H), 1.28 - 1.60 (m, 2H), 1.18 (br d, *J* = 6.6 Hz, 3H). LCMS (ESI): [M+H]+ m/z: calcd 518.2, found 518.3; HPLC: 100 % at 220 nm, 100% at 254 nm; 100 % ee.

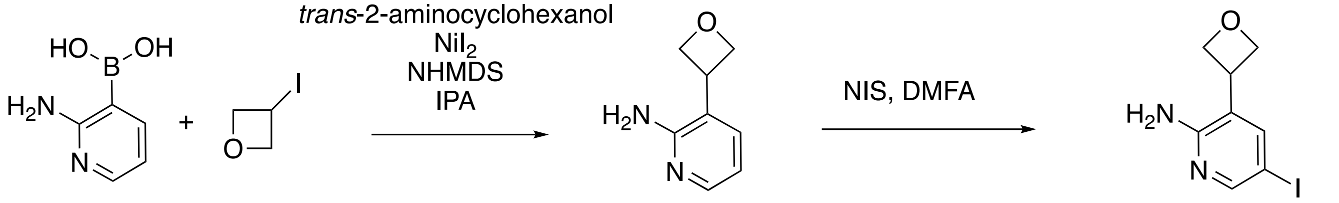

Intermediate 4a

3-(oxetan-3-yl)pyridin-2-amine.

trans-2-aminocyclohexanol (198.13 mg, 1.72 mmol) and nickel (II) iodide (537.58 mg, 1.72 mmol) were added to a solution of (2-amino-3-pyridyl)boronic acid (5 g, 28.67 mmol, HCl) in isopropyl alcohol (50 mL). Sodium bis(trimethylsilyl)amide (40% in THF) (39.43 g, 86.01 mmol, 44.11 mL) was added dropwise followed by 3-iodooxetane (7.91 g, 43.01 mmol, 3.70 mL). The reaction flask was purged with Ar and the resulting mixture was stirred at 75 °C for 8 h. The volatiles were removed under reduced pressure and the residue was purified by gradient column chromatography (SiO_2, $CHCl_3$ / ACN, from 0~100%, flow rate =60mL/min, cv=5), affording 3-(oxetan-3-yl)pyridin-2-amine (1 g, 6.66 mmol, 23% yield). LCMS (ESI): [M+H]+ m/z: calcd 150.09, found 151.0.

5-iodo-3-(oxetan-3-yl)pyridin-2-amine, **Intermediate 4a**.

3-(oxetan-3-yl)pyridin-2-amine (0.8 g, 5.33 mmol) was dissolved in DMFA (15 mL) followed by portionwise addition of 1-iodopyrrolidine-2,5-dione (1.60 g, 7.11 mmol). The reaction mixture was stirred overnight and concentrated under reduced pressure. The residue was dissolved in DCM and the organic layer was washed with $Na_2S_2O_3$ solution and water multiple times. DCM layer was separated, dried over Na_2SO_4, and concentrated under reduced pressure to afford pure 5-iodo-3-(oxetan-3-yl)pyridin-2-amine, **Intermediate 4a** (0.6 g, 2.17 mmol, 41% yield). LCMS (ESI): [M+H]+ m/z: calcd 275.98, found 277.0.

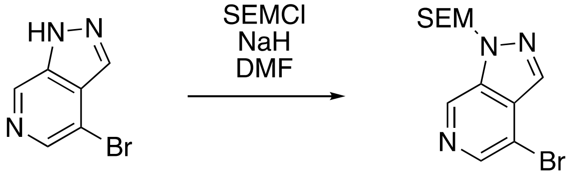

Intermediate 9a

4-bromo-1-((2-(trimethylsilyl)ethoxy)methyl)-1*H*-pyrazolo[3,4-*c*]pyridine, **Intermediate 9a**.
Sodium hydride (127.71 mg, 3.33 mmol, 60 % dispersion in mineral oil) was added portionwise to a solution of 4-bromo-1*H*-pyrazolo[3,4-c]pyridine (600 mg, 3.03 mmol) in DMF (15 mL) at 0 °C . When H_2 evolution ceased, 2-(chloromethoxy)ethyl-trimethyl-silane (530.42 mg, 3.18 mmol, 563.08 µL) was added dropwise the the mixture was stirred at 0 °C for 3 h after which it was diluted with water (60 mL) and extracted with MTBE (50 mL). The organic layer was washed successively with water (20 mL) and brine (20 mL), dried over Na_2SO_4, and concentrated under reduced pressure, affording 4-bromo-1-((2-(trimethylsilyl)ethoxy)methyl)-1*H*-pyrazolo[3,4-*c*]pyridine, **Intermediate 9a** (940 mg, 2.86 mmol, 95% yield). LCMS (ESI): [M]+ m/z: calcd 328.2; found 329.2; Rt = 1.465 min.

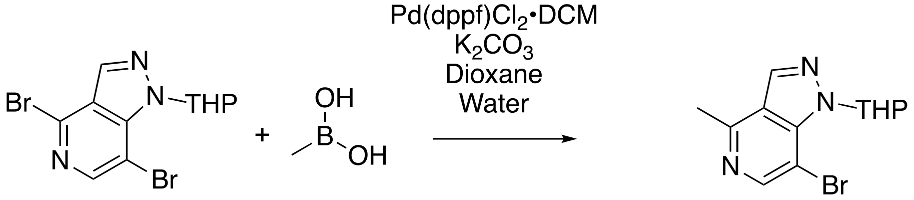

Intermediate 12a

7-bromo-4-methyl-1-(tetrahydro-2*H*-pyran-2-yl)-1*H*-pyrazolo[4,3-*c*]pyridine, **Intermediate 12a**.
Methylboronic acid (332 mg, 5.54 mmol) was added portionwise to a solution of 4,7-dibromo-1-tetrahydropyran-2-yl-pyrazolo[4,3-*c*]pyridine (1.00 g, 2.77 mmol), Pd(dppf)Cl_2 (50.7 mg, 69.3 µmol) and potassium carbonate - granular (1.91 g, 13.9 mmol) in water (5 mL) / dioxane (25 mL). The reaction mixture was stirred at 100 °C for 72 h. The resulting mixture was filtered and concentrated in vacuo. The residue was purified by column chromatography (Companion; 40g SiO_2; $CHCl_3$- MeCN from 0 – 20 %) to afford 7-bromo-4-methyl-1-(tetrahydro-2*H*-pyran-2-yl)-1*H*-pyrazolo[4,3-*c*]pyridine, **Intermediate 12a** (0.36 g, 1.22 mmol, 44 % yield) as a beige solid. LCMS (ESI): [M+H]+ m/z: calcd 296.05 and 298.05; found 298.0; Rt = 0.874.

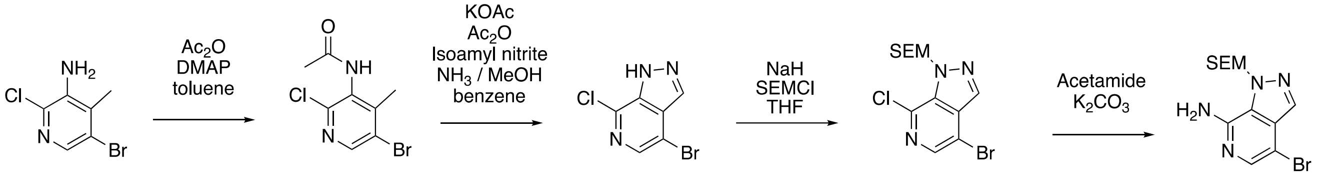

Intermediate 13a

N-(5-bromo-2-chloro-4-methylpyridin-3-yl)acetamide.
5-Bromo-2-chloro-4-methyl-pyridin-3-amine (8 g, 36.12 mmol), DMAP (4.41 g, 36.12 mmol) and acetic anhydride (5.90 g, 57.79 mmol, 5.46 mL) were stirred in toluene (100 mL) and heated for 12 h at 100 °C. The reaction mixture was concentrated under vacuum and purified by column silica gel chromatography (eluent gradient chloroform-acetonitrile) to afford *N*-(5-bromo-2-chloro-4-methylpyridin-3-yl)acetamide (6.3 g, 23.91 mmol, 66 % yield). LCMS (ESI): [M]+ m/z: calcd 263.2; found 264.2; Rt = 0.948 min.

4-bromo-7-chloro-1*H*-pyrazolo[3,4-*c*]pyridine.
Potassium acetate (3.52 g, 35.86 mmol, 2.24 mL) and acetic anhydride (7.32 g, 71.72 mmol, 6.77 mL) were added to a solution of *N*-(5-bromo-2-chloro-4-methylpyridin-3-yl)acetamide (6.3 g, 23.91 mmol) in dry benzene (200 mL). The reaction mixture was heated at reflux and isoamyl nitrite (5.60 g, 47.81 mmol, 6.40 mL) was added. Reflux was continued for 16 h. The insoluble material was filtered off and the filtrate was evaporated under vacuum. The residue was dissolved in a saturated solution of ammonium in MeOH (407.15 mg, 23.91 mmol) and the resulting solution was stirred at room temperature for 2 h, then concentrated under vacuum. The crude product was dissolved in 200 mL EtOAc and washed with water. The organic phase was dried over Na_2SO_4 and concentrated under vacuum to give 4-bromo-7-chloro-1*H*-pyrazolo[3,4-*c*]pyridine (4.3 g, crude). LCMS (ESI): [M]+ m/z: calcd 232.2; found 233.2; Rt = 1.018 min.

4-bromo-7-chloro-1-((2-(trimethylsilyl)ethoxy)methyl)-1*H*-pyrazolo[3,4-*c*]pyridine.
Sodium hydride (0.9 g, 22.50 mmol, 60 % dispersion in mineral oil) was added portionwise to a solution of 4-bromo-7-chloro-1*H*-pyrazolo[3,4-*c*]pyridine (4.3 g, 14.80 mmol) in dry THF (50 mL). After 30 min the reaction mixture was cooled to 5 °C with an ice-water bath and 2-(chloromethoxy)ethyl-trimethyl-silane (3.21 g, 19.24 mmol, 3.40 mL) in 10 mL THF was added dropwise and stirred overnight at room temperature. The reaction mixture was diluted with water and extracted with DCM (2 x 100 mL), dried over Na_2SO_4, and concentrated under vacuum to give 4-bromo-7-chloro-1-((2-(trimethylsilyl)ethoxy)methyl)-1*H*-pyrazolo[3,4-*c*]pyridine (3.5 g, crude) which was used as is. LCMS (ESI): [M]+ m/z: calcd 362.2; found 363.2; Rt = 1.749 min.

4-bromo-1-((2-(trimethylsilyl)ethoxy)methyl)-1*H*-pyrazolo[3,4-*c*]pyridin-7-amine, **Intermediate 13a**.
4-bromo-7-chloro-1-((2-(trimethylsilyl)ethoxy)methyl)-1*H*-pyrazolo[3,4-*c*]pyridine (2 g, 4.14 mmol), acetamide (3.66 g, 62.03 mmol), and potassium carbonate, anhydrous, 99 % (2.86 g, 20.68 mmol, 1.25 mL) was stirred at 210 °C for 5 h. After cooling to room temperature, the reaction mixture was poured into water and extracted with DCM, dried over sodium sulfate, evaporated, and purified by HPLC: 2 - 10 min 30 - 65 % MeCN + FA , 30 mL / min) to afford 4-bromo-1-((2-(trimethylsilyl)ethoxy)methyl)-1*H*-pyrazolo[3,4-*c*]pyridin-7-amine, **Intermediate 13a**. LCMS (ESI): [M]+ m/z: calcd 343.2; found 344.2; Rt = 3.022 min.

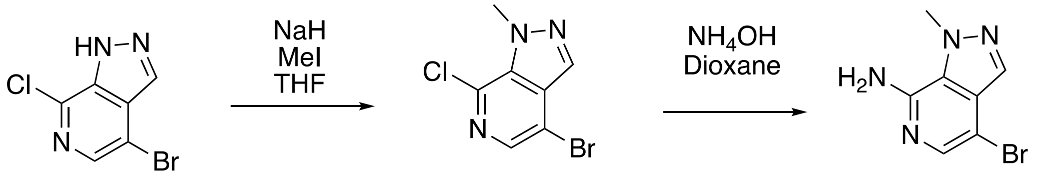

Intermediate 15a

4-bromo-7-chloro-1-methyl-1*H*-pyrazolo[3,4-*c*]pyridine.
Sodium hydride (941.86 mg, 23.55 mmol, 60 % dispersion in mineral oil) was added portionwise to the solution of 4-bromo-7-chloro-1*H*-pyrazolo[3,4-*c*]pyridine (3.6 g, 15.49 mmol) in dry THF (48.75 mL). After 30 min iodomethane (2.86 g, 20.13 mmol, 1.25 mL) was added dropwise, then stirred overnight at room temperature. The reaction mixture was diluted with water and extracted with DCM (2 x 15 mL), dried over anhydrous sodium sulfate, and concentrated in vacuo. The residue was purified by column chromatography - Interchim; 120 g SiO_2 chloroform / ACN with ACN from 0 ~ 30 %, flow rate =85 mL / min, to afford 4-bromo-7-chloro-1-methyl-1*H*-pyrazolo[3,4-*c*]pyridine (1.2 g, 4.87 mmol, 31% yield) as an off-white solid.

4-bromo-1-methyl-1*H*-pyrazolo[3,4-*c*]pyridin-7-amine, **Intermediate 15a**.
4-bromo-7-chloro-1-methyl-1*H*-pyrazolo[3,4-*c*]pyridine (1.20 g, 4.87 mmol) was dissolved in MeOH (5 mL) and ammonia (1.24 g, 73.02 mmol) was bubbled through the solution. The solution was then stirred at 125 °C for 96 h, then cooled and concentrated. The residue was purified by column chromatography (Interchim, 40 g SiO_2, ethyl acetate / methanol, flow rate = 40 mL / min) to afford 4-bromo-1-methyl-1*H*-pyrazolo[3,4-*c*]pyridin-7-amine, **Intermediate 15a** (0.2 g, 880.82 mol, 18% yield) as a beige solid. LCMS (ESI): [M+H]+ m/z: calcd 227.0; found 227.0; Rt = 0.530 min.

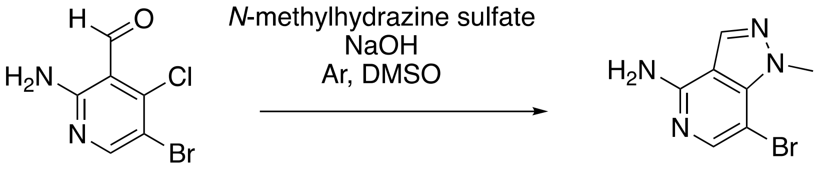

Intermediate 16a

7-bromo-1-methyl-1*H*-pyrazolo[4,3-*c*]pyridin-4-amine, **Intermediate 16a**.
2-amino-5-bromo-4-chloro-pyridine-3-carbaldehyde (1.3 g, 5.52 mmol), *N*-methylhydrazine sulfate (1.19 g, 8.28 mmol), and NaOH (1.10 g, 27.60 mmol, 518.40 µL) were stirred in DMSO under Ar, then stirred for 18 h at 90 °C. The reaction mixture was then poured into water, extracted three times with EtOAc, and the combined organics were washed with water (2 x 25 mL), brine, dried, and evaporated to give a residue which was purified by HPLC (10 – 40 % 2 - 7 min; flow 30 mL / min water-acetonitrile + NH_3 (loading pump 4 mL / min; acetonitrile); column XBridge C18 19 x 100 mm) to afford 7-bromo-1-methyl-1*H*-pyrazolo[4,3-*c*]pyridin-4-amine, **Intermediate 16a** (46.4 mg, 204.35 µmol, 4% yield). LCMS (ESI): [M+H]+ m/z: calcd 229.2; found 229.2; Rt = 1.443 min.

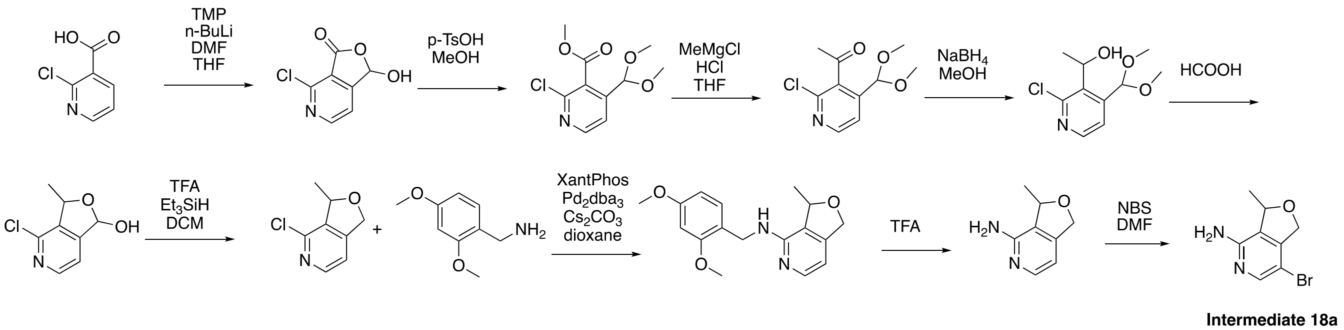

Intermediate 18a

4-chloro-1-hydroxyfuro[3,4-*c*]pyridin-3(1*H*)-one.
2,2,6,6-Tetramethylpiperidine (32.28 g, 228.49 mmol, 38.56 mL) was dissolved in THF (300 mL) and cooled to -10 °C. n-Butyllithium (228.49 mmol, 92.23 mL, 2.5 M in hexanes) was added dropwise at -10 °C, and the resulting mixture was stirred for 10 min. The mixture was cooled to -80 °C and 2-chloropyridine-3-carboxylic acid (12 g, 76.16 mmol) was added then it was stirred at -50 °C for 3 h, then cooled to -80 °C. DMF (33.40 g, 456.99 mmol, 35.38 mL) was added and it was stirred for 1.5 h then allowed to warm to room temperature and stirred overnight. Water (200 mL) was added to the reaction and then it was extracted with EtOAc (3 × 150 mL). The aqueous layer was acidified to pH 2 and then it was extracted with EtOAc (3 × 150 mL). The combined organic layers were dried over anhydrous sodium sulfate and filtered. The filtrate was concentrated in vacuo. The residue was purified by column chromatography (gradient ACN in chloroform from 8% to 100%) to obtain 4-chloro-1-hydroxyfuro[3,4-*c*]pyridin-3(1*H*)-one (6.5 g, 35.03 mmol, 46% yield) as a yellow solid. LCMS (ESI): [M+H]+ m/z: calcd 186.0; found 186.0; Rt = 0.663 min.

Methyl 2-chloro-4-(dimethoxymethyl)nicotinate.

4-chloro-1-hydroxyfuro[3,4-*c*]pyridin-3(1*H*)-one (6.3 g, 33.95 mmol) was dissolved in MeOH (125 mL) and p-toluenesulfonic acid monohydrate (645.81 mg, 3.40 mmol, 520.81 µL) was added. The resulting mixture was heated to reflux and stirred overnight then concentrated in vacuo. The residue was dissolved in EtOAc (150 mL) and washed with aq. $NaHCO_3$ solution (2 × 50 mL), dried over anhydrous sodium sulfate, and filtered. The filtrate was concentrated in vacuo to obtain methyl 2-chloro-4-(dimethoxymethyl)nicotinate (7.08 g, 28.80 mmol, 85% yield) as a yellow oil. LCMS (ESI): [M+H]+ m/z: calcd 246.06; found 246.2; Rt = 1.097 min.

1-(2-chloro-4-(dimethoxymethyl)pyridin-3-yl)ethan-1-one.
Methyl 2-chloro-4-(dimethoxymethyl)nicotinate (5.64 g, 22.94 mmol) was dissolved in THF (170 mL) and cooled to 0 °C in an ice / methanol bath under an Ar atmosphere. Methylmagnesium chloride (3 M in THF, 68.81 mmol) was added dropwise and the bath was removed. The resulting mixture was allowed to warm to room temperature and stirred for 30 min, after which the reaction mixture was heated at 70 °C (oil bath) overnight then cooled in an ice bath and hydrochloric acid, 36% w/w aq. soln. (6.97 g, 68.81 mmol, 6 mL, 36 % purity) (+40 mL of water) was added dropwise. The aqueous layer was extracted with EtOAc (2 × 200 mL) and the combined organic layers were washed with brine (150 mL), dried over anhydrous sodium sulfate, and filtered. The filtrate was concentrated in vacuo to obtain 1-(2-chloro-4-(dimethoxymethyl)pyridin-3-yl)ethan-1-one (4.92 g, 21.41 mmol, 93% yield) as a thick, red oil which was used in the next step without further purification. LCMS (ESI): [M+H]+ m/z: calcd 230.06; found 230.2; Rt = 1.052 min.

1-(2-chloro-4-(dimethoxymethyl)pyridin-3-yl)ethan-1-ol.
1-(2-chloro-4-(dimethoxymethyl)pyridin-3-yl)ethan-1-one (4.77 g, 20.76 mmol) was dissolved in MeOH (100 mL) and the resulting mixture was cooled to 0 °C in an ice / methanol bath. Sodium borohydride (2.75 g, 72.66 mmol, 2.56 mL) was added portionwise and the reaction mixture was allowed to warm to room temperature and stirred overnight after which it was concentrated in vacuo. Water (100 mL) was added and the resulting mixture was extracted with DCM (2 × 100 mL) and the combined organic layers were dried over anhydrous sodium sulfate and filtered. The filtrate was concentrated in vacuo and the residue was dissolved in MeOH (100 mL). The resulting mixture was cooled to 0 °C in an ice / methanol bath. Sodium borohydride (3.93 g, 103.81 mmol, 3.66 mL) was added portionwise, then the reaction mixture was allowed to warm to room temperature and stirred overnight after which it was concentrated in vacuo and water (100 mL) was added. The resulting mixture was extracted with DCM (2 × 100 mL) and the combined organic layers were dried over anhydrous sodium sulfate and filtered. The filtrate was concentrated in vacuo to obtain 1-(2-chloro-4-(dimethoxymethyl)pyridin-3-yl)ethan-1-ol (4.57 g, 19.72 mmol, 95% yield) as a thick, red oil which was used in the next step without further purification. LCMS (ESI): [M+H]+ m/z: calcd 232.08; found 232.0; Rt = 1.015 min.

4-chloro-3-methyl-1,3-dihydrofuro[3,4-*c*]pyridin-1-ol.
1-(2-chloro-4-(dimethoxymethyl)pyridin-3-yl)ethan-1-ol (4.57 g, 19.72 mmol) was dissolved in 85 % formic acid (40 mL) and the resulting mixture was stirred overnight after which it was concentrated in vacuo. The residue was dissolved in DCM (100 mL) and washed with aq. $NaHCO_3$ solution (2 × 45 mL). The organic layer was dried over anhydrous sodium sulfate and filtered. The filtrate was concentrated in vacuo and the residue was purified by column chromatography (gradient MTBE in hexane from 10 % to 50 %) to obtain 4-chloro-3-methyl-1,3-dihydrofuro[3,4-*c*]pyridin-1-ol (948 mg, 5.11 mmol, 26% yield) as a white solid. LCMS (ESI): [M+H]+ m/z: calcd 186.03; found 186.0; Rt = 0.826 min.

4-chloro-3-methyl-1,3-dihydrofuro[3,4-*c*]pyridine.
4-chloro-3-methyl-1,3-dihydrofuro[3,4-*c*]pyridin-1-ol (630 mg, 3.39 mmol) was suspended in DCM (10 mL) and trifluoroacetic acid (1.94 g, 16.97 mmol, 1.30 mL) was added. The resulting mixture was stirred for 5 min and triethylsilane (1.18 g, 10.18 mmol, 1.63 mL) was added dropwise, then stirred for 3 h. The reaction mixture was poured into aq. $NaHCO_3$ solution (2 g in 20 mL of water) and the resulting mixture was extracted with DCM (3 × 45 mL). The combined organic layers were dried over anhydrous sodium sulfate and filtered. The filtrate was concentrated in vacuo and purified by column chromatography (Hexane-MTBE 3:1) to obtain 4-chloro-3-methyl-1,3-dihydrofuro[3,4-*c*]pyridine (1.15 g, crude). LCMS (ESI): [M+H]+ m/z: calcd 170.04; found 170.0; Rt = 1.059 min.

N-(2,4-dimethoxybenzyl)-3-methyl-1,3-dihydrofuro[3,4-*c*]pyridin-4-amine.
4-chloro-3-methyl-1,3-dihydrofuro[3,4-*c*]pyridine (984 mg, 5.80 mmol), (2,4-dimethoxyphenyl)methanamine (1.26 g, 7.54 mmol), cesium carbonate (3.78 g, 11.60 mmol), 4,5-bis(diphenylphosphino)-9,9-dimethylxanthene (503.54 mg, 870.24 µmol) and tris(dibenzylideneacetone)dipalladium(0) (398.45 mg, 435.12 µmol) were stirred in dioxane (20 mL). The resulting mixture was evacuated and backfilled three times with Ar and heated at 110 °C overnight, then cooled and filtered. The filtrate was concentrated in vacuo and the residue was purified by column chromatography (hexane-MTBE 3:1) to obtain *N*-(2,4-dimethoxybenzyl)-3-methyl-1,3-dihydrofuro[3,4-*c*]pyridin-4-amine (472 mg, 1.57 mmol, 27% yield) as a reddish gum. LCMS (ESI): $[M+H]^+$ m/z: calcd 301.16; found 301.2; Rt = 0.728 min.

3-methyl-1,3-dihydrofuro[3,4-*c*]pyridin-4-amine.
N-(2,4-dimethoxybenzyl)-3-methyl-1,3-dihydrofuro[3,4-*c*]pyridin-4-amine (430 mg, 1.43 mmol) was dissolved in TFA (8.6 mL) and the resulting mixture was stirred for 2 h, then concentrated in vacuo to obtain 3-methyl-1,3-dihydrofuro[3,4-*c*]pyridin-4-amine (300 mg, crude) as a light-brown solid. LCMS (ESI): [M+H]+ m/z: calcd 151.09; found 151.0; Rt = 0.418 min.

7-bromo-3-methyl-1,3-dihydrofuro[3,4-*c*]pyridin-4-amine, **Intermediate 18a**.
3-methyl-1,3-dihydrofuro[3,4-*c*]pyridin-4-amine (200 mg, 1.33 mmol) was dissolved in DMF (4 mL) and the resulting mixture was cooled to 0 °C in an ice bath. A solution of *N*-bromosuccinimide (237.03 mg, 1.33 mmol) in DMF (4 mL) was slowly added dropwise at 0 °C. After the addition was completed, the reaction mixture was stirred at 0 °C for 1 h, then diluted with EtOAc (50 mL) and the resulting mixture was washed with water (2 × 25 mL), and brine (2 × 30 mL), dried over anhydrous sodium sulfate, and filtered. The filtrate was concentrated in vacuo and the residue was purified by column chromatography (gradient ACN in $CHCl_3$ from 10 % to 50 %) to obtain 7-bromo-3-methyl-1,3-dihydrofuro[3,4-*c*]pyridin-4-amine, **Intermediate 18a** (40.3 mg, 175.93 µmol, 13% yield) as a red gum. LCMS (ESI): [M+H]+ m/z: calcd 229.0; found 229.0; Rt = 0.606 min.

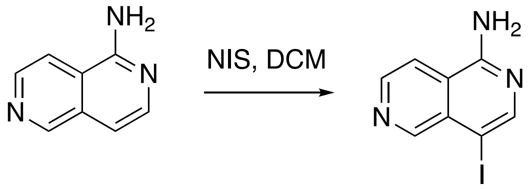

Intermediate 20a

4-iodo-2,6-naphthyridin-1-amine, **Intermediate 20a**.
NIS (1.01 g, 4.48 mmol) was added in one portion to a stirred mixture of 2,6-naphthyridin-1-amine (0.5 g, 3.44 mmol) in DCM (20 mL) and stirred at 25 °C for 8 h. Half of starting solvent was evaporated, the resulting suspension was filtered, washed with water (2 x 10 mL) and TBME (10 mL), air dried at 50 °C to give pure 4-iodo-2,6-naphthyridin-1-amine, **Intermediate 20a** (0.9 g, 3.32 mmol, 96% yield). LCMS (ESI): [M]+ m/z: calcd 271.2; found 272.2; Rt = 0.722 min.

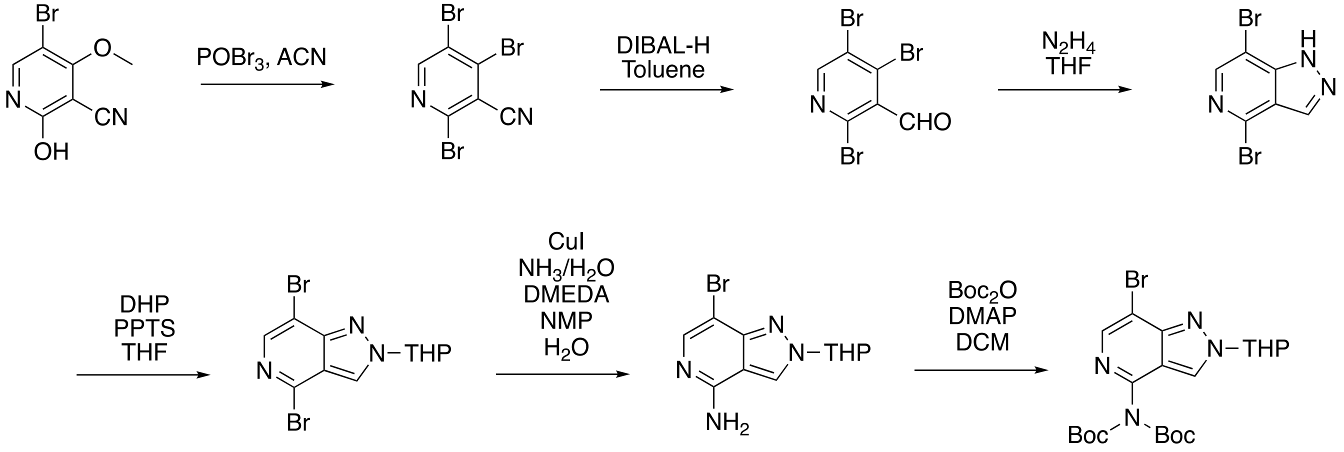

Intermediate 36a

2,4,5-tribromonicotinonitrile.
To a solution of 5-bromo-2-hydroxy-4-methoxynicotinonitrile 1 (9.00 kg, 39.3 mol, 1.00 eq) in ACN (63 L) was added $POBr_3$ (22.53 kg, 78.6 mol, 7.98 L, 2.00 eq). The mixture was stirred at 80 °C for 12 h. The reaction mixture was poured to H_2O (20.0 L) at 0 - 10 °C and filtered. The filter cake was dissolved with ethyl acetate (10 V), adjusted the pH to 8 - 9 by adding saturated K_2CO_3 aqueous solution, then extracted with EtOAc (5.00 L x 3). The combined organic layers were washed with brine (5 L x 2), dried over Na_2SO_4, filtered, and concentrated under reduced pressure to give a residue. The crude product was not purified and was directly used in the next step. 2,4,5-tribromonicotinonitrile (8.00 kg, crude) was obtained as off white solid.

2,4,5-tribromonicotinaldehyde.
To a solution of 2,4,5-tribromonicotinonitrile (2.00 kg, 5.87 mol, 1.00 eq) in toluene (14.0 L) was added DIBAL-H (1 M, 7.63 L, 1.30 eq) at -55 °C. The mixture was stirred at -40 ~ -50 °C for 2 h. Four reactions were combined for work-up. The reaction mixture was poured into MeOH (4 L) at 0 - 5 °C and adjusted pH to 5 - 6 by 1 M HCl (3 L) at 0 - 10 °C. The mixture was extracted with EtOAc (12 L x 2) and washed with brine (4.00 L x 2), dried over Na_2SO_4, filltered, and concentrated under reduced pressure at 40 - 45 °C to give a residue. The crude product was not purified and was directly used in the next step. 2,4,5-tribromonicotinaldehyde (9.05 kg, crude) was obtained as yellow solid. ^{1}H NMR (400 MHz, DMSO-d_6) δ 10.03 (s, 1 H), 10.06 - 10.00 (m, 1 H).

4,7-dibromo-1*H*-pyrazolo[4,3-*c*]pyridine.
To a solution of 2,4,5-tribromonicotinaldehyde (3.00 kg, 8.73 mol, 1.00 eq) in THF (21 L) was dropwised N_2H_4 (891 g, 17.4 mol, 865 mL, 98% purity, 2.00 eq) at 25 -30 °C for 1 h. The mixture was stirred at 60 °C for 3h. Three reactions were combined for work-up. The reaction mixture was poured into H_2O (9 L) at 0 - 5 °C, and added 1 M HCl (1.20 L) adjusted the mixture to pH = 4 - 5 at 0-10 °C, and extracted the mixture with EtOAc (9 L x 3), and washed the mixture with brine (6 L x 3), dried over Na_2SO_4, filtered, and concentrated under reduced pressure at 40 - 45 °C to give a residue. The crude product was not purified and was directly used in the next step. 4,7-dibromo-1*H*-pyrazolo[4,3-*c*]pyridine (6.30 kg, crude) was obtained as yellow solid.

4,7-dibromo-2-(tetrahydro-2*H*-pyran-2-yl)-2*H*-pyrazolo[4,3-*c*]pyridine.
To a solution of 4,7-dibromo-1*H*-pyrazolo[4,3-*c*]pyridine (2.10 kg, 5.31 mol, 70%, 1.00 eq) in THF (14.7 L) was added DHP (2.23 kg, 26.5 mol, 2.43 L, 5.00 eq) and PPTS (133 g, 530 mmol, 0.10 eq) at 25 °C. The mixture was stirred at 60 °C for 12 h. Three reactions were combined for work-up. The reaction mixture was quenched by saturated $NaHCO_3$ (14 L) at 0 - 20 °C and extracted with EtOAc (5 L x 3). The combined organic layers were washed with brine (5 L), dried over Na_2SO_4, filtered, and concentrated under reduced pressure to give a residue. The residue was purified by column chromatography (SiO_2, petroleum ether/ethyl acetate = 50 / 1 to 0 / 1). 4,7-dibromo-2-(tetrahydro-2*H*-pyran-2-yl)-2*H*-pyrazolo[4,3-*c*]pyridine (1.55 kg, 3.86 mol, 24% yield, 90.0% purity) was obtained as yellow solid. ^{1}H NMR (400 MHz, DMSO-d_6) δ 8.99 (s, 1 H), 8.23 (s, 1 H), 5.87 (d, 1 H), 4.02 (d, 1 H), 3.75 (d, 1 H), 2.20 (s, 1 H), 2.11 (d, 1 H), 1.95 (d, 1 H), 1.79 - 1.55 (m, 3 H).

7-bromo-2-(tetrahydro-2*H*-pyran-2-yl)-2*H*-pyrazolo[4,3-*c*]pyridin-4-amine.

To a solution of 4,7-dibromo-1*H*-pyrazolo[4,3-*c*]pyridine (158 g, 439 mmol, 1.00 eq) in NMP (632 mL) and H_2O (1.11 L) was added NH_3/H_2O (739 g, 5.27 mol, 813 mL, 25.0% purity, 12.0 eq), CuI (8.37 g, 43.9 mmol, 0.10 eq) and DMEDA (7.75 g, 87.9 mmol, 9.46 mL, 0.20 eq). The mixture was stirred at 90 °C for 8 h. The reaction mixture was filtered and extracted the filtrate with EtOAc (600 mL x 2) and washed the mixture with brine (300 mL x 2), dried over Na_2SO_4, filtered, and concentrated under reduced pressure at 40 - 45 °C to give a residue. The crude product was not purified and was directly used in the next step. 7-bromo-2-(tetrahydro-2*H*-pyran-2-yl)-2*H*-pyrazolo[4,3-*c*]pyridin-4-amine (116 g, crude) was obtained as brown solid. ^{1}H NMR (400 MHz, DMSO) δ 8.52 (s, 1 H), 7.43 (s, 1 H), 6.78 - 6.80 (m, 1 H), 6.74 (s, 1 H), 5.52 (d, 1 H), 3.87 - 3.76 (m, 2 H), 3.60 - 3.50 (m, 1 H), 3.60 - 3.47 (m, 1 H), 1.90 (d, 1 H), 1.79 (s, 1 H), 1.45 - 1.33 (m, 3 H).

tert-butyl (7-bromo-2-(tetrahydro-2*H*-pyran-2-yl)-2*H*-pyrazolo[4,3-*c*]pyridin-4-yl)(*tert*-butoxycarbonyl)carbamate, **Intermediate 36a**.
To a solution of 7-bromo-2-(tetrahydro-2*H*-pyran-2-yl)-2*H*-pyrazolo[4,3-*c*]pyridin-4-amine (271 g, 912 mmol, 1.00 eq) in DCM (1.90 L) was added Boc_2O (497 g, 2.28 mol, 523 mL, 2.50 eq) and DMAP (22.3 g, 182 mmol, 0.20 eq). The mixture was stirred at 25 °C for 12 h. The reaction mixture was washed with citric acid aq (2.5 L x 2), and washed with NH_4Cl aq (2.5 L x 2), filtered, and concentrated under reduced pressure to give a residue. The crude product was triturated with EtOH (300 mL) at - 20 °C for 2 h. *tert*-butyl (7-bromo-2-(tetrahydro-2*H*-pyran-2-yl)-2*H*-pyrazolo[4,3-*c*]pyridin-4-yl)(*tert*-butoxycarbonyl)carbamate, **Intermediate 36a** (410 g, 812 mmol, 45% yield, 98.6% purity was obtained as an off-white solid. ^{1}H NMR (400 MHz, DMSO-d_6) δ 8.96 (s, 1 H), 8.29 (s, 1 H), 5.88 (d, 1 H), 3.99 (s, 1 H), 3.66 - 3.80 (m, 1 H), 2.27 - 2.16 (m, 1 H), 2.09 (s, 1 H), 1.92 - 1.99 (m, 1 H), 1.68 - 1.80 (m, 1 H), 1.54 - 1.65 (m, 2 H), 1.36 (s, 18 H). LCMS: Rt = 2.988 min, M+1=497.

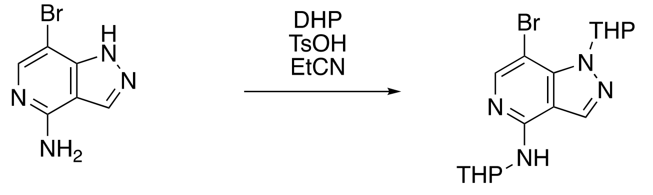

74 **Intermediate 37a**

7-bromo-*N*,1-bis(tetrahydro-2*H*-pyran-2-yl)-1*H*-pyrazolo[4,3-*c*]pyridin-4-amine, **Intermediate 37a**.
74 (0.2 g, 938.82 µmol), 3,4-dihydro-2H-pyran (0.5 g, 5.94 mmol, 539.96 µL), and p-toluenesulfonic acid monohydrate (0.05 g, 262.85 µmol, 40.32 µL) were stirred in CH_3CH_2CN (5 mL) and the resulting solution was stirred in an autoclave overnight at 100 °C, after which the reaction was concentrated and the desired product 7-bromo-*N*,1-bis(tetrahydro-2*H*-pyran-2-yl)-1*H*-pyrazolo[4,3-*c*]pyridin-4-amine, **Intermediate 37a** (0.35 g, 917.99 µmol, 98% yield) was isolated as with 29 % purity and used in the next step as is. LCMS(ESI): [M]+ m/z: calcd 297.15; found 297.0; Rt = 0.663 min.

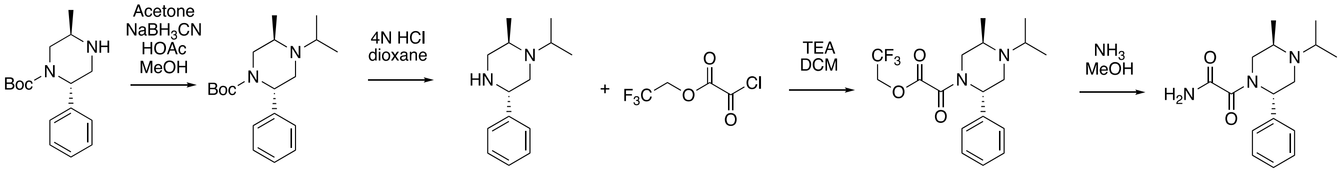

Intermediate 34a

tert-butyl (2*S*,5*R*)-4-isopropyl-5-methyl-2-phenylpiperazine-1-carboxylate.
Acetone (945.66 mg, 16.28 mmol, 1.20 mL) and acetic acid (391.10 mg, 6.51 mmol, 372.83 µL) were added to a stirred solution of *tert*-butyl (2*S*,5*R*)-5-methyl-2-phenylpiperazine-1-carboxylate (0.9 g, 3.26 mmol) in MeOH (30 mL) at 25 °C. The resulting mixture was stirred at 25 °C for 0.5 h, then sodium cyanoborohydride (409.27 mg, 6.51 mmol) was added in one portion at 25 °C. The reaction mixture was stirred at 25 °C for 48 h, then concentrated in vacuo. The residue was diluted with 10% aqueous sodium hydroxide solution (20 mL) and extracted with dichloromethane (2 x 20 mL). The combined organic extracts were dried over sodium sulphate and concentrated in vacuo to afford *tert*-butyl (2*S*,5*R*)-4-isopropyl-5-methyl-2-phenylpiperazine-1-carboxylate (0.7 g, crude). LCMS (ESI): [M+H]+ m/z: calcd 319.2; found 319.2; Rt = 1.050 min.

(2*R*,5*S*)-1-isopropyl-2-methyl-5-phenylpiperazine.
tert-butyl (2*S*,5*R*)-4-isopropyl-5-methyl-2-phenylpiperazine-1-carboxylate (0.7 g, 2.20 mmol) was dissolved in dioxane / HCl (20 mL), the resulting mixture was left at 25 °C for 17 h. Upon completion, the solvent was concentrated to dryness, water was added to the residue (20 mL) and it was basified with K_2CO_3 to alkaline pH, the aqueous phase was extracted with DCM (3 x 15 mL), and the combined organic layers were dried over Na_2SO_4, filtered, and concentrated in vacuo to obtain (2*R*,5*S*)-1-isopropyl-2-methyl-5-phenylpiperazine (0.4 g, crude). LCMS (ESI): [M+H]+ m/z: calcd 219.2; found 219.2; Rt = 0.382 min.

2,2,2-trifluoroethyl 2-((2*S*,5*R*)-4-isopropyl-5-methyl-2-phenylpiperazin-1-yl)-2-oxoacetate.
(2*R*,5*S*)-1-isopropyl-2-methyl-5-phenylpiperazine (0.4 g, 1.83 mmol) and TEA (278.07 mg, 2.75 mmol, 383.02 µL) were stirred in DCM (20 mL) and the resulting solution was cooled to 5 °C in an ice bath. 2,2,2-Trifluoroethyl 2-chloro-2-oxo-acetate (383.91 mg, 2.02 mmol) was added dropwise and the resulting mixture was allowed to warm to room temperature and stirred for 18 h. The solution was washed with brine (2 x 20 mL), the organic layer was dried over Na_2SO_4, filtered, and concentrated in vacuo to afford 2,2,2-trifluoroethyl 2-((2*S*,5*R*)-4-isopropyl-5-methyl-2-phenylpiperazin-1-yl)-2-oxoacetate (0.55 g, crude). LCMS (ESI): [M+H]+ m/z: calcd 373.2; found 373.2; Rt = 0.997 min.

2-((2*S*,5*R*)-4-isopropyl-5-methyl-2-phenylpiperazin-1-yl)-2-oxoacetamide, **Intermediate 34a**.
2,2,2-trifluoroethyl 2-((2*S*,5*R*)-4-isopropyl-5-methyl-2-phenylpiperazin-1-yl)-2-oxoacetate (0.55 g, 1.48 mmol) was dissolved in NH_3 / MeOH (25 mL). The resulting clear solution was left for 16 h at 25 °C. The solution was concentrated to dryness to obtain crude product which was purified by HPLC (column: XBridge BEH C18 100 x 19 mm, 5 μm; mobile phase: 30 – 70 % 0-5 min H_2O / MeCN / 0.1 % NH_4OH, flow rate: 30 mL / min (loading pump 4 mL / min MeOH) to afford 2-((2*S*,5*R*)-4-isopropyl-5-methyl-2-phenylpiperazin-1-yl)-2-oxoacetamide, **Intermediate 34a**. LCMS (ESI): [M+H]+ m/z: calcd 219.2; found 219.2; Rt = 0.513 min.

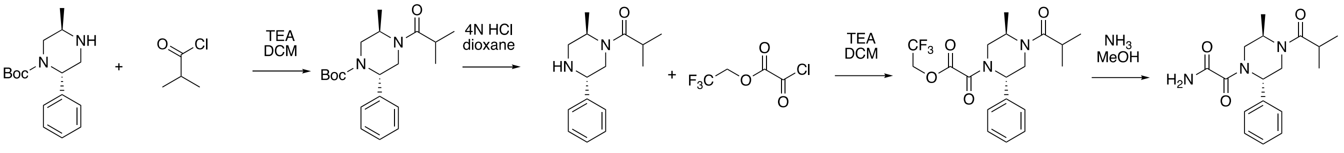

Intermediate 35a

tert-butyl (2*S*,5*R*)-4-isobutyryl-5-methyl-2-phenylpiperazine-1-carboxylate.
isobutyryl chloride (1.3 eq) was added dropwise to a solution of *tert*-butyl (2*S*,5*R*)-5-methyl-2-phenylpiperazine-1-carboxylate (1 eq) and TEA (3 eq) in DCM. After addition was complete, resulting mixture was stirred at 20 °C for 3 h after which 15% aq. K_2CO_3 solution was added and stirring was continued for 10 min. The organic layer was then separated, dried over Na_2SO_4, filtered, and evaporated to obtain *tert*-butyl (2*S*,5*R*)-4-isobutyryl-5-methyl-2-phenylpiperazine-1-carboxylate Yield: 94 %. LCMS (ESI): [M]+ m/z: calcd 346.2; found 347.2; Rt = 1.559 min.

2-methyl-1-((2*R*,5*S*)-2-methyl-5-phenylpiperazin-1-yl)propan-1-one.
tert-butyl (2*S*,5*R*)-5-methyl-4-(1-methylcyclopropane-1-carbonyl)-2-phenylpiperazine-1-carboxylate (1 eq) was dissolved in DCM and TFA (7 eq) was added. The resulting mixture was stirred at 25 °C for 15 h, then evaporated in vacuum to afford 2-methyl-1-((2*R*,5*S*)-2-methyl-5-phenylpiperazin-1-yl)propan-1-one, Yield: 98 %. LCMS (ESI): [M]+ m/z: calcd 246.2; found 247.2; Rt = 0.821 min.

2,2,2-trifluoroethyl 2-((2*S*,5*R*)-4-isobutyryl-5-methyl-2-phenylpiperazin-1-yl)-2-oxoacetate.
2-methyl-1-((2*R*,5*S*)-2-methyl-5-phenylpiperazin-1-yl)propan-1-one and TEA (1.1 eq) were dissolved in DCM and cooled to 0 °C, followed by the dropwise addition of 2,2,2-trifluoroethyl 2-chloro-2-oxo-acetate (1.1 eq). The reaction mixture was stirred for 12 h at room temperature. The mixture was diluted with DCM and washed with water and brine. The organic layer was dried over Na_2SO_4 and evaporated under reduced pressure to give 2,2,2-trifluoroethyl 2-((2*S*,5*R*)-4-isobutyryl-5-methyl-2-phenylpiperazin-1-yl)-2-oxoacetatewas used in the next step without further purification. LCMS (ESI): [M]+ m/z: calcd 400.2; found 401.2; Rt = 1.351 min.

2-((2*S*,5*R*)-4-isobutyryl-5-methyl-2-phenylpiperazin-1-yl)-2-oxoacetamide, **Intermediate 35b**.
2,2,2-trifluoroethyl 2-((2*S*,5*R*)-4-isobutyryl-5-methyl-2-phenylpiperazin-1-yl)-2-oxoacetate oxoacetate (1 eq) was dissolved in NH_3 / MeOH. The reaction mixture was stirred overnight and then evaporated to dryness to give 2-((2*S*,5*R*)-4-isobutyryl-5-methyl-2-phenylpiperazin-1-yl)-2-oxoacetamide, **Intermediate 35b** which was used in the next step without further purification. LCMS (ESI): [M]+ m/z: calcd 317.2; found 318.2; Rt = 0.811 min.

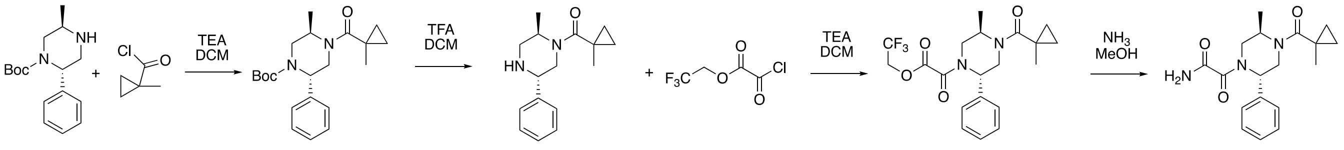

Intermediate 36b

tert-butyl (2*S*,5*R*)-5-methyl-4-(1-methylcyclopropane-1-carbonyl)-2-phenylpiperazine-1-carboxylate.
1-methylcyclopropane-1-carbonyl chloride (1.3 eq) was added dropwise to a solution of *tert*-butyl (2*S*,5*R*)-5-methyl-2-phenylpiperazine-1-carboxylate (1 eq) and TEA (3 eq) in DCM. After addition was complete, resulting mixture was stirred at 20 °C for 3 h after which 15% aq. K_2CO_3 solution was added and stirring was continued for 10 min. The organic layer was then separated, dried over Na_2SO_4, filtered, and evaporated to obtain *tert*-butyl (2*S*,5*R*)-5-methyl-4-(1-methylcyclopropane-1-carbonyl)-2-phenylpiperazine-1-carboxylate, Yield: 99 %. LCMS (ESI): [M]+ m/z: calcd 358.2; found 359.2; Rt = 1.312 min.

((2*R*,5*S*)-2-methyl-5-phenylpiperazin-1-yl)(1-methylcyclopropyl)methanone.
tert-butyl (2*S*,5*R*)-5-methyl-4-(1-methylcyclopropane-1-carbonyl)-2-phenylpiperazine-1-carboxylate (1 eq) was dissolved in DCM and TFA (7 eq) was added. The resulting mixture was stirred at 25 °C for 15 h, then evaporated in vacuum to afford ((2*R*,5*S*)-2-methyl-5-phenylpiperazin-1-yl)(1-methylcyclopropyl)methanone (TFA-salt), Yield: 92 %. LCMS (ESI): [M]+ m/z: calcd 258.2; found 259.2; Rt = 0.820 min.

2,2,2-trifluoroethyl 2-((2*S*,5*R*)-5-methyl-4-(1-methylcyclopropane-1-carbonyl)-2-phenylpiperazin-1-yl)-2-oxoacetate.
((2*R*,5*S*)-2-methyl-5-phenylpiperazin-1-yl)(1-methylcyclopropyl)methanone and TEA (1.1 eq) were dissolved in DCM and cooled to 0 °C, followed by the dropwise addition of 2,2,2-trifluoroethyl 2-chloro-2-oxo-acetate (1.1 eq). The reaction mixture was stirred for 12 h at room temperature. The mixture was diluted with DCM and washed with water and brine. The organic layer was dried over Na_2SO_4 and evaporated under reduced pressure to give 2,2,2-trifluoroethyl 2-((2*S*,5*R*)-5-methyl-4-(1-methylcyclopropane-1-carbonyl)-2-phenylpiperazin-1-yl)-2-oxoacetate which was used in the next step without further purification, yield: 98 %. LCMS (ESI): [M]+ m/z: calcd 412.2; found 413.2; Rt = 1.223 min.

2-((2*S*,5*R*)-5-methyl-4-(1-methylcyclopropane-1-carbonyl)-2-phenylpiperazin-1-yl)-2-oxoacetamide, **Intermediate 36b**.
2,2,2-trifluoroethyl 2-((2*S*,5*R*)-5-methyl-4-(1-methylcyclopropane-1-carbonyl)-2-phenylpiperazin-1-yl)-2-oxoacetate (1 eq) was dissolved in NH_3 / MeOH. The reaction mixture was stirred overnight and then evaporated to dryness to give 2-((2*S*,5*R*)-5-methyl-4-(1-methylcyclopropane-1-carbonyl)-2-

phenylpiperazin-1-yl)-2-oxoacetamide, **Intermediate 36b** which was used in the next step without further purification, yield: 99 %. LCMS (ESI): [M]+ m/z: calcd 329.2; found 330.2; Rt = 1.025 min.

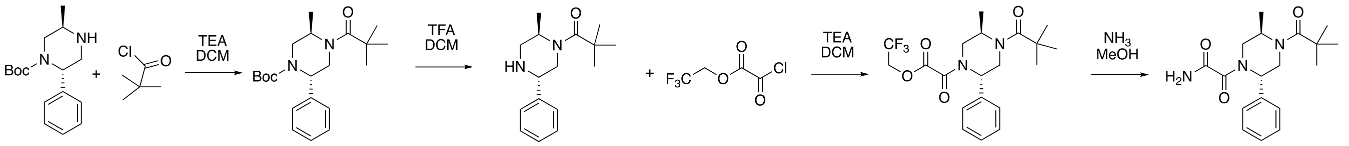

Intermediate 37b

tert-butyl (2*S*,5*R*)-5-methyl-2-phenyl-4-pivaloylpiperazine-1-carboxylate.
Pivaloyl chloride (1.3 eq) was added dropwise to a solution of *tert*-butyl (2*S*,5*R*)-5-methyl-2-phenylpiperazine-1-carboxylate (1 eq) and TEA (3 eq) in DCM. After addition was complete, the resulting mixture was stirred at 20 °C for 3 h, after which 15% aq. K_2CO_3 solution was added and stirring was continued for 10 min. After that, organic layer was separated, dried over Na2SO4, filtered, and evaporated to obtain *tert*-butyl (2*S*,5*R*)-5-methyl-2-phenyl-4-pivaloylpiperazine-1-carboxylate, Yield: 100 %. LCMS (ESI): [M-t-Bu]+ m/z: calcd 304.2; found 305.2; Rt = 1.597 min.

2,2-dimethyl-1-((2*R*,5*S*)-2-methyl-5-phenylpiperazin-1-yl)propan-1-one.
tert-butyl (2*S*,5*R*)-5-methyl-2-phenyl-4-pivaloylpiperazine-1-carboxylate (1 eq) was dissolved in DCM and TFA (7 eq) was added. The resulting mixture was stirred at 25 °C for 15 h, hen evaporated in vacuum to afford 2,2-dimethyl-1-((2*R*,5*S*)-2-methyl-5-phenylpiperazin-1-yl)propan-1-one (TFA-salt), Yield: 98 %. LCMS (ESI): [M]+ m/z: calcd 260.2; found 261.2; Rt = 0.910 min.

2,2,2-trifluoroethyl 2-((2*S*,5*R*)-5-methyl-2-phenyl-4-pivaloylpiperazin-1-yl)-2-oxoacetate.
2,2-dimethyl-1-((2*R*,5*S*)-2-methyl-5-phenylpiperazin-1-yl)propan-1-one and TEA (1.1 eq) were dissolved in DCM and cooled to 0 °C , followed by the dropwise addition of 2,2,2-trifluoroethyl 2-chloro-2-oxo-acetate (1.1 eq). The reaction mixture was stirred for 12 h at room temperature. The mixture was diluted with DCM and washed with water and brine. The organic layer was dried over Na_2SO_4 and evaporated under reduced pressure to give 2,2,2-trifluoroethyl 2-((2*S*,5*R*)-5-methyl-2-phenyl-4-pivaloylpiperazin-1-yl)-2-oxoacetate which was used in the next step without further purification, yield: 94 %. LCMS (ESI): [M]+ m/z: calcd 414.2; found 415.2; Rt = 1.451 min.

2-((2*S*,5*R*)-5-methyl-2-phenyl-4-pivaloylpiperazin-1-yl)-2-oxoacetamide, **Intermediate 37b**.
2,2,2-trifluoroethyl 2-((2*S*,5*R*)-5-methyl-2-phenyl-4-pivaloylpiperazin-1-yl)-2-oxoacetate (1 eq) was dissolved in NH_3 / MeOH. The reaction mixture was stirred overnight and then evaporated to dryness to give 2-((2*S*,5*R*)-5-methyl-2-phenyl-4-pivaloylpiperazin-1-yl)-2-oxoacetamide, **Intermediate 37b** which was used in the next step without further purification, yield: 97 %. LCMS (ESI): [M]+ m/z: calcd 331.2; found 332.2; Rt = 1.136 min.

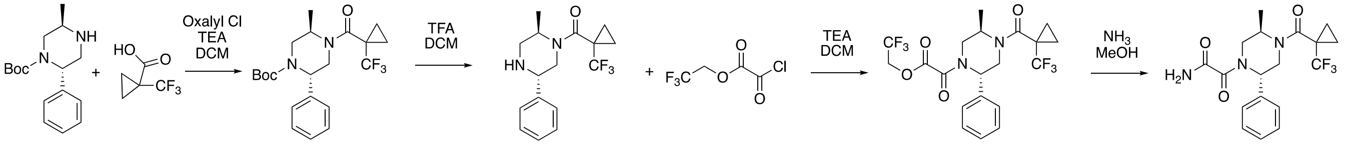

Intermediate 46a

Intermediate 38b

tert-butyl (2*S*,5*R*)-5-methyl-2-phenyl-4-(1-(trifluoromethyl)cyclopropane-1-carbonyl)piperazine-1-carboxylate.
1-(Trifluoromethyl)cyclopropanecarboxylic acid (0.2 g, 1.30 mmol) and few drops of DMF were stirred in $CHCl_3$ (6 mL) and the resulting solution was cooled to 5 °C in an ice bath . Oxalyl chloride (192.89 mg, 1.52 mmol, 132.11 uL) was added dropwise and the resulting mixture was allowed to warm to room temperature and stirred for 2 h. After completion the reaction mixture was poured into aq. K_2CO_3 solution (4 g in 10 mL of water) and the resulting mixture was extracted with $CHCl_3$ (2 x 10 mL). The combined organic layers were dried over Na_2SO_4 and added dropwise at 0 °C to a stirred solution of *tert*-butyl (2*S*,5*R*)-5-methyl-2-phenylpiperazine-1-carboxylate (0.28 g, 1.01 mmol) and TEA (153.78 mg, 1.52 mmol, 211.81 μL) in $CHCl_3$ (6 mL).The resulting mixture was allowed to warm to room temperature and stirred overnight. The reaction mixture was diluted with $CHCl_3$ (5mL) and the resulting solution was washed with water (2 x 10 mL), dried over Na_2SO_4, filtered, and evaporated to afford *tert*-butyl (2*S*,5*R*)-5-methyl-2-phenyl-4-(1-(trifluoromethyl)cyclopropane-1-carbonyl)piperazine-1-carboxylate (0.4 g, 969.83 umol, 96% yield). LCMS (ESI): $[M\text{-}Boc]^+$ m/z: calcd 312.2; found 313.2; Rt = 1.456 min.

((2*R*,5*S*)-2-methyl-5-phenylpiperazin-1-yl)(1-(trifluoromethyl)cyclopropyl)methanone, **Intermediate 46a**.
tert-butyl (2*S*,5*R*)-5-methyl-2-phenyl-4-(1-(trifluoromethyl)cyclopropane-1-carbonyl)piperazine-1-carboxylate (1 eq) was dissolved in DCM and TFA (7 eq) was added. The resulting mixture was stirred at 25 °C for 15 h, hen evaporated in vacuum to afford ((2*R*,5*S*)-2-methyl-5-phenylpiperazin-1-yl)(1-(trifluoromethyl)cyclopropyl)methanone (TFA-salt), Yield: 99 %. LCMS (ESI): $[M]^+$ m/z: calcd 312.2; found 313.2; Rt = 0.922 min.

2,2,2-trifluoroethyl 2-((2*S*,5*R*)-5-methyl-2-phenyl-4-(1-(trifluoromethyl)cyclopropane-1-carbonyl)piperazin-1-yl)-2-oxoacetate.
((2*R*,5*S*)-2-methyl-5-phenylpiperazin-1-yl)(1-(trifluoromethyl)cyclopropyl)methanone and TEA (1.1 eq) were dissolved in DCM and cooled to 0 °C, followed by the dropwise addition of 2,2,2-trifluoroethyl 2-chloro-2-oxo-acetate (1.1 eq). The reaction mixture was stirred for 12 h at room temperature. The mixture was diluted with DCM and washed with water and brine. The organic layer was dried over Na_2SO_4 and evaporated under reduced pressure to give 2,2,2-trifluoroethyl 2-((2*S*,5*R*)-5-methyl-2-phenyl-4-(1-(trifluoromethyl)cyclopropane-1-carbonyl)piperazin-1-yl)-2-oxoacetate which was used in the next step without further purification. LCMS (ESI): [M]+ m/z: calcd 466.2; found 467.2; Rt = 1.368 min.

2-((2*S*,5*R*)-5-methyl-2-phenyl-4-(1-(trifluoromethyl)cyclopropane-1-carbonyl)piperazin-1-yl)-2-oxoacetamide, **Intermediate 38b**.
2,2,2-trifluoroethyl 2-((2*S*,5*R*)-5-methyl-2-phenyl-4-(1-(trifluoromethyl)cyclopropane-1-carbonyl)piperazin-1-yl)-2-oxoacetate (1 eq) was dissolved in NH_3 / MeOH. The reaction mixture was stirred overnight and then evaporated to dryness to give 2-((2*S*,5*R*)-5-methyl-2-phenyl-4-(1-

(trifluoromethyl)cyclopropane-1-carbonyl)piperazin-1-yl)-2-oxoacetamide, **Intermediate 38b** which was used in the next step without further purification. LCMS (ESI): [M]+ m/z: calcd 383.2; found 384.2; Rt = 0.914 min.

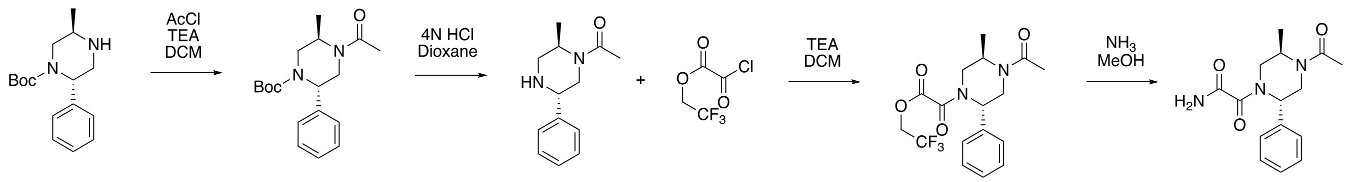

tert-butyl (2*S*,5*R*)-4-acetyl-5-methyl-2-phenylpiperazine-1-carboxylate.
tert-butyl (2*S*,5*R*)-5-methyl-2-phenyl-piperazine-1-carboxylate (1.2 g, 4.34 mmol) and TEA (659.04 mg, 6.51 mmol, 907.77 µL) were stirred in DCM (39.80 mL) and the resulting solution was cooled to 5 °C on an ice bath. Acetyl chloride (374.92 mg, 4.78 mmol, 289.73 µL) was added dropwise and the resulting solution was allowed to warm to room temperature and stirred overnight. The solution was washed with brine (2 x 20 mL) and the organic layer was dried over Na_2SO_4, filtered, and concentrated under vacuum to afford *tert*-butyl (2*S*,5*R*)-4-acetyl-5-methyl-2-phenylpiperazine-1-carboxylate (1 g, crude). LCMS (ESI): [M-Boc]+ m/z: calcd 218.2; found 219.2; Rt = 1.283 min

1-((2*R*,5*S*)-2-methyl-5-phenylpiperazin-1-yl)ethan-1-one.
tert-butyl (2*S*,5*R*)-4-acetyl-5-methyl-2-phenylpiperazine-1-carboxylate (1 g, 3.14 mmol) was dissolved in 4 N HCl/dioxane (20 mL) and the resulting solution was stirred at 25 °C for 17 h. The solvent was removed under reduced pressure, water was added to the residue (20 mL) and then basified with K_2CO_3 to alkaline pH. The aqueous phase was extracted with DCM (3 x 15 mL) and the combined organic layers were dried over Na_2SO_4, filtered, and concentrated under vacuum to obtain 1-((2*R*,5*S*)-2-methyl-5-phenylpiperazin-1-yl)ethan-1-one (0.48 g, crude). LCMS (ESI): [M]+ m/z: calcd 218.2; found 219.2; Rt = 0.453 min.

2,2,2-trifluoroethyl 2-((2*S*,5*R*)-4-acetyl-5-methyl-2-phenylpiperazin-1-yl)-2-oxoacetate.
1-((2*R*,5*S*)-2-methyl-5-phenylpiperazin-1-yl)ethan-1-one and TEA (1.1 eq) were dissolved in DCM and cooled to 0 °C, followed by the dropwise addition of 2,2,2-trifluoroethyl 2-chloro-2-oxo-acetate (1.1 eq). The reaction mixture was stirred for 12 h at room temperature. The mixture was diluted with DCM and washed with water and brine. The organic layer was dried over Na_2SO_4 and evaporated under reduced pressure to give 2,2,2-trifluoroethyl 2-((2*S*,5*R*)-4-acetyl-5-methyl-2-phenylpiperazin-1-yl)-2-oxoacetate which was used in the next step without further purification. LCMS (ESI): [M]+ m/z: calcd 372.2; found 373.2; Rt = 1.223 min.

2-((2*S*,5*R*)-4-acetyl-5-methyl-2-phenylpiperazin-1-yl)-2-oxoacetamide, **Intermediate 42a**.
2,2,2-trifluoroethyl 2-((2*S*,5*R*)-4-acetyl-5-methyl-2-phenylpiperazin-1-yl)-2-oxoacetate (1 eq) was dissolved in NH_3 / MeOH. The reaction mixture was stirred overnight and then evaporated to dryness to 2-((2*S*,5*R*)-4-acetyl-5-methyl-2-phenylpiperazin-1-yl)-2-oxoacetamide, **Intermediate 42a** which was used in the next step without further purification. LCMS (ESI): [M]+ m/z: calcd 289.2; found 290.2; Rt = 0.673 min.

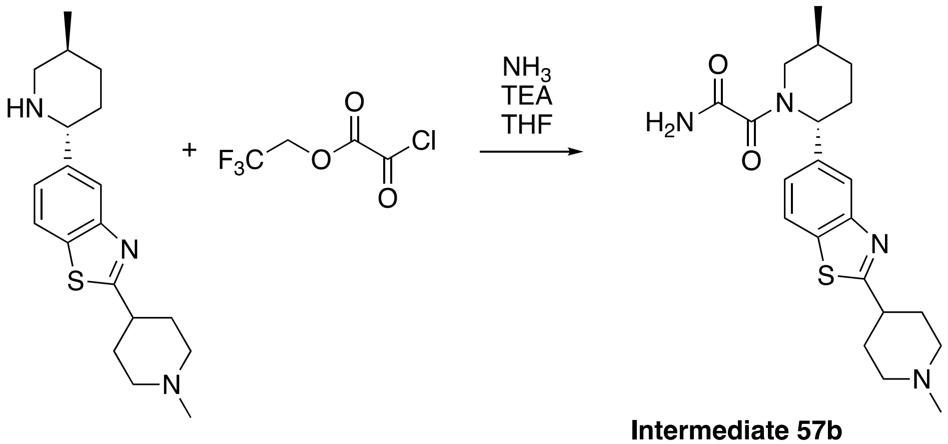

Intermediate 57b

2-((2*R*,5*S*)-5-methyl-2-(2-(1-methylpiperidin-4-yl)benzo[*d*]thiazol-5-yl)piperidin-1-yl)-2-oxoacetamide, **Intermediate 57b**.
2,2,2-Trifluoroethyl 2-chloro-2-oxo-acetate (1.04 g, 5.46 mmol) was slowly added to a stirred solution of **92** (1.5 g, 4.55 mmol) and TEA (921.30 mg, 9.10 mmol, 1.27 mL) in dry THF (50 mL) at 25 °C. The resulting mixture was stirred at 25 °C for 0.5 h, then NH_3 (g) was bubbled through the reaction mixture at 25 °C for 0.5 h. The resulting ammonium chloride precipitate was filtered and discarded, the filtrate was concentrated in vacuum to afford 2-((2*R*,5*S*)-5-methyl-2-(2-(1-methylpiperidin-4-yl)benzo[*d*]thiazol-5-yl)piperidin-1-yl)-2-oxoacetamide, **Intermediate 57b** (1.6 g, 3.99 mmol, 88% yield) as yellow solid, which was used directly in the next step. LCMS (ESI): [M]+ m/z: calcd 400.2; found 401.2; Rt = 0.867 min.

Compounds 4, 9, 11– 20, 34 – 38, 42 – 45, 57, 59 – 61

General Procedure B

Oxamide-NH_2 (1 eq) was added to a mixture of halopyridine (1 eq), (*S,S*)-(+)-*N,N'*-dimethyl-1,2-cyclohexanediamine (1 eq), Cu (0.5 eq), CuI (0.1 eq), and Cs_2CO_3 (2eq) in dioxane (0.18 M). The resulting mixture was stirred at 100 °C for 18 h, then filtered through a pad of Celite and concentrated in vacuo. The residue was purified by HPLC. Where indicated the material was deprotected. Where indicated the material was further purified with chiral SFC or similar.

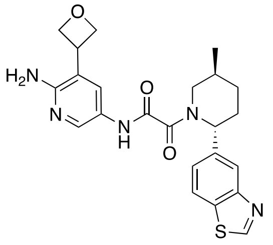

N-(6-amino-5-(oxetan-3-yl)pyridin-3-yl)-2-((2*R*,5*S*)-2-(benzo[*d*]thiazol-5-yl)-5-methylpiperidin-1-yl)-2-oxoacetamide, **Compound 4**.
Oxamide-NH_2: **Intermediate 4b**. Halopyridine: **Intermediate 4a**. HPLC (0.5 - 6.5 min 20 – 0 % water – ACN, +0.1 % vol. of 25 % aq. NH_3, 30 mL / min, column: XBridge, 100 x 19 mm, 5 µm). Chiral HPLC (1st run - Chiralcel OJ-H (250 x 21, 5 µm), Hexane-IPA-MeOH, 50-25-25, 12 mL / min, main peak at 38.717 min; 2nd run - Chiralpak IA II (250 x 21, 5 µm), IPA-MeOH,50-50, 10 mL / min). ^{1}H NMR (600 MHz, DMSO-d_6) δ 0.92 – 1.06 (m, 3H), 1.22 – 1.41 (m, 2H), 1.72 – 1.92 (m, 2H), 2.08 – 2.33 (m, 2H), 2.80 – 2.83 (m, 1H), 3.86 – 4.56 (m, 3H), 4.87 – 4.95 (m, 2H), 5.35 – 5.74 (m, 3H), 7.43 – 7.76 (m, 3H), 8.02 – 8.19 (m, 2H), 9.39 – 9.41 (m, 1H), 10.61 – 10.67 (m, 1H). LCMS (ESI): [M+H]+ m/z: calcd 452.19; found 452.0; Rt = 0.82 min.

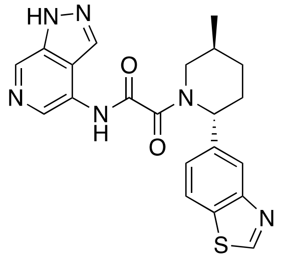

2-((2*R*,5*S*)-2-(benzo[*d*]thiazol-5-yl)-5-methylpiperidin-1-yl)-2-oxo-*N*-(1*H*-pyrazolo[3,4-*c*]pyridin-4-yl)acetamide, **Compound 9**.
Oxamide-NH_2: **Intermediate 4b**. Halopyridine: **Intermediate 9a**. LCMS (ESI): [M]+ m/z: calcd 550.2; found 551.2; Rt = 1.439 min. Deprotection: Trifluoroacetic acid (2.96 g, 25.96 mmol, 2 mL) was added to a solution of 2-[(2*R*,5*S*)-2-(1,3-benzothiazol-5-yl)-5-methyl-1-piperidyl]-2-oxo-*N*-[1-(2-trimethylsilylethoxymethyl)pyrazolo[3,4-c]pyridin-4-yl]acetamide (675 mg, 1.23 mmol) in DCM (10 mL). The resulting mixture was stirred at 25 °C for 15 h after which the volatiles were removed under reduced pressure and the residue was purified by HPLC (40-40-90 % 0-1-6 min H_2O / MeOH /0.1 % NH_4OH, flow: 30 mL / min; column: YMC Triart C18, 100 x 20 mm, 5 um) to afford 2-((2*R*,5*S*)-2-(benzo[*d*]thiazol-5-yl)-5-methylpiperidin-1-yl)-2-oxo-*N*-(1*H*-pyrazolo[3,4-*c*]pyridin-4-yl)acetamide, **Compound 9**. ^{1}H NMR (600 MHz, DMSO-d_6) δ 1.00 – 1.11 (m, 3H), 1.31 – 1.47 (m, 1H), 1.66 – 1.78 (m, 1H), 1.83 – 2.01 (m, 1H), 2.12 – 2.29 (m, 1H), 2.88 – 2.93 (m, 0.3H), 3.37 – 3.39 (m, 0.7H), 3.46 – 4.14 (m, 1H), 5.24 – 5.92 (m, 1H), 7.45 – 7.58 (m, 1H), 7.94 – 8.07 (m, 1H), 8.07 – 8.17 (m, 1H), 8.17 – 8.23 (m, 1H), 8.23 – 8.41 (m, 1H), 8.50 – 8.68 (m, 1H), 8.68 – 8.89 (m, 1H), 9.35 – 9.46 (m, 1H), 11.40 (br s, 1H), 13.59 (br s, 1H). LCMS (ESI): [M]+ m/z: calcd 420.2; found 421.2; Rt = 2.084 min.

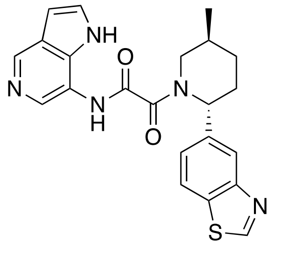

2-((2*R*,5*S*)-2-(benzo[*d*]thiazol-5-yl)-5-methylpiperidin-1-yl)-2-oxo-*N*-(1*H*-pyrrolo[3,2-*c*]pyridin-7-yl)acetamide, **Compound 11**.
Oxamide-NH_2: **Intermediate 4b**. Halopyridine: 7-bromo-1*H*-indole. HPLC (0 - 5 min 25 – 55 % water – ACN, +0.1 % vol. of 25 % aq. NH_3, 30 mL / min, column: XBridge C18, 100 x 20 mm, 5 µm) to afford 2-((2*R*,5*S*)-2-(benzo[*d*]thiazol-5-yl)-5-methylpiperidin-1-yl)-2-oxo-*N*-(1*H*-pyrrolo[3,2-*c*]pyridin-7-yl)acetamide, **Compound 11** (2% yield). ^{1}H NMR (600 MHz, DMSO-d_6) δ 0.99 – 1.14 (m, 3H), 1.32 – 1.53 (m, 1H), 1.71 – 1.79 (m, 1H), 1.85 – 2.00 (m, 1H), 2.07 – 2.36 (m, 1H), 2.63 – 2.76 (m, 1H), 2.81 – 3.21 (m, 1H), 3.49 – 4.17 (m, 1H), 5.42 – 5.88 (m, 1H), 6.52 – 6.67 (m, 1H), 7.36 – 7.60 (m, 2H), 8.01 – 8.11 (m, 1H), 8.13 – 8.23 (m, 1H), 8.25 – 8.51 (m, 1H), 8.52 – 8.75 (m, 1H), 9.37 – 9.45 (m, 1H). LCMS (ESI): [M+H]+ m/z: calcd 420.16; found 420.2; Rt = 2.215 min.

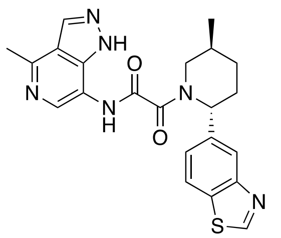

2-((2*R*,5*S*)-2-(benzo[*d*]thiazol-5-yl)-5-methylpiperidin-1-yl)-*N*-(4-methyl-1*H*-pyrazolo[4,3-*c*]pyridin-7-yl)-2-oxoacetamide, **Compound 12**.
Oxamide-NH_2: **Intermediate 4b**. Halopyridine: **Intermediate 12a**. HPLC (0-1-6 min 40-40-80 % water – methanol, +0.1 % vol. of 25 % aq. NH_3, 30 mL /min, column: XBridge BEH C18, 100 x 20 mm, 5 µm) to afford 2-[(2*R*,5*S*)-2-(1,3-benzothiazol-5-yl)-5-methyl-1-piperidyl]-*N*-(4-methyl-1-tetrahydropyran-2-yl-pyrazolo[4,3-c]pyridin-7-yl)-2-oxo-acetamide (27% yield) as a brown solid. LCMS (ESI): [M+H]+ m/z: calcd 519.24; found 519.0; Rt = 2.359 min.
Deprotection: Material dissoved in a mixture of MeOH and Dioxane/HCl, 1:2. The resulting mixture was stirred for 90 min then concentrated in vacuo to dryness (69% yield, HCl salt).
Chiral chromatography to remove residual isomers (Chiralpak IC-III (250 x 20 mm, 5 µm), Hexane-IPA-MeOH, 50-25-25, 12 mL / min) to afford 2-((2*R*,5*S*)-2-(benzo[*d*]thiazol-5-yl)-5-methylpiperidin-1-yl)-*N*-(4-methyl-1*H*-pyrazolo[4,3-*c*]pyridin-7-yl)-2-oxoacetamide, **Compound 12** (HCl salt) as a yellow solid. Analytical RT (Chiralpak IC (250 x 4.6, 5 µm), IPA-MeOH, 50-50, 0.6 mL / min) = 20.0972 min. ^{1}H NMR (500 MHz, DMSO-d_6) δ 1.04 – 1.14 (m, 3H), 1.17 – 1.27 (m, 1H), 1.33 – 1.56 (m, 2H), 1.64 – 1.72 (m, 1H), 1.83 – 1.93 (m, 1H), 2.12 – 2.20 (m, 1H), 2.97 – 3.06 (m, 3H), 3.30 – 3.36 (m, 1H), 3.99 – 4.12 (m, 1H), 7.46 – 7.58 (m, 1H), 8.01 – 8.10 (m, 1H), 8.12 – 8.24 (m, 1H), 8.65 – 8.87 (m, 1H), 8.91 – 9.14 (m, 1H), 9.37 – 9.46 (m, 1H), 11.72 (br s, 1H), 14.45 (br s, 1H). LCMS (ESI): [M+H]+ m/z: calcd 435.17; found 435.2; Rt = 2.281 min.

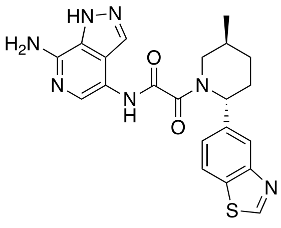

N-(7-amino-1*H*-pyrazolo[3,4-*c*]pyridin-4-yl)-2-((2*R*,5*S*)-2-(benzo[*d*]thiazol-5-yl)-5-methylpiperidin-1-yl)-2-oxoacetamide, **Compound 13**.
Oxamide-NH_2: **Intermediate 4b**. Halopyridine: **Intermediate 13a**. HPLC conditions: Column: SunFire C18, 100 x 19 mm, 5 µm; 2-10 min 0 – 45 % MeCN + FA, flow: 30 mL / min; (loading pump 4 mL / min MeCN). LCMS (ESI): [M]+ m/z: calcd 565.2; found 566.2; Rt = 1.045 min.
Deprotection: Material dissolved in mixture of MeOH and dioxane, 1:3, saturated with HCl (10% by weight), stirred for 3 h, concentrated in vacuum and purified by HPLC: 2 - 10 min 30-60 MeOH + FA 30 mL / min) to afford *N*-(7-amino-1*H*-pyrazolo[3,4-*c*]pyridin-4-yl)-2-((2*R*,5*S*)-2-(benzo[*d*]thiazol-5-yl)-5-methylpiperidin-1-yl)-2-oxoacetamide, **Compound 13** (30% yield, HCOOH). LCMS (ESI): [M]+ m/z: calcd 435.2; found 436.2; Rt = 2.046 min.

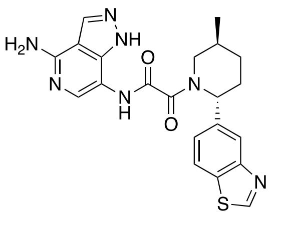

N-(4-amino-1*H*-pyrazolo[4,3-*c*]pyridin-7-yl)-2-((2*R*,5*S*)-2-(benzo[*d*]thiazol-5-yl)-5-methylpiperidin-1-yl)-2-oxoacetamide, **Compound 14**
Oxamide-NH_2: **Intermediate 4b**. Halopyridine: **75**. HPLC (column: YMC Triart C18, 100 x 20 mm 5 µm; mobile phase: 50-50-100 % 0-1-5 min H_2O / MeOH / 0.1 % NH_4OH, flow rate: 30 mL / min (loading pump 4 mL / min methanol), affording *N*-(4-amino-1-((2-(trimethylsilyl)ethoxy)methyl)-1*H*-pyrazolo[4,3-*c*]pyridin-7-yl)-2-((2*R*,5*S*)-2-(benzo[*d*]thiazol-5-yl)-5-methylpiperidin-1-yl)-2-oxoacetamide which was using in the next step as is. LCMS (ESI): [M+H]+ m/z: calcd 565.27; found 566.4; Rt = 3.325 min. The material was dissolved in a mixture of MeOH and dioxane/HCl, 1:1. The resulting mixture was stirred at 28 °C for 3 h after which the solvent was evaporated and the residue was purified by HPLC (column: Chromatorex 18 SMB100-5T, 100 x 19 mm, 5 µm; mobile phase: 5 – 5 – 45 %, 0 – 5 min H_2O / ACN / 0.1 % FA, flow rate: 30 mL / min (loading pump 4 mL / min ACN), affording **14**, *N*-(4-amino-1*H*-pyrazolo[4,3-c]pyridin-7-yl)-2-((2*R*,5*S*)-2-(benzo[d]thiazol-5-yl)-5-methylpiperidin-1-yl)-2-oxoacetamide (52% yield, HCl). ^{1}H NMR (600 MHz, DMSO-d_6) δ 1.06 (m, 3H), 1.30 (m, 2H), 1.74 (m, 1H), 1.91 (m, 1H), 2.23 (m, 2H), 3.80 (m, 1H), 5.70 (m, 1H), 6.65 (m, 2H), 7.56 (m, 2H), 8.12 (m, 4H), 9.37 (m, 1H), 10.54 (m, 1H), 12.72 (s, 1H). LCMS (ESI): [M+H]+ m/z: calcd 435.2; found 436.2; Rt = 2.452 min.

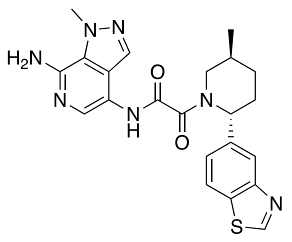

N-(7-amino-1-methyl-1*H*-pyrazolo[3,4-*c*]pyridin-4-yl)-2-((2*R*,5*S*)-2-(benzo[*d*]thiazol-5-yl)-5-methylpiperidin-1-yl)-2-oxoacetamide, **Compound 15**. Oxamide-NH_2: **Intermediate 4b**. Halopyridine: **Intermediate 15a**. HPLC (column: Chromatorex 18 SMB100-5T 100 x 19 mm 5 um; mobile phase: 10-10-60 % 0-1-5 min H_2O /ACN / 0.2 % FA; flow rate: 30 mL /min (loading pump 4 mL / min acetonitrile)) to afford *N*-(7-amino-1-methyl-1*H*-pyrazolo[3,4-*c*]pyridin-4-yl)-2-((2*R*,5*S*)-2-(benzo[*d*]thiazol-5-yl)-5-methylpiperidin-1-yl)-2-oxoacetamide, **Compound 15** (58 mg, 129.02 µmol, 29 % yield) as light-brown solid. ^{1}H NMR(600 MHz, DMSO-d_6) δ 1.00 – 1.09 (m, 3H), 1.31 – 1.45 (m, 1H), 1.69 – 1.79 (m, 1H), 1.85 – 1.98 (m, 1H), 2.10 – 2.24 (m, 1H), 2.26 – 2.40 (m, 1H), 2.85 – 3.24 (m, 1H), 3.42 – 4.10 (m, 1H), 4.24 – 4.34 (m, 3H), 5.16 – 5.85 (m, 1H), 7.14 (s, 2H), 7.43 – 7.60 (m, 1H), 7.85 – 8.05 (m, 2H), 8.10 – 8.25 (m, 2H), 9.34 – 9.46 (m, 1H), 10.87 – 11.11 (m, 1H), 12.24 – 13.17 (m, 1H). LCMS (ESI): [M+H]+ m/z: calcd 450.2; found 450.2; Rt = 2.154 min.

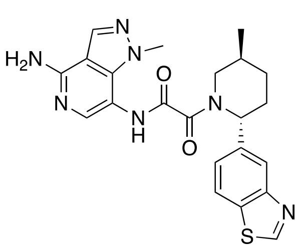

N-(4-amino-1-methyl-1*H*-pyrazolo[4,3-*c*]pyridin-7-yl)-2-((2*R*,5*S*)-2-(benzo[*d*]thiazol-5-yl)-5-methylpiperidin-1-yl)-2-oxoacetamide, **Compound 16**. Oxamide-NH_2: **Intermediate 4b**. Halopyridine: **Intermediate 16a**. HPLC 0.5-6.5 min 20 % water-ACN + NH_3; flow 30 mL / min (loading pump 4 mL ACN); column: Cromatorex Phenil 100 x 19 mm, 5 µM to afford *N*-(4-amino-1-methyl-1*H*-pyrazolo[4,3-*c*]pyridin-7-yl)-2-((2*R*,5*S*)-2-(benzo[*d*]thiazol-5-yl)-5-methylpiperidin-1-yl)-2-oxoacetamide, **Compound 16** (8% yield) as a yellow gum. ^{1}H NMR(600 MHz, DMSO-d_6) δ 0.88 – 1.11 (m, 3H), 1.19 – 1.24 (m, 1H), 1.31 – 1.42 (m, 1H), 1.42 – 1.67 (m, 1H), 1.74 – 1.84 (m, 1H), 1.88 – 1.97 (m, 1H), 1.98 – 2.10 (m, 1H), 2.10 – 2.29 (m, 1H), 2.83 – 3.10 (m, 1H), 3.85 – 4.11 (m, 3H), 5.10 – 5.81 (m, 1H), 6.72 – 7.08 (m, 2H), 7.26 – 7.61 (m, 2H), 7.81 – 8.06 (m, 1H), 8.07 – 8.20 (m, 2H), 9.31 – 9.43 (m, 1H). LCMS (ESI): [M+H]+ m/z: calcd 450.2; found 450.2; Rt = 2.149 min.

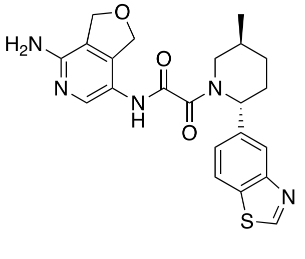

N-(4-amino-1,3-dihydrofuro[3,4-*c*]pyridin-7-yl)-2-((2*R*,5*S*)-2-(benzo[*d*]thiazol-5-yl)-5-methylpiperidin-1-yl)-2-oxoacetamide, **Compound 17**. Oxamide-NH_2: **Intermediate 4b**. Halopyridine: 7-bromo-1,3-dihydroisobenzofuran-4-amine. HPLC: SYSTEM 20-70 % 0 – 5 min H_2O / MeOH / 0.1 % NH_4OH, flow: 30 mL / min (loading pump 4 mL / min MeOH), column: XBridge C18 100 × 19 mm, 5 µm) to afford *N*-(4-amino-1,3-dihydrofuro[3,4-*c*]pyridin-7-yl)-2-((2*R*,5*S*)-2-(benzo[*d*]thiazol-5-yl)-5-methylpiperidin-1-yl)-2-oxoacetamide, **Compound 17** (29% yield) as a light-yellow solid. LCMS (ESI): [M+H]+ m/z: calcd 438.18; found 438.2; Rt = 2.281 min. ^{1}H NMR (600 MHz, DMSO-d_6) δ 1.01 – 1.10 (m, 3H), 1.31 – 1.45 (m, 1H), 1.68 – 1.80 (m, 1H), 1.86 – 1.96 (m, 1H), 2.07 – 2.23 (m, 1H), 2.27 – 2.37 (m, 1H), 2.78 – 3.25 (m, 1H), 3.40 – 4.09 (m, 1H), 4.64 – 4.76 (m, 1H), 4.75 – 4.98 (m, 3H), 5.20 – 5.79 (m, 1H), 5.85 – 6.03 (m, 2H), 7.37 – 7.54 (m, 1H), 7.73 – 7.92 (m, 1H), 7.95 – 8.10 (m, 1H), 8.15 – 8.22 (m, 1H), 9.40 (s, 1H), 10.32 – 10.65 (m, 1H).

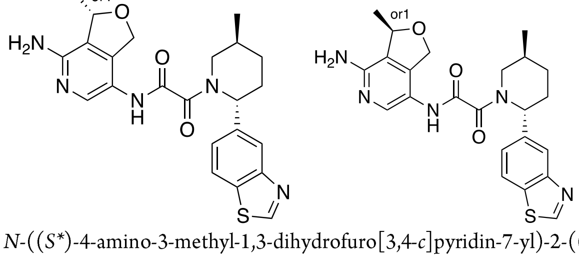

N-((*S**)-4-amino-3-methyl-1,3-dihydrofuro[3,4-*c*]pyridin-7-yl)-2-((2*R*,5*S*)-2-(benzo[*d*]thiazol-5-yl)-5-methylpiperidin-1-yl)-2-oxoacetamide, **Compound 18**.

Oxamide-NH_2: **Intermediate 4b**. Halopyridine: **Intermediate 18a**. HPLC (3-10-40 %, 0-2-10 H_2O / MeCN, flow 30 mL / min ((loading pump 4 mL MeCN), target mass 451, column : XBridge BEH C18 100 x 19 mm,5 μM) to obtain *N*-(4-amino-3-methyl-1,3-dihydrofuro[3,4-c]pyridin-7-yl)-2-oxo-2-[(2*R*,5*S*)-2-(1,3-benzothiazol-5-yl)-5-methyl-1-piperidyl]acetamide (32.7 mg, 72.42 μmol, 33 % yield). ^{1}H NMR (600 MHz, DMSO-d_6) δ 1.00 – 1.12 (m, 3H), 1.24 – 1.36 (m, 4H), 1.65 – 1.76 (m, 1H), 1.83 – 1.96 (m, 1H), 2.07 – 2.25 (m, 1H), 2.28 – 2.36 (m, 1H), 2.78 – 3.29 (m, 1H), 3.39 – 4.10 (m, 1H), 4.52 – 4.99 (m, 2H), 5.12 – 5.73 (m, 2H), 5.78 – 5.94 (m, 2H), 7.38 – 7.56 (m, 1H), 7.72 – 7.92 (m, 1H), 7.96 – 8.10 (m, 1H), 8.18 (d, 1H), 9.40 (s, 1H), 10.37 – 10.60 (m, 1H). LCMS (ESI): [M+H]+ m/z: calcd 451.2; found 452.2; Rt = 2.706 min. *N*-(4-amino-3-methyl-1,3-dihydrofuro[3,4-c]pyridin-7-yl)-2-oxo-2-[(2*R*,5*S*)-2-(1,3-benzothiazol-5-yl)-5-methyl-1-piperidyl]acetamide (24 mg, 53.15 μmol) was separated (Column: CHIRALPAK IC (250 x 21 mm, 5 μm)-II; Mobile Phase: Hexane (0.1 % DEA):IPA:MeOH, 50:25:25 Flow Rate: 14 mL / min; m = 0,024 mg, 1 inj. , 24 mg / inj., V= 2 l, 1,1 h) to obtain *N*-((*S**)-4-amino-3-methyl-1,3-dihydrofuro[3,4-*c*]pyridin-7-yl)-2-((2*R*,5*S*)-2-(benzo[*d*]thiazol-5-yl)-5-methylpiperidin-1-yl)-2-oxoacetamide, **Compound 18** (9.71 mg, 21.50 μmol, 40 % yield) and *N*-((*R**)-4-amino-3-methyl-1,3-dihydrofuro[3,4-*c*]pyridin-7-yl)-2-((2*R*,5*S*)-2-(benzo[*d*]thiazol-5-yl)-5-methylpiperidin-1-yl)-2-oxoacetamide, **Compound 19** (8.82 mg, 19.53 μmol, 37 % yield).

Compound 18 Rt = 25.206 min. ^{1}H NMR (600 MHz, DMSO-d_6) δ 0.93 – 1.08 (m, 3H), 1.21 – 1.39 (m, 4H), 1.66 – 1.77 (m, 1H), 1.84 – 1.95 (m, 1H), 2.05 – 2.33 (m, 2H), 2.76 – 3.24 (m, 1H), 3.40 – 4.13 (m, 1H), 4.49 – 4.99 (m, 2H), 5.09 – 6.23 (m, 4H), 7.32 – 7.54 (m, 1H), 7.74 – 7.90 (m, 1H), 7.98 – 8.08 (m, 1H), 8.13 – 8.26 (m, 1H), 9.30 – 9.44 (m, 1H), 10.40 – 10.57 (m, 1H). LCMS (ESI): [M+H]+ m/z: calcd 451.2; found 452.2; Rt = 2.659 min.

Compound 19 Rt = 54.349 min. ^{1}H NMR (600 MHz, DMSO-d_6) δ 0.94 – 1.10 (m, 3H), 1.24 – 1.41 (m, 4H), 1.64 – 1.82 (m, 1H), 1.82 – 1.97 (m, 1H), 2.04 – 2.35 (m, 2H), 2.75 – 3.26 (m, 1H), 3.39 – 4.12 (m, 1H), 4.53 – 5.03 (m, 2H), 5.14 – 6.28 (m, 4H), 7.37 – 7.55 (m, 1H), 7.75 – 7.89 (m, 1H), 7.97 – 8.08 (m, 1H), 8.14 – 8.20 (m, 1H), 9.32 – 9.70 (m, 1H), 10.39 – 10.54 (m, 1H). LCMS (ESI): [M+H]+ m/z: calcd 451.2; found 452.2; Rt = 2.664 min.

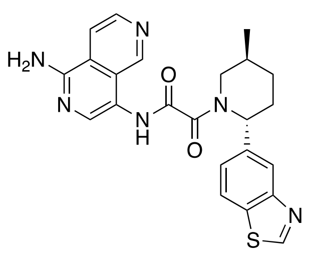

N-(1-amino-2,6-naphthyridin-4-yl)-2-((2*R*,5*S*)-2-(benzo[*d*]thiazol-5-yl)-5-methylpiperidin-1-yl)-2-oxoacetamide, **Compound 20**.

Oxamide-NH_2: **Intermediate 4b**. Halopyridine: **Intermediate 20a**. HPLC (column: Chromatorex 18 SMB100-5T 100 x 19 mm 5 μm; mobile phase: 10-10-35 % 0-1-5 min H_2O /MeCN / 0.1 % FA, flow rate: 30 mL / min) to afford *N*-(1-amino-2,6-naphthyridin-4-yl)-2-((2*R*,5*S*)-2-(benzo[*d*]thiazol-5-yl)-5-methylpiperidin-1-yl)-2-oxoacetamide, **Compound 20** (9% yield). ^{1}H NMR (600 MHz, DMSO-d_6) δ 1.05 – 1.17 (m, 3H), 1.36 – 1.47 (m, 1H), 1.56 – 1.88 (m, 2H), 1.90 – 1.98 (m, 1H), 2.10 – 2.31 (m, 1H), 2.85 – 3.17 (m, 1H), 3.59 – 4.15 (m, 1H), 5.29 – 5.86 (m, 1H), 7.17 – 7.36 (m, 2H), 7.46 – 7.57 (m, 1H), 7.99 – 8.22 (m, 4H), 8.72 (br s, 2H), 9.41 (s, 1H), 10.65 – 10.82 (m, 1H). LCMS (ESI): [M]+ m/z: calcd 446.2; found 447.2; Rt = 2.078 min.

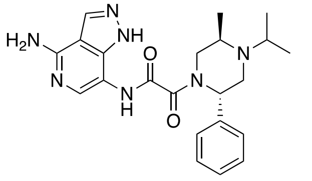

N-(4-amino-1*H*-pyrazolo[4,3-*c*]pyridin-7-yl)-2-((2*S*,5*R*)-4-isopropyl-5-methyl-2-phenylpiperazin-1-yl)-2-oxoacetamide.

Oxamide-NH_2: **Intermediate 34a**. Halopyridine: **75**. HPLC (column: XBridge C18 100 x 19 mm, 5 μm; mobile phase: 60 – 100 % 0-5 min H_2O / MeOH / 0.1 % NH_4OH, flow rate: 30 mL / min (loading pump 4 mL / min MeOH) *N*-[4-amino-2-(2-trimethylsilylethoxymethyl)pyrazolo[4,3-c]pyridin-7-yl]-2-oxo-2-[rac-(2*S*,5*R*)-4-isopropyl-5-methyl-2-phenyl-piperazin-1-yl]acetamide (80.7 mg, 146.26 μmol, 25% yield). LCMS (ESI): [M+2H]+ m/z: calcd 553.2; found 553.2; Rt = 0.724 min.

Deprotection: the material was dissolved in a mixture of dioxane / HCl and MeOH, 1:1, and the resulting mixture was stirred at 25 °C for 4 h. The solvent was evaporated to dryness to afford crude product which was purified by HPLC (column: XBridge BEH C18, 5 μm, 130 A; mobile phase: 40-40-90 % 0-1-6 min H_2O / MeOH / 0.1 % NH_4OH, flow rate: 30 mL / min (loading pump 4 mL / min MeOH), to afford *N*-(4-amino-1*H*-pyrazolo[4,3-*c*]pyridin-7-yl)-2-((2*S*,5*R*)-4-isopropyl-5-methyl-2-phenylpiperazin-1-yl)-2-oxoacetamide, **Compound 34** (22% yield). ^{1}H NMR (600 MHz, DMSO-d_6) δ 0.92 – 1.11 (m, 9H), 2.64 – 3.25 (m, 4H), 3.42 – 3.53 (m, 1H), 3.87 – 4.10 (m, 1H), 5.38 – 5.50 (m, 1H), 6.61 – 6.97 (m, 2H), 7.03 – 7.52 (m, 5H), 7.58 – 7.70 (m, 1H), 8.12 – 8.22 (m, 1H), 10.40 – 10.50 (m, 1H), 12.68 (s, 1H). LCMS (ESI): [M+H]+ m/z: calcd 422.2; found 422.2; Rt = 0.927 min.

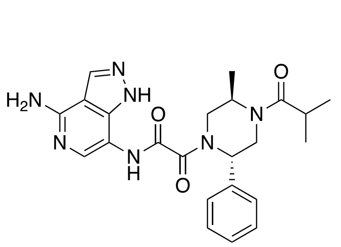

N-(4-amino-1*H*-pyrazolo[4,3-*c*]pyridin-7-yl)-2-((2*S*,5*R*)-4-isobutyryl-5-methyl-2-phenylpiperazin-1-yl)-2-oxoacetamide, **Compound 35**.

Oxamide-NH_2: **Intermediate 35b**. Halopyridine: **75**. 17% yield. HPLC conditions: Column: XBridge C18, 100 x 19 mm, 5 μM; 0-5 min 40 – 90. % water-MeOH + 0.1% NH_4OH 30 mL / min; (loading pump 4 mL / min MeOH). LCMS (ESI): [M]+ m/z: calcd 579.2; found 580.2; Rt = 1.079 min. The material was then stirred in 1/1 4 N HCl dioxane / MeOH at 25 °C for 3 h, after which it was concentrated under reduced pressure. The crude was purified by reverse phase HPLC chromatography (5-5-25 % 0-1-5 min H_2O / MeCN / 0.1 % FA, flow: 30 mL / min (loading pump 4 mL / min MeCN): Chromatorex 18 SMB100-5T, 100 x 19 mm, 5 μm) to afford product *N*-(4-amino-1*H*-pyrazolo[4,3-*c*]pyridin-7-yl)-2-((2*S*,5*R*)-4-isobutyryl-5-methyl-2-phenylpiperazin-1-yl)-2-oxoacetamide, **35** (32% yield, HCl). LCMS (ESI): [M]+ m/z: calcd 449.2; found 450.2; Rt = 1.778 min. ^{1}H NMR (600 MHz, DMSO-d_6) δ 0.26 – 1.32 (m, 9H), 2.64 – 3.19 (m, 4H), 3.56 – 5.77 (m, 4H), 6.28 – 6.99 (m, 2H), 7.02 – 7.52 (m, 5H), 7.65 – 8.25 (m, 2H), 9.68 – 10.74 (m, 1H), 12.61 – 13.39 (m, 1H).

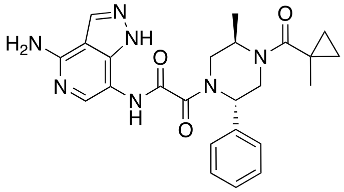

N-(4-amino-1*H*-pyrazolo[4,3-*c*]pyridin-7-yl)-2-((2*S*,5*R*)-5-methyl-4-(1-methylcyclopropane-1-carbonyl)-2-phenylpiperazin-1-yl)-2-oxoacetamide, **Compound 36**.

Oxamide-NH_2: **Intermediate 36b**. Halopyridine: **Intermediate 36a**. LCMS (ESI): [M]+ m/z: calcd 530.2; found 531.2; Rt = 1.224 min.
Deprotection: The material was dissolved in MeOH and dioxane / HCl, 1:1, was stirred at 25 °C for 16 h. Upon completion, the reaction mixture was concentrated under reduced pressure to obtain crude product which was purified by reverse phase HPLC chromatography to afford *N*-(4-amino-1*H*-pyrazolo[4,3-*c*]pyridin-7-yl)-2-((2*S*,5*R*)-5-methyl-4-(1-methylcyclopropane-1-carbonyl)-2-phenylpiperazin-1-yl)-2-oxoacetamide, **Compound 36**, 6% yield. ^{1}H NMR (600 MHz, DMSO-d_6) δ 0.48 – 0.80 (m, 2H), 1.04 (s, 3H), 1.22 – 1.31 (m, 2H), 2.75 – 2.95 (m, 2H), 3.63 – 3.64 (m, 1H), 4.12 -4.14 (m, 1H), 4.48 – 4.57 (m, 2H), 4.87 – 4.95 (m, 1H), 5.30 – 5.33 (m, 1H), 5.72 – 5.79 (m, 1H), 7.26 – 7.39 (m, 5H), 8.33 (s, 1H), 8.60 (s, 1H), 8.85 (s, 1H), 11.42 (m, 1H), 13.72 (m, 1H). LCMS (ESI): [M]+ m/z: calcd 446.2; found 447.2; Rt = 2.297 min.

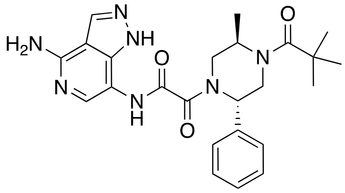

N-(4-amino-1*H*-pyrazolo[4,3-*c*]pyridin-7-yl)-2-((2*S*,5*R*)-5-methyl-2-phenyl-4-pivaloylpiperazin-1-yl)-2-oxoacetamide, **Compound 37**.

Oxamide-NH_2: **Intermediate 37b**. Halopyridine: **Intermediate 37a**. LCMS (ESI): [M-THP]+ m/z: calcd 547.2; found 548.2; Rt = 1.049 min.
Deprotection: The material was dissolved in 4N HCl/dioxane (5 mL) and MeOH (5 mL) and the solution was stirred at 25 °C for 16 h after which the reaction mixture was concentrated under reduced pressure to obtain a crude product which was purified by reverse phase HPLC: 10 – 10 – 30 % 0 – 1 – 6 min H_2O / MeCN / 0.1 % FA, flow: 30 mL / min (loading pump 4mL / min MeCN) target mass 464, column: Chromatorex 18 SMB100-5T, 100 x 19 mm, 5 μm) to afford *N*-(4-amino-1*H*-pyrazolo[4,3-*c*]pyridin-7-yl)-2-((2*S*,5*R*)-5-methyl-2-phenyl-4-pivaloylpiperazin-1-yl)-2-oxoacetamide, **Compound 37** (2 mg, 4.31 μmol, 3% yield). LCMS (ESI): [M]+ m/z: calcd 463.2; found 464.2; Rt = 2.549 min.

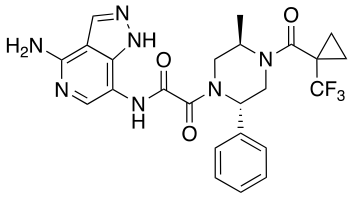

N-(4-amino-1*H*-pyrazolo[4,3-*c*]pyridin-7-yl)-2-((2*S*,5*R*)-5-methyl-2-phenyl-4-(1-(trifluoromethyl)cyclopropane-1-carbonyl)piperazin-1-yl)-2-oxoacetamide, **Compound 38**.

Oxamide-NH_2: **Intermediate 38b**. Halopyridine: **75**. Yield 21 %. HPLC conditions: Column: XBridge C18, 100 x 19 mm, 5 μM; 0-5 min 30 - 55 % water-MeCN + 0.1 % NH_4OH 30 mL / min; (loading pump 4 mL / min MeCN). LCMS (ESI): [M]+ m/z: calcd 645.2; found 646.2; Rt = 1.321 min.
Deprotection: The material was stirred in MeOH and dioxane / HCl, 1:1. The resulting reaction mixture was stirred at 25 °C for 3 h. The reaction mixture was then concentrated under reduced pressure and purified by reverse phase HPLC: 5-5-25 % 0-1-5 min H_2O / MeCN / 0.1 % FA, flow: 30 mL / min (loading pump 4 mL / min MeCN): Chromatorex 18 SMB100-5T 100 x 19 mm, 5 μm) to afford *N*-(4-amino-1*H*-pyrazolo[4,3-*c*]pyridin-7-yl)-2-((2*S*,5*R*)-5-methyl-2-phenyl-4-(1-(trifluoromethyl)cyclopropane-1-carbonyl)piperazin-1-yl)-2-oxoacetamide, **Compound 38** (38% yield, HCOOH). ^{1}H NMR (600 MHz, DMSO-d_6) δ 0.81 – 1.12 (m, 2H), 1.12 – 1.38 (m, 6H), 2.98 – 3.17 (m, 2H), 4.04 – 4.18 (m, 1H), 4.27 – 4.58 (m, 1H), 4.94 – 5.95 (m, 1H), 6.26 – 6.85 (m, 2H), 6.98 – 7.31 (m, 3H), 7.33 – 7.74 (m, 3H), 8.02 – 8.31 (m, 2H), 9.56 – 10.86 (m, 1H), 12.65 – 13.53 (m, 1H). LCMS (ESI): [M]+ m/z: calcd 551.2; found 552.2; Rt = 1.723 min.

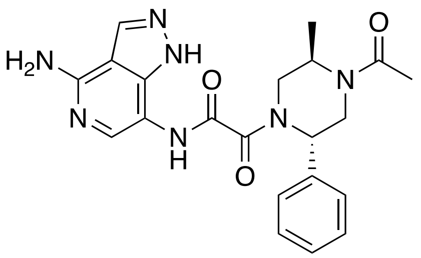

2-((2*S*,5*R*)-4-acetyl-5-methyl-2-phenylpiperazin-1-yl)-*N*-(4-amino-1*H*-pyrazolo[4,3-*c*]pyridin-7-yl)-2-oxoacetamide, **Compound 42**.
Oxamide-NH_2: **Intermediate 42a**. Halopyridine: **75**. Yield: 13%. HPLC conditions: 1st run: Column: XBridge BEH C18, 100 x 19 mm, 5 μM; 0 – 1 - 6 min, 30 – 30 – 45 % water – MeCN + 0.1 % NH_4OH 30 mL / min; (loading pump 4 mL / min MeCN). 2nd run: Column: Chromatorex C18 100 x 19 mm, 5 μM; 0 – 1 - 6 min, 55 – 55 – 60 % water – MeOH + 0.1 % FA 30 mL / min; (loading pump 4 mL / min MeOH). 3rd run: Column: Chromatorex C18, 100 x 19 mm, 5 μM; 0 – 1 - 6 min 55 – 55 – 60 % water – MeOH + 0.1 % FA 30 mL / min; (loading pump 4 mL / min MeOH). LCMS (ESI): [M]+ m/z: calcd 551.2; found 552.2; Rt = 0.954 min.
Deprotection: The material was stirred in 4 N HCl solution in dioxane and MeOH at 25 °C. The resulting solution was stirred at 25 °C for 15 h, then concentrated to dryness under vacuum and the residue was purified by reverse phase HPLC (column: Chromatorex 18 SMB100 - 5T, 100 x 19 mm, 5μm; mobile phase: 5 – 5 – 30 % 0 – 1 – 5 min H_2O / MeCN / 0.2 % FA, flow: 30 mL / min (loading pump 4 mL / min MeCN)) to afford 2-((2*S*,5*R*)-4-acetyl-5-methyl-2-phenylpiperazin-1-yl)-*N*-(4-amino-1*H*-pyrazolo[4,3-*c*]pyridin-7-yl)-2-oxoacetamide, **Compound 42** (59% yield, HCOOH) as light-yellow solid. ^{1}H NMR (600 MHz, DMSO-d_6) δ 1.01 – 1.32 (m, 3H), 1.79 – 2.04 (m, 3H), 2.65 – 3.22 (m, 1H), 3.45 – 4.47 (m, 3H), 4.49 – 6.40 (m, 2H), 6.73 – 7.02 (m, 2H), 7.04 – 7.43 (m, 5H), 7.44 – 8.28 (m, 3H), 9.64 – 10.74 (m, 1H), 12.56 – 13.56 (m, 1H). LCMS (ESI): [M+1]+ m/z: calcd 421.2; found 422.2; Rt = 1.790 min.

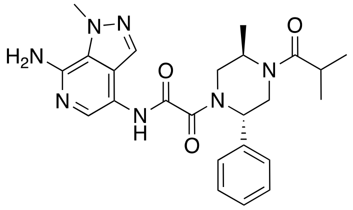

N-(7-amino-1-methyl-1*H*-pyrazolo[3,4-*c*]pyridin-4-yl)-2-((2*S*,5*R*)-4-isobutyryl-5-methyl-2-phenylpiperazin-1-yl)-2-oxoacetamide, **Compound 43**.
Oxamide-NH_2: **Intermediate 35b**. Halopyridine: **Intermediate 15a**. HPLC (0-2-9 min 0-5-60 % MeOH / H_2O + FA, 30 mL / min (loading pump 4 mL MeOH); column: Chromarorex C18 SMB100-5T, 100 x 19 mm, 5 μM) to obtain *N*-(7-amino-1-methyl-1*H*-pyrazolo[3,4-*c*]pyridin-4-yl)-2-((2*S*,5*R*)-4-isobutyryl-5-methyl-2-phenylpiperazin-1-yl)-2-oxoacetamide, **Compound 43** (6 % yield) ^{1}H NMR (600 MHz, DMSO-d_6) δ 0.75 – 0.88 (m, 3H), 0.93 – 1.03 (m, 3H), 1.14 – 1.30 (m, 3H), 2.72 – 2.80 (m, 1H), 3.15 – 3.18 (m, 1H), 3.54 – 3.87 (m, 2H), 4.06 – 4.27 (m, 4H), 4.46 – 5.06 (m, 1H), 5.20 – 5.83 (m, 1H), 6.14 – 6.30 (m, 2H), 7.21 – 7.31 (m, 2H), 7.34 – 7.40 (m, 3H), 7.59 – 7.83 (m, 1H), 7.85 – 7.96 (m, 1H), 10.47 – 10.95 (m, 1H). LCMS (ESI): [M+H]+ m/z: calcd 464.2; found 464.2; Rt = 0.792 min.

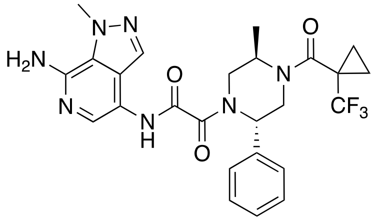

N-(7-amino-1-methyl-1*H*-pyrazolo[3,4-*c*]pyridin-4-yl)-2-((2*S*,5*R*)-5-methyl-2-phenyl-4-(1-(trifluoromethyl)cyclopropane-1-carbonyl)piperazin-1-yl)-2-oxoacetamide, **Compound 44**.
Oxamide-NH_2: **Intermediate 38b**. Halopyridine: **Intermediate 15a**. HPLC (8-15-40 % 0-2-10 H_2O / ACN / 0.1 NH_4OH, flow 30 mL / min ((loading pump; 4 mL ACN); column: XBridge BEH C18, 100 x 19 mm, 5 μM) to obtain *N*-(7-amino-1-methyl-1*H*-pyrazolo[3,4-*c*]pyridin-4-yl)-2-((2*S*,5*R*)-5-methyl-2-phenyl-4-(1-(trifluoromethyl)cyclopropane-1-carbonyl)piperazin-1-yl)-2-oxoacetamide, **Compound 44** (2 % yield). ^{1}H NMR (600 MHz, DMSO-d_6) δ 0.91 – 1.02 (m, 1H), 1.14 – 1.38 (m, 6H), 2.60 – 3.27 (m, 2H), 3.57 – 4.22 (m, 2H), 4.22 – 4.30 (m, 3H), 4.44 – 5.92 (m, 2H), 6.11 – 6.29 (m, 2H), 7.19 – 7.38 (m, 5H), 7.69 – 8.02 (m, 2H), 10.63 – 11.01 (m, 1H). LCMS (ESI): [M+H]+ m/z: calcd 530.2; found 530.2; Rt = 0.862 min.

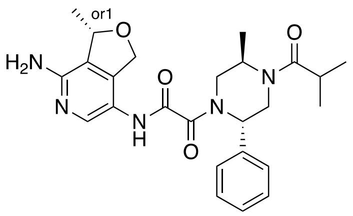

N-((*S**)-4-amino-3-methyl-1,3-dihydrofuro[3,4-*c*]pyridin-7-yl)-2-((2*S*,5*R*)-4-isobutyryl-5-methyl-2-phenylpiperazin-1-yl)-2-oxoacetamide, **Compound 45**.

Oxamide-NH_2: **Intermediate 35b**. Halopyridine: **Intermediate 18a**. HPLC (0-2-9 min, 18-25-40 % MeOH / H_2O + NH_4OH, 30 mL / min (loading pump 4 mL MeOH), column: XBridge BEH C18, 100 × 19 mm, 5 μM) to obtain *N*-(4-amino-3-methyl-1,3-dihydrofuro[3,4-c]pyridin-7-yl)-2-oxo-2-[rac-(2*S*,5*R*)-5-methyl-4-(2-methylpropanoyl)-2-phenyl-piperazin-1-yl]acetamide (41.9 mg, 90.0 μmol, 41 % yield) as a light-yellow gum. LCMS (ESI): [M-H]- m/z: calcd 466.28; found 466.0; Rt = 0.990 min. *N*-(4-amino-3-methyl-1,3-dihydrofuro[3,4-c]pyridin-7-yl)-2-oxo-2-[rac-(2*S*,5*R*)-5-methyl-4-(2-methylpropanoyl)-2-phenyl-piperazin-1-yl]acetamide (41.9 mg, 90.0 μmol) was chirally separated (Column: CHIRALPAK IC (250 × 30 mm, 10 μm), Mobile Phase: Hexane:IPA:MeOH, 50:25:25, Flow Rate: 30 mL / min, , 20 mg/inj., V = 1,8 l, 2 hr, 2 inj.) to obtain *N*-((*S**)-4-amino-3-methyl-1,3-dihydrofuro[3,4-*c*]pyridin-7-yl)-2-((2*S*,5*R*)-4-isobutyryl-5-methyl-2-phenylpiperazin-1-yl)-2-oxoacetamide, **Compound 45** (19.5 mg, 41.9 μmol, 93 % yield) and *N*-((*R**)-4-amino-3-methyl-1,3-dihydrofuro[3,4-*c*]pyridin-7-yl)-2-((2*S*,5*R*)-4-isobutyryl-5-methyl-2-phenylpiperazin-1-yl)-2-oxoacetamide, (19.1 mg, 40.9 μmol, 91 % yield) as light-yellow solids.
Analytical Column: Chiralpak IC (250 × 4.6 mm, 5 μm)-1, Mobile Phase: Hexane (0.1 % EDA):IPA:MeOH, 50:25:25, Flow Rate: 0.6 mL / min. Rt1(**Compound 45**) = 17.573 min. Rt2(*N*-((*R**)-4-amino-3-methyl-1,3-dihydrofuro[3,4-*c*]pyridin-7-yl)-2-((2*S*,5*R*)-4-isobutyryl-5-methyl-2-phenylpiperazin-1-yl)-2-oxoacetamide) = 24.988 min. Preparative Column: CHIRALPAK IC (250 × 30 mm, 10 μm), Mobile Phase: Hexane:IPA:MeOH, 50:25:25, Flow Rate: 30 mL / min, 20 mg/inj., V = 1,8 l, 2 hr, 2 inj. Rt1(**Compound 45**) = 18.34 min. Rt2 (*N*-((*R**)-4-amino-3-methyl-1,3-dihydrofuro[3,4-*c*]pyridin-7-yl)-2-((2*S*,5*R*)-4-isobutyryl-5-methyl-2-phenylpiperazin-1-yl)-2-oxoacetamide) = 26.87 min.
Compound 45: LCMS (ESI): [M+H]+ m/z: calcd 466.28; found 466.0; Rt = 2.504 min. ^{1}H NMR (600 MHz, DMSO-d_6) δ 10.58 - 10.51 (m, 1H), 10.49 - 10.18 (m, 1H), 7.89 - 7.83 (m, 1H), 7.41 - 7.3 (m, 3H), 7.3 - 7.21 (m, 2H), 5.94 - 5.81 (m, 2H), 5.68 and 5.44 (two singlets, 1H), 5.33 - 5.12 (m, 1H), 5.03 - 4.89 (m, 2H), 4.89 - 4.72 (m, 1H), 4.65 - 4.43 (m, 1.5H), 4.35 - 4.28 (m, 0.5H), 4.24 - 4.15 (m, 1H), 4.06 - 3.94 (m, 0.5H), 3.82 - 3.67 (m, 0.5H), 3.64 - 3.5 (m, 1H), 3.24 - 3.11 (m, 1H), 2.83 - 2.59 (m, 2H), 1.38 - 1.29 (m, 3H), 1.28 - 0.63 (m, 6H). *N*-((*R**)-4-amino-3-methyl-1,3-dihydrofuro[3,4-*c*]pyridin-7-yl)-2-((2*S*,5*R*)-4-isobutyryl-5-methyl-2-phenylpiperazin-1-yl)-2-oxoacetamide: LCMS (ESI): [M+H]+ m/z: calcd 466.28; found 466.0; Rt = 2.509 min.

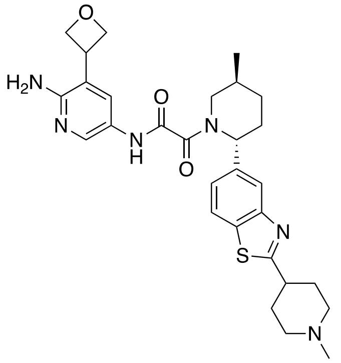

N-(6-amino-5-(oxetan-3-yl)pyridin-3-yl)-2-((2*R*,5*S*)-5-methyl-2-(2-(1-methylpiperidin-4-yl)benzo[*d*]thiazol-5-yl)piperidin-1-yl)-2-oxoacetamide, **Compound 57**.
Oxamide-NH_2: **Intermediate 57b**. Halopyridine: **Intermediate 4a**. Yield: 8 %. HPLC conditions: 15 – 40 % 0 – 5 min H_2O / MeCN / 0.1 % NH_4OH, flow: 30 mL / min (loading pump 4 mL / min MeCN), column: XBridge C18, 100 x 19 mm, 5 μm.). ^{1}H NMR (600 MHz, DMSO-d_6) δ 1.01 – 1.08 (m, 3H), 1.31 – 1.43 (m, 1H), 1.63 – 1.73 (m, 1H), 1.73 – 2.00 (m, 4H), 2.00 – 2.14 (m, 5H), 2.18 (s, 3H), 2.26 – 2.35 (m, 1H), 2.80 – 2.85 (m, 2H), 3.03 – 3.11 (m, 1H), 3.44 – 4.10 (m, 1H), 4.12 – 4.28 (m, 1H), 4.43 – 4.59 (m, 2H), 4.81 – 4.99 (m, 2H), 5.26 – 5.58 (m, 1H), 5.58 – 5.76 (m, 2H), 7.32 – 7.45 (m, 1H), 7.66 – 7.79 (m, 1H), 7.84 – 7.94 (m, 1H), 7.98 – 8.18 (m, 2H), 10.66 (br s, 1H). LCMS (ESI): [M+1]+ m/z: calcd 549.4; found 549.4; Rt = 1.895 min.

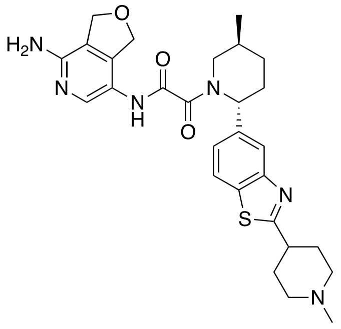

N-(4-amino-1,3-dihydrofuro[3,4-*c*]pyridin-7-yl)-2-((2*R*,5*S*)-5-methyl-2-(2-(1-methylpiperidin-4-yl)benzo[*d*]thiazol-5-yl)piperidin-1-yl)-2-oxoacetamide, **Compound 59**.
Oxamide-NH_2: **Intermediate 57b**. Halopyridine: 7-bromo-1,3-dihydrofuro[3,4-*c*]pyridin-4-amine. HPLC: 20-20-45 % 0-2-5 min H_2O / MeOH / 0.2 % FA, flow: 30mL / min (loading pump 4 mL / min MeOH), column: Chromatorex 18 SMB100-5T 100 × 19 mm, 5 μm) to *N*-(4-amino-1,3-dihydrofuro[3,4-*c*]pyridin-7-yl)-2-((2*R*,5*S*)-5-methyl-2-(2-(1-methylpiperidin-4-yl)benzo[*d*]thiazol-5-yl)piperidin-1-yl)-2-oxoacetamide, **Compound 59,** 25 % yield, as a light-yellow solid. ^{1}H NMR (600 MHz, DMSO-d_6) δ 0.81 – 1.06 (m, 3H), 1.30 – 1.40 (m, 1H), 1.64 – 1.95 (m, 4H), 2.02 – 2.10 (m, 4H), 2.15 – 2.33 (m, 4H), 2.74 – 3.22 (m, 4H), 3.38 – 4.08 (m, 2H), 4.59 – 4.95 (m, 4H), 5.14 – 5.80 (m, 1H), 5.90 – 6.06 (m, 2H), 7.30 – 7.45 (m, 1H), 7.70 – 7.94 (m, 2H), 8.03 – 8.11 (m, 1H), 10.38 – 10.65 (m, 1H). LCMS (ESI): [M-H]- m/z: calcd 533.27; found 533.2; Rt = 1.775 min.

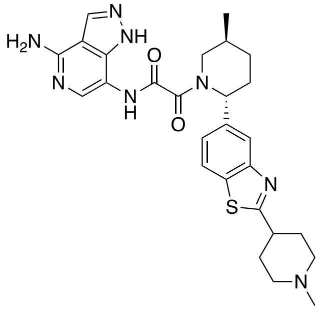

N-(4-amino-1H-pyrazolo[4,3-c]pyridin-7-yl)-2-((2R,5S)-5-methyl-2-(2-(1-methylpiperidin-4-yl)benzo[d]thiazol-5-yl)piperidin-1-yl)-2-oxoacetamide, **Compound 60**.

Oxamide-NH_2: **Intermediate 57b**. Halopyridine: **75**. Yield: 3 %. HPLC conditions: Column: 30-55 % 0-5 min H_2O / MeCN / 0.1 % NH_4OH, flow: 30 mL / min (loading pump 4 mL / min ACN), column: XBridge C18, 100 x 19 mm, 5 µm. LCMS (ESI): [M+1]+ m/z: calcd 663.2; found 663.2; Rt = 2.489 min. Deprotection: The material was stirred in MeOH (0.5 mL) and hydrogen chloride solution 4.0 M in dioxane (400.00 mg, 10.97 mmol, 0.5 mL) was added. The resulting mixture was stirred at 25 °C for 12 hr. The reaction mixture was concentrated under reduce pressure and was then purified by reverse phase HPLC: 40-40-90 % 0-1-5 min H_2O / MeOH / 0.1 % NH_4OH, flow: 30 mL / min (loading pump 4 mL / min methanol) column: YMC Triart C18, 100 x 20 mm, 5 µm) to give N-(4-amino-1H-pyrazolo[4,3-c]pyridin-7-yl)-2-((2R,5S)-5-methyl-2-(2-(1-methylpiperidin-4-yl)benzo[d]thiazol-5-yl)piperidin-1-yl)-2-oxoacetamide, **Compound 60**, 16% yield. ^{1}H NMR (600 MHz, DMSO-d_6) δ 0.99 – 1.10 (m, 3H), 1.33 – 1.42 (m, 1H), 1.66 – 1.75 (m, 1H), 1.86 – 1.95 (m, 1H), 2.06 – 2.15 (m, 2H), 2.26 – 2.34 (m, 3H), 2.71 – 2.91 (m, 4H), 3.05 – 3.15 (m, 2H), 3.42 – 4.11 (m, 5H), 5.41 – 5.76 (m, 1H), 7.42 – 7.50 (m, 1H), 7.89 – 8.02 (m, 2H), 8.05 – 8.13 (m, 1H), 8.48 – 10.48 (m, 3H), 10.90 – 11.23 (m, 1H), 12.22 – 14.15 (m, 1H). LCMS (ESI): [M+1]- m/z: calcd 531.2; found 531.2; Rt = 2.155 min.

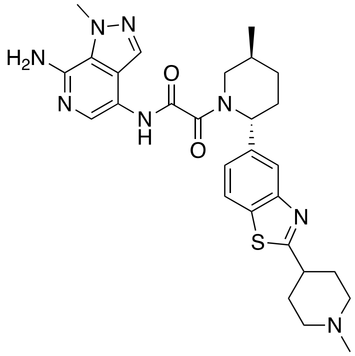

N-(7-amino-1-methyl-1H-pyrazolo[3,4-c]pyridin-4-yl)-2-((2R,5S)-5-methyl-2-(2-(1-methylpiperidin-4-yl)benzo[d]thiazol-5-yl)piperidin-1-yl)-2-oxoacetamide, **Compound 61**.

Oxamide-NH_2: **Intermediate 57b**. Halopyridine: **Intermediate 15a**. HPLC: 30-30-80 % 0-1.3-6.3 min H_2O / MeOH / 0.1 % NH_4OH, flow: 30 mL / min (loading pump 4 mL / min MeOH); column: XBridge BEH C18, 5 µm, 130 A) to afford N-(7-amino-1-methyl-1H-pyrazolo[3,4-c]pyridin-4-yl)-2-((2R,5S)-5-methyl-2-(2-(1-methylpiperidin-4-yl)benzo[d]thiazol-5-yl)piperidin-1-yl)-2-oxoacetamide, **Compound 61**, 8% yield. LCMS (ESI): [M+H]+ m/z: calcd 547.2; found 547.2; Rt = 1.685 min. ^{1}H NMR (600 MHz, DMSO-d_6) δ 10.87 - 10.65 (m, 1H), 8.13 - 8.02 (m, 1H), 8 - 7.94 (m, 1H), 7.94 - 7.86 (m, 1H), 7.83 - 7.72 (m, 1H), 7.52 - 7.36 (m, 1H), 6.31 - 6.1 (m, 2H), 5.88 - 5.7 (m, 1H), 5.27 (s, 1H), 4.35 - 4.17 (m, 3H), 3.47 (d, J = 13.2 Hz, 1H), 3.14 - 3.00 (m, 1H), 2.85 (m, 2H), 2.42 - 2.28 (m, 1H), 2.2 (s + m, 3H + 1H), 2.15 - 1.98 (m, 4H), 1.98 - 1.65 (m, 4H), 1.47 - 1.28 (m, 1H), 1.08 (d, J = 6.9 Hz, 3H).

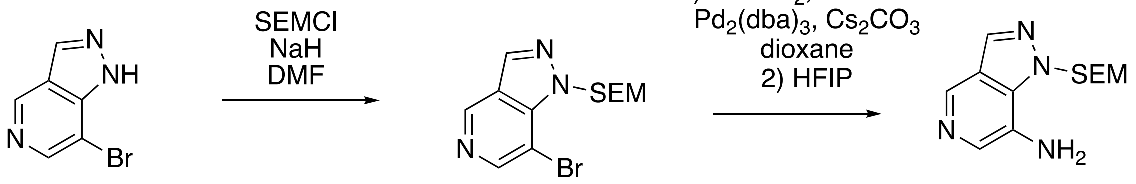

Intermediate 10a

7-bromo-1-((2-(trimethylsilyl)ethoxy)methyl)-1H-pyrazolo[4,3-c]pyridine.

Sodium hydride (212.85 mg, 5.55 mmol, 60 % dispersion in mineral oil) was added portionwise to a solution of 7-bromo-1H-pyrazolo[4,3-c]pyridine (1 g, 5.05 mmol) in DMF (20 mL) at 0 °C . When H_2 evolution ceased, 2-(chloromethoxy)ethyl-trimethyl-silane (884.04 mg, 5.30 mmol, 938.47 µL) was added dropwise. The resulting mixture was stirred at 0 °C for 3 h after which it was diluted with water (60 mL) and extracted with MTBE (50 mL). The organic layer was washed successively with water (20 mL) and brine (20 mL), dried over Na_2SO_4, and concentrated under reduced pressure, affording 7-bromo-1-((2-(trimethylsilyl)ethoxy)methyl)-1H-pyrazolo[4,3-c]pyridine (1.6 g, 4.87 mmol, 97% yield). LCMS (ESI): [M]+ m/z: calcd 328.2; found 329.2; Rt = 1.458 min.

1-((2-(trimethylsilyl)ethoxy)methyl)-1*H*-pyrazolo[4,3-*c*]pyridin-7-amine, **Intermediate 10a**.
7-bromo-1-((2-(trimethylsilyl)ethoxy)methyl)-1*H*-pyrazolo[4,3-*c*]pyridine (2.2 g, 6.70 mmol), *tert*-butyl carbamate (1.18 g, 10.05 mmol) and cesium carbonate (3.28 g, 10.05 mmol) were stirred in dioxane (40 mL). The reaction flask was evacuated and refilled three times with Ar after which $Pd_2(dba)_3$ (153.42mg, 167.54 μmol) and Xantphos (193.88 mg, 335.08 μmol) were added under stream of Ar. The mixture was stirred at 100 °C for 16 h, then it was cooled, diluted with MTBE (50 mL), and filtered through a short pad of silica gel. The filtrate was concentrated under reduced pressure, affording *tert*-butyl *N*-[1-(2-trimethylsilylethoxymethyl)pyrazolo[4,3-c]pyridin-7-yl]carbamate (3.1 g, crude).
LCMS (ESI): [M]+ m/z: calcd 364.2; found 365.2; Rt = 1.374 min. This material (3.1 g, 8.50 mmol) was dissolved in HFIP (24.00 g, 142.82 mmol, 15 mL) and the resulting solution was stirred at 58 °C for 96 h. The solvent was removed under reduced pressure affording 1-((2-(trimethylsilyl)ethoxy)methyl)-1*H*-pyrazolo[4,3-*c*]pyridin-7-amine, **Intermediate 10a** (2.7 g, crude) which was used as is. LCMS (ESI): [M]+ m/z: calcd 264.2; found 265.2; Rt = 1.093 min.

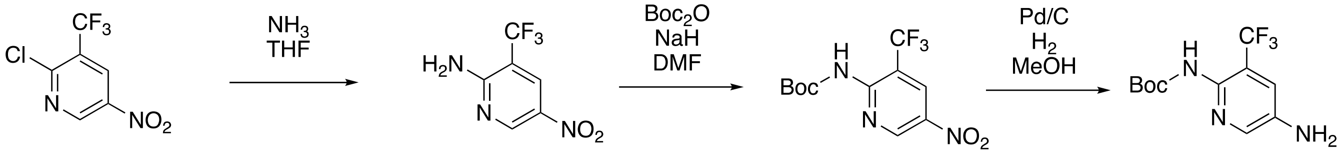

Intermediate 6a

5-nitro-3-(trifluoromethyl)pyridin-2-amine.
A solution of 2-chloro-5-nitro-3-(trifluoromethyl)pyridine (4.50 g, 19.9 mmol) and ammonia (338 mg, 19.8 mmol) in THF (50.0 mL) was stirred at room temperature for 14 h. The reaction mixture was filtered and concentrated under reduced pressure to afford 5-nitro-3-(trifluoromethyl)pyridin-2-amine (3.50 g, crude) as a white solid. LCMS (ESI): [M+H]+ m/z: calcd 208.02; found 208.0; Rt = 0.965 min.

tert-butyl (5-nitro-3-(trifluoromethyl)pyridin-2-yl)carbamate.
To a solution of 5-nitro-3-(trifluoromethyl)pyridin-2-amine (3.50 g, 16.9 mmol) in DMF (15.0 mL) was added sodium hydride (744 mg, 18.6 mmol, 60 % dispersion in mineral oil) portionwise at 0 °C. The resulting mixture was stirred for 0.5 h (until the end of gas evolution), then a solution of di-tert-butyl dicarbonate (4.06 g, 18.6 mmol, 4.27 mL) in DMF (5.00 mL) was added dropwise. The resulting mixture was stirred at 25 °C for 16 h after which water (1000 mL) was added and the mixture was extracted 3 times with EtOAc. The combined organic layers were washed with water, brine, dried and concentrated in vacuo to afford *tert*-butyl (5-nitro-3-(trifluoromethyl)pyridin-2-yl)carbamate (6.80 g, crude) as a brown solid. LCMS (ESI): [M-H]+ m/z: calcd 306.08; found 306.2; Rt = 1.213 min.

tert-butyl (5-amino-3-(trifluoromethyl)pyridin-2-yl)carbamate, **Intermediate 6a**.
To a solution of *tert*-butyl (5-nitro-3-(trifluoromethyl)pyridin-2-yl)carbamate (5.00 g, 16.3 mmol) in MeOH (125 mL) was added Pd/C (0.50 g, 16.3 mmol). The reaction mixture was evacuated and then backfilled with H_2 and stirred overnight. The resulting mixture was filtered through a thin pad of silica gel, concentrated in vacuo and dried to afford *tert*-butyl (5-amino-3-(trifluoromethyl)pyridin-2-yl)carbamate, **Intermediate 6a** (4.00 g, crude) as a beige solid. LCMS (ESI): [M-H]+ m/z: calcd 276.12; found 276.2; Rt = 1.035.

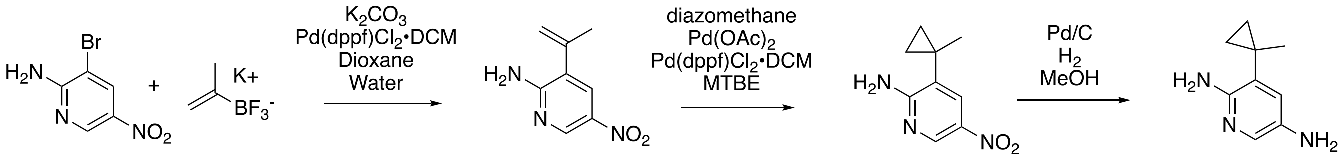

Intermediate 7a

5-nitro-3-(prop-1-en-2-yl)pyridin-2-amine.
To a solution of 3-bromo-5-nitro-pyridin-2-amine (1.00 g, 4.59 mmol) in dioxane (10.0 mL) and water (2.00 mL) was added potassium trifluoro(prop-1-en-2-yl)borate (1.02 g, 6.88 mmol), potassium carbonate, anhydrous, 99% (1.90 g, 13.8 mmol, 831 μL) and [1,1'-bis(diphenylphosphino)ferrocene]dichloropalladium(II), complex with dichloromethane (37.5 mg, 45.9 μmol). The reaction mixture was purged with Ar for 2 min and heated to 90 °C for 16 h. The mixture was concentrated in vacuo and partitioned between water and EtOAc. The aqueous layer was extracted with EtOAc. The combined organic layers were concentrated in vacuo to dryness, triturated with DCM, and filtered to afford 5-nitro-3-(prop-1-en-2-yl)pyridin-2-amine (0.90 g, crude) as a greenish solid. LCMS (ESI): [M+H]+ m/z: calcd 180.07; found 180.2; Rt = 2.546 min.

3-(1-methylcyclopropyl)-5-nitropyridin-2-amine.
To a solution of 5-nitro-3-(prop-1-en-2-yl)pyridin-2-amine (0.90 g, 5.02 mmol) in MTBE (30.0 mL) was added palladium (II) acetate (56.4 mg, 251 μmol). The resulting mixture was cooled to -60 °C and a solution of diazomethane (634 mg, 15.1 mmol, 25.0 mL) in MTBE (250 mL) was added dropwise. The mixture was warmed to room temperature and [1,1'-bis(diphenylphosphino)ferrocene]palladium(II) dichloride (36.8 mg, 50.2 μmol) was added. The resulting mixture was filtered and the filtrate was concentrated in vacuo and purified by HPLC (0.5 - 6.5 min 25 – 50 % water – ACN, + 0.1 % vol. of 25 % aq. NH_3, 30 mL / min, column: XBridge, 100 x 20 mm, 5 μm) to afford 3-(1-methylcyclopropyl)-5-nitropyridin-2-amine (24.3 mg, 126 μmol, 3% yield) as a beige solid. LCMS (ESI): [M+H]+ m/z: calcd 194.09; found 194.4; Rt = 2.644 min.

3-(1-methylcyclopropyl)pyridine-2,5-diamine, **Intermediate 7a**.

To a solution of 3-(1-methylcyclopropyl)-5-nitropyridin-2-amine (24.3 mg, 126 µmol) in MeOH (5.00 mL) was added palladium (10% on carbon, Type 487, 13.4 mg, 126 µmol). The reaction flask was evacuated and then backfilled with H_2 and stirred overnight. The reaction was filtered through a thin pad of silica gel and concentrated in vacuo to afford 3-(1-methylcyclopropyl)pyridine-2,5-diamine, **Intermediate 7a** (20.5 mg, crude) as a yellow oil. LCMS (ESI): [M+H]+ m/z: calcd 164.13; found 164.0; Rt = 1.018 min.

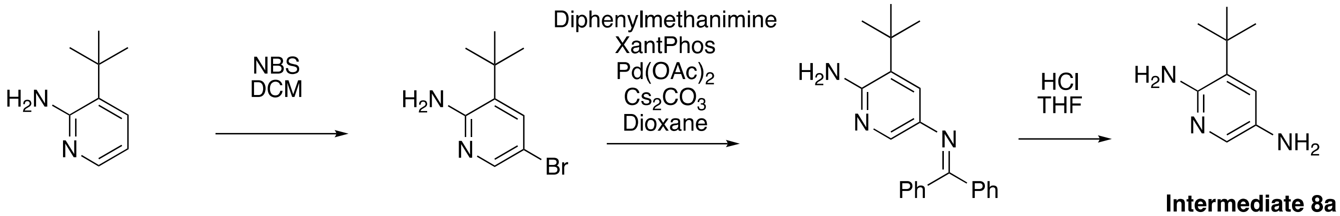

Intermediate 8a

5-bromo-3-(*tert*-butyl)pyridin-2-amine.
NBS (6.52 g, 36.6 mmol, 3.10 mL) was added portionwise to a solution of 3-*tert*-butylpyridin-2-amine (5.00 g, 33.3 mmol) in dry DCM (100 mL). The reaction mixture was stirred at room temperature for 3 h after which it was washed with water. The aqueous layer was extracted with dichloromethane and the combined organic extracts were washed with brine and dried over Na_2SO_4. The obtained mixture was concentrated under reduced pressure to afford 5-bromo-3-(*tert*-butyl)pyridin-2-amine (7.60 g, 33.2 mmol, 100% yield) as a brown solid. ^{1}H NMR (400 MHz, $CDCl_3$) δ 1.35 (d, 9H), 4.68 (br, 2H), 7.48 – 7.49 (m, 1H), 7.95 – 7.98 (m, 1H).

3-(*tert*-butyl)-5-((diphenylmethylene)amino)pyridin-2-amine.
5-bromo-3-(*tert*-butyl)pyridin-2-amine (7.60 g, 33.2 mmol), diphenylmethanimine (7.82 g, 43.1 mmol, 7.24 mL), cesium carbonate (32.4 g, 99.5 mmol), XantPhos (960 mg, 1.66 mmol) and $Pd(OAc)_2$ (759 mg, 3.38 mmol) were stirred in dioxane (250 mL). The flask was evacuated and then backfilled with Ar and the reaction mixture was stirred at 90 °C for 12 h after which it was cooled to room temperature, diluted with MTBE (250 mL), and filtered. The filtrate was concentrated in vacuo and the residue was purified by column chromatography (Interchim; 330g SiO_2, petroleum ether / ethyl acetate with ethyl acetate from 5 ~ 60 %, flow rate = 135 mL / min, Rv = 10 - 13 CV) to afford 3-(*tert*-butyl)-5-((diphenylmethylene)amino)pyridin-2-amine (8.00 g, 24.3 mmol, 73% yield) as a brown gum. LCMS (ESI): [M+H]+ m/z: calcd 330.23; found 330.4; Rt = 1.114 min.

3-(*tert*-butyl)pyridine-2,5-diamine, **Intermediate 8a**.
Hydrogen chloride (221 mg, 6.07 mmol, 10.0 mL) was added to a solution of 3-(*tert*-butyl)-5-((diphenylmethylene)amino)pyridin-2-amine (2.00 g, 6.07 mmol) in THF (50 mL). The resulting mixture was stirred at 21 °C for 1 h after which it was diluted with water (20 mL) and extracted with MTBE (2 x 20 mL). The aqueous layer was basified to pH ≈ 10 - 11 with K_2CO_3 and extracted with DCM (4 x 10 mL). The organic layers were dried over K_2CO_3 and concentrated under reduced pressure to afford 3-(*tert*-butyl)pyridine-2,5-diamine, **Intermediate 8a** (69 % yield) as a red oil. LCMS (ESI): [M+H]+ m/z: calcd 166.15; found 166.0; Rt = 0.460 min.

Compounds 1, 6 – 8, 10

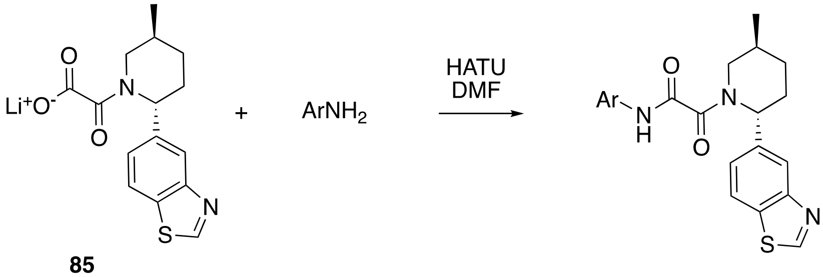

85

General Procedure C
Aryl amine (1 eq) and HATU (1.05 eq) were stirred in DMF (0.2 M) at room temperature. The mixture was stirred for 15 minutes after which **85** (1 eq) was added. The solution was stirred overnight, after which it was poured into water and extracted with EtOAc. The combined organic layers were washed with water, then brine, then concentrated in vacuo. The residue was purified as described and deprotected where appropriate.

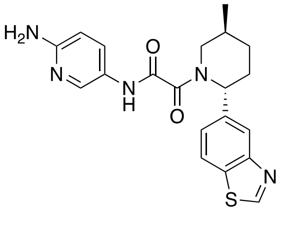

N-(6-aminopyridin-3-yl)-2-((2*R*,5*S*)-2-(benzo[*d*]thiazol-5-yl)-5-methylpiperidin-1-yl)-2-oxoacetamide, **Compound 1**.

Arylamine: *tert*-butyl (5-aminopyridin-2-yl)carbamate. HPLC (0.5 - 6.5 min 40 – 65 % water - ACN; flow: 30 mL / min, column: Waters SunFire C18, 100 x 19 mm, 5 µm) to afford *tert*-butyl *N*-[5-[[2-[(2*R*,5*S*)-2-(1,3-benzothiazol-5-yl)-5-methyl-1-piperidyl]-2-oxo-acetyl]amino]-2-pyridyl]carbamate (61 % yield) as a beige solid. LCMS (ESI): [M+H]+ m/z: calcd 496.22; found 496.0; Rt = 3.822 min.
Deprotection: The material was dissolved in dioxane /water (1:1). The reaction mixture was heated at 90 °C overnight then concentrated in vacuo to dryness. The residue was purified by HPLC (0.5 - 6.5 min 30 – 55 % water - ACN; flow: 30 mL / min, column: Waters SunFire C18, 100 x 19 mm, 5 µm) to afford *N*-(6-aminopyridin-3-yl)-2-((2*R*,5*S*)-2-(benzo[*d*]thiazol-5-yl)-5-methylpiperidin-1-yl)-2-oxoacetamide, **Compound 1** (57 % yield) as a yellow solid. ^{1}H NMR (600 MHz, DMSO-d_6) δ 1.02 – 1.05 (m, 3H), 1.21 – 1.41 (m, 2H), 1.71 (m, 1H), 1.88 (m, 1H), 2.05 – 2.30 (m, 1H), 2.78 (m, 1H), 3.50 (m, 1H), 4.06 (m, 1H), 5.72 – 5.84 (m, 2H), 6.35 – 6.45 (m, 1H), 7.42 – 7.62 (m, 2H), 8.01 – 8.19 (m, 3H), 9.40 (m, 1H), 10.60 (m, 1H). LCMS (ESI): [M+H]+ m/z: calcd 396.16; found 396.2; Rt = 2.423 min.

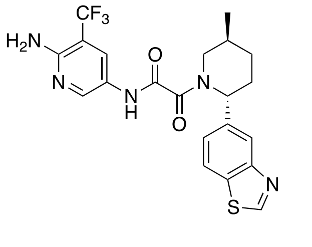

N-(6-amino-5-(trifluoromethyl)pyridin-3-yl)-2-((2*R*,5*S*)-2-(benzo[*d*]thiazol-5-yl)-5-methylpiperidin-1-yl)-2-oxoacetamide, **Compound 6**.
Arylamine: **Intermediate 6a**. HPLC (0.5 - 6.5 min 30 – 70 % water - ACN; flow: 30 mL / min, column: Waters SunFire C18, 100 x 19 mm, 5 µm) to afford *tert*-butyl *N*-[5-[[2-[(2*R*,5*S*)-2-(1,3-benzothiazol-5-yl)-5-methyl-1-piperidyl]-2-oxo-acetyl]amino]-3-(trifluoromethyl)-2-pyridyl]carbamate (114 mg, 202 µmol, 37 % yield) as a yellow solid. LCMS (ESI): [M-H]+ m/z: calcd 562.21; found 562.0; Rt = 3.879 min.
Deprotection: The material was dissolved in dioxane / water (1:1) and heated at 90 °C overnight after which it was concentrated in vacuo. The residue was purified by HPLC (0.5 - 6.5 min 40 – 65 % water - ACN; flow: 30 mL / min, column: Waters SunFire C18, 100 x 19 mm, 5 µm) to afford *N*-(6-amino-5-(trifluoromethyl)pyridin-3-yl)-2-((2*R*,5*S*)-2-(benzo[*d*]thiazol-5-yl)-5-methylpiperidin-1-yl)-2-oxoacetamide, **Compound 6** (46 % yield) as a yellow solid. Further purification using chiral chromatography increased ee to 99+. Analytical: RT (Chiralcel OD-H (250 x 4.6 mm, 5 µm), Hexane-IPA-MeOH, 70-15-15, 0.6 mL / min) - 16.728 min; Preparative: RT (Chiralcel OD-H (250 x 20, 5 µm), Hexane- IPA-MeOH, 70-15-15, 12 mL / min) - 18.584 min. ^{1}H NMR (600 MHz, DMSO-d_6) δ 1.02 – 1.05 (m, 3H), 1.22 – 1.40 (m, 1H), 1.72 – 1.92 (m, 2H), 2.05 – 2.19 (m, 1H), 2.30 – 2.37 (m, 1H), 2.82 (m, 1H), 3.54 (m, 1H), 4.03 (m, 1H), 5.37 – 5.72 (m, 2H), 6.39 (m, 1H), 7.43 – 7.49 (m, 1H), 7.99 – 8.43 (m, 3H), 9.40 (m, 1H), 10.92 (m, 1H). LCMS (ESI): [M+H]+ m/z: calcd 464.15; found 464.2; Rt = 3.482 min.

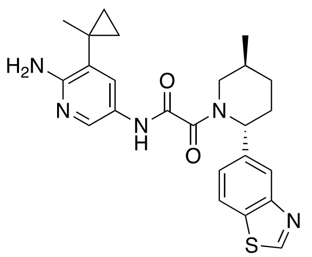

N-(6-amino-5-(1-methylcyclopropyl)pyridin-3-yl)-2-((2*R*,5*S*)-2-(benzo[*d*]thiazol-5-yl)-5-methylpiperidin-1-yl)-2-oxoacetamide, **Compound 7**.
Arylamine: **Intermediate 7a**. HPLC (0.5 - 6.5 min 30 – 45 % water – ACN, + 0.1 % vol. of 25 % aq. NH_3, 30 mL/min, column: XBridge, 100 x 20 mm, 5 µm) to afford *N*-(6-amino-5-(1-methylcyclopropyl)pyridin-3-yl)-2-((2*R*,5*S*)-2-(benzo[*d*]thiazol-5-yl)-5-methylpiperidin-1-yl)-2-oxoacetamide, **Compound 7** (7% yield) as a beige solid. ^{1}H NMR (600 MHz, DMSO-d_6) δ 0.55 – 0.79 (m, 4H), 1.02 – 1.22 (m, 6H), 1.33 – 1.40 (m, 1H), 1.68 – 1.91 (m, 2H), 2.07 – 2.30 (m, 2H), 2.78 – 2.80 (m, 1H), 3.49 – 4.06 (m, 2H), 5.31 – 5.72 (m, 3H), 7.42 – 7.61 (m, 2H), 8.01 – 8.19 (m, 3H), 9.40 (m, 1H), 10.54 – 10.59 (m, 1H). LCMS (ESI): [M+H]+ m/z: calcd 450.22; found 450.2; Rt = 2.602 min.

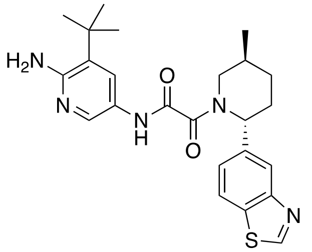

N-(6-amino-5-(*tert*-butyl)pyridin-3-yl)-2-((2*R*,5*S*)-2-(benzo[*d*]thiazol-5-yl)-5-methylpiperidin-1-yl)-2-oxoacetamide, **Compound 8**.
Arylamine: **Intermediate 8a**. HPLC (0.5 - 6.5 min 45 – 70 % water - ACN; flow: 30 mL / min, column: Waters SunFire C18, 100 x 19 mm, 5 µm) to afford *N*-(6-amino-5-(*tert*-butyl)pyridin-3-yl)-2-((2*R*,5*S*)-2-(benzo[*d*]thiazol-5-yl)-5-methylpiperidin-1-yl)-2-oxoacetamide, **Compound 8** (30 %) as a yellow solid. Analytical - Chiralpak IC (250 x 4.6 mm, 5 µm), Hexane-IPA-MeOH, 50-25-25, 0.6 mL / min. RT = 53.109min. Preparative - Chiralpak IC-III (250 x 20 mm, 5 µm), Hexane-IPA-MeOH, 50-25-25, 12 mL / min. RT = 58.670 min
^{1}H NMR (600 MHz, DMSO-d_6) δ 10.68 – 10.49 (m, 1H), 9.42 – 9.37 (m, 1H), 8.20 – 8.09 (m, 2H), 8.06 – 8.00 (m, 1H), 7.67 – 7.55 (m, 1H), 7.53 – 7.41 (m, 1H), 5.74 – 5.30 (m, 3H), 4.08 – 3.48 (m, 1H), 3.18 – 2.77 (m, 1H), 2.31 (d, 1H), 2.22 – 2.07 (m, 1H), 1.94 – 1.83 (m, 1H), 1.76 – 1.65 (m, 1H), 1.42 – 1.33 (m, 1H), 1.32 – 1.25 (m, 9H), 1.06 – 1.02 (m, 3H). LCMS (ESI): [M+H]+ m/z: calcd 452.24; found 452.2; Rt = 2.371 min.

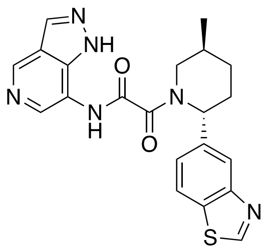

2-((2*R*,5*S*)-2-(benzo[*d*]thiazol-5-yl)-5-methylpiperidin-1-yl)-2-oxo-*N*-(1*H*-pyrazolo[4,3-*c*]pyridin-7-yl)acetamide, **Compound 10**.
Arylamine: **Intermediate 10a**. HPLC (0.5 - 6.5 min 73 % water-MeOH, 30 mL / min (loading pump 4 mL MeOH); column: SunFire, 100 x 19 mm, 5 μM) to afford 2-[(2*R*,5*S*)-2-(1,3-Benzothiazol-5-yl)-5-methyl-1-piperidyl]-2-oxo-*N*-[1-(2-trimethylsilylethoxymethyl)pyrazolo[4,3-c]pyridin-7-yl]acetamide (19 % yield) as a brown solid. LCMS (ESI): [M]+ m/z: calcd 550.2; found 551.2; Rt = 4.100 min.
Deprotection: The material was stirred in dioxane and 4.0 M hydrogen chloride solution in dioxane (1.84 g, 50.55 mmol, 2.30 mL) was added at 21 °C then stirred for 72 h after which it was evaporated to dryness and purified by HPLC (0.5 - 6.5 min 20 – 45 % water-MeCN + NH_3, 30 mL / min (loading pump 4 mL / MeCN), column:YMC-ACTUS TRIART C18, 100 x 20 mm, 5 μM) followed by chiral chromatography to remove residual isomers. Ananlytical: Chiralpak AD-H (250 x 4.6 mm, 5 μm), IPA-MeOH, 50-50, 0.6 mL / min – RT = 50.736 min. Preparative: Chiralpak AD-H (250 x 20 mm, 5 μm), MeOH-IPA, 50-50, 10 mL / min - RT = 53.968 min to afford 2-((2*R*,5*S*)-2-(benzo[*d*]thiazol-5-yl)-5-methylpiperidin-1-yl)-2-oxo-*N*-(1*H*-pyrazolo[4,3-*c*]pyridin-7-yl)acetamide, **Compound 10** as a beige solid. ^{1}H NMR (600 MHz, DMSO-d_6) δ 1.04 – 1.11 (m, 3H), 1.34 – 1.45 (m, 1H), 1.72 – 1.81 (m, 1H), 1.88 – 1.99 (m, 1H), 2.14 – 2.27 (m, 1H), 2.27 – 2.36 (m, 1H), 2.87 – 3.05 (m, 1H), 3.75 – 4.20 (m, 1H), 5.58 – 5.85 (m, 1H), 7.44 – 7.54 (m, 1H), 7.99 – 8.09 (m, 1H), 8.12 – 8.22 (m, 1H), 8.35 – 8.45 (m, 1H), 8.45 – 8.62 (m, 1H), 8.93 – 9.08 (m, 1H), 9.35 – 9.46 (m, 1H), 11.09 – 11.29 (m, 1H), 13.07 – 13.31 (m, 1H). LCMS (ESI): [M]+ m/z: calcd 420.2; found 421.2; Rt = 2.388 min.

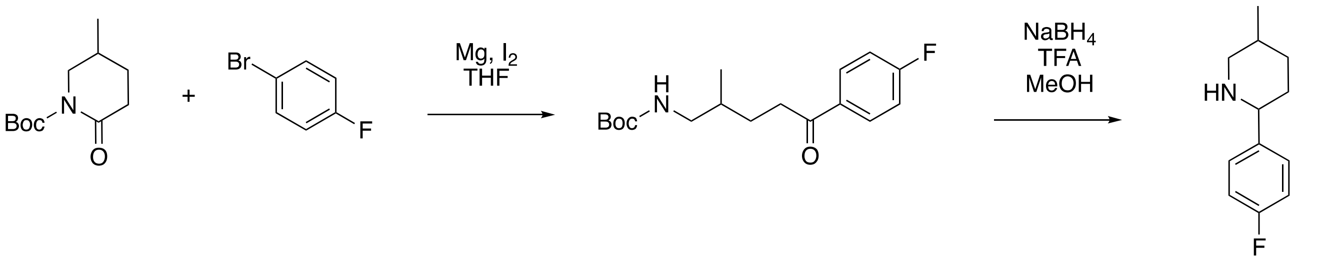

Intermediate 21a

tert-butyl (5-(4-fluorophenyl)-2-methyl-5-oxopentyl)carbamate.
To a dry 2 necked flask was added magnesium (1.04 g, 42.86 mmol, 598.65 μL), dry THF (50 mL) and 1-bromo-4-fluoro-benzene (5 g, 28.57 mmol, 3.14 mL) with stirring under Ar. Iodine (72.52 mg, 285.72 umol) was added and the mixture was heated gently until it maintained its own reflux. When reflux had subsided, external heating was applied to maintain reflux for a further 1 h. *tert*-Butyl 5-methyl-2-oxo-piperidine-1-carboxylate (6.09 g, 28.57 mmol) was added to a dry 3 necked round bottomed flask with a thermometer. Dry THF (50 mL) was added with stirring under Ar and the solution was cooled to -78 °C. The Grignard reagent was added to the *tert*-Boc-lactam over 1 h, maintaining the internal temperature below -70°C. The solution was warmed to room temperature and sat. NH_4Cl was added. The aqueous layer was extracted 3 x 50 mL with DCM and the organic layers combined, dried over Na_2SO_4, filtered, and concentrated in vacuo to afford *tert*-butyl *N*-[5-(4-fluorophenyl)-2-methyl-5-oxo-pentyl]carbamate (6.7 g, crude) as a light-yellow oil which was used in the next step without further purification. ^{1}H NMR (500 MHz, $CDCl_3$) δ 0.95 (d, 3H), 1.44 (s, 9H), 1.82 (m, 3H), 3.05 (m, 4H), 4.71 (bds, 1H), 7.13 (d, 2H), 7.99 (d, 2H). LCMS (ESI): [M-Boc]+ m/z: calcd 209.2; found 210.2; Rt = 1.508 min.

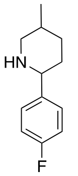

2-(4-fluorophenyl)-5-methylpiperidine, **Intermediate 21a**.
tert-butyl *N*-[5-(4-fluorophenyl)-2-methyl-5-oxo-pentyl]carbamate (6.7 g, 21.66 mmol) was stirred in trifluoroacetic acid (12.35 g, 108.28 mmol, 8.34 mL) for 1 h. 50% w/v NaOH solution was added to the mixture until the pH was 13 - 14. The product was extracted 4 x 20 mL with DCM and the organic layers combined, dried with $MgSO_4$ and evaporated. The product was dissolved in a mixture of methanol (50 mL) / water (10 mL) and added to a flask followed by sodium borohydride (819.32 mg, 21.66 mmol). The mixture was stirred under Ar overnight. The mixture was acidified with 1 - 2M HCl until the pH was 1 - 3. NaOH solution was then added until the pH was 13 - 14 and the product was extracted with DCM (4 x 100 mL), the organic layers were combined, dried with

Na_2SO_4, filtered, and evaporated to give 2-(4-fluorophenyl)-5-methylpiperidine (1.4 g, 7.24 mmol, 33% yield) as a colorless oil. ^{1}H NMR (400 MHz, $CDCl_3$) δ 0.86 (d, 3H), 1.13 (m, 1H), 1.50 (m, 1H), 1.62 (m, 1H), 1.82 (m, 3H), 2.38 (t, 1H), 3.09 (d, 1H), 3.51 (d, 1H), 6.96 (d, 2H), 7.30 (d, 2H). LCMS (ESI): [M]+ m/z: calcd 193.3; found 194.2; Rt = 1.985 min.

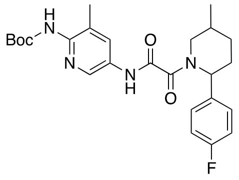

tert-butyl (5-(2-(2-(4-fluorophenyl)-5-methylpiperidin-1-yl)-2-oxoacetamido)-3-methylpyridin-2-yl)carbamate

To a solution of 2,2,2-trifluoroethyl 2-[[6-(tert-butoxycarbonylamino)-5-methyl-3-pyridyl]amino]-2-oxo-acetate, **Intermediate 21b** (585.71 mg, 1.55 mmol) and **Intermediate 21a** (0.3 g, 1.55 mmol) in THF (15 mL) was added n-Butyllithium (1.30 g, 4.66 mmol, 1.87 mL, 2.5 M in hexanes) at -78 °C under Ar atmosphere. The resulting mixture was warmed to rt, quenched with NH_4Cl aq solution, extracted with EtOAc, dried over Na_2SO_4, evaporated, and purified by HPLC (50 - 75 % ACN, 30 mL / min, Sunfire C18 19 x 100, 5 μM) to afford *tert*-butyl *N*-[5-[[2-[2-(4-fluorophenyl)-5-methyl-1-piperidyl]-2-oxo-acetyl]amino]-3-methyl-2-pyridyl]carbamate (181.7 mg, 386.16 umol, 25% yield) was obtained as a pale yellow solid. ^{1}H NMR (400 MHz, $CDCl_3$) δ 1.07 (d, 3H), 1.39 (m, 1H), 1.48 (s, 9H), 1.65 – 1.97 (m, 4H), 2.17 – 2.31 (m, 4H), 2.92 – 3.33 (m, 1H, two separate signals of rotamers), 4.19 – 4.76 (m, 1H, two separate signals of rotamers), 5.73 – 6.39 (m, 1H, two separate signals of rotamers), 6.83 (brs, 1H), 7.04 (m, 2H), 8.05 (s, 1H), 8.41 (s, 1H), 8.40 (m, 1H), 9.39 (s, 1H). LCMS (ESI): [M+1]+ m/z: calcd 470.5; found 471.2; Rt = 1.495 min.

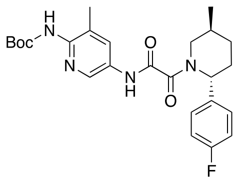

tert-butyl (5-(2-((2*R*,5*S*)-2-(4-fluorophenyl)-5-methylpiperidin-1-yl)-2-oxoacetamido)-3-methylpyridin-2-yl)carbamate.

tert-butyl *N*-[5-[[2-[2-(4-fluorophenyl)-5-methyl-1-piperidyl]-2-oxo-acetyl]amino]-3-methyl-2-pyridyl]carbamate was separated with the following conditions: IC (250 x 30, 5 μm), Hexane-IPA-MeOH, 70-15-15, 13 mL / min.

Rt for *tert*-butyl (5-(2-((2*R*,5*S*)-2-(4-fluorophenyl)-5-methylpiperidin-1-yl)-2-oxoacetamido)-3-methylpyridin-2-yl)carbamate = 23.601 min.

Rt for *tert*-butyl (5-(2-((2*S*,5*R*)-2-(4-fluorophenyl)-5-methylpiperidin-1-yl)-2-oxoacetamido)-3-methylpyridin-2-yl)carbamate = 30.270 min.

Minor cis fractions have Rt = 20.5 min and 28.5 min.

tert-butyl (5-(2-((2*R*,5*S*)-2-(4-fluorophenyl)-5-methylpiperidin-1-yl)-2-oxoacetamido)-3-methylpyridin-2-yl)carbamate: LCMS (ESI): [M+1]+ m/z: calcd 470.5; found 472.0; Rt = 5.536 min. Rt (IC, Hexane-IPA-MeOH, 70-15-15, 0.6 mL / min) = 27.123 min.

tert-butyl (5-(2-((2*S*,5*R*)-2-(4-fluorophenyl)-5-methylpiperidin-1-yl)-2-oxoacetamido)-3-methylpyridin-2-yl)carbamate: LCMS (ESI): [M+1]+ m/z: calcd 470.5; found 472.0; Rt = 5.529 min. Rt (IC, Hexane-IPA-MeOH, 70-15-15, 0.6 ml/min) = 36.392 min.

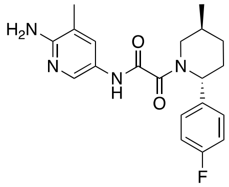

Compound 21

N-(6-amino-5-methylpyridin-3-yl)-2-((2*R*,5*S*)-2-(4-fluorophenyl)-5-methylpiperidin-1-yl)-2-oxoacetamide, **Compound 21**.

To a solution of *tert*-butyl *N*-[5-[[2-[(2*R*,5*S*)-2-(4-fluorophenyl)-5-methyl-1-piperidyl]-2-oxo-acetyl]amino]-3-methyl-2-pyridyl]carbamate (34.38 mg, 73.07 μmol) in dioxane (1 mL) was added 4 M hydrogen chloride solution in dioxane (13.32 mg, 365.33 umol, 16.65 μL) at 21 °C. The resulting mixture was left to stir for 2 h then evaporated to dryness and purified by HPLC (C18, H_2O-ACN, 34 - 51 % ACN, 30 mL / min) to afford **21**, *N*-(6-amino-5-methylpyridin-3-yl)-2-((2*R*,5*S*)-2-(4-fluorophenyl)-5-methylpiperidin-1-yl)-2-oxoacetamide (16.5 mg, 44.54 μmol, 61% yield) as a beige solid. ^{1}H NMR (600 MHz, DMSO-d_6) δ 0.92 – 1.05 (m, 3H), 1.26 – 1.38 (m, 1H), 1.60 – 1.69 (m, 1H), 1.80 – 1.90 (m, 1H), 1.95 – 2.10 (m, 4H), 2.12 – 2.25 (m, 1H), 2.66 – 3.18 (m, 1H), 3.41 – 4.03 (m, 1H), 5.05 – 5.56 (m, 1H), 5.57 – 6.08 (m, 2H), 6.94 – 7.24 (m, 2H), 7.28 – 7.40 (m, 2H), 7.42 – 7.50 (m, 1H), 7.62 – 8.08 (m, 1H), 9.69 – 10.55 (m, 1H). LCMS (ESI): [M+1]+ m/z: calcd 368.4; found 369.2; Rt = 2.787 min.

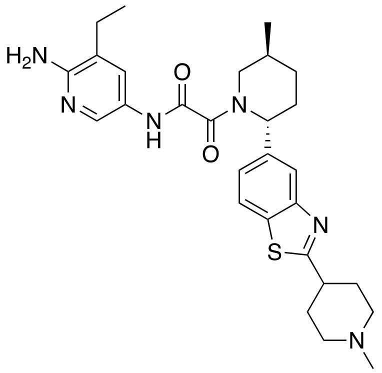

N-(6-amino-5-ethylpyridin-3-yl)-2-((2*R*,5*S*)-5-methyl-2-(2-(1-methylpiperidin-4-yl)benzo[d]thiazol-5-yl)piperidin-1-yl)-2-oxoacetamide (**TNG462**). **88** (53.0 g, 129 mmol, 1.00 eq) and **92** (42.7 g, 129 mmol, 1.00 eq) were stirred in DMF （250 mL）. TEA (39.3 g, 388 mmol, 54.1 mL, 3.00 eq) was added at 0 °C, followed by HATU (49.3 g, 129 mmol, 1.00 eq). The reaction was stirred for 6 h at 25 °C. The reaction was then poured into H_2O (500 mL) and extracted with ethyl acetate (500 mL x 3). The combined organic layer was washed with brine (200 mL x 2) and the organic layer was concentrated. The residue was purified by column chromatography (SiO_2, petroleum ether / ethyl acetate = 1 / 1 to 0 / 1) to afford *tert*-butyl (*tert*-butoxycarbonyl)(3-ethyl-5-(2-((2*R*,5*S*)-5-methyl-2-(2-(1-methylpiperidin-4-yl)benzo[*d*]thiazol-5-yl)piperidin-1-yl)-2-oxoacetamido)pyridin-2-yl)carbamate (60.0 g, 83.2 mmol, 64 % yield) as a brown solid. This was then stirred in MeOH (60 mL) and 4 N HCl / MeOH (600 mL) was added, and the reaction was stirred at 20 – 25 °C for 5 h. The solvent was then removed under reduce pressure and the residue was dissolved with H_2O (300 mL) and saturated Na_2CO_3 was added to bring the solution to pH = 8 ~ 9. The aqueous was then extracted with ethyl acetate (200 mL x 3) and the combined organic layer was concentrated. The crude product was purified by reversed-phase HPLC (Waters SFC350 preparative SFC; Column: DAICEL CHIRALPAK IC (250 mm x 50 mm, 10 μm); Mobile phase: A: CO_2 and B: MeOH (0.1% NH_3•H_2O) ：ACN, 1:1, (v/v); Gradient: B% = 60 % isocratic elution mode; Flow rate: 200 mL / min; Wavelength: 220 nm; Column temperature: 40 °C; System back pressure: 100 bar) to afford **TNG462** (13.4 g, 25.1 mmol, 30% yield, 97.6% purity) as a white solid. ^{1}H NMR (400 MHz $CDCl_3$) δ 9.23 - 9.37 (m, 1H), 8.03 - 8.17 (m, 1H), 7.92 (br s, 1H), 7.83 (br d, *J* = 7.94 Hz, 1H), 7.71 (br s, 1H), 7.28 - 7.38 (m, 1H), 6.56 (br s, 1H), 5.92 (br s, 1H), 4.77 (br d, *J* = 13.23 Hz, 1H), 4.47 (br d, *J* = 11.47 Hz, 2H), 4.19 - 4.30 (m, 1H), 3.39 (br d, *J* = 12.57 Hz, 1H), 2.90 - 3.14 (m, 3H), 2.13 - 2.53 (m, 14H), 1.84 - 2.06 (m, 5 H), 1.42 (br s, 1H), 1.17 - 1.32 (m, 3H), 1.05 - 1.14 (m, 3H). Melting point of crystalline material = 145.8 °C. [α]21D = +158.68° (c=0.121 g/100 mL, EtOH). LCMS (ESI): [M + H]+ m/z calcd 521.27; found 521.2. Rt = 1.784 min. HRMS (ESI, + vw ion): m/z calcd for $C_{28}H_{37}N_6O_2S$ [M + H] 521.2693; found 521.2697.

NMR SPECTRA AND HPLC/LCMS TRACES OF FINAL COMPOUNDS

COMPOUND 1

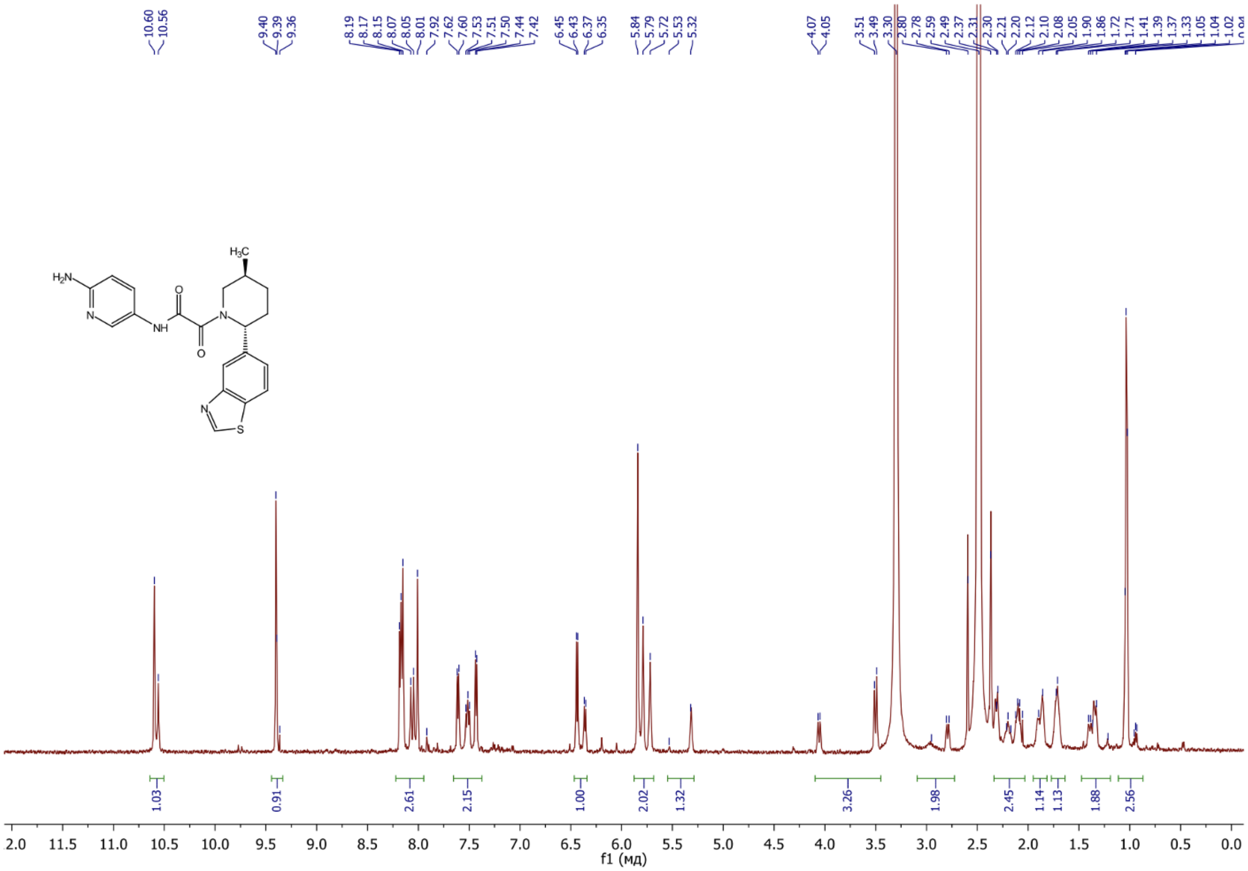
10.60
10.56
9.40
9.39
9.36
8.19
8.17
8.15
8.07
8.05
8.01
7.92
7.62
7.60
7.53
7.51
7.50
7.44
7.42
6.45
6.43
6.37
6.35
5.84
5.79
5.72
5.53
5.32
4.07
4.05
3.51
3.49
3.30
2.80
2.78
2.59
2.49
2.37
2.31
2.30
2.21
2.20
2.12
2.10
2.08
2.05
1.90
1.86
1.72
1.71
1.41
1.39
1.37
1.33
1.05
1.04
1.02
H2N
N
NH
O
O
N
H3C
N
S
1.03
0.91
2.61
2.15
1.00
2.02
1.32
3.26
1.98
2.45
1.14
1.13
1.88
2.56
f1 (мд)

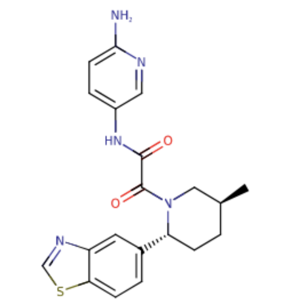

Mol Wt 395.48

Exact Mass 395.16

#	Time	Area%
1	2.084	100.00

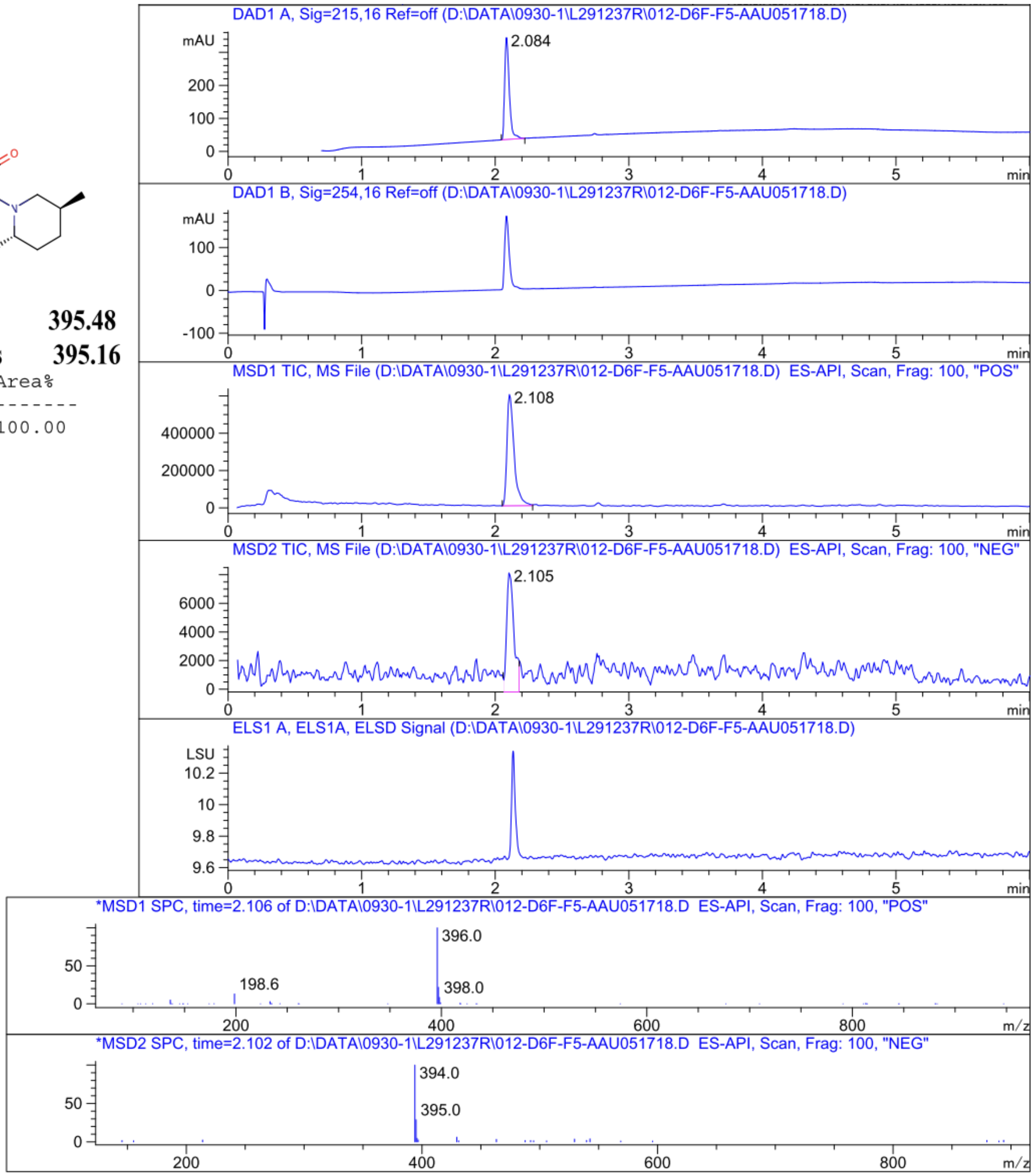

RT 2.108

RT 2.105

COMPOUND 2

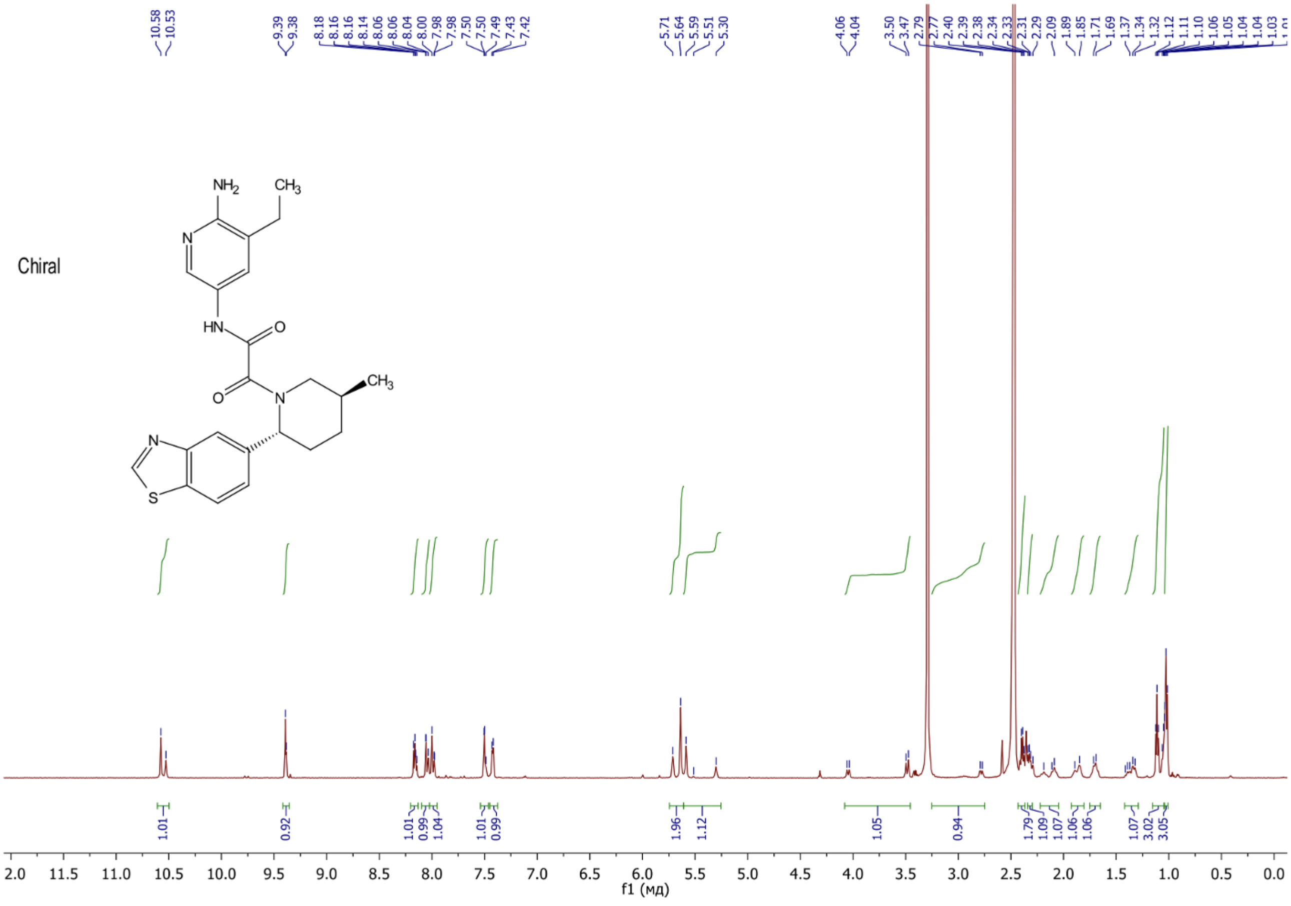

```
MaxPeak:  100.00%
Ret_Time: 2.161 min
```

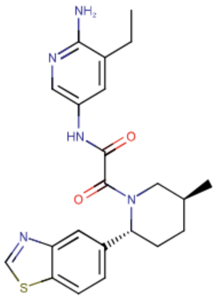

Mol Wt 423.53

Exact Mass 423.2

#	Time	Area%
1	2.161	100.00

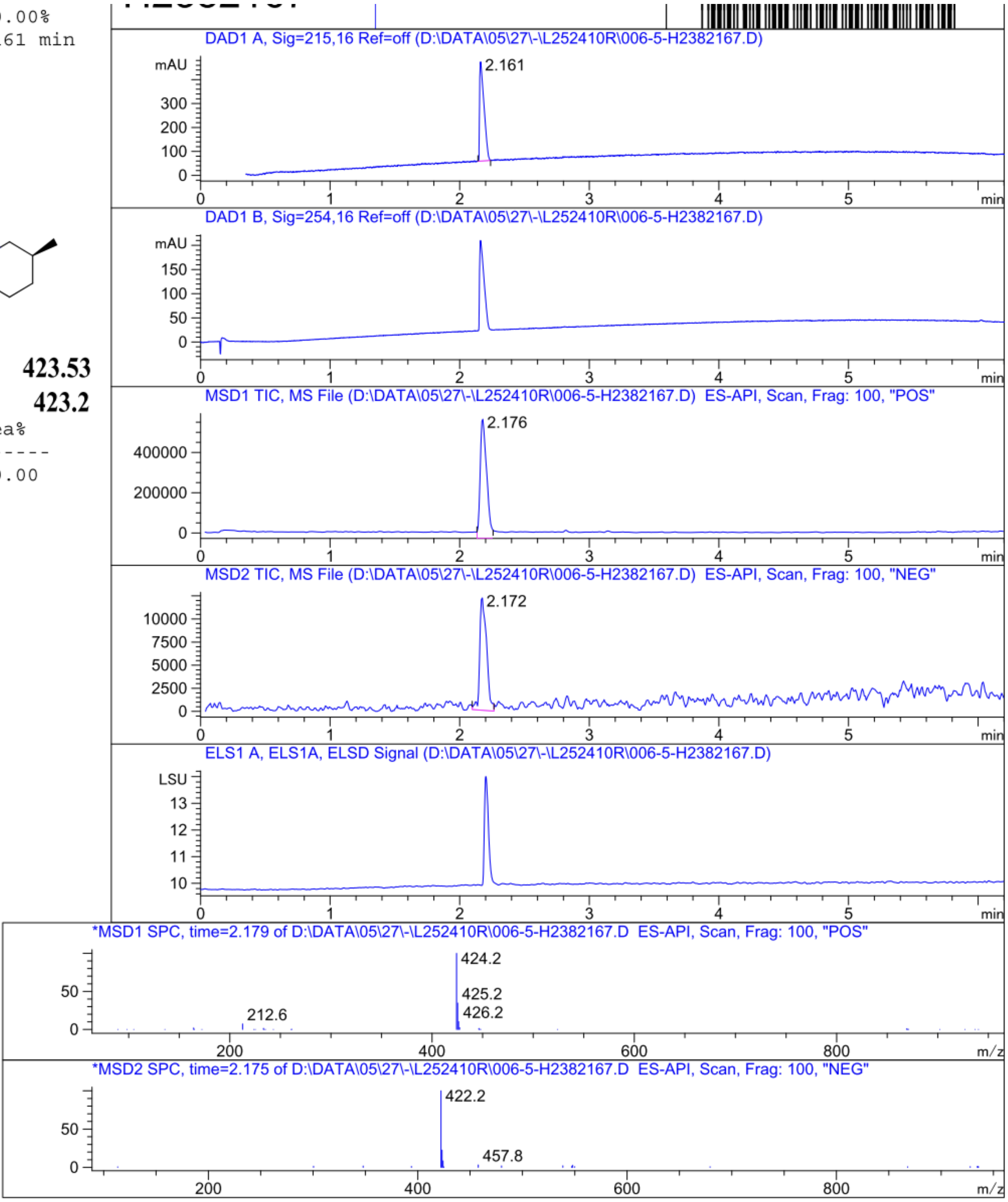

RT 2.176

RT 2.172

COMPOUND 3

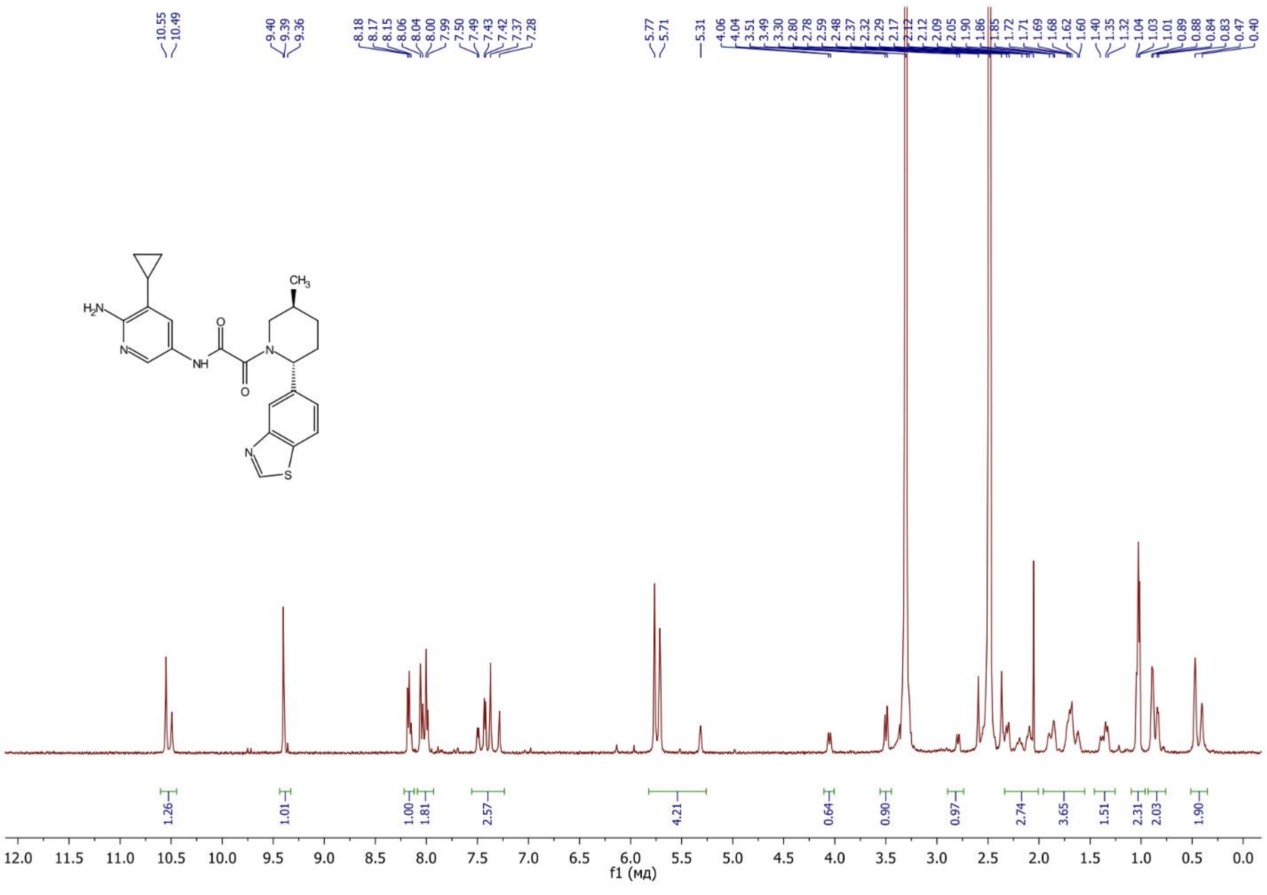

H2N
N
NH
O
O
N
CH3
N
S
12.0
11.5
11.0
10.5
10.0
9.5
9.0
8.5
8.0
7.5
7.0
6.5
6.0
5.5
5.0
4.5
4.0
3.5
3.0
2.5
2.0
1.5
1.0
0.5
0.0
f1 (мд)

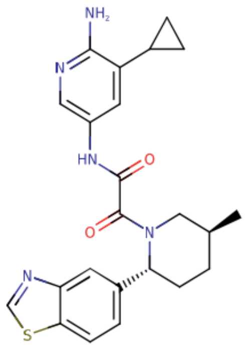

Mol Wt 435.54
Exact Mass 435.2

```
#  Time   Area%
-----------------
1  2.536  100.00
```

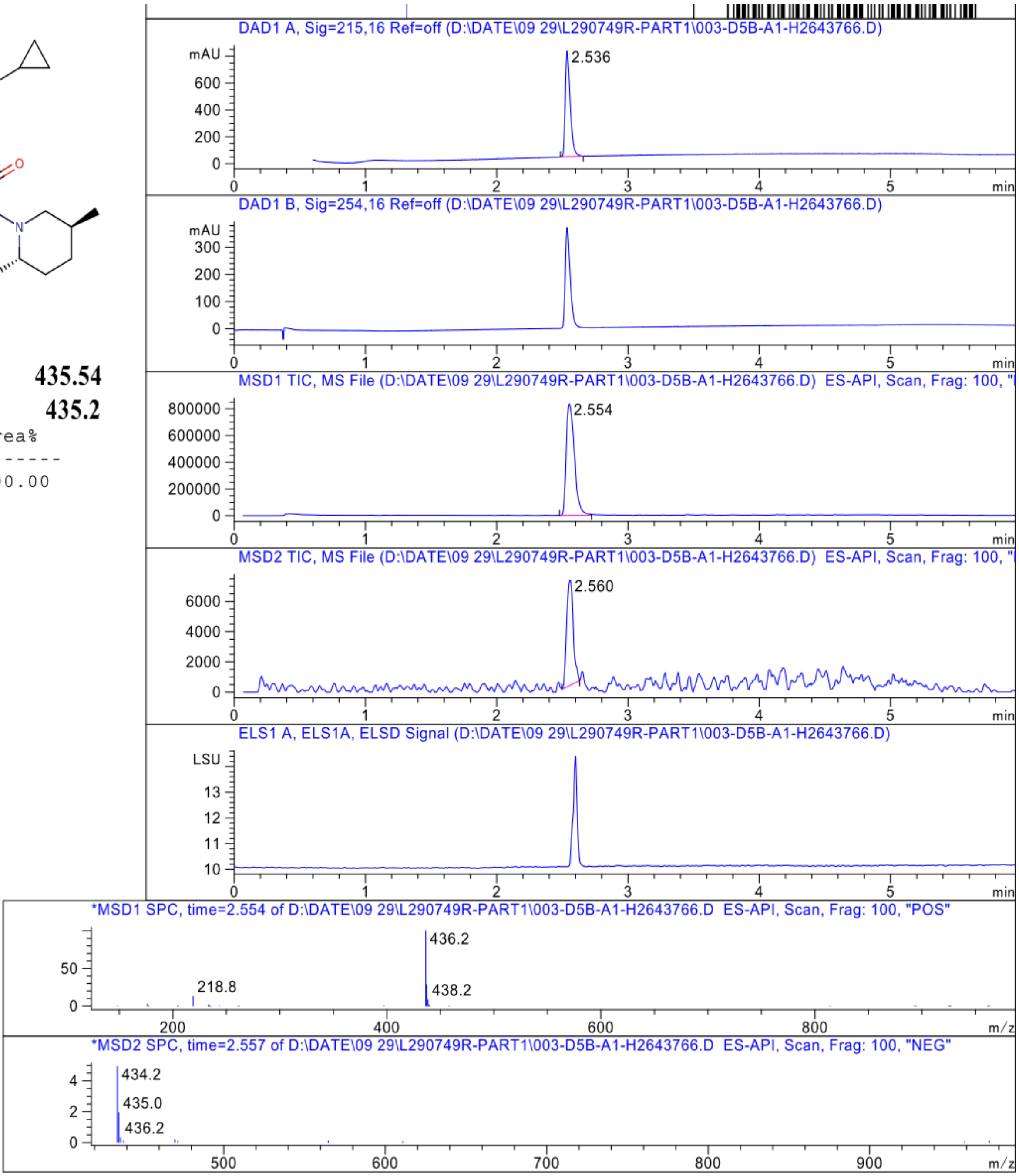

COMPOUND 4

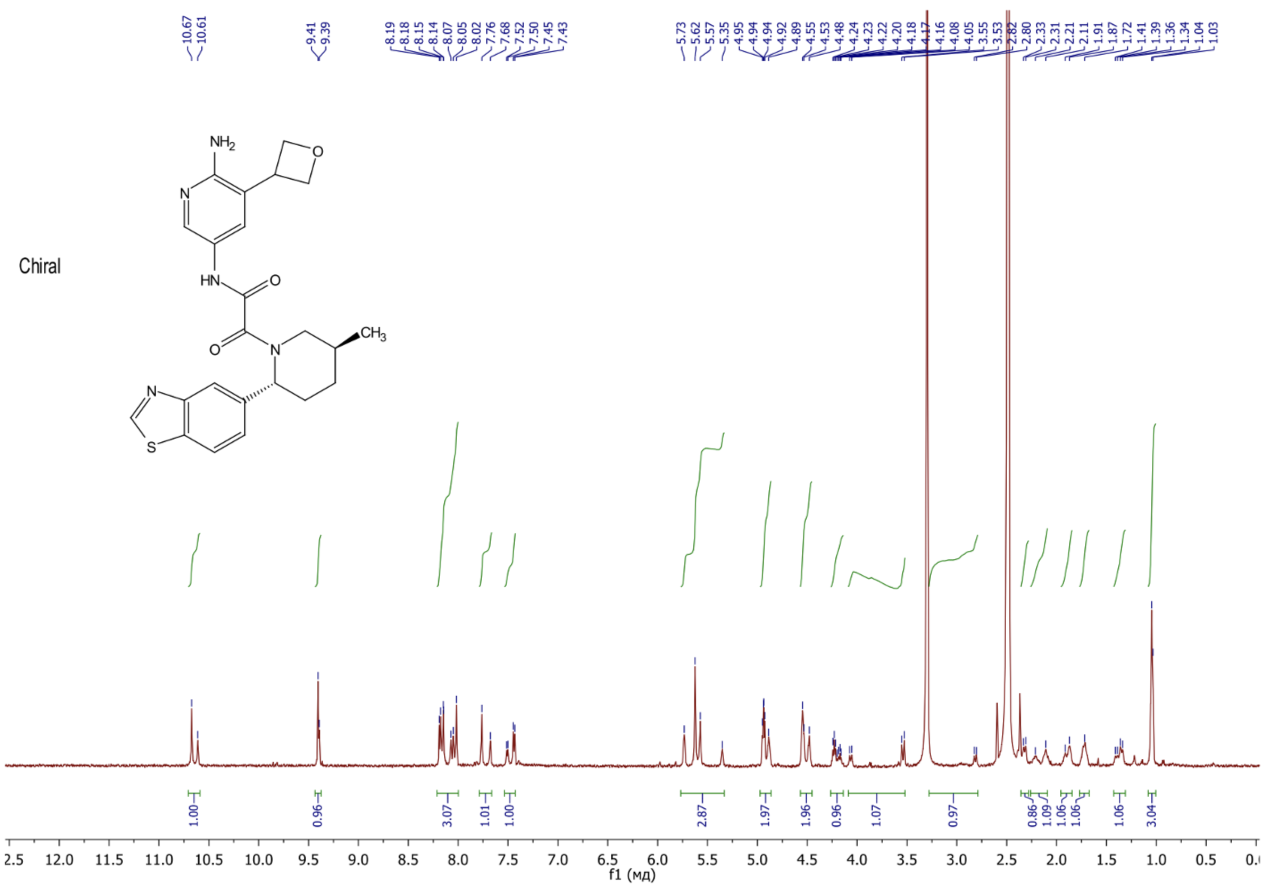

MaxPeak: 96.99%
Ret_Time: 2.522 min

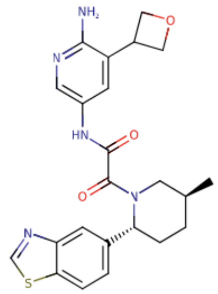

Mol Wt 451.54

Exact Mass 451.19

#	Time	Area%
1	2.522	96.99
2	2.596	3.01

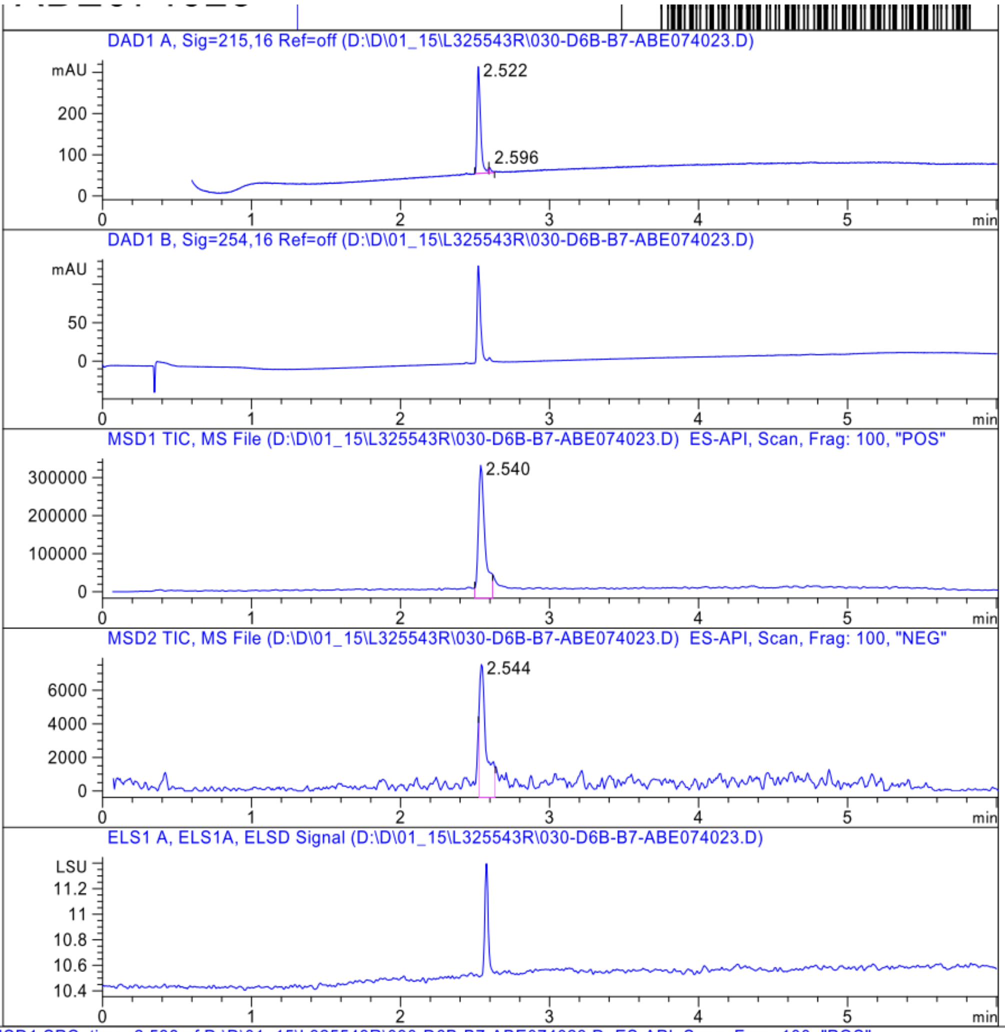

RT 2.540

RT 2.544

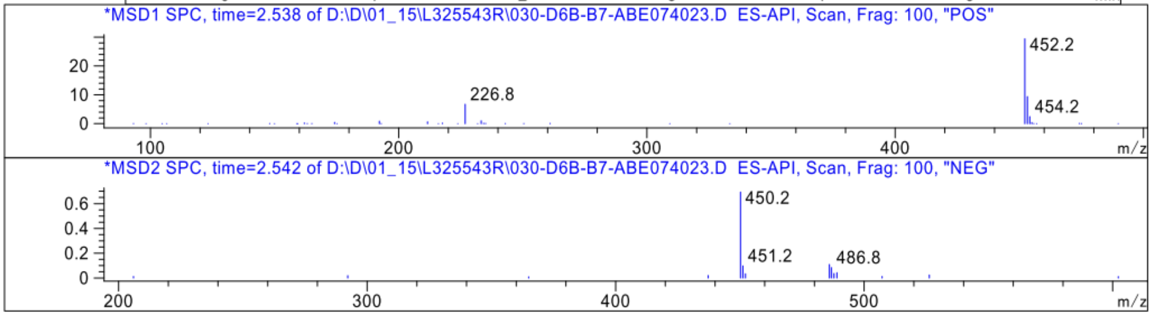

COMPOUND 5

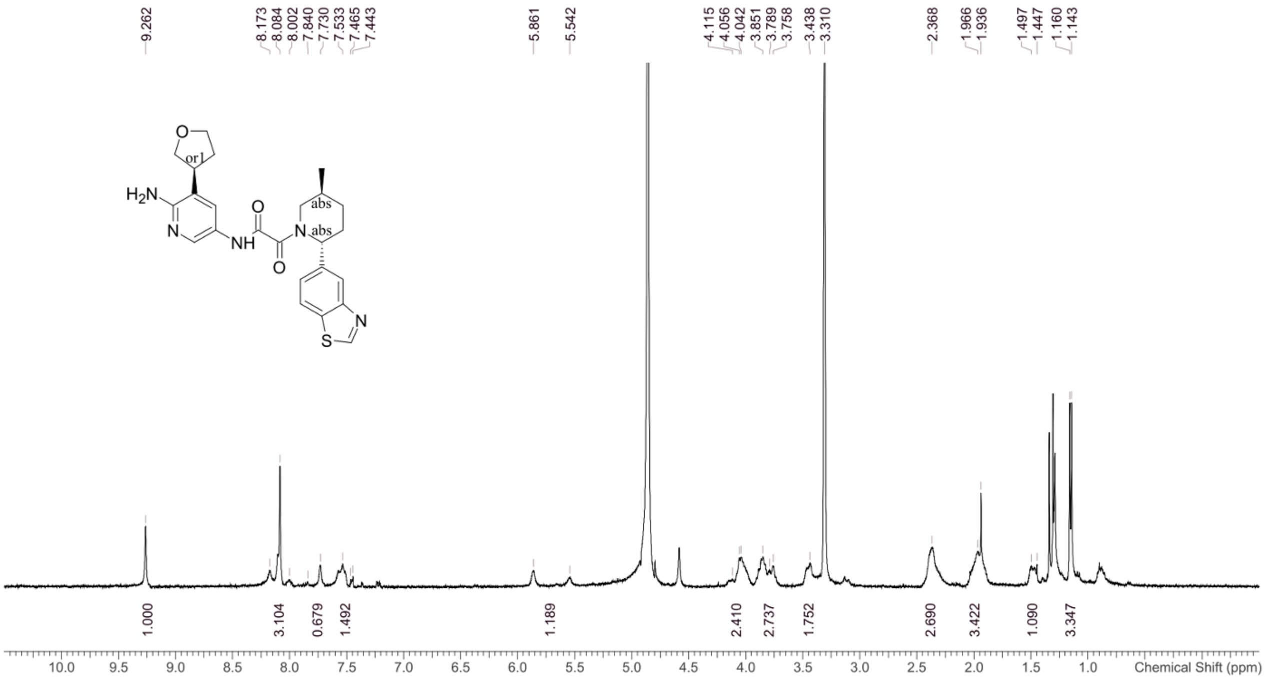
O
or1
H2N
N
NH
O
O
abs
N
abs
N
S
Chemical Shift (ppm)

COMPOUND 6

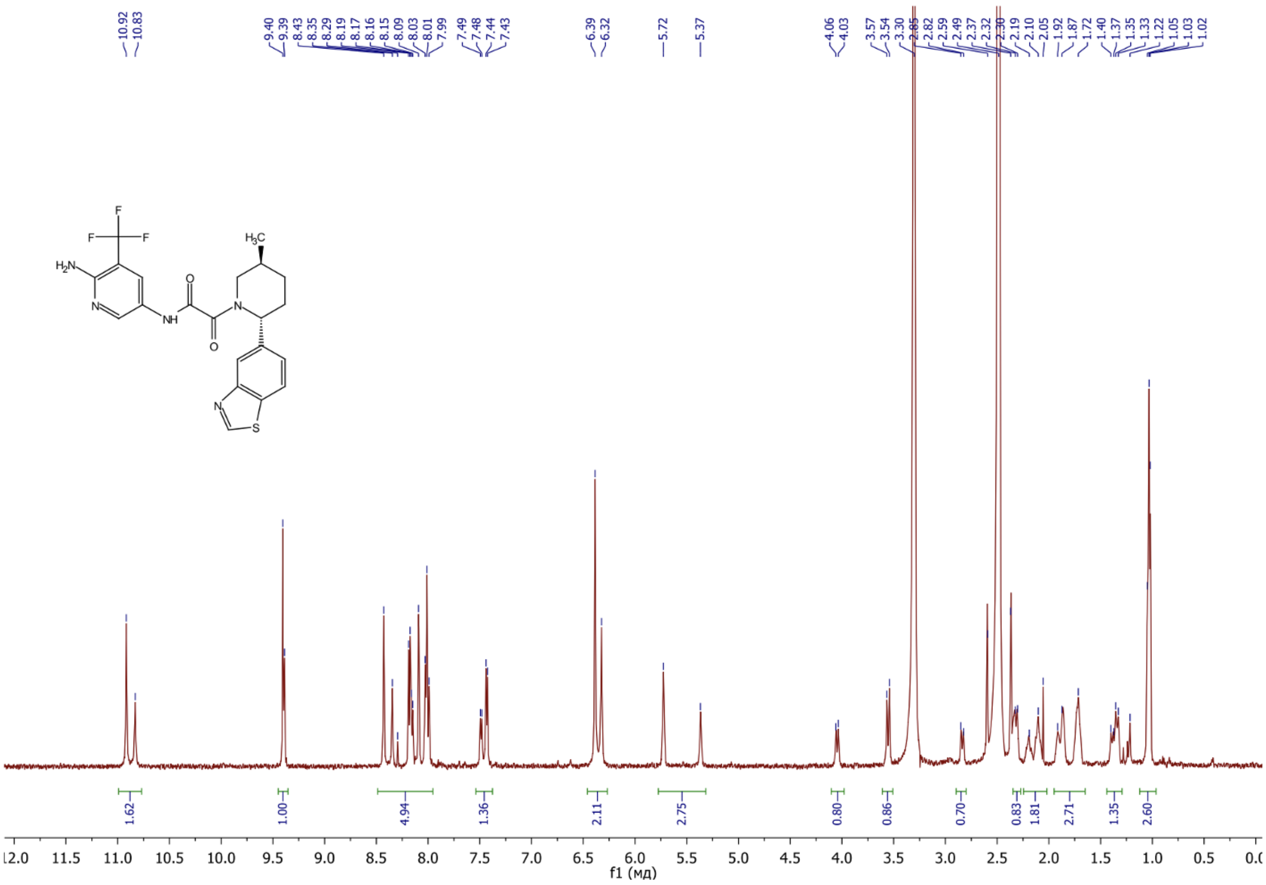
F
F
F
H2N
N
NH
O
O
H3C
N
N
S
f1 (мд)

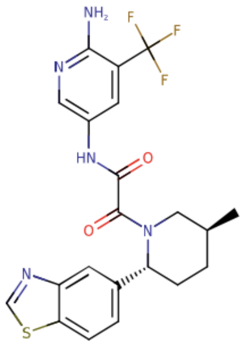

Mol Wt	463.48
Exact Mass	463.15

```
#  Time   Area%
------------------
1  3.482  100.00
```

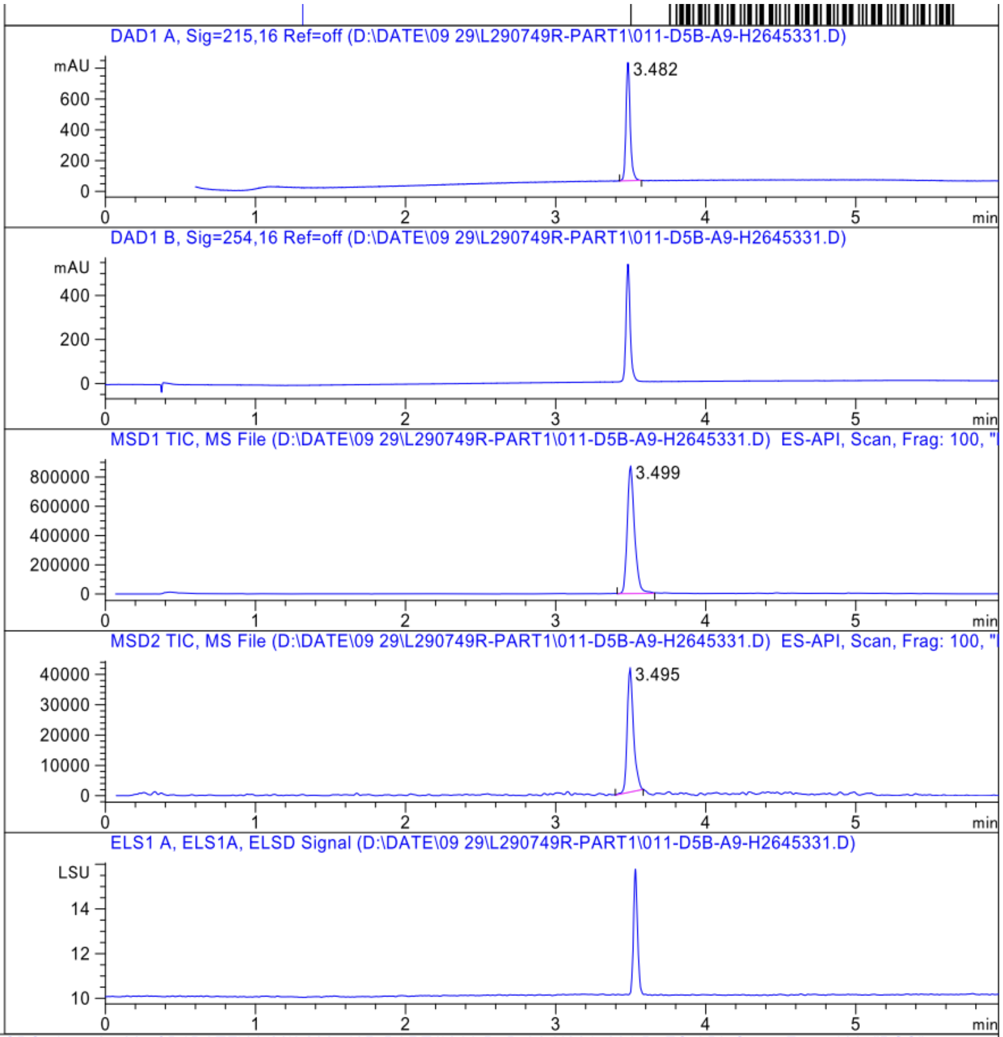

RT 3.499

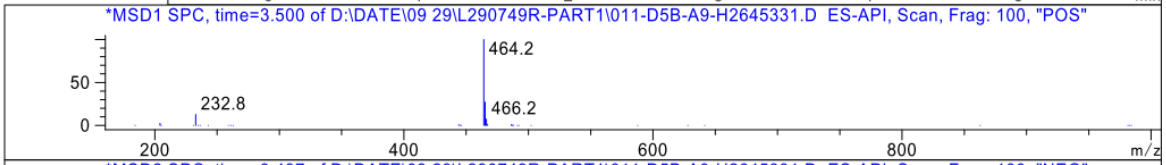

RT 3.495

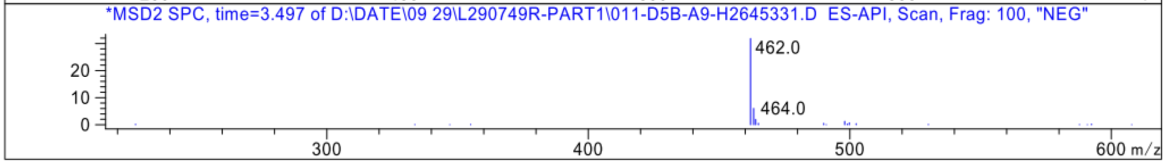

COMPOUND 7

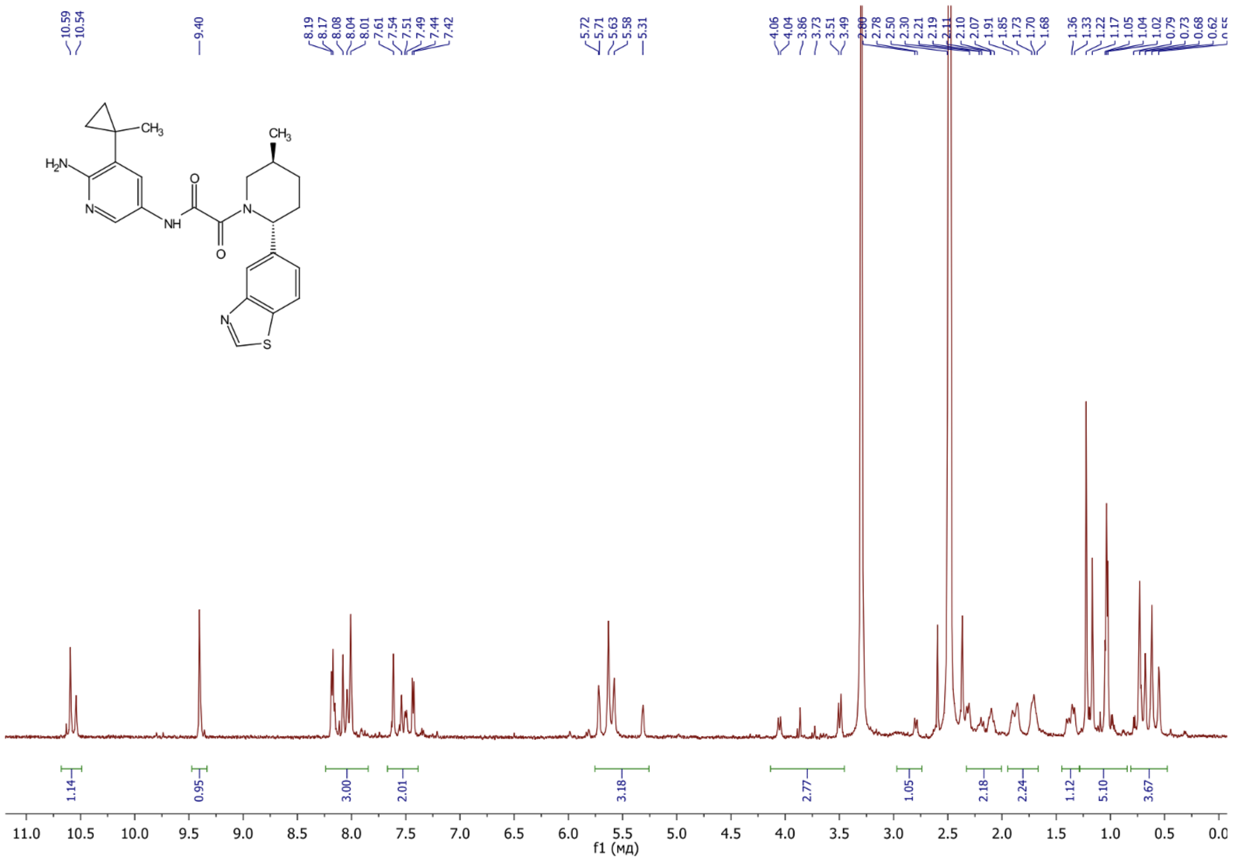
10.59
10.54
9.40
8.19
8.17
8.08
8.04
8.01
7.61
7.54
7.51
7.49
7.44
7.42
5.72
5.71
5.63
5.58
5.31
4.06
4.04
3.86
3.73
3.51
3.49
2.78
2.50
2.30
2.21
2.19
2.11
2.10
2.07
1.91
1.85
1.73
1.70
1.68
1.36
1.33
1.22
1.17
1.05
1.04
1.02
0.79
0.73
0.68
0.62
CH3
H2N
N
NH
O
O
N
CH3
N
S
1.14
0.95
3.00
2.01
3.18
2.77
1.05
2.18
2.24
1.12
5.10
3.67
11.0 10.5 10.0 9.5 9.0 8.5 8.0 7.5 7.0 6.5 6.0 5.5 5.0 4.5 4.0 3.5 3.0 2.5 2.0 1.5 1.0 0.5 0.0
f1 (мд)

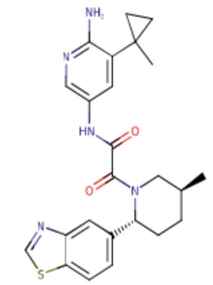

Mol Wt 449.57

Exact Mass 449.22

#	Time	Area%
1	2.428	5.80
2	2.602	81.70
3	2.680	9.08
4	3.129	3.43

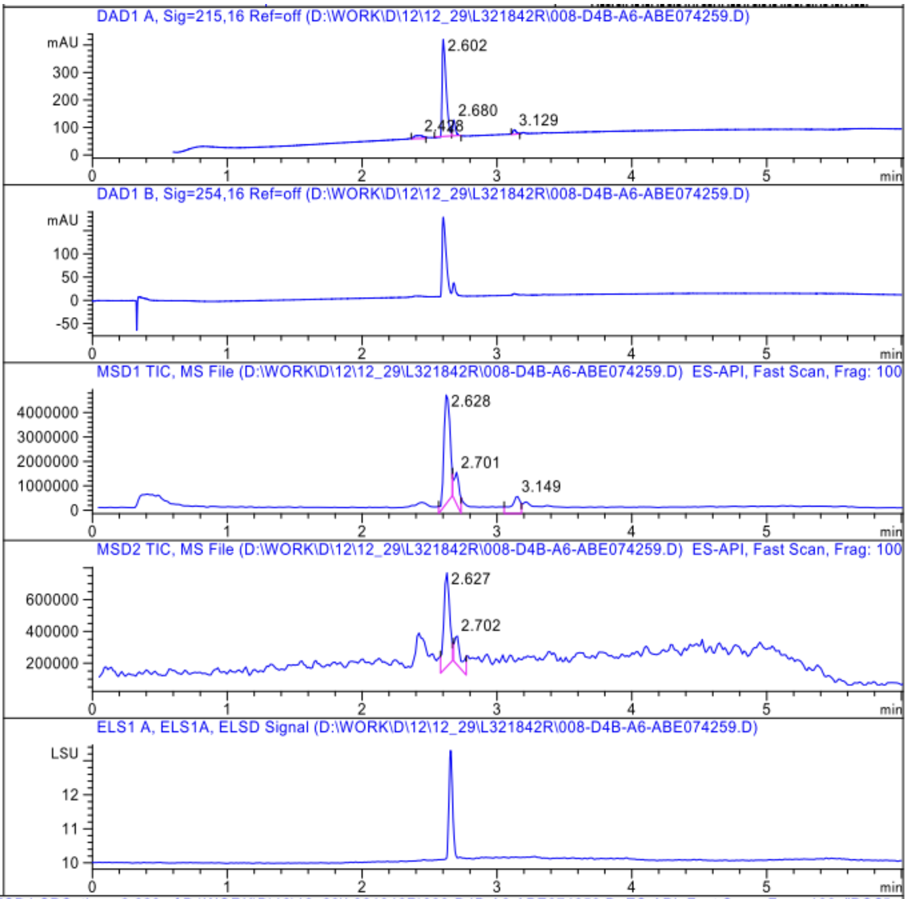

RT 2.628

RT 2.701

RT 3.149

RT 2.627

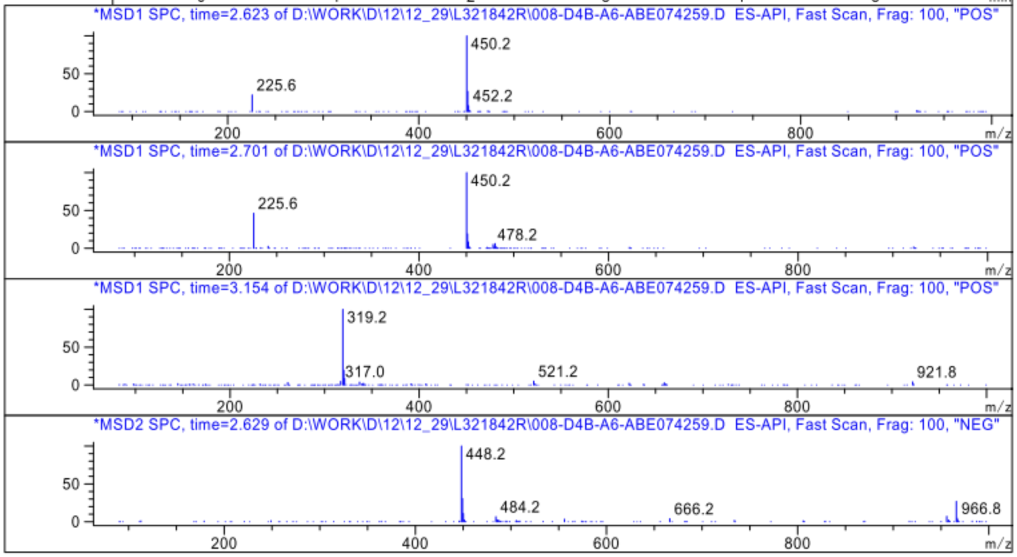

COMPOUND 8

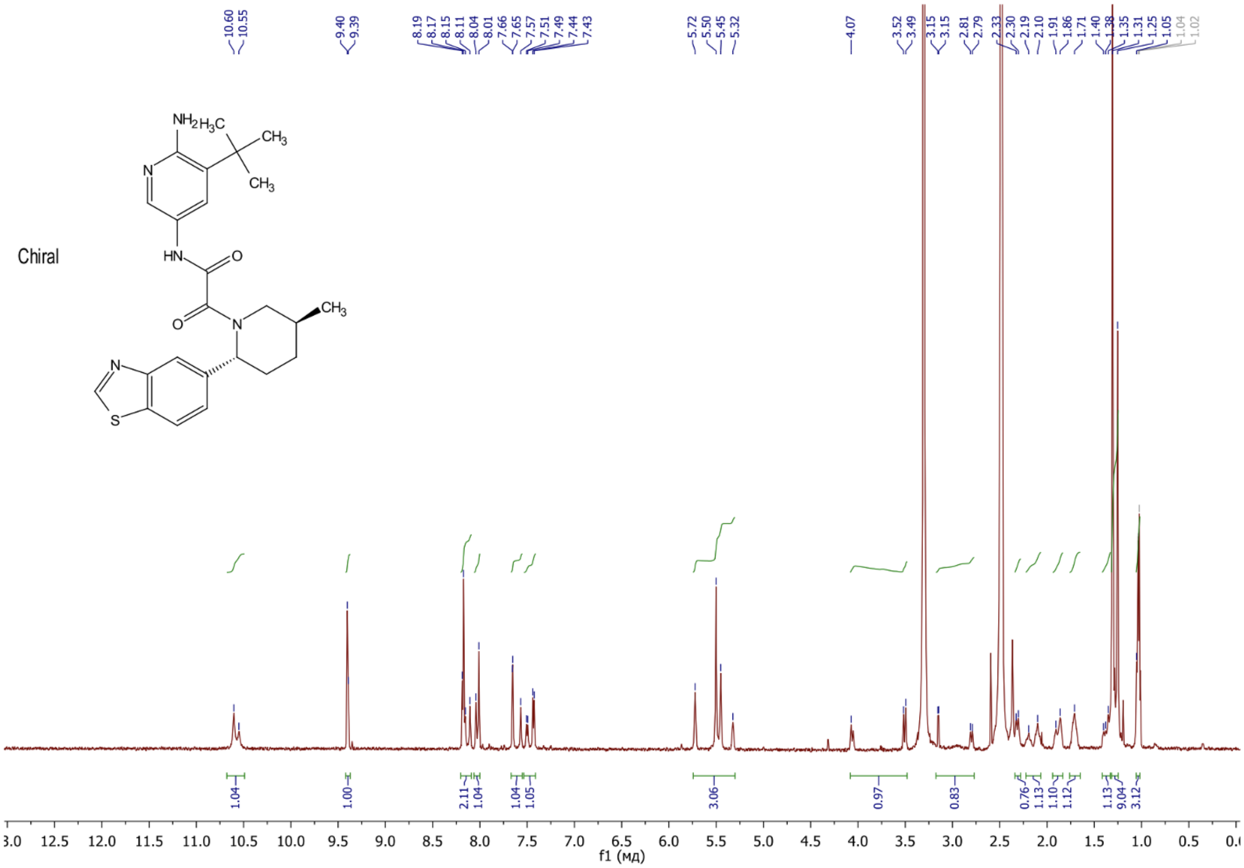
Chiral
NH2
H3C
CH3
CH3
N
HN
O
O
N
CH3
N
S
f1 (мд)

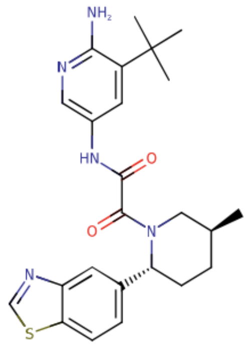

Mol Wt 451.58

Exact Mass 451.24

#	Time	Area%
1	2.371	100.00

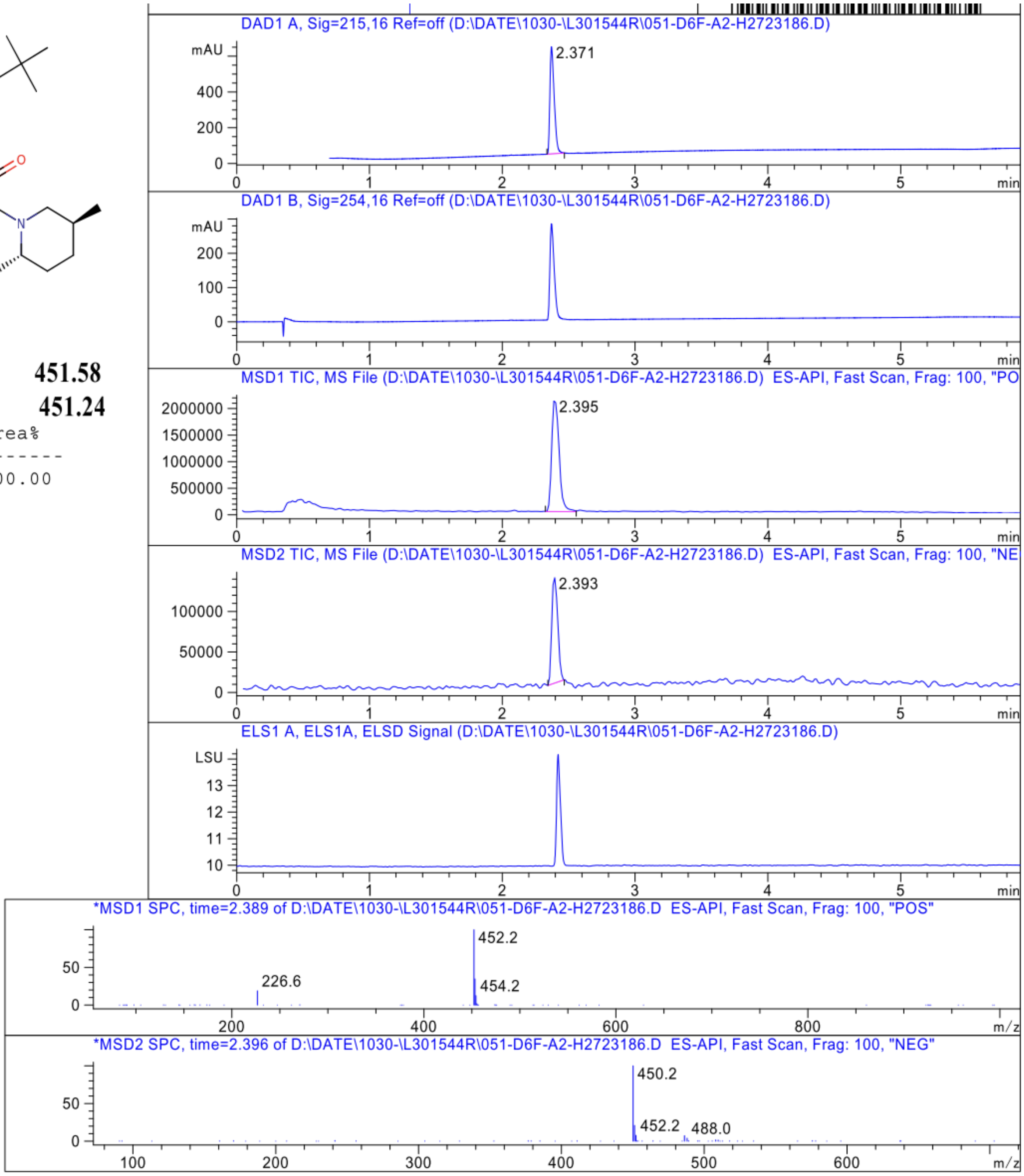

RT 2.395

RT 2.393

COMPOUND 9

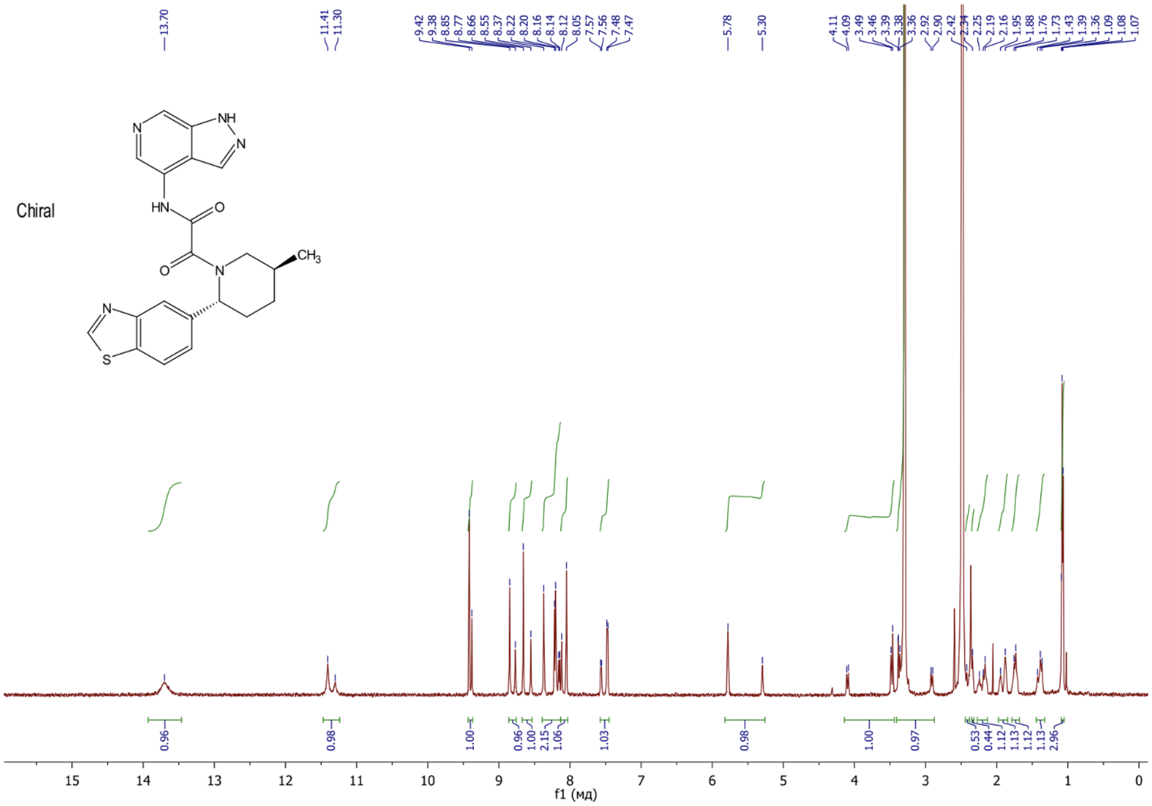
Chiral
HN
O
N
NH
N
CH3
S
f1 (мд)

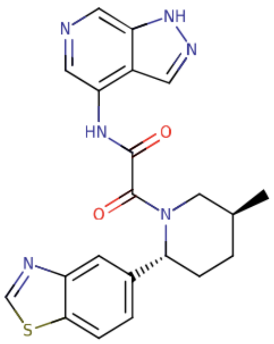

Mol Wt 420.49

Exact Mass 420.15

#	Time	Area%
1	2.374	98.79
2	2.464	1.21

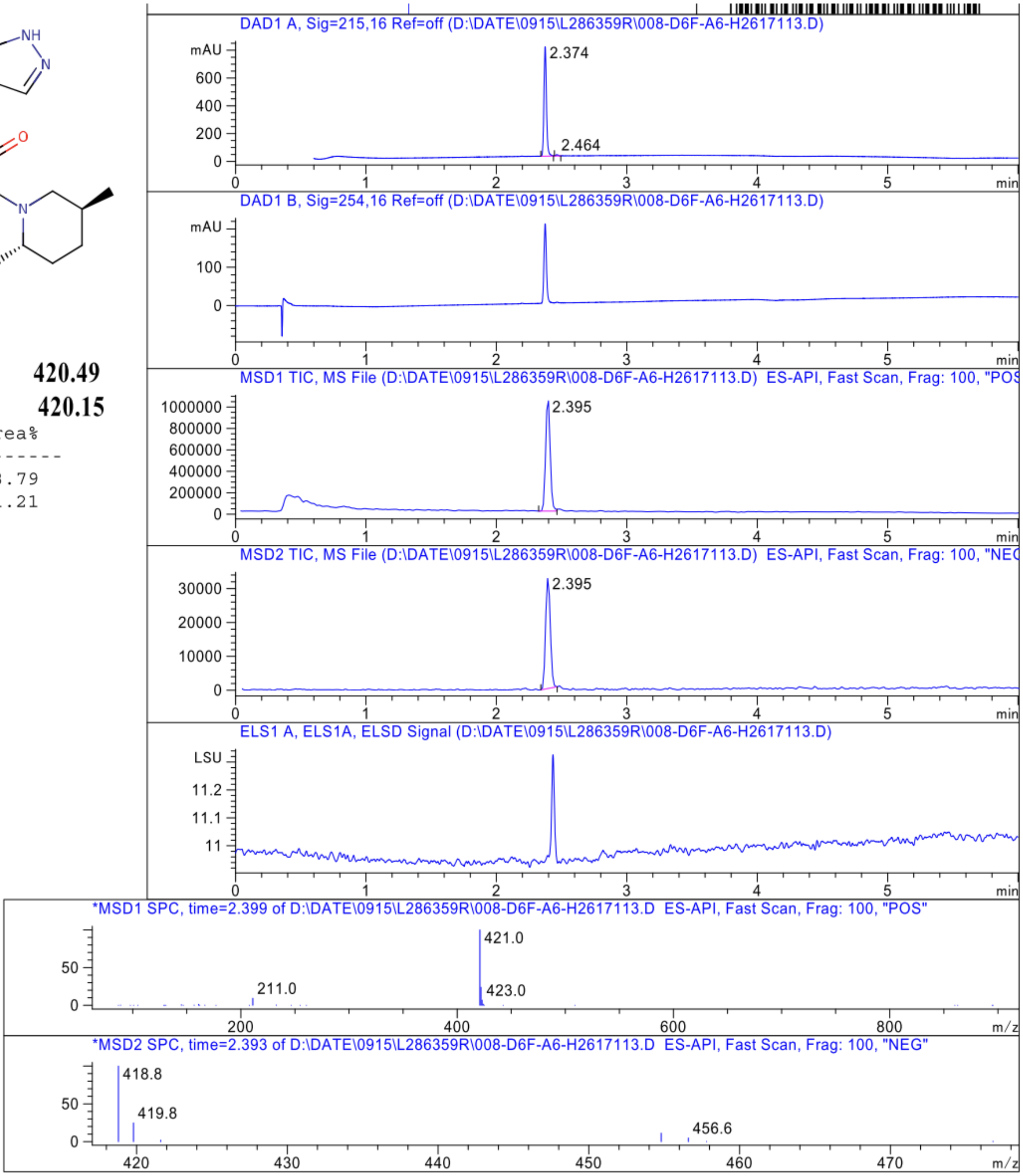

RT 2.395

RT 2.395

COMPOUND 10

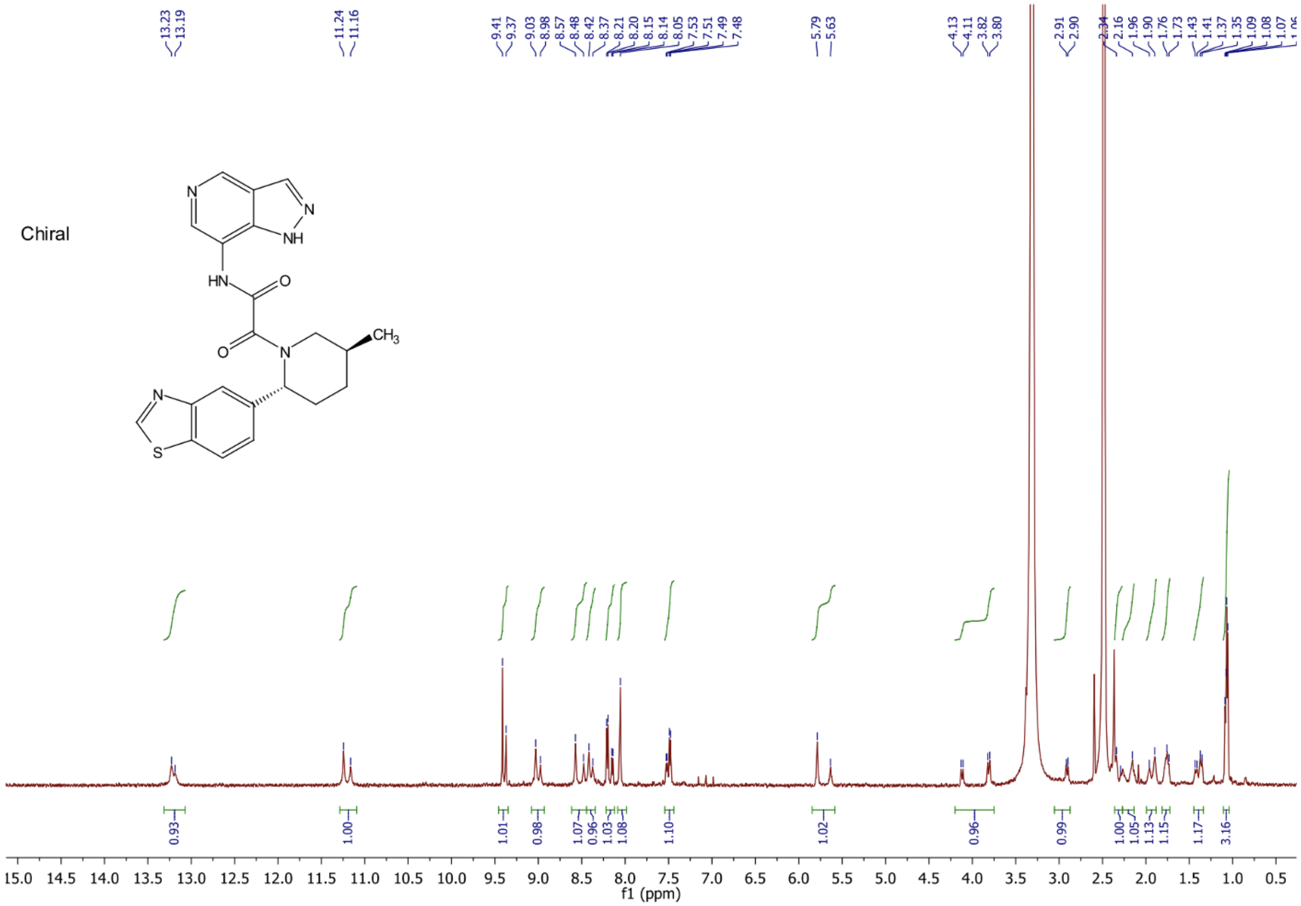
Chiral
f1 (ppm)

MaxPeak: 100.00%
Ret_Time: 2.509 min

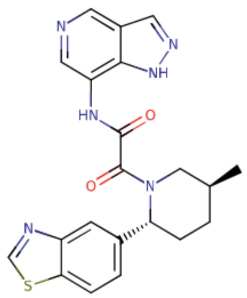

Mol Wt 420.49

Exact Mass 420.15

#	Time	Area%
1	2.509	100.00

RT 2.529

RT 2.526

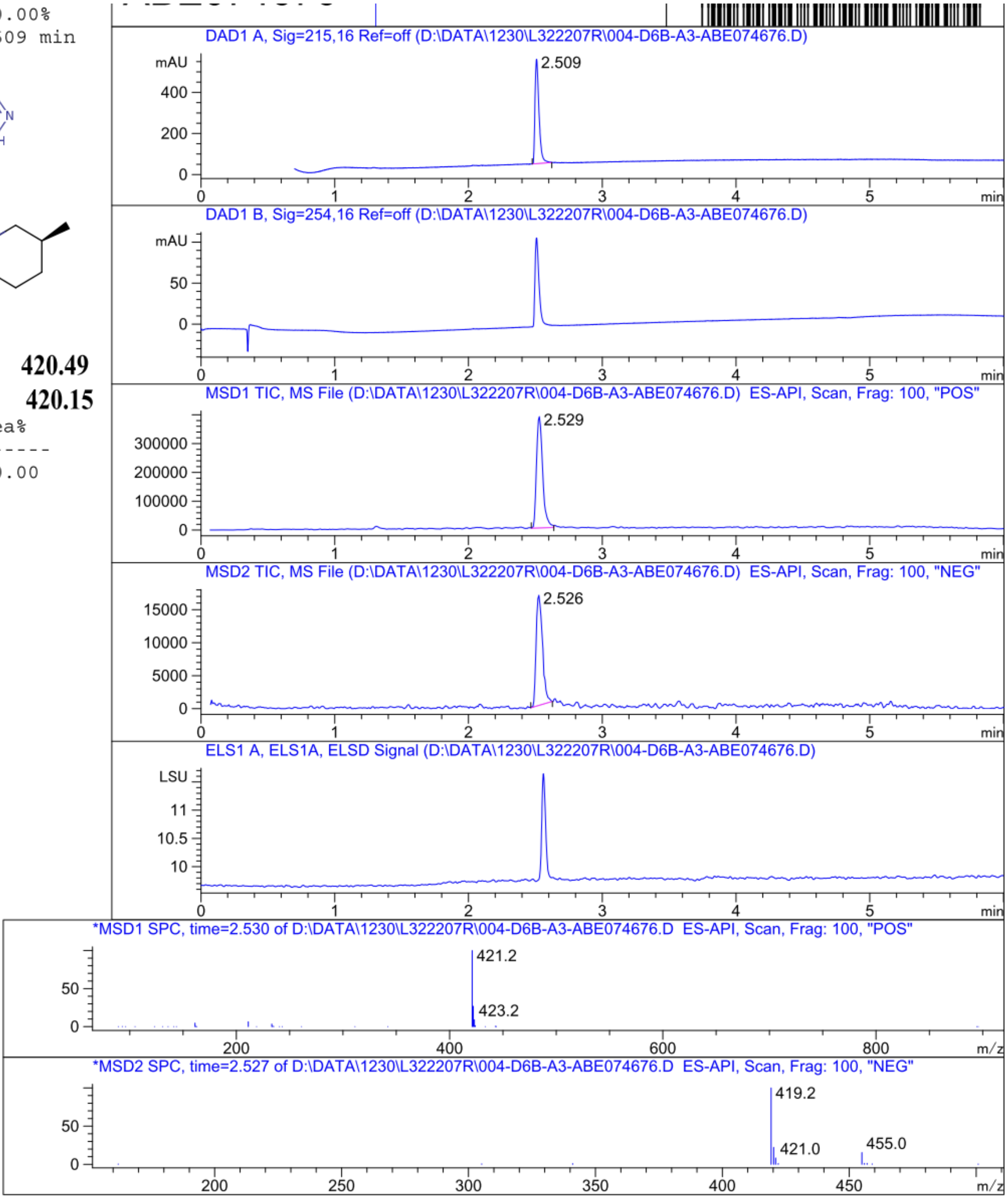

COMPOUND 11

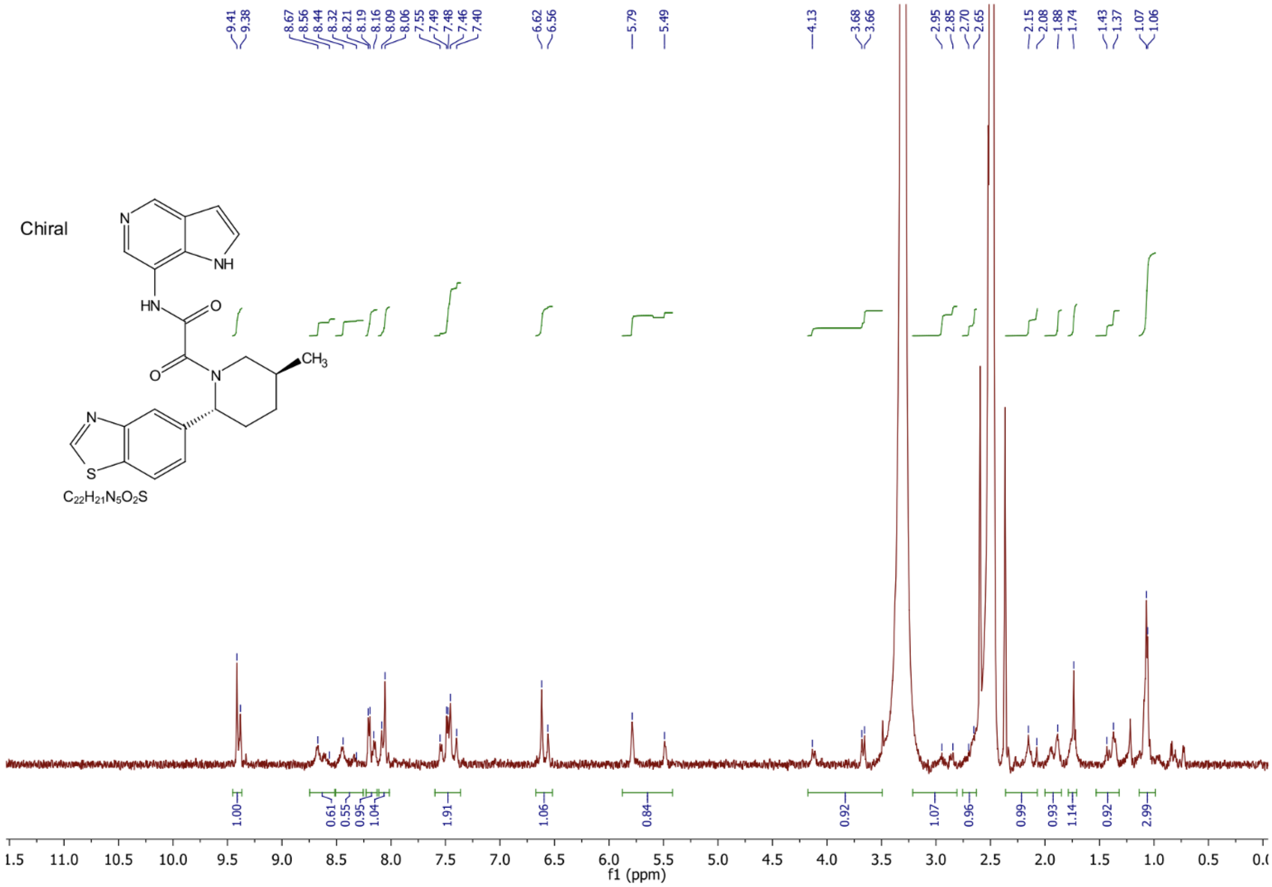
Chiral
C22H21N5O2S
f1 (ppm)

Ret_Time: 2.492 min

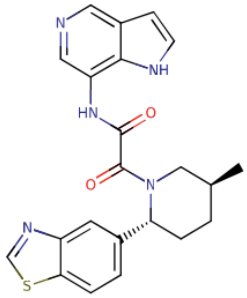

Mol Wt 419.5

Exact Mass 419.16

#	Time	Area%
1	2.492	90.07
2	2.565	9.93

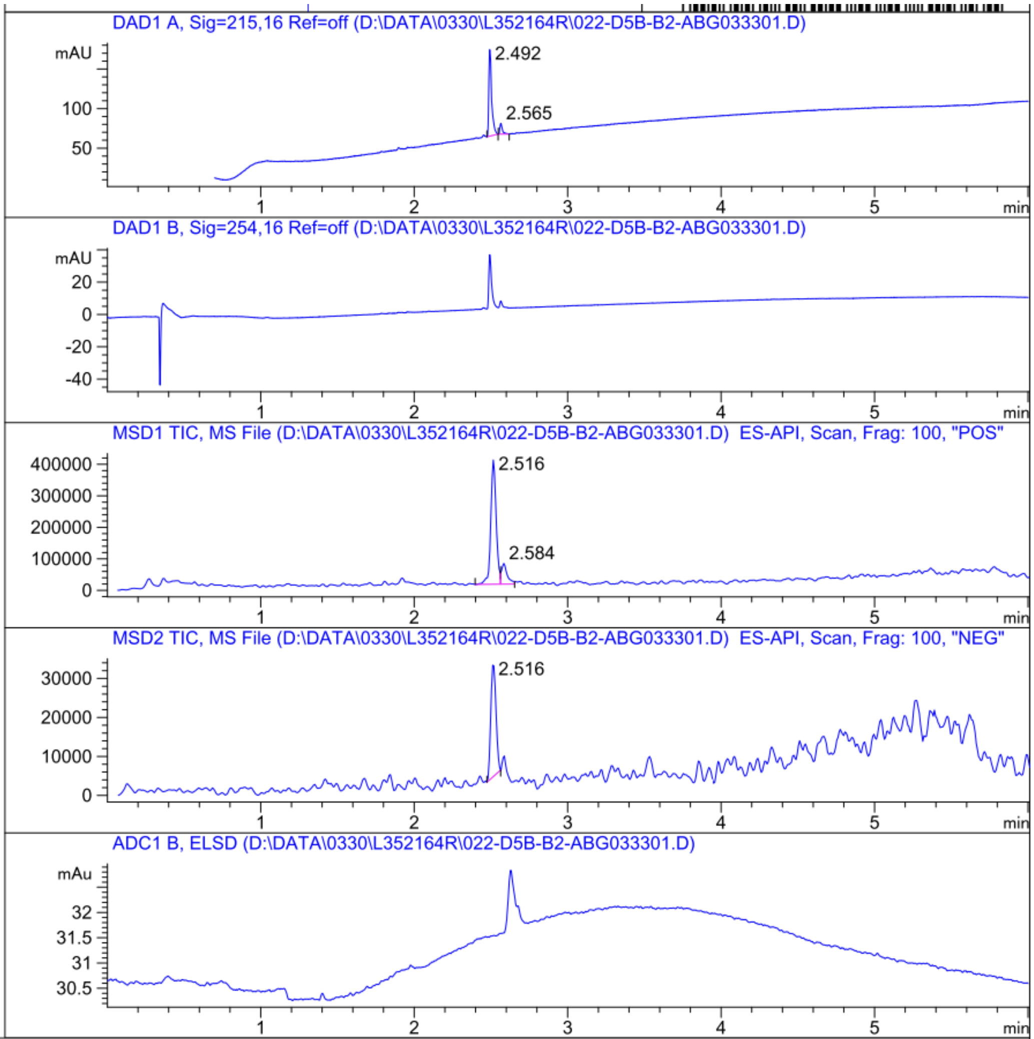

RT 2.516

RT 2.584

RT 2.516

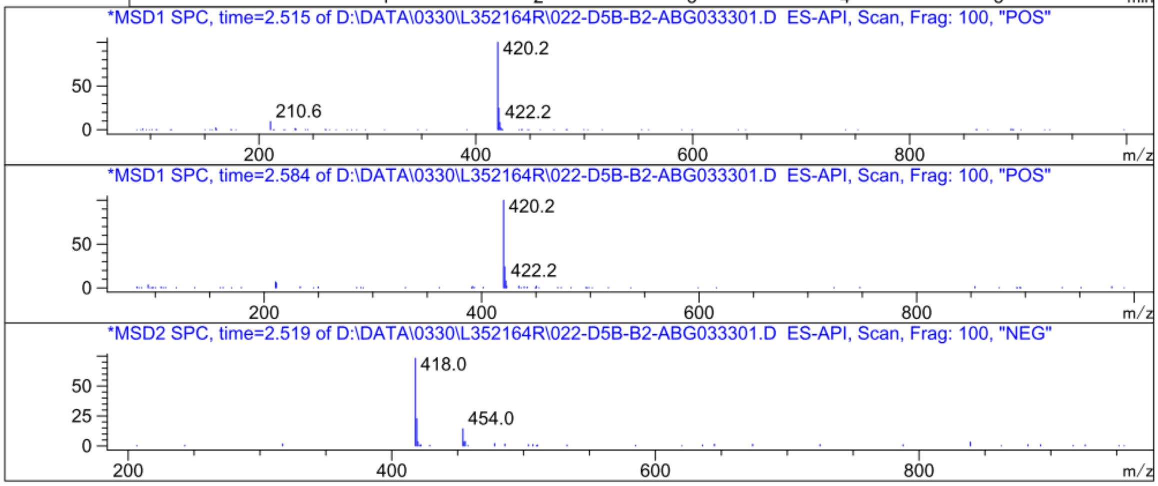

COMPOUND 12

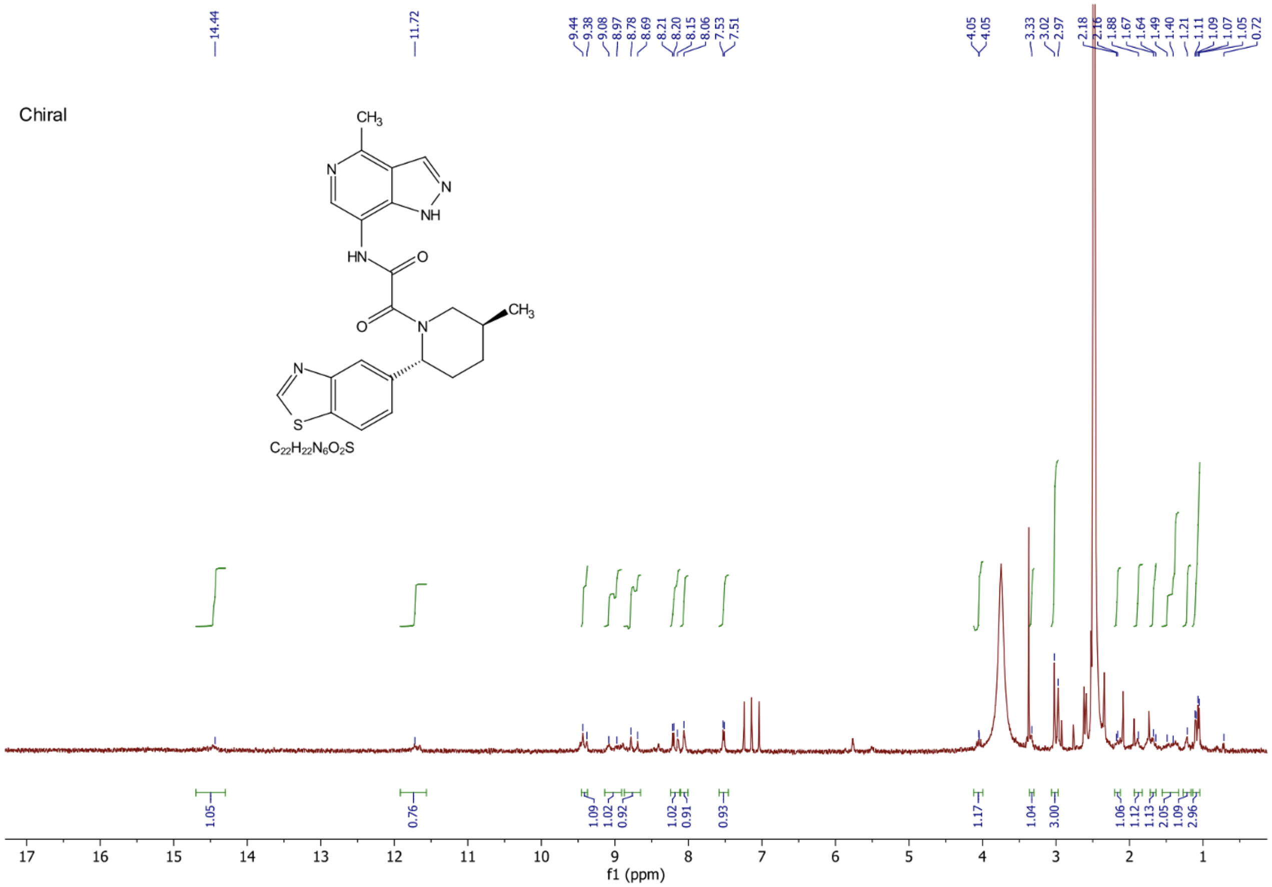

Chiral
$C_{22}H_{22}N_6O_2S$
14.44
11.72
9.44
9.38
9.08
8.97
8.78
8.69
8.21
8.20
8.15
8.06
7.53
7.51
4.05
4.05
3.33
3.02
2.97
2.18
2.16
1.88
1.67
1.64
1.49
1.40
1.21
1.11
1.09
1.07
1.05
0.72
1.05
0.76
1.09
1.02
0.92
1.02
0.91
0.93
1.17
1.04
3.00
1.06
1.12
1.13
2.05
1.09
2.96
f1 (ppm)

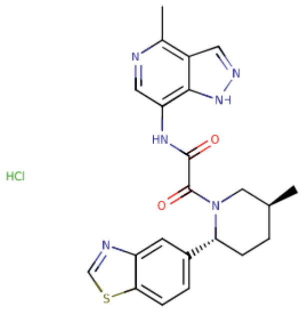

Mol Wt 470.98
Exact Mass 434.17

#	Time	Area%
1	2.575	98.91
2	3.424	1.09

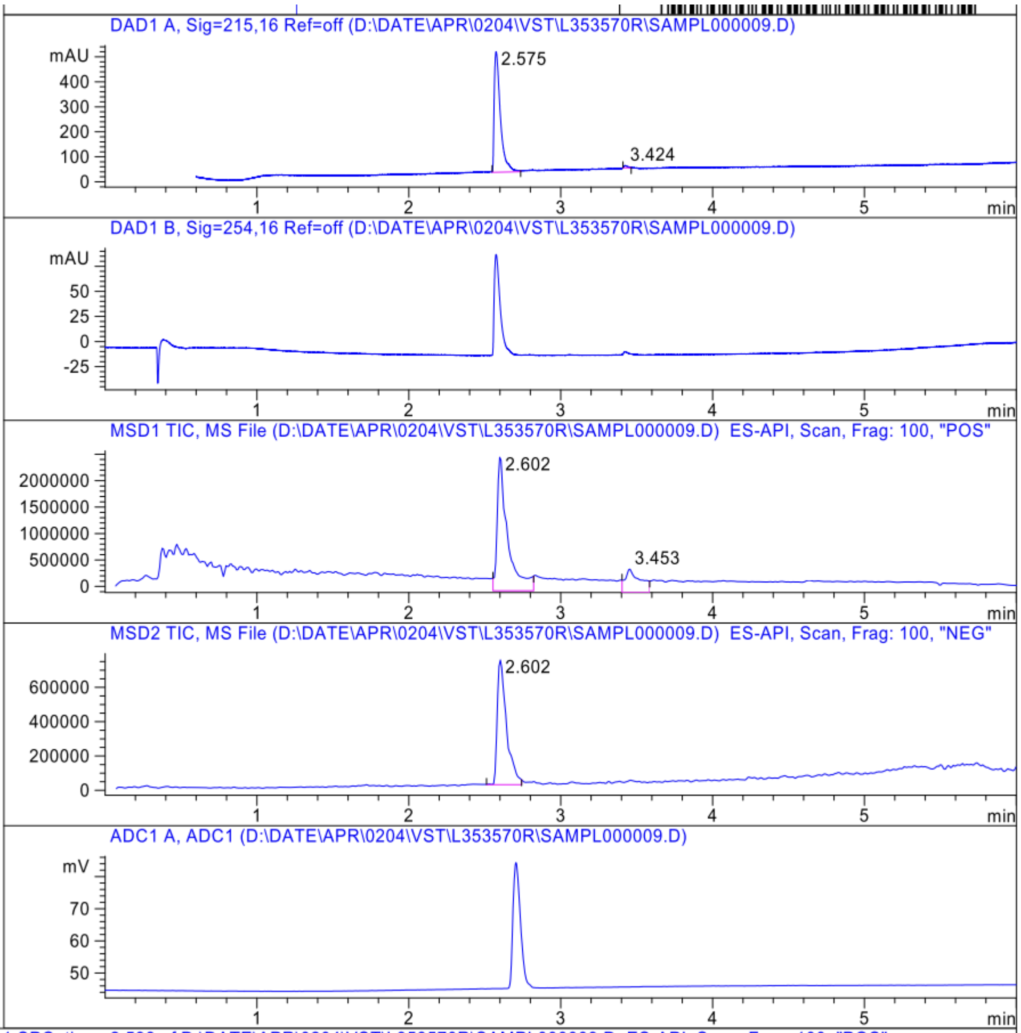

RT 2.602

RT 3.453

RT 2.602

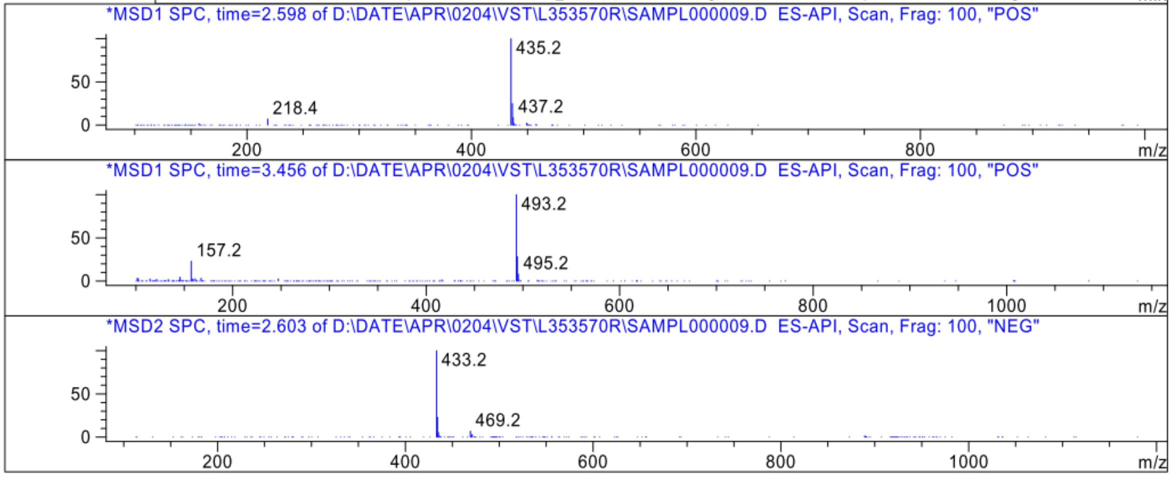

COMPOUND 13

MaxPeak: 94.84%
Ret_Time: 2.320 min

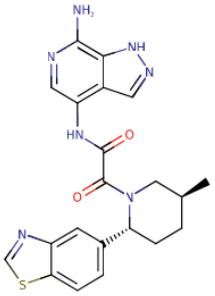

Mol Wt 435.5

Exact Mass 435.16

#	Time	Area%
1	2.320	94.84
2	2.396	3.37
3	2.450	1.79

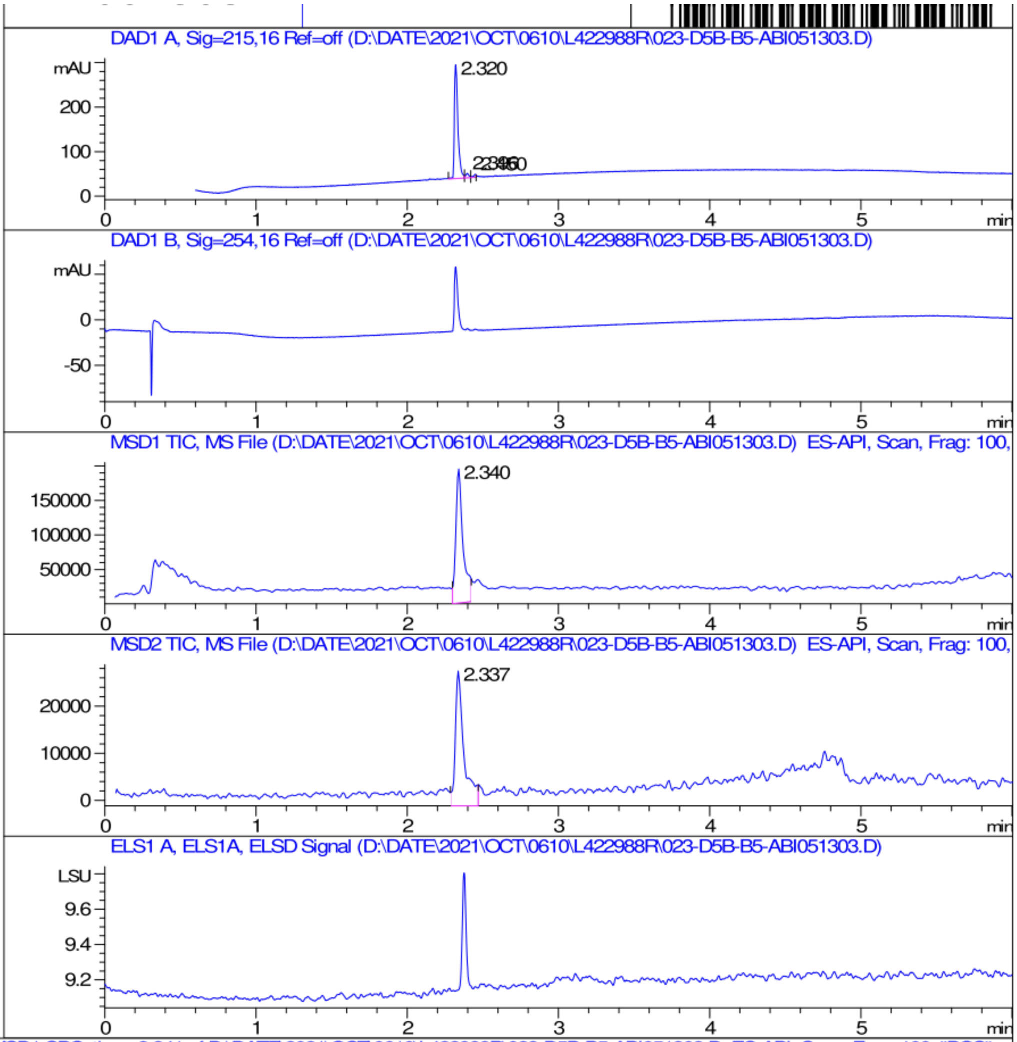

RT 2.340

RT 2.337

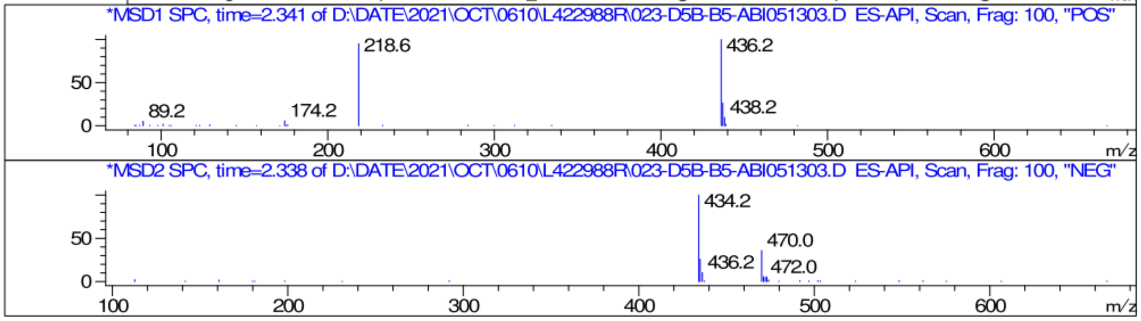

COMPOUND 14

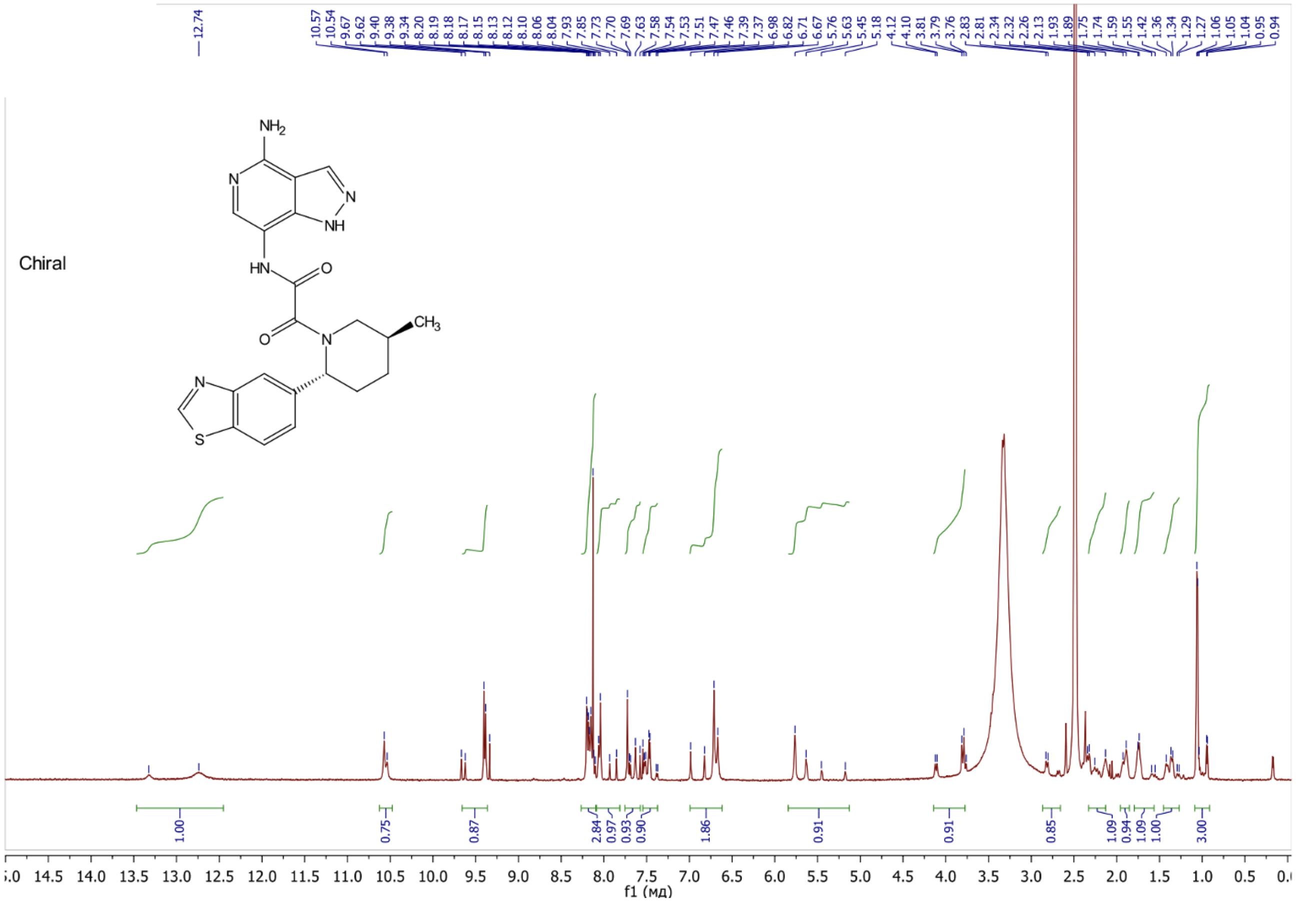
Chiral
f1 (мд)

MaxPeak: 98.01%
Ret_Time: 1.930 min

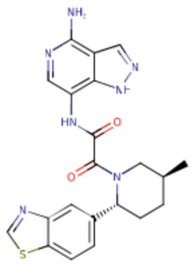

Mol Wt 435.5

Exact Mass 435.16

#	Time	Area%
1	1.487	1.04
2	1.930	98.01
3	2.070	0.95

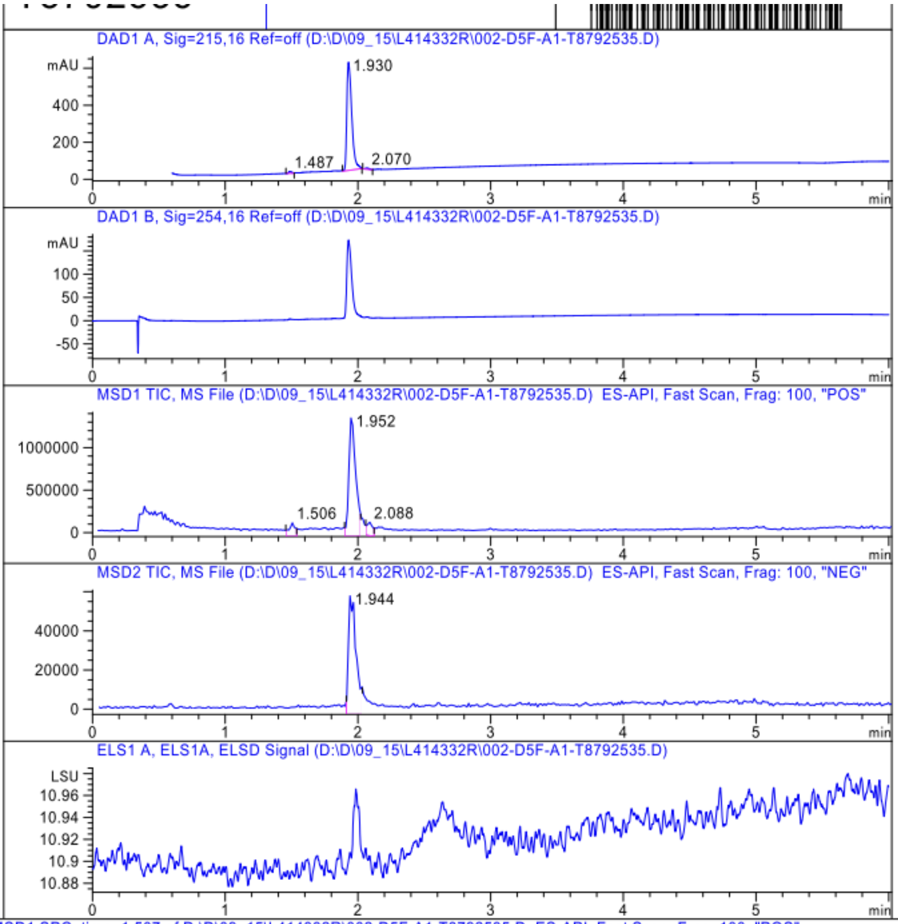

RT 1.506

RT 1.952

RT 2.088

RT 1.944

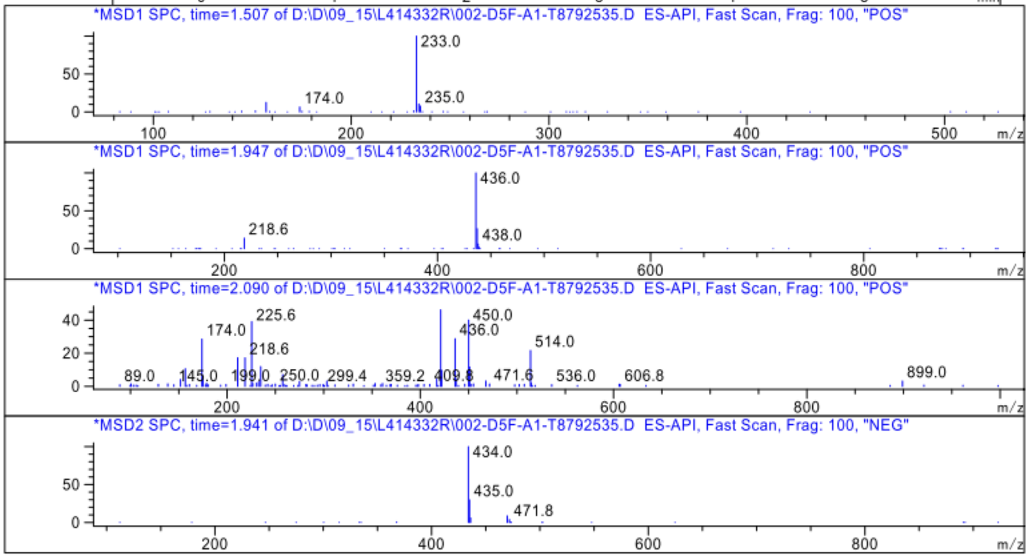

COMPOUND 15

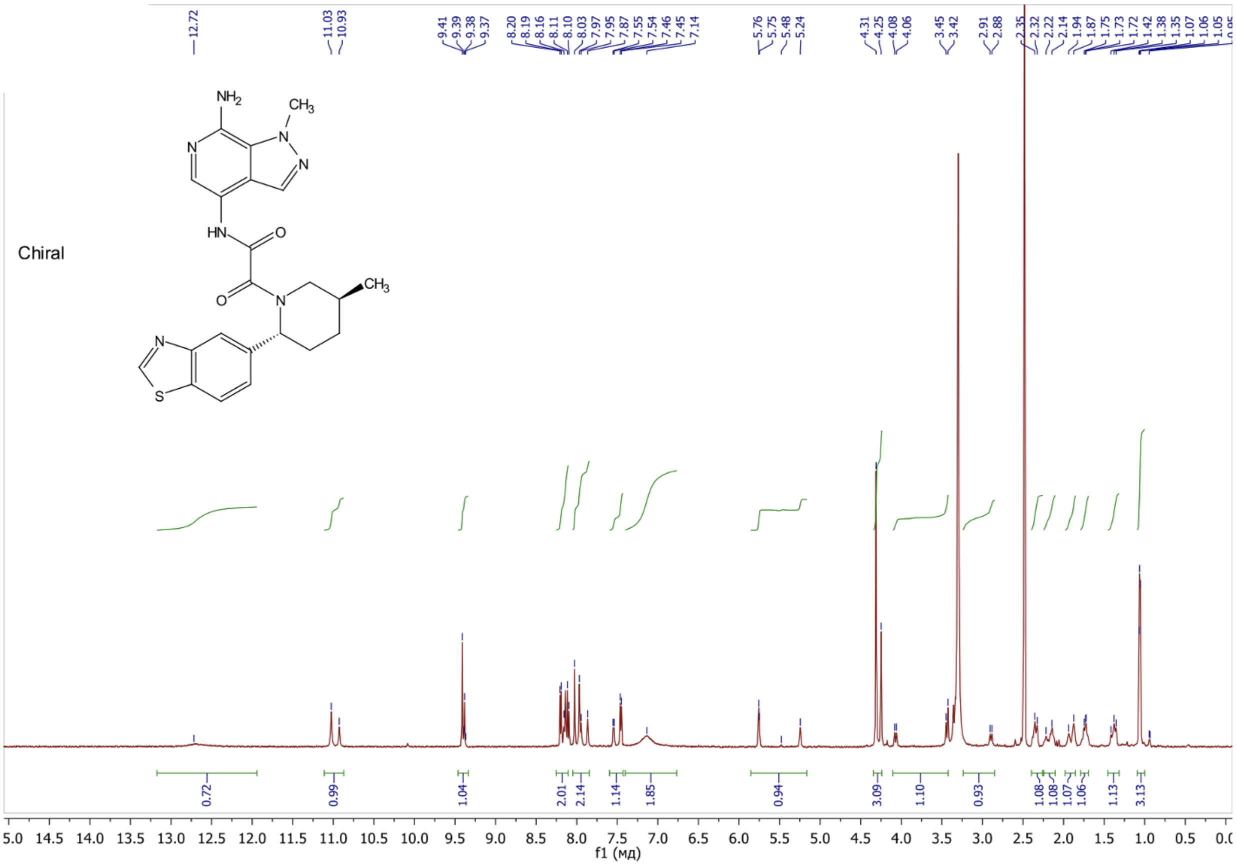
Chiral
NH2
CH3
N
HN
O
S
f1 (мд)

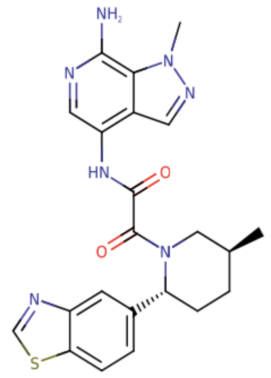

Mol Wt 449.53

Exact Mass 449.18

#	Time	Area%
1	2.154	100.00

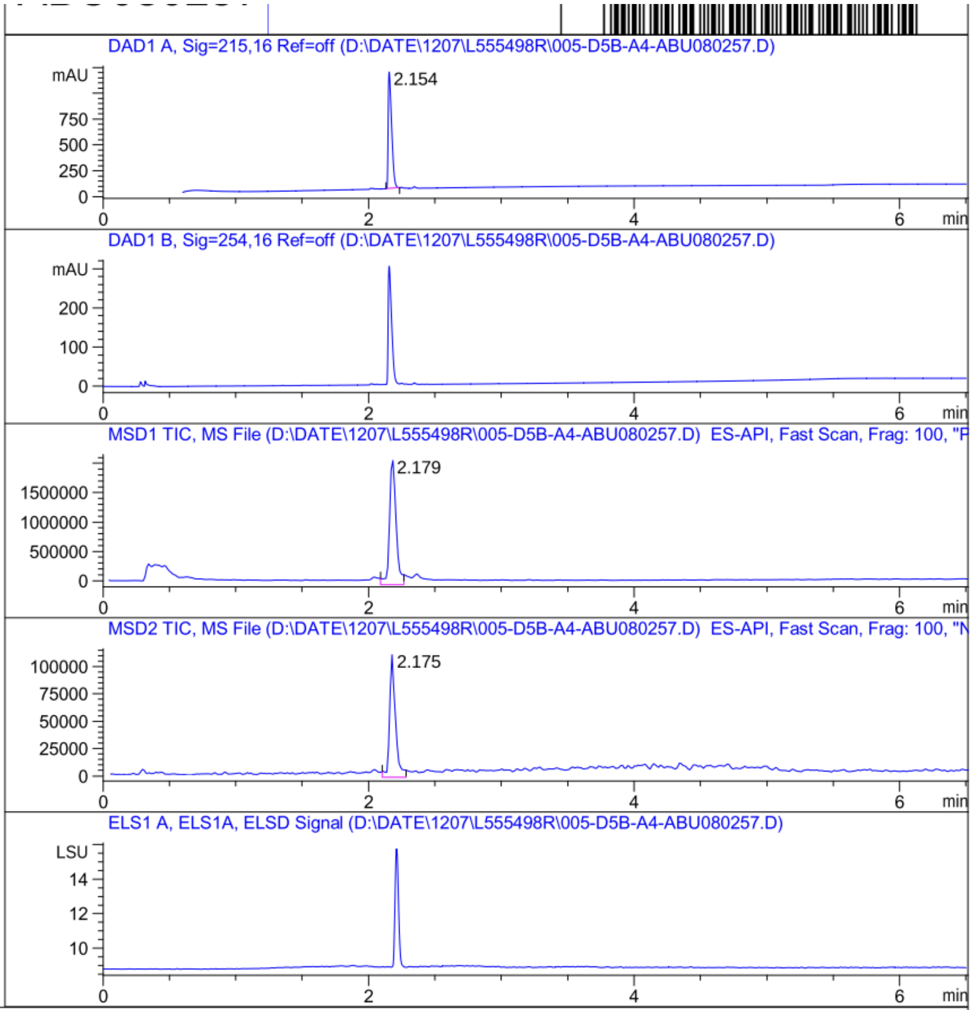

RT 2.179

RT 2.175

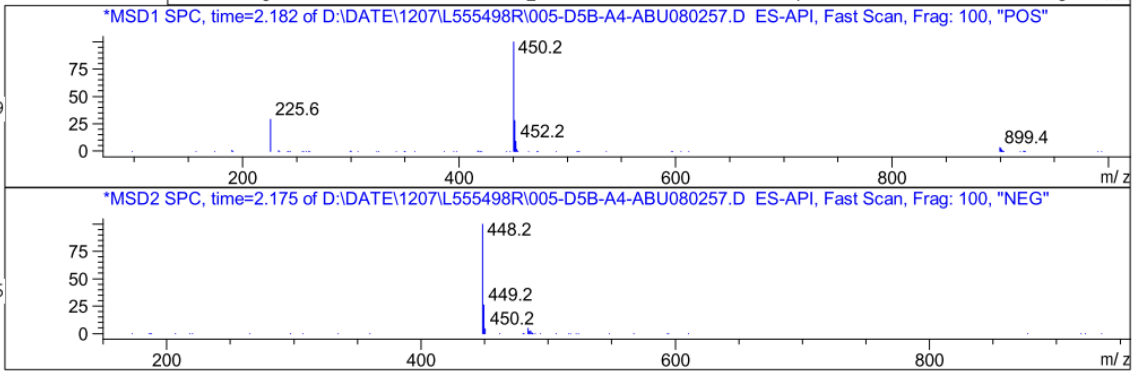

COMPOUND 16

EN-TG2-8272

Chiral

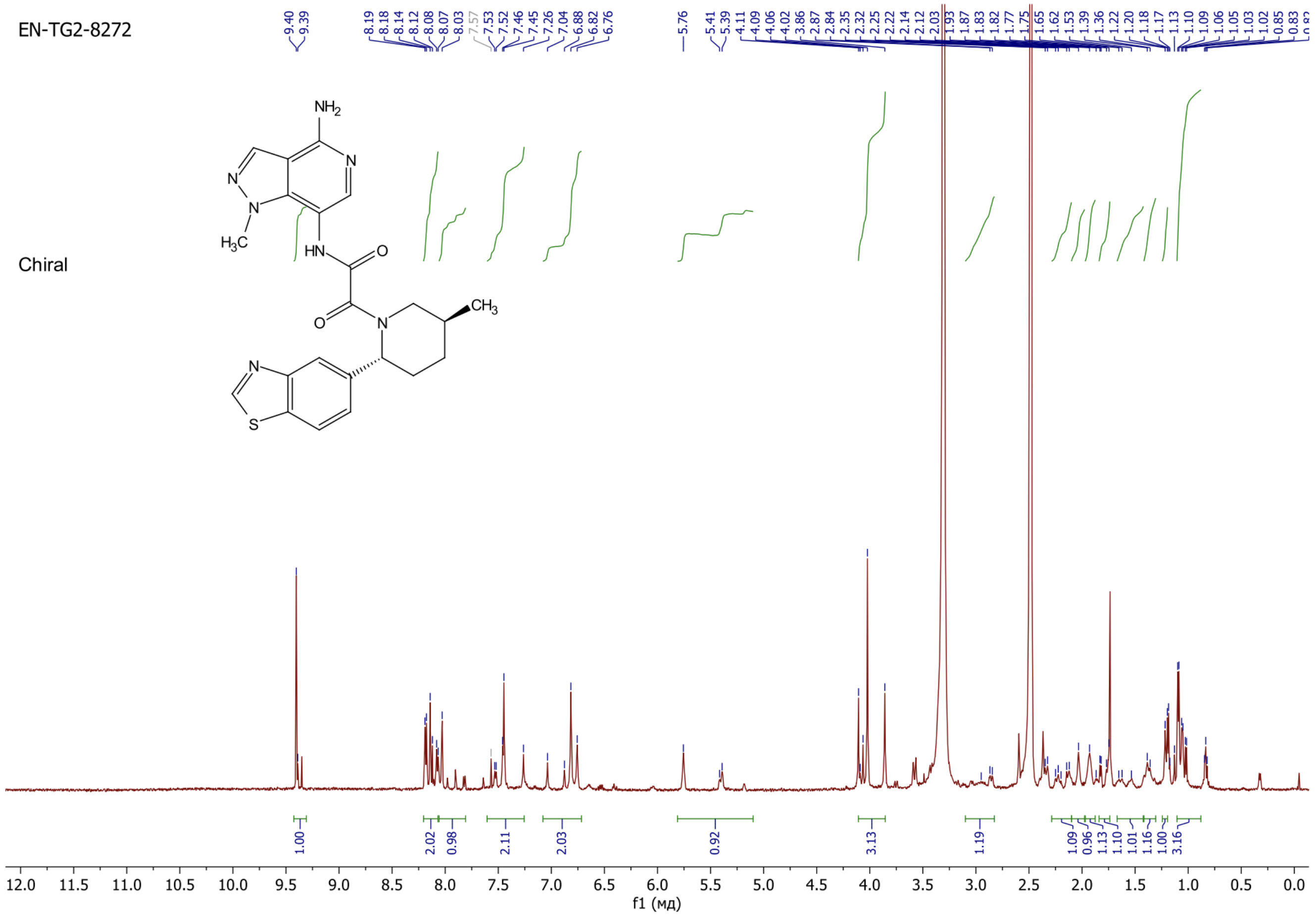

MaxPeak: 98.76%
Ret_Time: 2.149 min

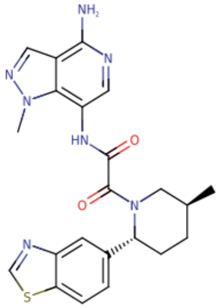

Mol Wt 449.53

Exact Mass 449.18

#	Time	Area%
1	2.149	98.76
2	2.356	1.24

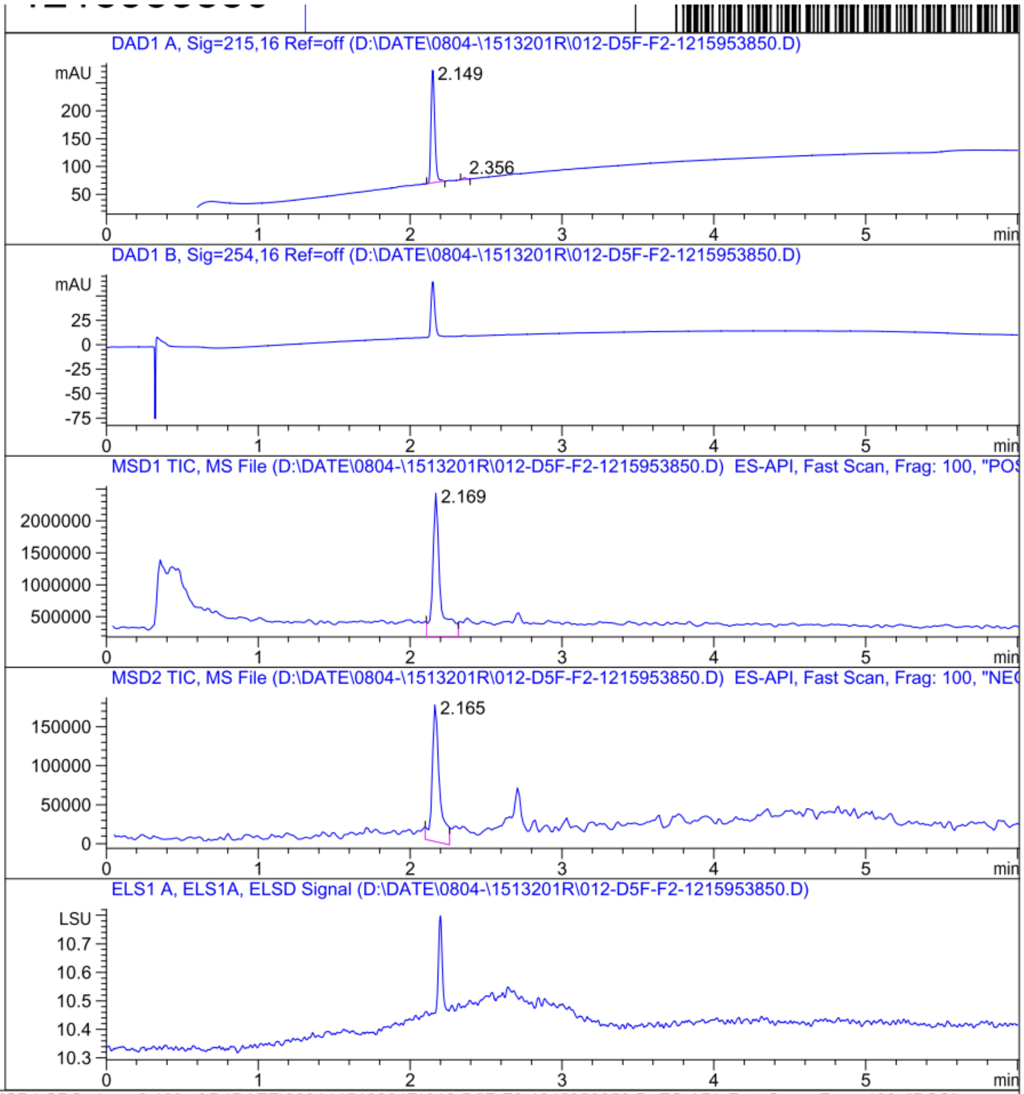

RT 2.169

RT 2.165

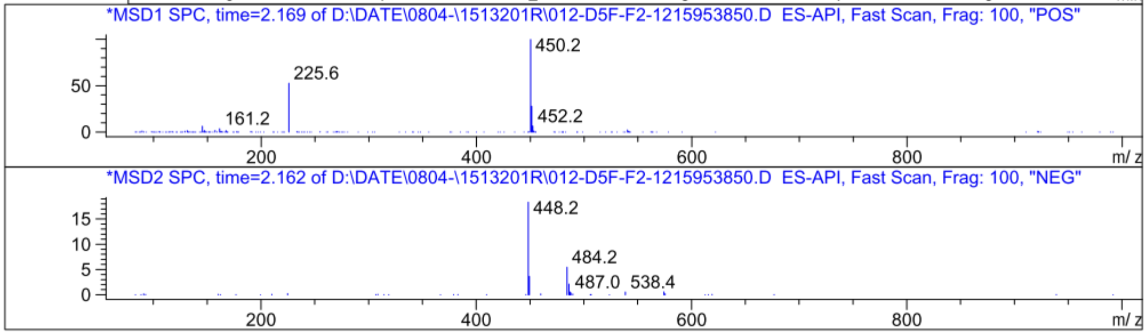

COMPOUND 17

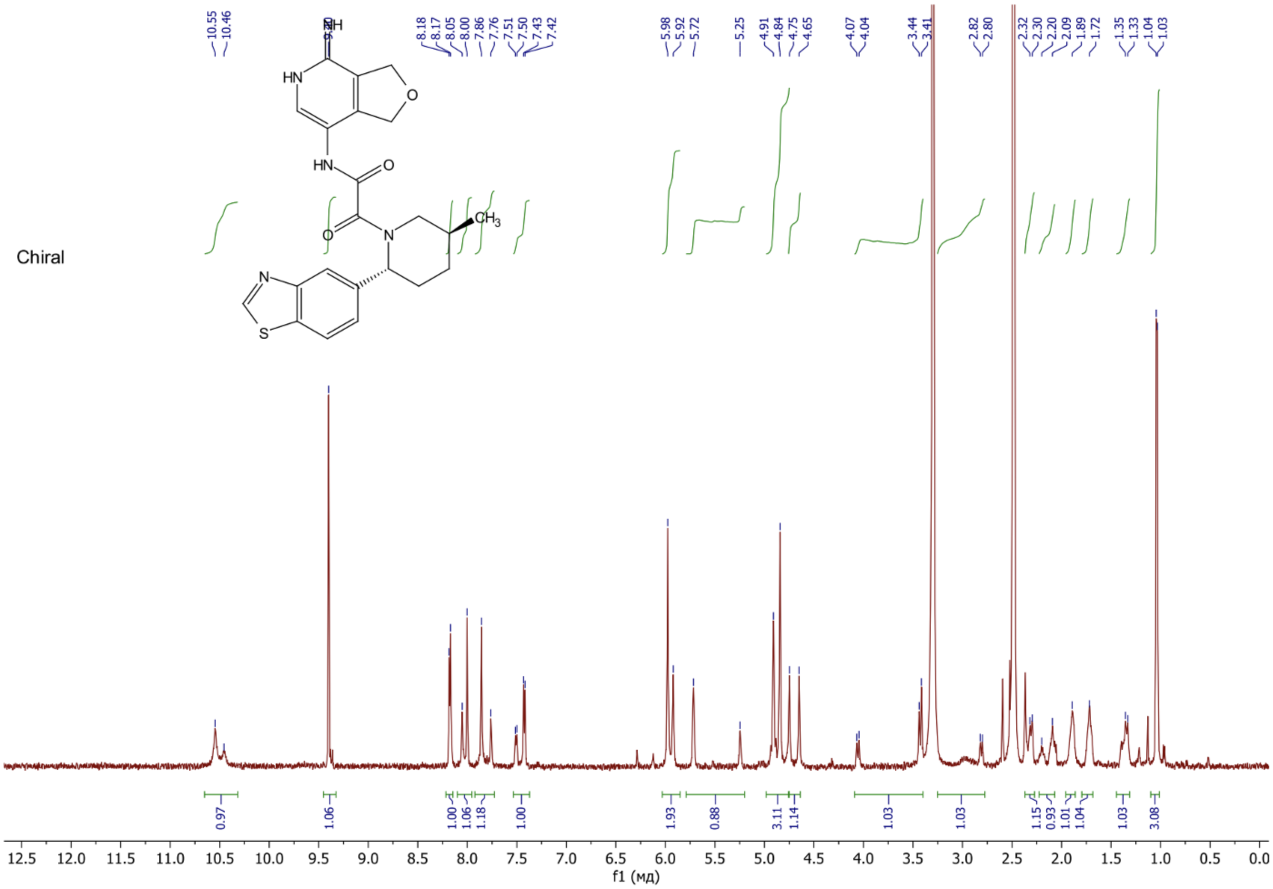
Chiral
NH
HN
O
HN
O
O
N
CH3
N
S
10.55
10.46
9.40
8.18
8.17
8.05
8.00
7.86
7.76
7.51
7.50
7.43
7.42
5.98
5.92
5.72
5.25
4.91
4.84
4.75
4.65
4.07
4.04
3.44
3.41
2.82
2.80
2.32
2.30
2.20
2.09
1.89
1.72
1.35
1.33
1.04
1.03
0.97
1.06
1.00
1.06
1.18
1.00
1.93
0.88
3.11
1.14
1.03
1.03
1.15
0.93
1.01
1.04
1.03
3.08
12.5 12.0 11.5 11.0 10.5 10.0 9.5 9.0 8.5 8.0 7.5 7.0 6.5 6.0 5.5 5.0 4.5 4.0 3.5 3.0 2.5 2.0 1.5 1.0 0.5 0.0
f1 (мд)

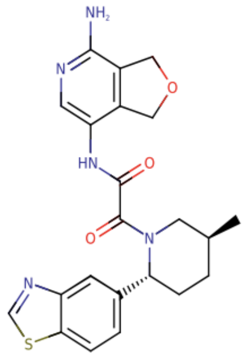

Mol Wt 437.52

Exact Mass 437.17

#	Time	Area%
1	2.482	98.17
2	2.570	1.83

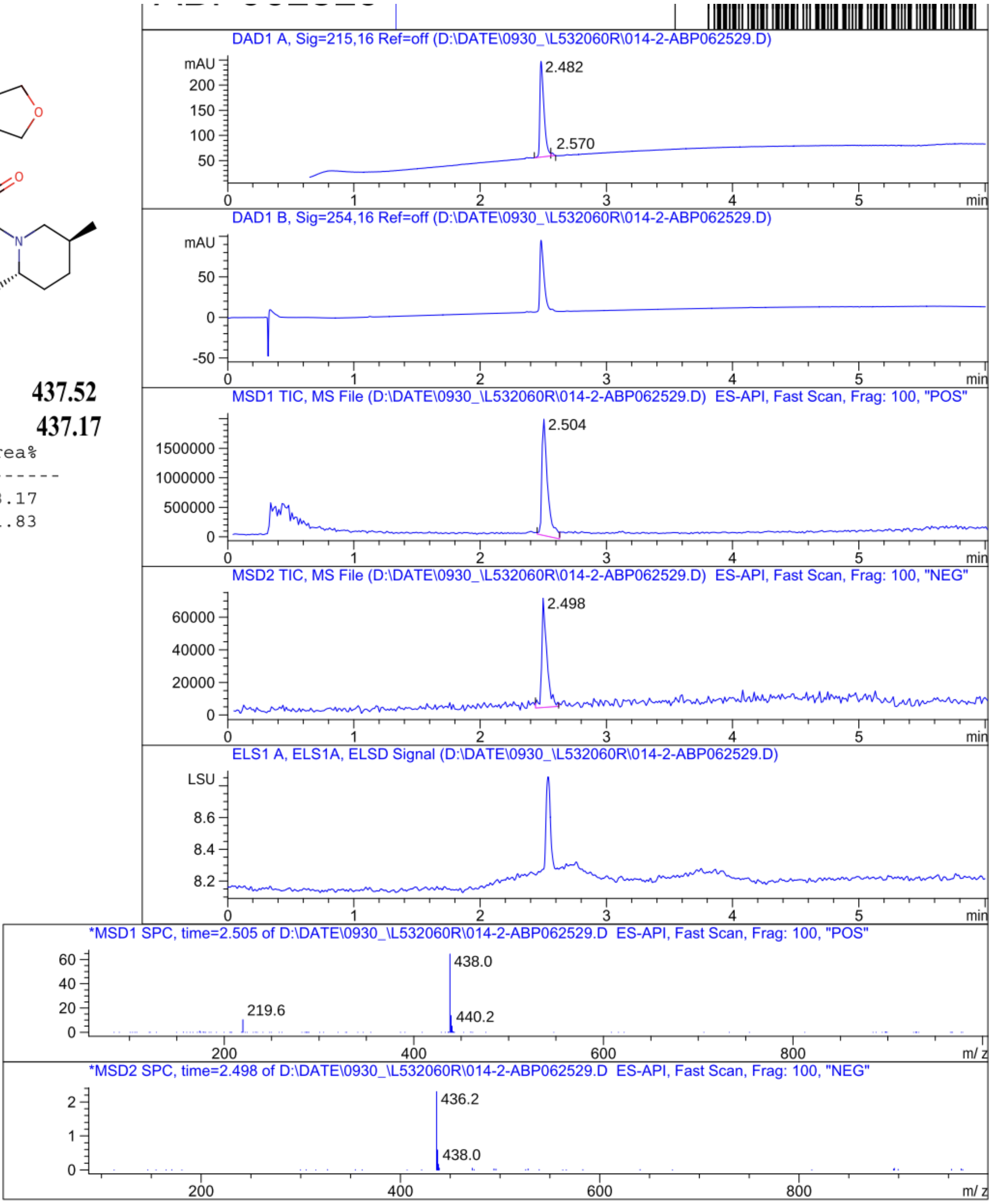

RT 2.504

RT 2.498

COMPOUND 18

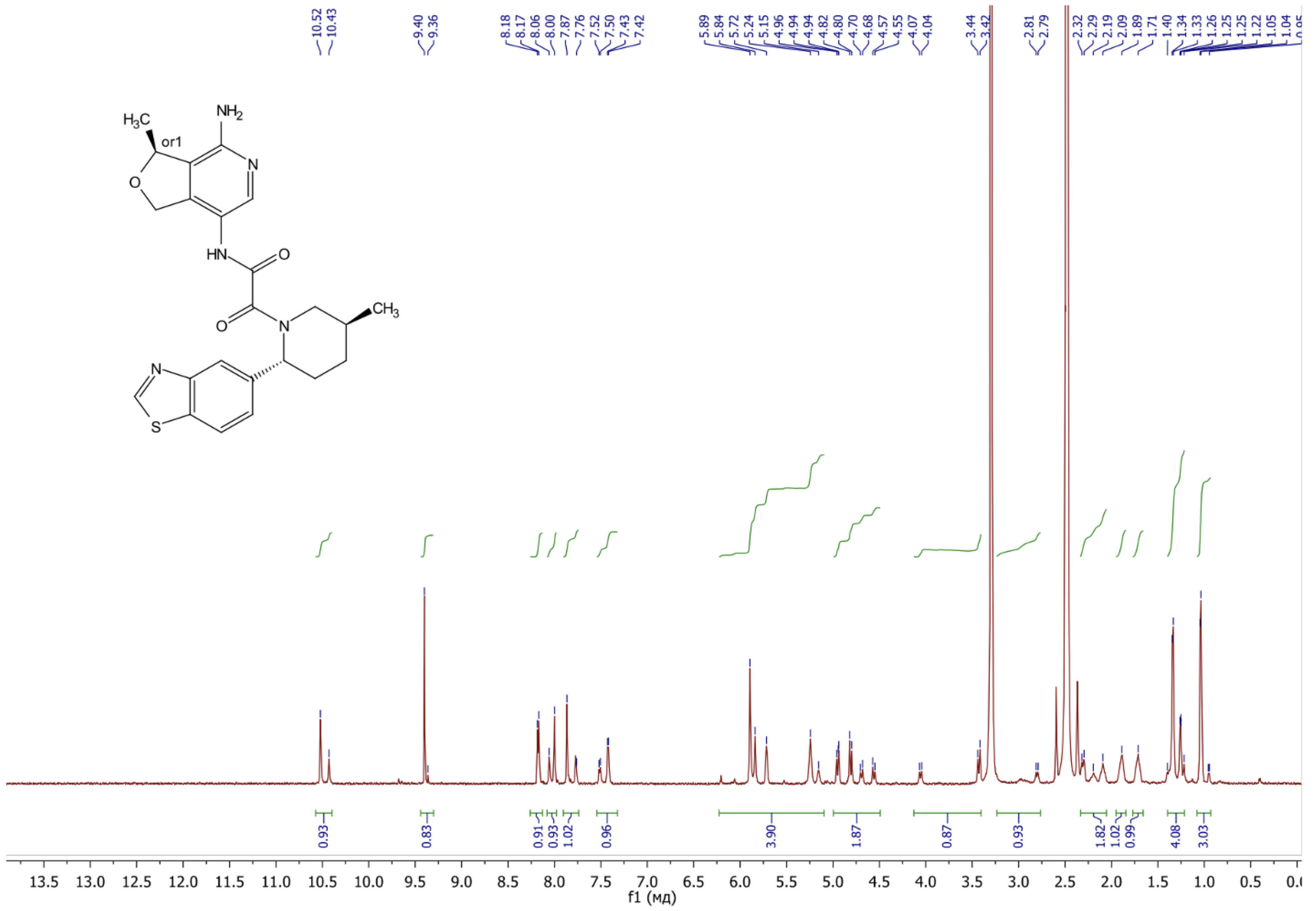

MaxPeak: 100.00%
Ret_Time: 2.659 min

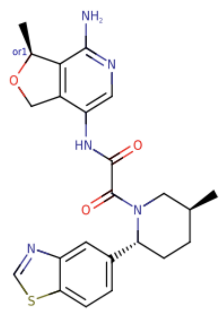

Mol Wt 451.54

Exact Mass 451.19

#	Time	Area%
1	2.659	100.00

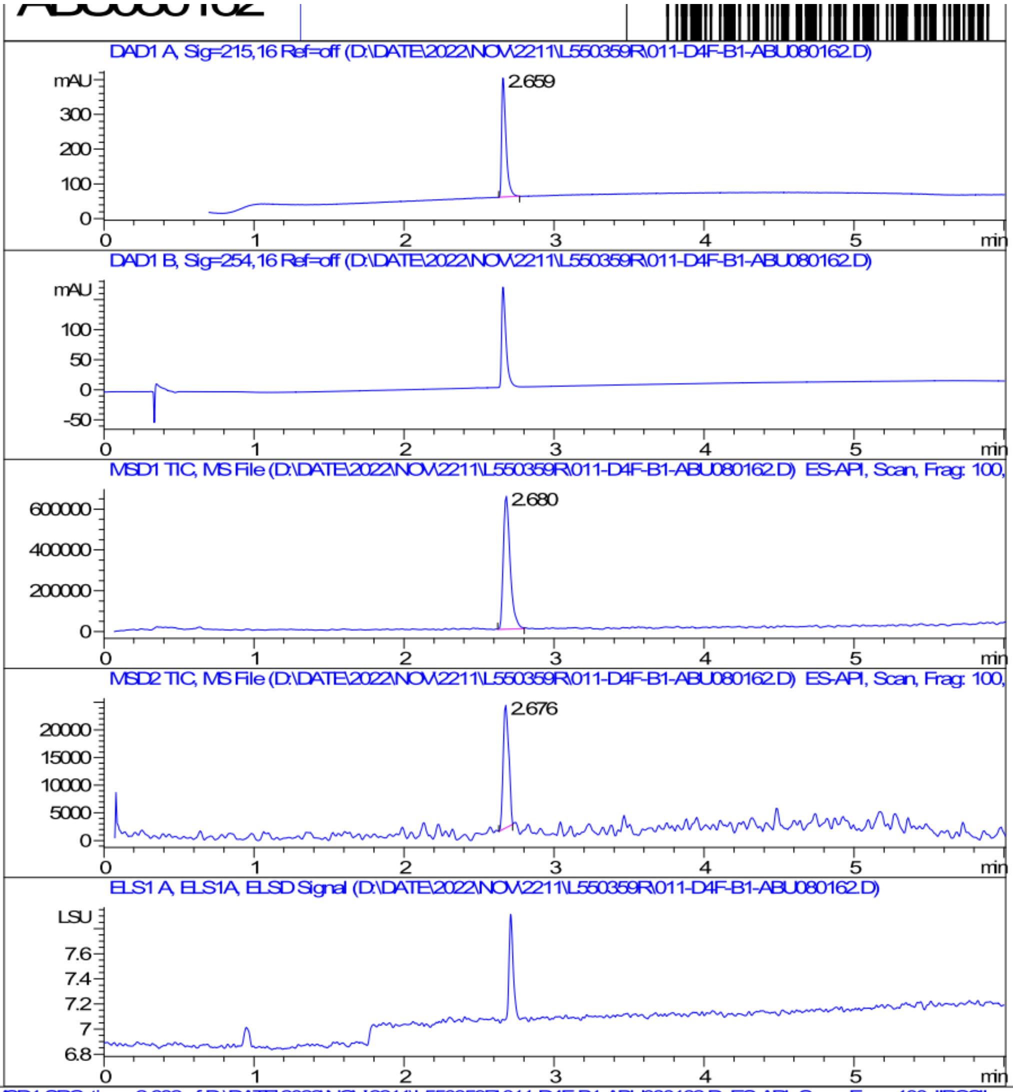

RT 2.680

RT 2.676

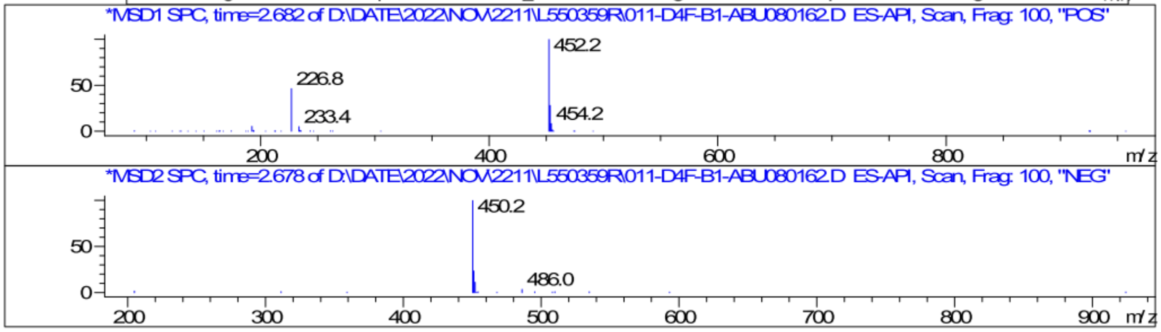

COMPOUND 19

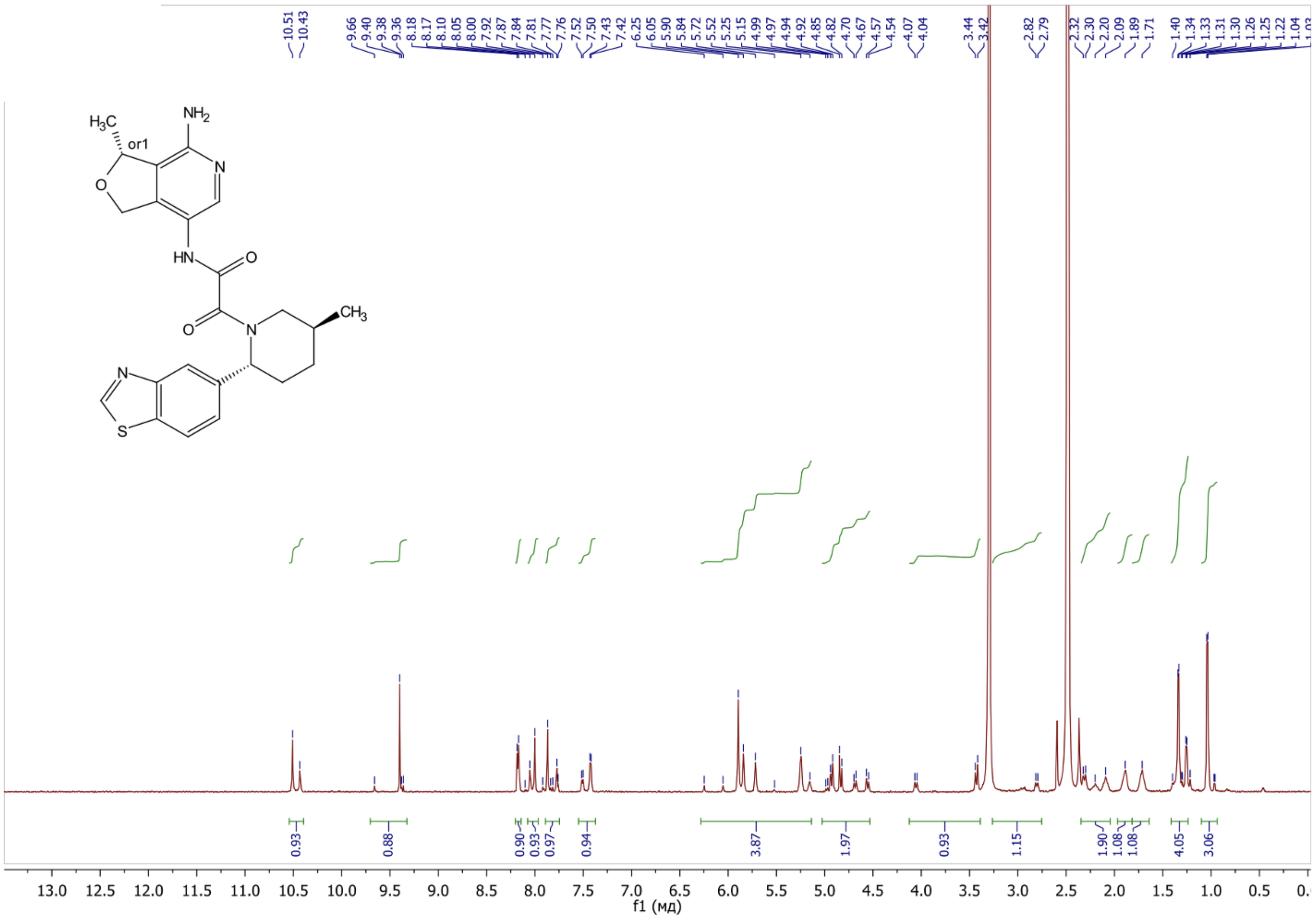

```
MaxPeak:  100.00%
Ret_Time: 2.664 min
```

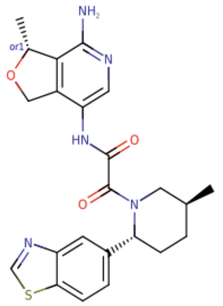

Mol Wt 451.54

Exact Mass 451.19

```
#  Time    Area%
-----------------
1  2.664   100.00
```

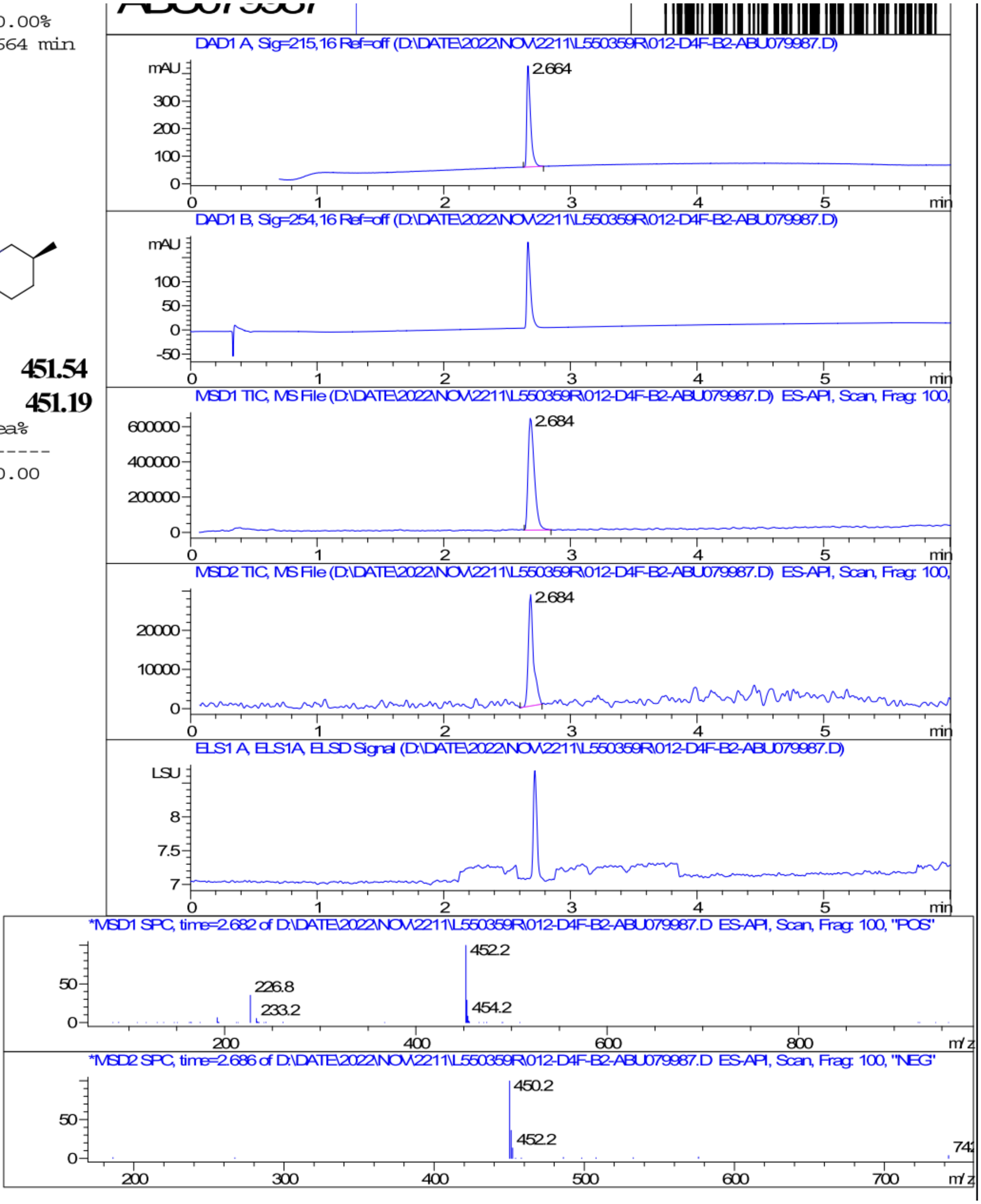

RT 2.684

RT 2.684

COMPOUND 20

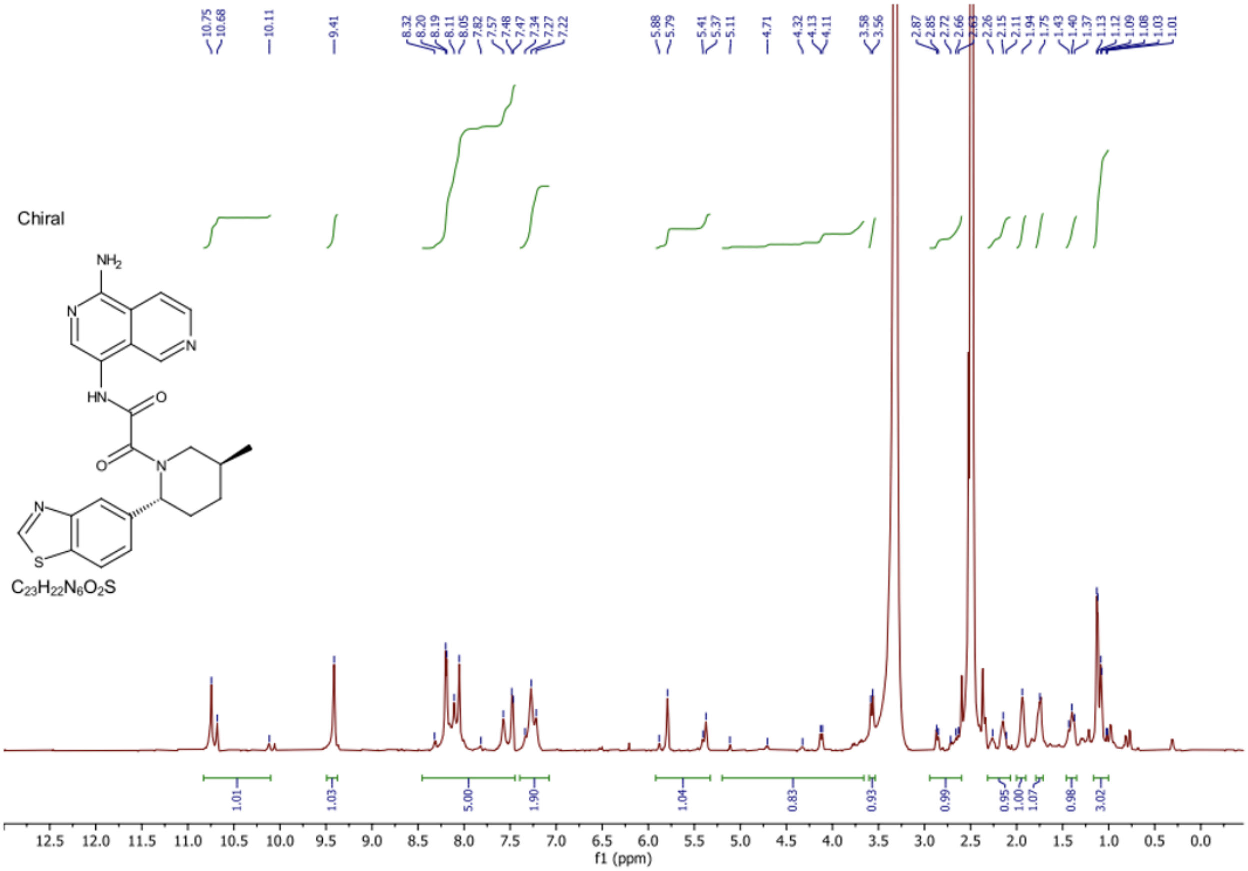

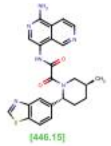

ABH032617

L403250R
LCMS-16
6min_4-6x30_1-5_V.M
12:32 16.08.2021
MaxPeak: 85.6%

#	RT	DAD1A	DAD1B	MSD1	MSD2	ELSD	MSD1 ions	MSD1 rt	MSD2 ions	MSD2 rt	Info
1	2.359	1.3%	1.7%	2.0%	—	—	437.0(100)	2.380	—	—	
2	2.498	85.6%	82.6%	94.0%	100.0%	100.0%	447.2(73),224.2(27)	2.512	445.2(100)	2.513	P +H+,P NEG
3	2.565	11.8%	11.6%	—	—	—	—	—	—	—	
4	3.098	1.3%	4.1%	4.0%	—	—	246.2(100)	3.117	—	—	

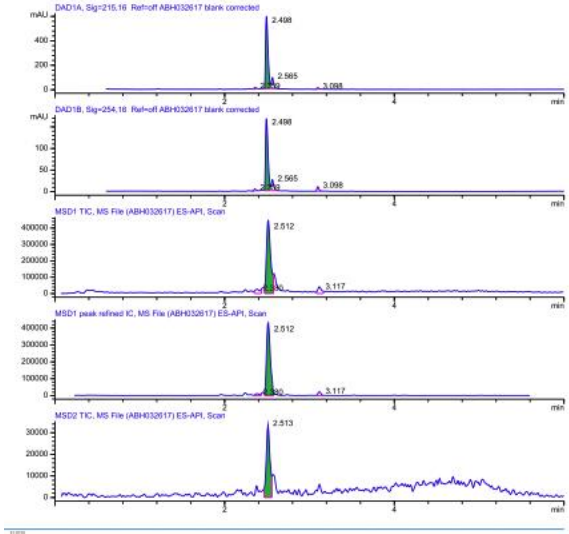

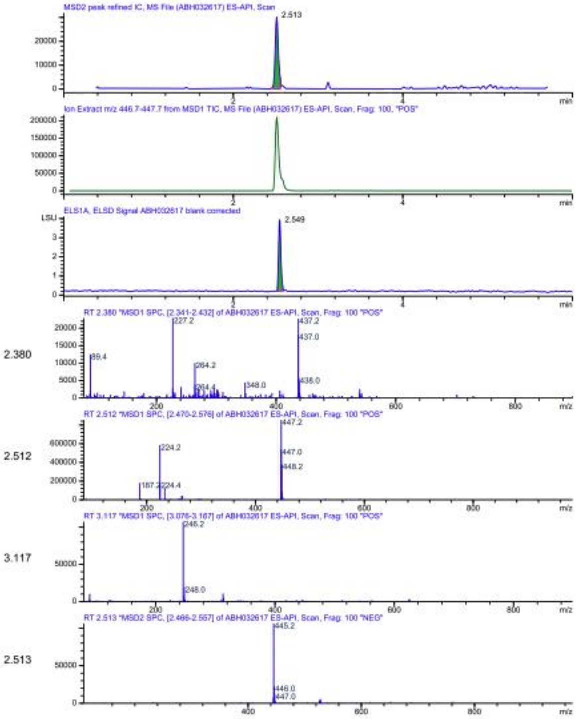

COMPOUND 21

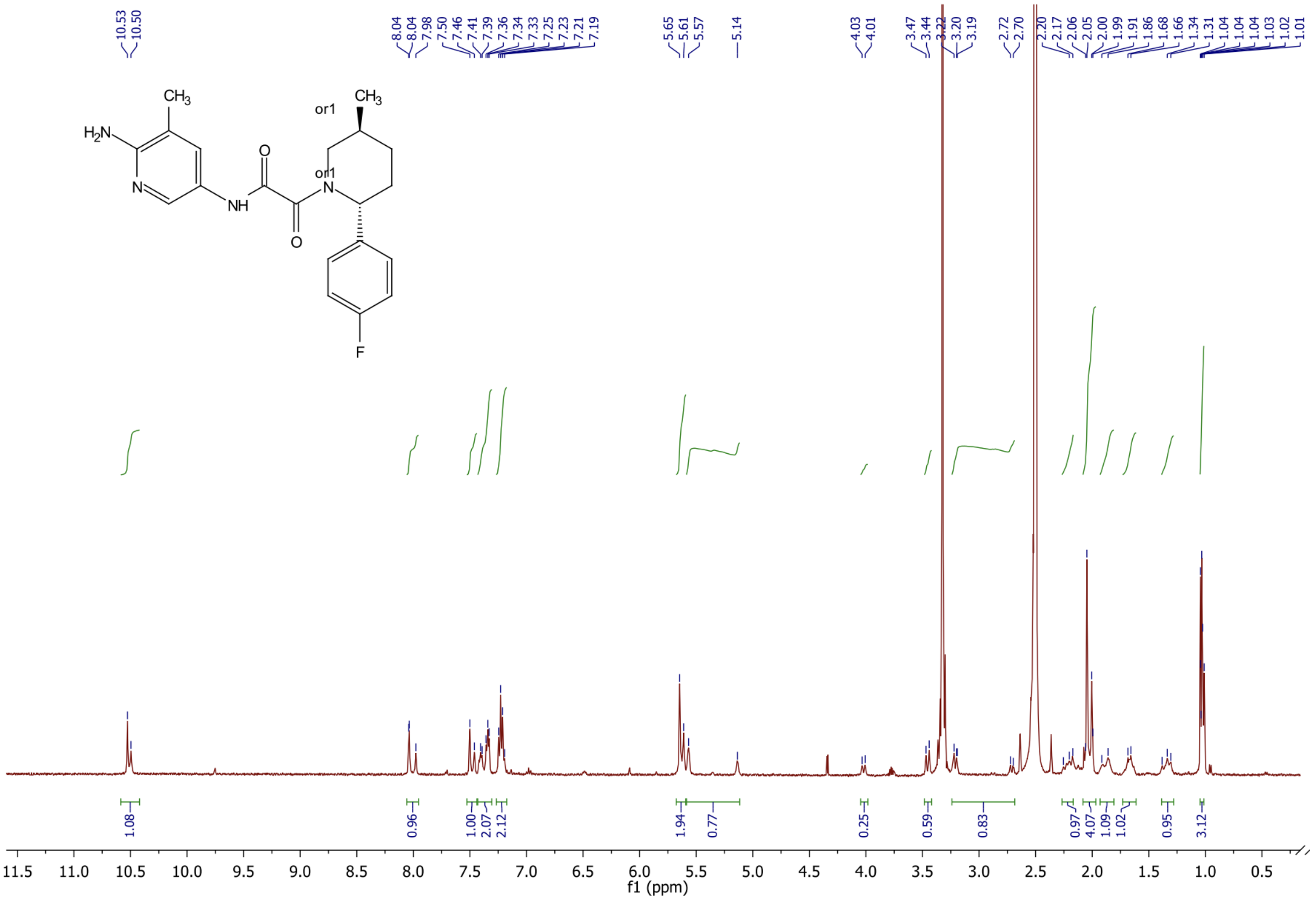
10.53
10.50
8.04
8.04
7.98
7.50
7.46
7.41
7.39
7.36
7.34
7.33
7.25
7.23
7.21
7.19
5.65
5.61
5.57
5.14
4.03
4.01
3.47
3.44
3.22
3.20
3.19
2.72
2.70
2.20
2.17
2.06
2.05
2.00
1.99
1.91
1.86
1.68
1.66
1.34
1.31
1.04
1.04
1.04
1.03
1.02
1.01
CH_3
H_2N
N
NH
O
O
or1
or1
N
CH_3
F
1.08
0.96
1.00
2.07
2.12
1.94
0.77
0.25
0.59
0.83
0.97
4.07
1.09
1.02
0.95
3.12
11.5
11.0
10.5
10.0
9.5
9.0
8.5
8.0
7.5
7.0
6.5
6.0
5.5
5.0
4.5
4.0
3.5
3.0
2.5
2.0
1.5
1.0
0.5
f1 (ppm)

MaxPeak: 100.00%
Ret_Time: 2.730 min

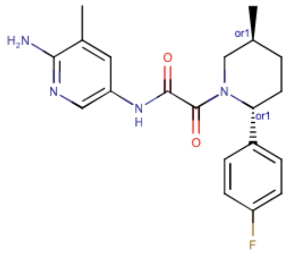

Mol Wt 370.42
Exact Mass 370.21

#	Time	Area%
1	2.730	100.00

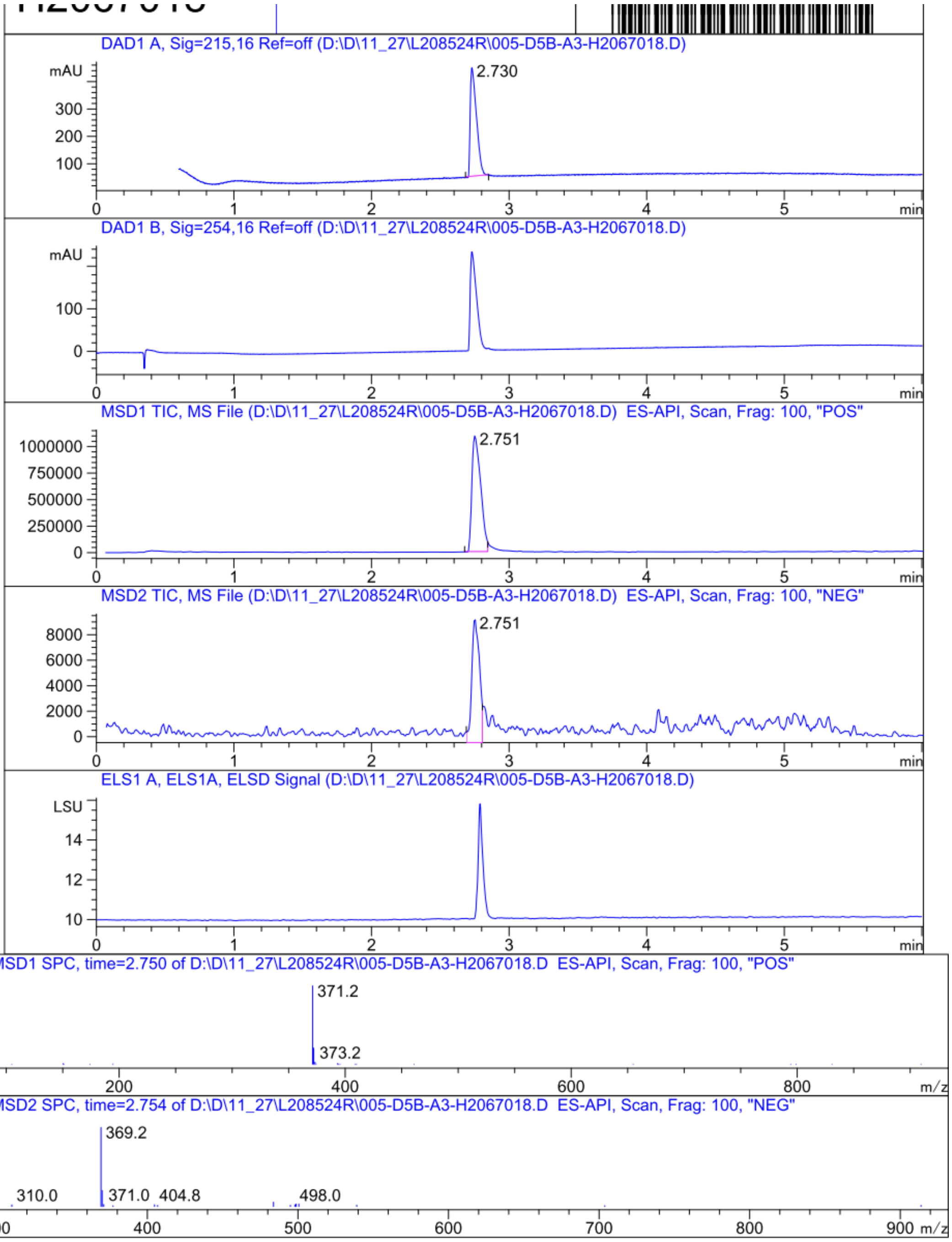

COMPOUND 22

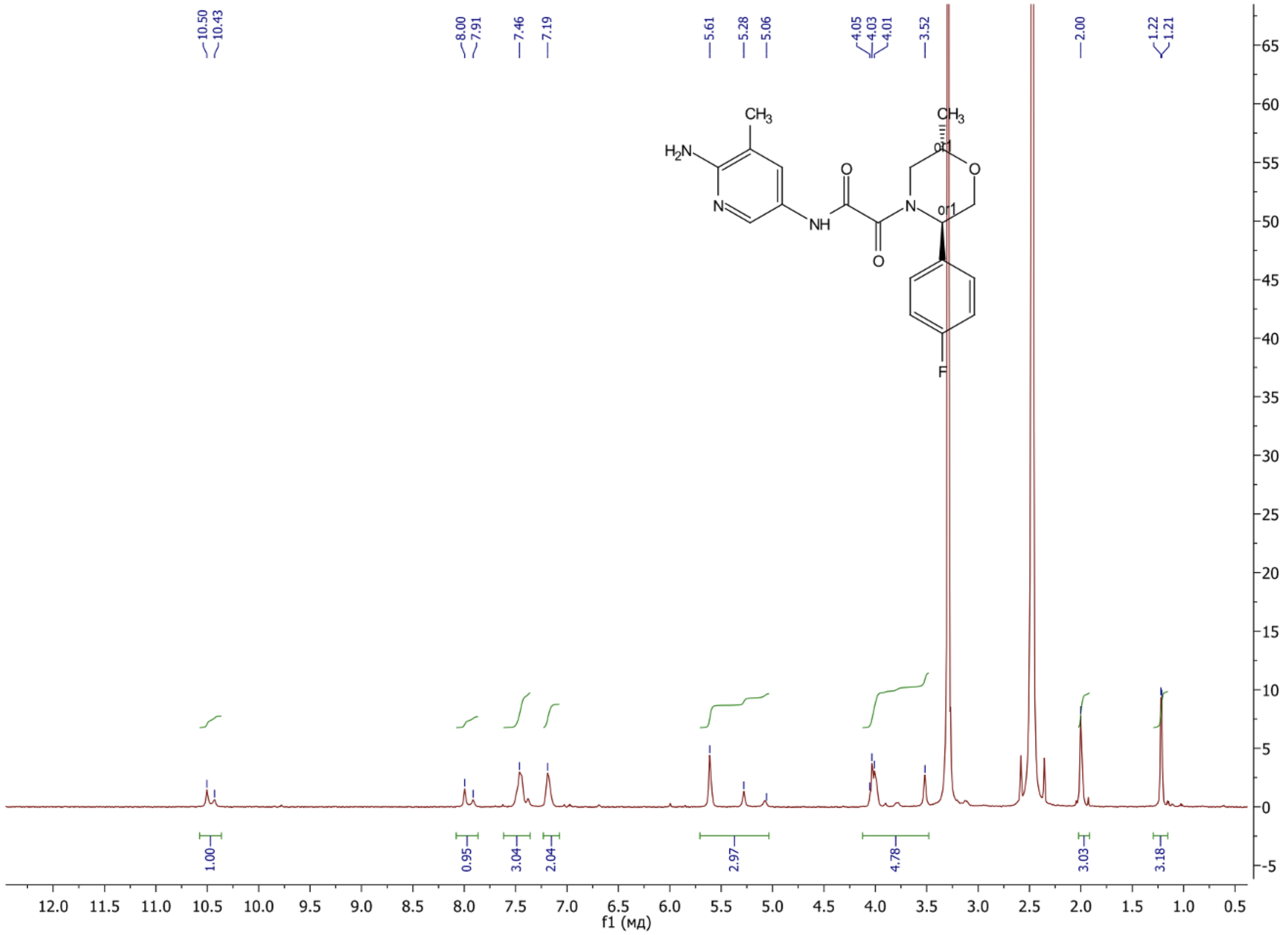

10.50
10.43
8.00
7.91
7.46
7.19
5.61
5.28
5.06
4.05
4.03
4.01
3.52
2.00
1.22
1.21
1.00
0.95
3.04
2.04
2.97
4.78
3.03
3.18
f1 (мд)

MaxPeak: 100.00%
Ret_Time: 1.157 min

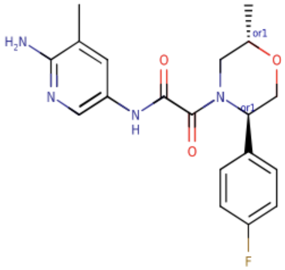

Mol Wt 372.39
Exact Mass 372.18

#	Time	Area%
1	1.157	100.00

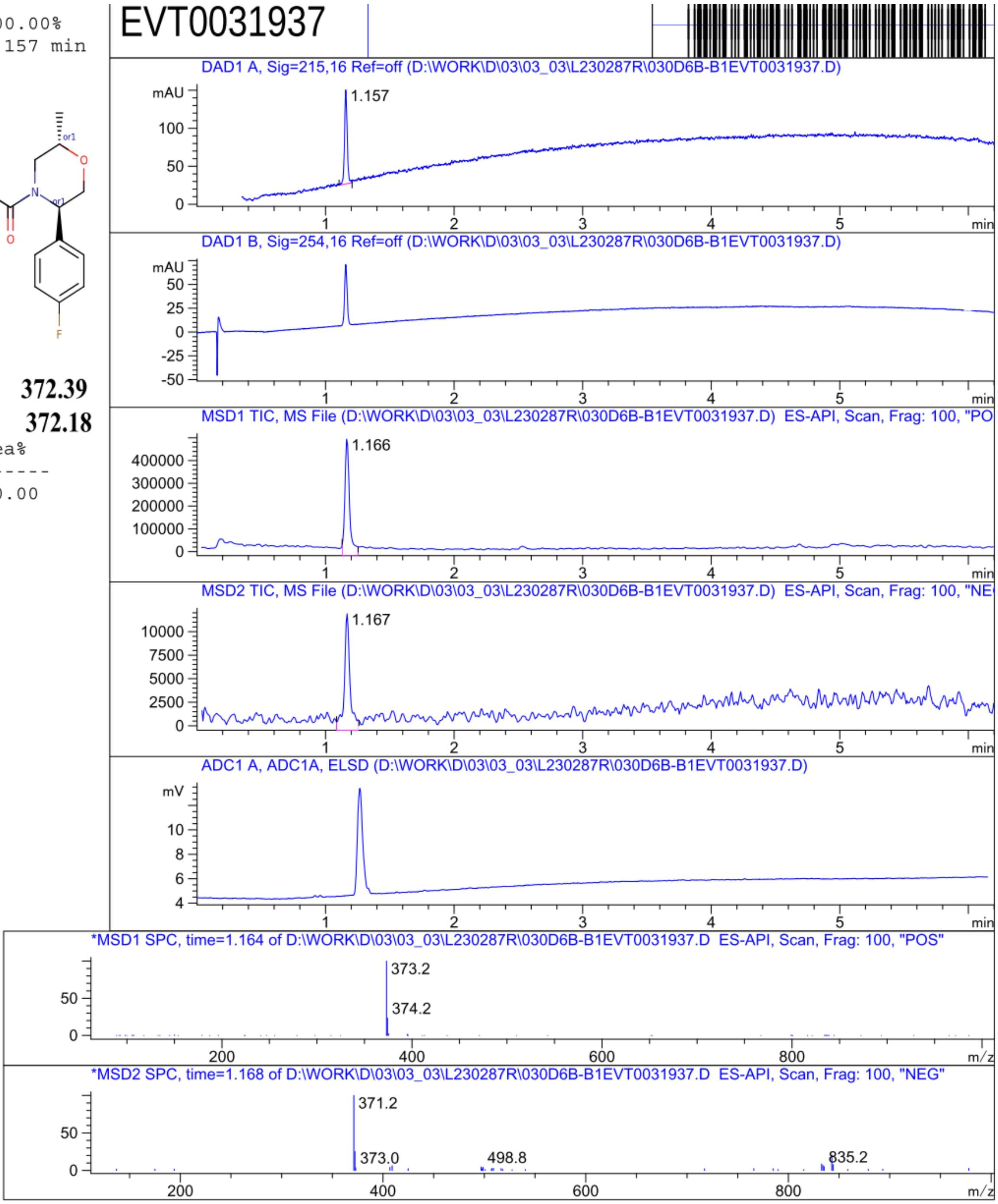

RT 1.166

RT 1.167

COMPOUND 23

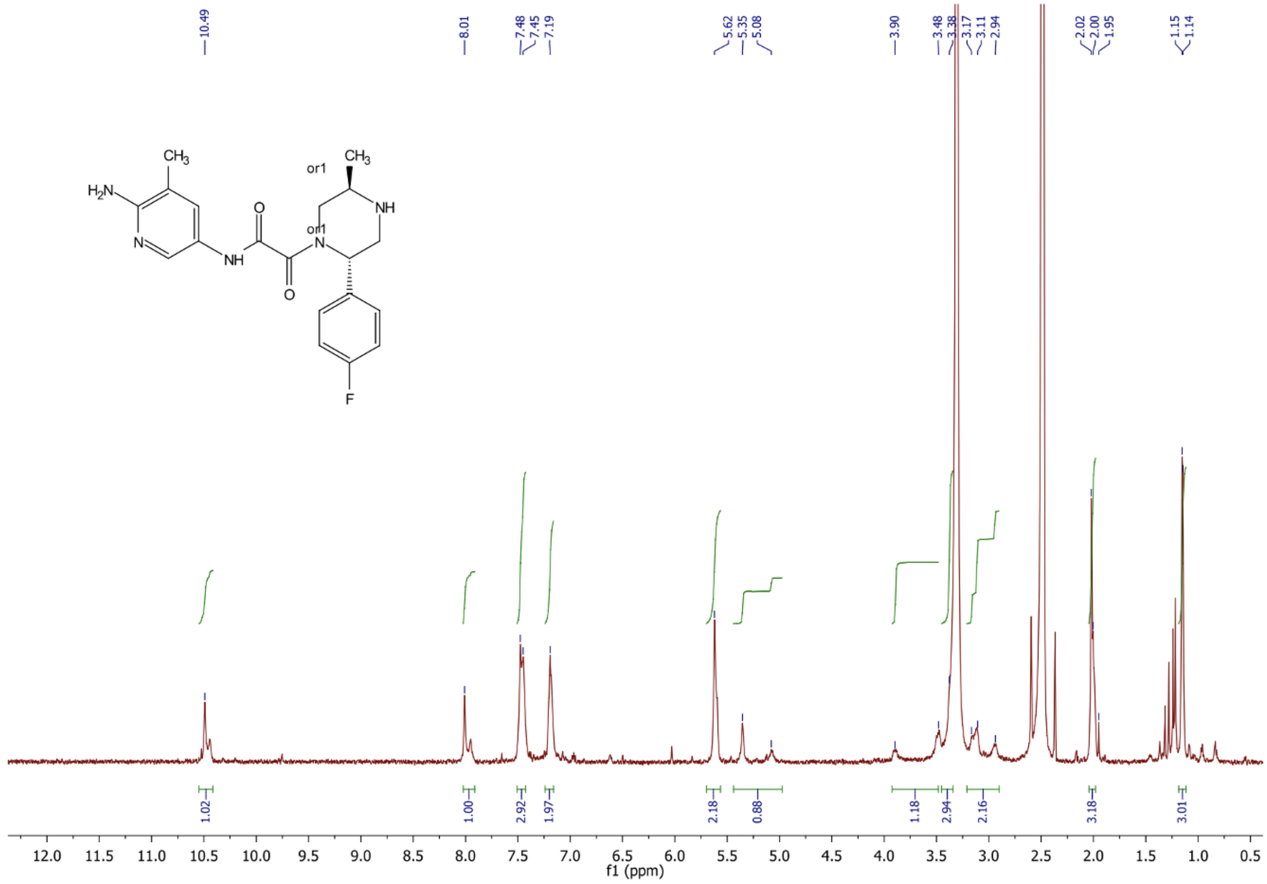

10.49
8.01
7.48
7.45
7.19
5.62
5.35
5.08
3.90
3.48
3.38
3.17
3.11
2.94
2.02
2.00
1.95
1.15
1.14
CH3
H2N
N
NH
O
or1
CH3
NH
or1
N
O
F
1.02
1.00
2.92
1.97
2.18
0.88
1.18
2.94
2.16
3.18
3.01
12.0 11.5 11.0 10.5 10.0 9.5 9.0 8.5 8.0 7.5 7.0 6.5 6.0 5.5 5.0 4.5 4.0 3.5 3.0 2.5 2.0 1.5 1.0 0.5
f1 (ppm)

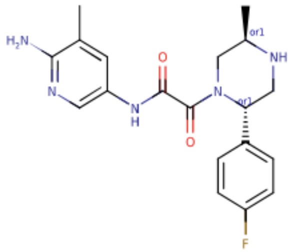

Mol Wt 371.41

Exact Mass 371.2

#	Time	Area%
1	1.098	95.27
2	2.044	4.73

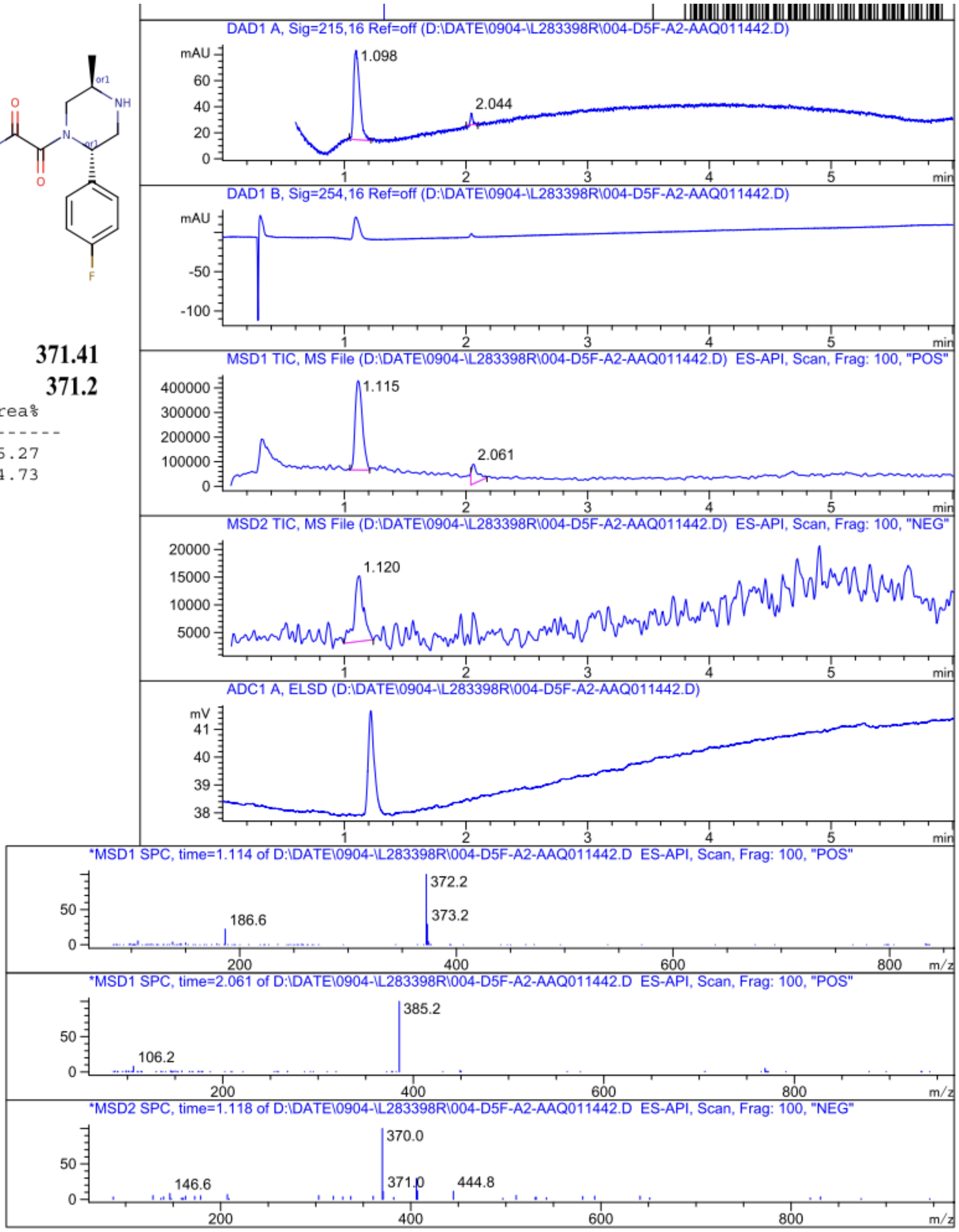

COMPOUND 24

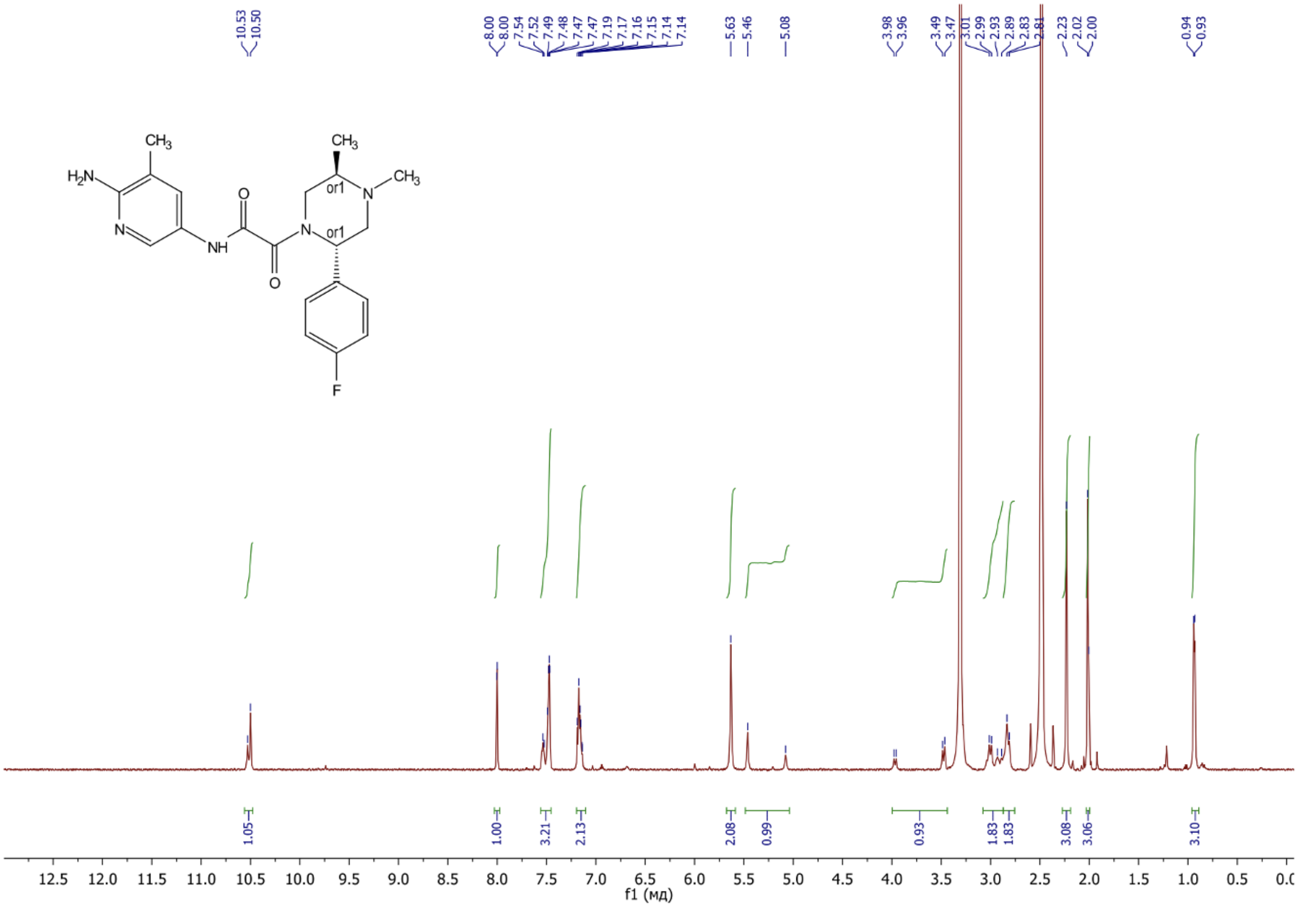
10.53
10.50
8.00
8.00
7.54
7.52
7.49
7.48
7.47
7.47
7.19
7.17
7.16
7.15
7.14
7.14
5.63
5.46
5.08
3.98
3.96
3.49
3.47
3.01
2.99
2.93
2.89
2.83
2.81
2.23
2.02
2.00
0.94
0.93
CH3
H2N
N
NH
O
O
CH3
or1
N
CH3
N
or1
F
1.05
1.00
3.21
2.13
2.08
0.99
0.93
1.83
1.83
3.08
3.06
3.10
12.5 12.0 11.5 11.0 10.5 10.0 9.5 9.0 8.5 8.0 7.5 7.0 6.5 6.0 5.5 5.0 4.5 4.0 3.5 3.0 2.5 2.0 1.5 1.0 0.5 0.0
f1 (мд)

MaxPeak: 100.00%
Ret_Time: 1.553 min

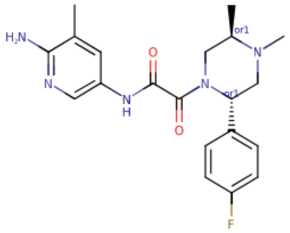

Mol Wt 385.44

Exact Mass 385.22

#	Time	Area%
1	1.553	100.00

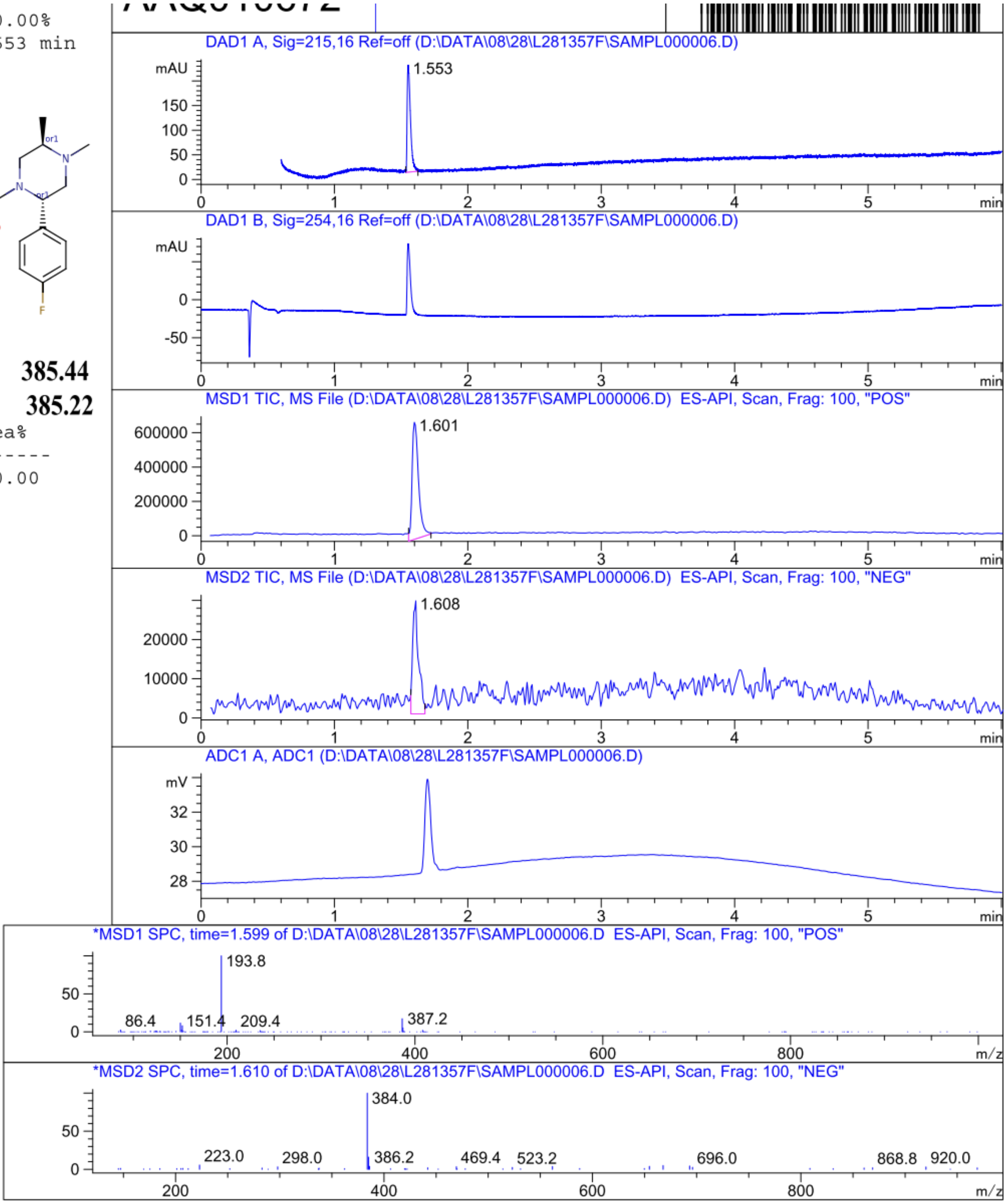

RT 1.601

RT 1.608

COMPOUND 25

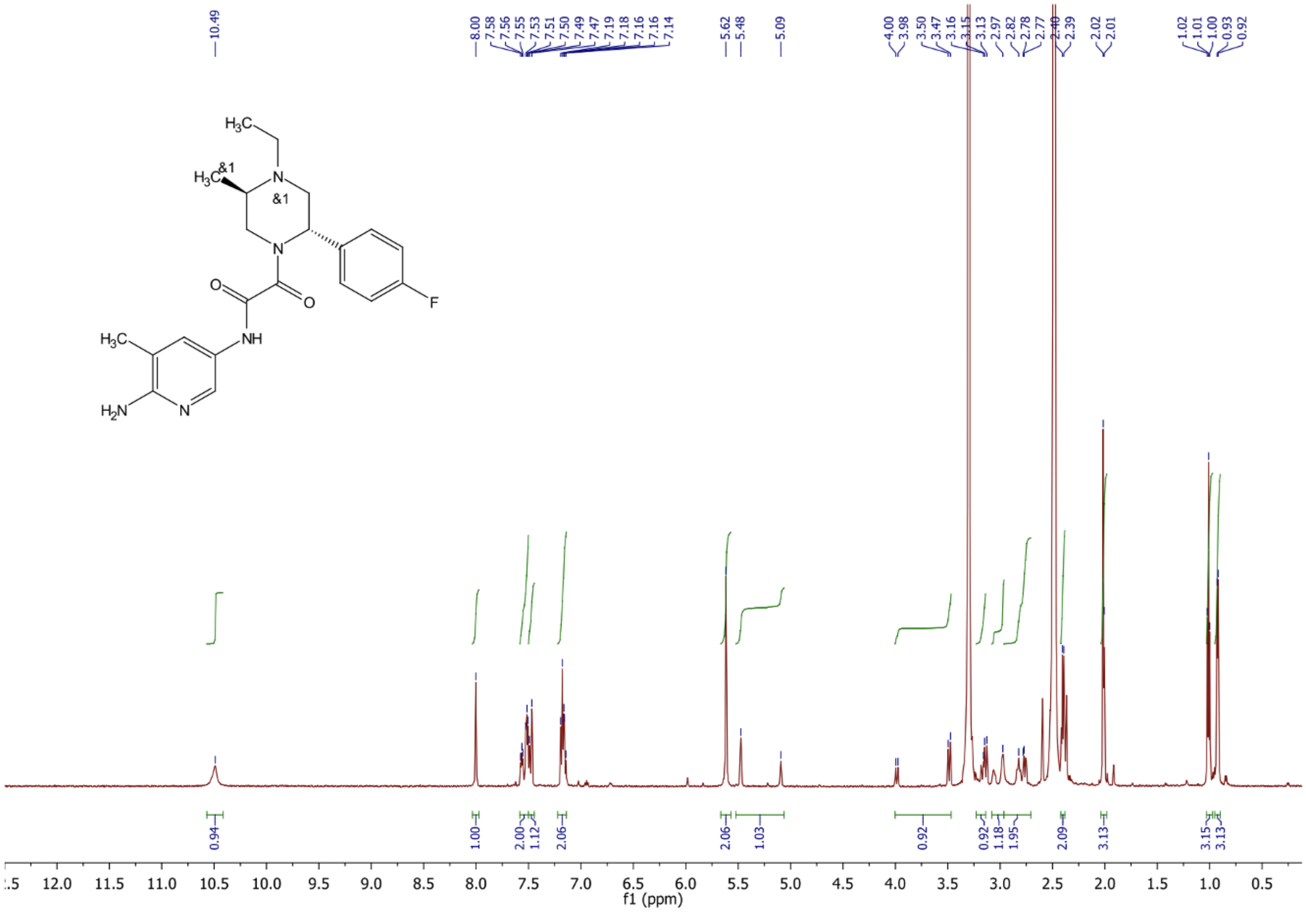
10.49
8.00 7.58 7.56 7.55 7.53 7.51 7.50 7.49 7.47 7.19 7.18 7.16 7.16 7.14
5.62 5.48
5.09
4.00 3.98 3.50 3.47 3.16 3.15 3.13 2.97 2.82 2.78 2.77 2.40 2.39
2.02 2.01
1.02 1.01 1.00 0.93 0.92
H3C
&1
N
&1
N
O
O
F
H3C
NH
H2N
N
0.94
1.00
2.00
1.12
2.06
2.06
1.03
0.92
0.92
1.18
1.95
2.09
3.13
3.15
3.13
f1 (ppm)

Ret_Time: 1.154 min

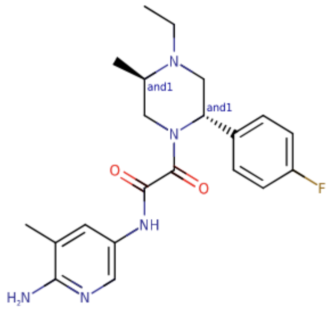

Mol Wt 399.46

Exact Mass 399.24

```
#  Time   Area%
------------------
1  1.154  100.00
```

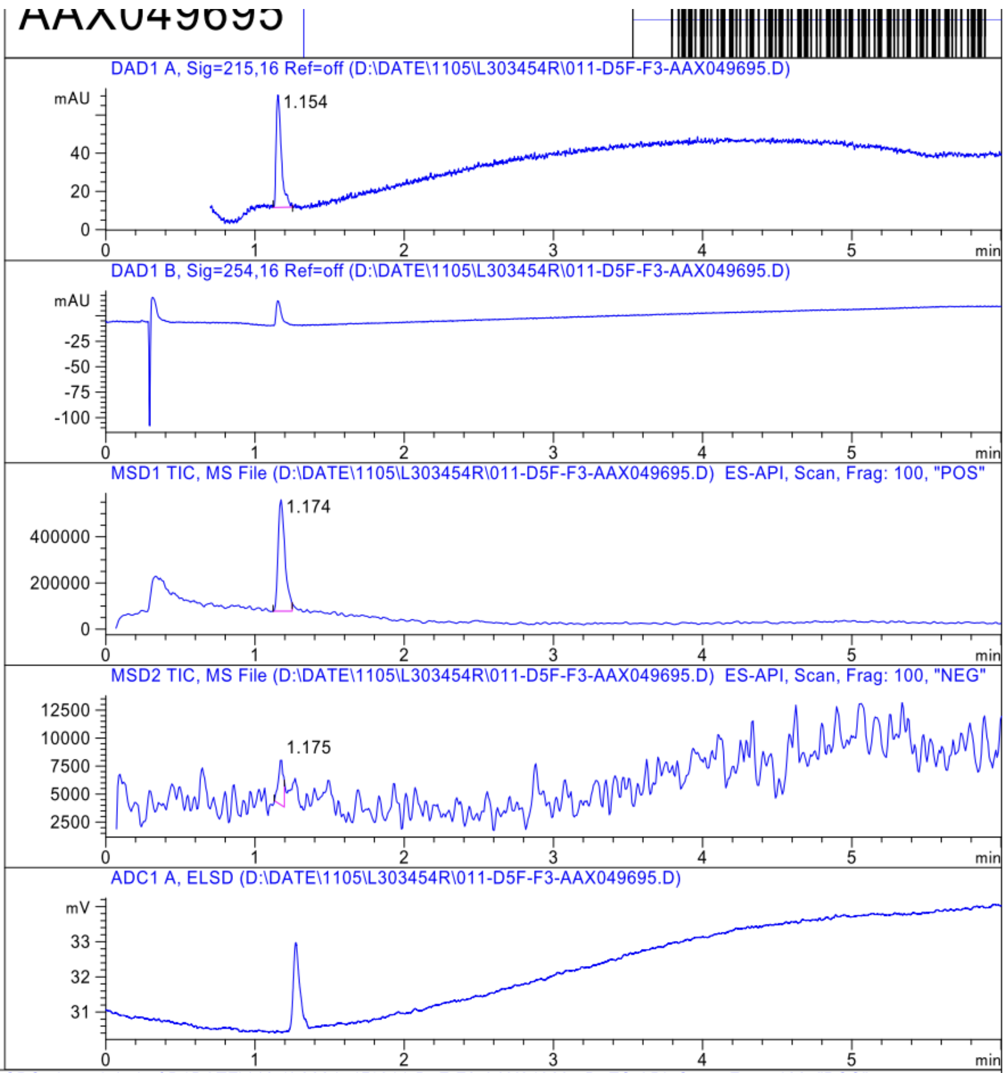

RT 1.174

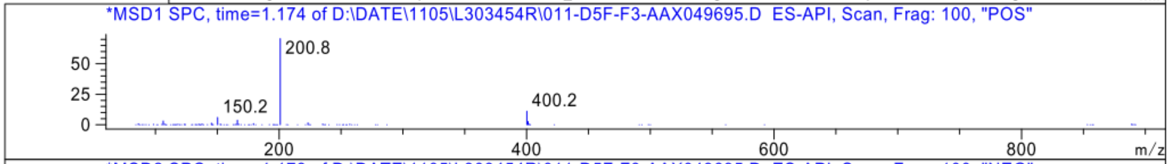

RT 1.175

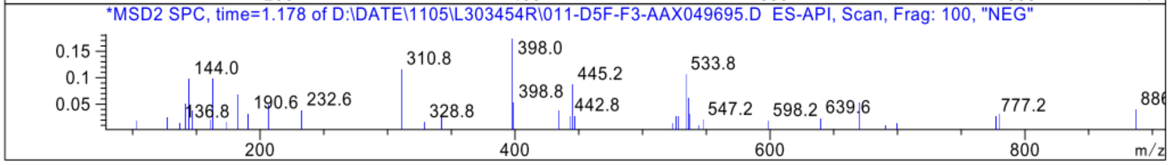

COMPOUND 26

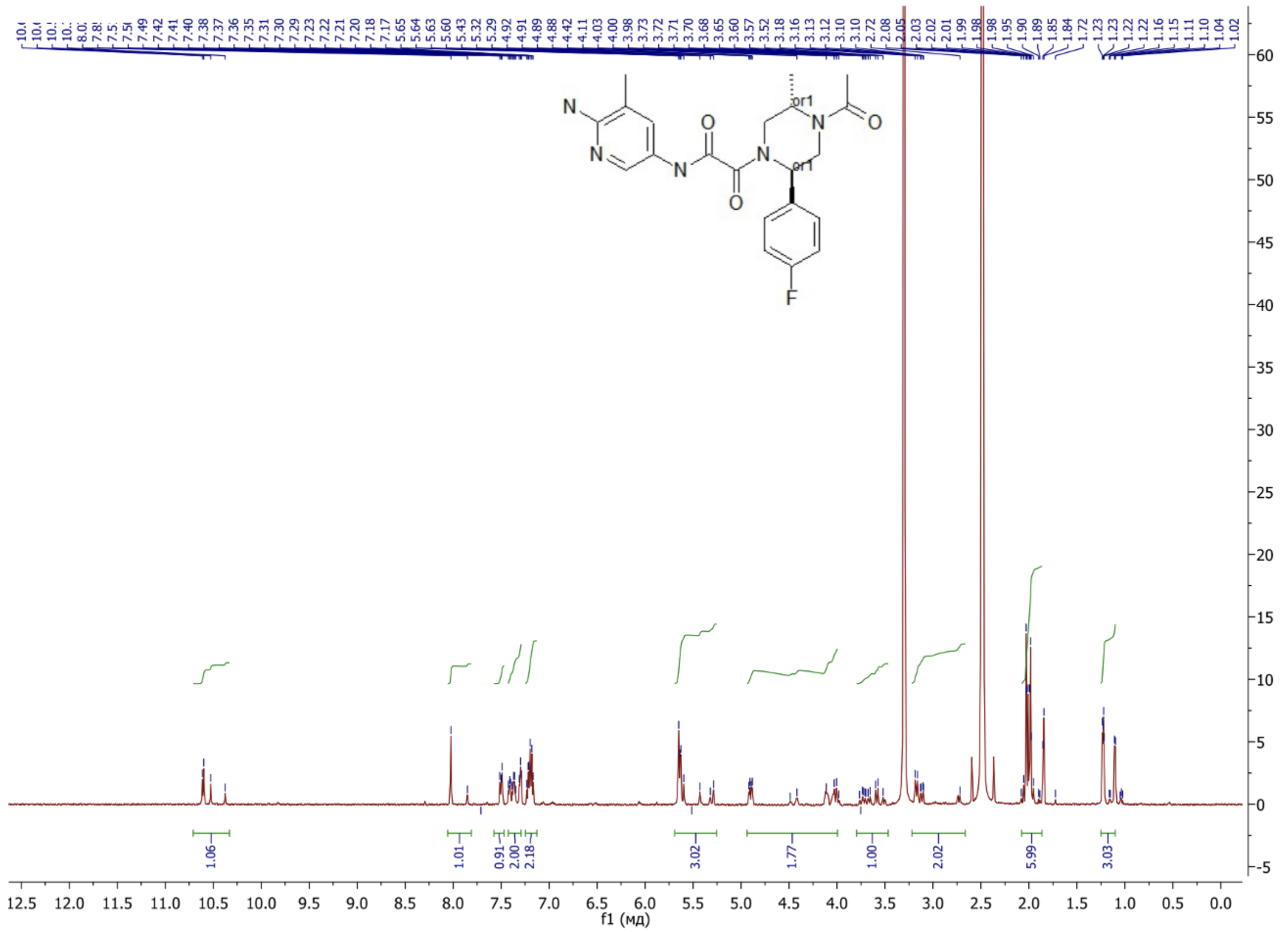

MaxPeak: 100.00%
Ret_Time: 2.061 min

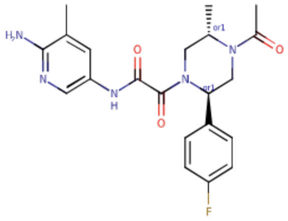

Mol Wt 413.44

Exact Mass 413.21

#	Time	Area%
1	2.061	100.00

RT 2.085

RT 2.084

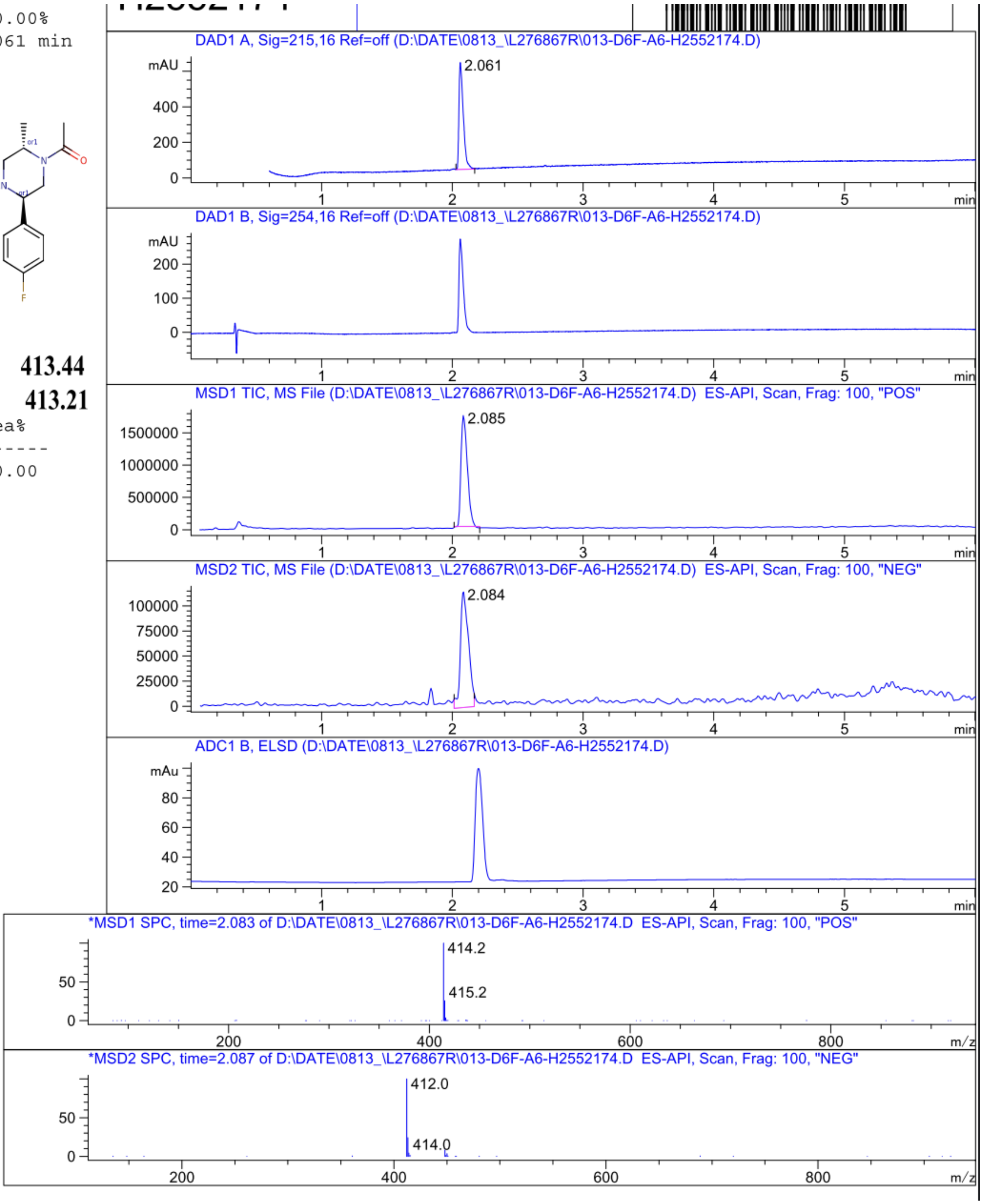

COMPOUND 27

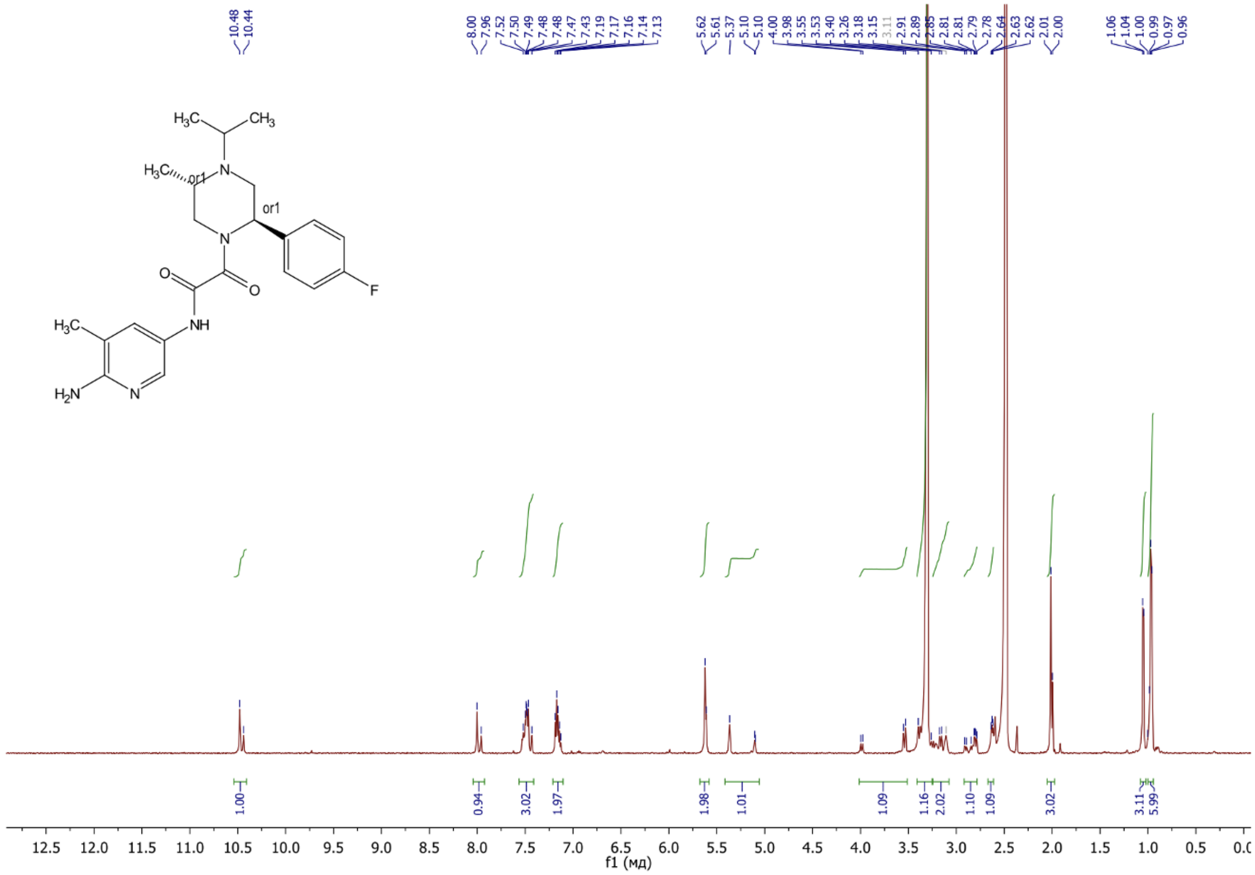

MaxPeak: 100.00%
Ret_Time: 1.654 min

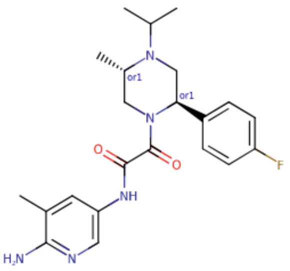

Mol Wt 413.49

Exact Mass 413.26

#	Time	Area%
1	1.654	100.00

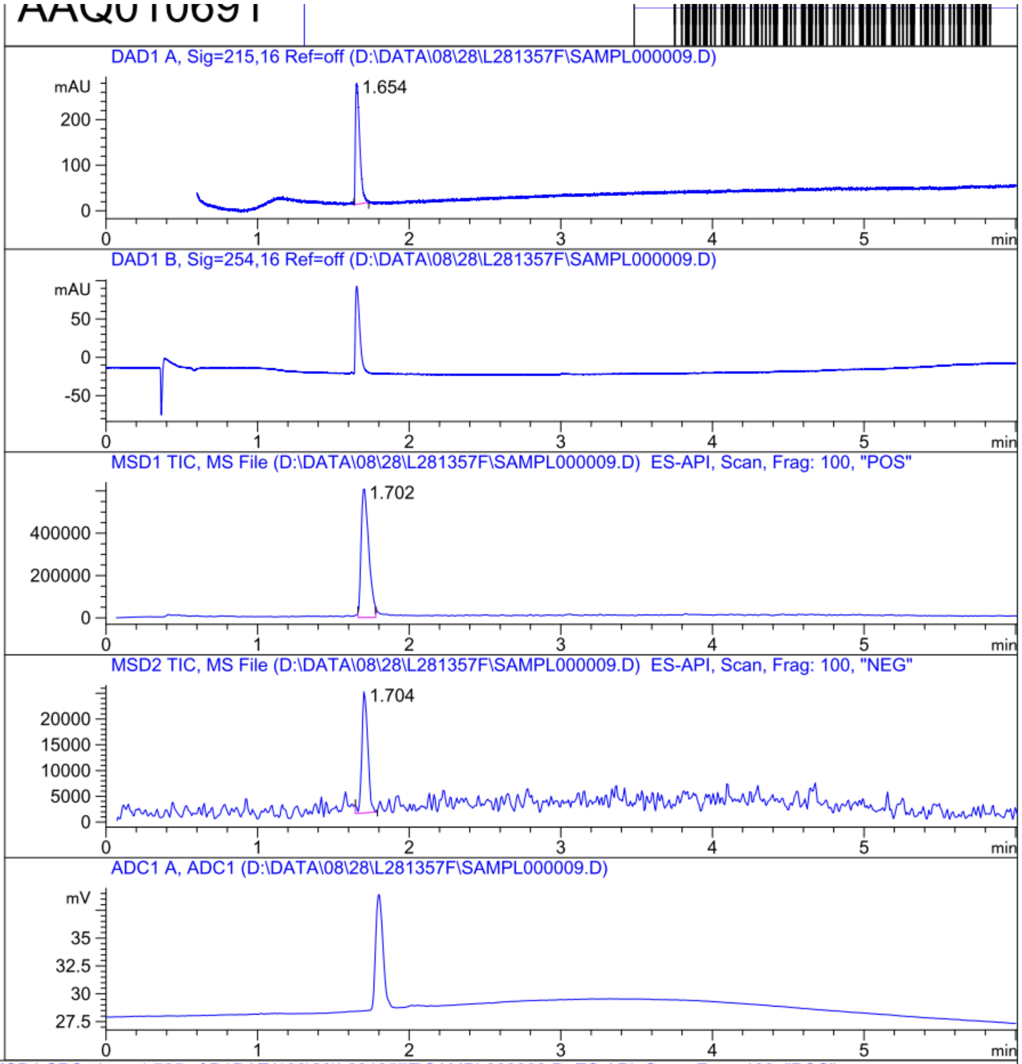

RT 1.702

RT 1.704

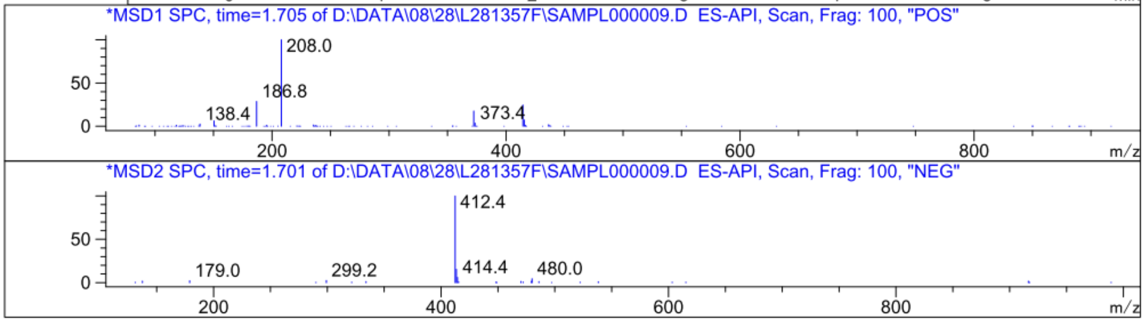

COMPOUND 28

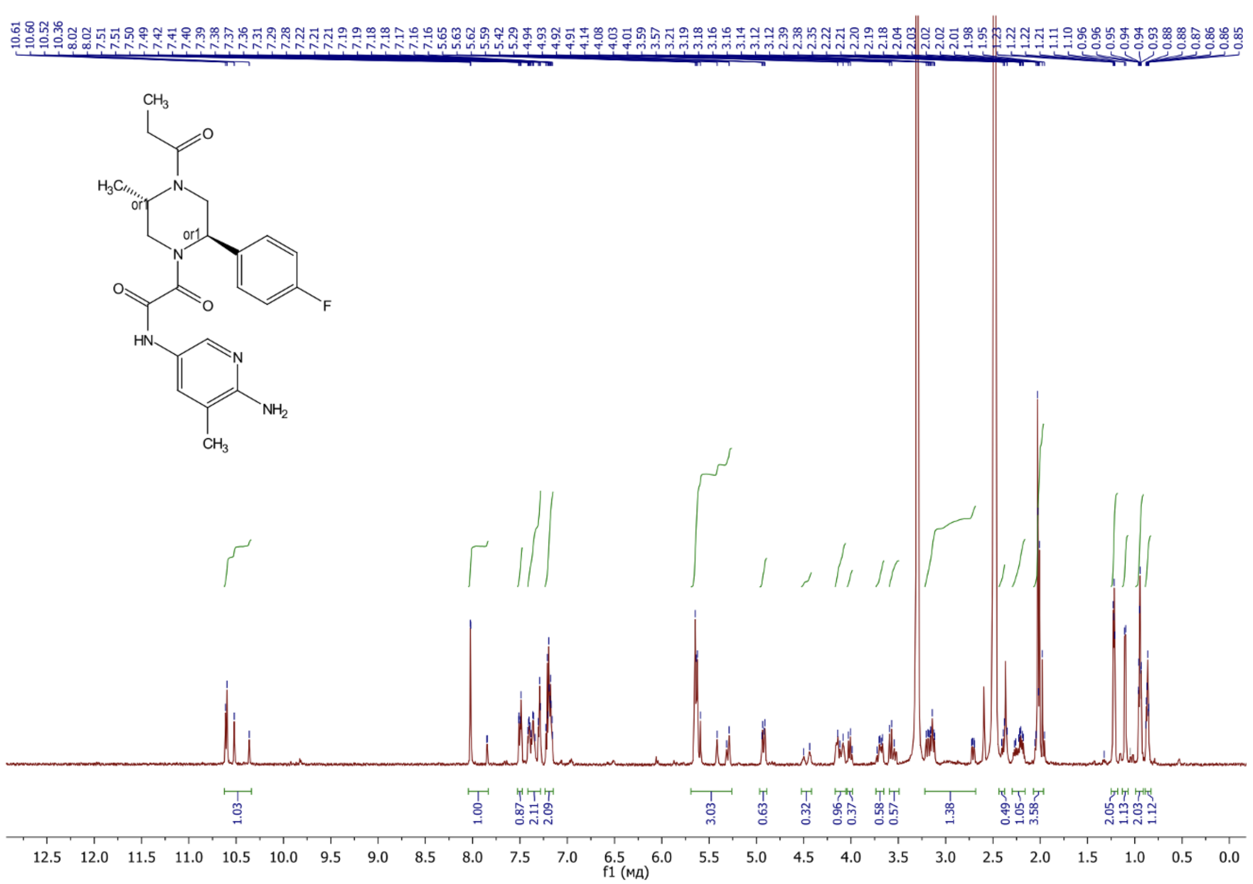

MaxPeak: 97.99%
Ret_Time: 2.217 min

H2624701

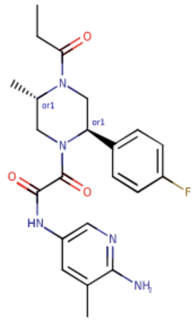

Mol Wt 427.47

Exact Mass 427.23

#	Time	Area%
1	1.817	2.01
2	2.217	97.99

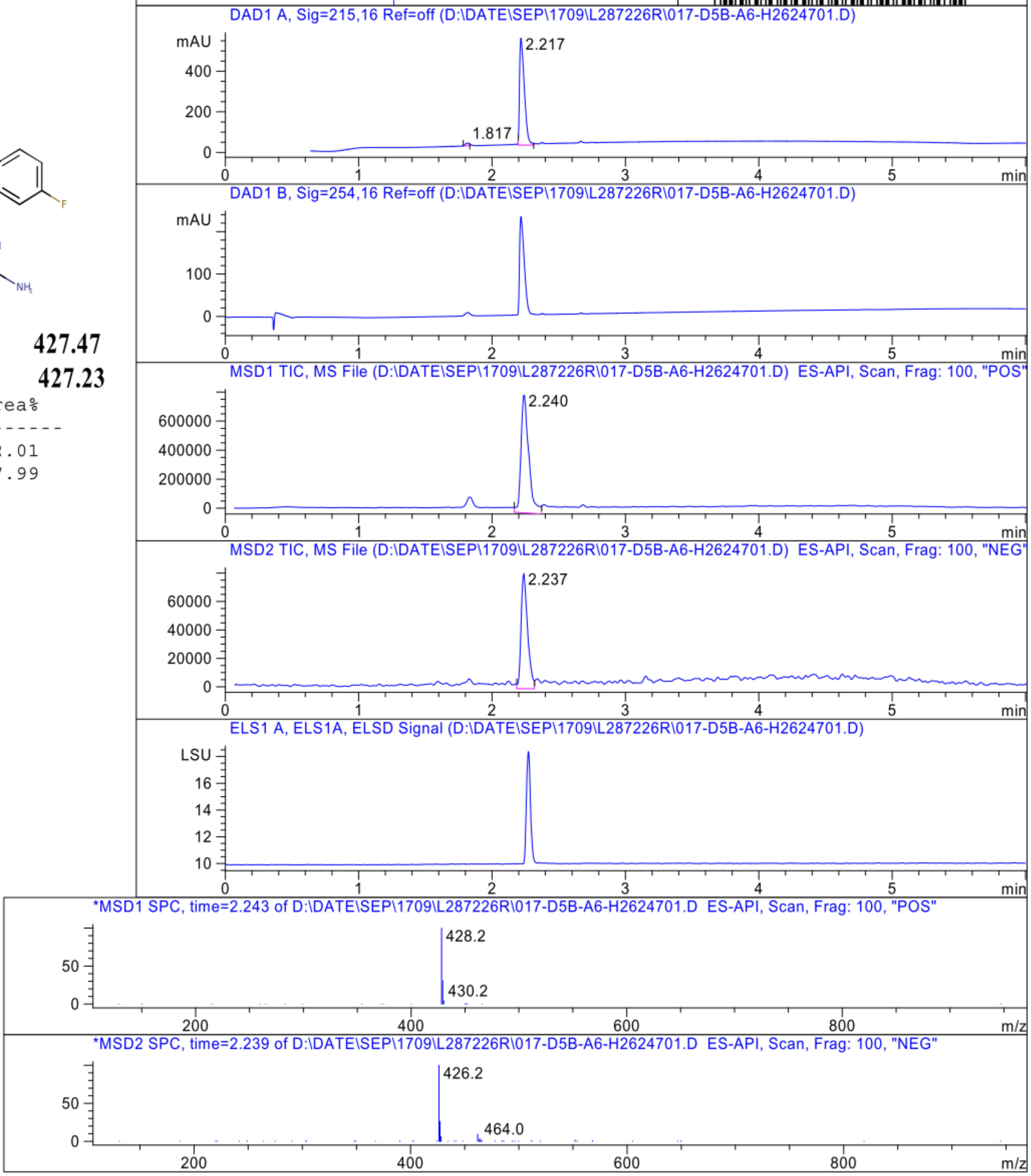

RT 2.240

RT 2.237

COMPOUND 29

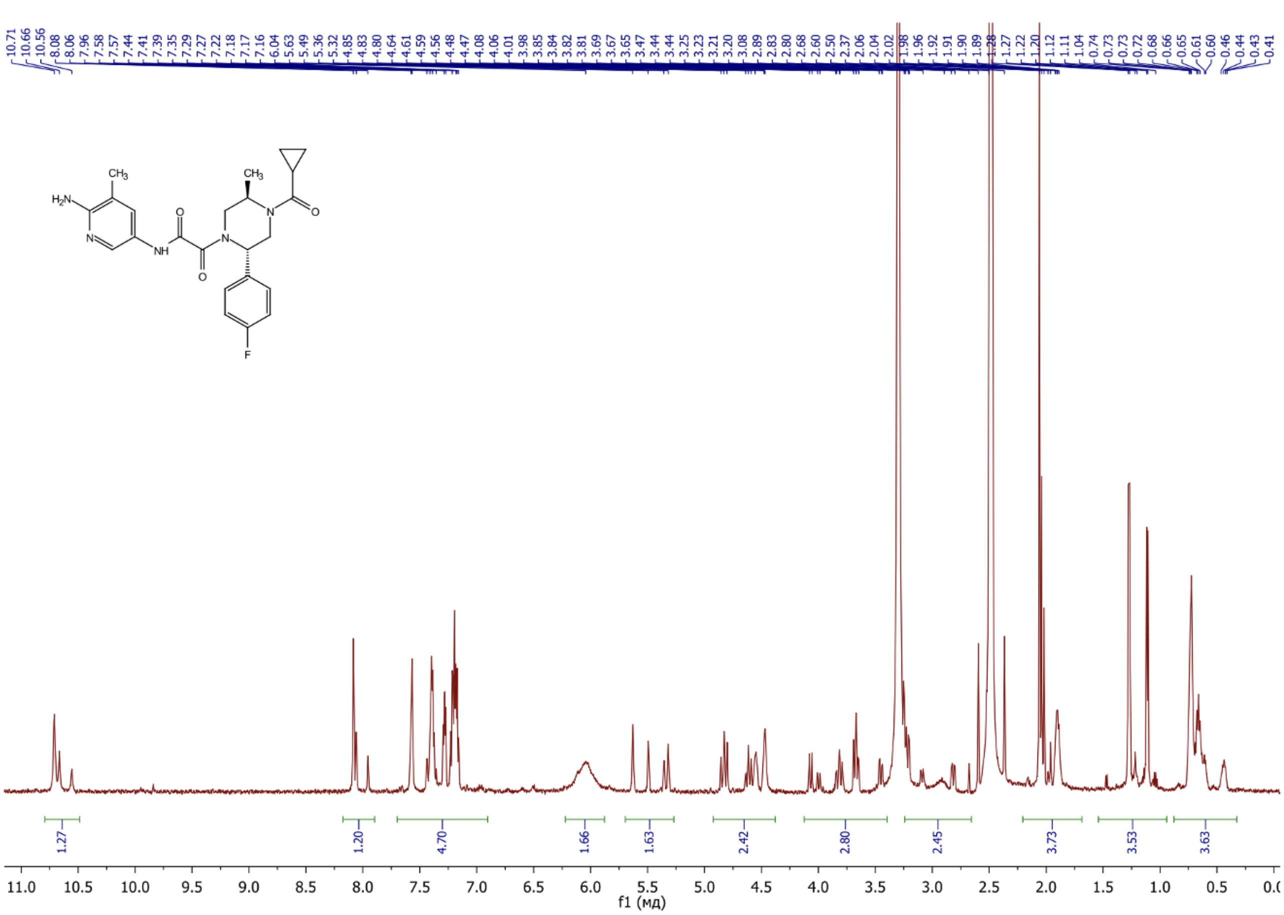
10.71 10.66 10.56 8.08 8.06 7.96 7.58 7.57 7.44 7.41 7.39 7.35 7.29 7.27 7.22 7.18 7.17 7.16 6.04 5.63 5.49 5.36 5.32 4.85 4.83 4.80 4.64 4.61 4.59 4.56 4.48 4.47 4.08 4.06 4.01 3.98 3.85 3.84 3.82 3.81 3.69 3.67 3.65 3.47 3.44 3.25 3.23 3.21 3.20 3.08 2.89 2.83 2.80 2.68 2.60 2.50 2.37 2.06 2.04 2.02 1.98 1.96 1.92 1.91 1.90 1.89 1.28 1.27 1.22 1.20 1.12 1.11 1.04 0.74 0.73 0.73 0.72 0.68 0.66 0.65 0.61 0.60 0.46 0.44 0.43 0.41
CH3
H2N
N
NH
O
O
CH3
N
N
O
F
1.27
1.20
4.70
1.66
1.63
2.42
2.80
2.45
3.73
3.53
3.63
11.0 10.5 10.0 9.5 9.0 8.5 8.0 7.5 7.0 6.5 6.0 5.5 5.0 4.5 4.0 3.5 3.0 2.5 2.0 1.5 1.0 0.5
f1 (мд)

MaxPeak: 100.00%
Ret_Time: 2.082 min

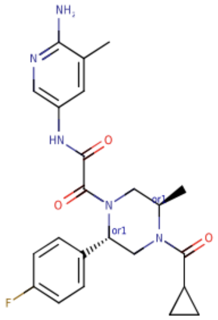

Mol Wt 439.48

Exact Mass 439.23

#	Time	Area%
1	2.082	100.00

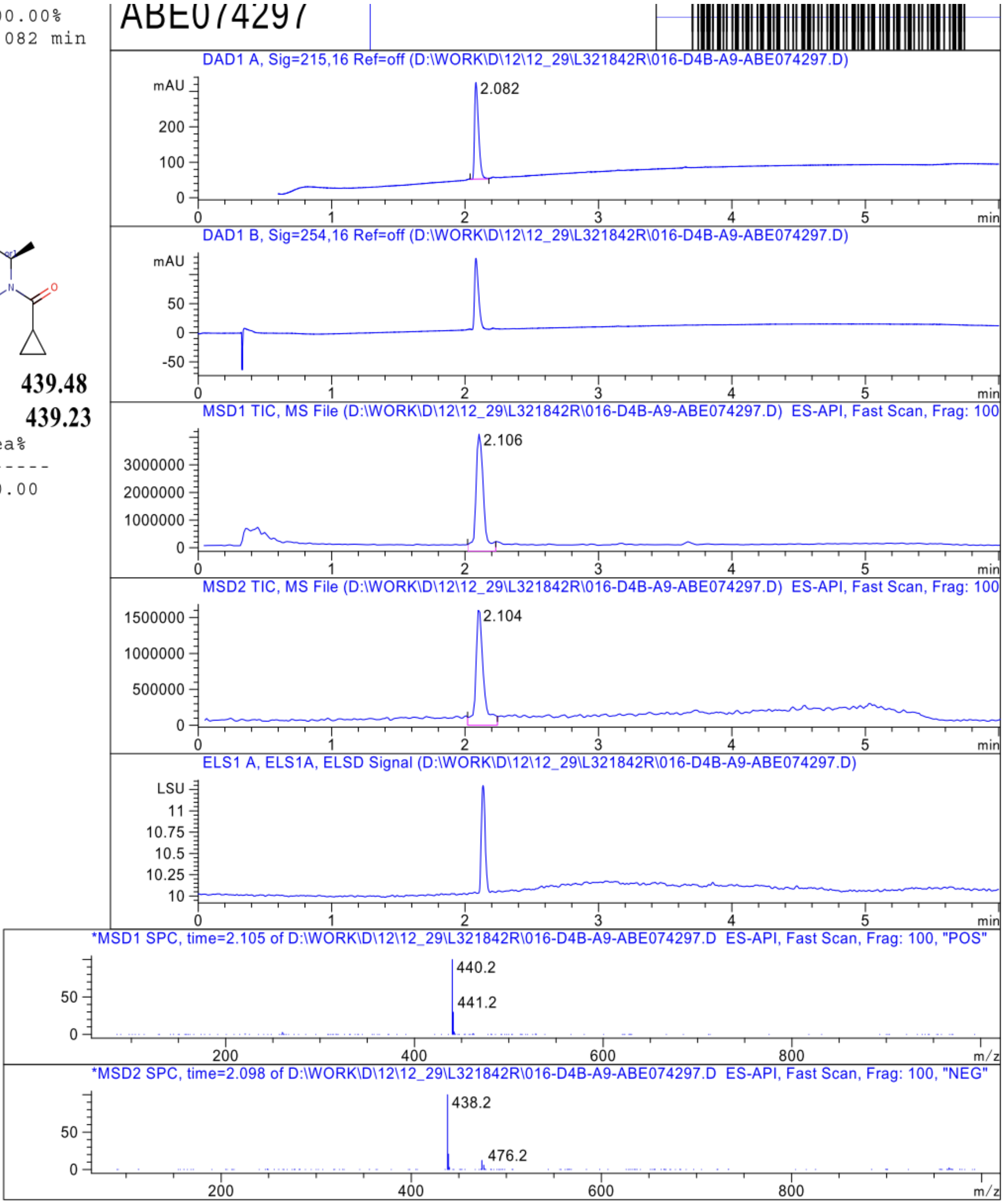

RT 2.106

RT 2.104

COMPOUND 30

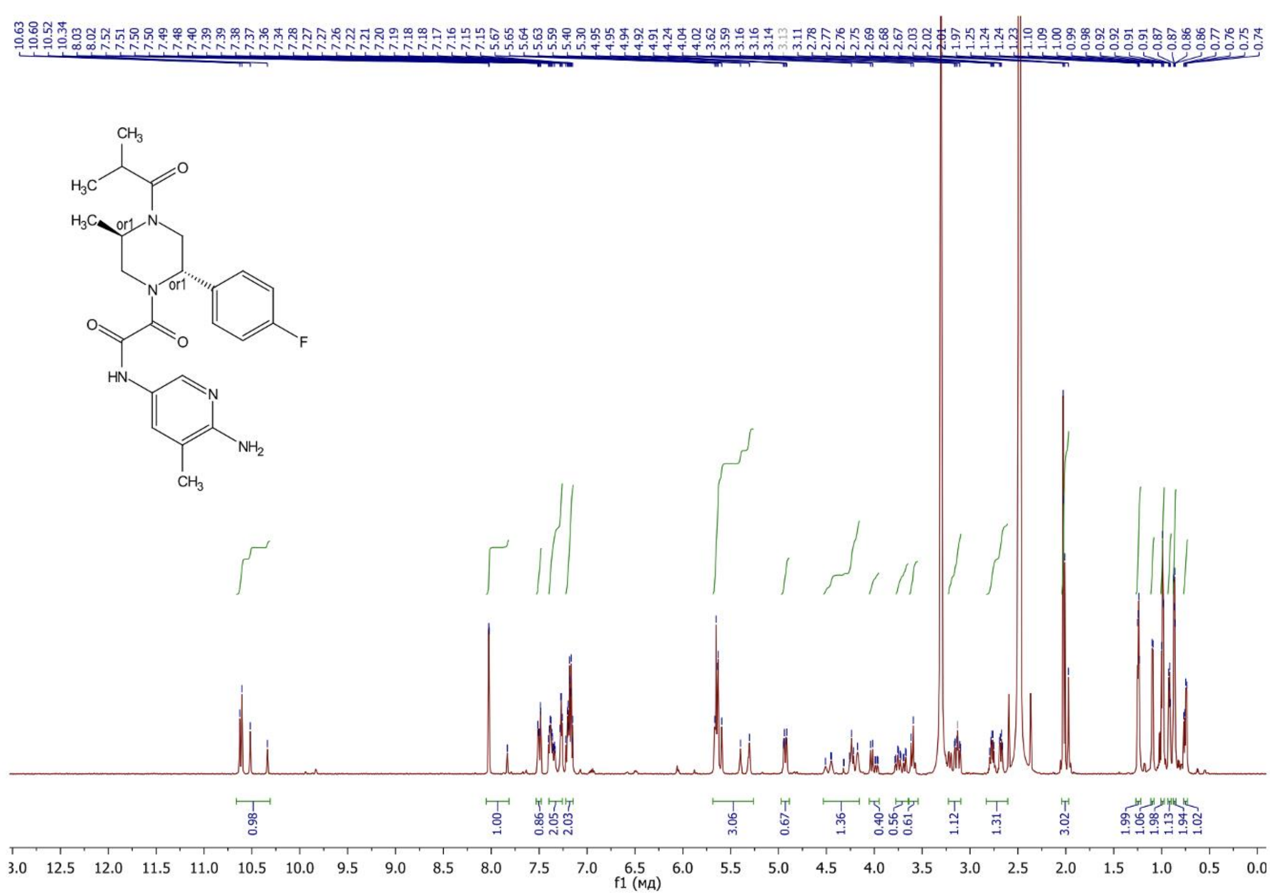

MaxPeak: 100.00%
Ret_Time: 2.179 min

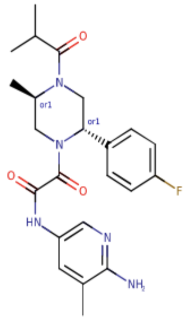

Mol Wt 441.5

Exact Mass 441.25

#	Time	Area%
1	2.179	100.00

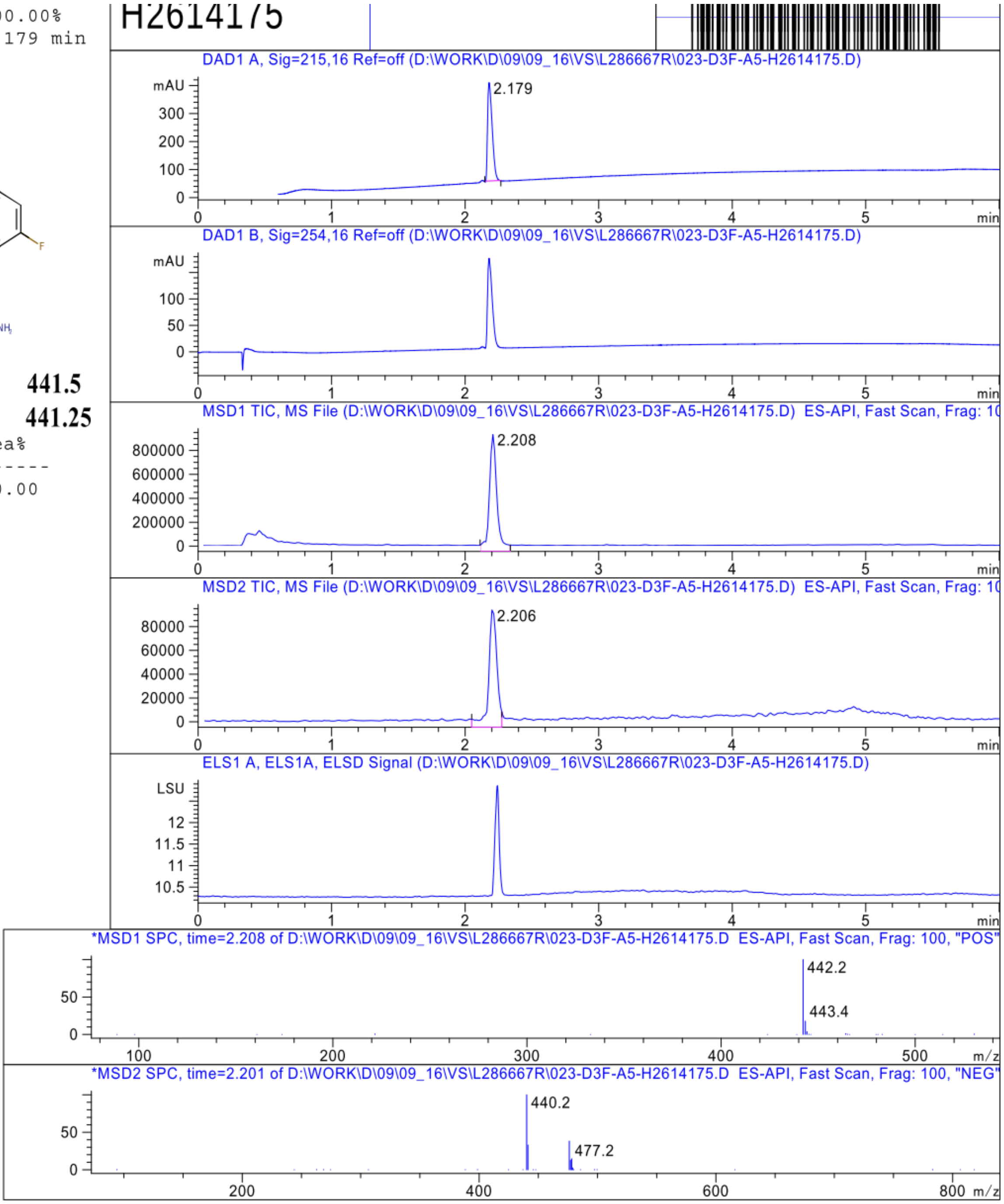

RT 2.208

RT 2.206

COMPOUND 31

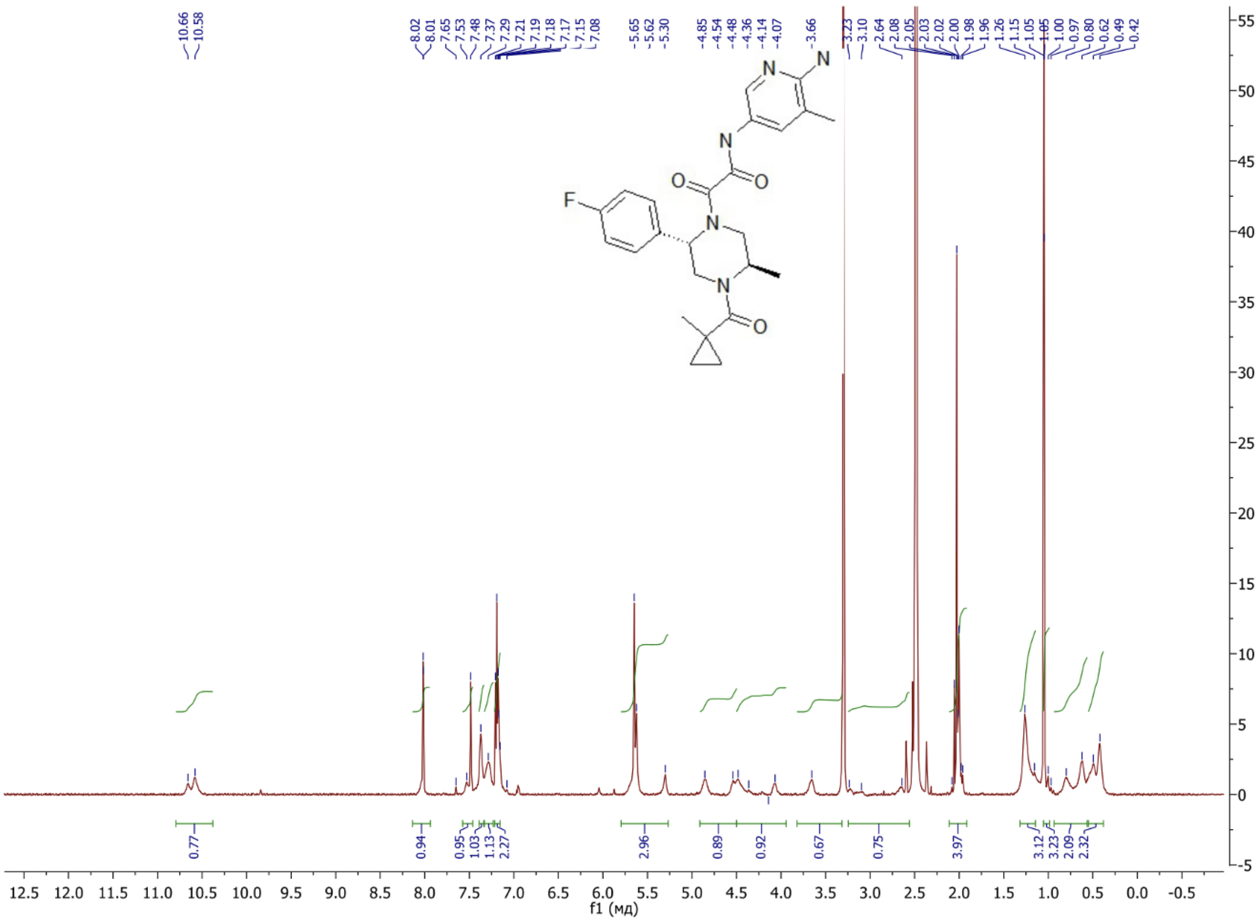
f1 (мд)

MaxPeak: 100.00%
Ret_Time: 2.521 min

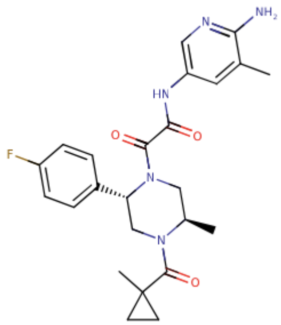

Mol Wt 453.51

Exact Mass 453.25

#	Time	Area%
1	2.521	100.00

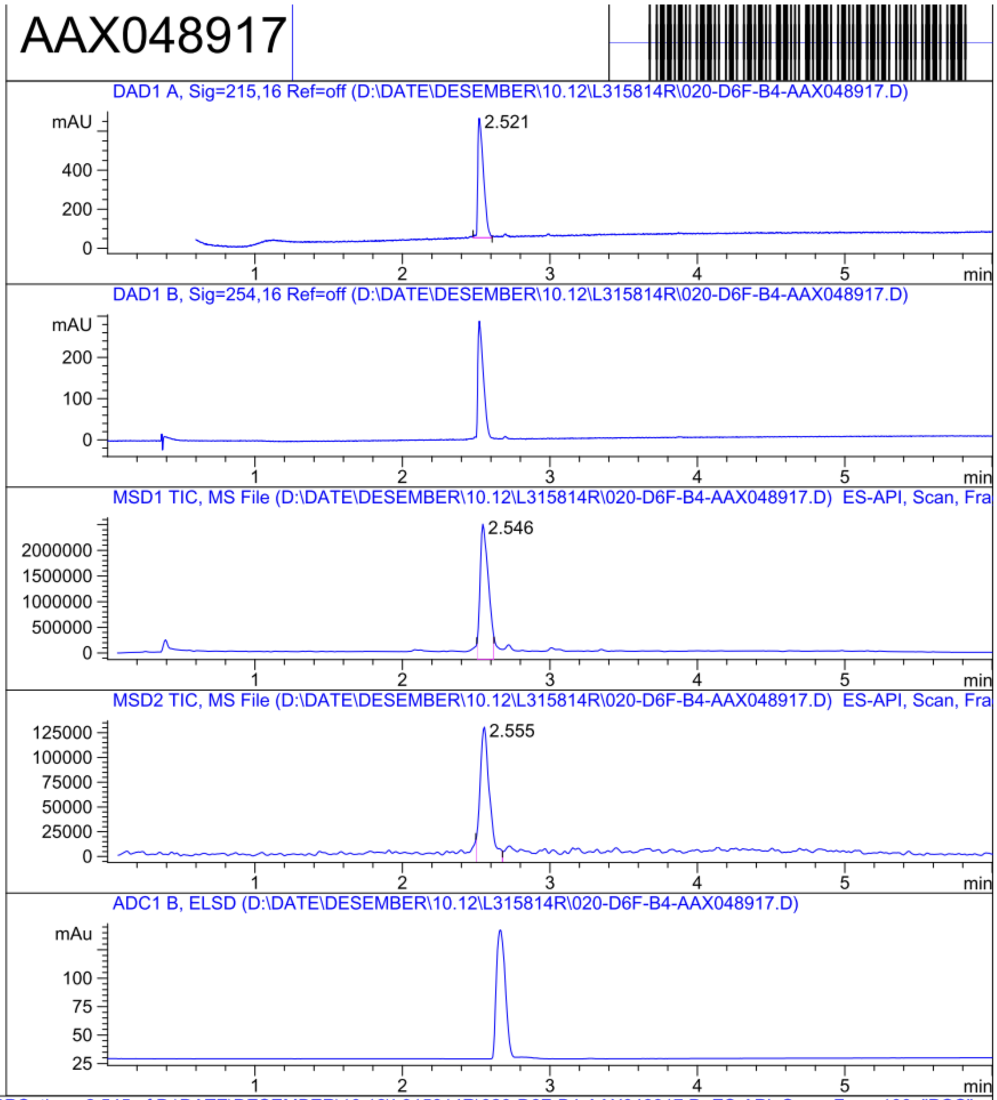

RT 2.546

RT 2.555

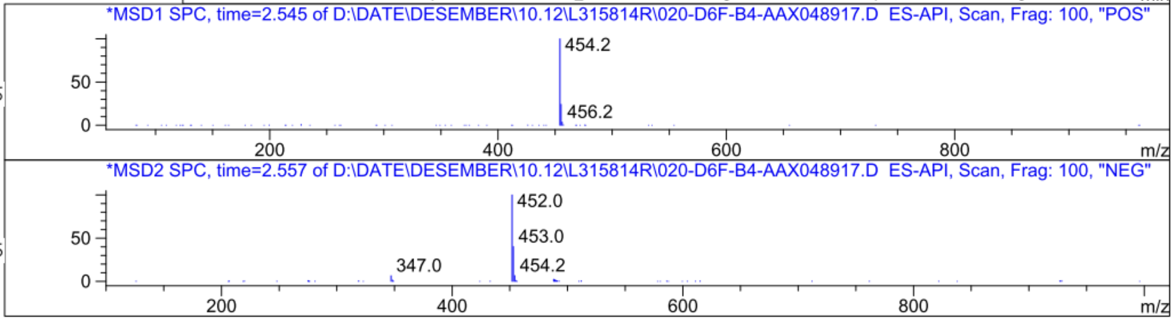

COMPOUND 32

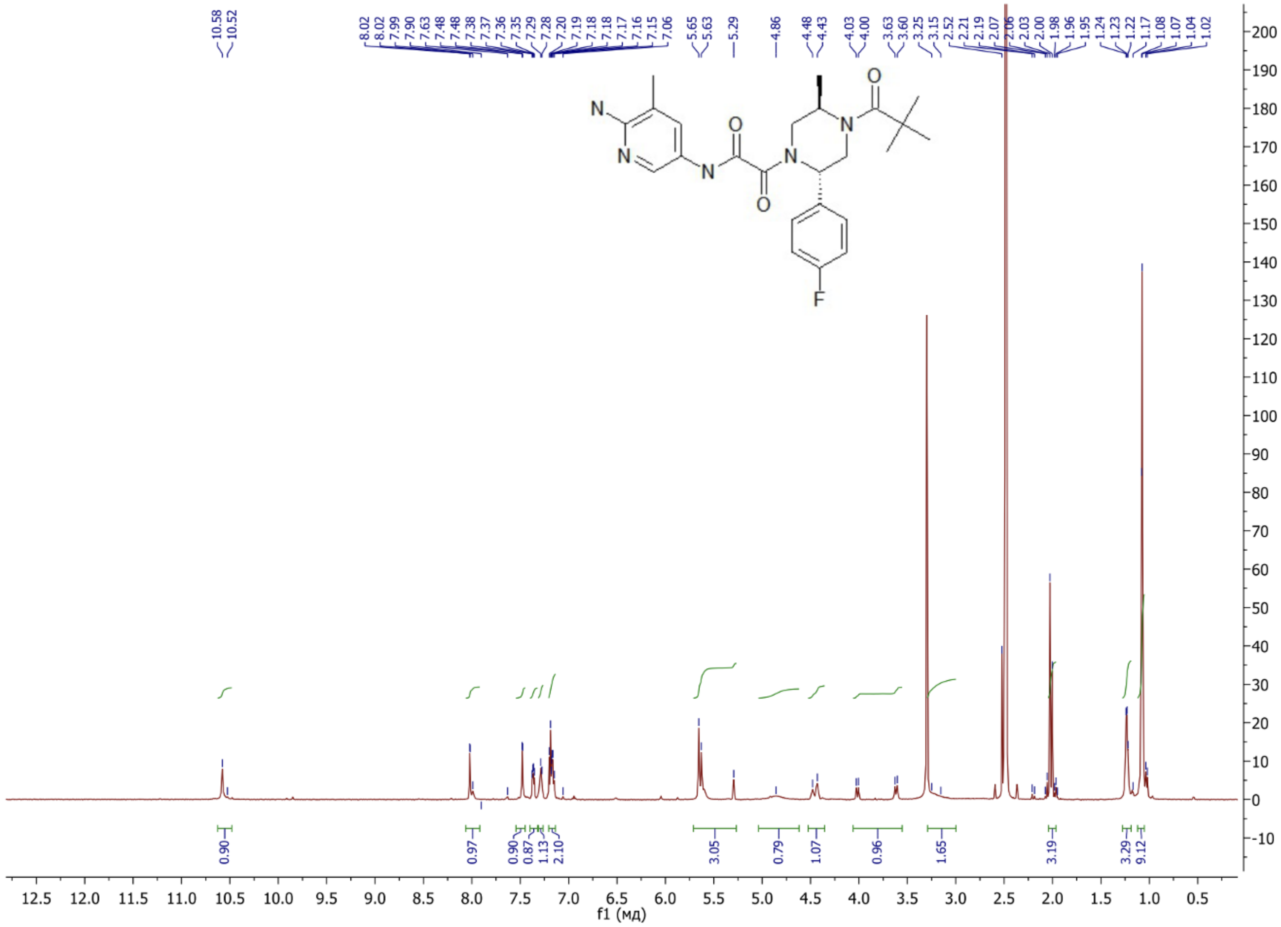

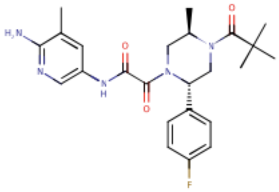

Mol Wt 455.52

Exact Mass 455.27

#	Time	Area%
1	2.798	92.53
2	2.920	3.33
3	3.043	4.14

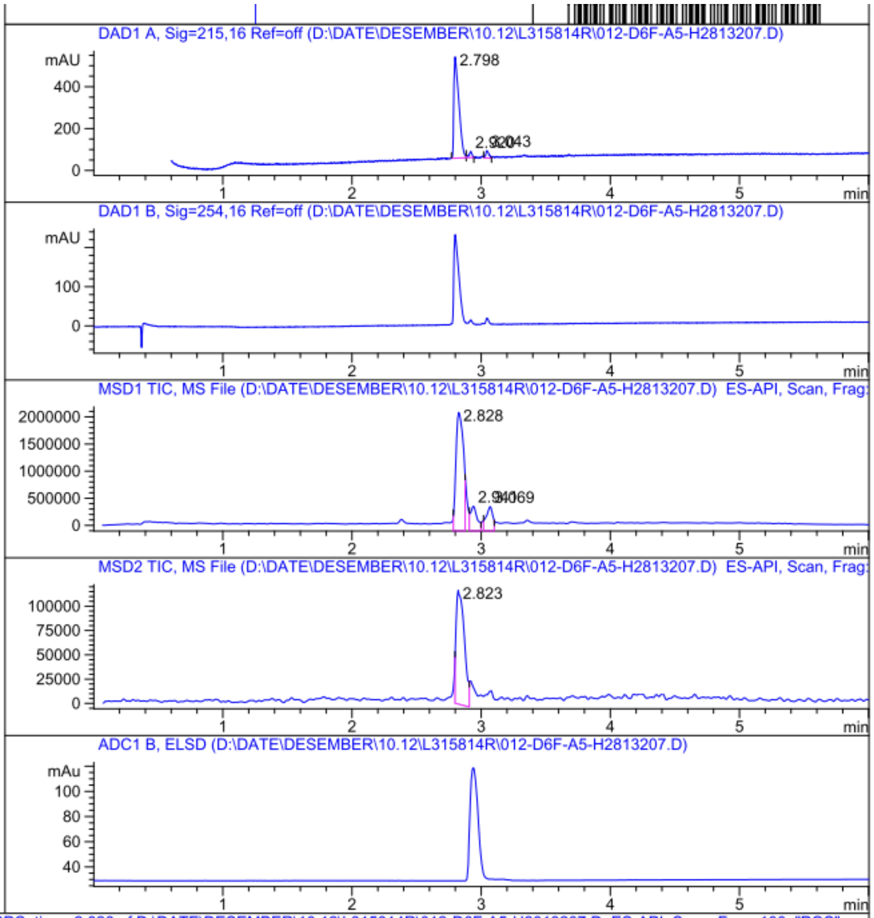

RT 2.828

RT 2.941

RT 3.069

RT 2.823

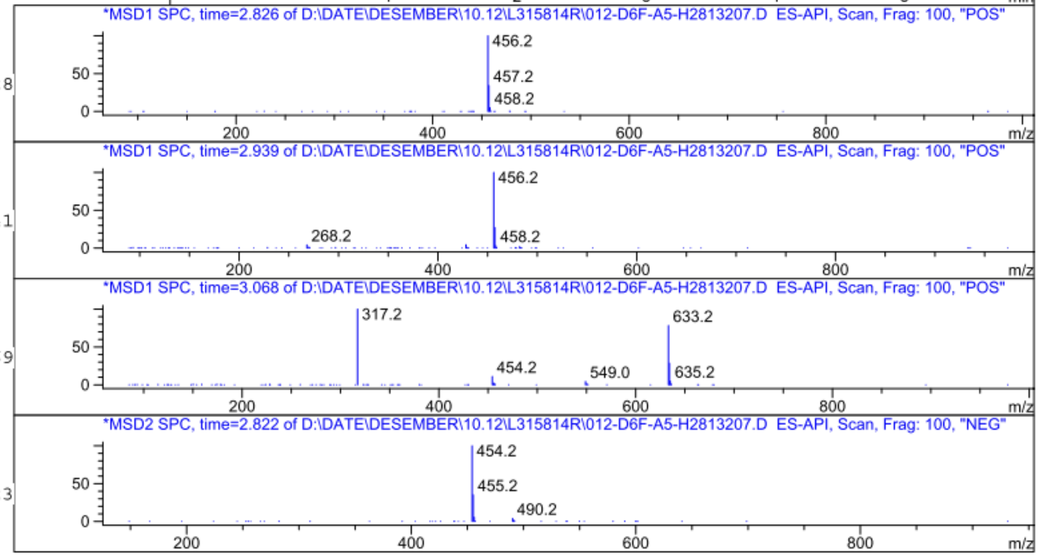

COMPOUND 33

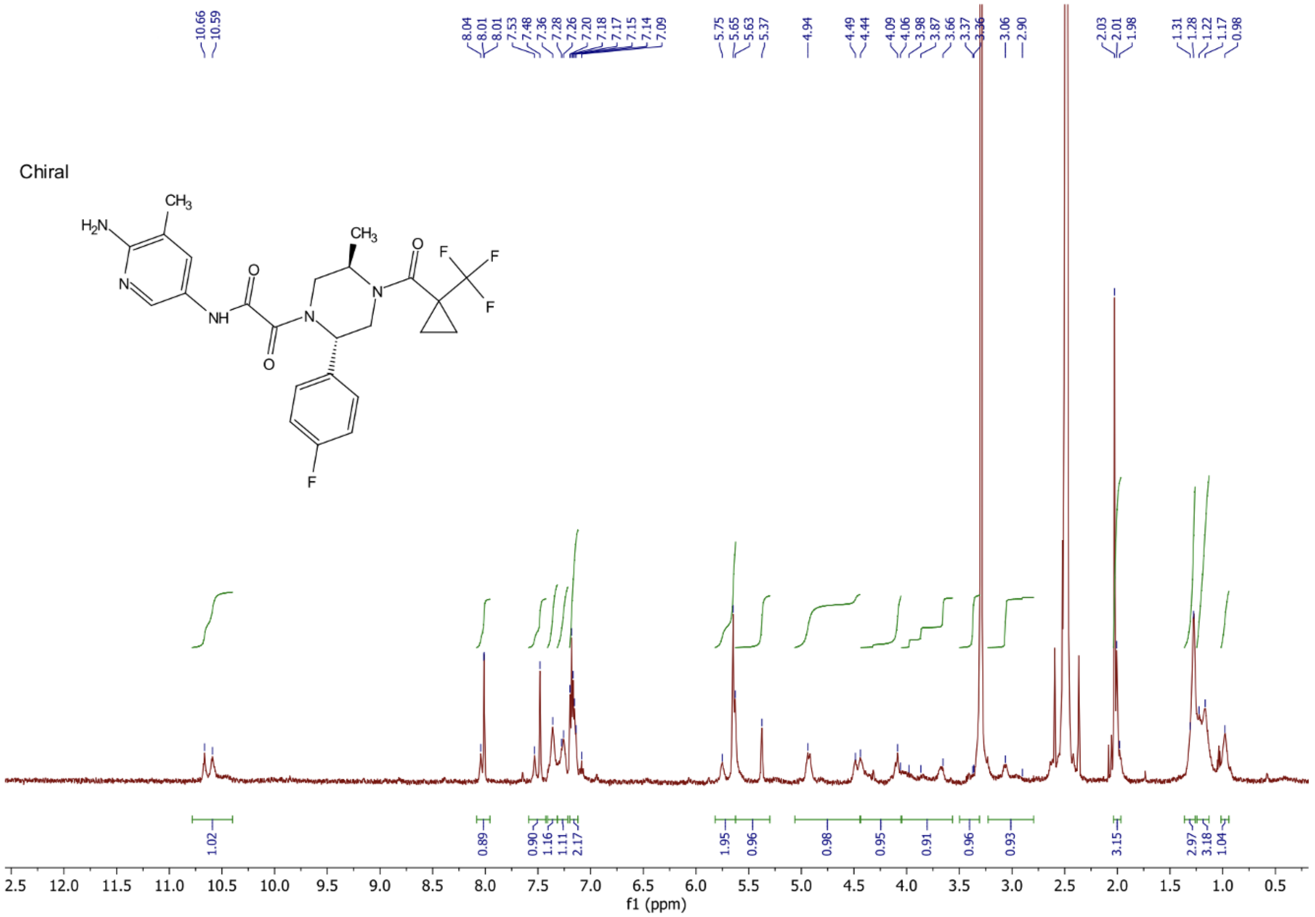

```
MaxPeak:  98.38%
Ret_Time: 2.496 min
```

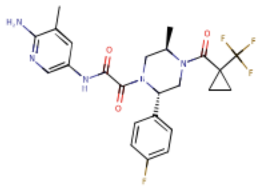

Mol Wt 507.48

Exact Mass 507.22

#	Time	Area%
1	2.043	1.62
2	2.496	98.38

RT 2.068

RT 2.526

RT 2.527

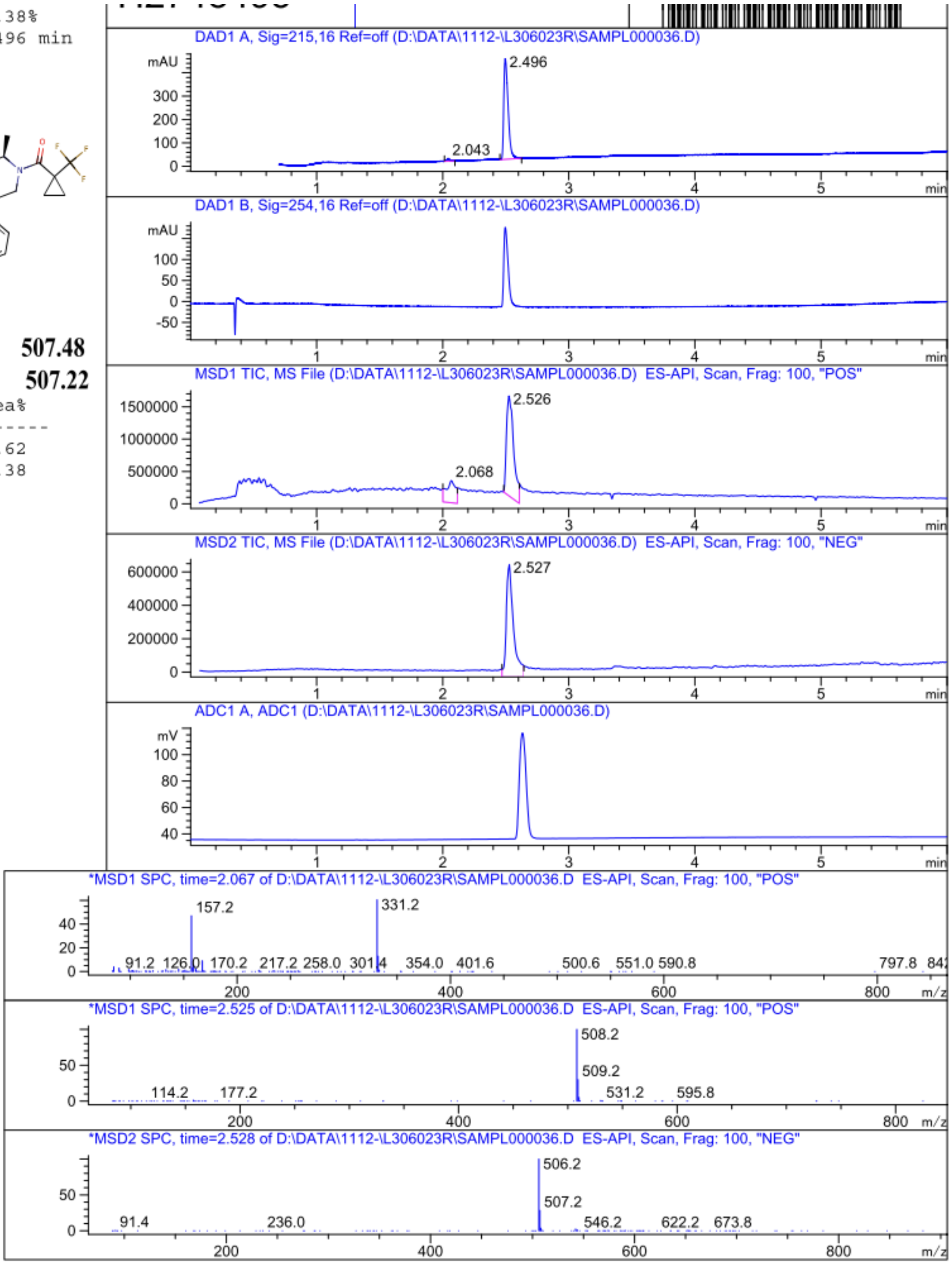

COMPOUND 34

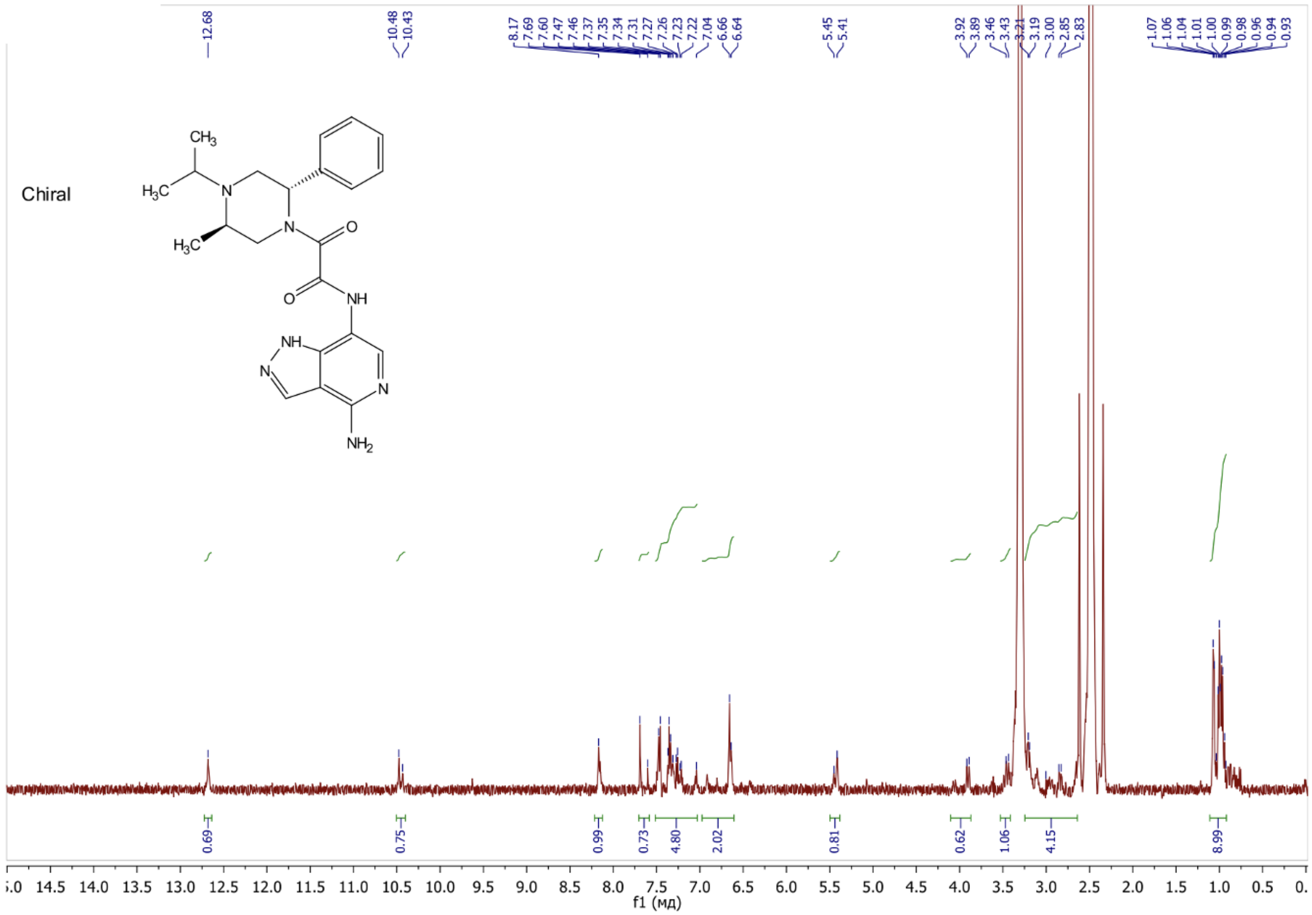
Chiral
f1 (мд)

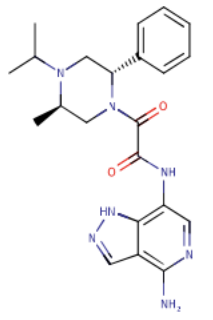

Mol Wt 421.5

Exact Mass 421.25

#	Time	Area%
1	1.377	1.16
2	1.510	96.38
3	2.708	2.46

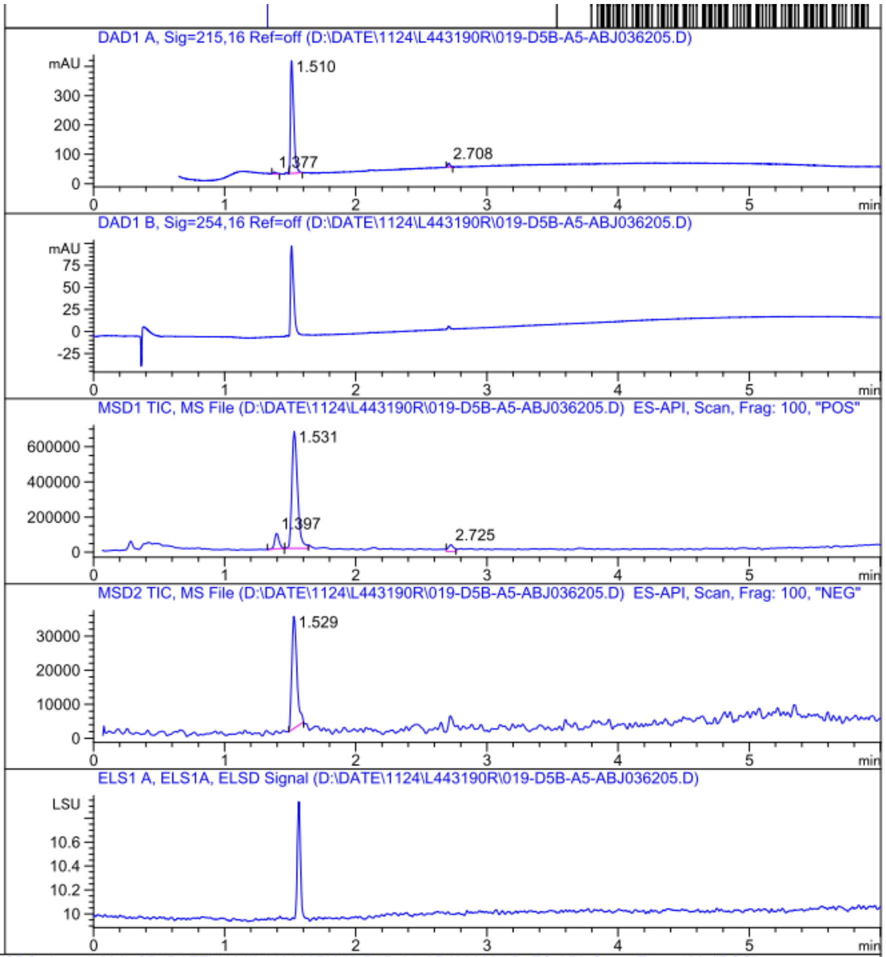

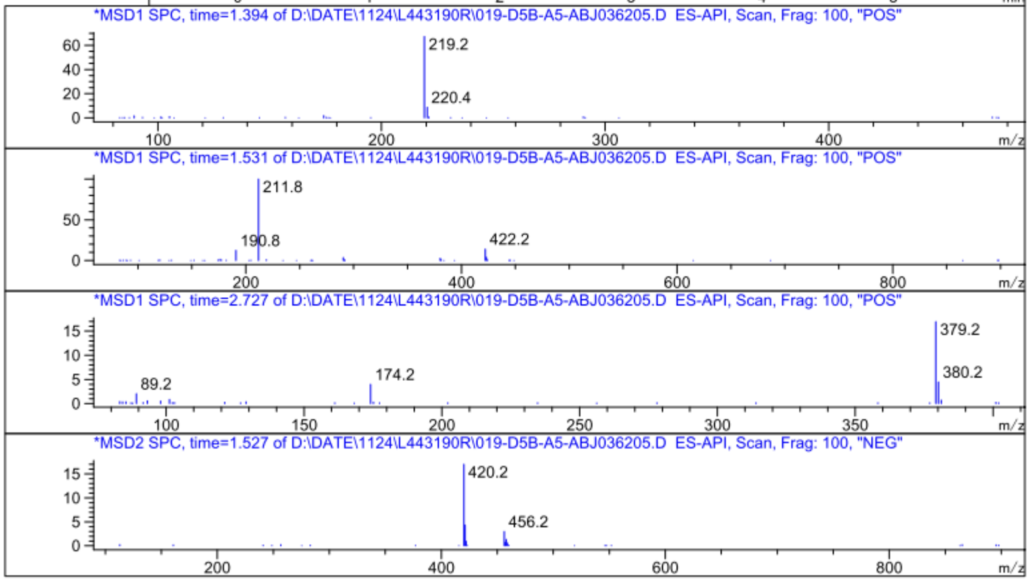

RT 1.397

RT 1.531

RT 2.725

RT 1.529

COMPOUND 35

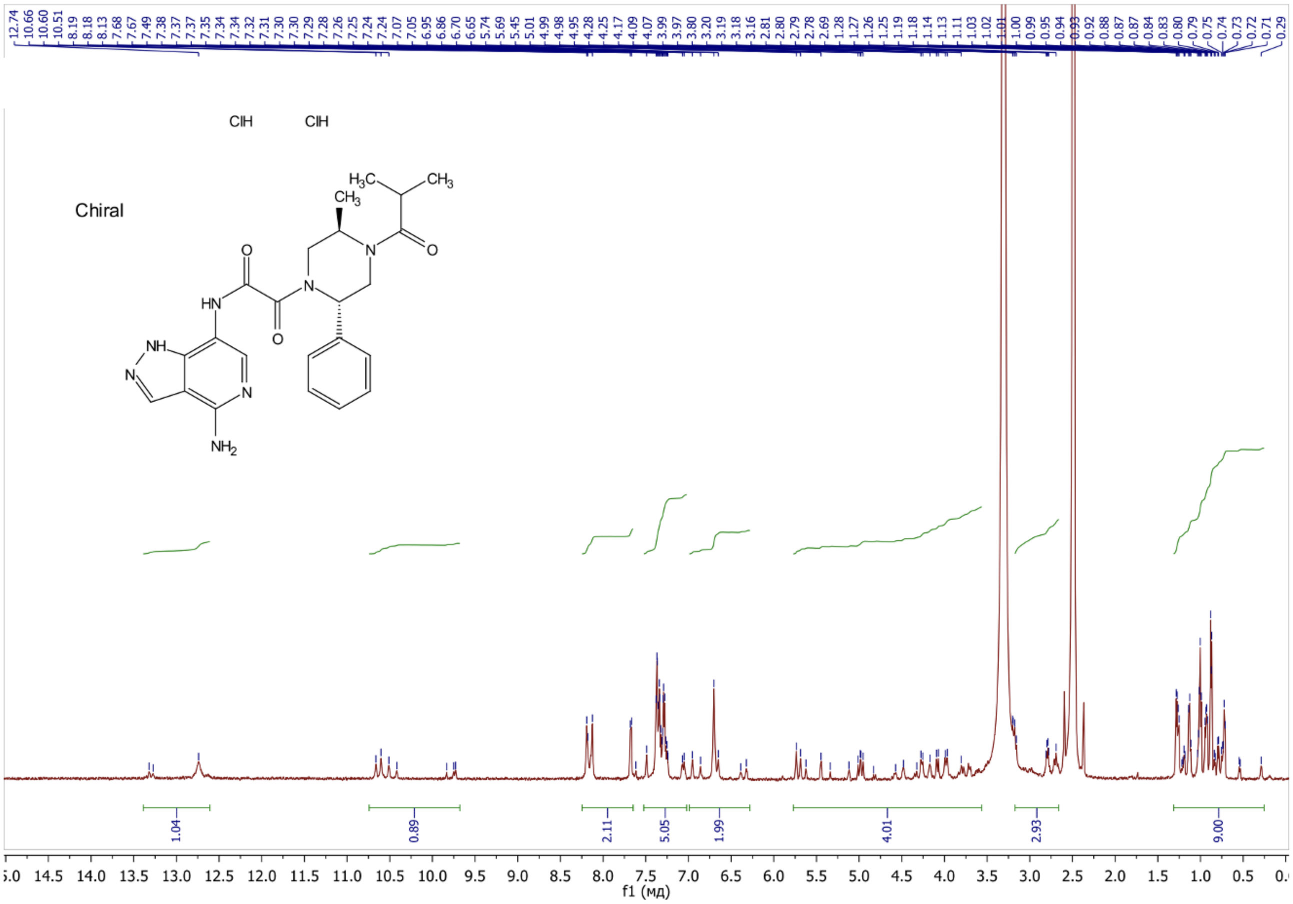
Chiral
ClH
ClH
f1 (мд)

MaxPeak: 97.61%
Ret_Time: 2.023 min

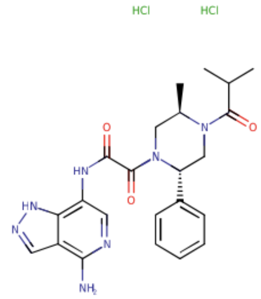

Mol Wt 522.43
Exact Mass 449.24

#	Time	Area%
1	1.956	2.39
2	2.023	97.61

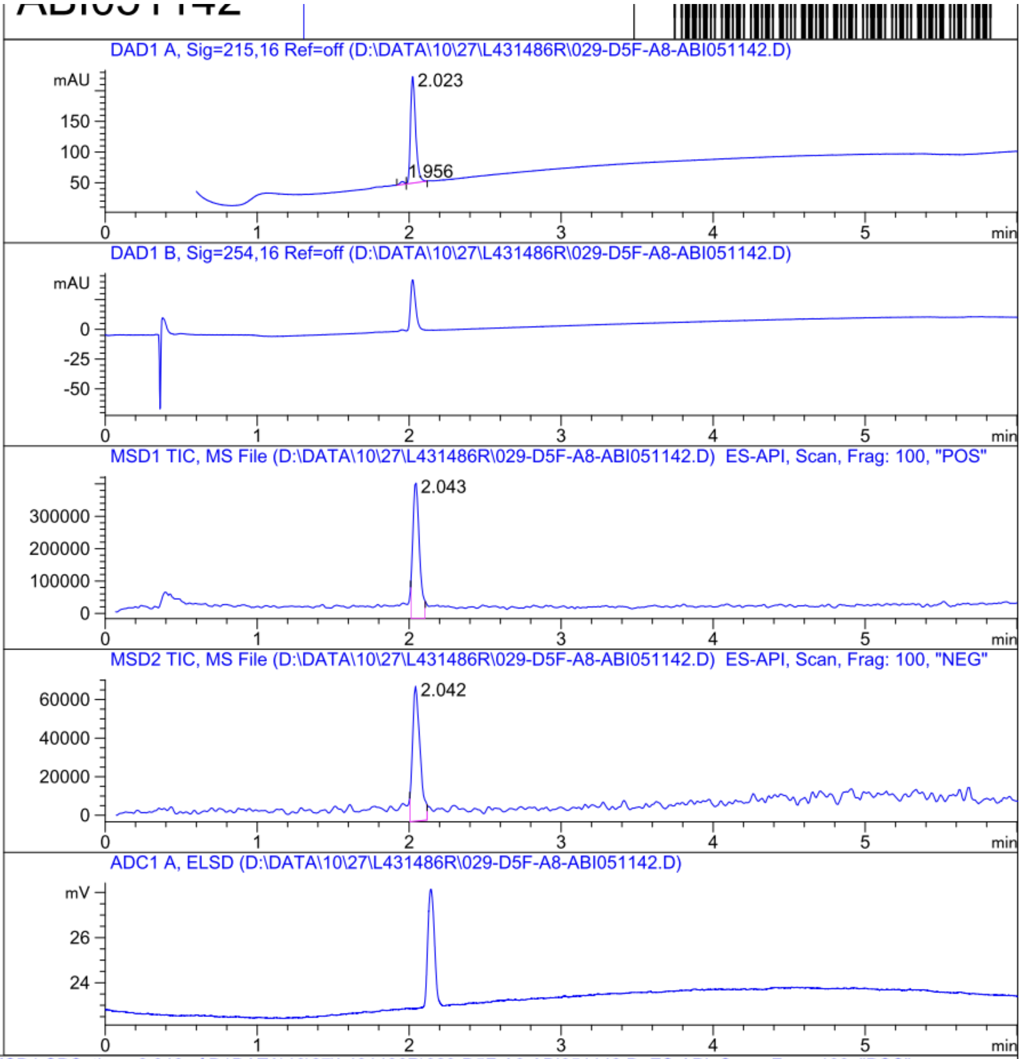

RT 2.043

RT 2.042

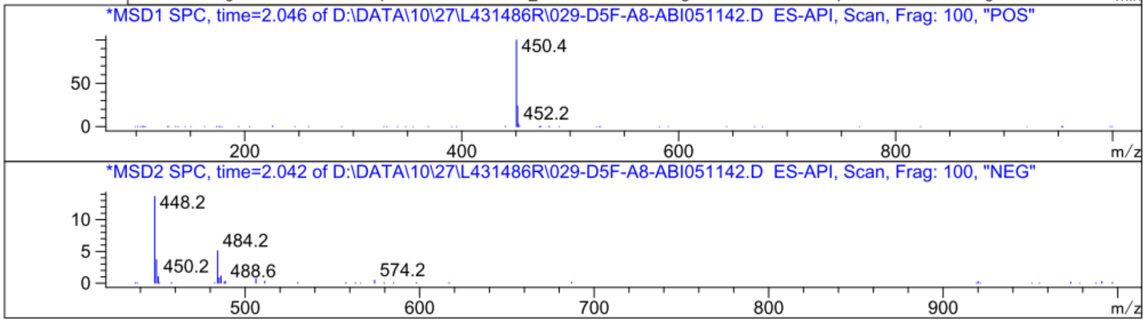

COMPOUND 36

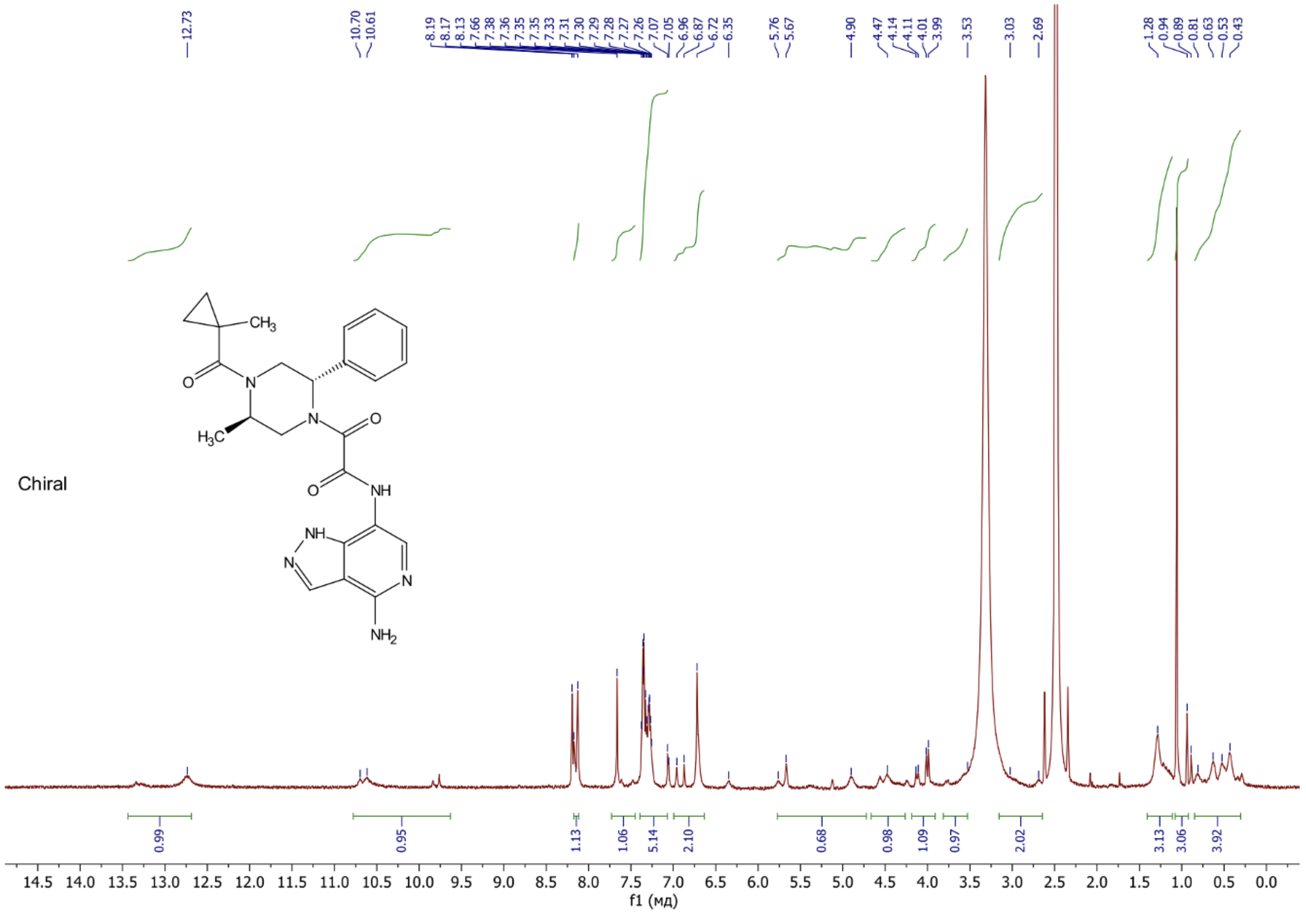
Chiral
f1 (мд)

MaxPeak: 100.00%
Ret_Time: 1.048 min

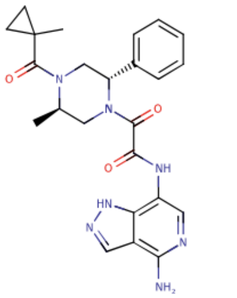

Mol Wt 461.52
Exact Mass 461.24

#	Time	Area%
1	1.048	100.00

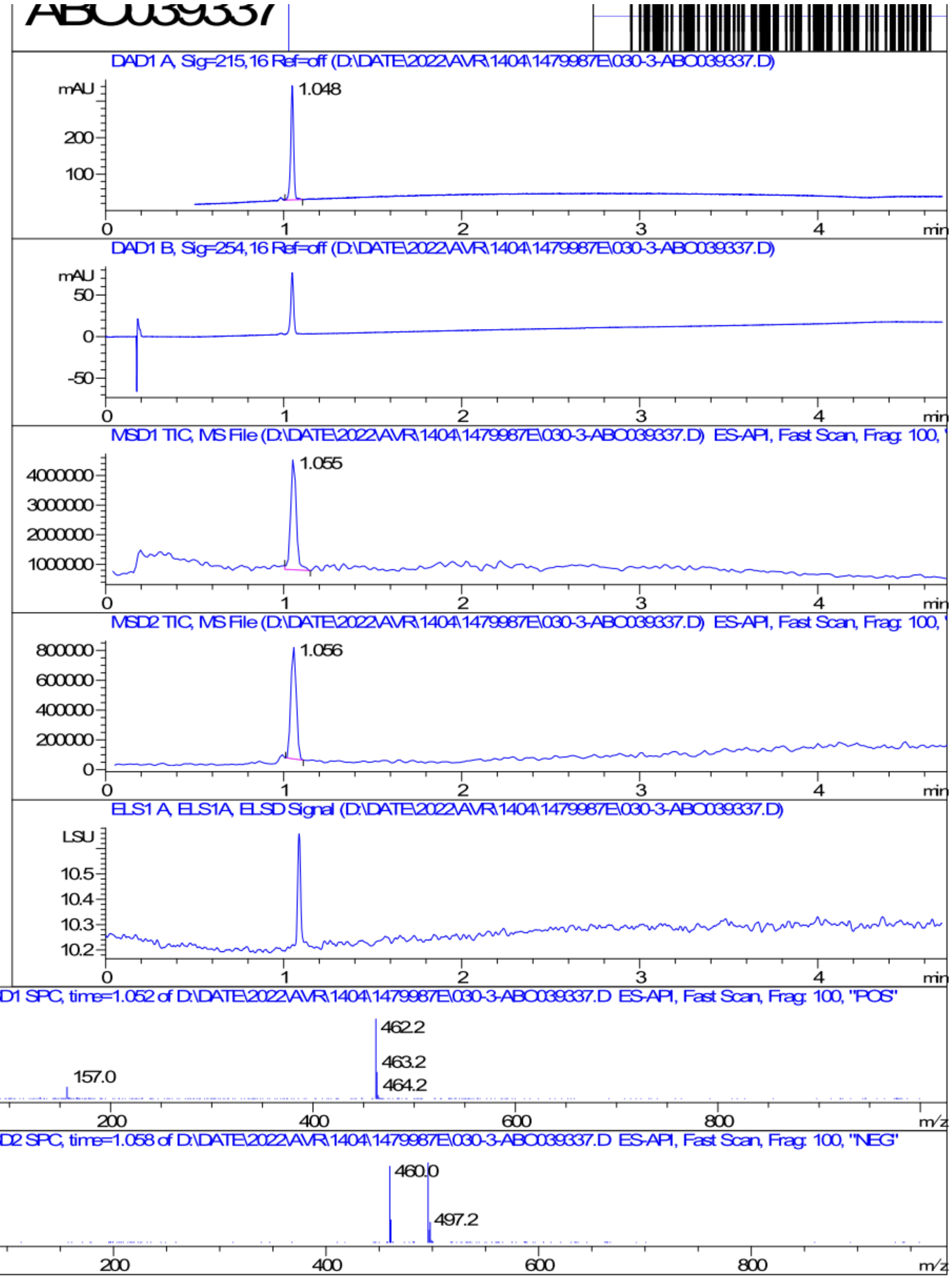

COMPOUND 37

MaxPeak: 100.00%
Ret_Time: 2.529 min

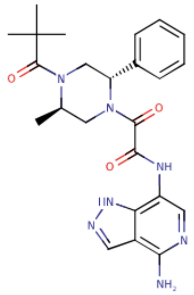

Mol Wt 463.53
Exact Mass 463.26

#	Time	Area%
1	2.529	100.00

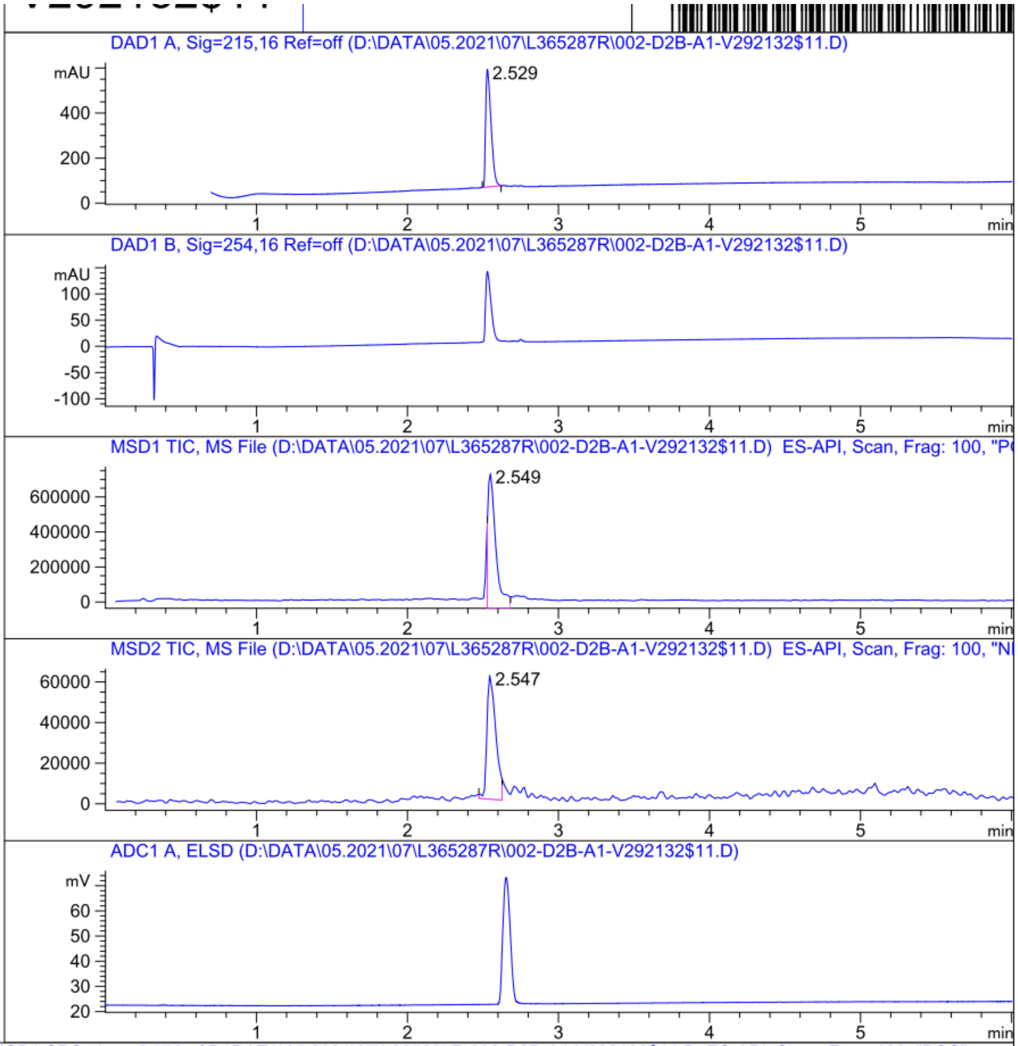

RT 2.549

RT 2.547

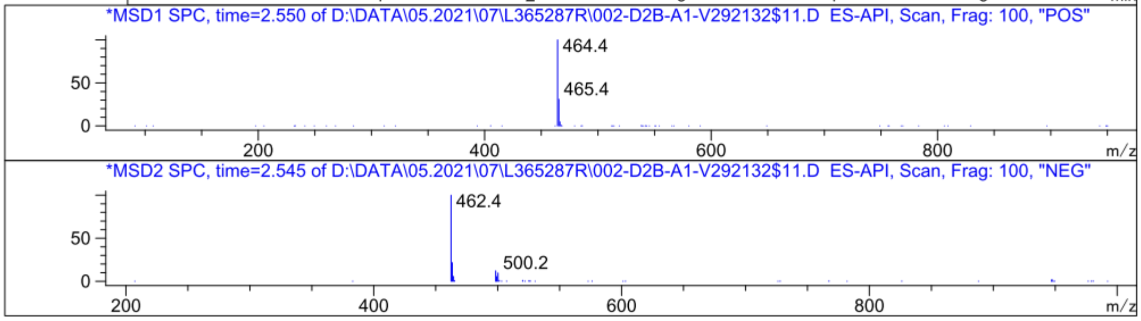

COMPOUND 38

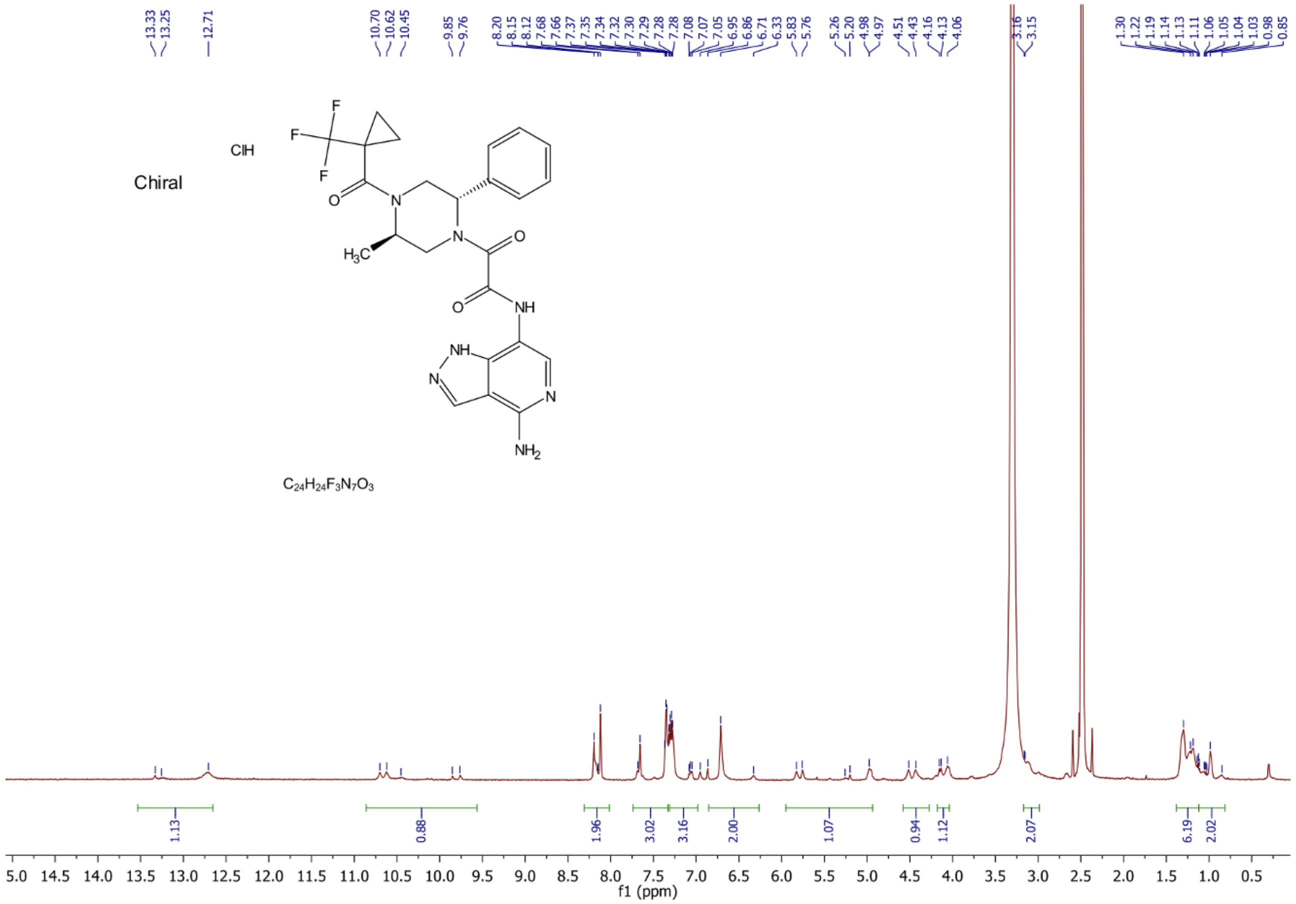
Chiral
ClH
$C_{24}H_{24}F_3N_7O_3$
f1 (ppm)

Ret_Time: 2.004 min

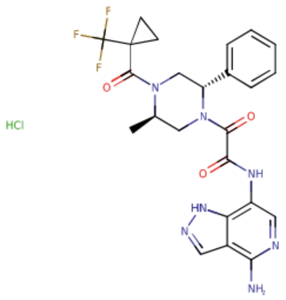

Mol Wt 551.95

Exact Mass 515.21

#	Time	Area%
1	2.004	100.00

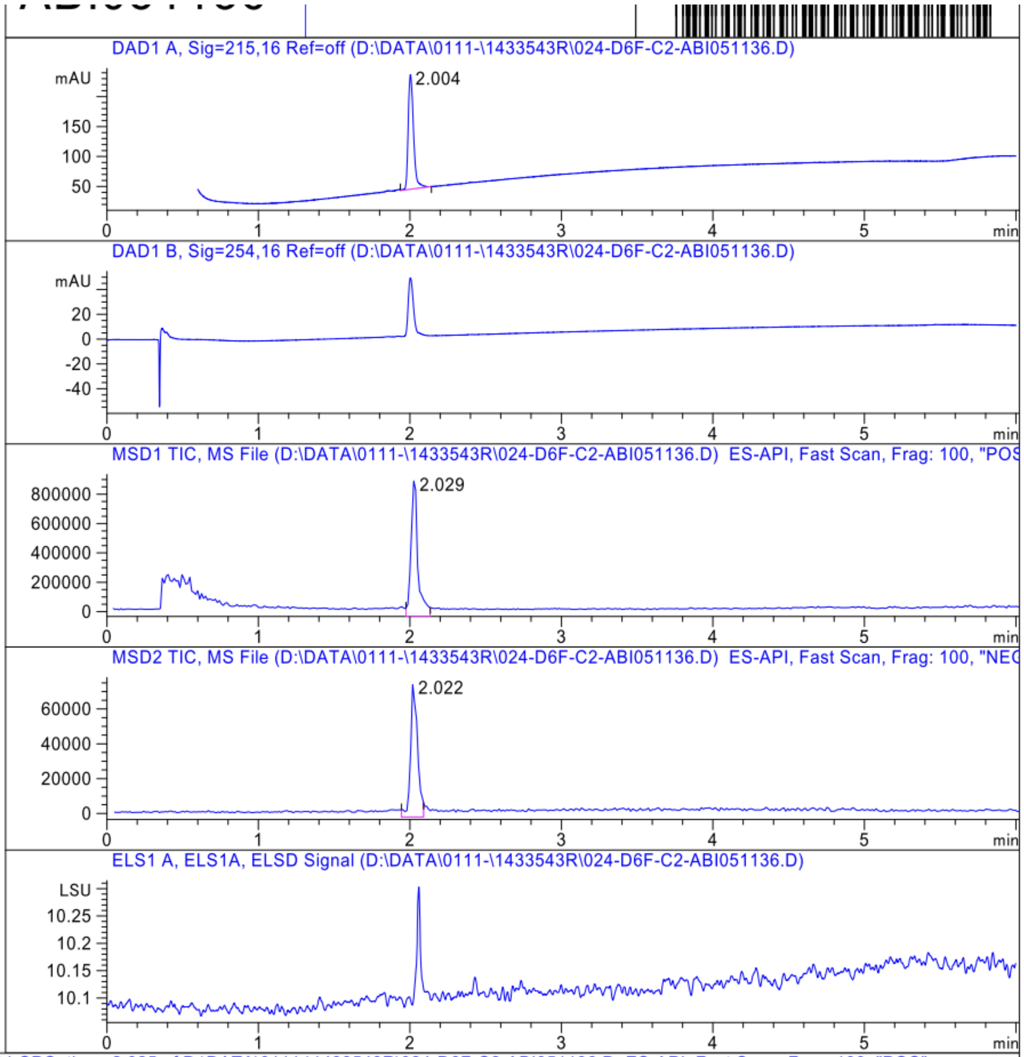

RT 2.029

RT 2.022

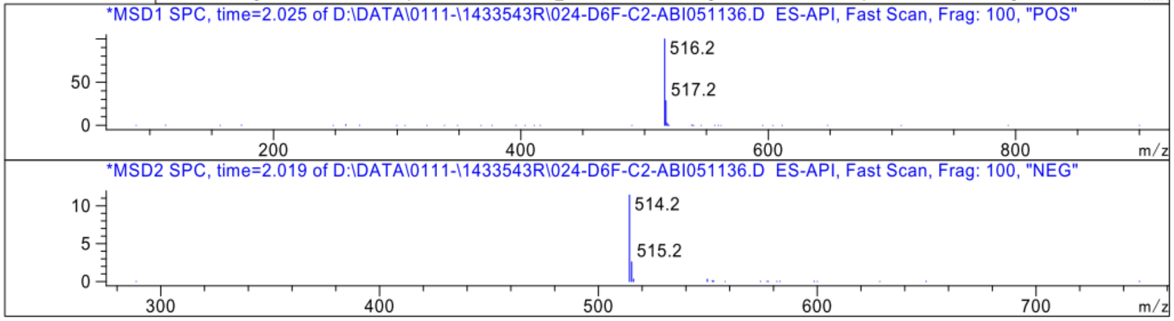

COMPOUND 39

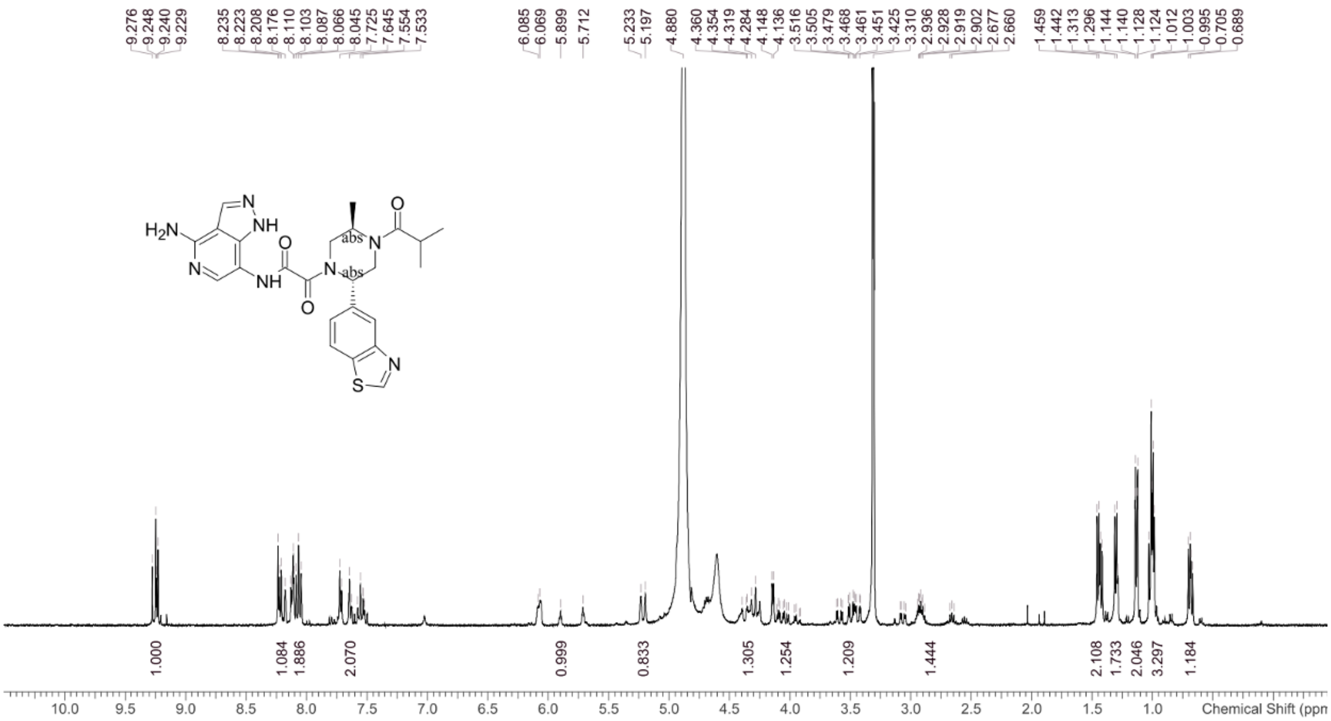

COMPOUND 40

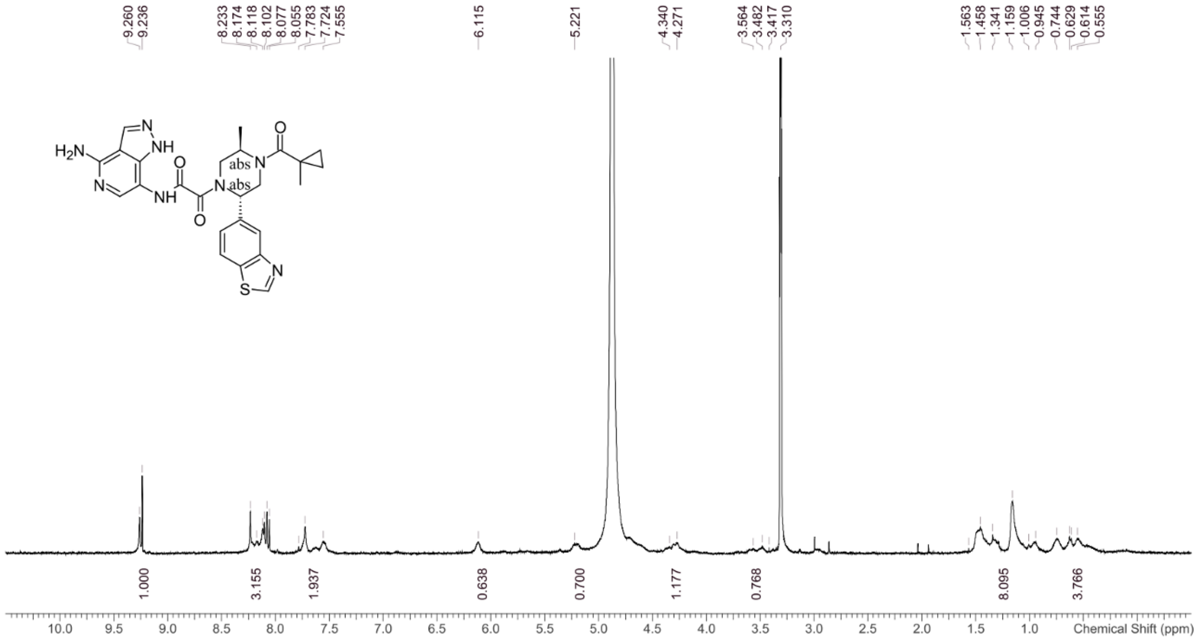

COMPOUND 41

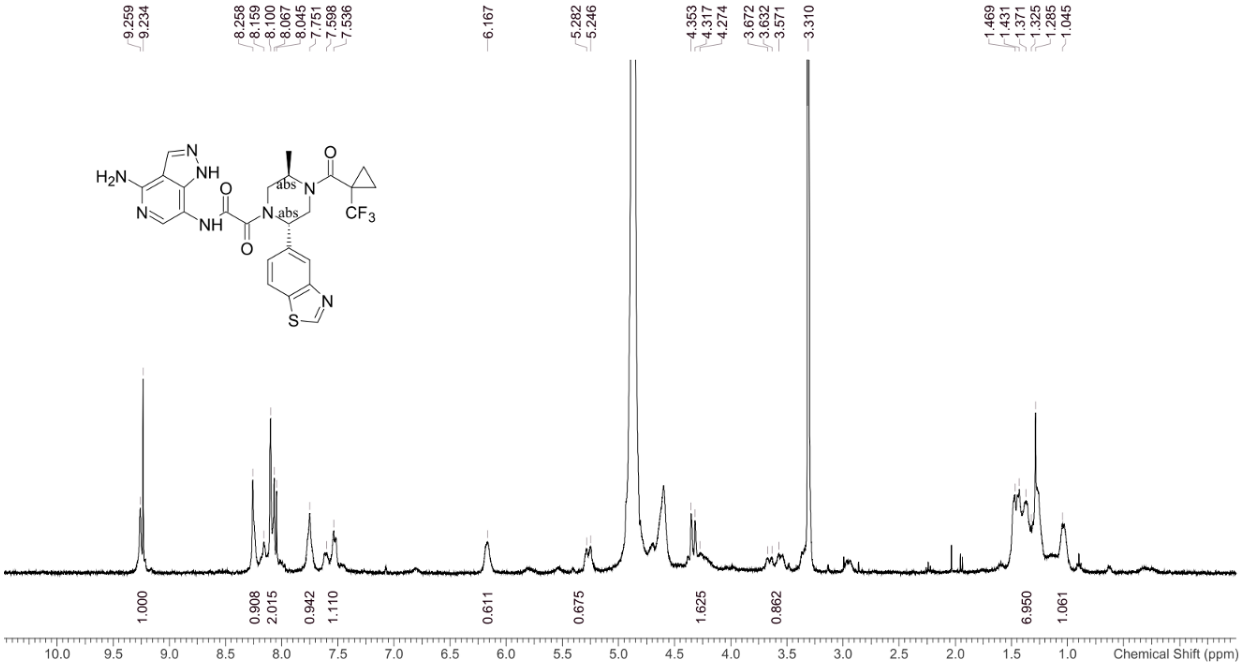
9.259
9.234
8.258
8.159
8.100
8.067
8.045
7.751
7.598
7.536
6.167
5.282
5.246
4.353
4.317
4.274
3.672
3.632
3.571
3.310
1.469
1.431
1.371
1.325
1.285
1.045
1.000
0.908
2.015
0.942
1.110
0.611
0.675
1.625
0.862
6.950
1.061
Chemical Shift (ppm)

COMPOUND 42

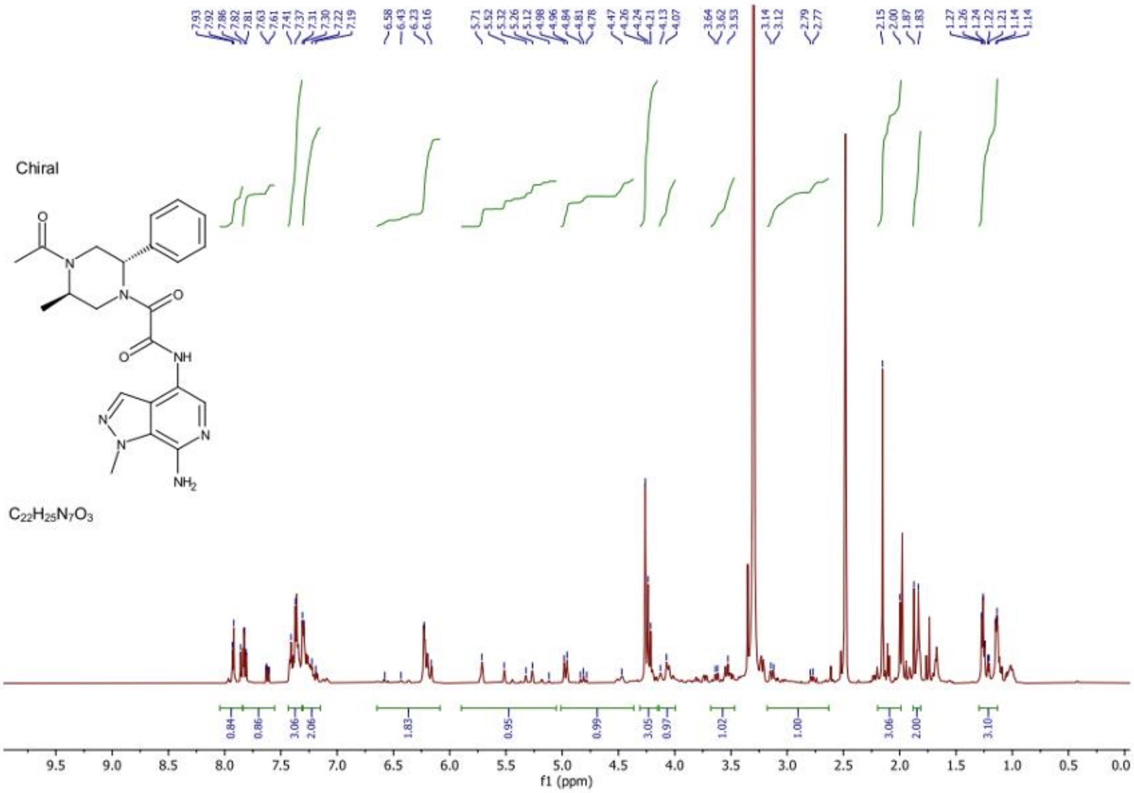

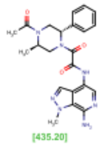

ABU080263

L560777
LCMS-19
6min_4-6x30_1-5_V.M
12:23 22.12.2022
MaxPeak: 90.9%

#	RT	DAD1A	DAD1B	MSD1	MSD2	ELSD	MSD1 ions	MSD1 rt	MSD2 ions	MSD2 rt	Info
1	1.523	1.0%	---	---	---	---	---	---	---	---	
2	1.597	90.9%	94.5%	84.3%	87.2%	---	436.2(98),514.2(2)	1.612	434.0(55),470.0(32),472.0(13)	1.613	P +H+,P NEG
3	1.795	6.5%	---	8.5%	---	---	261.2(100)	1.812	---	---	
4	2.032	1.6%	5.5%	7.2%	12.8%	---	396.2(100)	2.048	430.0(72),432.0(28)	2.051	

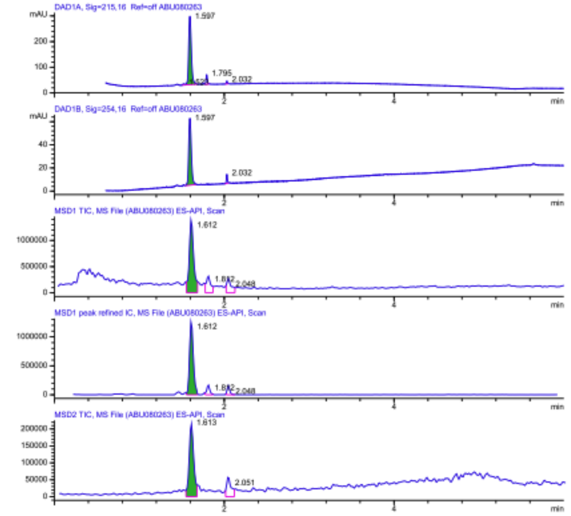

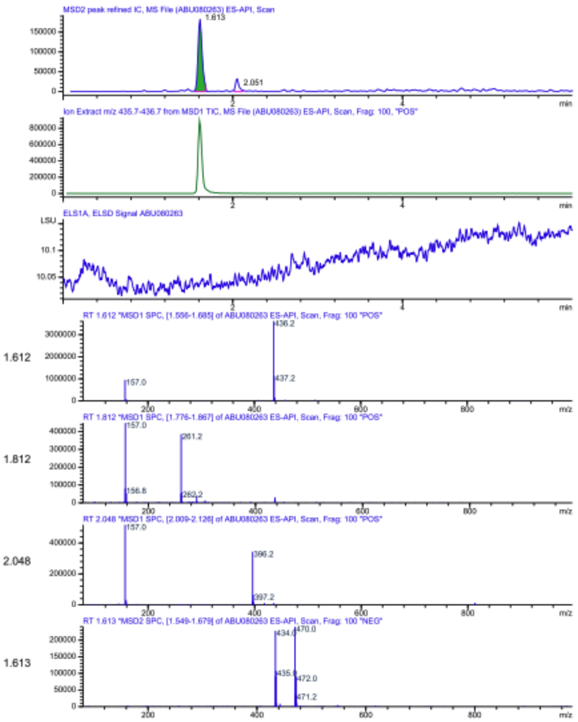

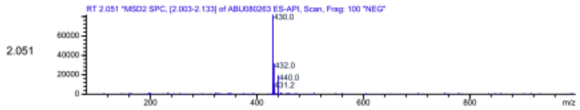

COMPOUND 43

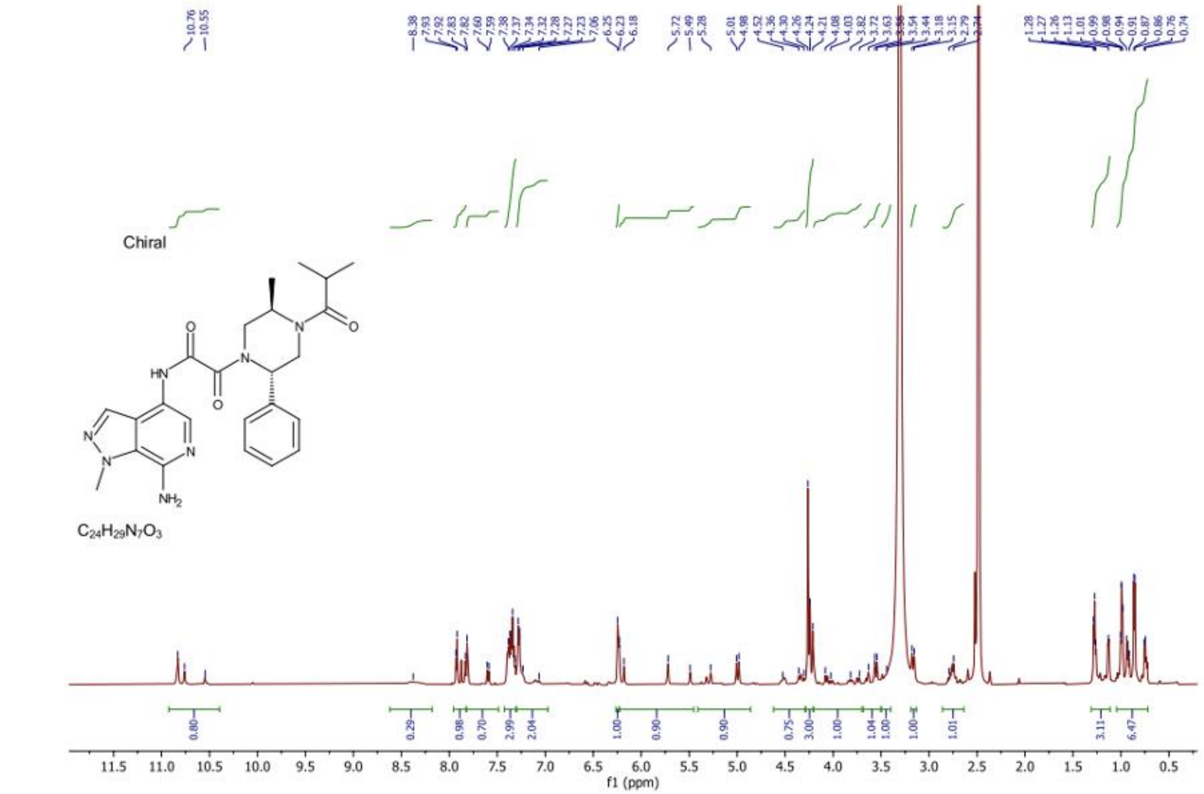

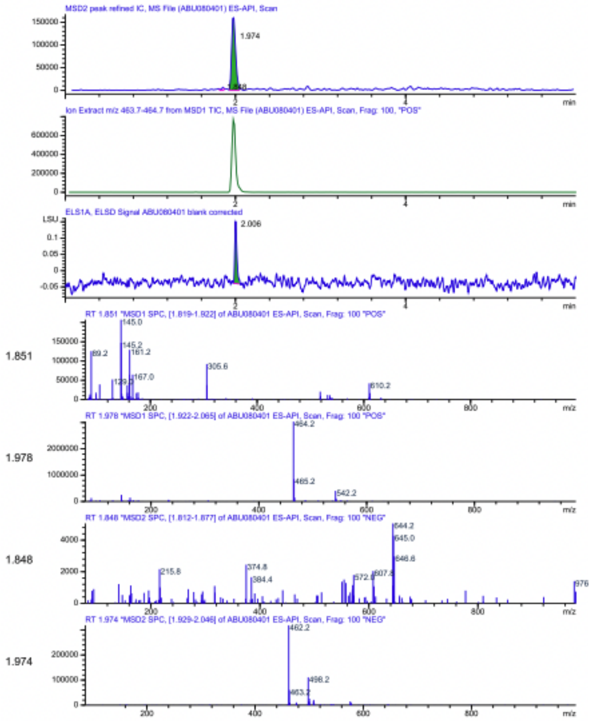

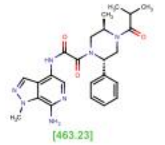

ABU080401

L558464R
LCMS-20
6min_4-6x30_1-5_V.M
11:20 15.12.2022
MaxPeak: 97.8%

#	RT	DAD1A	DAD1B	MSD1	MSD2	ELSD	MSD1 ions	MSD1 rt	MSD2 ions	MSD2 rt	Info
1	1.833	2.2%	2.3%	3.5%	0.9%	---	305.6(59),610.2(41)	1.851	644.2(100)	1.848	
2	1.959	97.8%	97.7%	96.5%	99.1%	100.0%	464.2(85),542.2(11),232.6(2)	1.978	462.2(58),498.2(26),508.2(7)	1.974	P +H+,P NEG

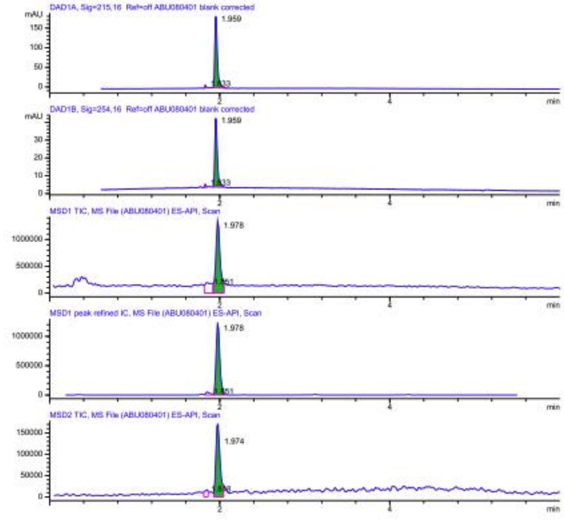

COMPOUND 44

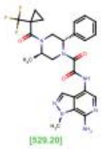

ABU080386

L557514R
LCMS-19
6min_4-6x30_1-5_V.M
11:40 13.12.2022
MaxPeak: 100.0%

#	RT	DAD1A	DAD1B	MSD1	MSD2	ELSD	MSD1 ions	MSD1 rt	MSD2 ions	MSD2 rt	Info
1	2.280	100.0%	100.0%	100.0%	100.0%	—	530.2(100)	2.297	528.0(85),564.0(15)	2.298	P +H+,P NEG

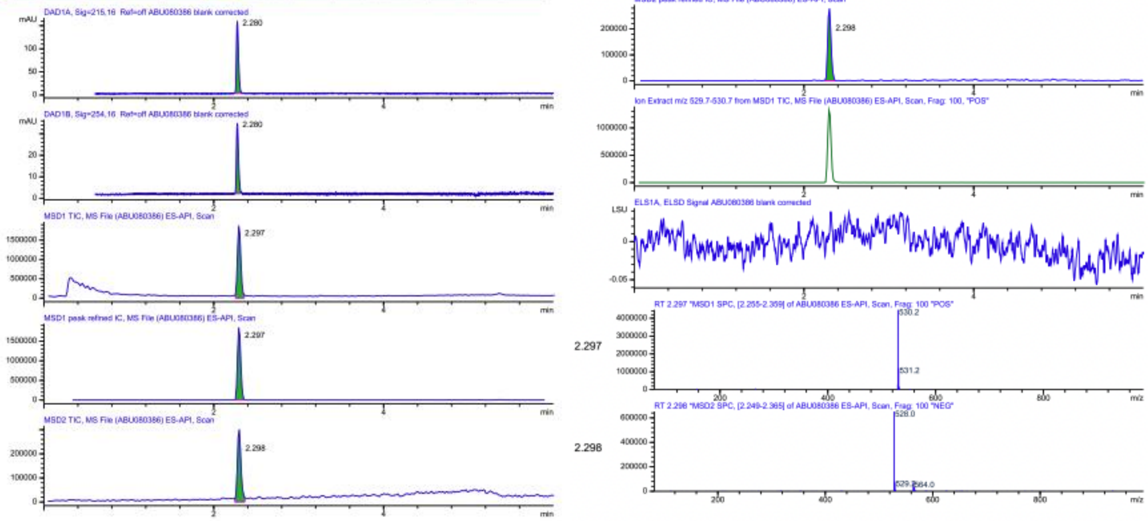

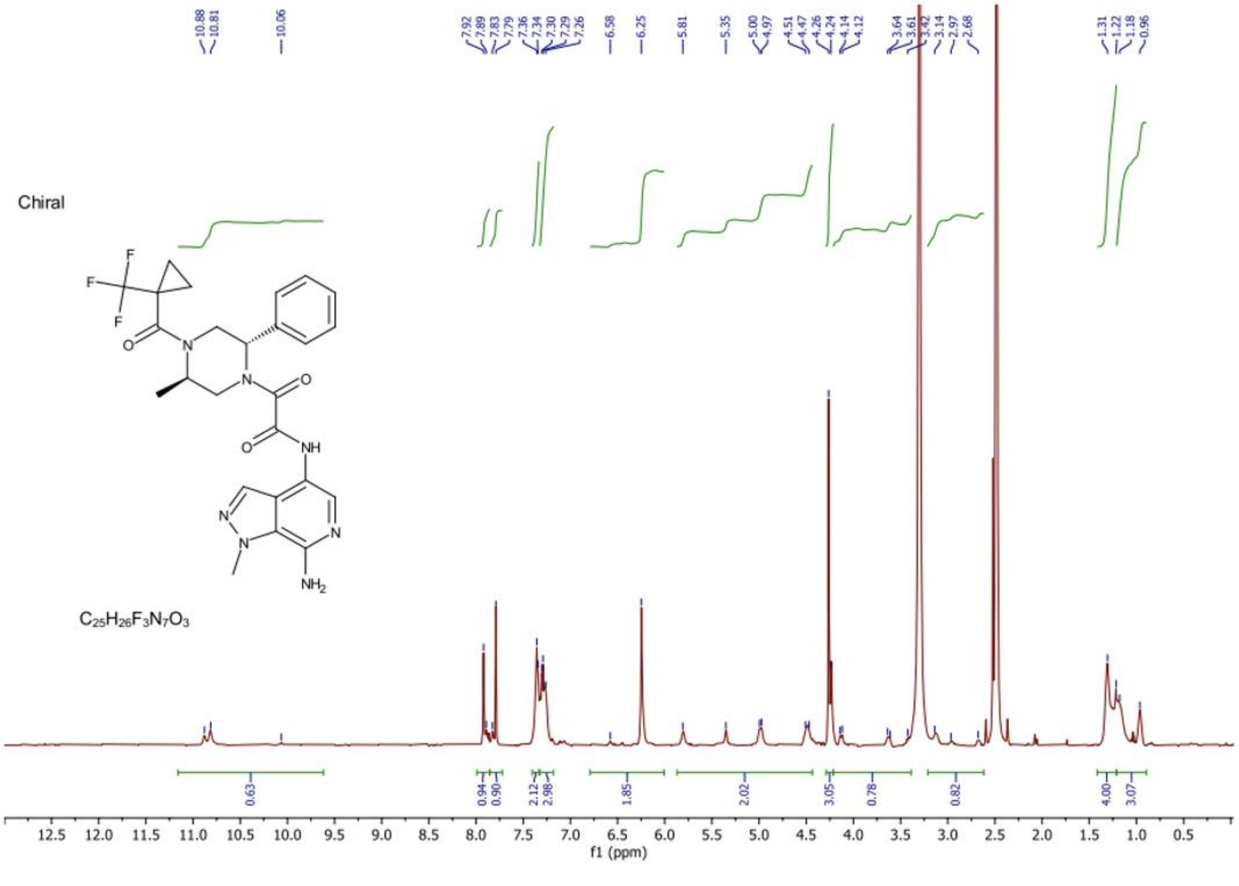

COMPOUND 45

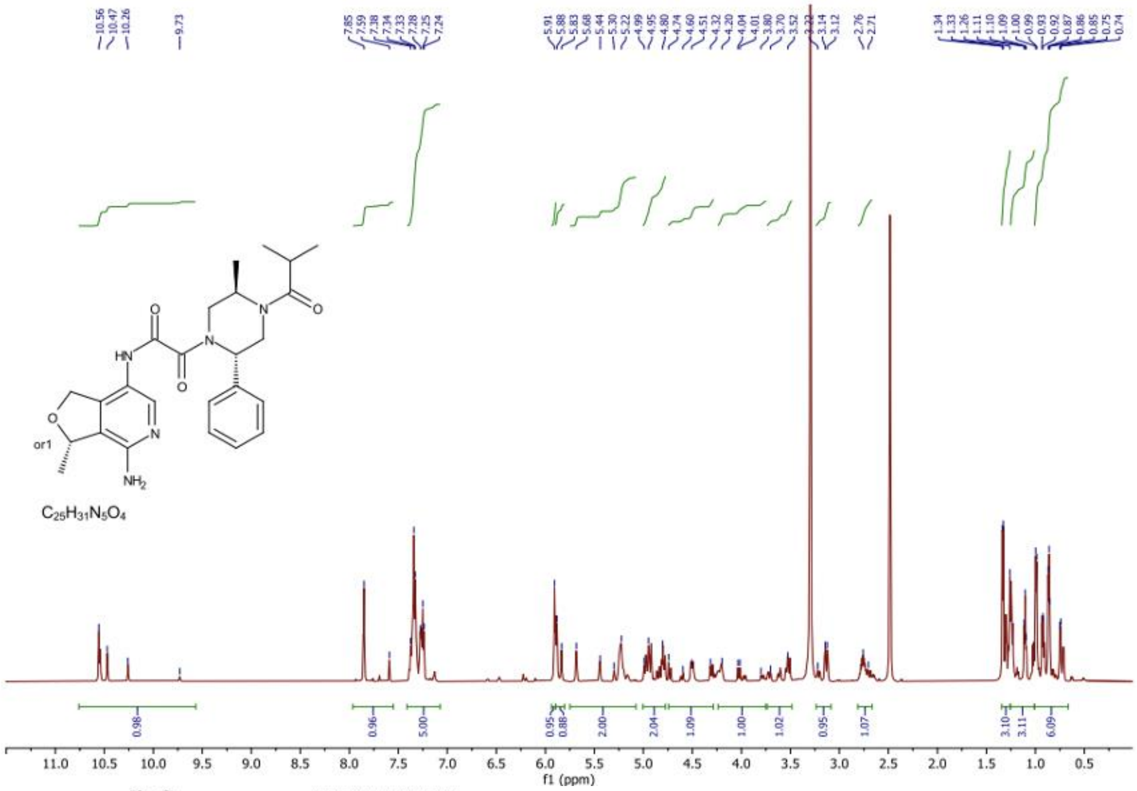

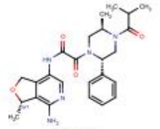

ABU078556

L564947D
LCMS-31
6min_4-6x30_1-5_V.M
11:48 05.01.2023
MaxPeak: 100.0%
Several product peaks

#	RT	DAD1A	DAD1B	MSD1	MSD2	ELSD	MSD1 ions	MSD1 rt	MSD2 ions	MSD2 rt	Info
1	2.133	—	—	42.0%	8.0%	—	466.2(100)	2.149	465.2(100)	2.147	P +H+
2	2.143	100.0%	100.0%	24.9%	92.0%	100.0%	467.2(100)	2.158	464.2(100)	2.156	P NEG
3	2.153	—	—	33.2%	—	—	466.2(96),468.2(4)	2.169	—	—	P +H+

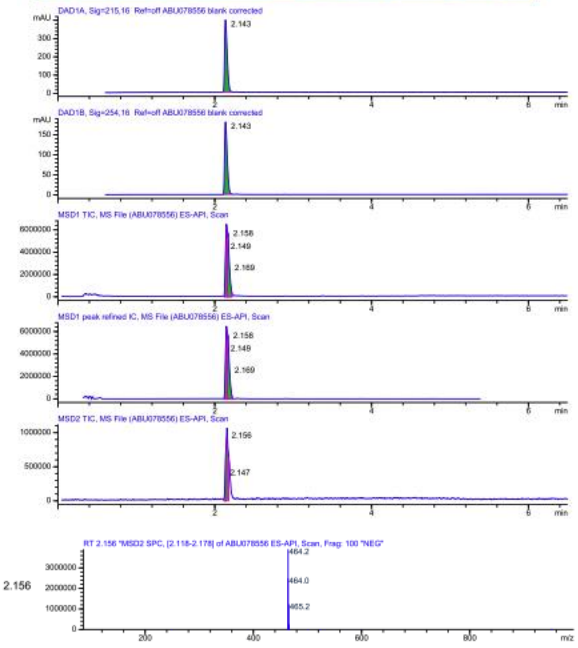

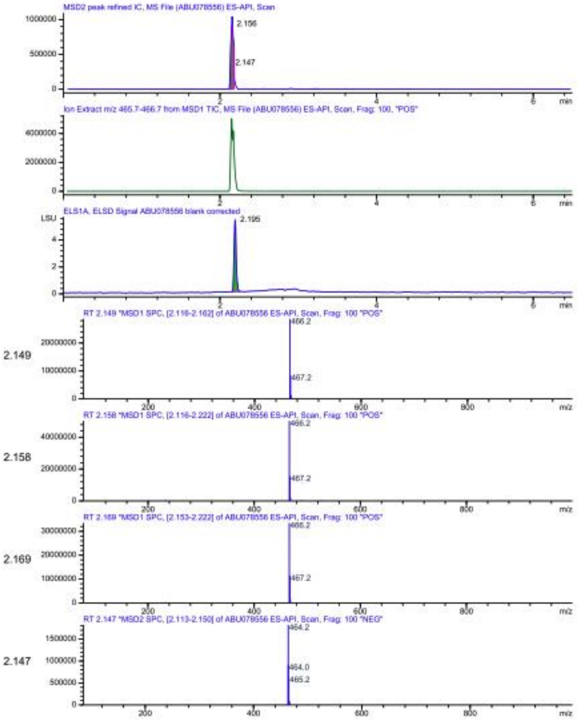

COMPOUND 46

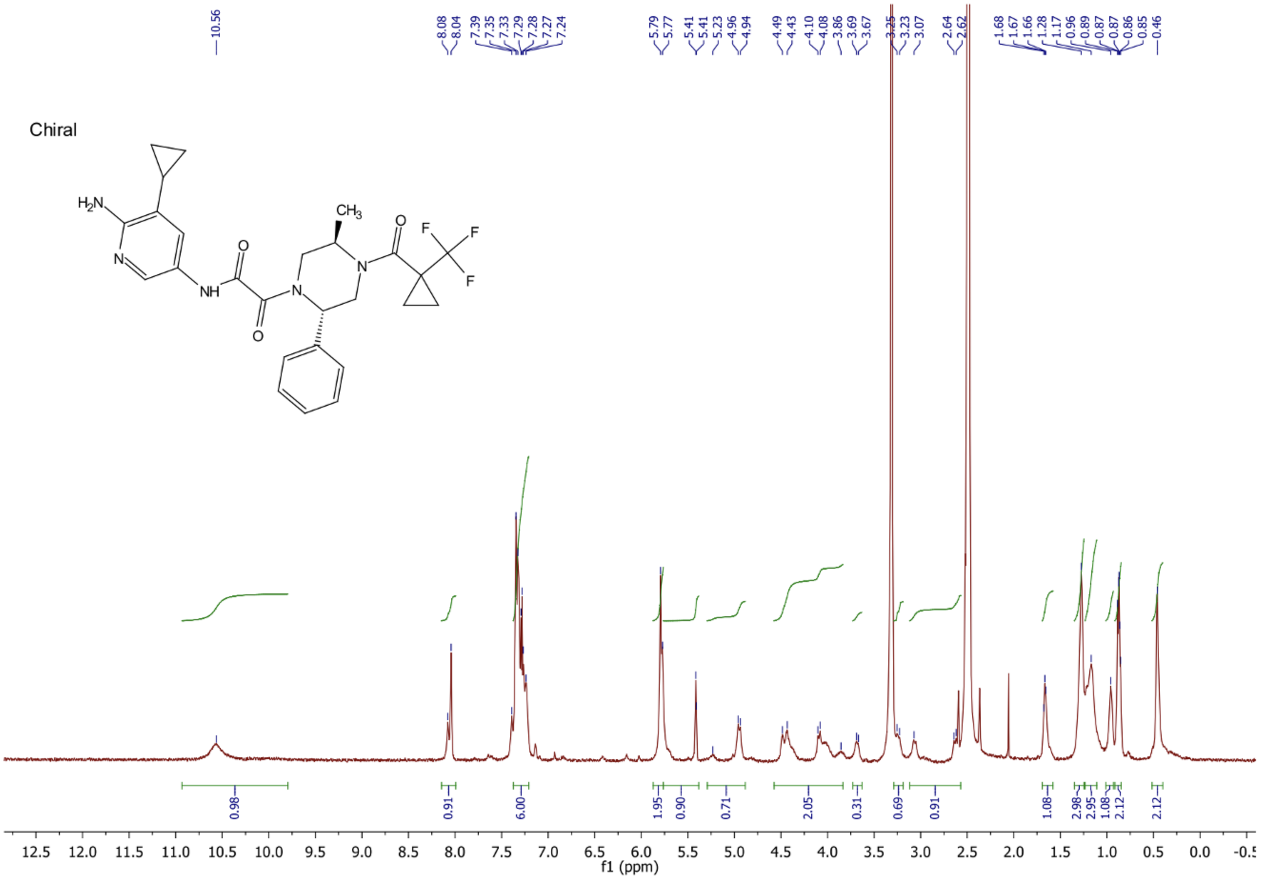
Chiral
f1 (ppm)

MaxPeak: 100.00%
Ret_Time: 2.843 min

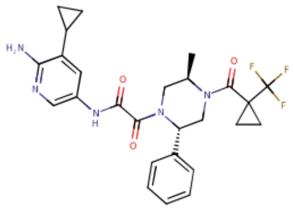

Mol Wt 515.53
Exact Mass 515.25

#	Time	Area%
1	2.843	100.00

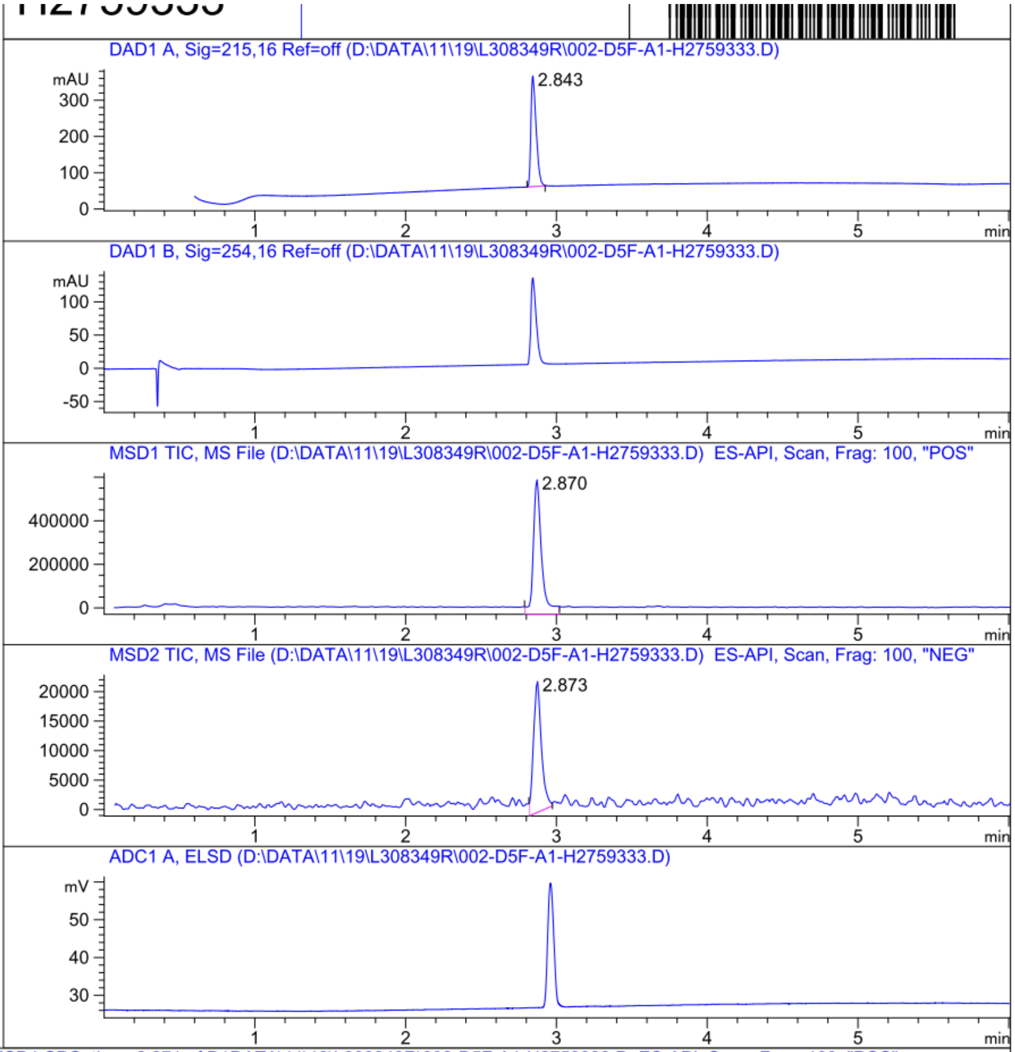

RT 2.870

RT 2.873

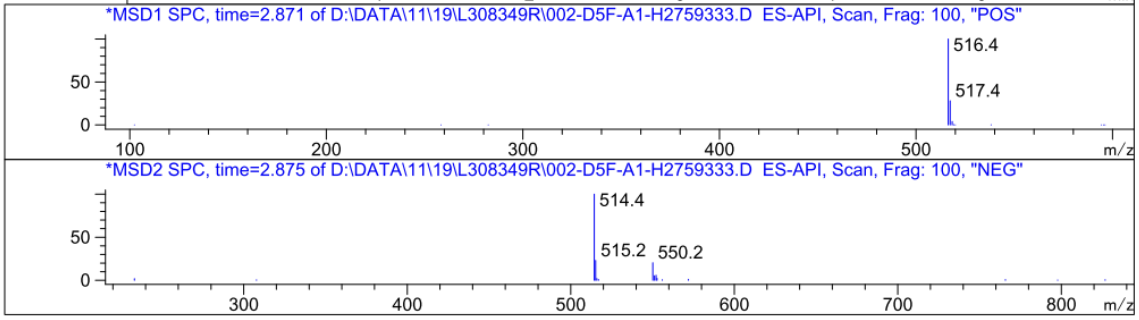

COMPOUND 47

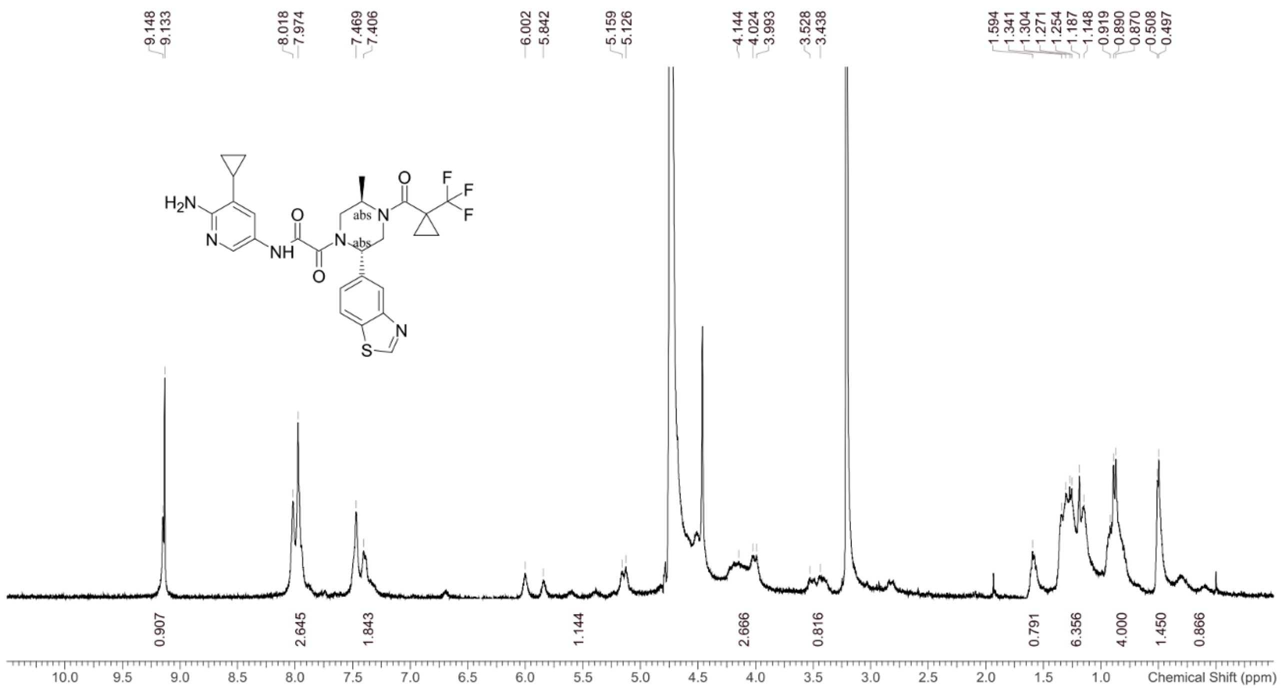

COMPOUND 48

MaxPeak: 96.38%
Ret_Time: 2.719 min

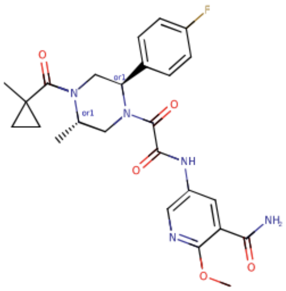

Mol Wt 497.52

Exact Mass 497.23

#	Time	Area%
1	2.719	96.38
2	2.776	3.62

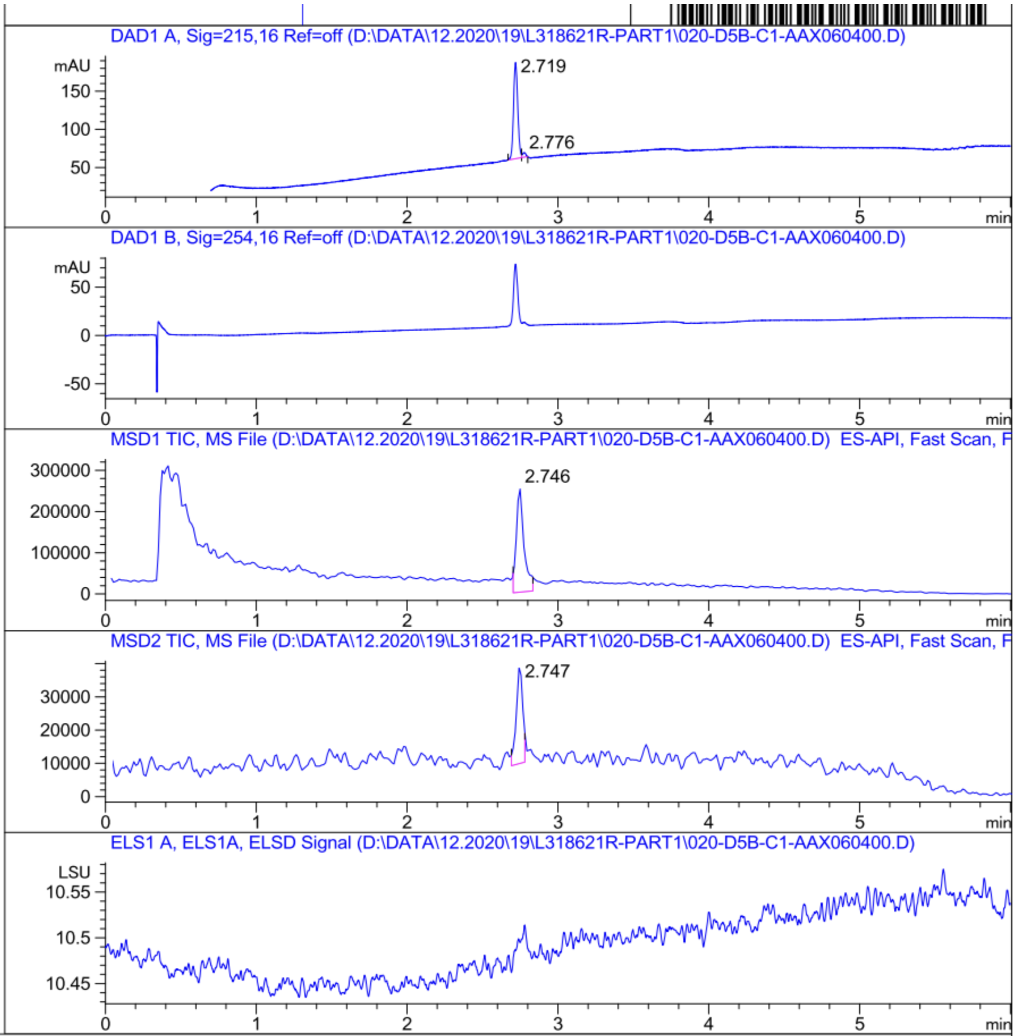

RT 2.746

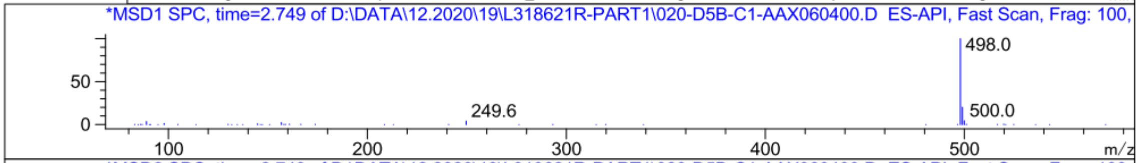

RT 2.747

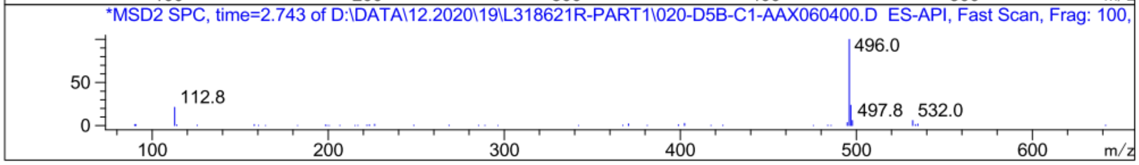

COMPOUND 49

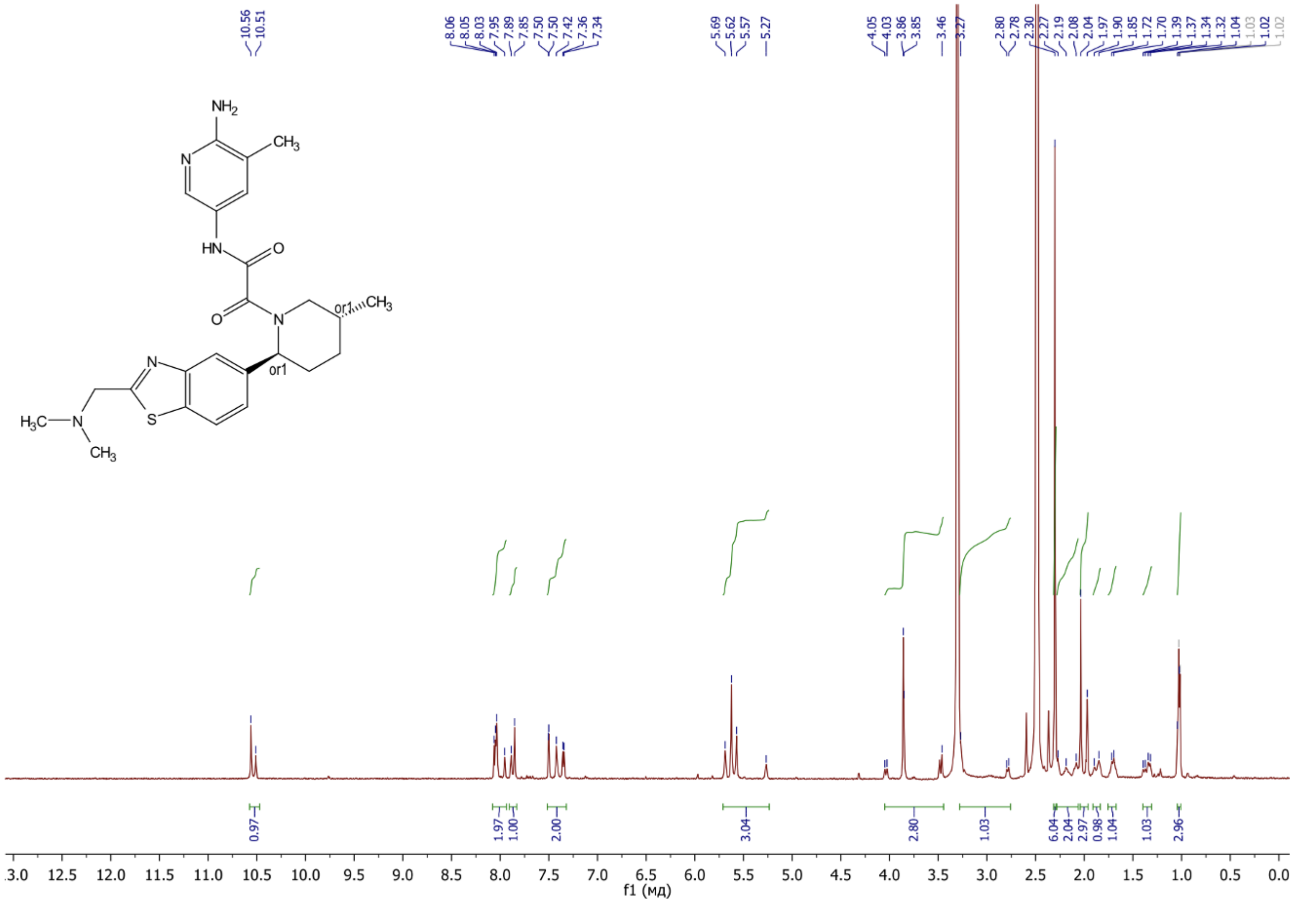
NH2
CH3
N
HN
O
or1
CH3
S
H3C
f1 (мд)

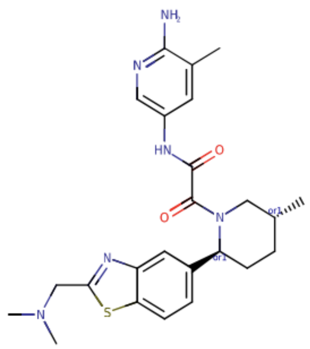

Mol Wt 466.6

Exact Mass 466.25

#	Time	Area%
1	1.440	97.96
2	2.208	2.04

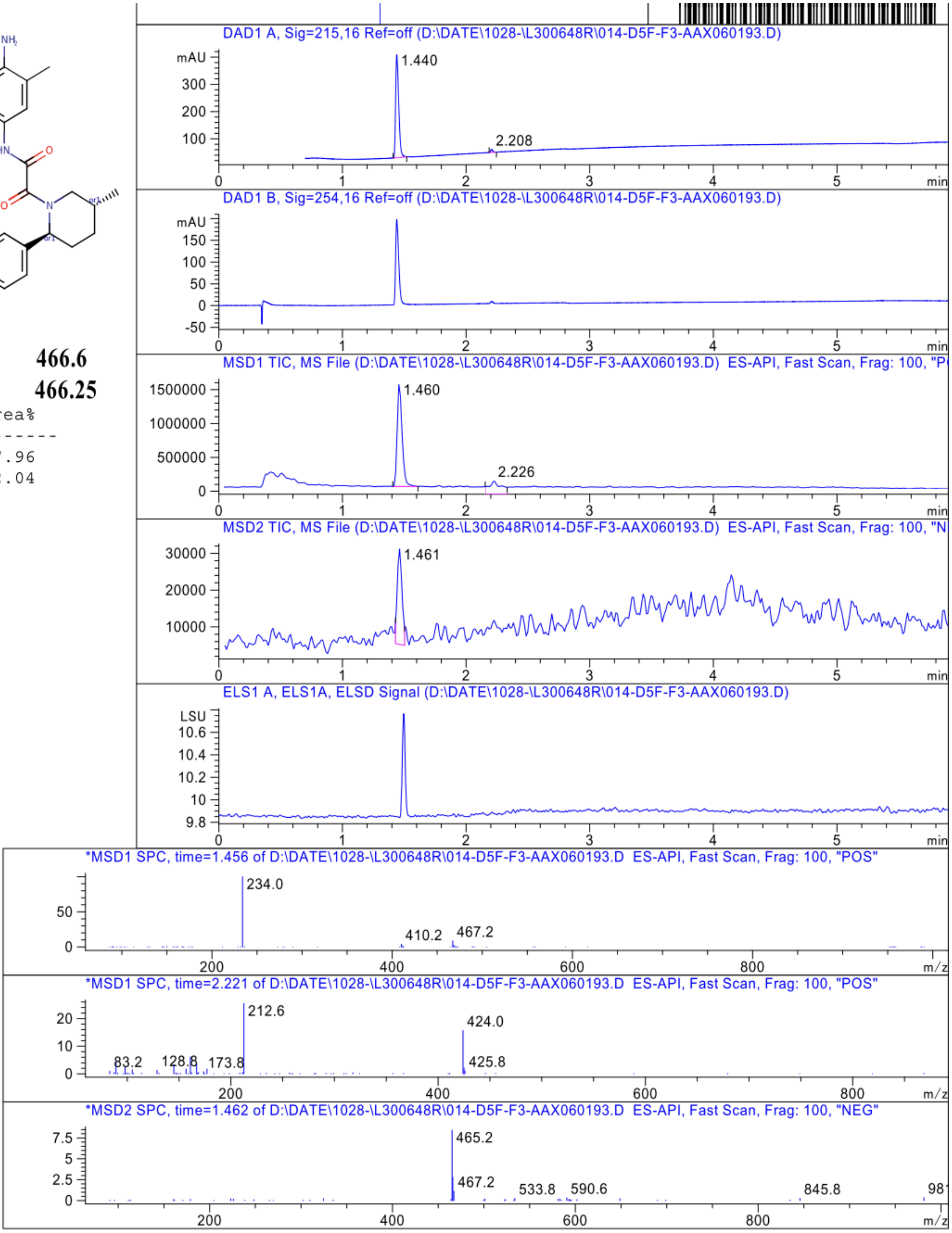

RT 1.460

RT 2.226

RT 1.461

COMPOUND 50

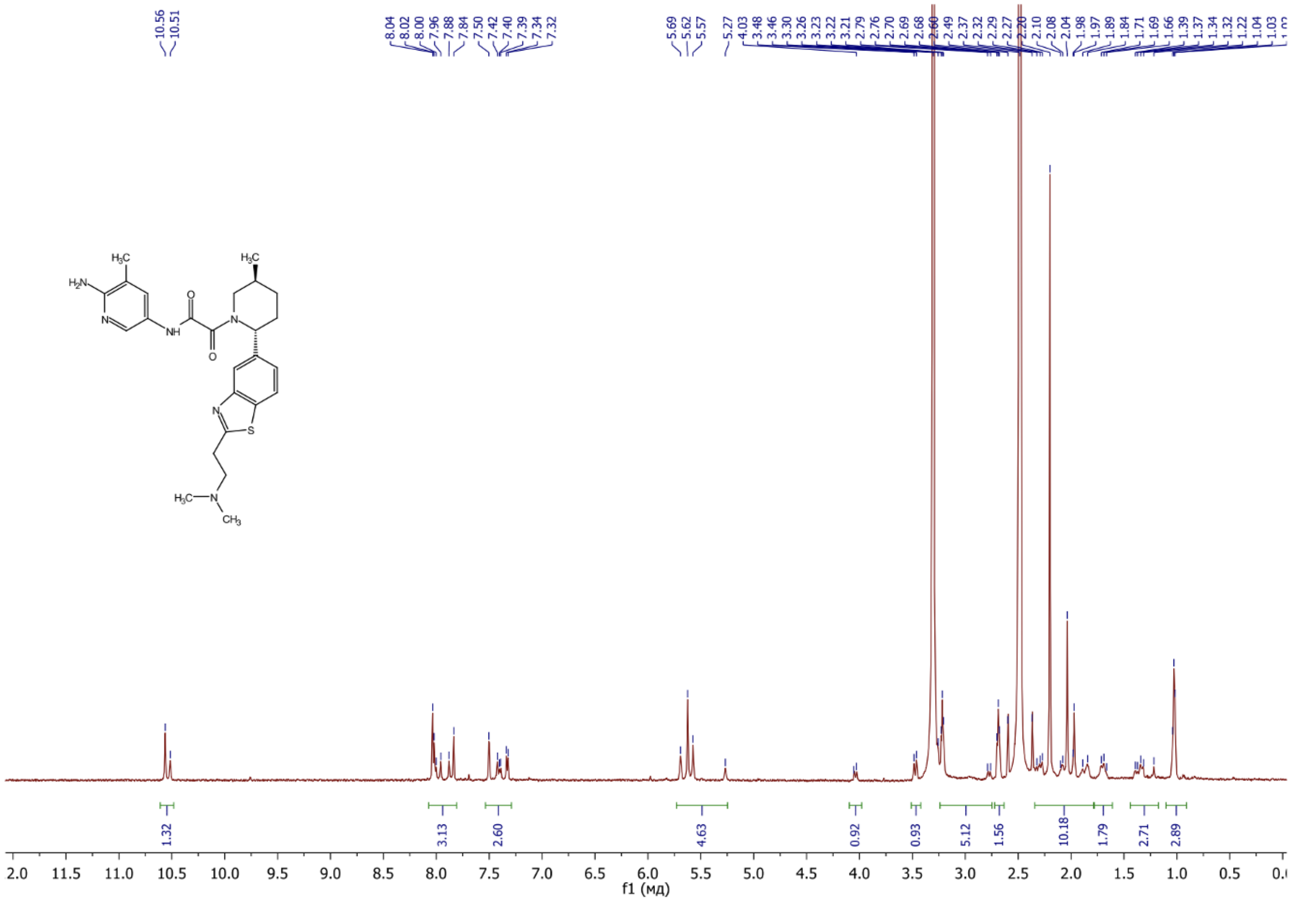

MaxPeak: 98.49%
Ret_Time: 1.800 min

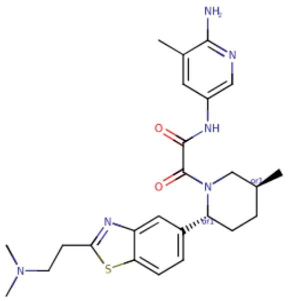

Mol Wt 480.63
Exact Mass 480.27

#	Time	Area%
1	1.800	98.49
2	2.654	1.51

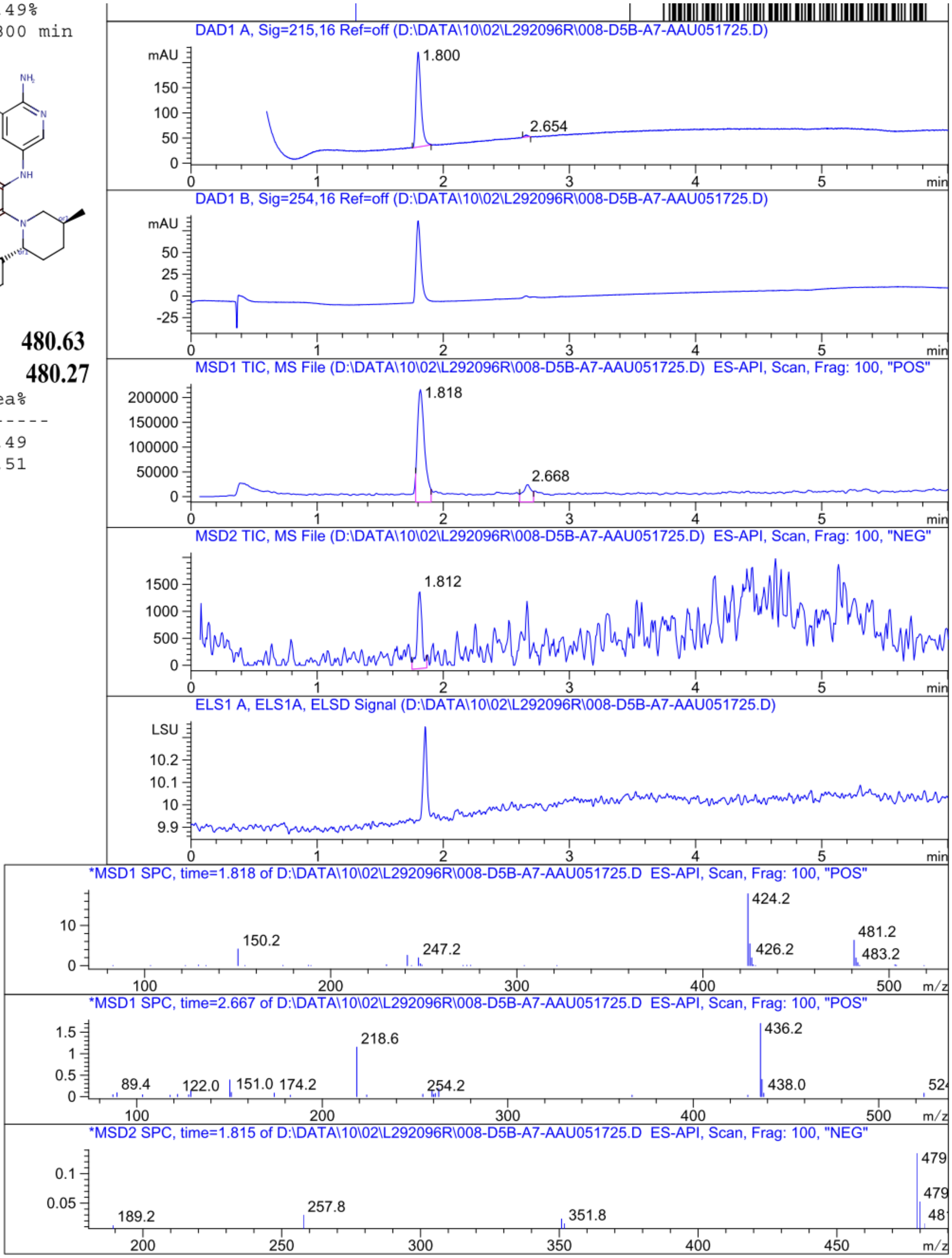

COMPOUND 51

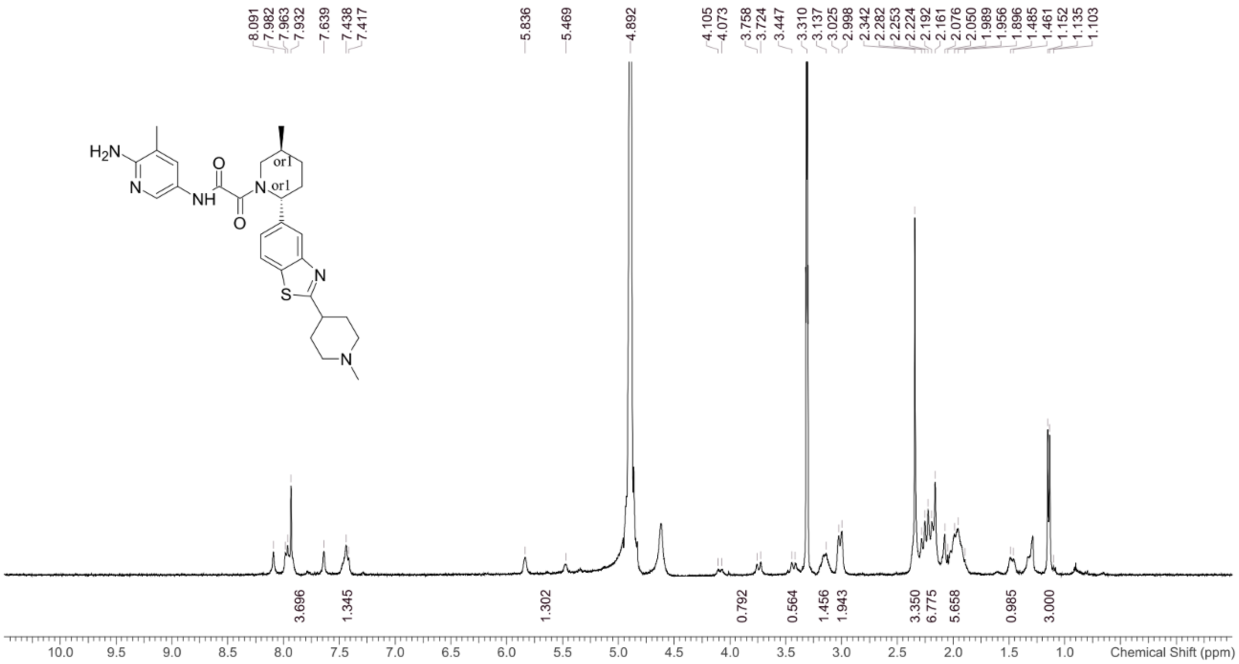

COMPOUND 52

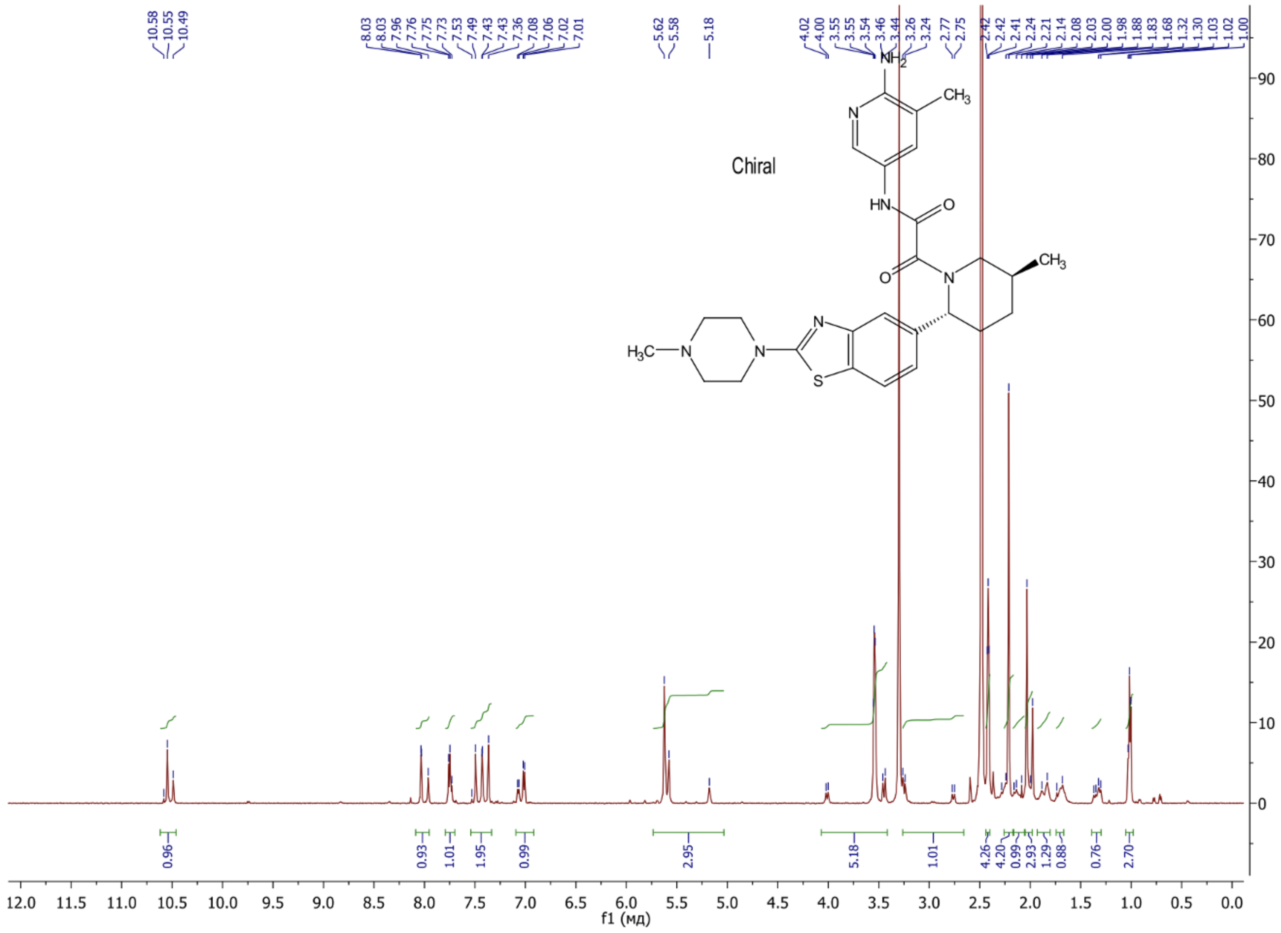
Chiral
f1 (мд)

MaxPeak: 91.29%
Ret_Time: 1.961 min

H2784380

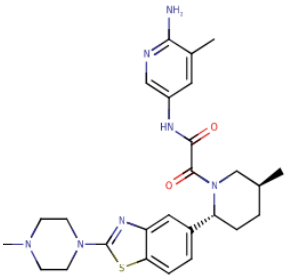

Mol Wt		507.65
Exact Mass		507.28

#	Time	Area%
1	1.961	91.29
2	2.029	8.71

RT 1.998

RT 1.993

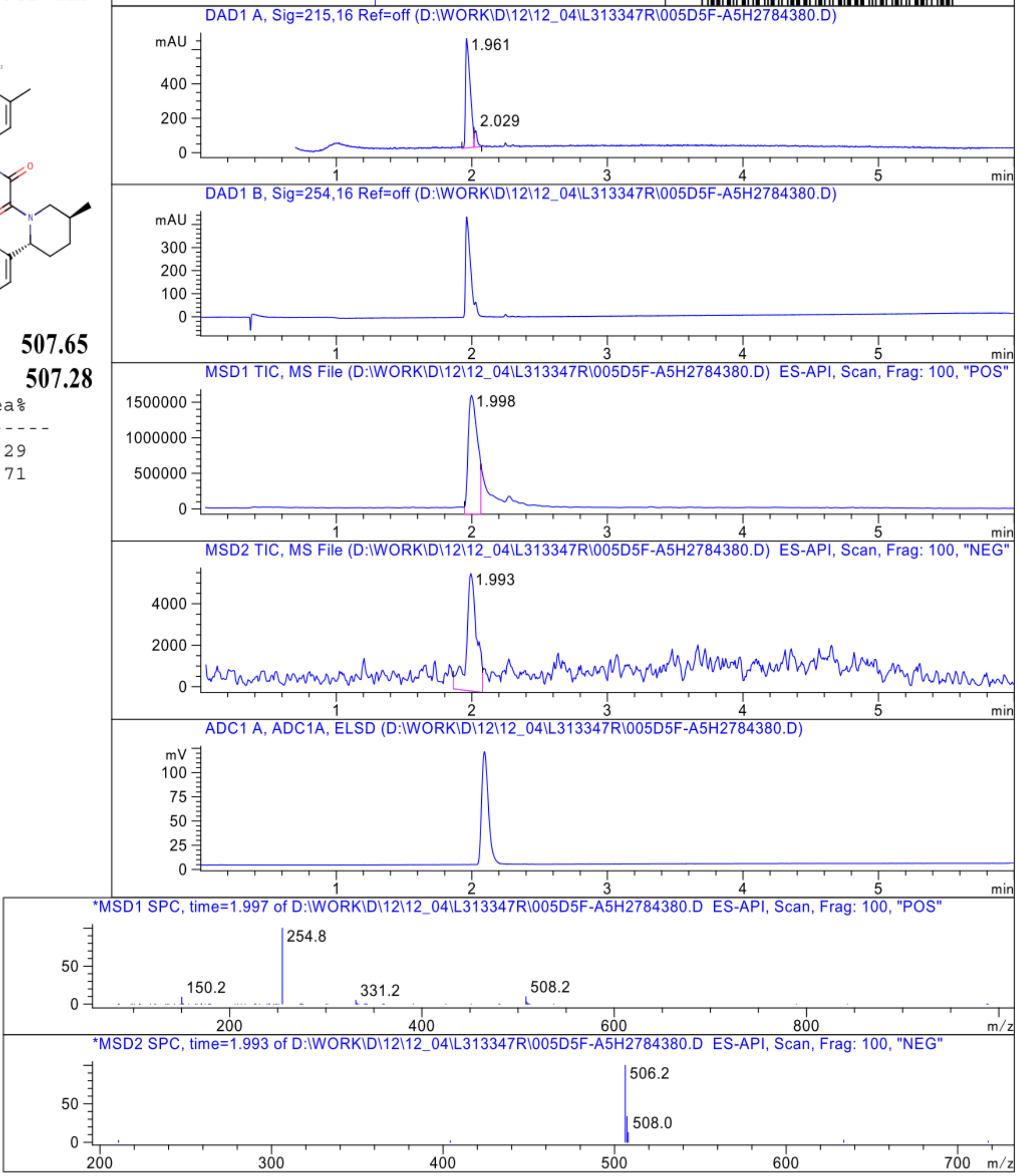

COMPOUND 53

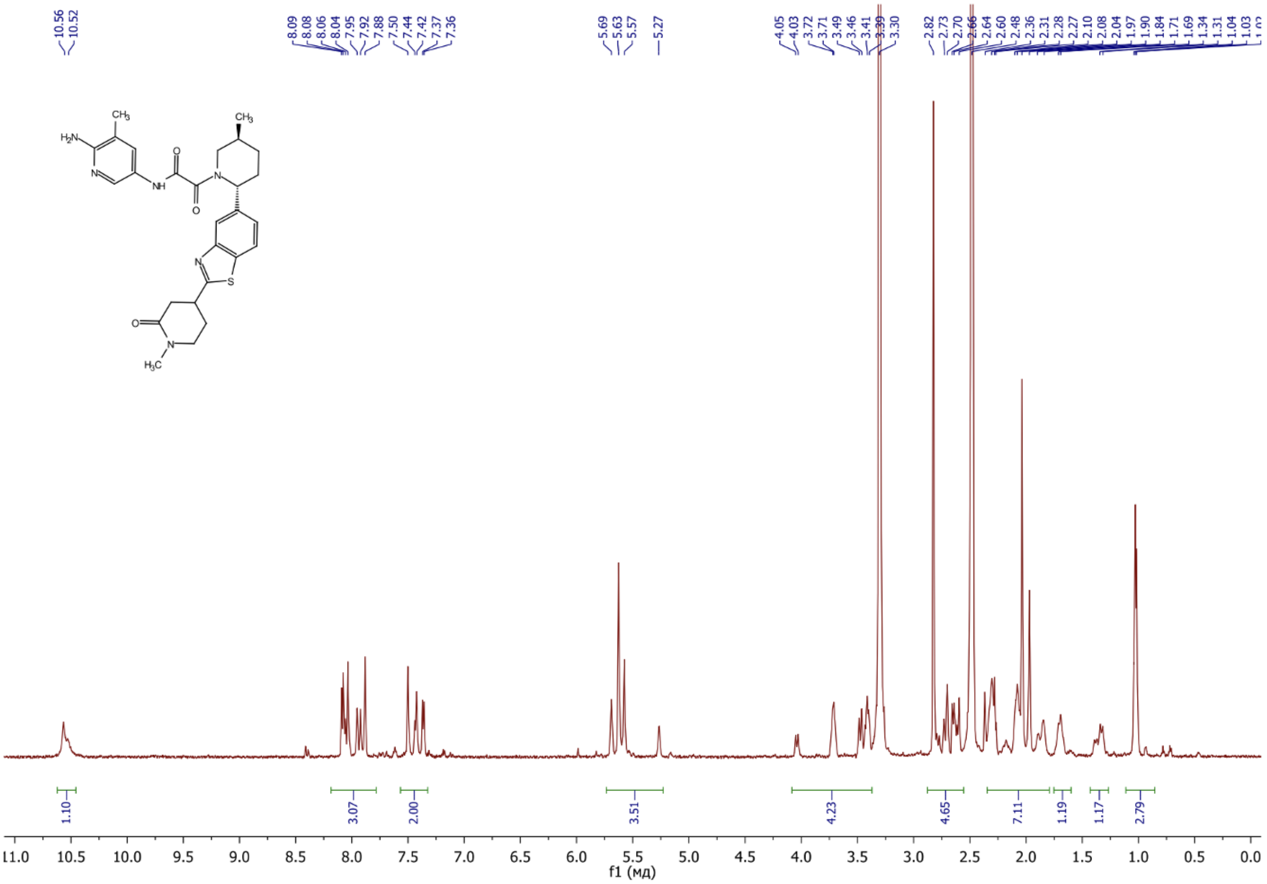
10.56
10.52
8.09
8.08
8.06
8.04
7.95
7.92
7.88
7.50
7.44
7.42
7.37
7.36
5.69
5.63
5.57
5.27
4.05
4.03
3.72
3.71
3.49
3.46
3.41
3.39
3.30
2.82
2.73
2.70
2.66
2.64
2.60
2.48
2.36
2.31
2.28
2.27
2.10
2.08
2.04
1.97
1.90
1.84
1.71
1.69
1.34
1.31
1.04
1.03
CH3
H2N
N
NH
O
O
N
CH3
N
S
O
N
H3C
1.10
3.07
2.00
3.51
4.23
4.65
7.11
1.19
1.17
2.79
11.0
10.5
10.0
9.5
9.0
8.5
8.0
7.5
7.0
6.5
6.0
5.5
5.0
4.5
4.0
3.5
3.0
2.5
2.0
1.5
1.0
0.5
0.0
f1 (мд)

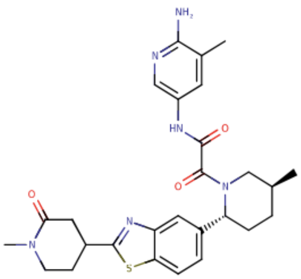

Mol Wt 520.65

Exact Mass 520.26

#	Time	Area%
1	1.873	2.78
2	2.331	94.09
3	2.405	3.14

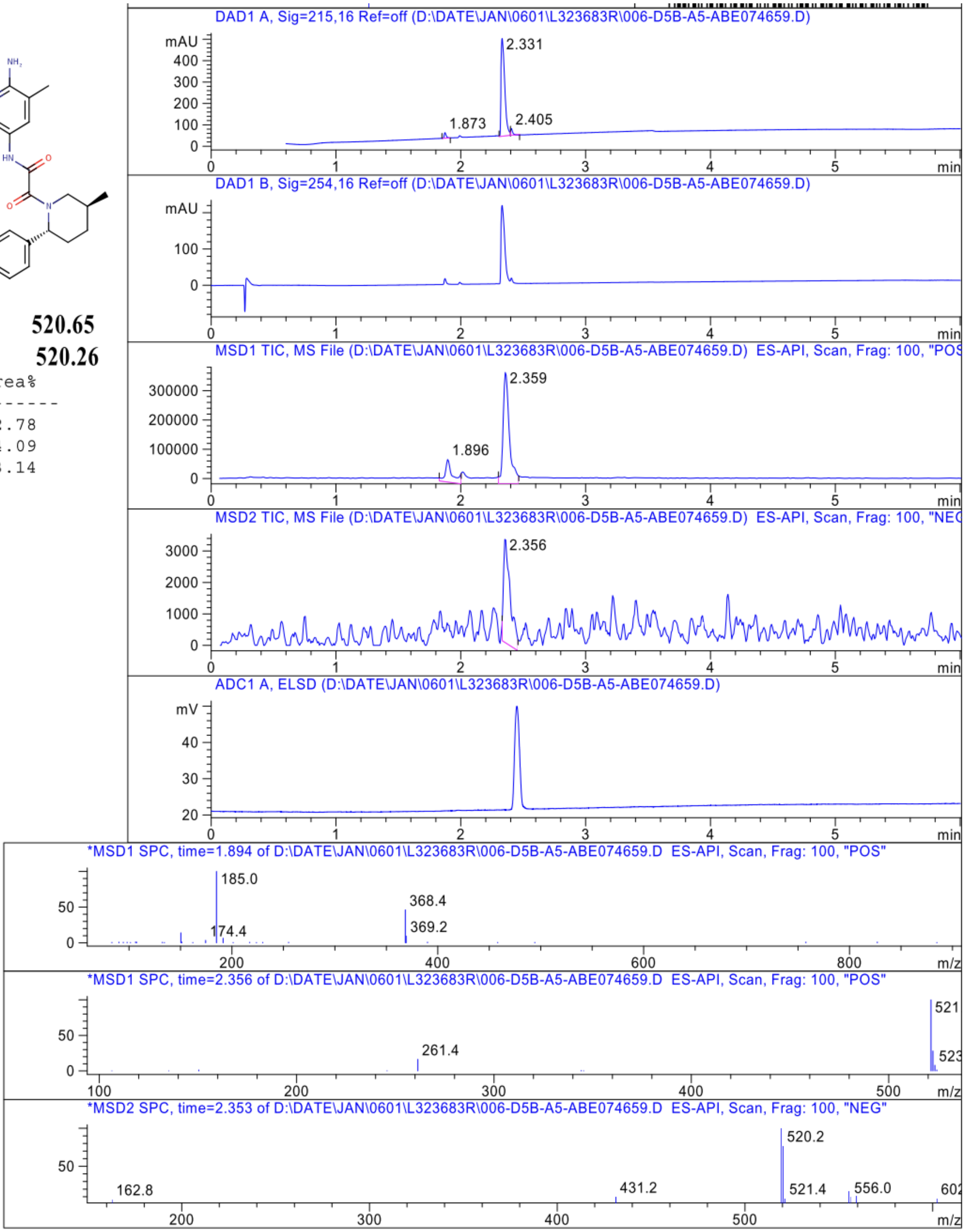

RT 1.896

RT 2.359

RT 2.356

COMPOUND 54

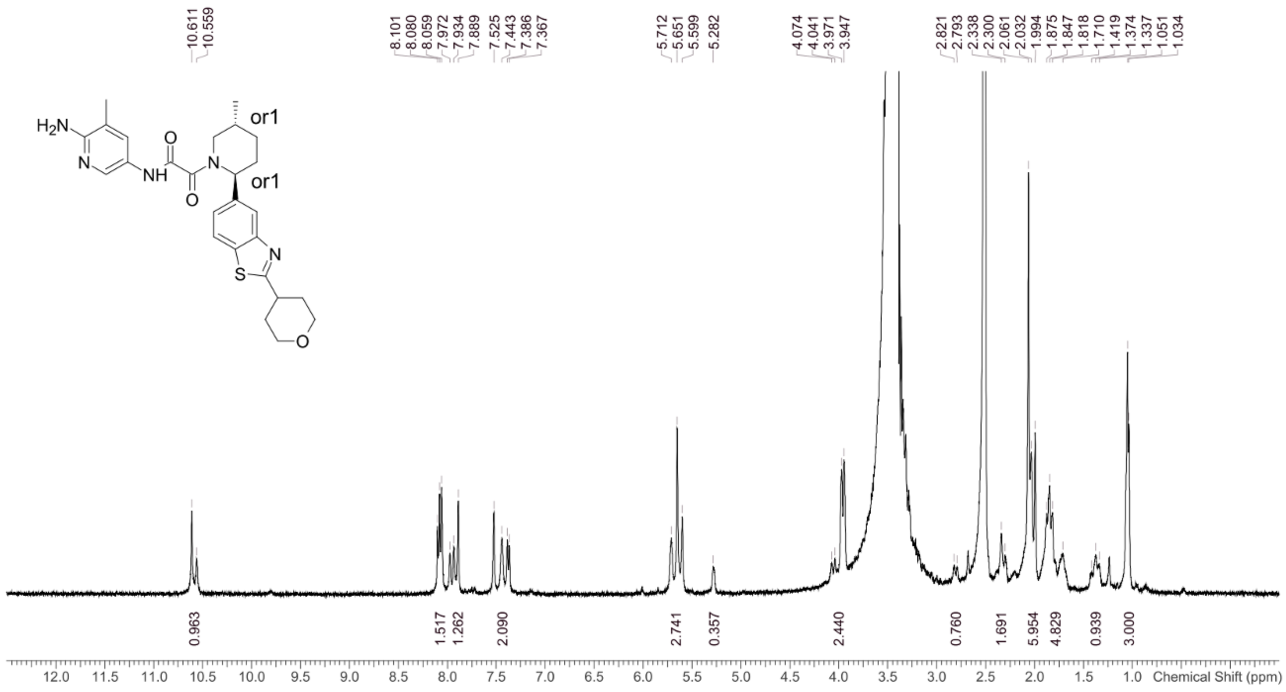

COMPOUND 55

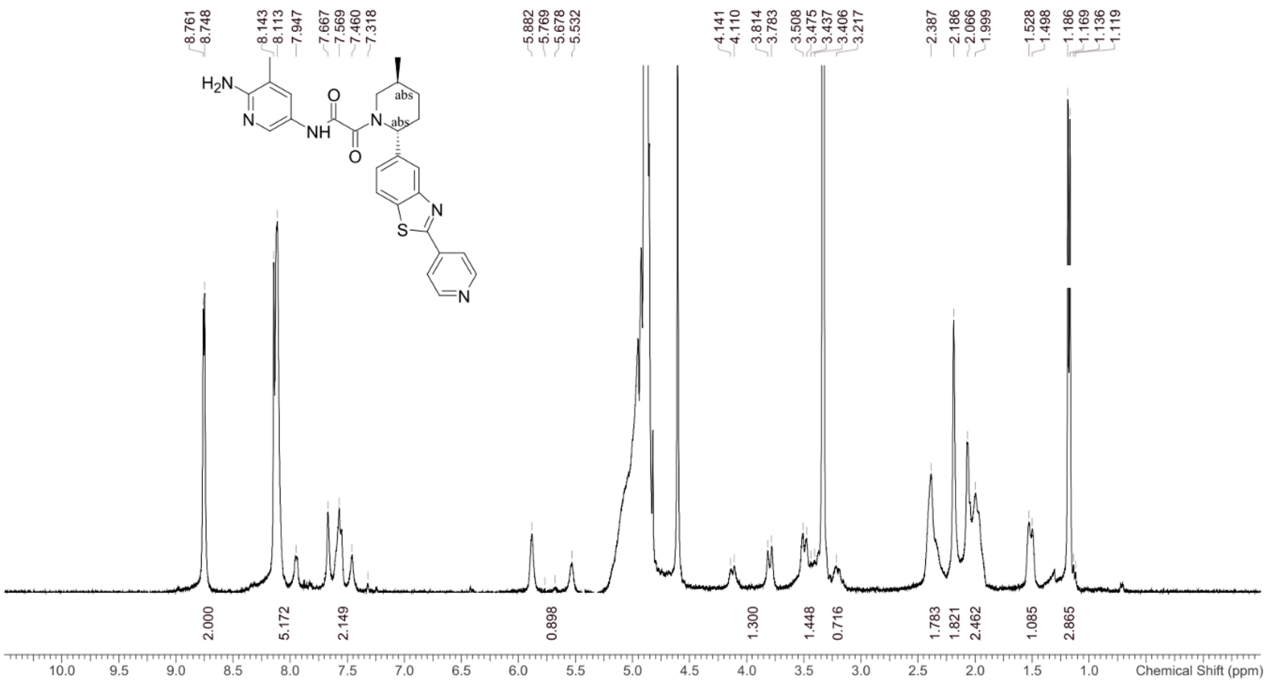

COMPOUND 56

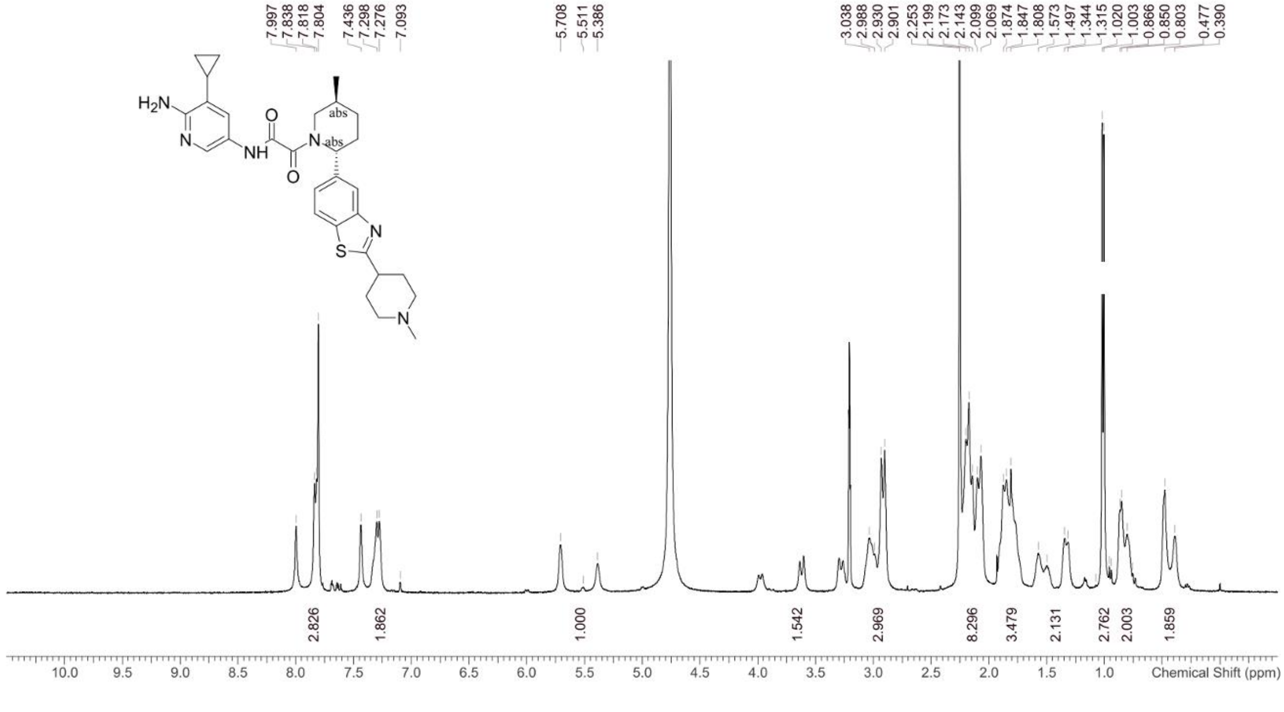
7.997
7.838
7.818
7.804
7.436
7.298
7.276
7.093
5.708
5.511
5.386
3.038
2.988
2.930
2.901
2.253
2.199
2.173
2.143
2.099
2.069
1.874
1.847
1.808
1.573
1.497
1.344
1.315
1.020
1.003
0.866
0.850
0.803
0.477
0.390
H2N
N
NH
O
O
N
abs
abs
N
S
N
2.826
1.862
1.000
1.542
2.969
8.296
3.479
2.131
2.762
2.003
1.859
10.0
9.5
9.0
8.5
8.0
7.5
7.0
6.5
6.0
5.5
5.0
4.5
4.0
3.5
3.0
2.5
2.0
1.5
1.0
Chemical Shift (ppm)

COMPOUND 57

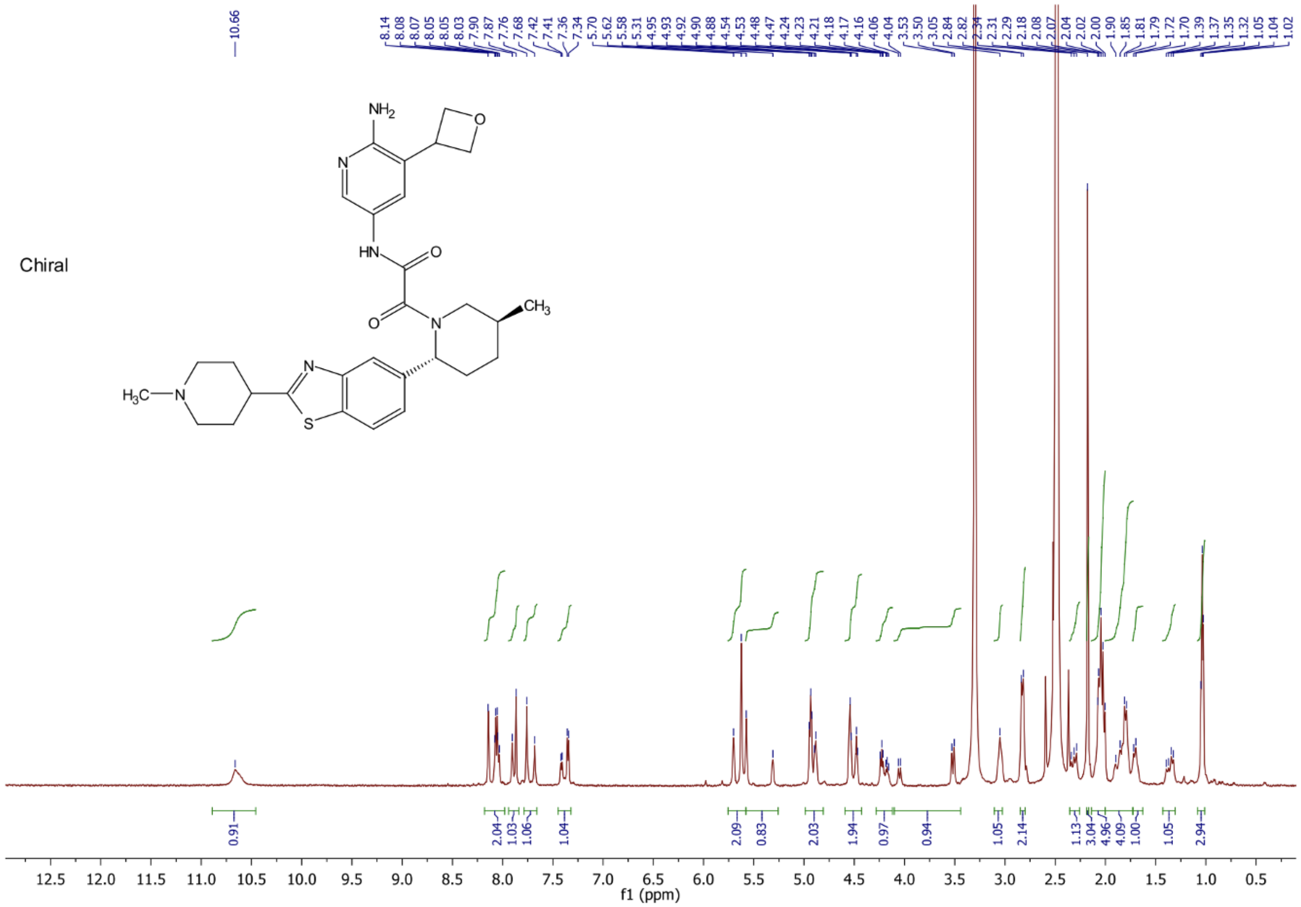
Chiral
NH2
O
N
HN
O
O
N
CH3
N
H3C—N
S
f1 (ppm)

MaxPeak: 97.55%
Ret_Time: 0.857 min

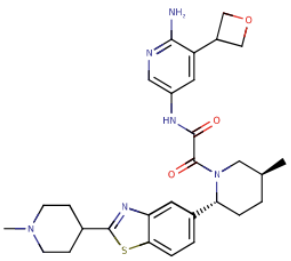

Mol Wt 548.7
Exact Mass 548.3

#	Time	Area%
1	0.841	2.45
2	0.857	97.55

RT 0.877

RT 0.882

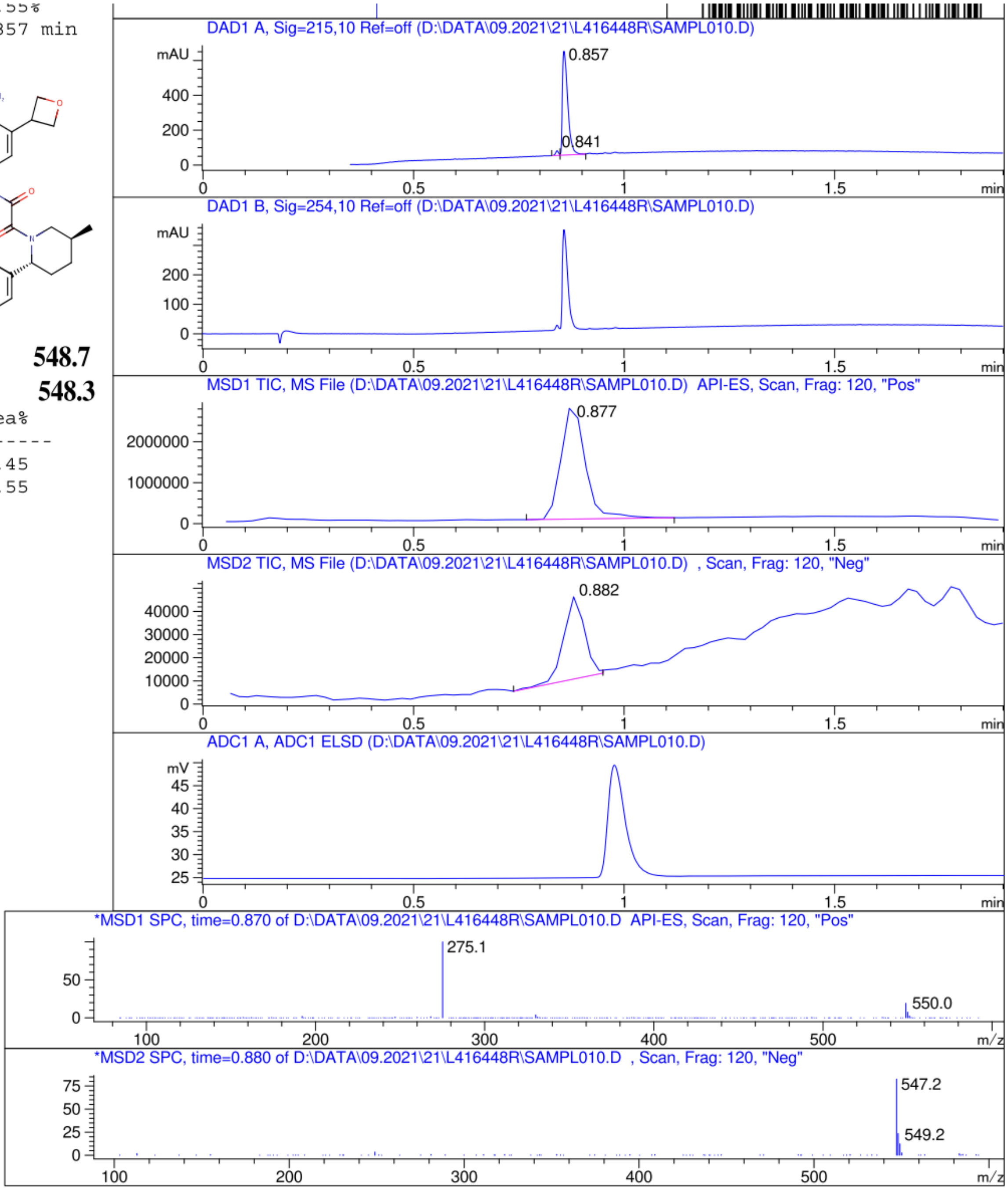

COMPOUND 58

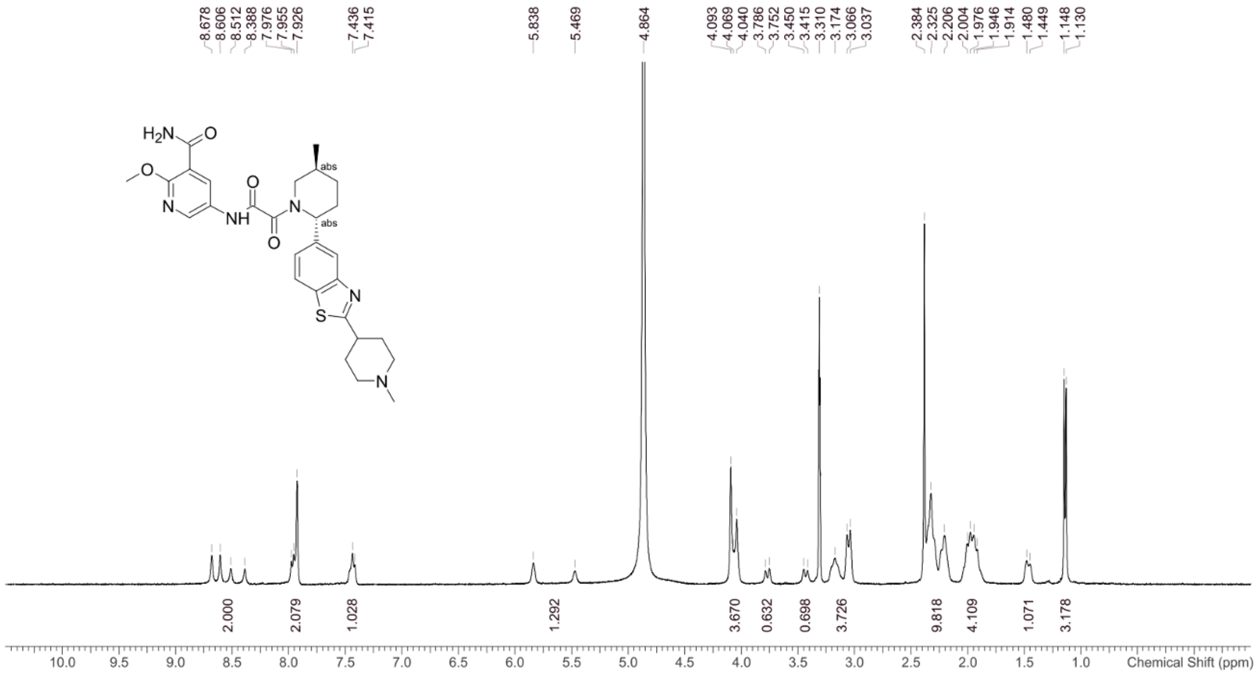
8.678
8.606
8.512
8.388
7.976
7.955
7.926
7.436
7.415
5.838
5.469
4.864
4.093
4.069
4.040
3.786
3.752
3.450
3.415
3.310
3.174
3.066
3.037
2.384
2.325
2.206
2.004
1.976
1.946
1.914
1.480
1.449
1.148
1.130
2.000
2.079
1.028
1.292
3.670
0.632
0.698
3.726
9.818
4.109
1.071
3.178
10.0
9.5
9.0
8.5
8.0
7.5
7.0
6.5
6.0
5.5
5.0
4.5
4.0
3.5
3.0
2.5
2.0
1.5
1.0
Chemical Shift (ppm)

COMPOUND 59

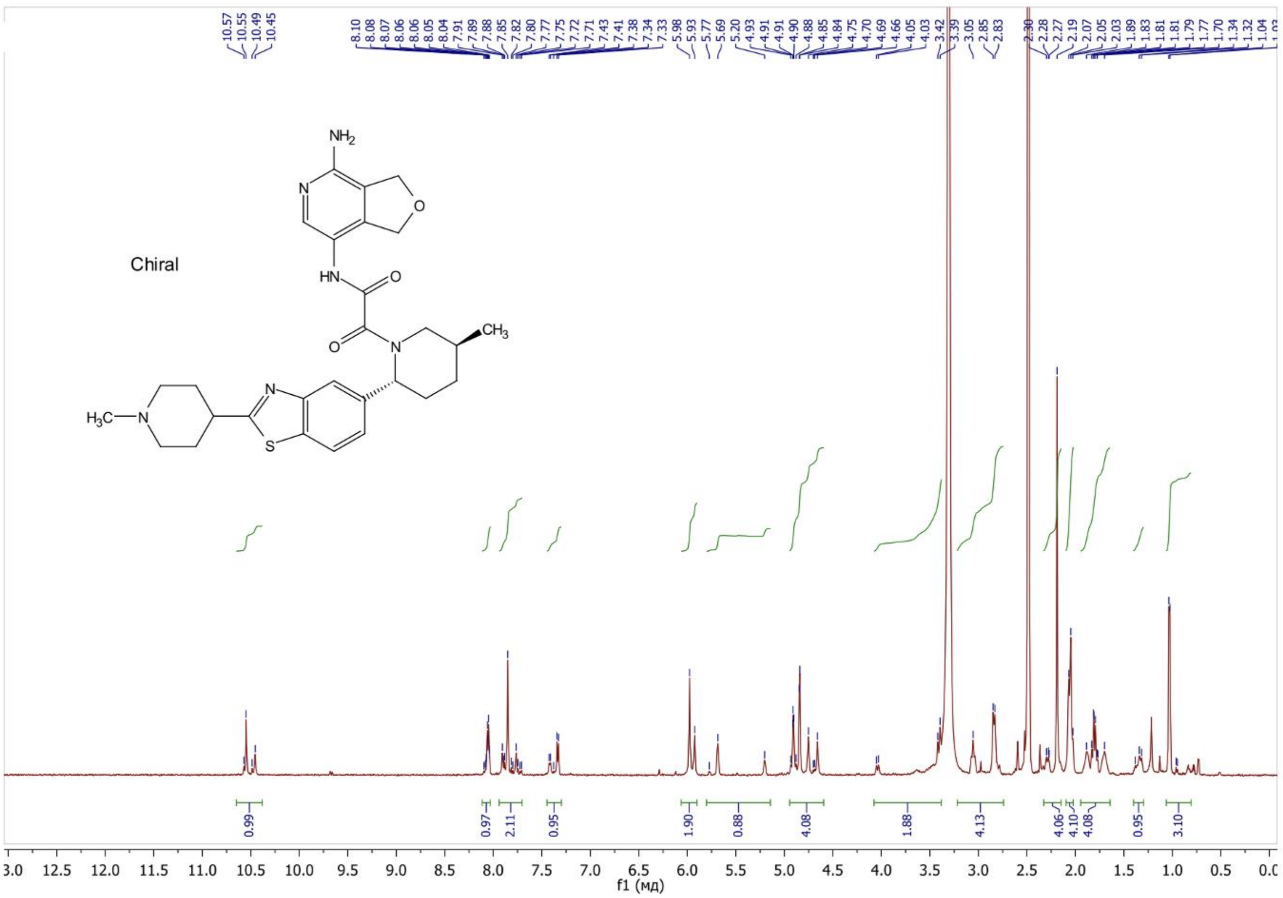
Chiral
f1 (мд)

MaxPeak: 91.52%
Ret_Time: 2.065 min

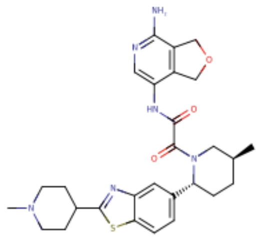

Mol Wt 534.67

Exact Mass 534.28

#	Time	Area%
1	2.065	91.52
2	2.145	8.48

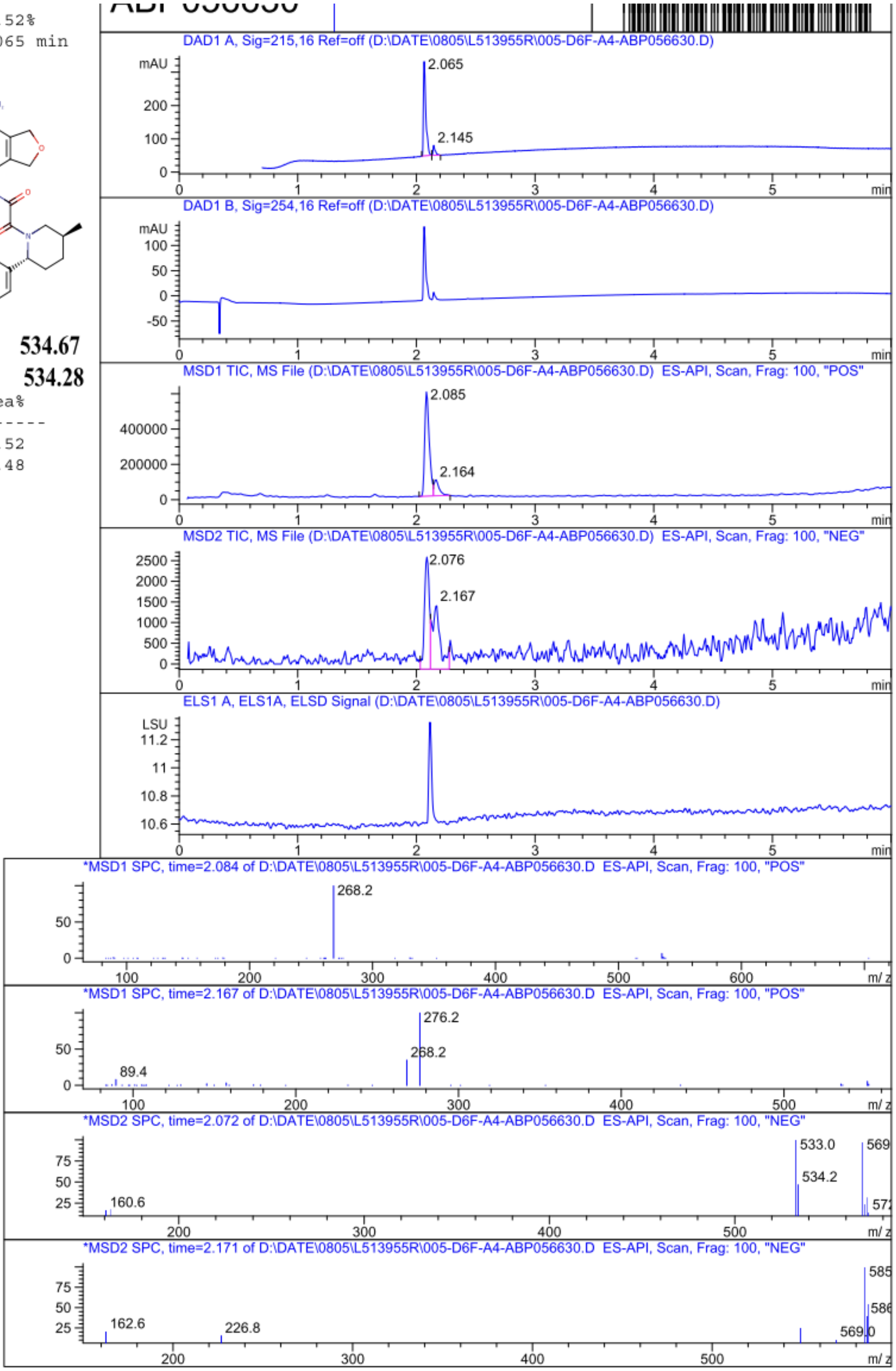

RT 2.085

RT 2.164

RT 2.076

RT 2.167

COMPOUND 60

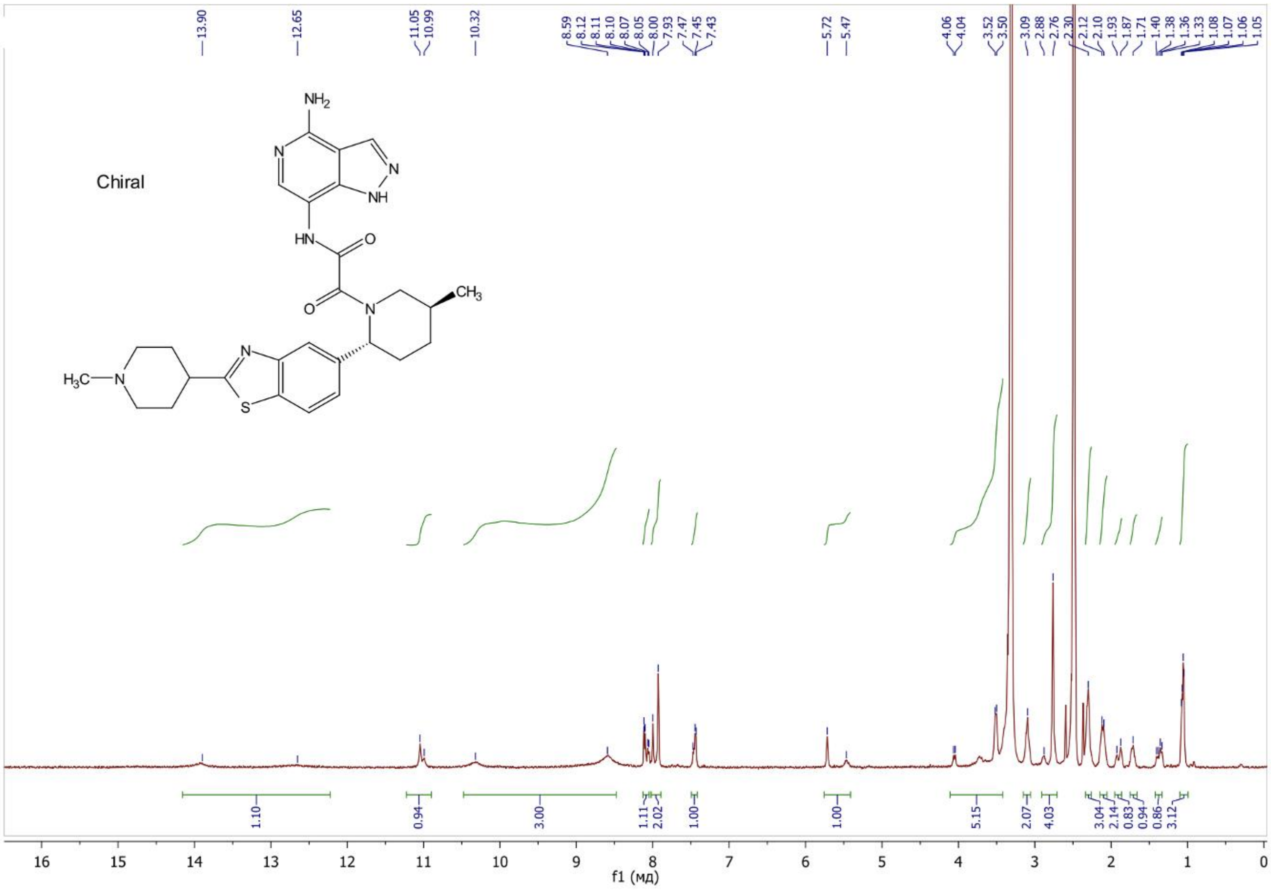
Chiral
13.90
12.65
11.05
10.99
10.32
8.59
8.12
8.11
8.10
8.07
8.05
8.00
7.93
7.47
7.45
7.43
5.72
5.47
4.06
4.04
3.52
3.50
3.09
2.88
2.76
2.30
2.12
2.10
1.93
1.87
1.71
1.40
1.38
1.36
1.33
1.08
1.07
1.06
1.05
1.10
0.94
3.00
1.11
2.02
1.00
1.00
5.15
2.07
4.03
3.04
2.14
0.83
0.94
0.86
3.12
f1 (мд)

Ret_Time: 2.025 min

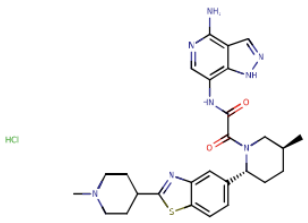

Mol Wt 569.12

Exact Mass 532.27

#	Time	Area%
1	2.025	100.00

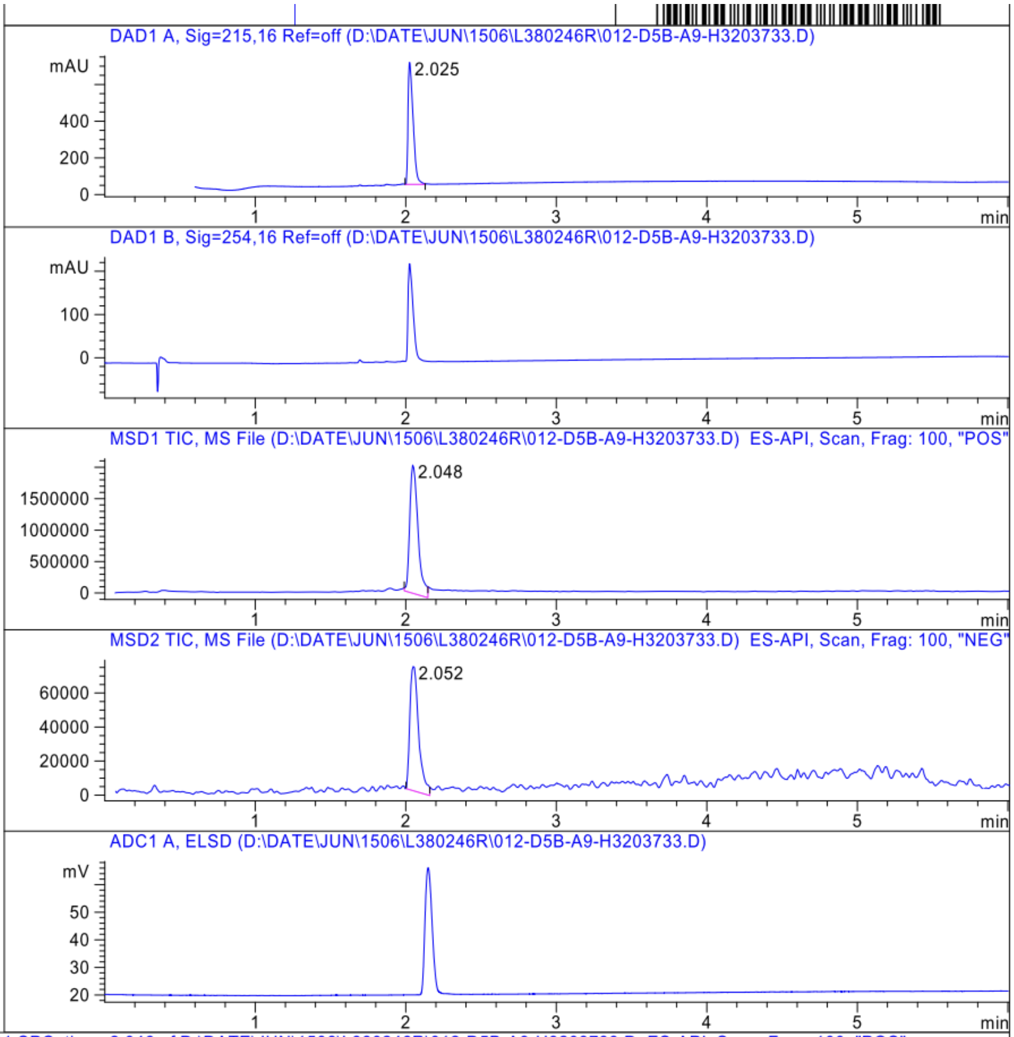

RT 2.048

RT 2.052

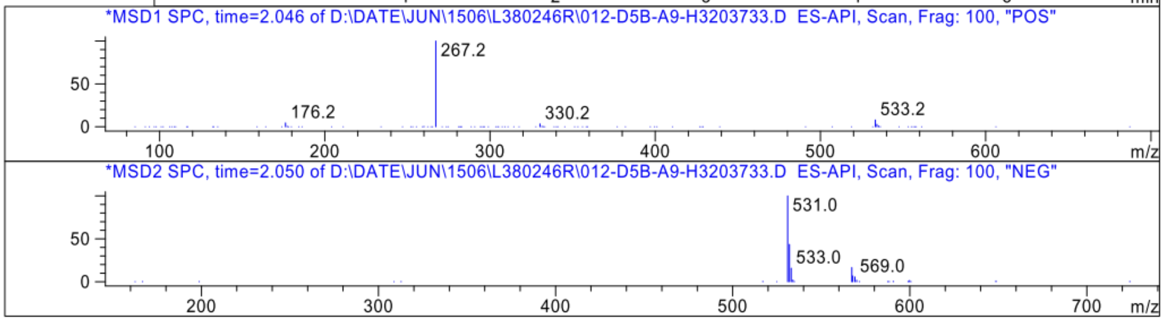

COMPOUND 61

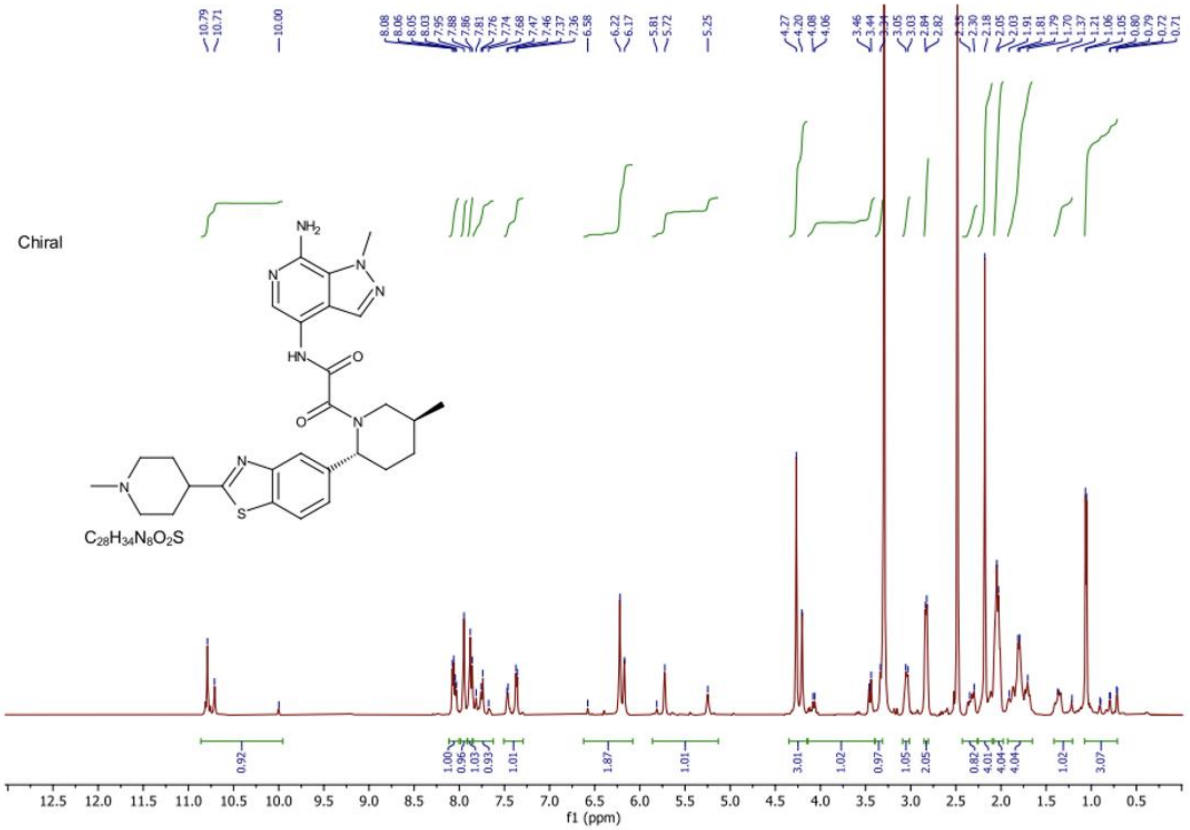
Chiral
$C_{28}H_{34}N_8O_2S$
f1 (ppm)

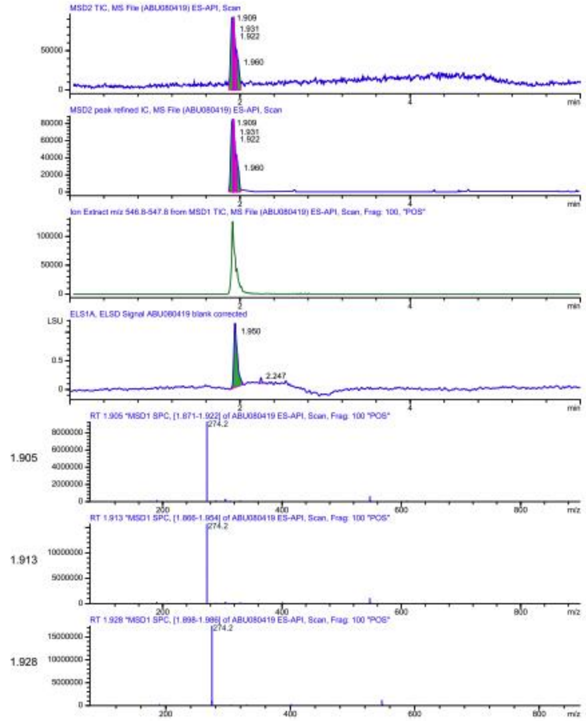

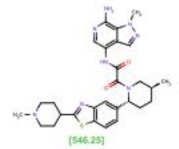

ABU080419

L560324
LCMS-28
MANUAL_6min_4-6x30_1-5_V.M
10:49 21.12.2022
MaxPeak: 97.0%
Several product peaks

#	RT	DAD1A	DAD1B	MSD1	MSD2	ELSD	MSD1 ions	MSD1 rt	MSD2 ions	MSD2 rt	Info
1	1.892	—	—	38.9%	35.4%	—	274.2(100)	1.905	545.2(100)	1.909	P +2H+,P NEG
2	1.902	100.0%	100.0%	5.5%	—	97.0%	547.2(84),190.0(16)	1.913	—	—	P +H+
3	1.909	—	—	—	45.3%	—	—	—	545.2(100)	1.922	P NEG
4	1.915	—	—	26.5%	1.0%	—	274.2(90),401.2(7),548.2(3)	1.928	547.2(100)	1.931	P +2H+
5	1.947	—	—	—	18.3%	—	—	—	545.2(100)	1.960	P NEG
6	1.954	—	—	29.1%	—	—	274.2(100)	1.967	—	—	P +2H+
7	2.199	—	—	—	—	3.0%	—	—	—	—	

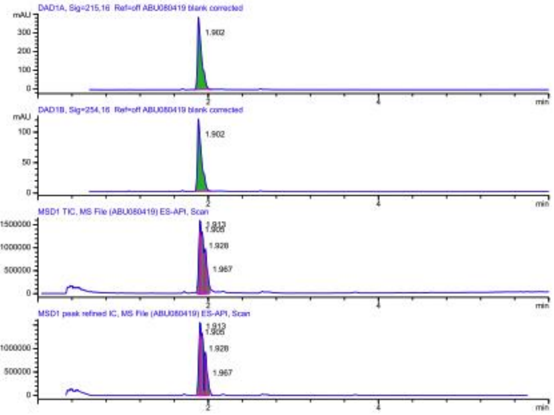

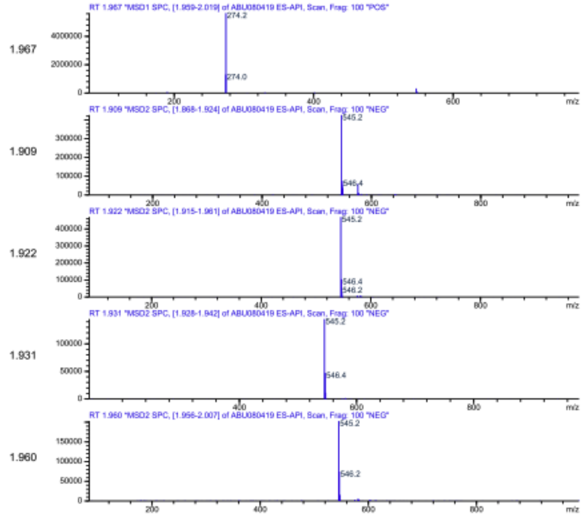

COMPOUND 62

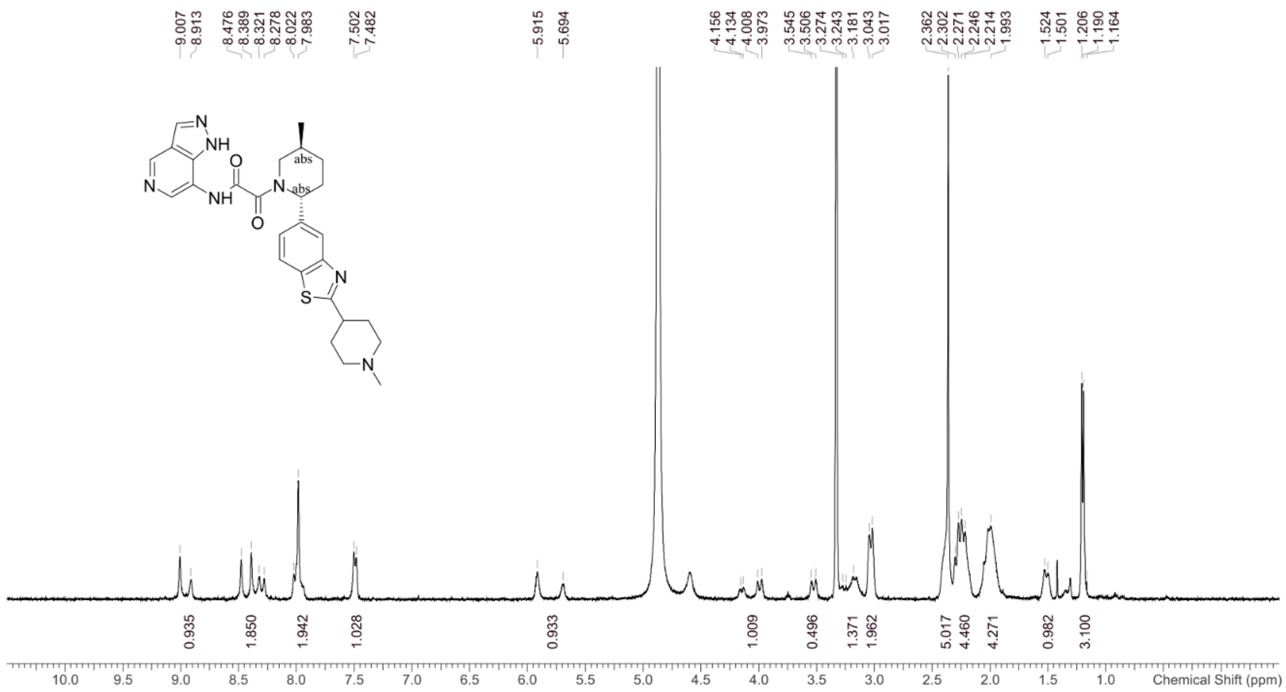

TNG462

1H NMR TNG462

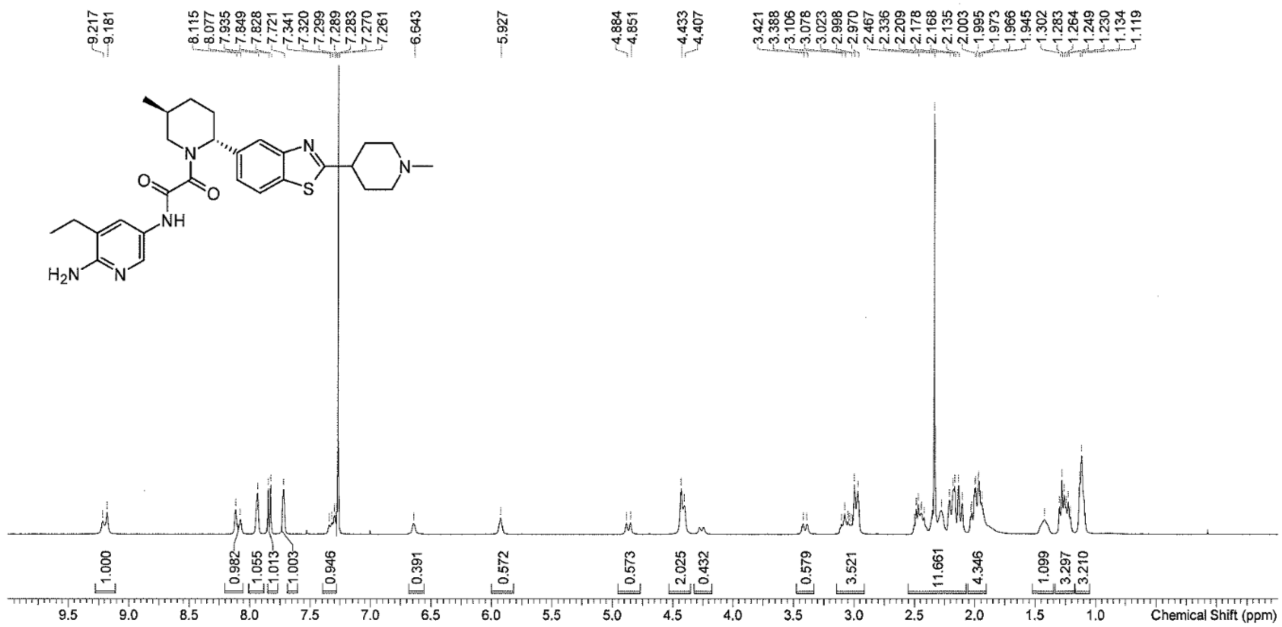

13C NMR – TNG462

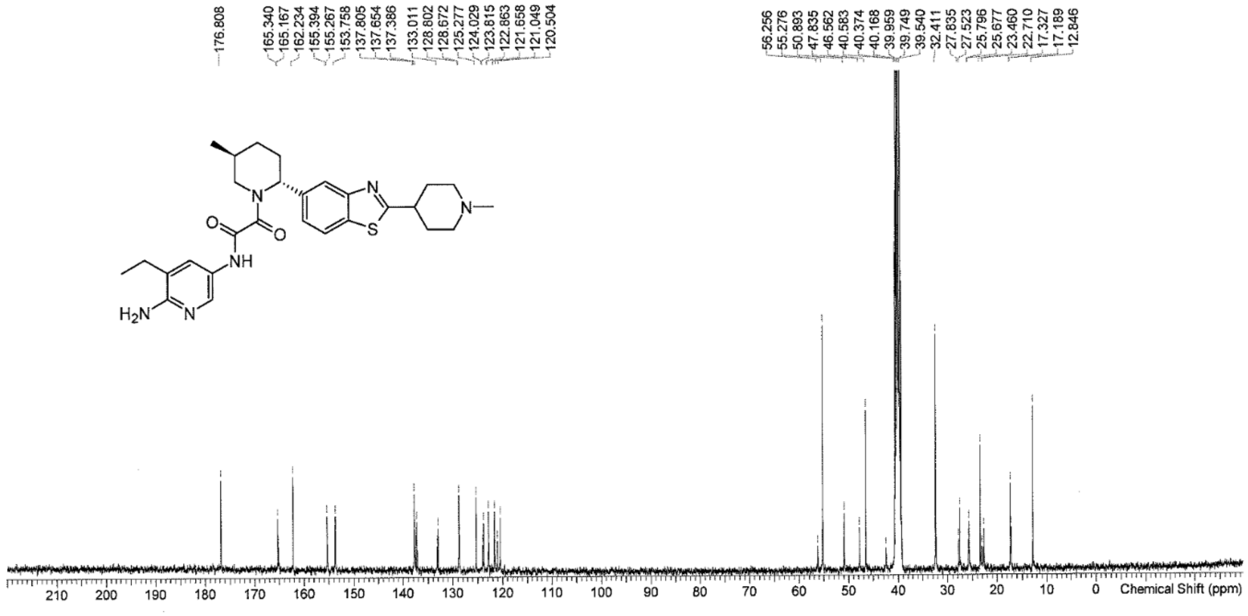

HIGH RESOLUTION MASS SPEC – TNG462

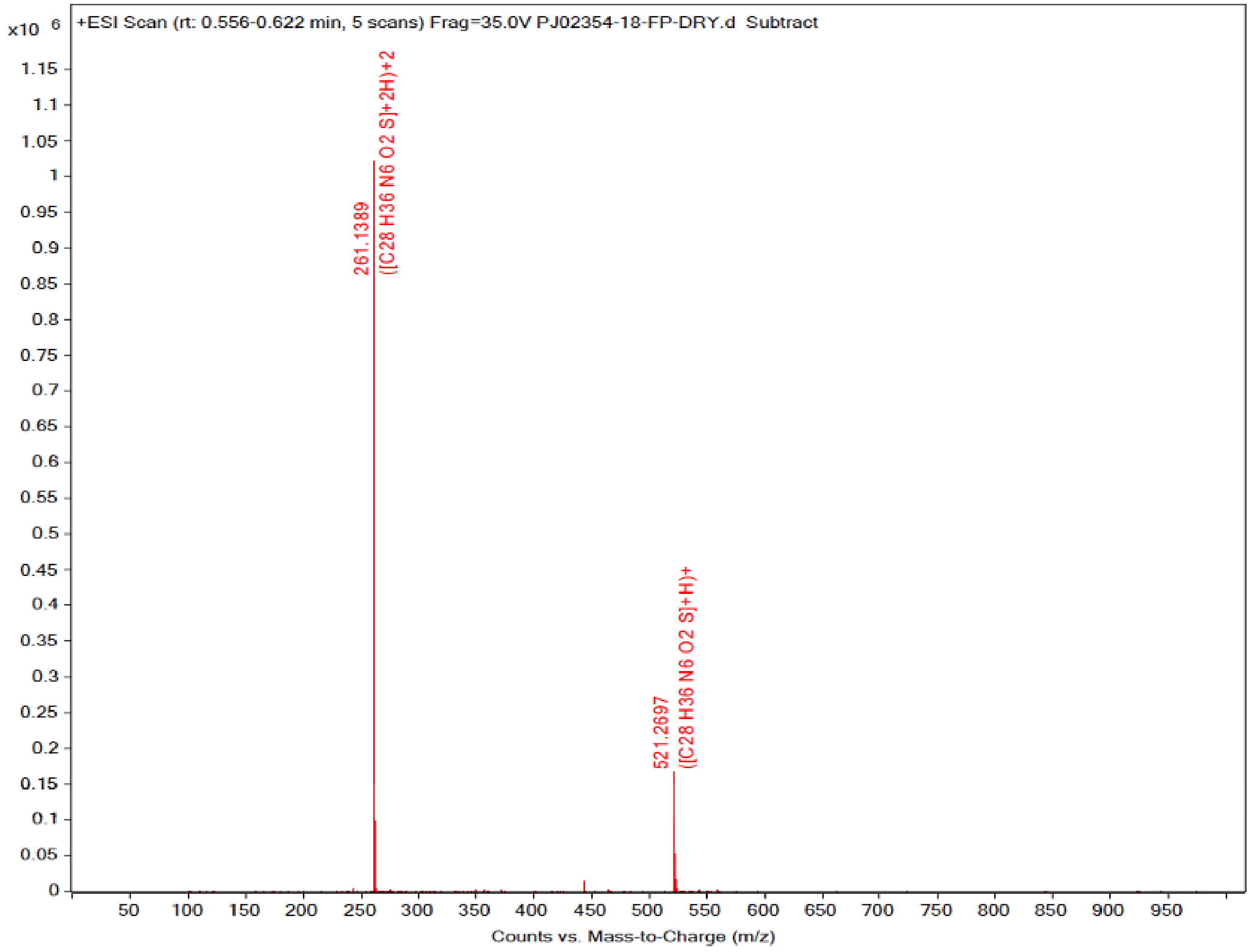

HPLC – TNG462

Method Info :Instrument:Shimadzu LC-20AD
Column :Gemini NX-C18 4.6*150mm 5um
Column Temp:40°C
Mobile Phase:A:0.04%TFA in H2O
Mobile Phase:B:0.02%TFA in ACN
Flow Rate:1.2 mL/min(0.01-19min,2mL/min(19.01-21.00min)

Time	B%	Flow(ml/min)
0.01	10	1.2
16.00	80	1.2
19.00	100	1.2
19.01	10	2
21.00	10	2

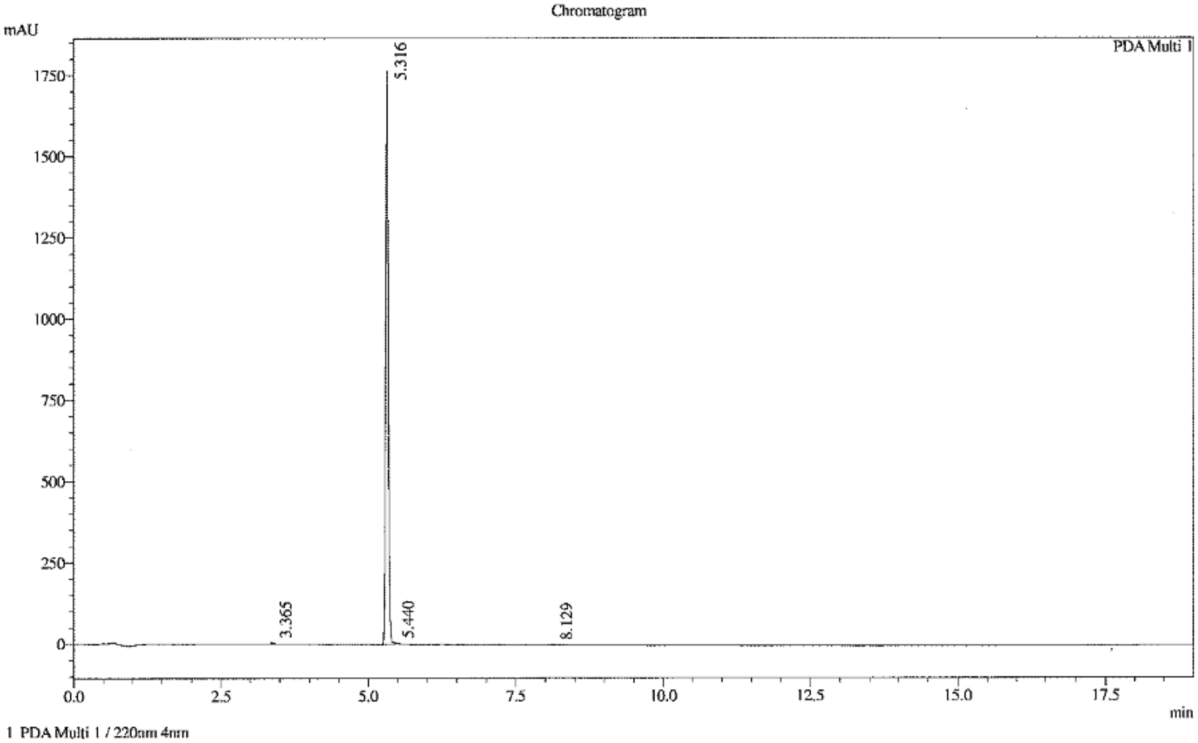

Integration Result

PDA Ch1 220nm 4nm

Peak#	Ret. Time	Height	Height %	USP Width	Area	Area %
1	3.365	5445	0.307	0.068	13214	0.252
2	5.316	1766025	99.458	0.081	5229544	99.558
3	5.440	1392	0.078	0.049	2364	0.045
4	8.129	2786	0.157	0.077	7655	0.146

Method Info :Instrument:Shimadzu LC-20AD
Column :Gemini NX-C18 4.6*150mm 5um
Column Temp:40°C
Mobile Phase:A:0.04%TFA in H2O
Mobile Phase:B:0.02%TFA in ACN
Flow Rate:1.2 mL/min(0.01-19min,2mL/min(19.01-21.00min)

Time	B%	Flow(ml/min)
0.01	10	1.2
16.00	80	1.2
19.00	100	1.2
19.01	10	2
21.00	10	2

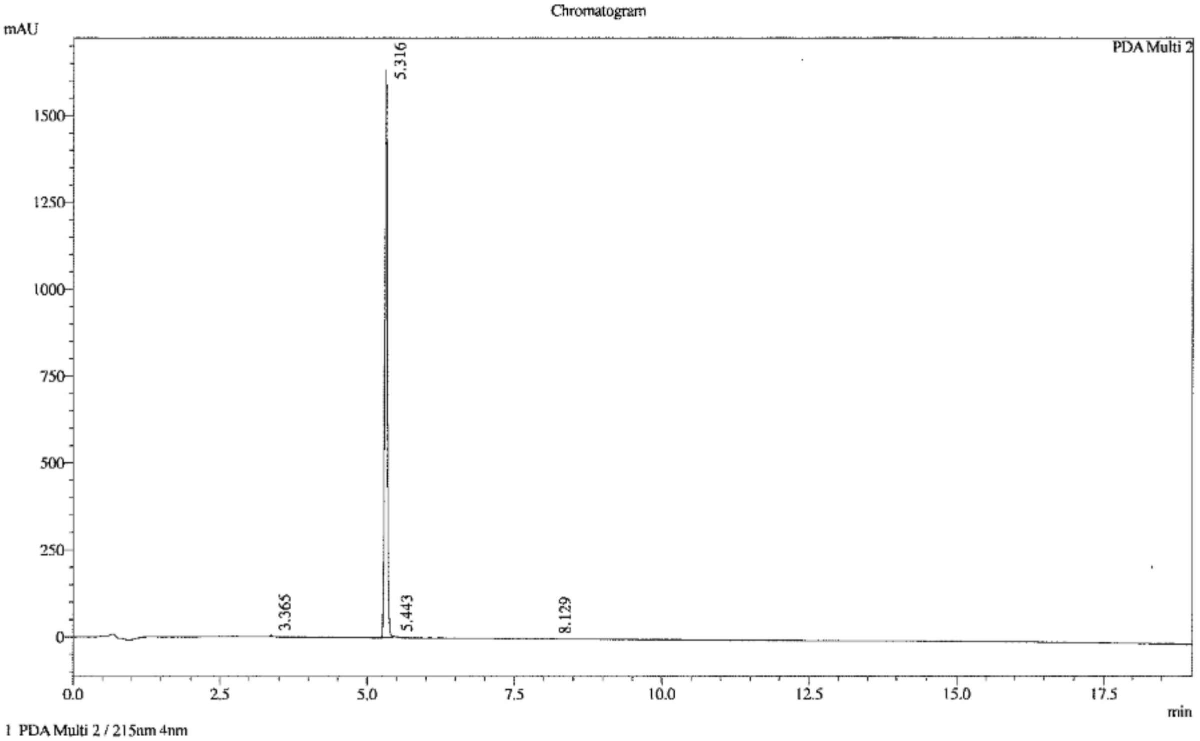

1 PDA Multi 2 / 215nm 4nm

Integration Result

PDA Ch2 215nm 4nm

Peak#	Ret. Time	Height	Height %	USP Width	Area	Area %
1	3.365	4690	0.286	0.067	11353	0.235
2	5.316	1632774	99.488	0.081	4804957	99.583
3	5.443	1305	0.080	0.048	2105	0.044
4	8.129	2416	0.147	0.076	6669	0.138

Method Info :Instrument:Shimadzu LC-20AD
Column :Gemini NX-C18 4.6*150mm 5um
Column Temp:40°C
Mobile Phase:A:0.04%TFA in H2O
Mobile Phase:B:0.02%TFA in ACN
Flow Rate:1.2 mL/min(0.01-19min,2mL/min(19.01-21.00min)

Time	B%	Flow(ml/min)
0.01	10	1.2
16.00	80	1.2
19.00	100	1.2
19.01	10	2
21.00	10	2

Chromatogram

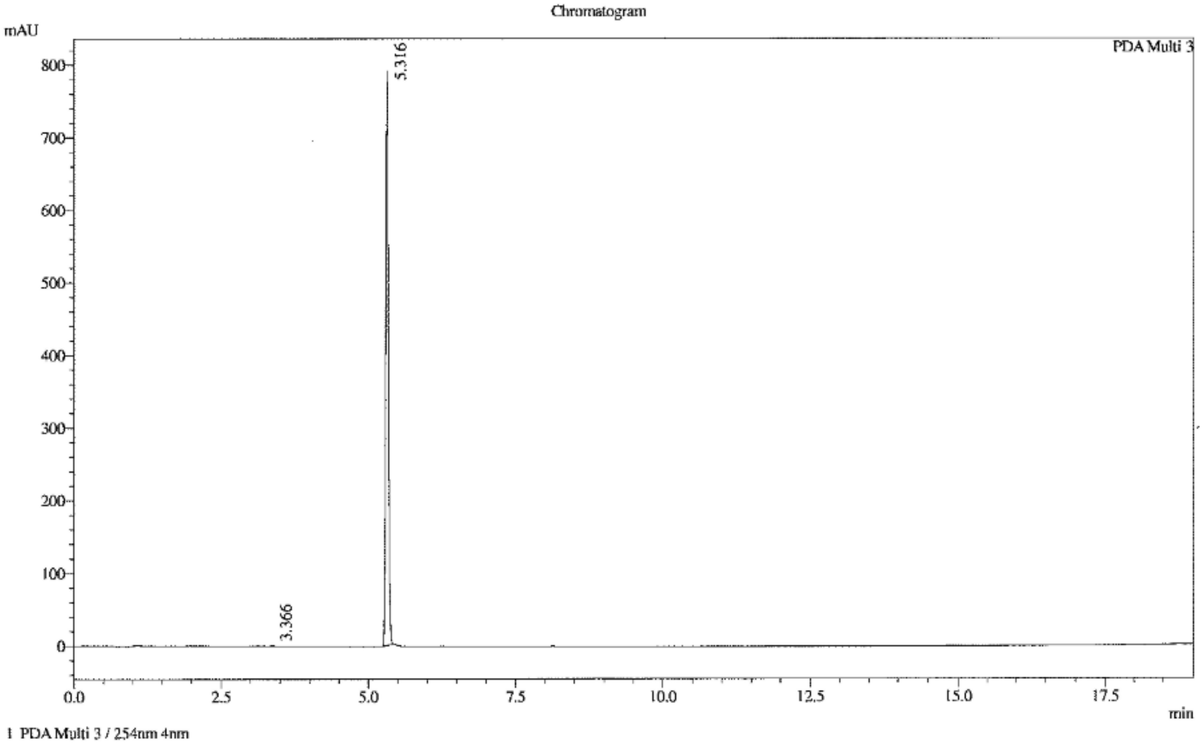

1 PDA Multi 3 / 254nm 4nm

Integration Result

PDA Ch3 254nm 4nm

Peak#	Ret. Time	Height	Height %	USP Width	Area	Area %
1	3.366	1187	0.150	0.065	2689	0.111
2	5.316	791102	99.850	0.083	2409449	99.889

LCMS – TNG462

Method Info : Instrument:Agilent 1200 HPLC MSD:6110 single quadrupole MSD
Column: Luna C18,2.0*50mm, 5μm
Column Temp: 40 °C
Mobile Phase:A:0.04%TFA in H2O
Mobile Phase:B:0.02%TFA in ACN
Flow Rate: 1ml/min

Time	B%	Flow(ml/min)
0.01	5	1
0.40	5	1
3.00	95	1
4.00	95	1
4.01	5	1
4.50	5	1

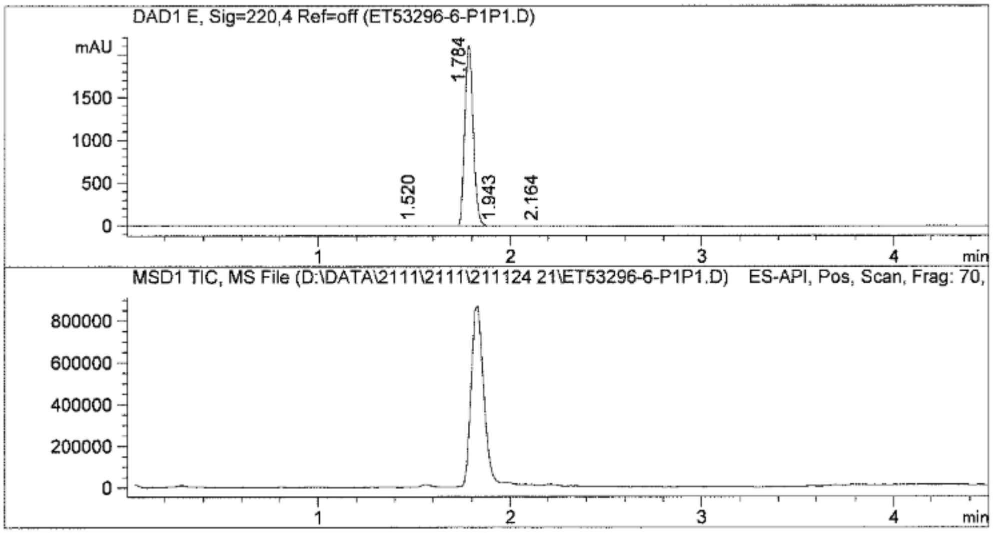

Report

Signal 1 : DAD1 E, Sig=220,4 Ref=off

Peak #	RT [min]	Height	Height %	Width [min]	Area	Area %
1	1.520	7.939	0.372	0.043	21.223	0.321
2	1.784	2115.402	99.035	0.050	6525.656	98.686
3	1.943	6.497	0.304	0.082	38.888	0.588
4	2.164	6.167	0.289	0.063	26.770	0.405

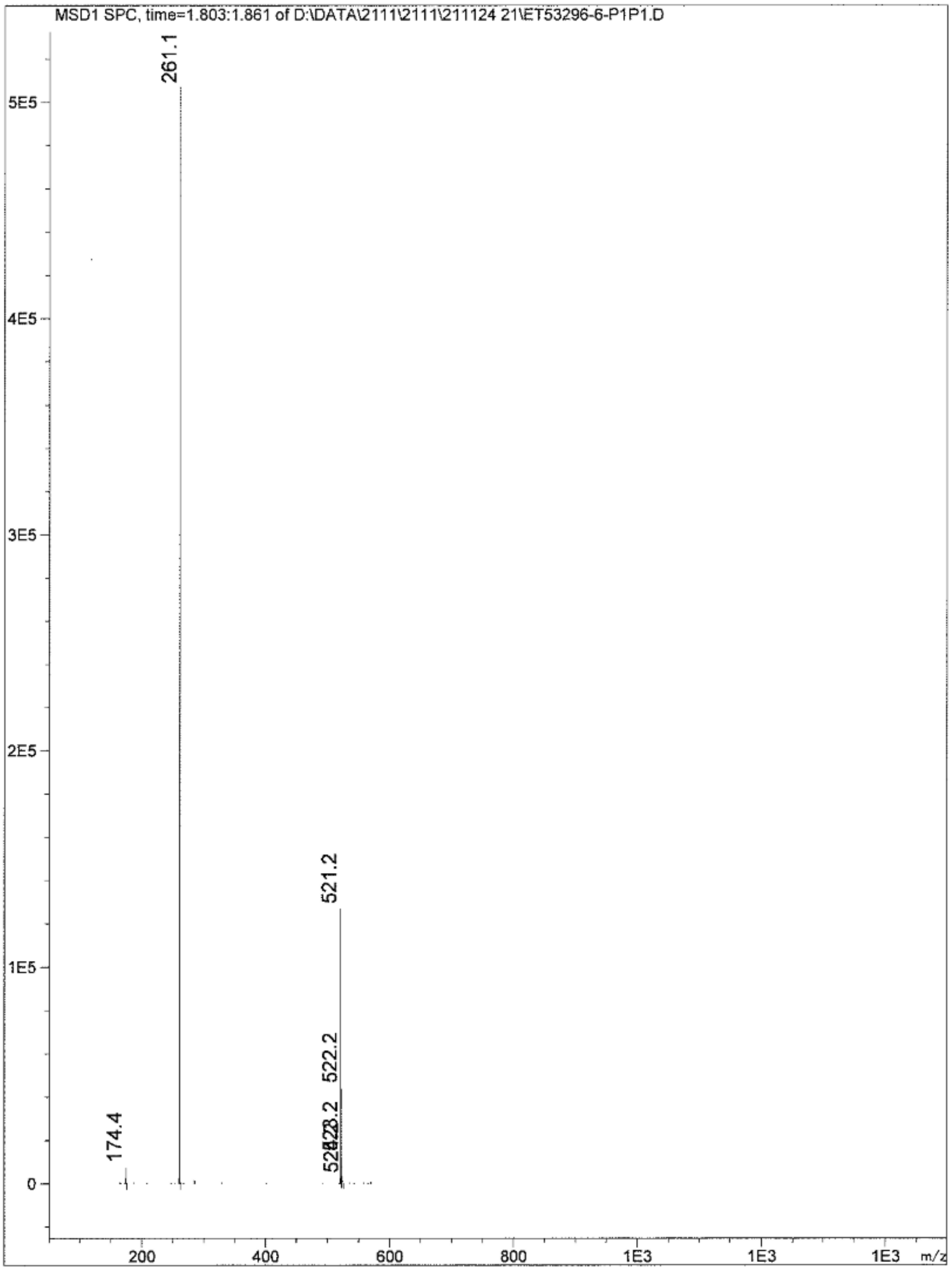

OPTICAL ROTATION – TNG462

Optical Rotation Report

Solvent : EtOH

Set Temperature : 20.0

n	Average	Std.Dev.	% RSD	Maximum	Minimum
2	158.68	0.00	0.00	158.68	158.68

S.No	Result	Scale	OR °Arc	WLG.nm	Lg.mm	Conc.g/100ml	Temp.
1	158.68	SR	0.096	589	50.00	0.121	20.0
2	158.68	SR	0.096	589	50.00	0.121	20.0

CHIRAL HPLC -TNG462

SAMPLE INFORMATION

		Acquired By:	System
		Sample Set Name:	adada
Acq Method :	IC H I MeOH 50 25 25 1 30	Processing Method:	1111111111132g
		Injection Volume:	5.00 ul
		Vial:	1:C,8
Channel Name:	220.0nm	Run Time:	40.0 Minutes
		Raw Data:	D:\Data\ID_m32_result_24446
		Label:	AssayEE
Instrument:	CAS-TJ-ANA-Chiral HPLC-F (Waters Arc with 2998)		
Proc. Chnl. Descr.:	2998 PDA 220.0 nm (2998 (210-400)nm)		

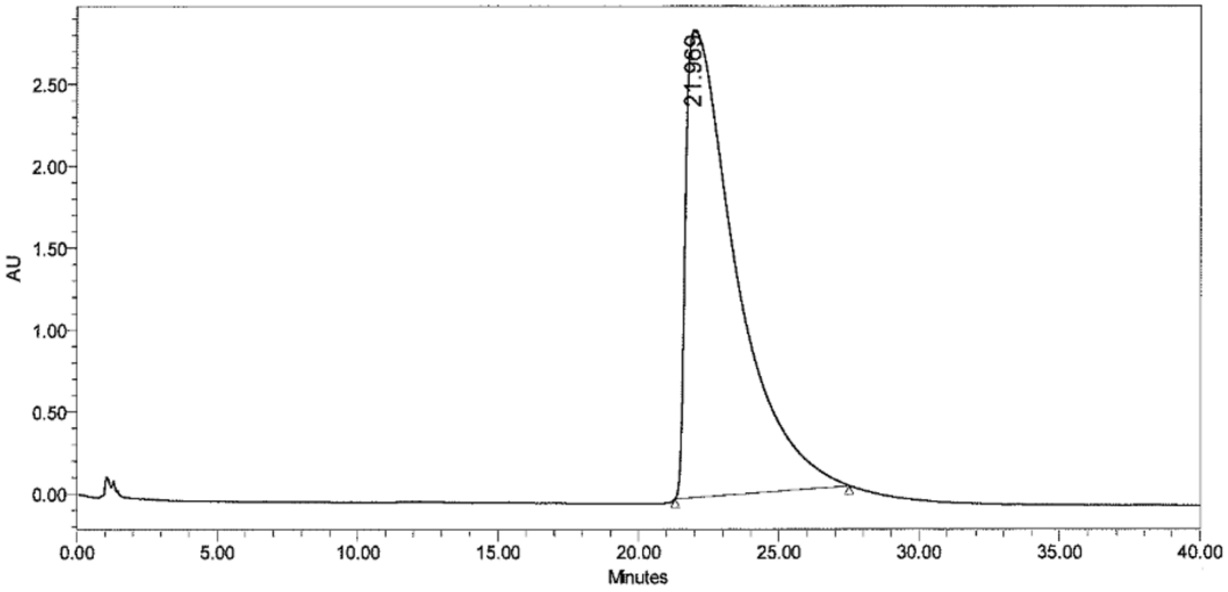

	RT	Area	% Area	Height
1	21.969	352770330	100.00	2854462

FT-IR SPECTRUM – TNG462

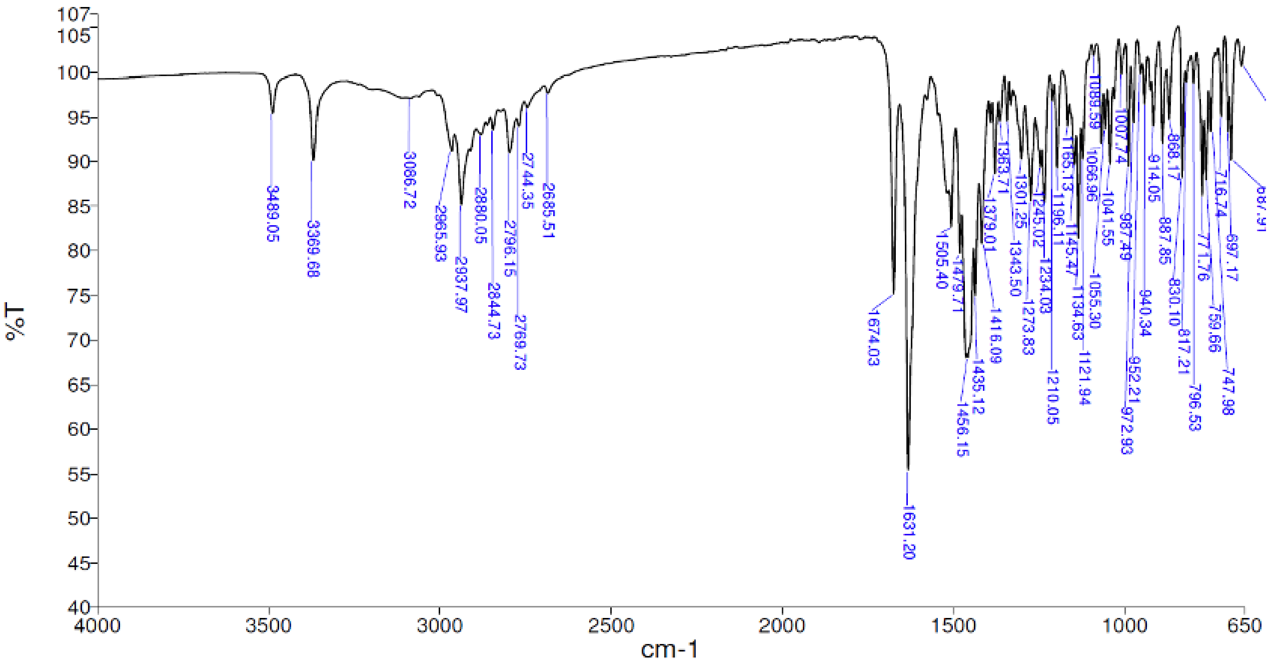

DSC THERMOGRAM – TNG462

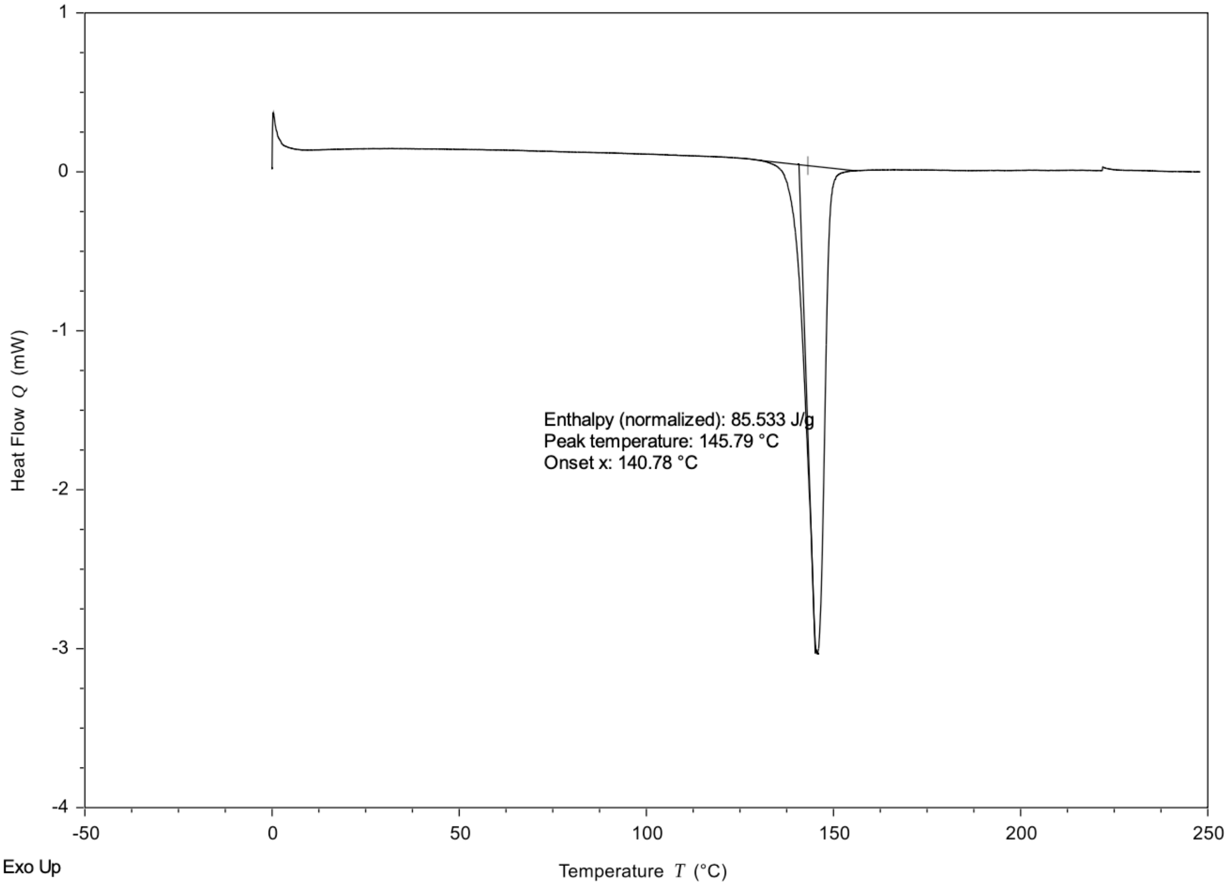

ELEMENTAL ANALYSIS – TNG462

Table SI-1. Elemental analysis data for TNG462

Analysis	Carbon (%)	Hydrogen (%)	Nitrogen (%)
Found value[a]	64.33	6.92	15.91
Theoretical value[b]	64.59	6.97	16.14
Absolute difference	0.3	0.1	0.2

[a]Found value was based on an average of 2 determinations. [b]Theoretical value was based on a 100% pure sample of TNG462. The residual water, residual solvents, and impurities were not taken into consideration.

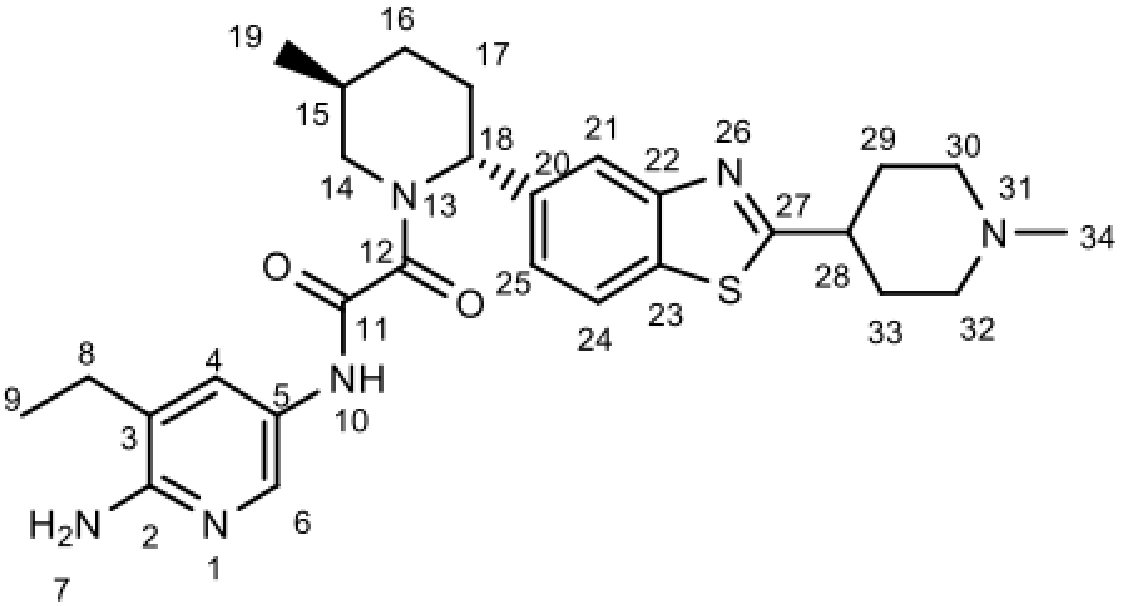

Table SI-2. ^{1}H, ^{13}C spectroscopic data for TNG462 in $CDCl_3$

Number	δ_H (ppm) (mult, J in Hz)[a]	δ_C (ppm)	Relative Intensity[b]
1 (N)	-	-	-
2	-	154.27	-
2	-	154.20	-
3	-	122.39	-
4	7.67 s	129.10	1H
4[c]	-	128.90	-
5	-	126.01	-
6	8.09 s	137.81	1H
6	8.04 s	137.56	-
7 (NH_2)	4.50 s	-	2H
7 (NH_2)	4.47 s	-	-
8	2.42 ov	23.79	2H
9	1.22 (t, J = 7.3 Hz)	12.12	3H
9	1.17 (t, J = 7.0 Hz)	-	-
10 (NH)	9.43 s	-	1H
11	-	159.21	-
12	-	163.00	-
12	-	162.77	-
13 (N)	-	-	-
14a	4.69 (d, J = 13.7 Hz)	47.49	1H

Number	δ_H (ppm) (mult, J in Hz)[a]	δ_C (ppm)	Relative Intensity[b]
14a	4.22 (d, J = 12.7 Hz)	44.65	-
14b	3.36 (d, J = 12.5 Hz)	47.49	1H
14b	2.96 ov	44.65	-
15	1.92 ov	28.36	-
16	1.91 ov<br>1.39 m	25.70	2H
16	-	25.51	-
17	2.23 br s	22.91	2H
17[c]	2.30 ov	23.63	-
18	5.90 s	53.28	1H
18	6.49 s	56.08	-
19	1.09 ov	17.00	3H
19	1.06 ov	16.93	-
20	-	136.97	-
20	-	137.46	-
21	7.90 s	120.52	1H
21	7.92 s	120.60	-
22	-	153.67	-
22	-	153.74	-
23	-	133.27	-
24	7.78 (d, J = 8.0 Hz)	122.01	1H
24[c]	7.80 ov	-	-
25	7.25 ov	123.55	1H
25[c]	7.31 (d, J = 7.8 Hz)	-	-
26 (N)	-	-	-
27	-	176.99	-
28	3.04 ov	41.09	1H
29a/33a	2.15 ov	32.57	2H

Abbreviations: NMR, nuclear magnetic resonance; ppm, parts per million;

Non-equivalent protons attached to the same atom are designed as "a" and "b".

[a]s = singlet, d = doublet, t = triplet, m = multiplet, ov=overlapped.

[b]Relative integrated intensities of proton resonances were measured from 1-D proton NMR spectrum.

[c]A minor rotamer exists in solution and exhibits slightly different chemical shifts in the ^{1}H-NMR and ^{13}C-NMR spectra. The minor rotamer can be distinguished by peak intensity.

SMALL MOLECULE CRYSTAL STRUCTURE OF TNG462 (FIGURE SI-1)

The single crystal X-ray diffraction studies were carried out on a Bruker Kappa Photon III CPAD diffractometer equipped with Mo K_α radiation (λ = 0.71073 Å). Crystals of TNG462 were grown by dissolving approximately 1mg of sample in 350µL of 90/10 dichloroethane / methanol solution, which was then vapor diffused with pentane over several days. A 0.249 x 0.074 x 0.024 mm piece of a colorless blade was mounted on a Cryoloop with Paratone 24EX oil. Data were collected in a nitrogen gas stream at 100(2) K using ϕ and ϖ scans. Crystal-to-detector distance was 50 mm using variable exposure time (5s-15s) depending on θ with a scan width of 1.0 °. Data collection was 99.8 % complete to 25.00 ° in θ (0.83 Å). A total of 16914 reflections were collected covering the indices, -12<=h<=12, -7<=k<=7, -25<=l<=25. 5277 reflections were found to be symmetry independent, with a R_{int} of 0.0466. Indexing and unit cell refinement indicated a primitive, monoclinic lattice. The space group was found to be $P2_1$. The data were integrated using the Bruker SAINT software program and scaled using the SADABS software program. Solution by direct methods (SHELXT) produced a complete phasing model for refinement.

All nonhydrogen atoms were refined anisotropically by full-matrix least-squares (SHELXL-2014). All carbon bonded hydrogen atoms were placed using a riding model. Their positions were constrained relative to their parent atom using the appropriate HFIX command in SHELXL-2014. All other hydrogen atoms (H-bonding) were located in the difference map. Their relative positions were restrained using DFIX commands and their thermals freely refined. The absolute stereochemistry of the molecule was established by anomalous dispersion using the Parson's method with a Flack parameter of 0.031(48). Crystallographic data are summarized in Table SI-3.

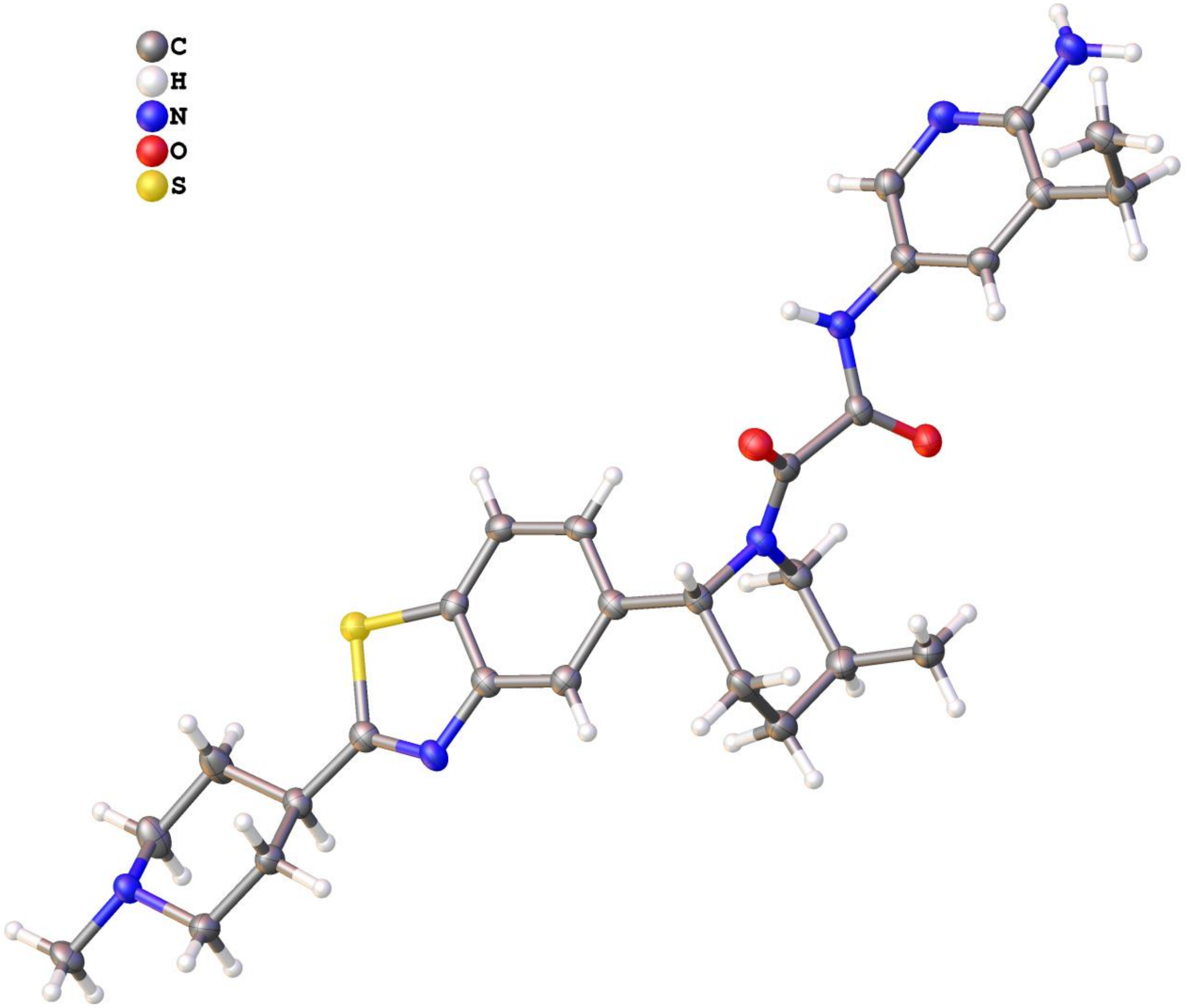

Figure SI-1. Small molecule crystal structure of TNG462.

Table SI-3. Small molecule crystal structure parameters, TNG462.

Empirical formula	C_{28} H_{36} N_6 O_2 S
Molecular formula	C_{28} H_{36} N_6 O_2 S
Formula weight	520.69
Temperature	100.0 K
Wavelength	0.71073 Å
Crystal system	Monoclinic
Space group	P 1 21 1
Unit cell dimensions	a = 10.1504(8) Å α = 90 °
	b = 6.3607(4) Å β = 90.060(2) °

	c = 20.2290(14) Å γ = 90 °
Volume	1306.06(16) $Å^3$
Z	2
Density (calculated)	1.324 Mg/m^3
Absorption coefficient	0.162 mm^{-1}
F(000)	556
Crystal size	0.249 x 0.074 x 0.024 mm^3
Crystal color, habit	Colorless Blade
Theta range for data collection	2.006 to 26.377°.
Index ranges	-12<=h<=12, -7<=k<=7, -25<=l<=25
Reflections collected	16914
Independent reflections	5277 [R(int) = 0.0466, R(sigma) = 0.0493]
Completeness to theta = 68.000°	99.8 %
Absorption correction	Semi-empirical from equivalents
Max. and min. transmission	0.0932 and 0.0664
Refinement method	Full-matrix least-squares on F^2
Data / restraints / parameters	5277 / 4 / 349
Goodness-of-fit on F^2	1.027
Final R indices [I>2sigma(I)]	R1 = 0.0375, wR2 = 0.0766
R indices (all data)	R1 = 0.0498, wR2 = 0.0821
Absolute structure parameter	0.03(5)
Extinction coefficient	n/a
Largest diff. peak and hole	0.209 and -0.228 e.$Å^{-3}$

Table SI-4. Atomic coordinates (x 10^4) and equivalent isotropic displacement parameters ($Å^2$x 10^3) for TNG462. U(eq) is defined as one third of the trace of the orthogonalized U^{ij} tensor.

	x	y	z	U(eq)
S(1)	7274(1)	4806(1)	5256(1)	25(1)
O(1)	1706(2)	7348(3)	2696(1)	28(1)
O(2)	2669(2)	5331(3)	1224(1)	30(1)
N(1)	3881(2)	7026(4)	2466(1)	23(1)
N(2)	7704(2)	8498(4)	4749(1)	23(1)
N(3)	1213(3)	3854(4)	1950(1)	24(1)
N(4)	10537(2)	8760(4)	6899(1)	22(1)
N(5)	-670(3)	-424(4)	1148(1)	28(1)
N(6)	-2141(3)	-474(5)	275(1)	30(1)
C(1)	5008(3)	6252(5)	2082(2)	25(1)
C(2)	5717(3)	8070(5)	1734(2)	32(1)
C(3)	6076(3)	9781(6)	2240(1)	32(1)
C(4)	4885(3)	10450(5)	2652(2)	30(1)
C(5)	4224(3)	8570(5)	2985(1)	24(1)
C(6)	5014(3)	7530(5)	3541(1)	22(1)

C(7)	6013(3)	8576(5)	3866(1)	23(1)
C(8)	6666(3)	7612(5)	4396(1)	22(1)
C(9)	6288(3)	5586(5)	4599(1)	23(1)
C(10)	5279(3)	4520(5)	4278(1)	24(1)
C(11)	4653(3)	5506(5)	3751(1)	23(1)
C(12)	4899(4)	8952(6)	1164(2)	38(1)
C(13)	2600(3)	6603(5)	2357(1)	22(1)
C(14)	2184(3)	5164(5)	1780(1)	23(1)
C(15)	8119(3)	7213(5)	5205(1)	23(1)
C(16)	9270(3)	7684(5)	5649(1)	24(1)
C(17)	9181(3)	9868(5)	5947(1)	25(1)
C(18)	10382(3)	10323(5)	6373(2)	27(1)
C(19)	9481(4)	6076(5)	6197(2)	35(1)
C(20)	10682(4)	6665(5)	6610(2)	34(1)
C(21)	11692(3)	9285(5)	7296(2)	30(1)
C(22)	426(3)	2757(5)	1472(1)	24(1)
C(23)	-197(3)	3845(5)	962(1)	25(1)
C(24)	-1048(3)	2805(5)	542(1)	24(1)
C(25)	-1252(3)	642(5)	656(1)	25(1)
C(26)	162(3)	647(5)	1543(2)	27(1)
C(27)	-1834(3)	3970(5)	27(2)	27(1)
C(28)	-3219(3)	4460(5)	284(2)	33(1)

Table SI-5. Bond lengths [Å] and angles [°] for TNG462.

S(1)-C(9)	1.736(3)	C(6)-C(7)	1.379(4)
S(1)-C(15)	1.758(3)	C(6)-C(11)	1.405(4)
O(1)-C(13)	1.233(4)	C(7)-H(7)	0.9500
O(2)-C(14)	1.234(3)	C(7)-C(8)	1.401(4)
N(1)-C(1)	1.468(4)	C(8)-C(9)	1.406(4)
N(1)-C(5)	1.480(4)	C(9)-C(10)	1.389(4)
N(1)-C(13)	1.345(4)	C(10)-H(10)	0.9500
N(2)-C(8)	1.392(4)	C(10)-C(11)	1.390(4)
N(2)-C(15)	1.302(4)	C(11)-H(11)	0.9500
N(3)-H(3)	0.92(2)	C(12)-H(12A)	0.9800
N(3)-C(14)	1.336(4)	C(12)-H(12B)	0.9800
N(3)-C(22)	1.434(4)	C(12)-H(12C)	0.9800
N(4)-C(18)	1.464(4)	C(13)-C(14)	1.542(4)
N(4)-C(20)	1.462(4)	C(15)-C(16)	1.502(4)
N(4)-C(21)	1.460(4)	C(16)-H(16)	1.0000
N(5)-C(25)	1.340(4)	C(16)-C(17)	1.517(4)
N(5)-C(26)	1.347(4)	C(16)-C(19)	1.524(4)
N(6)-H(6A)	0.94(2)	C(17)-H(17A)	0.9900
N(6)-H(6B)	0.93(2)	C(17)-H(17B)	0.9900

N(6)-C(25)	1.382(4)	C(17)-C(18)	1.519(4)
C(1)-H(1A)	0.9900	C(18)-H(18A)	0.9900
C(1)-H(1B)	0.9900	C(18)-H(18B)	0.9900
C(1)-C(2)	1.533(4)	C(19)-H(19A)	0.9900
C(2)-H(2)	1.0000	C(19)-H(19B)	0.9900
C(2)-C(3)	1.537(5)	C(19)-C(20)	1.524(5)
C(2)-C(12)	1.527(4)	C(20)-H(20A)	0.9900
C(3)-H(3A)	0.9900	C(20)-H(20B)	0.9900
C(3)-H(3B)	0.9900	C(21)-H(21A)	0.9800
C(3)-C(4)	1.530(5)	C(21)-H(21B)	0.9800
C(4)-H(4A)	0.9900	C(21)-H(21C)	0.9800
C(4)-H(4B)	0.9900	C(22)-C(23)	1.395(4)
C(4)-C(5)	1.529(4)	C(22)-C(26)	1.376(4)
C(5)-H(5)	1.0000	C(23)-H(23)	0.9500
C(5)-C(6)	1.530(4)	C(23)-C(24)	1.380(4)
C(24)-C(25)	1.411(4)	C(12)-C(2)-C(3)	111.8(3)
C(24)-C(27)	1.505(4)	C(2)-C(3)-H(3A)	109.2
C(26)-H(26)	0.9500	C(2)-C(3)-H(3B)	109.2
C(27)-H(27A)	0.9900	H(3A)-C(3)-H(3B)	107.9
C(27)-H(27B)	0.9900	C(4)-C(3)-C(2)	111.9(3)
C(27)-C(28)	1.531(4)	C(4)-C(3)-H(3A)	109.2
C(28)-H(28A)	0.9800	C(4)-C(3)-H(3B)	109.2
C(28)-H(28B)	0.9800	C(3)-C(4)-H(4A)	109.3
C(28)-H(28C)	0.9800	C(3)-C(4)-H(4B)	109.3
C(9)-S(1)-C(15)	89.25(15)	H(4A)-C(4)-H(4B)	107.9
C(1)-N(1)-C(5)	114.5(2)	C(5)-C(4)-C(3)	111.7(3)
C(13)-N(1)-C(1)	126.9(2)	C(5)-C(4)-H(4A)	109.3
C(13)-N(1)-C(5)	118.4(2)	C(5)-C(4)-H(4B)	109.3
C(15)-N(2)-C(8)	110.7(3)	N(1)-C(5)-C(4)	108.0(2)
C(14)-N(3)-H(3)	117(2)	N(1)-C(5)-H(5)	107.3
C(14)-N(3)-C(22)	122.8(2)	N(1)-C(5)-C(6)	111.0(2)
C(22)-N(3)-H(3)	117(2)	C(4)-C(5)-H(5)	107.3
C(20)-N(4)-C(18)	109.9(2)	C(4)-C(5)-C(6)	115.6(3)
C(21)-N(4)-C(18)	109.3(2)	C(6)-C(5)-H(5)	107.3
C(21)-N(4)-C(20)	110.3(2)	C(7)-C(6)-C(5)	121.8(3)
C(25)-N(5)-C(26)	117.4(3)	C(7)-C(6)-C(11)	119.3(3)
H(6A)-N(6)-H(6B)	112(4)	C(11)-C(6)-C(5)	118.8(3)
C(25)-N(6)-H(6A)	122(3)	C(6)-C(7)-H(7)	120.0
C(25)-N(6)-H(6B)	112(3)	C(6)-C(7)-C(8)	120.1(3)
N(1)-C(1)-H(1A)	109.5	C(8)-C(7)-H(7)	120.0
N(1)-C(1)-H(1B)	109.5	N(2)-C(8)-C(7)	125.0(3)
N(1)-C(1)-C(2)	110.8(3)	N(2)-C(8)-C(9)	115.3(3)
H(1A)-C(1)-H(1B)	108.1	C(7)-C(8)-C(9)	119.7(3)
C(2)-C(1)-H(1A)	109.5	C(8)-C(9)-S(1)	109.2(2)

C(2)-C(1)-H(1B)	109.5	C(10)-C(9)-S(1)	130.0(2)
C(1)-C(2)-H(2)	107.8	C(10)-C(9)-C(8)	120.8(3)
C(1)-C(2)-C(3)	109.9(2)	C(9)-C(10)-H(10)	120.8
C(3)-C(2)-H(2)	107.8	C(9)-C(10)-C(11)	118.3(3)
C(12)-C(2)-C(1)	111.6(3)	C(11)-C(10)-H(10)	120.8
C(12)-C(2)-H(2)	107.8	C(6)-C(11)-H(11)	119.1
C(10)-C(11)-H(11)	119.1	C(10)-C(11)-C(6)	121.8(3)
C(2)-C(12)-H(12A)	109.5	C(16)-C(19)-C(20)	110.3(3)
C(2)-C(12)-H(12B)	109.5	H(19A)-C(19)-H(19B)	108.1
C(2)-C(12)-H(12C)	109.5	C(20)-C(19)-H(19A)	109.6
H(12A)-C(12)-H(12B)	109.5	C(20)-C(19)-H(19B)	109.6
H(12A)-C(12)-H(12C)	109.5	N(4)-C(20)-C(19)	111.2(3)
H(12B)-C(12)-H(12C)	109.5	N(4)-C(20)-H(20A)	109.4
O(1)-C(13)-N(1)	122.9(3)	N(4)-C(20)-H(20B)	109.4
O(1)-C(13)-C(14)	116.6(3)	C(19)-C(20)-H(20A)	109.4
N(1)-C(13)-C(14)	120.4(3)	C(19)-C(20)-H(20B)	109.4
O(2)-C(14)-N(3)	125.7(3)	H(20A)-C(20)-H(20B)	108.0
O(2)-C(14)-C(13)	122.0(3)	N(4)-C(21)-H(21A)	109.5
N(3)-C(14)-C(13)	112.2(2)	N(4)-C(21)-H(21B)	109.5
N(2)-C(15)-S(1)	115.5(2)	N(4)-C(21)-H(21C)	109.5
N(2)-C(15)-C(16)	123.2(3)	H(21A)-C(21)-H(21B)	109.5
C(16)-C(15)-S(1)	121.2(2)	H(21A)-C(21)-H(21C)	109.5
C(15)-C(16)-H(16)	106.9	H(21B)-C(21)-H(21C)	109.5
C(15)-C(16)-C(17)	112.0(2)	C(23)-C(22)-N(3)	120.7(3)
C(15)-C(16)-C(19)	114.2(3)	C(26)-C(22)-N(3)	120.9(3)
C(17)-C(16)-H(16)	106.9	C(26)-C(22)-C(23)	118.2(3)
C(17)-C(16)-C(19)	109.4(2)	C(22)-C(23)-H(23)	119.9
C(19)-C(16)-H(16)	106.9	C(24)-C(23)-C(22)	120.1(3)
C(16)-C(17)-H(17A)	109.5	C(24)-C(23)-H(23)	119.9
C(16)-C(17)-H(17B)	109.5	C(23)-C(24)-C(25)	117.3(3)
C(16)-C(17)-C(18)	110.7(3)	C(23)-C(24)-C(27)	121.4(3)
H(17A)-C(17)-H(17B)	108.1	C(25)-C(24)-C(27)	121.1(3)
C(18)-C(17)-H(17A)	109.5	N(5)-C(25)-N(6)	116.2(3)
C(18)-C(17)-H(17B)	109.5	N(5)-C(25)-C(24)	123.4(3)
N(4)-C(18)-C(17)	111.6(2)	N(6)-C(25)-C(24)	120.3(3)
N(4)-C(18)-H(18A)	109.3	N(5)-C(26)-C(22)	123.7(3)
N(4)-C(18)-H(18B)	109.3	N(5)-C(26)-H(26)	118.2
C(17)-C(18)-H(18A)	109.3	C(22)-C(26)-H(26)	118.2
C(17)-C(18)-H(18B)	109.3	C(24)-C(27)-H(27A)	109.5
H(18A)-C(18)-H(18B)	108.0	C(24)-C(27)-H(27B)	109.5
C(16)-C(19)-H(19A)	109.6	C(24)-C(27)-C(28)	110.6(2)
C(16)-C(19)-H(19B)	109.6	H(27A)-C(27)-H(27B)	108.1
C(28)-C(27)-H(27B)	109.5	C(28)-C(27)-H(27A)	109.5
C(27)-C(28)-H(28A)	109.5	H(28A)-C(28)-H(28B)	109.5

C(27)-C(28)-H(28B)	109.5	H(28A)-C(28)-H(28C)	109.5
C(27)-C(28)-H(28C)	109.5	H(28B)-C(28)-H(28C)	109.5

Table SI-6. Anisotropic displacement parameters ($Å^2$x 10^3) for TNG462. The anisotropic displacement factor exponent takes the form: $-2\pi^2[h^2 a^{*2}U^{11} + ... + 2 h k a^* b^* U^{12}]$

	U^{11}	U^{22}	U^{33}	U^{23}	U^{13}	U^{12}
S(1)	26(1)	26(1)	25(1)	3(1)	-3(1)	-1(1)
O(1)	24(1)	30(1)	30(1)	-3(1)	1(1)	0(1)
O(2)	31(1)	37(1)	23(1)	-1(1)	-2(1)	-6(1)
N(1)	22(1)	24(1)	24(1)	-3(1)	-3(1)	1(1)
N(2)	23(1)	24(1)	22(1)	-2(1)	-5(1)	1(1)
N(3)	27(2)	25(1)	21(1)	0(1)	-2(1)	-5(1)
N(4)	22(1)	22(1)	23(1)	1(1)	-5(1)	1(1)
N(5)	32(2)	22(1)	29(1)	1(1)	-4(1)	-2(1)
N(6)	36(2)	24(2)	29(1)	-4(1)	-5(1)	-4(1)
C(1)	20(2)	28(2)	28(2)	-4(1)	-4(1)	2(1)
C(2)	25(2)	42(2)	28(2)	3(2)	1(1)	-5(2)
C(3)	33(2)	33(2)	30(2)	3(2)	-7(1)	-8(2)
C(4)	35(2)	24(2)	31(2)	0(1)	-10(1)	-3(1)
C(5)	24(2)	25(2)	23(2)	-3(1)	-4(1)	2(1)
C(6)	19(2)	24(2)	23(2)	-4(1)	0(1)	2(1)
C(7)	24(2)	21(2)	25(2)	0(1)	1(1)	2(1)
C(8)	20(2)	24(2)	22(1)	-4(1)	-1(1)	2(1)
C(9)	22(2)	24(2)	21(1)	1(1)	3(1)	3(1)
C(10)	24(2)	24(2)	25(1)	-1(1)	2(1)	-4(1)
C(11)	18(2)	24(2)	26(2)	-4(1)	1(1)	-3(1)
C(12)	37(2)	48(2)	28(2)	6(2)	-4(2)	-12(2)
C(13)	26(2)	20(2)	20(1)	4(1)	-2(1)	-1(1)
C(14)	23(2)	25(2)	22(1)	-1(1)	-5(1)	2(1)
C(15)	22(2)	23(2)	24(2)	-3(1)	0(1)	2(1)
C(16)	21(2)	26(2)	23(2)	-1(1)	-2(1)	1(1)
C(17)	25(2)	23(2)	25(1)	4(1)	-5(1)	-1(1)
C(18)	27(2)	28(2)	27(2)	4(1)	-4(1)	-5(1)
C(19)	47(2)	19(2)	37(2)	-2(1)	-18(2)	2(1)
C(20)	37(2)	29(2)	34(2)	-4(1)	-12(2)	12(2)
C(21)	21(2)	36(2)	33(2)	-2(1)	-9(1)	-2(1)
C(22)	24(2)	27(2)	23(2)	-2(1)	-3(1)	-3(1)
C(23)	27(2)	20(1)	27(2)	0(1)	0(1)	-2(1)
C(24)	23(2)	26(2)	24(2)	-2(1)	-1(1)	1(1)
C(25)	24(2)	27(2)	24(2)	-2(1)	0(1)	-1(1)
C(26)	29(2)	27(2)	24(2)	1(1)	-3(1)	1(1)

C(27)	32(2)	24(2)	27(2)	-2(1)	-5(1)	-1(1)
C(28)	30(2)	28(2)	41(2)	-2(2)	-7(1)	0(1)

Table SI-7. Hydrogen coordinates (x 10^4) and isotropic displacement parameters (Å^{2}x 10^3) for TNG462.

	x	y	z	U(eq)
H(3)	880(30)	3990(50)	2372(12)	38(10)
H(6A)	-2330(40)	-100(70)	-163(12)	56(11)
H(6B)	-2030(40)	-1920(40)	320(20)	70(14)
H(1A)	4697	5227	1748	30
H(1B)	5631	5521	2380	30
H(2)	6556	7508	1546	38
H(3A)	6773	9244	2538	38
H(3B)	6431	11021	2004	38
H(4A)	5172	11464	2994	36
H(4B)	4238	11167	2363	36
H(5)	3379	9081	3182	28
H(7)	6258	9951	3730	28
H(10)	5024	3151	4415	29
H(11)	3963	4792	3528	28
H(12A)	4742	7843	837	56
H(12B)	5376	10115	956	56
H(12C)	4054	9464	1334	56
H(16)	10076	7659	5366	28
H(17A)	8375	9975	6220	29
H(17B)	9121	10925	5590	29
H(18A)	11180	10326	6092	33
H(18B)	10293	11737	6573	33
H(19A)	9611	4666	6001	42
H(19B)	8691	6023	6483	42
H(20A)	10800	5620	6968	40
H(20B)	11479	6631	6328	40
H(21A)	12483	9212	7020	45
H(21B)	11772	8285	7662	45
H(21C)	11597	10712	7473	45
H(23)	-36	5304	903	30
H(26)	591	-95	1889	32
H(27A)	-1899	3106	-378	33
H(27B)	-1379	5297	-87	33
H(28A)	-3154	5145	717	50
H(28B)	-3720	3150	327	50
H(28C)	-3668	5399	-27	50

Table SI-8. Hydrogen bonds for TNG462 [Å and °].

D-H...A	d(D-H)	d(H...A)	d(D...A)	<(DHA)
N(3)-H(3)...N(4)#1	0.92(2)	2.06(3)	2.932(3)	156(3)
N(6)-H(6A)...O(2)#2	0.94(2)	2.19(2)	3.121(3)	172(4)

Symmetry transformations used to generate equivalent atoms:
#1 -x+1,y-1/2,-z+1 #2 -x,y-1/2,-z

BIOCHEMICAL RECOVERY ASSAY TO MEASURE K_i OF TNG462 IN THE ABSENCE AND PRESENCE OF MTA

The biochemical measurement of PRMT5 enzyme activity recovery and calculation of K_i of TNG462 in the absence and presence of MTA follow the methods described in supporting information for TNG908[1]. The H4 peptide concentration was increased from 1 μM to 10 μM to enhance peptide competition.

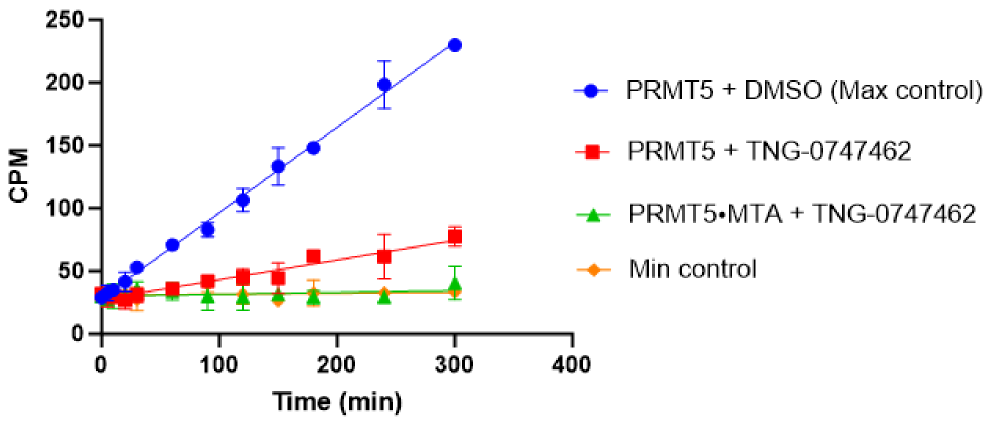

Figure SI-2. Representative progress curves of PRMT5 enzyme activity recovery from PRMT5·TNG462 binary complex (red) and PRMT5·MTA·TNG462 ternary complex (green).

Table SI-9. Calculated K_i values of TNG462 against PRMT5/MEP50 in the absence and presence of MTA

Experiment No.	K_i in the absence of MTA, pM	K_i in the presence of MTA, pM	Fold Change
N=1	3.1	0.3	10.3
N=2	2.2	<0.2	>10
N=3	3.4	<0.2	>10

METHYLTRANSFERASE PANEL DATA FOR TNG462

Table SI-10. TNG462 inhibition profile across 38 methyltransferases

Methyltransferase	Substrate	TNG462, % Enzyme Activity (@ 1 μM)	TNG462, % Enzyme Activity (@ 10 μM)	Control IC_{50} (M)
ASH1L	Nucleosomes	103	97	6.62E-08
DNMT	Poly dl-dC	98	98	2.96E-07
DNMT3a	Lambda DNA	94	98	2.22E-07
DNMT3b	Lambda DNA	96	97	8.96E-08
DNMT3b/3L	Lambda DNA	105	98	3.42E-08

DOT1L	Nucleosomes	93	97	3.80E-07
EZH1 Complex	Core Histone	94	99	9.86E-06
EZH2 Complex	Core Histone	100	97	1.10E-05
EZH2(Y641F) Complex	Core Histone	100	97	4.28E-05
G9a	Histone H3 1-21	109	98	1.03E-06
GLP	Histone H3 1-21	96	96	2.10E-06
MLL1 Complex	Nucleosomes	102	97	8.27E-07
MLL2 Complex	Nucleosomes	97	102	2.85E-05
MLL3 Complex	Nucleosomes	111	102	8.48E-06
MLL4 Complex	Nucleosomes	92	94	2.90E-06
NSD1	Nucleosomes	101	104	6.44E-06
NSD2	Nucleosomes	98	98	5.88E-06
NSD2 (E1099K)	Nucleosomes	107	98	2.16E-06
NSD2 (T1150A)	Nucleosomes	98	97	1.33E-06
NSD3	Nucleosomes	106	95	1.15E-07
PRDM9	Histone H3	108	101	3.72E-06
PRMT1	Histone H4	98	99	1.53E-07
PRMT3	Histone H4	102	93	1.93E-06
PRMT4	Histone H3	109	104	3.81E-07
PRMT5/MEP50 Complex	Histone H2A	7	1	1.11E-06
PRMT5 (C449S) /MEP50 Complex	Histone H2A	4	1	1.01E-06
PRMT6	GST-GAR	107	98	3.37E-07
PRMT7	GST-GAR	97	101	2.04E-07
PRMT8	Histone H4	98	101	1.23E-07
SET1b Complex	Core Histone	93	91	5.01E-06
SET7/9	Core Histone	96	96	1.48E-04
SET8	Nucleosomes	105	104	4.07E-07
SETD2	Nucleosomes	91	95	3.78E-06
SMYD2	Histone H4	103	98	2.85E-07
SMYD3	MEKK2	106	102	1.93E-05
SUV39H1	Histone H3	104	96	8.72E-05
SUV39H2	Histone H3	108	97	2.19E-05
SUV420H1TV2	Nucleosomes	96	97	9.90E-05

EUROFINS SAFETYSCAN PANEL FOR TNG462

Table SI-11. TNG462 E/IC50 ELECT data across 78 assays

Target Class	Assay Name	Assay Target	Mode	Result Type	RC50 (uM)	Max Response
GPCR	Calcium Flux	ADORA2A	Agonist	EC50	>10	0

GPCR	Calcium Flux	ADRA1A	Agonist	EC50	>10	0
GPCR	Calcium Flux	AVPR1A	Agonist	EC50	>10	0.78
GPCR	Calcium Flux	CCKAR	Agonist	EC50	>10	0
GPCR	Calcium Flux	CHRM1	Agonist	EC50	>10	1.22
GPCR	Calcium Flux	CHRM3	Agonist	EC50	>10	0.12
GPCR	Calcium Flux	EDNRA	Agonist	EC50	>10	0.25
GPCR	Calcium Flux	HRH1	Agonist	EC50	>10	0
GPCR	Calcium Flux	HTR2A	Agonist	EC50	>10	0
GPCR	Calcium Flux	HTR2B	Agonist	EC50	>10	0.19
GPCR	Calcium Flux	ADORA2A	Antagonist	IC50	>10	0
GPCR	Calcium Flux	ADRA1A	Antagonist	IC50	6.39	17.27
GPCR	Calcium Flux	AVPR1A	Antagonist	IC50	>10	5.62
GPCR	Calcium Flux	CCKAR	Antagonist	IC50	>10	0
GPCR	Calcium Flux	CHRM1	Antagonist	IC50	7.73	55.88
GPCR	Calcium Flux	CHRM3	Antagonist	IC50	2.08	89.46
GPCR	Calcium Flux	EDNRA	Antagonist	IC50	>10	0
GPCR	Calcium Flux	HRH1	Antagonist	IC50	>10	0
GPCR	Calcium Flux	HTR2A	Antagonist	IC50	>10	30.86
GPCR	Calcium Flux	HTR2B	Antagonist	IC50	>10	9.14
GPCR	cAMP	ADRA2A	Agonist	EC50	1.61	1.78
GPCR	cAMP	ADRB1	Agonist	EC50	>10	0
GPCR	cAMP	ADRB2	Agonist	EC50	>10	0
GPCR	cAMP	CHRM2	Agonist	EC50	>10	0
GPCR	cAMP	CNR1	Agonist	EC50	>10	5.14
GPCR	cAMP	CNR2	Agonist	EC50	>10	0
GPCR	cAMP	DRD1	Agonist	EC50	>10	0
GPCR	cAMP	DRD2S	Agonist	EC50	>10	0
GPCR	cAMP	HRH2	Agonist	EC50	>10	0
GPCR	cAMP	HTR1A	Agonist	EC50	>10	0
GPCR	cAMP	HTR1B	Agonist	EC50	>10	0
GPCR	cAMP	OPRD1	Agonist	EC50	>10	9.48
GPCR	cAMP	OPRK1	Agonist	EC50	>10	0
GPCR	cAMP	OPRM1	Agonist	EC50	>10	0
GPCR	cAMP	ADRA2A	Antagonist	IC50	>10	3.99
GPCR	cAMP	ADRB1	Antagonist	IC50	>10	4.84

GPCR	cAMP	ADRB2	Antago-nist	IC50	>10	9.98
GPCR	cAMP	CHRM2	Antago-nist	IC50	4.98	76
GPCR	cAMP	CNR1	Antago-nist	IC50	>10	12.08
GPCR	cAMP	CNR2	Antago-nist	IC50	5.64	65.65
GPCR	cAMP	DRD1	Antago-nist	IC50	>10	9.93
GPCR	cAMP	DRD2S	Antago-nist	IC50	>10	4.63
GPCR	cAMP	HRH2	Antago-nist	IC50	>10	8.02
GPCR	cAMP	HTR1A	Antago-nist	IC50	>10	0.57
GPCR	cAMP	HTR1B	Antago-nist	IC50	>10	38.94
GPCR	cAMP	OPRD1	Antago-nist	IC50	>10	0.66
GPCR	cAMP	OPRK1	Antago-nist	IC50	>10	0
GPCR	cAMP	OPRM1	Antago-nist	IC50	>10	0
Ion Channel	Ion Channel	CAV1.2	Blocker	IC50	>10	0
Ion Channel	Ion Channel	GABAA	Blocker	IC50	>10	13.24
Ion Channel	Ion Channel	hERG	Blocker	IC50	>10	4.24
Ion Channel	Ion Channel	HTR3A	Blocker	IC50	8.75	53.8
Ion Channel	Ion Channel	KvLQT1/minK	Blocker	IC50	>10	8.48
Ion Channel	Ion Channel	nAChR(a4/b2)	Blocker	IC50	7.99	55.14
Ion Channel	Ion Channel	NAV1.5	Blocker	IC50	>10	12.02
Ion Channel	Ion Channel	NMDAR (1A/2B)	Blocker	IC50	>10	0
Ion Channel	Ion Channel	GABAA	Opener	EC50	>10	5.35
Ion Channel	Ion Channel	HTR3A	Opener	EC50	>10	4.67
Ion Channel	Ion Channel	KvLQT1/minK	Opener	EC50	>10	0.41
Ion Channel	Ion Channel	nAChR(a4/b2)	Opener	EC50	>10	4.11
Ion Channel	Ion Channel	NMDAR (1A/2B)	Opener	EC50	>10	3.44
Kinases	Binding	INSR	Inhibitor	IC50	>10	3.63
Kinases	Binding	LCK	Inhibitor	IC50	>10	1.53
Kinases	Binding	ROCK1	Inhibitor	IC50	>10	9.83
Kinases	Binding	VEGFR2	Inhibitor	IC50	>10	14.29
NHR	NHR Nuclear Translocation	AR	Agonist	EC50	>10	0
NHR	NHR Nuclear Translocation	AR	Antago-nist	IC50	>10	0
NHR	NHR Protein Interaction	GR	Agonist	EC50	>10	0

NHR	NHR Protein Interaction	GR	Antago-nist	IC50	>10	2.55
Non-Kinase Enzymes	Enzymatic	AChE	Inhibitor	IC50	>10	7.21
Non-Kinase Enzymes	Enzymatic	COX1	Inhibitor	IC50	>10	0
Non-Kinase Enzymes	Enzymatic	COX2	Inhibitor	IC50	>10	0
Non-Kinase Enzymes	Enzymatic	MAOA	Inhibitor	IC50	>10	37.76
Non-Kinase Enzymes	Enzymatic	PDE3A	Inhibitor	IC50	>10	0
Non-Kinase Enzymes	Enzymatic	PDE4D2	Inhibitor	IC50	>10	5.98
Transporter	Transporter	DAT	Blocker	IC50	>10	0
Transporter	Transporter	NET	Blocker	IC50	>10	0
Transporter	Transporter	SERT	Blocker	IC50	>10	0

MDCKII AND MDR1-MDCKII ASSAYS.

Wild type (WT) MDCKII cells or MDR1-MDCKII cells (both obtained from Piet Borst at the Netherlands Cancer Institute) were seeded onto the polycarbonate membranes in the 96- well insert system at 4.44 x 10^5 cells/mL and cultured for 4-7 days until confluence before being used for the transport studies. Test compounds were diluted with the transport buffer (HPSS with 10 mM HEPES, pH 7.4) from DMSO stock solution to a concentration of 2 μM (DMSO < 1%) and applied to the apical or basolateral side of the cell monolayer. The plate was incubated for 2.5 h in CO_2 incubator at 37±1 °C, with 5% CO_2 at saturated humidity without shaking. Permeation of the test compounds from A to B or B to A direction was determined in duplicate. In addition, the efflux ratio of each compound was also calculated. For each transport assay, digoxin (P-gp efflux substrate) was tested at 10.0 μM bidirectionally, while nadolol (low permeability marker) and metoprolol (high permeability marker) were tested at 2.00 μM in A to B direction in duplicate. Test and reference compounds were quantified by LC-MS/MS analysis based on the peak area ratio of analyte/internal standard (IS). After transport assay, Lucifer yellow fluorescence rejection assay was performed to confirm the integrity of the cell monolayer.

HUMAN LIVER MICROSOME METABOLIC STABILITY ASSAY.

Test and reference compounds (testosterone, diclofenac and propafenone) at 1 μM were incubated individually in human liver microsome (0.5 mg protein/mL, from mixed-gender donors) supplemented with 1 mM NADPH at 37 °C for 60 minutes while shaking. Aliquots of 60 μl were taken at 5, 15, 30, 45 and 60 minutes of incubation and reactions were stopped by adding 180 ul of quenching solution. After which all sampling plates were shaken for 10 minutes, then centrifuged at 4000 rpm for 20 minutes at 4 °C. Supernatants were transferred to HPLC water (1:3) and mixed for 10 minutes prior to the LC-MS/MS analysis. Test and reference compounds were quantified by LC-MS/MS analysis based on the peak area ratio of analyte/internal standard (IS).

HEPATOCYTE STABILITY ASSAY METHOD

Cryopreserved hepatocytes were thawed, washed, and resuspended in pre-warmed incubation medium to achieve a final concentration of 0.5×10^6 cells/mL. Test compounds and positive controls were prepared as 100 μM dosing solutions in DMSO. In a 96-well plate, 198 μL of hepatocyte suspension was mixed with 2 μL of dosing solution to achieve a final test compound concentration of 1 μM. Plates were incubated at 37 °C in a 5% CO_2 atmosphere with constant shaking (600 rpm). At specified time points (0, 15, 30, 60, and 90 minutes), 20 μL aliquots were transferred to wells containing 100 μL of ice-cold stop solution (acetonitrile with internal standards). Samples were mixed, centrifuged at 3,220 × g for 20 minutes at 4 °C, and supernatants were transferred to pre-labeled plates. Sample analysis was conducted using LC-MS/MS, and the percentage of compound remaining was calculated to assess stability.

KINETIC SOLUBILITY ASSAY.

Medium:
The preparation of 50 mM phosphate buffer (PB) with pH 7.4:
The preparation of 50 mM NaH_2PO_4: Dissolved 3.000 g of NaH_2PO_4 in 500 mL H_2O, and the pH measured was about 4.5.
The preparation of 50 mM NaH_2PO_4: Dissolved 3.549 g of NaH_2PO_4 in 500 mL H_2O, and the pH measured was about 9.4.
The preparation of 50 mM PB (pH 7.4): 15 mL of 50 mM NaH_2PO_4 was added to a 50 mL tube and then adjusted to pH 7.4 +/- 0.05 with 50 mM NaH_2PO_4.

Procedure:
10μL of 10mM DMSO stock solution of test and control compounds was added into each well of a 96-well plate, respectively.
Added 490 μL of medium into the well of the 96-well plate, respectively.
Vortexed the solubility samples for at least 2 minutes.
Shook the 96-well plate on a shaker at room temperature at the speed of 800 rpm for 24h.
Centrifuged at 25 °C for 10 minutes (eq 4000 rpm).
Transferred the supernatant into a filter plate, and then collected the filtrates into a new 96-well plate by centrifuging for at least 5 minutes.
The concentrations of the filtrates were quantified by LC-UV system.

HUMAN ETHER-A-GO-GO-RELATED GENE (HERG) ASSAY.

CHO cells stably expressing hERG potassium channels from Sophion Biosciences were used for this test. The cells were cultured in a humidified and air-controlled (5 % CO_2)

incubator at 37 °C. The CHO cells which were at least two days after plating and more than 75 % confluent would be used for experiments. Before testing, cells were harvested using TrypLE and resuspended in the physiological solution at the room temperature. For the electrophysiological recordings the following solutions were used (Table SI-12).

Table SI-12. Composition of Physiological, External, and Internal Solutions

Reagent	Physiological Solution (mM)	External Solution (mM)	Internal Solution (mM)
NaCl	140	80	10
KCl	4	4	10
KF	-	-	110
$CaCl_2$	2	2	-
$MgCl_2$	1	1	-
Glucose	5	5	-
NMDG	-	60	-
HEPES	10	10	10
EGTA	-	-	10
pH	7.4 with NaOH	7.4 with NaOH	7.4 with KOH
Osmolarity	~298 mOsm	~289 mOsm	~280 mOsm

The physiological solution and external solution were prepared at least one month. The intracellular solution was prepared in batches aliquoted and stored at 4°C until used. Test compounds were dissolved in 100% DMSO to obtain stock solutions for different test concentrations. Then the stock solutions were further diluted into external solution to achieve final concentrations for testing. Visual check for precipitation was conducted before testing. Final DMSO concentration in external solution was not more than 0.30% for the test compounds. Voltage command protocol: From this holding potential of -80 mV, the voltage was first stepped to -50 mV for 80 ms for leak subtraction, and then stepped to +20 mV for 4800 ms to open hERG channels. After that, the voltage was stepped back down to -50 mV for 5000 ms, causing a "rebound" or tail current, which was measured and collected for data analysis. Finally, the voltage was stepped back to the holding potential (-80 mV, 1000 ms). This voltage command protocol was repeated every 20000 msec. This command protocol was performed continuously during the test (vehicle control and test compound). hERG SyncroPatch assay was conducted at room temperature. The Setup, Prime Chip, Catch and Seal Cells, Amplifier Settings, Voltage and Application Protocols were established with Biomek Software (Nanion). One addition of 40 μL of the vehicle was applied, followed by 300s for a baseline period. Then the doses of the compounds were added with 40 μL. The exposure of test compound at each concentration was no less than 300s. The recording for the whole process had to pass the quality control, or the well was abandoned and the compound was retested, all automatically set by PatchControl. Five concentrations (0.30 μM, 1.00 μM, 3.00 μM, 10.00 μM and 30.00 μM) were tested for each compound. A minimum 2 replicates per concentration were obtained. Data analysis was carried out using DataControl, Excel 2013 (Microsoft) and GraphPad Prism 5.0. Within each well recording, percent of control values were calculated for each test compound concentration current response based on peak current in presence of reference control (current response/ peak current) ×100%. The Dose-Response curves were fit to the standard Hill equation as shown below: Ipost cpd/Ipre cpd=Bottom+(Top-Bottom)/(1+10^((LogIC_{50}-X)*HillSlope)) Where X is the logarithm of concentration, Ipost cpd/Ipre cpd is the normalized peak current amplitude, Top is 1 and Bottom is equal to 0. Curve-fitting and IC50 calculations were performed by GraphPad Prism 5.0. If the inhibition obtained at the lowest concentration tested was over 50%, or at the highest concentration tested was less than 50 %, we reported the IC_{50} as less than lowest concentration, or higher than highest concentration, respectively.

IN VIVO PHARMACOKINETIC STUDIES

The animal studies were conducted in accordance with the testing facilities local IACUC guidelines that are in compliance with the Animal Welfare Act, the Guide for the Care and Use of Laboratory Animals.

The plasma pharmacokinetic properties of test compound (TC) were determined following intravenous (IV) bolus administration at a dose of 1 mg/kg in 20% wt/vol HPβCD, 1% vol/vol DMSO in saline and PO administration at 3 mg/kg in 20% wt/vol HPβCD, 1% vol/vol DMSO in water, to male Sprague Dawley rats, or male beagle dogs, or cynomolgus monkeys (fed for IV, fasted for PO). Plasma samples were collected from 3 animals/group at 0.05, 0.25, 0.5, 1, 2, 3, 4, 8, and 24hafter dosing. Concentrations of TC in plasma samples were determined by a liquid chromatography tandem mass spectrometry (LC-MS/MS) method.

PLASMA PROTEIN BINDING PROCEDURE

The binding of TNG462 (2 uM) to cynomolgus monkey plasma protein was measured using the rapid equilibrium dialysis (RED) device per manufacturer's instruction (part number 90,007 (Thermo Fisher Scientific, USA). The incubation was carried out at 37°C for 4 hours. The TNG462 concentration in the donor and receiver chambers was determined by a fit-for-purpose LCMSMS method and the unbound free fraction of TNG462 was calculated as the ratio of receiver chamber over the donor chamber.

PLASMA STABILITY ASSAY METHOD

Pooled frozen plasma was thawed at 37 °C in a water bath and centrifuged at 4,000 rpm for 5 minutes to remove clots. The pH was adjusted to 7.4 ± 0.1 if needed. Test compounds and positive control (propantheline bromide) were prepared as 100 μM dosing solutions by diluting 10 mM stock solutions in DMSO or H_2O. Plasma samples (98 μL) were spiked with 2 μL of dosing solution to achieve a final concentration of 2 μM and incubated at 37 °C. At specified

time points (0, 10, 30, 60, and 120 minutes), 400 µL of stop solution (200 ng/mL tolbutamide and labetalol in acetonitrile) was added to each sample to precipitate proteins. Samples were centrifuged at 4,000 rpm for 15 minutes, and 50 µL of supernatant was mixed with 100 µL of ultrapure water. The mixtures were shaken at 800 rpm for 10 minutes before LC-MS/MS analysis. The percentage of the compound remaining was calculated as % Remaining = 100 × (PAR at incubation time / PAR at T_0), where PAR is the peak area ratio of the analyte to the internal standard.

HAP1 MTAP WT AND MTAP-NULL IN-CELL WESTERN ASSAY

Detailed methods can be found in Cottrell et al., 2024.[1] In brief, the HAP1 *MTAP*-isogenic cell line pair was acquired from Horizon Discovery (HZGHC004894c005) and maintained in DMEM (high glucose) + 10 % FBS in a humidified, 10% CO_2 tissue culture incubator. The SAM-cooperative PRMT5 inhibitor, GSK3326595, was sourced from Selleck Chemicals and maintained as a 10 mM DMSO stock. HAP1 MTAP WT and MTAP-null cells were treated with compounds for 24 h in 384-well microtiter plates, and then normalized SDMA levels were determined using a multi-mAb SDMA antibody (Cell Signaling 13222) and DRAQ5 (LiCor 926-32211 and VWR 10761-508). Background signal was determined by signal from wells treated with 1 µM GSK3326595. Data analysis was performed using the 4-parameter logistic (4-PL) Hill equation with maximal effect constrained to 0. The fit was performed using GraphPad Prism or the default IC_{50} fitting procedure in Dotmatics Studies 5.4 as part of a customized data analysis protocol.

CELL LINE VIABILITY ASSAYS

Detailed methods can be found in Cottrell et al., 2024.[1] In brief, the HAP1 and HCT116 *MTAP*-isogenic cell line pairs were acquired from Horizon Discovery (HZGHC004894c005 and HD R02-033, respectively), the LU99 and LN18 MTAP-isogenic cell line pairs were engineered by stable introduction of a full-length MTAP cDNA under the control of a UbiC promoter. All cell lines were maintained in DMEM (high glucose) + 10% FBS in a humidified, 10% CO_2 tissue culture incubator and confirmed for their MTAP status by immunoblot. Cell viability was determined by CellTiter-Glo following 7-days of compound treatment. Data are plotted as % of the DMSO control wells and fit using a 4-parameter logistic (4-PL) Hill equation with maximal effect or baseline constrained to 0. The fit was performed using GraphPad Prism or the default IC_{50} fitting procedure in Dotmatics Studies 5.4 as part of a customized data analysis protocol. Absolute IC_{50}s are reported for each cell line.

For the 179-cancer cell line panel potency is reported as a relative IC50 as determined by a 4-parameter logistic (4-PL) Hill equation (GraphPad Prism) and selectivity was visualized by plotting the maximum effect (Amax) of TNG462 at 40 nM according to the curve fit.

CELLULAR PRMT5 THERMOSTABILITY ASSAY

The LN18 MTAP-null cell line was either treated with TNG462 for 1 hour and immediately assayed, treated for 1 hour and assayed 72 hours following compound washout and culture in compound-free media, or following 72 hours compound treatment. Treated cells were collected by trypsinization, washed with 1X PBS, and resuspended in a buffer containing 30 mM bicine, 0.003% Tween-20, and 150 mM NaCl. Cells were lysed by 3 freeze/thaw cycles using liquid N_2 and a room temperature water bath. Supernatant was heated to 57°C for 3 minutes and then centrifuged to removed insoluble protein. Immunoblots were run to detect PRMT5 (CST #2252) and densitometry was performed by the Licor analysis tool kit.

IN VIVO PHARMACOLOGY

All protocols for in vivo pharmacology studies were approved by the relevant Institutional Animal Care and Use Committees (Pharmaron, Beijing, China; CrownBio, San Diego, CA, and Taicang and Beijing, China; Champions Oncology, Rockville, MD; XenoSTART, San Antonio, TX; Charles River, Freiburg, Germany) following the guidance of the Association of Assessment and Accreditation of Laboratory Animal Care.

Following acclimatization, LU99 or OCI-LY19 cancer cells were injected subcutaneously into the right flank of 6- to 8-week-old female BALB/c nude mice and allowed to form palpable tumors. Mice were randomized to treatment groups with a mean tumor volume of approximately 200 mm^3 (LU99 efficacy), 300 mm^3 (LU99 PK/PD) or 125 mm^3 (OCI-LY19) in size. TNG462 or TNG908 were formulated in 5% DMA/20% Captisol in water (LU99) or acidified water (pH 4-6; OCI-LY19). TNG908 was formulated in 5% DMA / 20 % Captisol. PDX studies were conducted with similar study designs. Tumor volumes were measured using calipers and calculated as (length x width x width) / 2. For data analysis, tumor growth inhibition % TGI = [1-(Treated TV_{final}-Treated $TV_{initial}$)/(Vehicle TV_{final}-Vehicle $TV_{initial}$)] × 100; tumor regression % TV= [mean TV_{final} – mean $TV_{initial}$] × 100. Tumor volume data were analyzed using GraphPad Prism software.

WESTERN BLOTTING

Protein lysates were generated by lysis of frozen tumor tissue using RIPA buffer. Samples were normalized by protein concentration using Pierce Rapid Gold BCA Protein Assay Kit (A53225). SDS-PAGE was run using Invitrogen NuPAGE 4-12% Bis-Tris Midi Protein Gels (WG1402BOX). Antibodies SDMA (CST#13222), ACTB (CST#3700) were used at 1:1000 dilution.

PRMT5:MEP50 EXPRESSION AND PURIFICATION.

Recombinant PRMT5:MEP50 protein for use in biochemical assays and crystallography work was expressed in insect cells and purified as described previously.[1,2] Purified protein was stored as frozen aliquots at -80 ° C until further use.

PRMT5:MEP50 CRYSTALLOGRAPHY.

Crystal structures of MTA-cooperative inhibitors bound to PRMT5 were routinely obtained by first crystallizing the PRMT5:MEP50 + MTA complex as described previously[1] and then soaking crystals with 1 mM compound at room temperature for between 2 - 5 h. The crystals were flash-cooled prior to data collection using a cryo solution that consisted of mother liquor supplemented with 30 % ethylene glycol. Diffraction data were collected at synchrotron sources as noted in Table SI-13. Data reduction and scaling were performed with XDS and AIMLESS[3,4] and structures were refined using REFMAC from the CCP4 software suite[5]. Model building was done using COOT[6]. Final structural models exhibited good geometry, and all final coordinates and structure factors have been deposited in the PDB with accession codes as noted in Table SI-12.

Table SI-13. X-ray data collection and refinement statistics for PRMT5-MEP50 crystal structures.

Compound	3	14	30	51	TNG462

Cofactor	MTA	MTA	MTA	MTA	MTA
PDB ID	9N3N	9N3O	9N3P	9N3Q	9N3R
**Data Collection**					
Synchrotron source	Spring-8	CLS	Diamond	Spring-8	APS
Beamline	BL45XU	08B1	I03	BL45XU	23-ID-B
Space group	I222	I222	I222	P21212	P21212
Cell dimensions					
a, b, c (Å)	102.5,136.4,178.0	101.8,137.2,176.8	103.0,138.3,177.7	97.4,136.5,178.3	98.2,136.6,178.0
Resolution (Å)	2.75	2.37	2.51	2.54	2.47
Unique reflections	32799	50480	43689	44771	52173
Redundancy	9.5 (9.4)	7.4 (7.3)	13.9 (13.3)	13.4 (11.6)	13.7 (13.9)
Completeness (%)†	100 (100)	100 (99.9)	100 (100)	55.6 (2.8)‡	59.4 (2.7)‡
R_{merge}†	0.174 (1.052)	0.070 (0.779)	0.131 (1.404)	0.183 (0.985)	0.156 (0.778)
I/σ(I)†	10.1 (2.0)	17.4 (2.1)	15.3 (1.8)	11.5 (2.3)	13.3 (3.5)
$CC_{1/2}$	0.995 (0.872)	0.999 (0.849)	0.999 (0.797)	0.998 (0.739)	0.998 (0.931)
**Refinement**					
Reflections used	32789	50470	43676	44728	52128
R_{work}/R_{free}	0.219 / 0.264	0.194 / 0.227	0.194 / 0.223	0.232 / 0.267	0.210 / 0.243
Avg B-value ($Å^2$)	61.9	60.7	65.8	43.6	85.3
Number of atoms					
Protein	7357	7388	7374	14651	14707
Cofactor /Inhibitor	51	51	84	112	114
Solvent/Other	228	405	363	87	116
R.m.s. deviations					
Bond lengths (Å)	0.002	0.002	0.002	0.003	0.002
Bond angles (º)	0.60	0.52	0.48	0.63	0.51

† Values in parentheses are for the highest resolution shell.

‡ Completeness is lower due to anisotropic diffraction correction.

REFERENCES

(1) Cottrell, K. M.; Briggs, K. J.; Whittington, D. A.; Jahic, H.; Ali, J. A.; Davis, C. B.; Gong, S.; Gotur, D.; Gu, L.; McCarren, P.; Tonini, M. R.; Tsai, A.; Wilker, E. W.; Yuan, H.; Zhang, M.; Zhang, W.; Huang, A.; Maxwell, J. P. Discovery of TNG908: A Selective, Brain Penetrant, MTA-Cooperative PRMT5 Inhibitor That Is Synthetically Lethal with MTAP-Deleted Cancers. *J. Med. Chem.* **2024**, *67* (8), 6064–6080. https://doi.org/10.1021/acs.jmedchem.4c00133.

(2) Chan-Penebre, E.; Kuplast, K. G.; Majer, C. R.; Boriack-Sjodin, P. A.; Wigle, T. J.; Johnston, L. D.; Rioux, N.; Munchhof, M. J.; Jin, L.; Jacques, S. L.; West, K. A.; Lingaraj, T.; Stickland, K.; Ribich, S. A.; Raimondi, A.; Scott, M. P.; Waters, N. J.; Pollock, R. M.; Smith, J. J.; Barbash, O.; Pappalardi, M.; Ho, T. F.; Nurse, K.; Oza, K. P.; Gallagher, K. T.; Kruger, R.; Moyer, M. P.; Copeland, R. A.; Chesworth, R.; Duncan, K. W. A Selective Inhibitor of PRMT5 with in Vivo and in Vitro Potency in MCL Models. *Nat. Chem. Biol.* **2015**, *11* (6), 432–437. https://doi.org/10.1038/nchembio.1810.

(3) Kabsch. XDS. *Acta Crystallographica* **2010**, No. d66, 125–132. https://doi.org/10.1107/s0907444909047337.

(4) Evans, P. R.; Murshudov, G. N. How Good Are My Data and What Is the Resolution? *Acta Crystallogr. Sect. D: Biol. Crystallogr.* **2013**, *69* (7), 1204–1214. https://doi.org/10.1107/s0907444913000061.

(5) Murshudov, G. N.; Vagin, A. A.; Dodson, E. J. Refinement of Macromolecular Structures by the Maximum-Likelihood Method. *Acta Crystallogr. Sect. D: Biol. Crystallogr.* **1997**, *53* (3), 240–255. https://doi.org/10.1107/s0907444996012255.

(6) Emsley, P.; Cowtan, K. Coot: Model-Building Tools for Molecular Graphics. *Acta Crystallogr. Sect. D: Biol. Crystallogr.* **2004**, *60* (12), 2126–2132. https://doi.org/10.1107/s0907444904019158.

AUTHOR INFORMATION

Corresponding Author

* **Kevin M. Cottrell** – *Tango Therapeutics, Boston, MA 02215, United States;* Phone: (+1) 857-320-4900; Email: kcottrell@tangotx.com

Authors

Kimberly J. Briggs – *Tango Therapeutics, Boston, MA 02215, United States*
Alice Tsai – *Tango Therapeutics, Boston, MA 02215, United States*
Matthew R. Tonini – *Tango Therapeutics, Boston, MA 02215, United States*
Douglas A. Whittington – Tango Therapeutics, Boston, MA 02215, United States
Shanzhong Gong – *Tango Therapeutics, Boston, MA 02215, United States*
Colin Liang – *Tango Therapeutics, Boston, MA 02215, United States*
Patrick McCarren – *Tango Therapeutics, Boston, MA 02215, United States*
Minjie Zhang – *Tango Therapeutics, Boston, MA 02215, United States*
Wenhai Zhang – *Tango Therapeutics, Boston, MA 02215, United States*
Alan Huang – *Tango Therapeutics, Boston, MA 02215, United States*
John P. Maxwell – *Tango Therapeutics, Boston, MA 02215, United States*

AUTHOR CONTRIBUTIONS

All authors have given approval to the final version of the manuscript.

ACKNOWLEDGMENTS

We thank the following teams/people for their valuable contributions to this work: Oleg Michurin, Tanya Galushka and colleagues (Enamine, Kyiv, Ukraine). Chen Wei, Wan Shuangyi and colleagues (IDSU, WuXi AppTec, China). Shang Deju and colleagues (CSU, WuXi AppTec, Tianjin, China). Jian Shen and colleagues (Viva Biotech, Ltd., Shanghai, China) for X-ray crystallography work. Gang Chen, Xiaoyu Zhu, Kang Yan, and colleagues (WuXi AppTec, Shanghai, China) for peptide displacement and cellular assays. Yuzhou Xu and colleagues (ChemPartner, Shanghai, China) for biochemical characterization studies of TNG462. Yingying Ma, Tan Pang and colleagues (Pharmaron Inc, Beijing, China), Johnnie Flores and colleagues (XenoSTART, Texas), Andrew Layne and colleagues (Champions Oncology, Maryland), Jenny Wu and colleagues (Crown Bioscience, Taicang and Beijing, China), and Romina Walter (Charles River, Freiburg, Germany) for in vivo pharmacology studies. Ying Zhou and colleagues (WuXi AppTec, Shanghai, China) for in vitro ADME and PK studies. Xiling Wang and colleagues (ChemPartner, Shanghai, China) for additional cellular assays. Scott Throner for assistance in manuscript editing. All research described in this manuscript was funded by Tango Therapeutics.

ABBREVIATIONS

MTAP, methylthioadenosine phosphorylase; MTA, methylthioadenosine; SAM, *S*-adenosylmethionine; DMPK, drug metabolism and pharmacokinetics; BBB, blood-brain barrier; HTS, high-throughput screening; CDX, cell line-derived xenograft; PDX, patient-derived xenograft; GI_{50}, growth inhibition 50%; PRMT5, protein arginine methyltransferase 5
